# Assessment model for the justification of intrusive lifestyle interventions: literature study, reasoning and empirical testing

**DOI:** 10.1186/s12910-016-0097-1

**Published:** 2016-02-19

**Authors:** Michiel Wesseling, Lode Wigersma, Gerrit van der Wal

**Affiliations:** Royal Dutch Medical Association (KNMG), Mercatorlaan 1200, 3528 BL Utrecht, Netherlands; Independent health policy advisor, Amsterdam, Netherlands; EMGO+ institute, Department Public and Occupational Health, VU University Medical Center Amsterdam, Amsterdam, Netherlands

**Keywords:** Public health, Lifestyle related diseases, Intrusive interventions, Assessment model, Justification, Freedom, Privacy, Responsibility

## Abstract

**Background:**

In many countries health insurers, employers and especially governments are increasingly using pressure and coercion to enhance healthier lifestyles. For example by ever higher taxes on cigarettes and alcoholic beverages, and ever stricter smoke-free policies. Such interventions can enhance healthier behaviour, but when they become too intrusive, an unfree society can emerge. Which lifestyle interventions that use pressure or coercion are justifiable and which are not? We tried to develop an assessment model that can be used for answering this question, on a generally acceptable way, for all sorts of lifestyle interventions.

**Methods:**

The intended assessment model was developed in three phases. In the first phase the model was theoretically developed on the basis of literature study and reasoning. In the second phase the model was empirically tested by assessing two detailed cases from everyday practice using the model. The model was improved again and again. In the third phase (publication phase) the 10^th^ version of the model was developed while writing this article.

**Results:**

An assessment model for the justification of intrusive lifestyle interventions. It comprises three components: (1) 12 assessment criteria (necessity, causality, responsibility, appropriate design, effectiveness, intrusiveness, burdens-benefits-ratio, fairness, support, complementary policies, verifiability, implementation capacity); (2) an assessment structure with three filters (design logic, effects and side effects, implementation); (3) a way of assessing (based on reasonableness and transparency).

**Conclusions:**

We have developed an assessment model for the justification of lifestyle interventions that use pressure or coercion to promote health. The correctness, completeness and practicality of the model are likely. Important principles for the justification are the logic and completeness of the underlying argumentation and the proper use of the available scientific information. Parties for and against a particular intervention could use the model to test and strengthen their argumentation and to improve the quality of the intervention.

**Electronic supplementary material:**

The online version of this article (doi:10.1186/s12910-016-0097-1) contains supplementary material, which is available to authorized users.

## Background

Lifestyle related diseases currently pose serious threats to public health in developed and developing countries. These are, inter alia, the diseases caused by smoking, lack of exercise, too much or unhealthy eating, alcohol or drug abuse, or unsafe sex [1]^1,2^. The need to combat these unhealthy behaviours creates a dilemma for the government. For health’s sake, the government should intervene in people’s unhealthy lifestyles. Think of the smoking bans in public places, the alcohol excise taxes, and the drug prohibition laws. For the sake of freedom of choice and free trade, the government should not intervene, but allow people to live their unhealthy lives.

Especially in the area of fighting obesity, governments are quite cautious in taking legal action. Potentially effective prevention measures (for instance warnings on packaging, regulations in the area of portion size and packaging size) are rarely taken. And if the government decides to take legislative action, there is often fierce opposition from citizens and businesses [2, 3]. Employers face similar resistance when they try to impose a more healthy lifestyle to employees [4, 5]. As a result, problems arise with the enforcement of lifestyle interventions, taken interventions are withdrawn, and proposed interventions are not applied [6, 7]. Incomplete and selective reasoning by decision makers and lack of evidence for the effectivity of interventions are common reasons for failures in the application of lifestyle interventions.

To address the above mentioned dilemma, the stated incomplete and selective reasoning, and the application of interventions without evidence for the effectivity, we developed a model for systematically identifying and organizing all the arguments, which are relevant for deciding about the application of intrusive lifestyle interventions (Additional file 1) [8]. Intrusive means that interventions use pressure or coercion and restrict freedom or privacy of civilians. The purpose of this model is to promote the quality and transparency of this decision-making and therewith to improve the quality of interventions and diminish resistance against useful interventions.

## Methods

### Development phases

The model was developed [8] in three phases: 2006–2007: design phase (1^st^–6^th^ version of the model), 2008–2012: testing phase (7^th^–9^th^ version of the model) and in 2015: publication phase (10^th^ version of the model).

#### Design phase: theoretical development

The model was theoretically developed on the basis of extensive literature search and reasoning. The literature search comprised research into the criteria, structure and assessment principles (funnel principle [9]^3^, harm principle [10, 11], neutrality principle [12], falsification principle [13], and the principle of accountability for reasonableness [14, 15]) of assessment models in health care, public health, public policy [16], and science. The literature search also focused on examples from everyday practice of lifestyle interventions using pressure or coercion. For the initial design of the model (6^th^ version of the model), nine established assessment models were used: two models for screening [17, 18], three models for priority setting in health care [9, 19, 20], three models for the justification of public health interventions [21–23], one set of recommendations for the design of legitimate public health interventions [24], and one management instrument for effective health promotion [25, 26].

Elsewhere is described in detail, how the nine selected frameworks have led to the 12 criteria of the 6^th^ version of the assessment model [8]. Here we give a brief description. The criteria of the nine selected frameworks were placed into an empty table consisting of columns (vertical) and rows (horizontal) and cells:

- all criteria of the first framework were placed in the first column;
- all criteria of the second framework were placed in the second column, next to the substantively comparable criterion of the first column;
- if the second framework contained a criterion, without a substantively comparable criterion in the first column, for this new criterion a new row was opened;
- if the first column contained a criterion, without a substantively comparable criterion in the second column, the corresponding cell in the second column was left blank;
- and so on.

Then a summary was made of the entire table in which all criteria were included. Thereafter these criteria were placed in a logical order. This resulted in the 6^th^ version of the assessment model.

#### Testing phase: empirical testing

The model was empirically tested, using methods of argumentative text analysis and reasoning. The correctness, completeness and practicality of the model were empirically tested by assessing two cases from everyday practice with the help of the model: the intrusive prevention plan of hospital organization Clarion Health (Additional file 2), and the hotly discussed statutory smoking ban in the Dutch catering sector (Additional file 3). These cases were selected on basis of the following criteria. They concern the prevention of lifestyle-related diseases and pressure or coercion are used to change lifestyles, are of recent date and relevant to today’s society, contain many distinct aspects relevant to the acceptability of interventions that use pressure or coercion and have been the subject of public debate. Furthermore both detailed factual information and detailed information on the views of involved parties on the interventions are available.

The used texts about the cases were representative for the public information and debate about the cases (Additional files 2 and 3) and were carefully screened for arguments for and against the application of the lifestyle interventions.

Per case, all inventoried arguments were placed under the corresponding criteria of the assessment model (some arguments were placed under more than one criterion). If an argument did not fit under any criterion, the wording of a criterion was slightly modified, and if necessary, a new criterion was added; after all the model was intended to accommodate all possible arguments. If the arguments under different criteria had a lot of overlap, it was tried to merge the criteria as much as possible. If new arguments or new criteria were found in literature, the model’s criteria were also adjusted accordingly. The empirical testing of the model by assessing two cases, was especially important for the operationalization of the criteria and way of assessing (see Additional files 4 and 5) and for the investigation of the practicality of the model. During the testing the model was being improved again and again.

#### Publication phase: final version of the model

The 10^th^ version of the model was developed while writing this article. The content of the 10^th^ version is equal to that of the 9^th^ version. Only, the six criteria of the 1^st^ filter in version 9 of the model were merged into four criteria in version 10, so the model is optically even more balanced (each filter now has four criteria). In the period 2013–2015 we have not found publications that should lead to modification of the model.

### Literature search

For the initial design of the model (2006–2007) we extensively searched for literature in the online databases PubMed and PiCarta (PiCarta contains all the online scientific journals and books of more than 400 libraries in the Netherlands), the online publications available from the Health Care Insurance Board, Health Council, Council for Health and Care, and National Institute for Public Health and Environment, in the Netherlands, and the online publications available from investigation committees of the Dutch and British government.

PiCarta proved to be an important addition to PubMed, because various aspects of the model are not within the medical field. All found relevant publications contain a reference list with potentially relevant references. These references were also used to find relevant scientific literature. We used (combinations of) the following search terms in PubMed and PiCarta:

- public health, life style, health behavior, prevent(ion);
- persuasion, coercion, paternalism, responsibility, incentive(s), tax(es);
- human rights, civil rights, ethics, moral;
- autonomy, freedom, liberty, choice behavior, privacy, justice;
- overweight, obesity, tobacco, smoking, alcohol;
- framework, justification;
- review.

From the 157 publications we found with the above search terms [8], we selected frameworks that (1) fully described (2) the justification (3) of similar issues (4) on the basis of criteria. Based on these criteria, we selected nine frameworks.

Also in the testing phase (2008–2013) and publication phase (2015), we regularly checked whether new publications had appeared. The newly found publications in the testing phase were helpful in operationalizing the model’s criteria. The newly found frameworks in the testing phase [27] and publication phase [28–30] did not lead to modification of the model.

## Results

**Fig. 1:**
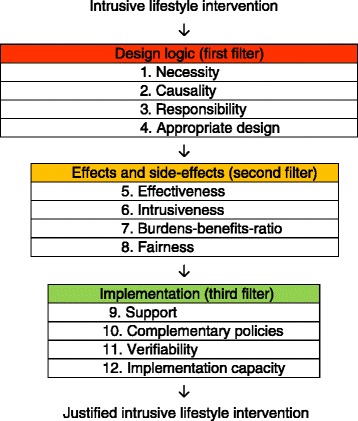
Assessment model for the justification of intrusive lifestyle interventions

The assessment model for the justification of intrusive lifestyle interventions comprises three components: assessment criteria, an assessment structure and a way of assessing (Fig. 1). The model is applicable only to lifestyle interventions that use pressure or coercion.

### Assessment criteria

Whether application of a lifestyle intervention is justified, can be assessed on the basis of twelve criteria. Within the model, these twelve criteria are logically divided into three clusters of four criteria (each cluster functions as a filter). These clusters are: design logic (1^st^ filter), effects and side-effects (2^nd^ filter), and implementation (3^rd^ filter). The meaning of these clusters (filters) is explained later. The twelve criteria should be applied in the given order, because previous criteria provide input for subsequent criteria.

#### Design logic (first filter)

1. *Necessity*
  What risk of harm (e.g., health damage or economic damage), of nuisance (e.g., noise pollution), or of infringement of moral views (e.g., drug use is objectionable) does the lifestyle intervention try to combat? What size are these risks? Who are at risk? To what extent is it necessary to combat these risks? Striving for an optimal situation (e.g., optimal health) is perfectionistic. The imposition of moral beliefs to others (e.g., promiscuity is objectionable) is moralistic. To what extent is the lifestyle intervention perfectionistic or moralistic and is this justifiable?
2. *Causality*
  What are the determining factors of the risks the lifestyle intervention tries to combat? To what extent do the lifestyles targeted by the intervention really determine the risks that the intervention tries to combat?
3. *Responsibility*
  Which parties are held responsible for creating or maintaining the risks the lifestyle intervention tries to combat? To what extent is it logical to hold these parties responsible, taking into account the determining factors of the risks (2^nd^ criterion)? Protecting competent adults against themselves is paternalistic. To what extent is the lifestyle intervention paternalistic and is this justifiable?
4. *Appropriate design*
  Are the target group and ‘the life-style influencing factors targeted by the intervention’ logically chosen, taking into account the determining factors of the risks (2^nd^ criterion) and the division of responsibilities (3^rd^ criterion)? How many people within the target group are missed and how many outside the target group are hit by the intervention? Is this justifiable? Are the parties implementing the intervention competent, authorised and suitable? Will implementation of the intervention not harm the nature or reputation of the parties implementing the intervention?

#### Effects and side-effects (second filter)

5. *Effectiveness*
  To what extent are the intended effects of the lifestyle intervention achieved and to what extent are these effects sustainable?
6. *Intrusiveness*
  To what extent forms the lifestyle intervention an intrusion in private life by infringement of physical integrity (e.g., blood tests to determine drug use), violation of freedom (of choice), infringement of privacy, infringement of perceived safety, discrimination, or stigmatisation? Is the ratio of the effectiveness (5^th^ criterion) and intrusiveness (6^th^ criterion) acceptable?
7. *Burdens-benefits-ratio*
  Is the ratio of the burdens (intrusiveness, costs, other negative side effects) and benefits (effects and positive side effects) justifiable? How will this ratio develop in time?
8. *Fairness*
  Are the burdens and benefits (7^th^ criterion) distributed fairly across parties (for example in proportion to their responsibility (3^rd^ criterion)? Are people treated equally? Are some individuals or organizations not disproportionately affected by the measure? Does the lifestyle intervention not violate the law or legally effective agreements?

#### Implementation (third filter)

9. *Support*
  Is the time ripe to apply pressure or coercion? To what extent is tried to acquire support for the lifestyle intervention? Is there sufficient support for the intervention, so that the implementation is not unduly difficult or expensive?
10. *Complementary policies*
  Has the lifestyle intervention been anchored in broader prevention or other policies? How are information provision and enforcement of compliance with the intervention organised? Do parties, who are disproportionately affected by the intervention, receive (for example financial) compensation?
11. *Verifiability*
  Is the lifestyle intervention adequately monitored and evaluated so that adjustments can be made if necessary?
12. *Implementation capacity*
  Have a sufficient number of people and funds been made available to ensure that implementation of the lifestyle intervention is feasible and sustainable?

### Assessment structure

The assessment model has the structure of a ‘funnel’ with the three successive filters: design logic, effects and side effects, and implementation (Fig. 1). The 1^st^, 2^nd^ and 3^rd^ filter cover different aspects of the same question, namely whether the application of the preventive measure is justified. The 1^st^ filter concerns the design logic, regardless of the effects and side-effects (2^nd^ filter). The 2^nd^ filter regards the acceptability of the effects and side effects, regardless of the quality of the implementation (3^rd^ filter). It happens that a proper design (1^st^ filter) still has unintended side effects (2^nd^ filter). It is also possible that the effects and side-effects (2^nd^ filter) are acceptable, while the implementation (3^rd^ filter) is carried out suboptimal. For example with better enforcement of compliance (10^th^ criterion in the 3^rd^ filter) the effectiveness (5^th^ criterion in the 2^nd^ filter) could be even more favourable.

The first reason for using the ‘funnel’ structure is that the sequence in which the assessment criteria in the model are used has a huge impact on the content and consequently on the validity of the assessment process. The second reason is that the successive filters make it easier to manage the total number of 12 criteria. If the intervention fails to pass through a certain filter, the underlying criteria need no longer be examined. Thirdly, the criteria within one filter may be weighed against each other. This means that a good score for a certain criterion can compensate for a poor score on another criterion. The criteria originating from different filters are not permitted to be weighed against each other. The following examples illustrate this: if the intervention is unnecessary (1^st^ filter), it is irrelevant to subsequently assess effectiveness (2^nd^ filter); if the intervention is ineffective (2^nd^ filter), implementation of the intervention no longer applies (3^rd^ filter).

### Way of assessing

#### Argumentative plea

The justification of a prevention measure using the model is similar to the assessment of the guilt of a suspect by a judge. The assessment is not an arithmetic exercise, but an argumentative plea. If the arguments on all criteria of a filter are weak, an overall negative judgement on that filter is more likely, than if the arguments on only one criterion are weak and strong on the other criteria. In this way, the (arguments of) different criteria can be weighed against each other.

#### Assessment based on reasonableness and transparency

Whether application of a lifestyle intervention is justified, is assessed on the basis of the principle ‘accountability for reasonableness’ [15, 16]. Daniels introduced this principle for priority setting in health care. If no consensus can be reached on the proper weighing of values and norms for making choices, choices should (partly) be made on the basis of moral or ideological views. That choices in health care are based on moral or ideological views, is considered as inevitable and not as unreasonable. However, one must provide maximum transparency about the views and reasoning that underlie the choice. So that a fair process allows to agree on what is legitimate and fair [15, 16]. The justification of an intrusive lifestyle intervention also depends on ideological views on, for example: own responsibility for own health; government’s responsibility for health; the importance of health, freedom of choice, privacy, etc. If people have different views on what is more or less important, the principle of ‘accountability for reasonableness’ is a good way to deal with this.

#### Assessing the reasonableness

Per criterion of the model it is assessed whether the reasoning that underlies the application of the lifestyle intervention meets the following requirements of reasonableness: *(1) correctness* (the reasoning isn’t in conflict with scientific information and isn’t illogical); *(2) completeness* (no relevant arguments or available scientific information are overlooked or ignored); *(3) robustness* (the evidence for the correctness of the reasoning is sufficiently strong); *(4) internal consistency* (the reasoning about a particular criterion is consistent with the reasoning about the previous criteria of the model); *(5) fairness* (the reasoning is not unfair); *(6) optimization* (it is plausible that in the given circumstances there are no better alternatives for the design or implementation of the lifestyle intervention).

#### Assessing the transparency

Assessed is whether the responsible parties have made enough effort to provide transparency about the lifestyle intervention in relation to all criteria of the model. When responsible parties have made insufficient effort to provide this transparency, application of the lifestyle intervention isn’t justified. In the context of the model, which focusses on *intrusive* lifestyle interventions, transparency is a condition sine qua non. If the right things are done for the wrong reasons (1^st^ filter), the argumentation underlying the intervention can be altered and then the intervention is still justified. It is not fair to delude people with false arguments or by withholding arguments. In time, deluding people will undermine the rule of law and democracy [23, 24].

## Discussion

### Correctness, completeness and practicality of the model

Nine different established assessment models were used to build the assessment model. These models have a partially different objective. For each model - of which one is based on an international study that compared models which were developed in different countries [20] it was examined whether the criteria, structure and way of assessing could be used for the development of our model. This method to a certain extent guarantees the ‘theoretical’ completeness of the model. The developed model was empirically tested by its application to two recent cases from everyday practice (Additional files 2 and 3). The used texts about these cases were representative for the public information and public debate about these cases. The successful application of the model to these cases is a strong indication of the correctness, completeness and practicality of the model.

The claim of correctness of the model does not relate to the correctness of the statements made by the assessors when they use the model, but to the appropriateness of the model to apply the principle of ‘accountability for reasonableness' in a proper way. The two assessments of practical cases (Clarian Health and smoking ban), based on the requirements of reasonableness and transparency, make this plausible.

The criteria of the model are exhaustive, meaning that they intend to include all possible arguments, for and against the introduction of all possible prevention measures.

### Assessment based on the falsification principle

It is hard to prove the correctness of a theory. For this, the correctness of all parts of the theory must be proven. It is much easier to prove the incorrectness of a theory. For this, the incorrectness of just one part of the theory must be proven. Science can especially make progress by searching for falsification of existing theories. If a falsification is found, the theory can be modified or rejected [13, 14].

During the assessment of the cases, it became clear, that for many criteria it was (nearly) impossible to reach a generally acceptable judgement without using the falsification principle. For example, how logical needs a design to be (1^st^ filter), before it is justified? In general it is easier to determine that there are no convincing arguments against a proposition or theory (no convincing falsification) than to determine that a proposition or theory are correct. In this example, this can be done by checking that it is not possible to come up with a more logical design.

For all criteria of the model, the assessor can try to prove that the lifestyle intervention doesn’t meet the requirements of reasonableness and transparency discussed above. The statement that application of the intervention is justified on the basis of the model, then means that the assessor is unable to demonstrate the unreasonableness of or lack of transparency about the intervention. Possibly more frequently the unreasonableness of, or lack of transparency about, certain parts of the lifestyle intervention will be demonstrated. In response, the intervention can be improved, more information about the intervention can be made available, or the intervention can be rejected.

### Acceptability of the ‘way of assessing’

We think that the principles on which the assessments are based, reasonableness and transparency (and if necessary falsification), are generally acceptable, which in this context means that these principles are acceptable for the parties involved by the lifestyle intervention.

### Scope and future assessments

When justifying prevention measures, distinction can be made between the argumentative (substantive) justification and procedural justification. We developed an argumentative model. Criteria relating to the procedural justice (public participation, decision-making procedures, information procedures, etc.) are outside the scope of the model.

The scope of the model is further limited to situations which seek to influence the lifestyle or behaviour of people with pressure or coercion. If people willingly accept a particular intervention (there is informed consent) they are individually considering whether the effectiveness of the prevention measure for them outweighs the intrusiveness, and the model is not applicable (and not necessary). It varies from person to person whether or not lifestyle influences are perceived as intrusive. In many cases there will be consensus between parties whether or not a certain lifestyle intervention is intrusive. If no consensus exists, parties may discuss this using the various aspects of the criterion ‘intrusiveness’ of the model (see Additional file 4). So, it is not absolute whether or not a prevention measure falls within the scope of the model. This is determined by the parties concerned.

Repeated assessment of the same cases by new assessors and assessment of new cases can further strengthen the evidence for the correctness, completeness, practicality and acceptability of the model.

The development of the model has taken place entirely within the health context. We suppose that the model can also be applied to lifestyle interventions outside the health sector. It seems worthwhile to experiment herewith.

### Retrospective and prospective assessments

For a retrospective assessment (evaluation) and prospective assessment (prediction) the same criteria can be used.

A retrospective assessment looks back to: the design (1^st^ filter) as it was actually carried out, the effects and side effects (2^nd^ filter) as they actually occurred, and the implementation (3^rd^ filter) as it was actually carried out. That’s why a retrospective assessment can only take place during or after the implementation of a prevention measure. In case of a retrospective assessment the impact of the implementation (3^rd^ filter) is automatically processed in the design (1^st^ filter) and the effects and side effects (2^nd^ filter).

A prospective assessment (prediction) looks forward to: the design (1^st^ filter) that one intends to implement, the effects and side effects (2^nd^ filter) that should occur yet, and the implementation (3^rd^ filter) that one intends to carry out. A prospective assessment can only be based on assumptions about the ‘design’, the ‘effects and side effects’ and the ‘implementation’. Working with assumptions offers the possibility to work with implementation scenarios (for example, strict versus limited enforcement of regulations). It is also possible to try out and investigate the ‘effects and side effects’ of a prevention measure in a controlled and/or defined environment with the aid of experiments or pilot studies.

## Conclusions

Based on literature, reasoning and empirical testing we developed an assessment model for the justification of lifestyle interventions that use pressure or coercion to promote health. The correctness, completeness and practicality of the model are likely, in particular based on extensively testing the model on two detailed cases from everyday practice. A strong feature of the model is the general acceptability of the ‘way of assessing’, achieved by basing the assessment on the generally accepted principle of accountability for reasonableness (reasonableness and transparency). In our opinion the model is usable by public and private parties for designing and examining of intrusive lifestyle interventions.

## Ethics statement

Our study had no human participants. Our study doesn’t include health information or other sensitive information that can be traced to individuals. The arguments and opinions of persons cited in the study, were obtained by visiting publicly accessible, legitimate websites on the Internet.

## Endnotes

^1^WHO. Global strategy on diet, physical activity and health. Programme of the World Health Organization. Geneva, 2003 - present.

^2^WHO. Tobacco Free Initiative. Programme of the World Health Organization. Geneva, 1998 - present.

^3^The funnel principle is explained in the section on the final assessment structure.

^4^McGregor J. Being unhealthy could cost you money. BusinessWeek. 2007 August 2. Article: 1282 words.

^5^Wojcik J. Workforce Management. Employer to fine unhealthy workers. 2007 August. Article: 920 words.

^6^NBC5. Employer to obese employees: shape up or pay up. Indiana company charging employers who smoke, are obese. 2007 Augustus 10. News report: 527 words.

^7^FoxNews.com. Company to charge ‘unhealthy’ workers more for insurance. 2007 July 1. News report: 453 words.

^8^Big Fat Blog. Clarian Health, Others will dock your paycheck if you’re fat. 2007 August 8–2007 September 9. ‘The fat acceptance weblog’: 32 reactions from visitors; 10.129 words.

^9^MSNBC, TodayShow. Company fines workers for being overweight. 2007 August 10. ‘The allDay weblog’: 35 reactions from visitors; 6.113 words.

^10^Website of the Dutch Ministry of Health, Welfare and Sport: all publications about smoking (2007 May - 2011 June).

^11^Website of the Dutch government: all parliamentary publications found with the search terms ‘tobacco control’ and ‘smoke’, and all laws and legal regulations found with the search term ‘Tobacco’ (1986 March - 2011 June). http://www.overheid.nl/english.

^12^Website of the Netherlands Food and Consumer Product Safety Authority: all reports on the compliance level of the smoking ban in the Dutch catering sector (2008–2011).

^13^Website of the Royal Library - National library of the Netherlands: some documents from the design period of the Dutch Tobacco Act (1984).

^14^Fourteen Dutch studies commissioned by the Dutch government to support the smoking policy in the Dutch catering sector (2007–2010).

## Additional files

Additional file 1:
**Dissertation in Dutch.** Summary in English-p.515-535. (PDF 1986 kb) Additional file 2:
**Assessment of the intrusive prevention plan of Clarian Health** ^**4–9**^ **.** (DOCX 18 kb) Additional file 3:
**Assessment of the smoking ban in the Dutch catering sector** **[** 31 **–** 35 **]** ^**10–14**^ **.** (DOCX 17 kb) Additional file 4:
**Operationalization of the criteria and assessment procedure, and adjustments to the model, based on the assessment of the Clarian Health case.** (DOCX 42 kb) Additional file 5:
**Operationalization of the criteria and assessment procedure, and adjustments to the model, based on the assessment of the Smoking Ban case.** (DOCX 26 kb)
