# Supplementary material for: Assessment model for the justification of intrusive lifestyle interventions: literature study, reasoning and empirical testing
Source: BMC Med Ethics. 2016 Feb 19;17:14. doi: 10.1186/s12910-016-0097-1 (PMC4759762; doi:10.1186/s12910-016-0097-1)
Supplement: Additional file 1: — Dissertation in Dutch. Summary in English-p.515-535. (PDF 1986 kb) [file 12910_2016_97_MOESM1_ESM.pdf]

VRIJE UNIVERSITEIT

Gerechtvaardigdheid van interventies ter beïnvloeding van leefstijl

Een waardeneutraal beoordelingsmodel

ACADEMISCH PROEFSCHRIFT

ter verkrijging van de graad Doctor aan
de Vrije Universiteit Amsterdam,
op gezag van de rector magnificus
prof.dr. L.M. Bouter,
in het openbaar te verdedigen
ten overstaan van de promotiecommissie
van de Faculteit der Geneeskunde
op woensdag 5 december 2012 om 13.45 uur
in de aula van de universiteit,
De Boelelaan 1105

door

Michiel Frans Wesseling

geboren te Maassluis

promotor:	prof. dr. G. van der Wal
copromotor:	dr. L. Wigersma

Gerechtvaardigdheid van interventies ter beïnvloeding van leefstijl

Een waardeneutraal beoordelingsmodel

Michiel Wesseling

VU University Press

VU University Press is een imprint van

VU Boekhandel / Uitgeverij bv
De Boelelaan 1105
1081 HV Amsterdam
The Netherlands

info@vu-uitgeverij.nl
www.vuuitgeverij.nl

© 2012 Michiel Wesseling

Verzorging binnenwerk: JAPES, Amsterdam (Jaap Prummel)
Verzorging omslag: VU Dienst Communicatie (Heleen ten Voorde)

ISBN 978 90 8659 623 2
NUR 770 / 775

All rights reserved. No part of this book may be reproduced, stored in a retrieval system, or transmitted, in any form or by any means, electronic, mechanical, photocopying, recording, or otherwise, without the prior written consent of the publisher.

Inhoudsopgave

1. Inleiding 9

1.1. Bestrijding van leefstijlgerelateerde aandoeningen 9
1.2. Probleemstelling en onderzoeksvraag 11
1.3. Focus op gerechtvaardigdheid van de argumentatie 13
1.4. Onderzoeksaanpak 14
1.5. Onderzoeksmethoden 17
1.6. Discussie 19

2. Criteria van het beoordelingsmodel (versie 1 t/m 6 van het model) 21

2.1. Ontwikkeling van de criteria van het beoordelingsmodel 21
2.2. Eerste versie van het beoordelingsmodel 22
2.3. Tweede versie van het beoordelingsmodel 28
2.4. Derde versie van het beoordelingsmodel 31
2.5. Vierde versie van het beoordelingsmodel 33
2.6. Vijfde versie van het beoordelingsmodel 34
2.7. Zesde versie van het beoordelingsmodel 56

3. Structuur van het beoordelingsmodel (versie 6 van het model) 75

3.1. Volgorde en werking van de beoordelingszeven 75
3.2. Rol van denkkaders bij de beoordeling 79
3.3. Fasering van de argumentatie 80

4. Wijze van beoordeling bij het model (versie 6 van het model) 89

4.1. Inleiding 89
4.2. Transparantie over de argumentatie die aan besluiten ten grondslag ligt 91
4.3. Gebruik van redeneringen die voor iedereen aanvaardbaar zijn 93
4.4. Herzien van besluiten als de onderbouwing onder druk staat 104
4.5. Samenvatting van de wijze van beoordeling 104

5. Inleiding bij casus 'Clarian Health' (versie 6 van het model) 107

6. Toetsing 1e zeef van het beoordelingsmodel aan casus 'Clarian Health' (versie 6 van het model) 111

6.1. Eerste criterium: schadelijkheid 111
6.2. Tweede criterium: noodzakelijkheid 120
6.3. Derde criterium: oorzakelijkheid 126
6.4. Vierde criterium: verantwoordelijkheid 131
6.5. Vijfde criterium: gerichtheid 141

6.6. Zesde criterium: geschiktheid 144
6.7. Eindoordeel over de 1e zeef: logica van het ontwerp 150

7. Toetsing 2e zeef van het beoordelingsmodel aan casus 'Clarian Health' (versie 6 van het model) 153
7.1. Zevende criterium: effectiviteit 153
7.2. Achtste criterium: indringendheid 159
7.3. Negende criterium: kosteneffectiviteit 187
7.4. Tiende criterium: lasten-batenverhouding 188
7.5. Elfde criterium: rechtvaardigheid 194
7.6. Twaalfde criterium: rechtsgelijkheid 203
7.7. Eindoordeel over de 2e zeef: effecten en neveneffecten 208

8. Toetsing 3e zeef van het beoordelingsmodel aan casus 'Clarian Health' (versie 6 van het model) 211
8.1. Dertiende criterium: draagvlak 211
8.2. Veertiende criterium: uitvoerbaarheid 217
8.3. Eindoordeel over de 3e zeef: implementatie 218

9. Conclusies van toetsing aan casus 'Clarian Health' (versie 7 van het model) 219
9.1. Eindoordeel over de gerechtvaardigdheid van het preventieplan van Clarian Health 219
9.2. Oordeel over de juistheid, volledigheid en praktische toepasbaarheid van het model 221

10. Inleiding bij casus 'wettelijk rookverbod in horeca' (versie 8 van het model) 225
10.1. Inleiding 225
10.2. Literatuuronderzoek naar wettelijk rookverbod in de horeca 227
10.3. Totstandkoming van het wettelijk rookverbod in de horeca 229
10.4. Aanpassing van het model naar aanleiding van het voorbereidende literatuuronderzoek 242
10.5. Onverwachte problemen bij toepassing van het model op casus 'wettelijk rookverbod in horeca' 246
10.6. Beoordeling van de gerechtvaardigdheid van de doelstelling van het wettelijk rookverbod in de horeca 253

11. Toetsing 1e zeef van het beoordelingsmodel aan casus 'wettelijk rookverbod in horeca' (versie 8 van het model) 263
11.1. Eerste criterium: schadelijkheid 263
11.2. Tweede criterium: noodzakelijkheid 281
11.3. Derde criterium: oorzakelijkheid 289
11.4. Vierde criterium: verantwoordelijkheid 291
11.5. Vijfde criterium: gerichtheid 295

11.6. Zesde criterium: geschiktheid 299
11.7. Eindoordeel over de 1e zeef: logica van het ontwerp 309

12. Toetsing 2e zeef van het beoordelingsmodel aan casus 'wettelijk rookverbod in horeca' (versie 8 van het model) 313
12.1. Zevende criterium: effectiviteit 313
12.2. Achtste criterium: indringendheid 332
12.3. Negende criterium: lasten-batenverhouding 340
12.4. Tiende criterium: rechtvaardigheid 354
12.5. Eindoordeel over de 2e zeef: effecten en neveneffecten 381

13. Toetsing 3e zeef van het beoordelingsmodel aan casus 'wettelijk rookverbod in horeca' (versie 8 van het model) 385
13.1. Elfde criterium: timing 385
13.2. Twaalfde criterium: draagvlak 395
13.3. Dertiende criterium: flankerend beleid 405
13.4. Veertiende criterium: handhaafbaarheid 414
13.5. Vijftiende criterium: controleerbaarheid 425
13.6. Zestiende criterium: uitvoerbaarheid 429
13.7. Eindoordeel over de 3e zeef: implementatie 434

14. Conclusies van toetsing aan casus 'wettelijk rookverbod in horeca' (versie 9 van het model) 445
14.1. Eindoordeel over de gerechtvaardigdheid van het wettelijk rookverbod in de horeca 445
14.2. Oordeel over de juistheid, volledigheid en praktische toepasbaarheid van het model 450

15. Discussie 453
15.1. Beantwoording van de onderzoeksvraag door presentatie van het model 453
15.2. Beschouwing van mijn onderzoek en beoordelingsmodel 475
15.3. Relevantie van mijn onderzoek en beoordelingsmodel 487

Samenvatting 493
I. Inleiding 493
II. Beschrijving van het onderzoek 494
III. Beoordeling van de casuïstiek 497
IV. Presentatie van het beoordelingsmodel 501
V. Samenvatting van de discussie 505

Summary 515
I. Introduction 515
II. Research description 516
III. Assessment of cases from day-to-day practice 519

IV. Presentation of the assessment model 523
V. Consideration of the research and assessment model 534

Bijlagen

1. Toelichting van begrippen (Brug, 2004) 537

2. Categorieën van preventiemaatregelen gecombineerd 541

3. Indeling van preventiemaatregelen naar doelgroep 549

4. Criteria van andere beoordelingsmodellen 551

5. Definiëring van het begrip 'schade' ten behoeve van het beoordelingsmodel 557

6. Voorbeelden van tegengestelde denkkaders (a versus b) 565

7. Artikel over casus Clarian Health in BusinessWeek 571

8. Toelichting op paragraaf f van HIPAA 575

9. Sociaaleconomische gezondheidsverschillen samengevat (Lucht, 2009) 577

10. Rapport van TNS NIPO biedt geen uitsluitsel over economische effecten van wettelijk rookverbod voor kleine cafés 579

Literatuur 587

Dankwoord 597

Curriculum vitae 599

1 Inleiding

1.1 Bestrijding van leefstijlgerelateerde aandoeningen

Leefstijlgerelateerde aandoeningen vormen wereldwijd, zowel in relatieve als absolute zin, een toenemend gevaar voor de volksgezondheid. In relatieve zin, omdat de meeste infectieziekten, die in het verleden de grootste bedreiging voor de volksgezondheid vormden, tegenwoordig met succes kunnen worden bestreden. In absolute zin, omdat, mede ten gevolge van de toenemende welvaart, een groot deel van de bevolking steeds ongezonder leeft (Scholtmeijer, 2006, p.219) (IFIC, 2007). Bij een ongezonde leefstijl kan in dit verband met name worden gedacht aan roken, te veel en ongezonde voeding, te weinig bewegen en overmatig alcoholgebruik. Nationale en internationale overheden onderkennen de toenemende bedreiging van een ongezonde leefstijl voor de volksgezondheid en trachten deze trend te keren door het treffen van preventieve maatregelen (Rabin, 2006, p.53), (Haby, 2006, p.1463). Zowel de World Health Organization als de Europese Commissie heeft het terugdringen van roken (WHO, 2007) (EC, 2007) en overgewicht (WHO, 2006) (EC, 2005) vastgesteld als speerpunten in respectievelijk het mondiale en Europese volksgezondheidbeleid.[1]

De essentie van leefstijlgerelateerde aandoeningen is dat het ontstaan van deze aandoeningen in belangrijke mate is toe te schrijven aan 'vermijdbaar' gedrag van mensen. Het woord 'vermijdbaar' is tussen aanhalingstekens geplaatst, omdat het, bijvoorbeeld voor mensen die verslaafd zijn aan tabak of alcohol, heel moeilijk blijkt te zijn om niet aan hun verslaving toe te geven. Ook kan het zijn dat mensen bepaald gedrag, zoals voldoende bewegen, juist te weinig vertonen. Ten slotte is het de vraag of ongezond gedrag gezien moet worden als abnormaal gedrag in een normale c.q. gezonde omgeving, of dat ongezond gedrag gezien moet worden als normaal gedrag in een abnormale c.q. ongezonde omgeving. Deze laatste visie wordt door veel wetenschappers, die trachten obesitas te bestrijden, aangehangen. Zij schrijven over de 'obesogene omgeving' die mensen aanzet tot te veel en ongezond eten en te weinig bewegen (zie bijlage 6). Hoewel het oorzakelijk verband tussen bepaalde gedragingen en bepaalde aandoeningen nog niet volledig is opge-

1. *"Voor gezondheidsbevordering zijn vier beleidskaders van belang: [1] International beleid zoals dat via de UN, de WTO, WHO en EU wordt vormgegeven ..."* (Saan, 2005, p.62)

helderd, is wel duidelijk dat gedragsverandering het aantal ziektegevallen sterk kan beperken. Dit inzicht biedt goede aanknopingspunten voor preventieve interventies, maar roept ook vragen op over de gerechtvaardigdheid van deze interventies, omdat zij tot doel hebben om het gedrag van mensen te veranderen. Hieronder zal ik kort op enkele vragen ingaan.

In de eerste plaats vraagt de preventie van leefstijlgerelateerde aandoeningen om een interventie in de leefstijl van mensen, bij voorkeur ruim voordat zij ten gevolge van hun leefstijl ziek worden. Dergelijke interventies beperken de vrijheid van mensen om een eigen leefstijl te kiezen. Is het bijvoorbeeld niet de eigen verantwoordelijkheid van mensen of zij ongezond eten of roken? In hoeverre mogen bijvoorbeeld overheden en werkgevers druk uitoefenen op respectievelijk de bevolking en werknemers om van leefstijl te veranderen? Volgens de Universele Verklaring van de Rechten van de Mens is een beperking van de vrijheid van mensen slechts in bepaalde gevallen te rechtvaardigen (VN, 1948, art. 29.2)[2]:

> *"In de uitoefening van zijn rechten en vrijheden zal een ieder slechts onderworpen zijn aan die beperkingen, welke bij de wet zijn vastgesteld en wel uitsluitend ter verzekering van de onmisbare erkenning en eerbiediging van de rechten en vrijheden van anderen en om te voldoen aan de gerechtvaardigde eisen van de moraliteit, de openbare orde en het algemeen welzijn in een democratische gemeenschap."*

Het is de vraag in hoeverre preventiemaatregelen ter bestrijding van leefstijlgerelateerde aandoeningen al dan niet strijdig zijn met het mensenrecht op (keuze)vrijheid, zoals hierboven verwoord. In de tweede plaats beogen preventiemaatregelen ter bestrijding van leefstijlgerelateerde aandoeningen veelal de consumptie van bepaalde levens- en genotsmiddelen (sigaretten, frisdrank, snacks, alcohol) te verminderen. Dergelijke maatregelen kunnen het financiële belang van de levens- en genotsmiddelenindustrie schaden. Bovendien vormen zij een schending van het recht op het vrije verkeer van goederen en diensten, dat is vastgelegd in diverse internationale verdragen (EG, 2002)[3]. In hoeverre is dat aanvaardbaar? (Grondwet)[4], [5].

2. www.overheid.nl > Wet- en regelgeving > Verdragen.
3. Het recht op het vrije verkeer van goederen en diensten is vastgelegd in het verdrag van de Europese Gemeenschap (EG, 2002, artikel 23-31 en 49-55).
4. Strikt genomen vormt een preventiemaatregel, die het maken van reclame voor ongezonde producten aan banden legt, ook een inperking van het recht op persvrijheid, dat in de grondwet van veel landen is vastgelegd. De Nederlandse grondwet stelt echter in artikel 7.4 dat de grondwetsartikelen over het recht op persvrijheid (artikel 7.1-7.3) niet van toepassing zijn op het maken van handelsreclame.
5. Grondwet. Geldend op 7 juli 2010. www.overheid.nl

1.2 Probleemstelling en onderzoeksvraag

De risico's van preventiemaatregelen ter bestrijding van leefstijlgerelateerde aandoeningen voor de keuzevrijheid van de bevolking, voor de financiële belangen van het bedrijfsleven en voor het vrije verkeer van goederen en diensten, roepen de belangrijke vraag op in hoeverre dergelijke preventiemaatregelen gerechtvaardigd zijn. Op basis van deze probleemstelling heb ik aan het begin van mijn onderzoek in 2005 een onderzoeksvraag geformuleerd, die in eerste instantie bestond uit een zestal deelvragen (deze worden besproken in §2.1).

Onderzoeksvragen (1e versie, 2005) (§2.1)

1. Wat kan? (Welke preventiemaatregelen kunnen worden uitgevoerd?)
2. Wie kan? (Wie kunnen deze preventiemaatregelen uitvoeren?)
3. Wat mag? (Welke preventiemaatregelen mogen worden uitgevoerd?)
4. Wie mag? (Wie mogen deze preventiemaatregelen uitvoeren?)
5. Wat moet? (Welke preventiemaatregelen moeten worden uitgevoerd?)
6. Wie moet? (Wie moeten deze preventiemaatregelen uitvoeren?)

Gedurende de loop van mijn onderzoek zijn deze zes deelvragen geëvolueerd tot de 4e en laatste versie van de onderzoeksvraag die ik in 2008 heb geformuleerd. Hieronder geef ik een overzicht van de aanscherping van de onderzoeksvraag gedurende de loop van het onderzoek. De onderzoeksvragen worden besproken in de genoemde paragrafen.

Onderzoeksvraag (2e versie, 2005) (§2.1)

Kan een model worden ontwikkeld op basis waarvan de ***legitimiteit*** van preventiemaatregelen ter voorkoming van leefstijlgerelateerde aandoeningen kan worden beoordeeld?

Onderzoeksvraag (3e versie, 2006) (§2.4)

Kan een model worden ontwikkeld op basis waarvan de ***gerechtvaardigdheid*** van preventiemaatregelen ter voorkoming van leefstijlgerelateerde aandoeningen kan worden beoordeeld?

Onderzoeksvraag (4e versie, 2008) (§2.7.2)

> Kan een model worden ontwikkeld aan de hand waarvan de gerechtvaardigdheid van preventiemaatregelen kan worden beoordeeld, die ter bestrijding van leefstijlgerelateerde aandoeningen ***trachten de leefstijl van mensen te beïnvloeden***?

De betekenis die de begrippen in de onderzoeksvraag in het kader van mijn onderzoek hebben, licht ik toe aan de hand van de zinsdelen waaruit de 4e en tevens laatste versie van de onderzoeksvraag is opgebouwd.

Kan een model worden ontwikkeld

Het streven is om een model te ontwikkelen dat algemeen geldig en toepasbaar is. Dit betekent dat het model internationaal en voor alle leefstijlgerelateerde aandoeningen bruikbaar is. Een voorbeeld van een algemeen geldig en toepasbaar model wordt gevormd door de 'criteria van Wilson en Jungner'. Al in 1968 hebben Wilson en Jungner criteria geformuleerd voor het bepalen van de aanvaardbaarheid van een bevolkingsonderzoek. Hoewel dit 'model' al bijna 40 jaar oud is, zijn de geformuleerde criteria nog steeds relevant (Brug, 2004, p.111).

Aan de hand waarvan … kan worden beoordeeld

Het model zelf geeft geen oordeel over de gerechtvaardigdheid, maar de beoordelaar gebruikt het model als beoordelingskader om tot een rationele, rechtvaardige en transparante beoordeling te komen. Uiteenlopende (ideologische) opvattingen van beoordelaars kunnen leiden tot een andere uitkomst van de beoordeling van de gerechtvaardigdheid van een preventiemaatregel. Het is bijvoorbeeld denkbaar dat een socialist in vergelijking tot een liberaal een zwaarder gewicht toekent aan de bescherming van de gezondheid van burgers dan aan de keuzevrijheid van burgers. Een dergelijke verschillende weging van waarden kan binnen het model tot een andere uitkomst van de beoordeling van de gerechtvaardigdheid van een preventiemaatregel leiden[6].

Gerechtvaardigdheid

Het begrip 'gerechtvaardigdheid' verwijst naar het Engelse begrip 'justification'. Het Engelse begrip 'justification' heeft een bredere betekenis dan het Nederlandse

6. Vergelijk dit bijvoorbeeld met de werking van de Stemwijzer (www.stemwijzer.nl) en het Kieskompas (www.kieskompas.nl) die afhankelijk van de opvattingen van het individu tot een ander stemadvies komen.

begrip 'legitimiteit', dat vooral refereert aan wet- en regelgeving (legislatie = wetgeving). De 'justification' van een preventiemaatregel betreft de beoordeling op basis van de algemeen aanvaarde normen en waarden binnen een democratische samenleving. De begrippen 'redelijkheid, billijkheid en transparantie' spelen daarbij een centrale rol (zie hoofdstuk 4). Het begrip 'justification' heeft geen equivalent in het Nederlands. Het woord 'gerechtvaardigdheid' lijkt de best mogelijke vertaling.

Ter bestrijding

Gekozen is voor de term 'ter bestrijding' in plaats van 'ter voorkoming' om te benadrukken dat de preventiemaatregelen ook worden toegepast ter bestrijding van de leefstijlgerelateerde aandoeningen die mensen al hebben (dan is er dus geen sprake meer van het voorkomen van de aandoeningen).

Trachten de leefstijl van mensen te beïnvloeden

Er zijn ook andere mogelijkheden dan leefstijlbeïnvloeding om (de gevolgen van) leefstijlgerelateerde aandoeningen te bestrijden. Mensen met een te hoge bloeddruk en een te hoog cholesterolgehalte ten gevolge van ernstig overgewicht kunnen ter preventie van schade aan hart en bloedvaten bijvoorbeeld ook bloeddruk- en cholesterolverlagende medicijnen innemen. Het beoogde model richt zich op de preventiemaatregelen die tot doel hebben de leefstijl van mensen te beïnvloeden.

1.3 Focus op gerechtvaardigdheid van de argumentatie

De gerechtvaardigdheid van de toepassing van een preventiemaatregel berust m.i. op twee pijlers (zie §4.1 en 4.5):

1. De gerechtvaardigdheid van de ***argumentatie*** die heeft geleid tot het besluit om de preventiemaatregel toe te passen;
2. De gerechtvaardigdheid van de ***procedure*** (welke partijen kunnen op welk moment welke invloed uitoefenen op de besluitvorming?) die heeft geleid tot het besluit om de preventiemaatregel toe te passen.

Het beoogde beoordelingsmodel richt zich op de gerechtvaardigdheid van de argumentatie. Bij de beoordeling van de gerechtvaardigdheid van de preventiemaatregel aan de hand van het model kunnen procedurele aspecten worden meegewogen, voor zover deze van invloed zijn op de gerechtvaardigdheid van de argumentatie. Afhankelijk van de casus dient per criterium te worden afgewogen in hoeverre procedurele aspecten relevant zijn voor de inhoudelijke beoordeling van de gerechtvaardigdheid van de preventiemaatregel (zie §12.4.4).

In het maatschappelijke, politieke en wetenschappelijke debat over de (on)wenselijkheid om mensen via drang of dwang hun ongezonde of risicovolle leefstijl aan te laten passen zijn veel onvolkomenheden in de aangevoerde argumentaties te vinden. De aangevoerde argumentaties zijn vaak onvolledig en onevenwichtig en soms feitelijk onjuist. Daarbij kan bijvoorbeeld worden gedacht aan het debat over de totstandkoming en toepassing van het wettelijk rookverbod in de Nederlandse horeca, waarbij voor- en tegenstanders meestal maar een of twee argumenten aanvoeren. Als argument voor het wettelijk rookverbod: het gedwongen passief meeroken door het horecapersoneel. Als argument tegen het wettelijk rookverbod: het klantenverlies van cafés die te klein zijn om een afzonderlijke rookruimte te realiseren, wat zou kunnen leiden tot faillissement. Dit zijn belangrijke argumenten, maar zij vormen slechts een klein deel van alle argumenten die relevant zijn om de gerechtvaardigdheid van het wettelijk rookverbod in de horeca te beoordelen (zie de hoofdstukken 10 t/m 14). Het doel van het beoordelingsmodel is het bevorderen van de redelijkheid en billijkheid van de argumentatie die ten grondslag ligt aan de toepassing van preventiemaatregelen ter bestrijding van leefstijlgerelateerde aandoeningen. In hoofdstuk 4 worden de eisen aan de redelijkheid en billijkheid van de argumentatie nader door mij uitgewerkt.

1.4 Fasering van het onderzoek

Om de onderzoeksvraag te beantwoorden heb ik onderzoek gedaan. Dit onderzoek valt in twee delen uiteen:

- ontwikkeling van het beoordelingsmodel (hoofdstuk 2 t/m 4);
- toetsing van de juistheid, volledigheid en praktische toepasbaarheid van het ontwikkelde beoordelingsmodel (hoofdstuk 5 t/m 14).

1.4.1 Ontwikkeling van het beoordelingsmodel op basis van literatuuronderzoek

Voor de ontwikkeling van het beoordelingsmodel heb ik literatuuronderzoek verricht. Inhoudelijk valt dit literatuuronderzoek in drie delen uiteen:

1. Het eerste deel van dit literatuuronderzoek betreft een onderzoek naar bestaande beoordelingsmodellen, die kunnen worden gebruikt bij de ontwikkeling van het beoogde model (zie hoofdstuk 2).
2. Het tweede deel van dit literatuuronderzoek betreft een onderzoek naar relevante beoordelingsprincipes voor het beoogde model (zie hoofdstuk 3 en 4).
3. Het derde deel van dit literatuuronderzoek betreft praktijkvoorbeelden van de preventie van leefstijlgerelateerde aandoeningen (zie bijlage 6).

Het eerste deel van het literatuuronderzoek heeft de volgende modellen opgeleverd, die ik heb gebruikt bij de ontwikkeling van het door mij beoogde model:

- de criteria van Wilson en Jungner voor beslissingen over de aanvaardbaarheid van screening van de bevolking op aandoeningen (1968);
- de Trechter van Dunning voor pakketbeslissingen (1991);
- de criteria van de Gezondheidsraad voor de aanvaardbaarheid van genetische screening (1994);
- een model over de gerechtvaardigdheid van public health interventies (Gostin, 2000);
- de criteria van Childress e.a. voor de rechtvaardiging van preventiemaatregelen in de public health (2002);
- een model – de Preffi – voor het effectief managen van gezondheidsbevordering (Molleman, 2003, 2005, 2006);
- de criteria van het CVZ voor pakketbeslissingen (Mastenbroek, 2006);
- een model van de RVZ voor pakketbeslissingen (2006);
- de aanbevelingen van Mello voor het ontwerp van gelegitimeerde 'incentive programs' in de public health (2008).

In het kader van het tweede deel van het literatuuronderzoek heb ik mij met name gericht op de volgende onderwerpen (tussen haakjes enkele voor mijn onderzoek belangrijke publicaties over het betreffende onderwerp):

- schadebeginsel (Maris, 2006);
- accumulerende schade (Kernohan, 1993);
- paternalisme (Feinberg, 1984);
- neutraliteit, perfectionisme (Clarke, 2006);
- libertair paternalisme (Sunstein en Thaler, 2003) (Camerer, 2003);
- life politics (Scholtmeijer, 2006);
- ethische integriteit (Dworkin, 2000);
- fair besluitvormingsproces (Daniels, 2000);
- mensenrechten (VN, 1948) (Nussbaum, 2001);
- keuzevrijheid (Schwartz, 2004).

In het derde deel van het literatuuronderzoek heb ik mij geconcentreerd op de literatuur over de preventie van overgewicht vanwege de actualiteit van dit onderwerp en omdat hierin bijna alle voor de beoordeling van de gerechtvaardigdheid van preventiemaatregelen relevante aspecten naar voren komen[7]:

---

7. Het verslavingsaspect is bij het gebruik van teveel of ongezonde voedingsmiddelen minder evident dan bij roken of overmatig alcoholgebruik. Een ander aspect dat mist, is dat het innemen van teveel of ongezonde voeding niet schadelijk is voor de gezondheid van derden, terwijl passief roken dat wel is.

- voeding is op zichzelf niet ongezond, wordt dagelijks gebruikt, en het gebruik is noodzakelijk;
- alleen een teveel aan voeding leidt tot overgewicht, maar meer bewegen vormt tot op zekere hoogte een alternatief voor minder eten;
- overgewicht is zowel onder volwassenen als onder kinderen een groeiend probleem dat kan leiden tot ernstige aandoeningen (diabetes, gewrichtsproblemen, cardiovasculaire aandoeningen);
- voeding is op veel verschillende plaatsen te koop, wordt gedeeltelijk door mensen zelf bereid (van invloed op de verantwoordelijkheid en aansprakelijkheid voor overgewicht) en wordt op veel verschillende plaatsen geconsumeerd;
- bij de verkoop van voedingsmiddelen zijn grote nationale en internationale financiële belangen gemoeid en er wordt veel reclame voor gemaakt;
- voor de preventie van overgewicht zijn veel verschillende preventiemaatregelen denkbaar en er kunnen veel verschillende actoren bij worden betrokken.

Op basis van nieuwe literatuur en toetsing van het ontwikkelde beoordelingsmodel aan casuïstiek uit de praktijk, heb ik het model steeds verbeterd. Dit heeft geleid tot opeenvolgende versies van het model. In de titel van ieder hoofdstuk wordt vermeld op welke versie van het model het betreffende hoofdstuk betrekking heeft.

1.4.2 Empirische toetsing van het beoordelingsmodel op basis van casuïstiek

In de hoofdstukken 5 t/m 14 toets ik de juistheid, volledigheid en praktische toepasbaarheid van het door mij ontwikkelde beoordelingsmodel aan de hand van twee praktijkcasussen. In de eerste casus ‘Clarian Health’ (hoofdstuk 5 t/m 9) speelt de preventie van overgewicht een grote rol. De tweede casus (hoofdstuk 10 t/m 14) betreft het wettelijk rookverbod in de Nederlandse horeca, omdat dit in Nederland actueel is, er veel informatie over de totstandkoming van dit rookverbod beschikbaar is, en er veel wetenschappelijk bewijs is over de schadelijkheid van passief roken. Versie 6 van het door mij ontwikkelde model heb ik getoetst aan de casus ‘Clarian Health’. De toetsing van het model aan de casus ‘Clarian Health’ heeft geleid tot versie 7 van het model. De toetsing van het model aan de casus ‘wettelijk rookverbod in de horeca’ heeft geleid tot versie 8 en 9 van het model. De toetsing van het model aan de twee genoemde praktijkcasussen heeft geleid tot nieuwe inzichten en daarmee tot verdere verbetering van het model.

1.5 Onderzoeksmethoden

Voor het onderzoek heb ik gebruik gemaakt van drie onderzoeksmethoden:
- literatuuronderzoek;
- argumentatieve tekstanalyse;
- redeneren.

1.5.1 Literatuuronderzoek

Het literatuuronderzoek dat ik heb uitgevoerd ten behoeve van de ontwikkeling van het beoordelingsmodel is gebaseerd op:
- wetenschappelijke publicaties die ik heb gevonden via de online databases van PubMed[8] en PiCarta[9];
- publicaties van het College voor zorgverzekeringen (CVZ), de Gezondheidsraad, de Raad voor Volksgezondheid en Zorg (RVZ), het Rijksinstituut voor Volksgezondheid en Milieu (RIVM), en onderzoekscommissies van de Nederlandse en van de Britse overheid;
- enkele Nederlandse publicaties (o.a. een proefschrift en een universitair studieboek).

Ik ben het literatuuronderzoek gestart met een uitgebreide zoektocht in PubMed en PiCarta op basis van combinaties van zoektermen die relevant zijn voor het te ontwikkelen beoordelingsmodel. Voor het literatuuronderzoek bleek PiCarta een zeer belangrijke aanvulling op PubMed, omdat veel onderwerpen waarop ik het literatuuronderzoek heb gericht (zie §1.4.1) niet binnen het medische domein vallen. Nagenoeg alle door mij gebruikte publicaties bevatten een literatuurlijst met verwijzingen naar potentieel relevante literatuur. Ook van deze mogelijkheid om relevante wetenschappelijke literatuur te vinden, heb ik gebruik gemaakt.

Belangrijke zoektermen in PubMed en PiCarta waren:
- public health, life style, health behavior, prevent(ion);

8. "PubMed comprises over 21 million citations for biomedical literature from MEDLINE, life science journals, and online books. … PubMed is a free resource that is developed and maintained by the National Center for Biotechnology Information (NCBI), at the U.S. National Library of Medicine (NLM), located at the National Institutes of Health (NIH)." (NCBI. Last update: September 23, 2011.) http://www.ncbi.nlm.nih.gov/books/NBK3827/#pubmedhelp.How_to_Get_the_Journ
9. "PiCarta geeft toegang tot de Nederlandse Centrale Catalogus NCC en de Online Contents. De NCC bevat titelbeschrijvingen van alle boeken en tijdschriften in het bezit van meer dan 400 bibliotheken in Nederland." (Universiteit Leiden. Laatst gewijzigd: 18 november 2010.) www.bibliotheek.leidenuniv.nl/catalogi-databases/veelgebruikte-databases/picarta.html

– persuasion, coercion, paternalism, responsibility, incentive(s), tax(es);
– human rights, civil rights, ethics, moral;
– autonomy, freedom, liberty, choice behavior, privacy, justice;
– overweight, obesity, tobacco, smoking, alcohol;
– framework, justification;
– review.

Een overzicht van de gebruikte literatuur is te vinden in de literatuurlijst.

1.5.2 Argumentatieve tekstanalyse

De toetsing van de juistheid, volledigheid en praktische toepasbaarheid van het model is gebaseerd op een argumentatieve analyse van teksten die op internet beschikbaar waren over het preventieplan van Clarian Health en over het wettelijk rookverbod in de Nederlandse horeca. In het geval van de Clarian-Healthcasus gaat het om enkele nieuwsberichten over het preventieplan van Clarian Health en een groot aantal schriftelijke reacties van burgers op deze nieuwsberichten (zie hoofdstuk 5). In het geval van de rookverbod-horecacasus gaat het om een groot aantal parlementaire verslagen en om de wet- en regelgeving m.b.t. (de totstandkoming van) het wettelijk rookverbod in de horeca (zie §10.2). In beide gevallen gaat het grotendeels om niet-wetenschappelijke teksten. Een belangrijk voordeel van toetsing van het model aan deze teksten is dat deze teksten niet met het oog op de toetsing van het model op schrift zijn gesteld. Een eerste voordeel hiervan is dat de inhoud van de teksten voorafgaand aan de argumentatieve analyse van de teksten niet is beïnvloed door de criteria en structuur van het model. Dit is van belang met het oog op de objectiviteit van de toetsing van het model. Als ik interviews of enquêtes had gebruikt voor de toetsing van het model, zou de inhoud van de dataverzameling onvermijdelijk door de criteria en structuur van het model zijn beïnvloed. Een tweede voordeel is dat deze vorm van toetsing meer zekerheid geeft over de volledigheid ervan. Met name de toetsing van het model aan de casus ‘wettelijk rookverbod in de horeca’ geeft de nodige zekerheid over de volledigheid van het model. De parlementaire verslagen en wetsteksten over deze casus beslaan namelijk een periode van meer dan 25 jaar en bevatten argumenten van alle politieke partijen die in die periode deel hebben uitgemaakt van het parlement. Een derde voordeel is dat deze vorm van toetsing meer zekerheid geeft over de praktische toepasbaarheid van het model. De teksten weerspiegelen immers het maatschappelijke debat over de casus Clarian Health en het politieke debat over de totstandkoming en toepassing van het wettelijk rookverbod in de horeca, zoals die daadwerkelijk hebben plaatsgevonden. Met het oog op de controleerbaarheid van de argumentatieve tekstanalyses aan de hand waarvan ik juistheid, volledigheid en

praktische toepasbaarheid van het model heb getoetst, werk ik in de betreffende hoofdstukken veel met citaten.

1.5.3 Redeneren

Redeneren kan als volgt worden gedefinieerd (Goel en Waechter, 2010):

> *"Reasoning is the process of evaluating given information and reaching conclusions not explicitly stated in the premises."* (p.2)

Volgens Goel en Waechter (2010) is redeneren een belangrijke mogelijkheid om kennis te vergaren en de juistheid ervan te toetsen:

> *"One major area of study for philosophy is the acquisition and justification of knowledge. Reasoning from given information (premises) to conclusions is one important source of knowledge."* (p.4)

De ontwikkeling van het model was een proces van het voortdurend bedenken en uit proberen van verbeteringen (zie §2.1), totdat het punt werd bereikt waarop ik geen substantiële mogelijkheden tot verbetering meer zag (zie §15.1.6). De verbeteringen die ik tussen de eerste versie van het model (§2.2) en de laatste versie van het model (§14.2) heb aangebracht, betreffen de criteria, de structuur, en de wijze van beoordeling van het model. Deze verbeteringen zijn tot stand gekomen op basis van voortschrijdend inzicht ten gevolge van nieuwe literatuur, toetsing van het model aan casuïstiek uit de praktijk, commentaar van mijn begeleiders, en een groeiend inzicht door jaren achtereen met de ontwikkeling en toetsing van het model bezig te zijn. Ik heb getracht de verbeteringen die ik heb aangebracht steeds goed te beargumenteren. Aan de keuzes die ik bij de ontwikkeling en toetsing van het model heb gemaakt, liggen vaak vele argumenten ten grondslag. Met het oog op de uitvoerbaarheid en leesbaarheid heb ik er veelal voor gekozen om alleen de belangrijkste argumenten weer te geven als deze voldoende onderbouwing voor mijn keuze vormden.

1.6 Discussie

In het eerste deel van de discussie wordt de onderzoeksvraag beantwoord door presentatie van het model (§15.1). In het tweede deel van de discussie (§15.12) worden de volgende aspecten van het definitieve beoordelingsmodel kritisch beschouwd:

- neutraliteit en volledigheid van het model;
- validiteit en praktische toepasbaarheid van het model;

- betrouwbaarheid van het model;
- generaliseerbaarheid van het model;
- ‘appraisal and assessment’;
- invloed van de implementatie op de effecten en neveneffecten van de preventiemaatregel.

In het derde deel van de discussie (§15.3) wordt de relevantie van het ontwikkelde model voor het beleid, de praktijk en de wetenschap besproken. In dit verband worden ook aanbevelingen gegeven.

2 Criteria van het beoordelingsmodel *(versie 1 t/m 6 van het model)*

In dit hoofdstuk bespreek ik in chronologische volgorde de ontwikkeling van de criteria van zes versies van het beoordelingsmodel. De structuur van het model (volgorde van de criteria) wordt in het volgende hoofdstuk besproken.

2.1 Ontwikkeling van de criteria van het beoordelingsmodel

In dit hoofdstuk bespreek ik de ontwikkeling van de criteria van het model tot aan de toetsing van de juistheid, volledigheid en praktische toepasbaarheid van het model aan de casus Clarian Health (1e praktijkcasus). De ontwikkeling van de criteria was een proces van steeds opnieuw blijven proberen, totdat ik de in mijn ogen juiste criteria had gevonden. Nu ik dit opschrijf, moet ik aan James Dyson denken, die ruim 5000 prototypes ontwikkelde voordat hij de in zijn ogen optimale stofzuiger uitvond[1]. Zo ver ben ik niet gegaan, maar als ik alle kleine wijzigingen in criteria meetel, zijn er veel meer varianten van het model geweest dan de zes versies die ik in dit hoofdstuk beschrijf. Dit ontwikkelingsproces heeft zich afgespeeld in een tijdsbestek van ruim 3 jaar (2005 t/m begin 2008). De argumentatieve onderbouwing van de verschillende versies van het model, betrof steeds de onderbouwing achteraf, na de totstandkoming van de betreffende versie. De reden daarvoor is dat het denkproces dat heeft geleid tot de betreffende versie vaak niet lineair maar sprongsgewijs is verlopen. Door te trachten hier een lineaire beschrijving van te maken, waarbij de 1e versie via kleine logische stappen is geëvolueerd tot de laatste versie, zou ik de werkelijkheid geweld aan doen. Daarom heb ik ervoor gekozen om uit alle beschikbare varianten van het model een zestal, voor de ontwikkeling van de criteria van het model bepalende, versies te kiezen en deze te onderbouwen met de argumentatie die ik had direct na de totstandkoming van de betreffende versie. Deze werkwijze heeft ertoe geleid dat ik in dit hoofdstuk een aantal keer al in het begin van de paragraaf de nieuwe versie van het model presenteer en pas daarna de argumentatieve onderbouwing. Door zo te werken hoefde ik niet een lineair denkproces te construeren dat op die manier niet heeft plaatsge-

---

1. http://www.dyson.com/about/story/default.asp?searchType=story

vonden en bleef ik zo dicht mogelijk bij de werkelijkheid. De chronologische bespreking van de door mij gekozen zes versies van het model geeft een goed beeld van de ontwikkeling van de criteria van het model, vanaf het moment van het vaststellen van de eerste onderzoeksvragen (2005) tot en met de totstandkoming van de versie die ik heb getoetst aan de casus Clarian Health (2008). Deze weergave geeft ook een goed beeld van het proces van modelontwikkeling dat heeft plaatsgevonden op een hoger abstractieniveau dan van alle afzonderlijke varianten.

2.2 Eerste versie van het beoordelingsmodel

De aanleiding voor mijn belangstelling om een model te ontwikkelen voor de beoordeling van de legitimiteit[2] van preventiemaatregelen ter bestrijding van leefstijlgerelateerde aandoeningen was een gesprek met mijn promotor over het toenemende gevaar van zulke aandoeningen voor de volksgezondheid (zie de inleiding). Wij bespraken dat in de afgelopen decennia duidelijk is geworden dat uitsluitend gezondheidsvoorlichting er in veel gevallen niet toe leidt dat mensen hun leefstijl aanpassen en gezonder gaan leven (Brug, 2004, p.98). Een mogelijkheid om preventiemaatregelen ter bestrijding van leefstijlgerelateerde aandoeningen minder vrijblijvend te maken dan gezondheidsvoorlichting, zou kunnen bestaan uit het aanbrengen van veranderingen in de omgeving. Mensen meer laten bewegen zou bijvoorbeeld kunnen worden gestimuleerd door in kantoorgebouwen het gebruik van de trap te bevorderen en het gebruik van de lift te ontmoedigen, of door de woon- en werkomgeving fietsvriendelijk en auto-onvriendelijk te maken. Een andere mogelijkheid om de bevordering van een gezonde leefstijl minder vrijblijvend te maken, zou kunnen bestaan uit het introduceren of versterken van financiële prikkels die gezond gedrag bevorderen (bv. korting op het lidmaatschap van een sportvereniging, of lagere prijzen voor gezonde voedingsmiddelen) of die ongezond gedrag terugdringen (bv. de invoering van een zogenaamde vet-tax of verhoging van de accijns op laag-alcoholische drank). Ten slotte is er nog de mogelijkheid om (on)gezond gedrag wettelijk te reguleren. Hierbij kan bijvoorbeeld worden gedacht aan het wettelijk verplicht stellen van een minimum aantal uren gymnastiek op scholen, een verbod op het bezit van harddrugs, of een verbod voor de horeca op het gebruik van bak- en frituurvetten met (een hoog gehalte aan) kunstmatige transvetten. In dit laatste geval wordt het ongezonde gedrag – consumptie van transvetten – niet rechtstreeks verboden, maar wordt getracht dit

2. Ten tijde van de ontwikkeling van de eerste versie van het model had ik in de onderzoeksvraag het woord ‘legitimiteit’ nog niet vervangen door het woord ‘gerechtvaardigdheid’ (zie §1.2).

gedrag terug te dringen door de bereiding van voedingsmiddelen te beïnvloeden. Het aantal mogelijkheden om met omgevingsveranderingen, financiële prikkels en wet- en regelgeving de leefstijl van mensen te beïnvloeden is legio en ik besprak met mijn promotor dat deze mogelijkheden in het kader van het nationale, Europese en mondiale volksgezondheidsbeleid waarschijnlijk worden onderbenut. Zouden overheden niet veel meer van dergelijke minder vrijblijvende maatregelen gebruik moeten maken? Aan de andere kant waren we het eens dat betutteling en onvrijheid op de loer liggen, als dergelijke minder vrijblijvende maatregelen grootschalig worden ingezet. Bovendien zijn de effecten en neveneffecten van veel maatregelen onbekend. Ik vond dit een interessant thema om nader uit te werken ten behoeve van een promotieonderzoek. Allereerst heb ik nagedacht hoe ik de voorgaande gedachtewisseling zou kunnen vertalen in onderzoeksvragen. Dit leverde zes korte (deel)onderzoeksvragen op.

Onderzoeksvragen (1e versie, 2005)

1. ***Wat kan?*** (Welke preventiemaatregelen kunnen worden uitgevoerd?)
2. ***Wie kan?*** (Wie kunnen deze preventiemaatregelen uitvoeren?)
3. ***Wat mag?*** (Welke preventiemaatregelen mogen worden uitgevoerd?)
4. ***Wie mag?*** (Wie mogen deze preventiemaatregelen uitvoeren?)
5. ***Wat moet?*** (Welke preventiemaatregelen moeten worden uitgevoerd?)
6. ***Wie moet?*** (Wie moeten deze preventiemaatregelen uitvoeren?)

Met name de vraag *'Wat moet?'* intrigeerde mij aanvankelijk. Zou ter preventie van leefstijlgerelateerde aandoeningen niet veel meer gebruik moeten worden gemaakt van (lichte) vormen van drang of dwang om mensen aan te zetten tot een gezondere leefstijl? Als er nieuwe kosteneffectieve behandelingen voor leefstijlgerelateerde aandoeningen (zoals diabetes, hart- en vaatziekten, e.d.) beschikbaar komen, worden deze in het algemeen gefinancierd en ingevoerd. Dit lijkt niet te gelden voor kosteneffectieve preventiemaatregelen ter bestrijding van leefstijlgerelateerde aandoeningen, terwijl voorkomen toch beter is dan genezen. Een veelgehoorde verklaring hiervoor is dat zulke preventiemaatregelen ten koste gaan van de keuzevrijheid van de bevolking. Maar is dat wel zo? Gaat het bijvoorbeeld wel ten koste van de keuzevrijheid van de bevolking, als er wettelijk een grens wordt gesteld aan de toegestane hoeveelheid zout in voedingsmiddelen (de consument kan immers altijd zelf zout toevoegen) of als portiegroottes voor bepaalde voedingsmiddelen wettelijk worden gemaximeerd (de consument kan immers altijd meer porties kopen en eten)? Vormt de drang tot kopen en consumeren die uitgaat van de marketing van levens- en genotsmiddelen niet een groter gevaar voor de keuze***vrijheid*** van de bevolking dan de bovengenoemde preventiemaatregelen?

Ten slotte kan men zich afvragen of een vermindering van de keuzevrijheid wel nadelig is voor de bevolking. In het artikel ‘The tyranny of choice’ (Schwartz, 2004), dat gebaseerd is op het boek ‘The paradox of choice: why more is less’ (Schwartz, 2003) stelt Schwartz dat Amerikaanse consumenten lijden onder keuzestress en dat een vermindering van het aantal keuzemogelijkheden hun welbevinden in veel gevallen positief zal beïnvloeden. Moeten bepaalde preventiemaatregelen ter voorkoming van leefstijlgerelateerde aandoeningen niet gewoon worden ingevoerd als ze kosteneffectief zijn *(‘Wat moet?’)*? Hieraan vooraf gaan natuurlijk de vragen naar welke (effectieve) preventiemaatregelen mogelijk zijn *(‘Wat kan?’)* en welke preventiemaatregelen zijn toegestaan *(‘Wat mag?’).* Als is vastgesteld dat een bepaalde preventiemaatregel ‘moet’ worden uitgevoerd, is de volgende vraag wie daarvoor dan verantwoordelijk is *(‘Wie moet?’)*: de landelijke overheid, of de lokale overheid, of het bedrijfsleven, enz.? Hieraan vooraf gaan de vragen welke partijen een bepaalde preventiemaatregel kunnen uitvoeren *(‘Wie kan?’)* en mogen uitvoeren *(‘Wie mag?’)*. Wetgeving kan bijvoorbeeld alleen worden gemaakt door de overheid.

Onderstaand zijn de zes genoemde onderzoeksvragen in een schema geplaatst, dat van links naar rechts dient te worden gelezen. Dit ***schema*** kan achteraf worden opgevat als de ***eerste versie*** van mijn beoordelingsmodel.

Versie 1 van het beoordelingsmodel (2005)

Wat kan?	Wie kan?	Wat mag?	Wie mag?	Wat moet?	Wie moet?

De vraag *‘Wat kan?’* (welke preventiemaatregelen zijn denkbaar) leidt tot de grootste verzameling van preventiemaatregelen ter bestrijding van leefstijlgerelateerde aandoeningen. Het aantal antwoorden op deze vraag is ‘oneindig’ groot, en trachten een opsomming te geven van alle denkbare preventiemaatregelen is daarom een onmogelijke en zinloze activiteit. Wat wel mogelijk en niet op voorhand zinloos is, is een ***categorisering*** van alle denkbare preventiemaatregelen. Wellicht bestaat er een verband tussen enerzijds de aard van een preventiemaatregel (de inhoudelijke categorie waartoe de preventiemaatregel behoort) en anderzijds de overige vijf onderzoeksvragen. De eerste vraag *‘Wat kan?’* heb ik daarom vertaald in de vraag wat met het oog op de vervolgvragen een zinvolle indeling in categorieën is van alle denkbare preventiemaatregelen.

Preventiemaatregelen kunnen op verschillende manieren in categorieën worden ingedeeld:

1. Doelstelling van de preventie:
   a. gezondheidsbescherming;
   b. ziektepreventie;
   c. gezondheidsbevordering.
2. Stadium van de aandoening:
   a. primaire preventie;
   b. secundaire preventie;
   c. tertiaire preventie.
3. Doelgroep van de preventie:
   a. collectieve preventie;
   b. opportunistische preventie;
   of:
   c. collectieve preventie;
   d. individuele preventie;
   of:
   e. populatiebenadering;
   f. hoogrisicobenadering.

In ***bijlage 1*** worden deze begrippen (categorieën van preventiemaatregelen) toegelicht. In ieder van de twaalf genoemde categorieën van preventiemaatregelen kan een oneindig aantal (varianten van) denkbare preventiemaatregelen worden geplaatst. Binnen iedere categorie kunnen preventiemaatregelen worden geplaatst die heel verschillend van aard zijn. Op basis hiervan ben ik tot de conclusie gekomen dat de genoemde categorieën afzonderlijk geen bruikbare aanknopingspunten bieden voor het beantwoorden van de vijf volgende onderzoeksvragen.

Vervolgens heb ik geprobeerd of het combineren van verschillende categorieën bruikbare aanknopingspunten zou kunnen bieden voor het beantwoorden van de onderzoeksvragen. De volgende drie combinaties heb ik onderzocht (de combinaties verschillen onderling alleen m.b.t. de onderstreepte categorieën):

– 1e combinatie: (1a, 1b, 1c), (2a, 2b, 2c), (3a, 3b);
– 2e combinatie: (1a, 1b, 1c), (2a, 2b, 2c), (3c, 3d);
– 3e combinatie: (1a, 1b, 1c), (2a, 2b, 2c), (3e, 3f).

Ter illustratie toont overzicht 1 de 3e combinatie (1a, 1b, 1c), (2a, 2b, 2c), (3e, 3f).

Overzicht 1: categorieën van preventiemaatregelen gecombineerd

	Primaire preventie (2a)		Secundaire preventie (2b)		Tertiaire preventie (2c)	
	Populatie-gericht (3e)	Hoogrisico-gericht (3f)	Populatie-gericht (3e)	Hoogrisico-gericht (3f)	Populatie-gericht (3e)	Hoogrisico-gericht (3f)
Gezondheids-bescherming (1a)	1	2	3	4	5	6
Ziekte-preventie (1b)	7	8	9	10	11	12
Gezondheids-bevordering (1c)	13	14	15	16	17	18

In ***bijlage 2*** heb ik overzicht 1 uitgewerkt door te proberen voor iedere cel (1 t/m 18) preventiemaatregelen te bedenken die in deze cel zouden kunnen worden geplaatst.

De drie onderzochte combinaties leiden ieder tot een onderverdeling in 18 subcategorieën van preventiemaatregelen. Voor alle drie de onderzochte combinaties geldt, dat het te vaak voorkomt dat in eenzelfde subcategorie preventiemaatregelen kunnen worden geplaatst die heel verschillend van aard zijn. Op basis hiervan heb ik geconcludeerd dat een onderverdeling van alle denkbare preventiemaatregelen in (sub)categorieën van preventiemaatregelen geen bruikbare aanknopingspunten biedt om de onderzoeksvragen *'wie kan, wat mag, wie mag, wat moet, wie moet'* te beantwoorden[3]. Daarom heb ik voor een andere benadering gekozen.

In plaats van alle denkbare preventiemaatregelen in (sub)categorieën te verdelen om de genoemde onderzoeksvragen per (sub)categorie van preventiemaatregelen te kunnen beantwoorden, ben ik er toe overgegaan om criteria te formuleren aan de hand waarvan de onderstaande onderzoeksvragen per afzonderlijke preventiemaatregel kunnen worden beantwoord:

– op welke manieren kan de preventiemaatregel worden uitgevoerd *(wat kan)*?
– wie kan de preventiemaatregel uitvoeren *(wie kan)*?
– mag de preventiemaatregel worden uitgevoerd *(wat mag)*?
– wie mag de preventiemaatregel uitvoeren *(wie mag)*?
– moet de preventiemaatregel worden uitgevoerd *(wat moet)*?
– wie moet de preventiemaatregel uitvoeren *(wie moet)*?

3. In 2009 is in Nederland in het kader van de discussie over mogelijke vergoeding van bepaalde preventiemaatregelen via de basiszorgverzekeringen een nieuwe indeling naar 'doelgroep van de preventie' gangbaar (Kroes, 2007) ***(zie bijlage 3)***. Deze nieuwe indeling leidt echter niet tot andere conclusies m.b.t. de onbruikbaarheid van de categorisering van preventiemaatregelen voor beantwoording van de genoemde onderzoeksvragen.

Omdat ook ***per preventiemaatregel*** oneindig veel variaties in de uitvoering mogelijk zijn – bijvoorbeeld door te variëren in doelgroep, intensiteit, tijdsduur, etc. – zijn op de onderzoeksvraag *'Wat kan?'* oneindig veel antwoorden mogelijk. Daarom heb ik besloten de onderzoeksvraag *'Wat kan?'* te laten vervallen. De onderzoeksvraag *'Wie kan?'* is afhankelijk en interfereert met de onderzoeksvraag *'Wat kan?'*. Ik heb daarom besloten de onderzoeksvraag *'Wie kan?'* eveneens te laten vervallen.

Gezien het grote aantal mogelijke (variaties binnen) preventiemaatregelen, lijkt het beantwoorden van de onderzoeksvragen 'Wat mag? Wie mag? Wat moet? Wie moet?' voor alle mogelijke preventiemaatregelen niet eenvoudig. Met het oog op de uitvoerbaarheid van het onderzoek stelde een van mijn begeleiders voor dat ik mij in ieder geval voorlopig zou beperken tot de 'legitimiteitsvragen' (*'Wat mag?'* en *'Wie mag?'*). Een bijkomende reden is dat de 'moet-vragen' (*'Wat moet?'* en *'Wie moet?'*) moeilijker objectief te beantwoorden lijken dan de 'mag-vragen'. Bij de 'moet-vragen' raakt men al snel in ideologische discussies verzeild over de (on) wenselijkheid van:

- ***perfectionisme*** (in hoeverre vertegenwoordigt de preventiemaatregel een overdreven streven naar een volmaakt gezonde leefstijl?),
- ***moralisme*** (in hoeverre vertegenwoordigt een ongezonde leefstijl 'kwaad' dat moet worden bestreden?), of
- ***paternalisme*** (in hoeverre moeten mensen met een ongezonde leefstijl tegen zichzelf worden beschermd?).

Dergelijke ideologische discussies zijn moeilijk objectiveerbaar en lijken haaks te staan op mijn streven om een rationeel beoordelingsmodel te ontwikkelen. In overleg met mijn begeleiders heb ik daarom besloten, de 'moet-vragen', in ieder geval voorlopig, te laten vallen en mij te concentreren op de 'mag-vragen'[4].

Bovenstaande overwegingen leidden tot een inperking van de oorspronkelijke zes onderzoeksvragen *(Wat kan? Wie kan? Wat mag? Wie mag? Wat moet? Wie moet?)* voor mijn proefschrift tot de volgende onderzoeksvraag.

---

4. In versie 5 van mijn model (§2.6.2) kom ik gedeeltelijk op dit besluit terug en wordt de vraag of de preventiemaatregel wel 'moet' worden uitgevoerd weer onderdeel van het model. Bij de bespreking van versie 5 wordt dit toegelicht.

Onderzoeksvraag (2e versie, 2005)

Kan een model worden ontwikkeld op basis waarvan de ***legitimiteit*** van preventiemaatregelen ter voorkoming van leefstijlgerelateerde aandoeningen kan worden beoordeeld?

De legitimiteit van de preventiemaatregel betreft de vragen '*Wat mag?*' en '*Wie mag?*'.

2.3 Tweede versie van het beoordelingsmodel

Versie 2 van het beoordelingsmodel is tot stand gekomen door de hierboven genoemde onderzoeksvraag uit te werken.

Bij de uitwerking van de 2e onderzoeksvraag heb ik vastgehouden aan het onderscheid tussen de vragen:
- Welke preventiemaatregelen mogen worden uitgevoerd? (*Wat mag?*).
- Wie mogen deze preventiemaatregelen uitvoeren? (*Wie mag?*).

Het is bijvoorbeeld denkbaar dat een bepaalde preventiemaatregel legitiem is als de overheid daartoe beslist, maar niet als de werkgever deze instelt. Denk hierbij bijvoorbeeld aan een verbod op het gebruik van softdrugs buiten werktijd, waartoe de overheid mogelijk wel en de werkgever mogelijk niet zou mogen beslissen.

Versie 2 van het beoordelingsmodel (2005)

Wat mag?
- Proportionaliteit
- Doelmatigheid
- Rechtvaardigheid

Wie mag?
- Bekwaamheid
- Bevoegdheid
- Geschiktheid

Ten opzichte van versie 1 van het model heb ik de volgende wijzigingen aangebracht:

- De vragen *'Wat kan? Wie kan? Wat moet? Wie moet?'* zijn vervallen. De redenen hiervoor zijn aangegeven bij de bespreking van versie 1 van het model.
- De vraag *'Wat mag?'* heb ik geoperationaliseerd middels de criteria: proportionaliteit (RVZ, 2002, p.7, 33), doelmatigheid en rechtvaardigheid.
- Het criterium 'proportionaliteit' betreft de vraag of de beoogde effecten van de preventiemaatregel (gezondheidswinst) opwegen tegen de neveneffecten (bijvoorbeeld verlies aan keuzevrijheid en economisch nadeel).
- Het criterium 'doelmatigheid' betreft de vraag of de beoogde effecten (gezondheidswinst) wel op een efficiënte wijze worden behaald.
- Het criterium 'rechtvaardigheid' is toegevoegd, omdat, ook als een preventiemaatregel proportioneel en doelmatig is, de effecten en neveneffecten onrechtvaardig over mensen verdeeld kunnen worden. Bovendien kan men van opvatting zijn dat het rechtvaardig is, als de sterkste schouders de zwaarste lasten dragen. De gedachte is dat onrechtvaardige maatregelen niet legitiem zijn. Het begrip 'legitiem' dient dus niet te letterlijk (legitiem = wettelijk) te worden genomen.
- De vraag *'Wie mag?'* heb ik geoperationaliseerd middels de criteria: bekwaamheid, bevoegdheid, geschiktheid.
- De criteria 'bekwaamheid en bevoegdheid' zijn geleend uit de Wet Big (Wet op de beroepen in de Individuele Gezondheidszorg). Het criterium 'bekwaamheid' betreft de vraag of men beschikt over de benodigde kennis en vaardigheden om de maatregel adequaat uit te kunnen voeren. Uitgangspunt van de Wet Big is, dat als men bekwaam is, men ook bevoegd is. Op dit uitgangspunt heeft de wetgever een uitzondering gemaakt door een aantal voorbehouden handelingen vast te stellen (Boomen, 1996, p14):

  > *"Het uitgangspunt van de Wet BIG is dat het handelen op het gebied van de individuele gezondheidszorg, al dan niet beroepsmatig verricht, in principe vrij is. Deze vrijheid geldt niet voor alle handelingen. Bepaalde handelingen die onaanvaardbare risico's voor de gezondheid van de patiënt meebrengen als ze door ondeskundigen worden uitgevoerd, zijn voorbehouden. De wet heeft een onderscheid gemaakt tussen beroepsbeoefenaren die zelfstandig bevoegd zijn en die niet zelfstandig bevoegd zijn om voorbehouden handelingen uit te voeren. Wie niet zelfstandig bevoegd is, mag in opdracht van een zelfstandig bevoegde en onder bepaalde voorwaarden een voorbehouden handeling uitvoeren. Alle anderen is het verboden om deze handelingen uit te voeren, tenzij er sprake is van een noodsituatie."*

  Misschien is het noodzakelijk om ook 'voorbehouden preventiemaatregelen' vast te stellen, waarbij het treffen van deze maatregelen aan bepaalde partijen is voorbehouden. Dit zou met name het geval kunnen zijn, als deze preventiemaatregelen een sterk vrijheidsbeperkend karakter hebben.

– Het criterium ‘geschiktheid’ is toegevoegd, omdat een actor wel over de bekwaam- en bevoegdheden kan beschikken om een bepaalde preventiemaatregel uit te voeren, maar ongeschikt kan zijn om deze preventiemaatregel uit te voeren. Denk bijvoorbeeld aan de tabaksindustrie, die wel beschikt over de bekwaam- en bevoegdheden om het roken onder jongeren terug te dringen, maar toch geen geschikte actor lijkt om de verantwoordelijkheid te dragen voor het terugdringen van roken onder jongeren.

Met het oog op de rationaliteit en algemene geldigheid (o.a. toepasbaarheid in verschillende culturen) van het beoordelingsmodel, streef ik naar een objectieve en neutrale (niet ideologische) (Clarke, 2006) beantwoording van de vragen *‘Wat mag?’* en *‘Wie mag?’*.

De vraag *‘Wat mag?’* wordt in versie 2 van het model beoordeeld aan de hand van de criteria ‘proportionaliteit, doelmatigheid en rechtvaardigheid’. Deze criteria betreffen de aanvaardbaarheid van de gevolgen van de preventiemaatregel, niet de aanvaardbaarheid van de doelstelling van de preventiemaatregel. Dit laatste leidt namelijk tot ideologische discussies over de perfectionistische, moralistische en paternalistische aspecten van de preventiemaatregel. Door in de beoordeling te focussen op de gevolgen van de preventiemaatregel en niet op de doelstelling, tracht ik de beoordeling te ont-ideologiseren. Bovendien kunnen de gevolgen van een preventiemaatregel empirisch worden onderzocht, waardoor de objectiviteit van de beantwoording wordt bevorderd.

De vraag *‘Wie mag?’* wordt beoordeeld aan de hand van de criteria ‘bekwaamheid, bevoegdheid en geschiktheid’. Door in de beoordeling gebruik te maken van de redenering die wordt gehanteerd in de Wet Big, dat wie bekwaam is daardoor bevoegd is, probeer ik weg te blijven bij vooringenomen opvattingen over bevoegdheid, die bijvoorbeeld cultureel of politiek-ideologisch bepaald zijn. Vooringenomen opvattingen bedreigen de objectiviteit en neutraliteit van de beoordeling aan de hand van het model. Om dezelfde reden probeer ik weg te blijven bij vooringenomen opvattingen over geschiktheid. De vraag of een partij geschikt is om een preventiemaatregel uit te voeren, dient te worden beoordeeld op basis van de effecten en neveneffecten van de uitvoering van de preventiemaatregel door een bepaalde partij en niet op basis van vooringenomen opvattingen over (on) geschiktheid. Zowel de bekwaamheid als geschiktheid van een partij kunnen

zodoende empirisch worden onderzocht, waardoor de objectiviteit van de beantwoording wordt bevorderd[5].

2.4 Derde versie van het beoordelingsmodel

Bij nader inzien vond ik versie 2 van het model te beperkt om daarmee de legitimiteit van preventiemaatregelen ter voorkoming van leefstijlgerelateerde aandoeningen te beoordelen. Doordenkend ontwikkelde ik versie 3. Eerst presenteer ik deze versie en daarna leg ik uit wat ik heb gewijzigd ten opzichte van versie 2, en waarom.

Versie 3 van het beoordelingsmodel (2005)

Legitimiteitsvraag:
1. Proportionaliteit
2. Doelmatigheid
3. Rechtvaardigheid
4. Frequentie en duur
5. Alternatieven

Verantwoordelijkheidsvraag:
1. Toepassingsbereik
2. Bekwaamheid en bevoegdheid
3. Geschiktheid
4. Procedurele zorgvuldigheid
5. Subsidiariteit

Wijzigingen t.o.v. versie 2 van het model:
– De *'wat-mag-vraag'* heeft het label 'legitimiteit' gekregen.
– De *'wie-mag-vraag'* heeft het label 'verantwoordelijkheid' gekregen (Wie mogen de preventiemaatregel wel en niet uitvoeren? Aan wie wordt de verantwoordelijkheid voor het uitvoeren van de preventiemaatregel wel en niet toevertrouwd?).

5. De empirie is per definitie politiek-ideologisch niet neutraal. In een Westerse samenleving waarin vrije marktwerking de norm is, zijn andere preventiemaatregelen (on)mogelijk dan in een communistische of een Islamitische samenleving.

- De verantwoordelijkheidsvraag betreft de vraag wie de verantwoordelijkheid mag dragen en niet de vraag wie de verantwoordelijkheid moet dragen. De onderzoeksvraag *'Wie moet?'* is immers vervallen.
- Aan de legitimiteitsvraag is het criterium 'frequentie en duur' toegevoegd. Voor de beoordeling van de legitimiteit van vrijheidsbeperkingen is dit een belangrijk criterium.
- Het criterium 'frequentie en duur' is niet toegevoegd aan de verantwoordelijkheidsvraag, omdat bekwaamheid en bevoegdheid in het algemeen niet tijdgebonden zijn.
- Aan de legitimiteitsvraag is het criterium 'alternatieven' toegevoegd, omdat de vraag of er minder vrijheidsbeperkende, of doelmatiger alternatieven zijn voor de preventiemaatregel, van grote invloed is op de legitimiteit.
- Aan de verantwoordelijkheidsvraag is het criterium 'toepassingsbereik' toegevoegd. Het toepassingsbereik betreft de aard en omvang van de doelgroep waarop de preventiemaatregel van toepassing is. Het toepassingsbereik is medebepalend voor de beoordeling van de bekwaamheid, bevoegdheid, en geschiktheid van de actor die de preventiemaatregel uitvoert.
- Bij de verantwoordelijkheidsvraag is het criterium 'procedurele zorgvuldigheid' toegevoegd, omdat het er niet alleen om gaat of de betreffende actor bekwaam en bevoegd is om de preventiemaatregel uit te voeren, maar ook of de betreffende actor de preventiemaatregel procedureel zorgvuldig uitvoert.

Analoog aan het criterium 'alternatieven' onder de legitimiteitsvraag, is het criterium 'subsidiariteit' toegevoegd onder de verantwoordelijkheidsvraag. Het criterium 'subsidiariteit' bevat een waardeoordeel[6], namelijk dat het de voorkeur verdient dat een preventiemaatregel wordt uitgevoerd door een actor zo laag mogelijk in de 'machtshiërarchie'. Bijvoorbeeld: als een preventiemaatregel kan en mag worden uitgevoerd door de nationale overheid of door de gemeentelijke overheid verdient, ceteris paribus, uitvoering door de gemeentelijke overheid de voorkeur. Dit om de afstand tussen de mens wiens vrijheid wordt beperkt en de actor die deze vrijheid beperkt, zo klein mogelijk te houden. De gedachte is dat hierdoor misstanden kunnen worden voorkomen, doordat de actor die de maatregel treft, direct de (neven)effecten kan zien (op microniveau).

6. Het criterium 'subsidiariteit' bevat een waardeoordeel en is dus niet neutraal. Daarom heb ik dit criterium in versie 5 van mijn model weer laten vervallen. Zie de bespreking van 'rij X van overzicht 2' in §2.6.2.

2.5 Vierde versie van het beoordelingsmodel

Discussie in de onderzoeksgroep bracht enkele tekortkomingen van versie 3 van het model aan het licht. Dat leidde tot versie 4. Ik presenteer nu eerst versie 4 en bespreek daarna de wijzigingen ten opzichte van versie 3.

Versie 4 van het beoordelingsmodel (2006)

Gerechtvaardigdheidsvraag (A):

1. Proportionaliteit:
2. Doelmatigheid
3. Toerekenbaarheid, aansprakelijkheid en strafbaarheid
4. Distributieve rechtvaardigheid
5. Acceptatie en handhaving
6. Frequentie en duur
7. Alternatieven

Verantwoordelijkheidsvraag (B):

1. Toepassingsbereik
2. Bekwaamheid en bevoegdheid
3. Geschiktheid
4. Procedurele zorgvuldigheid
5. Subsidiariteit

Wijzigingen ten opzichte van versie 3:

- De overkoepelende ***legitimiteitsvraag*** uit versie 3 is in versie 4 vervangen door een overkoepelende ***gerechtvaardigdheidsvraag***. In de toelichting op versie 2 (§2.3) is al beschreven dat het begrip 'legitimiteit' niet te letterlijk (legitiem = wettelijk) dient te worden genomen. Eén van mijn begeleiders trekt de conclusie dat ik met het begrip 'legitimiteit' eigenlijk doel op het ***Engelse begrip 'justification'***, waarvoor geen equivalent bestaat in het Nederlands. Daarom is in deze versie van het model de legitimiteitsvraag vervangen door de gerechtvaardigdheidsvraag, want het begrip 'gerechtvaardigdheid' ligt dichter bij het bedoelde Engelse begrip 'justification'. Het begrip 'legitimiteit' verwijst te sterk naar de wet, terwijl niet alles wat wettelijk is toegestaan, ook wenselijk is (of wat wettelijk niet is toegestaan, in alle gevallen onwenselijk is).
- Aan de gerechtvaardigdheidsvraag is het criterium 'toerekenbaarheid, aansprakelijkheid en strafbaarheid' toegevoegd (voor deze drie begrippen dien ik nog één overkoepelende term te vinden, die als naam van het criterium kan dienen). De vraag welke partijen of personen verantwoordelijk en aansprakelijk zijn

voor (het risico op) de leefstijlgerelateerde aandoening, is van groot belang voor het beoordelen van de gerechtvaardigdheid van de preventiemaatregel. Worden de 'juiste' (Engels: 'justification') mensen of organisaties belast met de vrijheidsbeperkingen en/of kosten die aan de preventiemaatregel verbonden zijn?
- Aan het criterium 'rechtvaardigheid' is het woord 'distributieve' toegevoegd om verwarring met de overkoepelende 'gerechtvaardigdheidsvraag' te voorkomen. Distributieve rechtvaardigheid c.q. verdelende rechtvaardigheid betreft de vraag of de effecten en neveneffecten van de preventiemaatregel rechtvaardig over de betrokken partijen worden verdeeld.
- Het criterium 'acceptatie en handhaving' is tijdens een bespreking door één van mijn promotoren ingebracht als extra criterium. Accepteert de bevolking (in het bijzonder de doelgroep) de preventiemaatregel en kan de naleving van de maatregel worden gehandhaafd?

Naar aanleiding van de bovenstaande overwegingen heb ik in de onderzoeksvraag (2e versie, 2006) het begrip 'legitimiteit' door het begrip 'gerechtvaardigdheid' vervangen.

Onderzoeksvraag (3e versie, 2006)

> Kan een model worden ontwikkeld op basis waarvan de ***gerechtvaardigdheid*** van preventiemaatregelen ter voorkoming van leefstijlgerelateerde aandoeningen kan worden beoordeeld?

2.6 Vijfde versie van het beoordelingsmodel

Ook versie 4 van mijn model bleek voor verbetering vatbaar. In de §2.6.1 en 2.6.2 licht ik toe waarom en hoe versie 5 tot stand is gekomen.

2.6.1 Criteria van andere beoordelingsmodellen

Bij de ontwikkeling van de 4e versie van het beoordelingsmodel (§2.5) heb ik het begrip 'gerechtvaardigdheid' gekozen als de best mogelijke vertaling van het Engelse begrip 'justification'. De vraag naar de 'justification' van preventiemaatregelen is breder dan de vraag naar de legitimiteit van preventiemaatregelen c.q. de vraag of preventiemaatregelen (wettelijk) zijn toegestaan. 'Justification' vraagt niet enkel om beantwoording van de vraag of een preventiemaatregel mag worden toegepast, maar ook om beantwoording van de vragen of het wenselijk, nodig of noodzakelijk is dat een preventiemaatregel wordt toegepast. Het ***begrip 'noodzake-***

lijk' roept de ***'Trechter van Dunning'*** (1991) in herinnering, waarvan noodzakelijkheid het eerste criterium vormt om te bepalen welke zorg dient te worden opgenomen in het basispakket van de zorgverzekeringen. Dit riep de vragen op of de ***beoordelingscriteria*** en de ***structuur*** (volgorde van de criteria) en/of de ***methodiek*** (trechter met zeven) van de Commissie Dunning (verder te noemen 'Dunning') mogelijk het juiste skelet zouden bieden om het model omheen te bouwen.

Naar aanleiding van de bovenstaande overwegingen heb ik in de literatuur gezocht naar besluitvormingsmodellen op het terrein van de volksgezondheid en gezondheidszorg waarvan ik de criteria, structuur en/of methodiek zou kunnen gebruiken ten behoeve van de ontwikkeling van het beoordelingsmodel. Achtereenvolgens heb ik de volgende vier besluitvormingsmodellen gevonden die hiervoor in aanmerking zouden kunnen komen:
- Trechter van Dunning voor pakketbeslissingen (Dunning, 1991);
- criteria van het College voor zorgverzekeringen (CVZ) voor pakketbeslissingen (Mastenbroek, 2006);
- criteria van Wilson en Jungner voor beslissingen over de aanvaardbaarheid van screening van de bevolking op aandoeningen (Wilson, 1968);
- criteria van de Gezondheidsraad voor de aanvaardbaarheid van genetische screening (Gezondheidsraad, 1994).

Ten behoeve van de ontwikkeling van mijn model heb ik deze modellen kort beschreven (***zie bijlage 4***). Vervolgens heb ik de beoordelingscriteria van deze modellen vergeleken met de criteria van versie 4 van mijn model en op basis daarvan de criteria van mijn model waar nodig aangepast. De vragen of de structuur en de methodiek van de bovengenoemde modellen geschikt zijn voor mijn model, behandel ik in hoofdstuk 3. In dit hoofdstuk beperk ik mij tot de beoordelingscriteria. In overzicht 2 worden de beoordelingscriteria van de commissie Dunning, het CVZ, Wilson en Jungner, de Gezondheidsraad en 'versie 4 van mijn model' met elkaar vergeleken.

Overzicht 2: vergelijking van de beoordelingscriteria van verschillende modellen

	**Commissie Dunning**	**CVZ**	**Wilson en Jungner**	**Gezondheidsraad**	**Versie 4 van mijn model**
	Pakketbeslissingen	Pakketbeslissingen	Aanvaardbaarheid screening	Aanvaardbaarheid genetische screening	Gerechtvaardigdheid preventiemaatregelen gericht op leefstijl
**I**	Noodzakelijkheid (1)	Noodzakelijkheid (rechtvaardigt de ziekte of benodigde zorg een claim op solidariteit, binnen de culturele context) (1)	Belangrijk gezondheidsprobleem (1)	Gezondheidsprobleem (1)<br><br>Doel is deelnemers faciliteren (3)<br><br>Prevalentie (12a)<br><br>Natuurlijk beloop en variatie in ernst aandoening (12b)	
**II**	Werkzaamheid (2)	Effectiviteit (in de breedste zin) (2)	Algemeen aanvaarde behandelwijze (2)<br><br>Herkenbaar latent stadium (4)<br><br>Passende test of onderzoeksmethode (5)<br><br>Inzicht in beloop van ziekte (7)	Zinvolle handelingsopties na testuitslag (4, 12e)<br><br>Geschiktheid testmethode (7)<br><br>Info over natuurlijk beloop en variatie in ernst aandoening (12b)<br><br>Kwaliteit testmethode (12d)	Bekwaamheid en bevoegdheid (B2)<br><br>Geschiktheid (B3)

	Commissie Dunning	CVZ	Wilson en Jungner	Gezondheidsraad	Versie 4 van mijn model
**III**	Doelmatigheid (3)	Kosteneffectiviteit (verhouding kosten/baten in de breedste zin) (3)	Aanvaardbaarheid voor bevolking (6) [duidt op proportionaliteit]<br><br>Verhouding kosten bevolkingsonderzoek versus beschikbare middelen voor volksgezondheid (9)	Bescherming privacy (9)<br><br>Meer voordelen dan nadelen voor deelnemers (12, 12g)<br><br>Belasting voor deelnemers (12d)<br><br>Kans op schade ten gevolge van (test)fouten (12h)<br><br>Kosten van uitvoering (screening en infrastructuur) (12j)	Proportionaliteit (A1)<br><br>Doelmatigheid (A2)
**IV**	Eigen rekening en verantwoording (4)	Noodzakelijkheid (rechtvaardigt de ziekte of benodigde zorg een claim op solidariteit; zo nee, dan zijn eventuele kosten voor eigen rekening) (1)			Toerekenbaarheid, aansprakelijkheid en strafbaarheid (A3)
**V**		Uitvoerbaarheid (haalbaarheid en houdbaarheid van opname in het pakket) (4)	Beschikbaarheid van voorzieningen voor diagnostiek en behandeling (3)<br><br>Continuïteit van bevolkingsonderzoek (10)	Faciliteiten voor vervolgacties (8)<br><br>Kosten van uitvoering (screening en infrastructuur) (12j)	Frequentie en duur (A6)

	**Commissie Dunning**	**CVZ**	**Wilson en Jungner**	**Gezondheidsraad**	**Versie 4 van mijn model**
**VI**			Algemeen aanvaarde behandelwijze (2)<br><br>Aanvaardbaarheid voor bevolking (6)<br><br>Algemeen aanvaard beleid over wie als patiënt wordt behandeld (8)	Informed consent (5)<br><br>Begrijpelijke voorlichting (6)<br><br>Informatie over neven-doelstellingen (10)	Acceptatie en handhaving (A5)
**VII**			Algemeen aanvaard beleid over wie als patiënt wordt behandeld (8)	Doelgroep is duidelijk (2)<br><br>Waarom van gekozen doelgroep en tijdstip in leven van test (12c)	Toepassingsbereik (B1)
**VIII**				Voortdurende kwaliteits-bewaking (11)<br><br>Tijd voor overwegen behandelingsopties (12f)	Procedurele zorgvuldig-heid (B4)
**IX**			Verhouding kosten bevol-kingsonderzoek versus beschikbare middelen voor volksgezondheid (9)	Voorkomen van onge-rechtvaardigde gevolgen (12i)	Distributieve rechtvaar-digheid (A4)
**X**					Alternatieven (A7)<br><br>Subsidiariteit (B5)

Toelichting op de totstandkoming van overzicht 2

Per rij (I t/m X) van overzicht 2 bestaat er een inhoudelijke samenhang tussen de criteria van de verschillende modellen. Ik heb overzicht 2 als volgt van links naar rechts opgebouwd:

- Ik ben begonnen met het plaatsen van de criteria van het model van Dunning in de tweede kolom (rij I t/m IV).
- Vervolgens heb ik per rij (I t/m IV) de overeenkomstige criteria van het model van het CVZ in de derde kolom geplaatst. Omdat het model van Dunning geen criterium 'uitvoerbaarheid' kent, heb ik voor het criterium 'uitvoerbaarheid' van het CVZ een nieuwe rij (V) geopend.
- Vervolgens heb ik per rij (I t/m V) de overeenkomstige criteria van het model van Wilson en Jungner in de vierde kolom geplaatst. Voor de criteria van Wilson en Jungner die inhoudelijk niet overeenkomen met criteria van Dunning of van het CVZ, heb ik de nieuwe rijen (VI en VII) geopend.
- Daarna heb ik per rij (I t/m VII) de overeenkomstige criteria van de Gezondheidsraad in de vijfde kolom geplaatst. Voor de criteria van de Gezondheidsraad die inhoudelijk niet overeenkomen met criteria van Dunning, het CVZ, of Wilson en Jungner, heb ik onderaan de nieuwe rijen (VIII en IX) geopend.
- Ten slotte heb ik per rij (I t/m IX) de overeenkomstige criteria van versie 4 van mijn model in de zesde kolom geplaatst. Voor de criteria van versie 4 die inhoudelijk niet overeenkomen met criteria van de andere modellen, heb ik onderaan een nieuwe rij (X) geopend.
- Achter ieder criterium heb ik tussen haakjes het rangnummer geplaatst dat het criterium in het oorspronkelijke model heeft (zie bijlage 4):
  - Commissie Dunning: criteria 1 t/m 4;
  - CVZ: criteria 1 t/m 4;
  - Wilson en Jungner: criteria 1 t/m 10;
  - Gezondheidsraad: criteria 1 t/m 12 en 12a t/m 12j;
  - Versie 4 van mijn model: criteria A1 t/m A7 en B1 t/m B5 (zie §2.5).

2.6.2 Vergelijking van mijn model met andere beoordelingsmodellen

Onderstaand vergelijk ik de criteria van enerzijds de modellen van Dunning, het CVZ, Wilson en Jungner, en de Gezondheidsraad met anderzijds de criteria van versie 4 van mijn model. Ik maak deze vergelijking aan de hand van de rijen (I t/m X) van overzicht 2. Op basis van deze vergelijking ontwikkel en bespreek ik de criteria van versie 5 van mijn model (zie verderop in deze subparagraaf).

Rij I van overzicht 2: in het model ontbreekt een criterium

Wat opvalt in rij I van overzicht 2 is het ontbreken van een criterium in mijn model. De eerste rij van overzicht 2 betreft de volgende vragen:

- Is het treffen van de preventiemaatregel wel 'noodzakelijk' (criterium van Dunning en van het CVZ)?
- Is er wel sprake van een (belangrijk) gezondheidsprobleem (criterium van Wilson en Jungner en van de Gezondheidsraad)?

Het eerste criterium van versie 4 van mijn model is 'proportionaliteit'. De proportionaliteit betreft de vraag of de beoogde effecten van de preventiemaatregel (gezondheidswinst) opwegen tegen de neveneffecten (bv. verlies aan keuzevrijheid en economisch nadeel). Naar aanleiding van de eerste rij van overzicht 2 ben ik tot het inzicht gekomen dat aan het criterium 'proportionaliteit' de vraag vooraf hoort te gaan of het überhaupt wel wenselijk of noodzakelijk is om het gestelde doel na te streven. Is de doelstelling van de preventiemaatregel wel gerechtvaardigd? In een uitgebreide studie van de Britse overheid naar procedures voor rationele beleidsontwikkeling, wordt eveneens benadrukt dat de ontwikkeling van nieuw beleid altijd dient te beginnen met de vraag of hiertoe wel een noodzaak bestaat (HM Treasury, 2003, p.4).

Ter toelichting van de vraag naar de gerechtvaardigdheid van de doelstelling van de preventiemaatregel volgen enkele voorbeelden van vragen die men zich over de gerechtvaardigdheid van de doelstelling kan stellen:

- In plaats van 'eindeloos' door te zoeken naar preventiemaatregelen om overgewicht te bestrijden (doel: BMI < 25), kan men zich ook ten doel stellen om alleen obesitas (ernstig overgewicht) te bestrijden (doel: BMI < 30), of om alleen ziekelijk overgewicht te bestrijden (doel: BMI < 40). Is het bestrijden van overgewicht (doel BMI < 25) wel een te rechtvaardigen doelstelling van het volksgezondheidsbeleid of is dit een vorm van onwenselijk ***perfectionisme*** (onwenselijk streven naar volmaaktheid van de mens)?
- Is een wettelijk verbod op het gebruik van harddrugs, als de democratische meerderheid van de bevolking het gebruik van harddrugs moreel verwerpelijk acht, een vorm van onwenselijk ***moralisme*** (opdringen van normen en waarden aan mensen, zonder dat de afwijking van deze normen en waarden derden benadeelt)?
- Is het verbieden van het gebruik van softdrugs, omdat dit kan ontaarden in het gebruik van harddrugs, niet een onwenselijke vorm van ***paternalisme*** (onwenselijk beschermen van volwassenen tegen zichzelf)?
- Een op zichzelf zinvolle doelstelling van het volksgezondheidsbeleid (terugdringen van overgewicht) kan een andere zinvolle doelstelling (terugdringen

van roken) ***verdringen.*** Als het ineffectief of praktisch onuitvoerbaar is om het preventiebeleid tegelijkertijd op beide doelstellingen te richten, dient de belangrijkste doelstelling prioriteit te krijgen (zie criterium 9 in het model van Wilson en Jungner).

Eigenlijk gaat het om de vraag of de preventiemaatregel wel 'moet' worden uitgevoerd. De vraag 'wat moet' was een vraag in versie 1 van het model. In versie 2 heb ik deze vraag beargumenteerd laten vervallen. Bij de bespreking van versie 1 heb ik geconcludeerd dat de vraag 'wat moet' leidt tot ideologische discussies over perfectionisme, moralisme en paternalisme (zie ter illustratie de bovenstaande voorbeelden). Ideologische discussies lijken haaks te staan op mijn streven om een neutraal beoordelingsmodel te ontwikkelen. Door het bestuderen van literatuur over de preventie van leefstijlgerelateerde aandoeningen ben ik echter tot de conclusie gekomen dat ideologische discussies onlosmakelijk verbonden zijn met het beoordelen van de gerechtvaardigdheid van preventiemaatregelen. In §3.2 zal ik deze stelling aan de hand van literatuur onderbouwen. In versie 5 van mijn model keert de vraag 'wat moet' daarom terug in het model en wel op de plaats van het criterium 'schadelijkheid' (1e criterium).

Versie 5 van het beoordelingsmodel (2007)

Doelstelling
1. Schadelijkheid
2. Verantwoordelijkheid
3. Gerichtheid
4. Alternatieven

Effecten
1. Kosteneffectiviteit
2. Proportionaliteit
3. Rechtvaardigheid
4. Alternatieven

Uitvoering
1. Aanvaardbaarheid
2. Uitvoerbaarheid
3. Zorgvuldigheid
4. Alternatieven

Een eerste verschil tussen versie 1 en versie 5 van mijn model m.b.t. de vraag 'wat moet' is de plaats van deze vraag in het model. In versie 1 was de vraag 'wat moet' de voorlaatste vraag ('wie moet' was de laatste vraag). In versie 5 wordt de vraag 'wat moet' direct aan het begin gesteld. Deze omkering is essentieel. De beoordeling van de gerechtvaardigdheid van een preventiemaatregel dient te beginnen met de beoordeling van de gerechtvaardigdheid van de doelstelling van de preventiemaatregel en niet met het beoordelen van de gevolgen c.q. effecten en neveneffecten van de maatregel (criterium 'proportionaliteit' in versie 4 van mijn model). Een tweede verschil tussen versie 1 en versie 5 van mijn model m.b.t. de vraag *'wat moet'* is de reikwijdte van deze vraag. In versie 1 was de vraag '*welke preventiemaatregelen moeten worden uitgevoerd?*', terwijl de vraag in versie 5 is '*moet deze preventiemaatregel wel worden uitgevoerd?*'. Probleem bij de beantwoording van de vraag *'welke preventiemaatregelen moeten worden uitgevoerd?'* is dat deze vraag betrekking heeft op een oneindig aantal denkbare preventiemaatregelen. De vraag *'moet deze preventiemaatregel wel worden uitgevoerd?'* betreft maar één preventiemaatregel en is daardoor beter te beantwoorden.

De beoordeling van de gerechtvaardigdheid van de doelstelling van de preventiemaatregel leidt, zoals toegelicht, tot ideologische vragen over de perfectionistische, paternalistische en morele aspecten van de preventiemaatregel en tot de vraag of met de preventiemaatregel niet een zinvollere doelstelling van het preventiebeleid wordt 'verdrongen'. Afhankelijk van individuele voorkeuren leiden met name de ideologische vragen tot uiteenlopende subjectieve antwoorden. In een streven om de beantwoording van de ideologische vragen te objectiveren, heb ik gezocht naar algemene uitgangspunten voor de beoordeling van (de gerechtvaardigdheid van) de perfectionistische, paternalistische en morele aspecten van preventiemaatregelen waarover binnen de samenleving een brede consensus bestaat. Ik meen dat het schadebeginsel van John Stuart Mill dergelijke algemeen aanvaarde uitgangspunten biedt.

In de literatuur wordt het schadebeginsel (en de vele bewerkingen daarvan) van de Engelse filosoof John Stuart Mill genoemd als principe om te bepalen of een interventie van de overheid in het persoonlijke leven van burgers gerechtvaardigd is. De oorspronkelijke formulering van het schadebeginsel is afkomstig uit 'On liberty', dat Mill in 1859 publiceerde (Maris, 2006, p.92 e.v.) (Mill, 1859):

> *"Dit principe is, dat (...) de enige reden waarom men rechtmatig macht kan uitoefenen over enig lid van een beschaafde samenleving, tegen zijn zin de zorg is dat anderen geen schade wordt toegebracht. Iemands eigen welzijn, hetzij fysiek, hetzij moreel, is geen voldoende rechtsgrond. Men kan iemand niet met recht dwingen iets te doen of te laten, omdat het beter voor hem zou zijn als hij dat deed, omdat het*

hem gelukkiger zou maken, omdat anderen het wijs of zelfs rechtvaardig zouden vinden als hij dat deed (...). Iemand is de maatschappij slechts verantwoording schuldig voor zover zijn gedrag anderen aangaat. Waar hij slechts met zichzelf te maken heeft is hij, met alle recht, volmaakt onafhankelijk. Over zichzelf, over zijn eigen lichaam en geest, is het individu soeverein".

Volgens Mill mag de overheid alleen interveniëren in het persoonlijke leven van burgers voor zo ver het gedrag van deze burgers schade toebrengt aan de rechtmatige belangen van derden (Maris, 2006, p.93). De overheid is volgens hem niet gerechtvaardigd om mensen met vrijheidsbeperkende maatregelen tegen zichzelf (c.q. tegen hun eigen ongezonde gedrag) te beschermen. Voor letterlijke slavernij maakt Mill een uitzondering op zijn afwijzing van overheidspaternalisme. Volgens hem dient de overheid mensen wel te beschermen tegen slavernij ook als mensen vrijwillig voor een leven als slaaf (lijken te) kiezen (Maris, 2006, p.99). Voor het overige worden moralisme en paternalisme van de overheid door Mill resoluut afgewezen. Hij is hiermee een vertegenwoordiger van het klassieke liberalisme. Binnen het liberalisme wordt ook algemeen geaccepteerd (dus ook binnen het klassieke liberalisme) dat de overheid kinderen en geesteszieken tegen zelfbeschadiging beschermt (Maris, 2006, p.99).

Sinds 1851 is het klassieke liberalisme door diverse liberale denkers genuanceerd door wel bepaalde vormen van overheidspaternalisme toe te staan. Zo maakt Joel Feinberg onderscheid tussen hard en zacht paternalisme, waarbij hij hard paternalisme niet, maar zacht paternalisme wel aanvaardbaar acht (Feinberg, 1980, 1986). Een andere nuancering van het klassieke liberalisme wordt gevormd door het 'libertair paternalisme' (Sunstein, 2003). Voor het ontwerp van het beoordelingsmodel zijn niet de uiteenlopende visies op de (on)aanvaardbaarheid van overheidspaternalisme en overheidsmoralisme van belang, maar een aantal algemeen aanvaarde uitgangspunten, dat van het schadebeginsel kan worden afgeleid (zie overzicht 3).

Overzicht 3: algemeen aanvaarde uitgangspunten voor beoordeling van aanvaardbaarheid van preventie

a. Preventie van ***schade aan rechtmatige belangen*** vormt een sterkere rechtvaardiging van een bepaalde leefstijlbeïnvloedende interventie dan ***bevordering van rechtmatige beleidsdoelen***[7]**.**

7. Ter illustratie enkele voorbeelden van rechtmatige beleidsdoelen:
   – meer bewegen door de jeugd;
   – meer burenhulp voor bejaarden;

b. Preventie van ***schade of overlast*** vormt een sterkere rechtvaardiging van een bepaalde leefstijlbeïnvloedende interventie dan preventie van de ***schending van morele opvattingen***.
c. Preventie van schade die men berokkent ***aan derden*** vormt een sterkere rechtvaardiging van een bepaalde leefstijlbeïnvloedende interventie dan preventie van schade die men berokkent ***aan zichzelf*** (als het gaat om 'dezelfde' schade).
d. Preventie van schade die ***kinderen*** berokkenen aan zichzelf vormt een sterkere rechtvaardiging van een bepaalde leefstijlbeïnvloedende interventie dan preventie van schade die ***volwassenen*** berokkenen aan zichzelf (als het gaat om 'dezelfde' schade).
e. Naarmate de ***autonomie, keuzevrijheid, wilsbekwaamheid en geïnformeerdheid*** van mensen groter is, is paternalisme minder gerechtvaardigd.

In deze paragraaf zal ik kort toelichten waarom ik deze uitgangspunten algemeen aanvaardbaar acht (zie ook de tekst bij overzicht 9 in §4.3). In de literatuur luidt de kritiek op de klassieke opvatting van het schadebeginsel (zoals geformuleerd door Mill) 'nooit' dat deze te veel preventieve interventies (van de overheid) toestaat, maar altijd dat de klassieke opvatting te weinig preventieve interventies toestaat (dat paternalistische en moralistische preventiemaatregelen te rigide worden afgewezen). Dit betekent dat als de toepassing van een vrijheidsbeperkende preventiemaatregel op basis van de klassieke opvatting van het schadebeginsel kan worden gerechtvaardigd, deze rechtvaardiging voor iedereen c.q. algemeen aanvaardbaar is. De klassieke opvatting van het schadebeginsel is niet algemeen aanvaardbaar, omdat deze opvatting volgens veel mensen te weinig paternalisme en te weinig moralisme toestaat. Door de klassieke opvatting van het schadebeginsel niet absoluut maar relatief te formuleren[8], vervalt dit bezwaar, en zijn de uitgangspunten (overzicht 3), die ik heb afgeleid van de klassieke opvatting van het schadebeginsel,

– economische groei van 3% per jaar;
– meer gelijkheid op de arbeidsmarkt tussen vrouwen en mannen en tussen allochtonen en autochtonen.

8. Ter illustratie een voorbeeld van een absolute formulering en relatieve formulering van hetzelfde uitgangspunt:
   – absolute formulering: de overheid is ***wel*** gelegitimeerd om in te grijpen in het leven van mensen ter preventie van schade aan derden, maar ***niet*** ter preventie van schade die mensen toebrengen aan zich zelf.
   – relatieve formulering: de overheid is ***sterker*** gelegitimeerd om in te grijpen in het leven van mensen ter preventie van schade aan derden, dan ter preventie van schade die mensen toebrengen aan zich zelf.

wel algemeen aanvaardbaar. Ik ga hierbij uit van algemene aanvaardbaarheid in Westerse samenlevingen. In samenlevingen waarin kerk en staat niet gescheiden zijn, kan de preventie van schending van geloofsopvattingen (een geloofsopvatting is een morele opvatting) wel zwaarder wegen dan de preventie van schade. In dergelijke samenlevingen zijn de uitgangspunten a en b uit overzicht 3 niet algemeen aanvaardbaar (met name niet in geval van geloofskwesties).

Binnen het kader van het schadebeginsel heeft het begrip 'schade' een andere betekenis dan in het gewone taalgebruik (Feinberg, 1984, p.32). In ***bijlage 5*** heb ik het ***begrip 'schade'*** ten behoeve van het beoordelingsmodel gedefinieerd als:
- aantasting van gezondheidsbelangen;
- aantasting van sociaal-economische belangen;
- schending van individuele mensenrechten.

Tevens heb ik in bijlage 5 het begrip 'schade' afgebakend van de begrippen 'overlast' en 'schending van morele opvattingen'.
Om de in overzicht 3 genoemde, algemeen aanvaarde, uitgangspunten een plaats te geven in mijn beoordelingsmodel, heb ik de volgende criteria aan het begin van het model toegevoegd:
- schadelijkheid;
- verantwoordelijkheid;
- gerichtheid.

Aan de hand van deze drie criteria wordt de gerechtvaardigdheid van de doelstelling van de preventiemaatregel beoordeeld.

Criterium schadelijkheid
Het criterium 'schadelijkheid' betreft de aard en omvang van de risico's op (zie bijlage 5):
- schade,
- overlast, en
- schending van morele opvattingen.

Het gaat hierbij om de risico's die men met de preventiemaatregel tracht te bestrijden. Ik heb gezocht naar een criterium voor het beoordelingsmodel dat overkoepelend is voor de beoordeling van de risico's op schade, op overlast en op schending van morele opvattingen. Uiteindelijk ben ik toch bij de benaming 'schadelijkheid' uitgekomen. Deze benaming heeft als bezwaar dat 'overlast' en 'schending van morele opvattingen' in de literatuur in het algemeen niet als schade worden erkend. De term 'schade' wordt in het algemeen gereserveerd voor de aantasting van basisbelangen in de zin van het schadebeginsel. De term 'schadelijkheid' heeft

als essentieel voordeel dat wordt benadrukt dat in het model de preventie van empirisch vast te stellen schade (ten gevolge van een ongezonde leefstijl), in plaats van de bevordering van morele beleidsdoelstellingen, het uitgangspunt vormt voor het beoordelen van de gerechtvaardigdheid van leefstijlbeïnvloedende preventiemaatregelen (zie punt a in overzicht 3). Bovendien benadrukt de term 'schadelijkheid' dat de schade aan basisbelangen in het algemeen zwaarder weegt dan de schending van morele opvattingen (zie punt b in overzicht 3). Het vaststellen van de schadelijkheid van de ongezonde leefstijl is van belang om te beoordelen in hoeverre de preventiemaatregel perfectionistisch of moralistisch is. Aan de hand van het criterium 'schadelijkheid' kan ook worden vastgesteld of met de preventiemaatregel niet een zinvollere doelstelling van het preventiebeleid wordt 'verdrongen' (het richten van het preventiebeleid op een leefstijl die tot nog meer schade leidt).

Criterium verantwoordelijkheid

De betekenis van het criterium 'verantwoordelijkheid' is tweeledig. Enerzijds betreft het de vraag welke partijen verantwoordelijk zijn voor het ontstaan en/of de instandhouding van de risico's die men met de preventiemaatregel tracht te bestrijden. Anderzijds betreft het de vraag welke partijen verantwoordelijk zijn voor de risicopreventie. Inzicht in beide vragen is van belang om te beoordelen in hoeverre er sprake is van paternalisme. De criteria 'bekwaamheid en bevoegdheid' en 'geschiktheid' uit versie 4 van mijn model (zie rij II van overzicht 2) maken onderdeel uit van het criterium 'verantwoordelijkheid' uit versie 5. In versie 5 vervallen de 'bekwaamheid en bevoegdheid' en 'geschiktheid' daarom als zelfstandige criteria.

Criterium gerichtheid

Bij het criterium 'gerichtheid' gaat het om de vraag of de preventiemaatregel is gericht op de juiste doelgroep of op de juiste omgevingsfactoren (zie rij VII van overzicht 2). Is de preventiemaatregel een vorm van symptoombestrijding of pakt de preventiemaatregel het ontstaan van de risico's die men wil bestrijden bij de bron aan. Is de preventiemaatregel gericht op de partijen die verantwoordelijk zijn voor het ontstaan en/of de instandhouding van de risico's die men wil bestrijden, of op anderen?

Rij II en III van overzicht 2: baten en lasten

In rij II betreffen de criteria van de andere modellen de:

- werkzaamheid (Dunning);
- effectiviteit (CVZ);
- 'behandelbaarheid' (Wilson en Jungner, en de Gezondheidsraad).

In rij III betreffen de criteria van de andere modellen de:
- doelmatigheid (Dunning);
- kosteneffectiviteit (CVZ);
- verhouding kosten bevolkingsonderzoek versus beschikbare middelen voor volksgezondheid (Wilson en Jungner);
- verhouding van voor- en nadelen voor deelnemers (Gezondheidsraad).

De criteria in rij II betreffen alleen de beoogde baten van de interventie. De criteria in rij III betreffen een vergelijking tussen enerzijds de beschikbare middelen of beoogde baten en anderzijds bepaalde kosten of lasten van de interventie. Daardoor kunnen de criteria in rij II worden gezien als een deelverzameling van de criteria in rij III.

In rij III betreft het criterium:
- 'doelmatigheid' (criterium van Dunning) de verhouding tussen de beoogde effecten en de uitvoeringskosten van de interventie, <u>exclusief</u> de baten en lasten van de neveneffecten;
- 'meer voordelen dan nadelen voor deelnemers' (criterium van de Gezondheidsraad) de verhouding tussen alle baten en lasten van de interventie, <u>exclusief</u> de baten en lasten voor niet-deelnemers;
- 'verhouding kosten bevolkingsonderzoek versus beschikbare middelen voor volksgezondheid' (criterium van Wilson en Jungner) de verhouding tussen uitvoeringskosten en beschikbare middelen voor volksgezondheid, <u>exclusief</u> de baten en lasten van effecten en neveneffecten.

Het begrip 'kosteneffectiviteit' wordt door het CVZ als volgt gedefinieerd (Mastenbroek, 2006, p.38):

> *"Is de verhouding tussen de kosten en baten in de breedste zin acceptabel?"*
>
> *"Het CVZ gaat bij voorkeur uit van een kostenutiliteitsanalyse. Wat zijn de kosten van deze zorgvoorziening per gewonnen levensjaar, gecorrigeerd voor kwaliteit (Quality Adjusted Life Year / QALY)? De uitkomst moeten we wel plaatsen binnen de context waarin de zorgvorm wordt ingezet (indicatie, zorgverlener). … De term 'baten' kunnen we in dit verband breed opvatten en we kunnen het daarbij zowel binnen als buiten het terrein van de gezondheidszorg zoeken."*

Lasten voor derden, zoals beperkingen van de keuzevrijheid en privacy, lijken hier buiten te vallen, terwijl deze voor het model zo belangrijk zijn. Bij preventiemaatregelen ter bestrijding van leefstijlgerelateerde aandoeningen spelen de baten en lasten voor derden (bijvoorbeeld verlies van keuzevrijheid of privacy voor mensen die niet tot de doelgroep van de maatregel behoren) een belangrijke rol bij het

beoordelen van de gerechtvaardigdheid van de maatregel. Ik geef daarom de voorkeur aan mijn criterium ***'proportionaliteit'***, dat ***alle baten en lasten voor iedereen*** betreft, boven de hierboven genoemde criteria van Dunning, de Gezondheidsraad, Wilson en Jungner, en het CVZ, die niet alle baten en lasten betreffen.

Het criterium 'proportionaliteit' is in veel gevallen moeilijk te objectiveren. Dat komt met name doordat het verlies aan keuzevrijheid en privacy moeilijk in maat en getal zijn uit te drukken. Daarom kies ik er voor om toch ook het criterium 'kosteneffectiviteit' op te nemen, omdat dit bij de huidige stand van de wetenschap beter te objectiveren is dan het criterium 'proportionaliteit'. Het criterium 'kosteneffectiviteit' vormt in versie 5 van mijn model een deelverzameling van het criterium 'proportionaliteit', dat alle baten en lasten omvat'. Het objectiveren van de kosteneffectiviteit is mogelijk door deze te kwantificeren. Dit kan bijvoorbeeld door de kosteneffectiviteit uit te drukken in:
- sterftereductie;
- QALY's (Quality Adjusted Life Years) (zie bv. Mastenbroek, 2006);
- DALY's (Disability Adjusted Life Years) (zie bv. Toenders, 2001);
- vermindering van ziektelast (zie bv. Wieringa, 2003).

Het criterium 'kosteneffectiviteit' definieer ik in het kader van mijn model als de verhouding tussen de uitvoeringskosten en de beoogde effecten van de interventie (dus minder breed dan de definitie van het CVZ). Ter illustratie een voorbeeld van een mogelijke kwantificering van de kosteneffectiviteit passend binnen het kader van het model (Bonsel, 2004):

> *Uit een onderzoek naar de kosteneffectiviteit van het opsporen en preventief behandelen van familiaire hypercholesterolemie is een kosteneffectiviteitsratio naar voren gekomen van € 25.000,– per gewonnen levensjaar.*

De baten en lasten van neveneffecten (bijvoorbeeld minder ziekteverzuim, hogere pensioenkosten, of verlies van keuzevrijheid) worden in mijn model bij het bepalen van de kosteneffectiviteit buiten beschouwing gelaten. Bij de beoordeling van de 'proportionaliteit' van de preventiemaatregel worden de baten en lasten van neveneffecten wel meegewogen.

De criteria 'doelmatigheid' (een criterium van Dunning, en een criterium in versie 4 van mijn model) en 'kosteneffectiviteit' lijken op elkaar. Voor versie 5 geef ik de voorkeur aan de benaming 'kosteneffectiviteit', omdat deze benaming duidelijker weergeeft hoe het criterium geoperationaliseerd c.q. gekwantificeerd en daarmee geobjectiveerd kan worden.

Rij IV van overzicht 2: eigen rekening en verantwoording

Opvallend is het ontbreken van een criterium betreffende de eigen verantwoordelijkheid voor de kosten van de preventiemaatregel in de modellen van 'Wilson en Jungner' en de Gezondheidsraad. De modellen van Dunning, het CVZ en het model bevatten wel een dergelijk criterium. Ik meen dat een dergelijk criterium in mijn model thuishoort, waarbij het niet alleen gaat om de eigen verantwoordelijkheid van mensen voor de kosten van de preventiemaatregel, maar ook om het zelf dragen van de overige lasten van de preventiemaatregel (bijvoorbeeld een beperkte keuzevrijheid of privacy).

De 'toerekenbaarheid, aansprakelijkheid en strafbaarheid' (criterium in versie 4 van mijn model) worden niet alleen bepaald door de 'verantwoordelijkheid voor het risico op schade' (criterium in versie 5), maar ook door overwegingen op het gebied van 'rechtvaardigheid' (criterium in versie 5). Daarom vervalt in versie 5 het criterium 'toerekenbaarheid, aansprakelijkheid en strafbaarheid' als zelfstandig criterium. De 'toerekenbaarheid, aansprakelijkheid en strafbaarheid' komen in versie 5 aan de orde bij de criteria 'verantwoordelijkheid', 'gerichtheid' en 'rechtvaardigheid'.

Rij V van overzicht 2: uitvoerbaarheid

Met uitzondering van het model van Dunning wordt in alle modellen van overzicht 2 aandacht besteed aan de uitvoerbaarheid (haalbaarheid en houdbaarheid) van de interventie in de praktijk. De 'haalbaarheid' betreft de vraag of kwantitatief en kwalitatief voldoende mensen en middelen beschikbaar zijn om de maatregel op verantwoorde wijze in te voeren. De 'houdbaarheid' betreft de vraag of de beschikbaarheid van voldoende mensen en middelen, om de maatregel op verantwoorde wijze uit te voeren, voor langere termijn gegarandeerd kan worden. Zo wil het CVZ bijvoorbeeld voorkomen dat zorgproducten die in een bepaald jaar in het basispakket worden opgenomen, na één of enkele jaren weer uit het basispakket moeten worden verwijderd wegens te hoge kosten. Versie 4 van mijn model kent in rij V een criterium 'frequentie en duur'. Bij nader inzien vind ik het begrip 'frequentie en duur' geen geschikte benaming voor een criterium, omdat het meer beschrijvend dan toetsend van aard is. Daarom geef ik de voorkeur aan het criterium 'uitvoerbaarheid' (haalbaarheid en houdbaarheid) van het CVZ, omdat dit goed onder woorden brengt wat dient te worden getoetst en algemener c.q. breder is geformuleerd dan de criteria van Wilson en Jungner en de Gezondheidsraad.

Rij VI van overzicht 2: aanvaardbaarheid

De gerechtvaardigdheid van een preventiemaatregel wordt mede bepaald door de vraag of de maatregel aanvaardbaar is voor de bevolking (criterium 'aanvaardbaarheid' van Wilson en Jungner) of voor het individu (criterium 'informed consent' van de Gezondheidsraad). Uitgangspunt van de modellen van 'Wilson en Jungner' en de Gezondheidsraad is vrijwilligheid van de screening. Uitgangspunt van mijn model is dat preventiemaatregelen ter bestrijding van leefstijlgerelateerde ook onvrijwillig kunnen worden opgelegd. Deze onvrijwilligheid betekent echter niet dat de aanvaardbaarheid geen criterium zou moeten zijn in mijn model (zie het criterium 'acceptatie en handhaving' in versie 4 van mijn model). Ik zal deze stelling onderbouwen aan de hand van het wettelijk rookverbod dat per 1 juli 2008 is ingevoerd in de Nederlandse Horeca.

Enerzijds zou de invoering van een rookverbod dat niet aanvaardbaar is voor de bevolking op gespannen voet staan met het democratische uitgangspunt dat wet- en regelgeving gesteund dienen te worden door de meerderheid van de bevolking. Als de besluitvorming over de invoering van het rookverbod democratisch (door een besluit van het parlement) tot stand is gekomen, is dat in principe voldoende om het rookverbod m.b.t. het aspect 'aanvaardbaarheid voor de bevolking' te rechtvaardigen. Als de meerderheid van de bevolking tegen het verbod zou zijn, zou dat echter vragen kunnen oproepen over het democratisch gehalte van de parlementaire besluitvorming. Anderzijds kan de invoering van een rookverbod dat niet aanvaardbaar is voor de bevolking of voor de doelgroep, problemen opleveren voor de handhaving van dit rookverbod. De handhaafbaarheid van het rookverbod in de Horeca wordt in grote mate bepaald door de acceptatie van deze maatregel door de Horecahouders en -bezoekers, maar ook door het draagvlak voor deze maatregel binnen de bevolking. Naarmate de acceptatie van en draagvlak voor het rookverbod groter zijn, worden de uitvoeringskosten voor handhaving van het rookverbod (veel) lager. Op dit punt interacteert het criterium 'aanvaardbaarheid' (rij VI van overzicht 2) met het criterium 'uitvoerbaarheid' (rij V van overzicht 2). Ondanks deze interactie acht ik het van belang om de criteria 'aanvaardbaarheid' en 'uitvoerbaarheid' van elkaar te onderscheiden. Voldoende acceptatie van en draagvlak voor een vrijheidsbeperkende preventiemaatregel vormen voorwaarden om de uitvoering überhaupt in overweging te nemen. Daarom stel ik voor om binnen versie 5 van mijn model eerst te kijken naar de 'aanvaardbaarheid' van de maatregel voor de betrokken partijen en pas daarna naar de 'uitvoerbaarheid' van de maatregel (de beschikbaarheid van voldoende mensen en middelen voor de uitvoering).

Als ik moet kiezen uit de begrippen 'aanvaardbaarheid' van Wilson en Jungner, 'informed consent' van de Gezondheidsraad en 'acceptatie' uit versie 4 van mijn model, geef ik de voorkeur aan het begrip 'aanvaardbaarheid'. Het begrip 'informed consent' (geïnformeerde instemming) veronderstelt vrijwilligheid en valt daarom af als (algemeen) criterium binnen het model. Vanuit taalkundig perspectief geef ik de voorkeur aan de benaming 'aanvaardbaarheid' boven de benaming 'acceptatie'. De reden is dat de benaming 'aanvaardbaarheid' beter aangeeft dat het om een criterium gaat. Met het oog op de handhaafbaarheid van een vrijheidsbeperkende preventiemaatregel kan zowel het draagvlak onder de bevolking als de acceptatie door de doelgroep en het individu van belang zijn. In versie 5 van mijn model betreft het criterium 'aanvaardbaarheid' daarom zowel de bevolking, de doelgroep, als het individu.

Rij VII van overzicht 2: doelgroep van de interventie

Aangaande de doelgroep van de interventie bestaan er belangrijke verschillen tussen enerzijds interventies in de individuele gezondheidszorg (modellen van Dunning en het CVZ) en screening (modellen van Wilson en Jungner, en de Gezondheidsraad) en anderzijds preventiemaatregelen ter bestrijding van leefstijlgerelateerde aandoeningen (mijn beoordelingsmodel):

- interventies in de individuele gezondheidszorg en screening:
  - richten zich in het algemeen op mensen met (risico op) de gezondheidsproblemen waarop de interventie zich richt;
  - worden in principe alleen vrijwillig toegepast op basis van informed consent.
- preventiemaatregelen ter bestrijding van leefstijlgerelateerde aandoeningen:
  - kunnen zich ook richten op mensen zonder (risico op) gezondheidsproblemen waarop de interventie zich richt, of op omgevingsfactoren;
  - kunnen ook onvrijwillig worden toegepast.

Bij de bespreking van rij I van overzicht 2 heb ik ervoor gepleit om de gerechtvaardigdheid van (onvrijwillige) interventies in de leefstijl van mensen mede te beoordelen vanuit het perspectief van het schadebeginsel. Om dit te kunnen doen, is inzicht in de samenstelling van de doelgroep en de omgevingsfactoren waarop de preventiemaatregel zich richt, essentieel. In versie 5 van mijn beoordelingsmodel wordt daarom het criterium 'gerichtheid' van de preventiemaatregel opgenomen, dat ondermeer betrekking heeft op de juistheid (Engels: justification) van de doelgroep of van de omgevingsfactoren waarop de preventiemaatregel zich richt. Het criterium 'toepassingsbereik' uit versie 4 van mijn model, wordt onderdeel van het criterium 'gerichtheid' in versie 5 en vervalt dus als zelfstandig criterium.

Rij VIII van overzicht 2: kwaliteitsbewaking

Rij VIII kent de criteria 'voortdurende kwaliteitsbewaking' van de Gezondheidsraad en 'procedurele zorgvuldigheid' van versie 4 van mijn model. Een criterium 'voortdurende kwaliteitsbewaking' is goed toepasbaar in geval van (genetische) screening, maar minder van toepassing in geval van preventiemaatregelen gericht op omgevingsfactoren. Ook in geval van wetgeving, bijvoorbeeld accijnzen op genotsmiddelen, is een criterium 'voortdurende kwaliteitsbewaking' minder van toepassing. Ik kies daarom voor handhaving van mijn criterium 'procedurele zorgvuldigheid'. Omdat de andere criteria in versie 5 van mijn model ook geen bijvoeglijk naamwoord kennen, laat ik het bijvoeglijk naamwoord 'procedurele' in versie 5 vervallen. Het criterium wordt dus 'zorgvuldigheid'.

Rij IX van overzicht 2: rechtvaardigheid

Op basis van voortschrijdend inzicht maak ik het volgende onderscheid tussen distributieve en vergelijkende rechtvaardigheid:

- ***Distributieve rechtvaardigheid*** betreft de vraag of de baten en lasten van de preventiemaatregel rechtvaardig over de betrokken partijen worden verdeeld. Hierbij zijn verschillende invalshoeken van belang:
  - Worden bepaalde partijen niet ***disproportioneel*** benadeeld door de preventiemaatregel in vergelijking tot het risico op gezondheidsschade waarvoor zij verantwoordelijk worden geacht? Ter illustratie een voorbeeld. Men zou kunnen redeneren dat een wettelijk rookverbod in de Horeca houders van kleine cafés disproportioneel benadeelt. Hun cafés zijn te klein om een aparte rookruimte in te richten. Hierdoor zouden veel klanten weg kunnen blijven; bijvoorbeeld door thuis te blijven of uit te wijken naar grotere cafés met een aparte rookruimte. Dat zou kunnen leiden tot faillissementen van kleine cafés. Men zou van opvatting kunnen zijn dat de 'lasten' van een faillissement disproportioneel zijn in vergelijking tot het risico op gezondheidsschade waarvoor de houders van kleine cafés medeverantwoordelijk zijn.
  - Worden bepaalde partijen niet ***onevenredig*** benadeeld door de preventiemaatregel ten opzichte van andere partijen? Zie ter illustratie nogmaals het bovenstaande voorbeeld. Men zou kunnen redeneren dat een wettelijk rookverbod in de Horeca houders van kleine cafés onevenredig benadeelt ten opzichte van houders van grote cafés.
  - Worden bepaalde partijen niet ***onterecht*** benadeeld door de preventiemaatregel, omdat zij niet verantwoordelijk zijn voor het risico op schade dat met de preventiemaatregel wordt bestreden? Denk bijvoorbeeld aan hoge accijnzen op alcohol ter voorkoming van alcoholmisbruik, die ook betaald moeten worden door mensen zonder neiging tot alcoholisme.

- Dienen de baten en lasten van de preventiemaatregel niet (ook) te worden verdeeld naar ***draagkracht***? Een ongezonde leefstijl komt vaker voor bij mensen met een lage sociaal-economische status (SES) dan bij mensen met een hoge SES. Als een preventiemaatregel mensen bijvoorbeeld financieel aansprakelijk stelt voor zorgkosten ten gevolge van hun ongezonde leefstijl, worden mensen met een lage SES 'dubbel' hard getroffen. Zij hebben vaker een ongezonde leefstijl en beschikken over minder geld om zorgkosten te betalen. In hoeverre is dat rechtvaardig?

- ***Vergelijkende rechtvaardigheid*** betreft de vraag of in vergelijkbare situaties vergelijkbaar wordt gehandeld. Zou het bijvoorbeeld niet onrechtvaardig zijn om rokers wel financieel aansprakelijk te stellen voor zorgkosten ten gevolge van roken, maar alcoholisten niet financieel aansprakelijk te stellen voor zorgkosten ten gevolge van alcoholisme? Zou het bijvoorbeeld niet onrechtvaardig zijn om een cursus 'stoppen met roken' wel te vergoeden, maar een cursus stoppen met alcoholmisbruik niet te vergoeden?

Voor mijn beoordelingsmodel acht ik zowel de distributieve als vergelijkende rechtvaardigheid van belang. In versie 5 van mijn model vervang ik de term 'distributieve rechtvaardigheid' uit versie 4 daarom door de algemene (overkoepelende) term 'rechtvaardigheid'. Het bezwaar dat ik eerder heb genoemd, dat een criterium 'rechtvaardigheid' erg lijkt op de overkoepelende term 'gerechtvaardigdheid' van de preventiemaatregel, neem ik hierbij voor lief.

Rij X van overzicht 2: alternatieven

Het criterium 'alternatieven' komt alleen voor in mijn eigen beoordelingsmodel en niet in die van anderen. De reden is dat één bepaalde leefstijlgerelateerde aandoening met veel verschillende preventiemaatregelen te bestrijden is. Deze maatregelen kunnen rechtsreeks worden gericht op de persoon met het ongezonde gedrag, maar ook op omgevingsfactoren. In de overige modellen worden interventies beoordeeld waarvoor er weinig of geen alternatieven zijn. Omdat de effecten en neveneffecten van verschillende preventiemaatregelen ter bestrijding van leefstijlgerelateerde aandoeningen sterk uiteen kunnen lopen, en de verdeling van baten en lasten over betrokken partijen meer of minder rechtvaardig kan uitvallen, vind ik het de moeite waard om het criterium 'alternatieven' voor mijn model te behouden.

Ik ***schrap het criterium 'subsidiariteit'***, omdat dit criterium een waardeoordeel bevat (zie de bespreking van versie 3 van mijn model). Een dergelijk waardeoordeel

in een criterium vind ik bij nader inzien niet geschikt voor de waardeneutraliteit[9] van mijn model. Ik vervang het criterium 'subsidiariteit' door het criterium 'alternatieven' dat geen waardeoordeel bevat.

Op basis van de bovenstaande bespreking van rij I t/m X van overzicht 2 heb ik versie 5 van mijn beoordelingsmodel opgesteld (zie voor versie 5 het begin van §2.6.2). Overzicht 4 bevat een vergelijking van de criteria van versie 4 en 5 van mijn model.

Overzicht 4: vergelijking criteria van versie 4 en 5

**Criteria in versie 4**	**Corresponderende criteria in versie 5**
**Gerechtvaardigdheidsvraag (A)**	
1. Proportionaliteit	– Schadelijkheid<br>– Proportionaliteit
2. Doelmatigheid	– Kosteneffectiviteit
3. Toerekenbaarheid, aansprakelijkheid en strafbaarheid	– Verantwoordelijkheid<br>– Gerichtheid<br>– Rechtvaardigheid
4. Distributieve rechtvaardigheid	– Rechtvaardigheid (distributief en vergelijkend)
5. Acceptatie en handhaving	– Aanvaardbaarheid<br>– Uitvoerbaarheid
6. Frequentie en duur	– Uitvoerbaarheid
7. Alternatieven	– Alternatieven
**Verantwoordelijkheidsvraag (B)**	
1. Toepassingsbereik	– Gerichtheid
2. Bekwaamheid en bevoegdheid	– Verantwoordelijkheid
3. Geschiktheid	– Verantwoordelijkheid
4. Procedurele zorgvuldigheid	– Zorgvuldigheid
5. Subsidiariteit	– Alternatieven

Uit overzicht 4 blijkt dat alle criteria uit versie 4 van mijn beoordelingsmodel, voor een deel in aangepaste vorm, een plaats hebben gekregen in versie 5. Onderstaand bespreek ik enkele aspecten van versie 5, die in de bespreking van rij I t/m X van overzicht 2 nog niet aan de orde zijn gekomen.

9. Zie §15.1.2 voor een toelichting op (de spelling van) het begrip 'waardeneutraliteit'.

Geïnspireerd door de structuur van de Trechter van Dunning, waarbij ieder criterium een zeef in een trechter vormt, heb ik versie 5 van mijn model opgebouwd uit ***een drietal zeven (doelstelling, effecten en uitvoering)*** die ieder uit vier criteria bestaan. Op deze plaats ga ik niet verder op deze zeefstructuur in, omdat ik de structuur pas bespreek in het volgende hoofdstuk. Reden om op deze plaats toch kort iets over de structuur van versie 5 op te merken, is dat het criterium 'alternatieven' daar drie keer in voorkomt.

De reden om het criterium 'alternatieven' op drie plaatsen in mijn model op te nemen, is dat ik het verstandig acht om bij de beoordeling ook tussentijds alternatieven te overwegen en om daar niet mee te wachten totdat de gerechtvaardigdheid van de preventiemaatregel aan de hand van alle criteria is getoetst. Eigenlijk kan het criterium 'alternatieven' aan alle overige criteria worden gekoppeld. De bij deze koppeling behorende vraag is of een andere (alternatieve) vormgeving van de preventiemaatregel zou leiden tot een betere score op het betreffende criterium. Voor de overzichtelijkheid heb ik het criterium 'alternatieven' in versie 5 niet aan alle criteria afzonderlijk gekoppeld, maar aan de drie zeven (doelstelling, effecten en uitvoering) waaruit het model is opgebouwd.

In versie 4 van mijn model wordt onderscheid gemaakt tussen (A) een overkoepelende gerechtvaardigdheidsvraag en (B) een overkoepelende verantwoordelijkheidsvraag. In versie 4 betreft de overkoepelende verantwoordelijkheidsvraag de vraag 'wie de preventiemaatregel mogen uitvoeren' (*'wie-vraag'* uit versie 1 t/m 3 van het model). Doordat ik vanaf versie 5 het schadebeginsel kies als uitgangspunt voor de beoordeling van de ideologische (perfectionistische, moralistische en paternalistische) aspecten van de preventiemaatregel, krijgt de verantwoordelijkheidsvraag in deze versie een bredere betekenis. De verantwoordelijkheidsvraag betreft in versie 5 ook de vraag naar de verantwoordelijkheid voor het ontstaan en de instandhouding van het risico op schade. Het antwoord op deze vraag is medebepalend voor de beoordeling van de 'gerichtheid' en de (distributieve en vergelijkende) 'rechtvaardigheid' van de preventiemaatregel (criteria in versie 5). De overkoepelende gerechtvaardigdheidsvraag *('wat-vraag')* en verantwoordelijkheidsvraag *('wie-vraag')* uit versie 4 van mijn model, beginnen hierdoor door elkaar heen te lopen. Daarom laat ik dit onderscheid in versie 5 vallen.

2.7 Zesde versie van het beoordelingsmodel

2.7.1 Vergelijking van het model met andere beoordelingsmodellen

Versie 5 van mijn model is tot stand gekomen door de vergelijking van versie 4 met andere beoordelingsmodellen op het terrein van de volksgezondheid en gezondheidszorg (zie overzicht 2 in §2.6.1). Deze vergelijking heb ik gebruikt als ***'literatuurtoets'*** van de ***volledigheid en juistheid*** van de criteria van het tot dusver door mij ontwikkelde model. De andere modellen gaven ook goede handvatten voor de ***operationalisering*** van de criteria van het model. Deze operationalisering bespreek ik echter pas in de hoofdstukken over casuïstiek uit de praktijk (hoofdstuk 5 t/m 14). Naar aanleiding van de vergelijking met de andere modellen is het model ingrijpend gewijzigd, doordat aan versie 5 de criteria 'schadelijkheid' en 'gerichtheid' zijn toegevoegd en de structuur is aangepast.

Voordat ik tot toetsing van mijn model aan de praktijk ben overgegaan (zie hoofdstuk 5), heb ik eerst onderzocht of ik in de literatuur nog meer modellen op het terrein van volksgezondheid en gezondheidszorg zou kunnen vinden aan de hand waarvan ik de criteria van mijn model zou kunnen toetsen (en operationaliseren). Ik heb nog drie andere modellen gevonden, die bruikbaar zijn gebleken:

- een model over de gerechtvaardigdheid van public health interventies (Gostin, 2000);
- een model – de Preffi[10] – voor het effectief managen van gezondheidsbevordering (Molleman, 2003), (Molleman, 2005), (Molleman, 2006);
- een model van de Raad voor Volksgezondheid en Zorg voor pakketbeslissingen (RVZ, 2006, p.15-47).

Een minutieuze vergelijking van versie 5 van mijn model met de bovengenoemde modellen van Gostin, Molleman (Preffi) en de RVZ heeft geleid tot ***versie 6*** [11]**.**

Overzicht 5: verhouding tussen mijn model en bestaande beoordelingsmodellen

10. Preffi: Health Promotion Effect Management Instrument.
11. Versie 5 van mijn model is gebaseerd op een vergelijking van versie 4 met de modellen van de Gezondheidsraad, Wilson en Jungner, het CVZ en Dunning (zie §2.6).

	**Versie 6 van mijn model**	**Gostin**	**Preffi**	**Gezondheidsraad**	**Wilson en Jungner**	**RVZ**	**CVZ**	**Dunning**
	Gerechtvaardigdheid preventiemaatregelen gericht op leefstijl	Gerechtvaardigdheid public health interventies	Managen gezondheidsbevordering ter vergroting effectiviteit	Voorwaarden aan toepassing genetische screening	Aanvaardbaarheid screening	Pakketbeslissingen	Pakketbeslissingen	Pakketbeslissingen
I	Schadelijkheid (1)	Risico op schade (1)	Probleemanalyse (2): aard, ernst, omvang (2.1) en spreiding van probleem (2.2)	Gezondheidsprobleem (1)<br><br>Prevalentie (12a)<br><br>Natuurlijk beloop en variatie in ernst aandoening (12b)	Belangrijk gezondheidsprobleem (1)	Noodzakelijkheid (ziektelast) (1)	Noodzakelijkheid (rechtvaardigt de ziekte of benodigde zorg een claim op solidariteit, gegeven de culturele context) (1)	Noodzakelijkheid (1)
II	Noodzakelijkheid (2)		Probleemanalyse (2): probleemperceptie van betrokkenen (2.3)	Doel is deelnemers faciliteren (3)	Belangrijk gezondheidsprobleem (1)			
III	Oorzakelijkheid (3)		Determinanten van problematiek, gedrag en omgeving (3): theoretisch model (3.1) en factoranalyse (3.2)	Prevalentie (12a)<br><br>Natuurlijk beloop en variatie in ernst aandoening (12b)	Inzicht in beloop van ziekte (7)			

	Versie 6 van mijn model	Gostin	Preffi	Gezondheids-raad	Wilson en Jung-ner	RVZ	CVZ	Dunning
IV	Verantwoorde-lijk-heid (4)					Rechtvaardig-heid/ solidariteit (4)	(Noodzakelijk-heid) rechtvaar-digt de ziekte of benodigde zorg een claim op solidariteit; zo nee, dan zijn kosten voor eigen rekening) (1)	Eigen rekening en verantwoor-ding (4)
V	Gerichtheid (5)		Beïnvloedbaarheid van determinant (3.3)<br>Aangrijpingspunten voor interventie (3.4)<br>Keuze van doelgroep (4, 4.1)<br>Keuze van doelen (5): aansluiting op ana-lyse (5.1)<br>Interventieontwikke-ling (6): rationale (6.1), afstemming op aard doelgroep (6.3) en samenhang (6.6)	Doelgroep is duidelijk (2)<br><br>Het waarom van gekozen doel-groep en tijdstip in leven van test (12c)	Algemeen aan-vaard beleid over wie als patiënt wordt behandeld (8)<br><br>Passende test (5)			
VI	Geschiktheid (6)		Inbedden interventie in bestaande struc-tuur (7.3)					

	Versie 6 van mijn model	Gostin	Preffi	Gezondheidsraad	Wilson en Jungner	RVZ	CVZ	Dunning
VII	Effectiviteit (7)	Effectiviteit (2)	Duur, intensiteit en timing interventie (6.2)<br><br>Effectieve technieken (6.4)<br><br>Effectevaluatie (8.3)	Zinvolle handelingsopties na testuitslag (4, 12e)	Algemeen aanvaarde behandelwijze (2)<br><br>Herkenbaar latent stadium (4)	Effectiviteit/ werkzaamheid (2)	Effectiviteit (in de breedste zin) (2)	Werkzaamheid (2)
VIII	Indringendheid (8)	Lasten voor individu (indringendheid van interventie) (4)	Doelen zijn aanvaardbaar (5.3)	Bescherming privacy (9)<br><br>Belasting voor deelnemers (12d)	Aanvaardbaarheid voor bevolking (6)			
IX	Kosteneffectiviteit (9)	Kosteneffectiviteit (3)	Evaluatie (8): overeenstemming over uitgangspunten (8.1) en procesevaluatie (8.2)	Kosten van uitvoering (screening en infrastructuur) (12j)	Verhouding kosten bevolkingsonderzoek versus beschikbare middelen voor volksgezondheid (9)	Doelmatigheid/ kosteneffectiviteit (3)	Kosteneffectiviteit (verhouding kosten en baten in de breedste zin) (3)	Doelmatigheid (3)

	Versie 6 van mijn model	Gostin	Preffi	Gezondheidsraad	Wilson en Jungner	RVZ	CVZ	Dunning
X	Lasten-batenverhouding (10)	Rechtvaardigheid (fair distribution of benefits, burdens and costs) (5)		Meer voordelen dan nadelen voor deelnemers (12, 12g)<br><br>Kans op schade ten gevolge van (test)fouten (12h)<br><br>Informatie over nevendoelstellingen (10)				
XI	Rechtvaardigheid (11)			Voorkomen van ongerechtvaardigde gevolgen (12i)	Verhouding kosten bevolkingsonderzoek versus beschikbare middelen voor volksgezondheid (9)	Rechtvaardigheid/ solidariteit (4)		
XII	Rechtsgelijkheid (12)							

	Versie 6 van mijn model	Gostin	Preffi	Gezondheidsraad	Wilson en Jungner	RVZ	CVZ	Dunning
XIII	Draagvlak (13)		Randvoorwaarden en haalbaarheid (1): draagvlak (1.1)<br><br>Motivatie en mogelijkheden doelgroep (4.2)<br><br>Doelen zijn aanvaardbaar (5.3)<br><br>Feedback aan betrokkenen (8.4)	Informed consent (5)	Algemeen aanvaarde behandelwijze (2)<br><br>Aanvaardbaarheid voor bevolking (6)<br><br>Algemeen aanvaard beleid over wie als patiënt wordt behandeld (8)	Maatschappelijk draagvlak (5)		

	Versie 6 van mijn model	Gostin	Preffi	Gezondheids-raad	Wilson en Jungner	RVZ	CVZ	Dunning
XIV	Uitvoerbaarheid (14)		Randvoorwaarden en haalbaarheid (1): capaciteit (1.2) en bekwaamheid projectleider (1.3)<br><br>Bereikbaarheid van de doelgroep (4.3)<br><br>Doelen zijn specifiek, tijdgebonden, meetbaar (5.2) en haalbaar (5.4)<br><br>Haalbaarheid in de praktijk (6.5)<br><br>Pretest (6.7)<br><br>Implementatie (strategie) (7, 7.1)	Faciliteiten voor vervolgacties (8)<br><br>Kosten van uitvoering (screening en infrastructuur) (12j)<br><br>Voortdurende kwaliteitsbewaking (11)	Beschikbaarheid van voorzieningen voor diagnostiek en behandeling (3)<br><br>Continuïteit van bevolkingsonderzoek (10)		Uitvoerbaarheid (haalbaarheid en houdbaarheid) (4)	

	Versie 6 van mijn model	Gostin	Preffi	Gezondheids-raad	Wilson en Jungner	RVZ	CVZ	Dunning
XV	(Kwaliteits-bewaking en transparantie)		Monitoren en genereren van feedback (7.2)<br><br>Feedback aan betrokkenen (8.4)	Kwaliteit testmethode (12d)<br><br>Voortdurende kwaliteitsbewaking (11)<br><br>Begrijpelijke voorlichting (6)<br><br>Informatie over nevendoelstellingen (10)<br><br>Tijd voor overwegen behandelingsopties (12f)				

Toelichting op de totstandkoming van overzicht 5

In overzicht 5 worden de beoordelingscriteria van acht modellen met elkaar vergeleken; achtereenvolgens:

1. Versie 6 van mijn model;
2. Gostin (Gostin, 2000);
3. Preffi (Molleman, 2006);
4. Gezondheidsraad (zie overzicht 2);
5. Wilson en Jungner (zie overzicht 2);
6. RVZ (RVZ, 2006);
7. CVZ (zie overzicht 2);
8. Dunning (zie overzicht 2).

Per rij (I t/m XV) van overzicht 5 bestaat er een inhoudelijke samenhang tussen de criteria van de verschillende modellen. Overzicht 5 is als volgt van links naar rechts opgebouwd:

- Ik ben begonnen met het plaatsen van de veertien criteria van versie 6 van mijn model in de tweede kolom (rij I t/m XIV)[12].
- Vervolgens heb ik per kolom de criteria van de andere modellen aan overzicht 5 toegevoegd.
- Voor het criterium 'kwaliteitsbewaking en transparantie', dat niet in versie 6 van mijn model voorkomt, heb ik, ten behoeve van enkele criteria van de Preffi en van het model van de Gezondheidsraad, een extra rij (XV) geopend.
- Achter ieder criterium heb ik tussen haakjes het volgordenummer geplaatst dat het criterium heeft in het oorspronkelijke model:
  - Versie 6 van mijn model: criteria 1 t/m 14;
  - Gostin: criteria 1 t/m 5;
  - Preffi: criteria 1 t/m 8 (1.1 t/m 1.3, 2.1 t/m 2.3, 3.1 t/m 3.4, 4.1 t/m 4.3, 5.1 t/m 5.4, 6.1 t/m 6.7, 7.1 t/m 7.3 en 8.1 t/m 8.4);
  - Gezondheidsraad criteria 1 t/m 12 en 12a t/m 12j;
  - Wilson en Jungner: criteria 1 t/m 10;
  - RVZ: criteria 1 t/m 5;
  - CVZ: criteria 1 t/m 4;
  - Dunning: criteria 1 t/m 4.

12. In 2.1 heb ik toegelicht waarom ik de bespreking van de totstandkoming van een nieuwe versie van het model soms begin met het presenteren van de nieuwe versie van het model in plaats van dat ik de bespreking hiermee eindig.

Toelichting op de 'nieuwe' modellen (Gostin, Preffi en RVZ)

Het model van Gostin is belangrijk, omdat de doelstelling van dit model 'toetsing van de gerechtvaardigdheid van public health interventies' inhoudelijk het dichtst bij de doelstelling van het model ligt. Dit kan verklaren waarom het model van Gostin, evenals versie 6 van mijn model, het begrip 'schade' heeft verwerkt in het eerste criterium.

De criteria van de Preffi hebben onderling de nodige overlap, die ik binnen mijn model juist tracht te vermijden. Deze overlap komt de overzichtelijkheid niet ten goede. Toch vind ik de Preffi een belangrijke aanwinst als vergelijkingsmateriaal met mijn model, met name vanwege de aandacht die binnen de Preffi wordt besteed aan de 'rationale' (logica) van de interventieontwikkeling en omdat het model wetenschappelijk is gevalideerd (Molleman, 2005, 2006). De overlap tussen criteria binnen de Preffi kan worden verklaard doordat het geen toetsingsmodel is, maar een managementinstrument.

De criteria van het model van de RVZ hebben veel overlap met de criteria van het model van het CVZ en Dunning. Toch vormt het model van de RVZ een aanvulling op deze modellen, omdat het gebaseerd is op een ***internationaal vergelijkende studie***, waarbij de onderzoekers hebben geconcludeerd dat er ***internationaal consensus*** blijkt te bestaan over de te hanteren criteria (Ottes, 2006, p32). Bovendien geeft de RVZ een uitgebreide beschrijving van het criterium 'draagvlak' (RVZ, 2006, p24-29, 37-38, p.45-47).

Versie 6 van mijn beoordelingsmodel (2008)

Logica van het ontwerp

1. Schadelijkheid (van leefstijl)
2. Noodzakelijkheid (van schadepreventie)
3. Oorzakelijkheid (van risico op schade)
4. Verantwoordelijkheid (voor risico en risicopreventie)
5. Gerichtheid (op doelgroep of doelfactoren in omgeving)
6. Geschiktheid (van uitvoerende partij)

Effecten en neveneffecten

7. Effectiviteit
8. Indringendheid
9. Kosteneffectiviteit
10. Lasten-batenverhouding
11. Rechtvaardigheid

12. Rechtsgelijkheid

Implementatie
13. Draagvlak
14. Uitvoerbaarheid

2.7.2 Wijzigingen in versie 6 t.o.v. versie 5 van mijn model

In deze subparagraaf bespreek ik de wijzigingen in versie 6 t.o.v. versie 5 van mijn model (zie overzicht 6).

Overzicht 6: vergelijking van versie 5 en 6

**Versie 5**	**Versie 6**
**Doelstelling**	**Logica van ontwerp**
1. Schadelijkheid (van leefstijl)	1. Schadelijkheid
	2. Noodzakelijkheid
	3. Oorzakelijkheid
2. Verantwoordelijkheid	4. Verantwoordelijkheid
3. Gerichtheid	5. Gerichtheid
	6. Geschiktheid
4. Alternatieven	
**Effecten**	**Effecten en neveneffecten**
	7. Effectiviteit
	8. Indringendheid
1. Kosteneffectiviteit	9. Kosteneffectiviteit
2. Proportionaliteit	10. Lasten-batenverhouding
3. Rechtvaardigheid	11. Rechtvaardigheid
	12. Rechtsgelijkheid
4. Alternatieven	
**Uitvoering**	**Implementatie**
1. Aanvaardbaarheid	13. Draagvlak
2. Uitvoerbaarheid	14. Uitvoerbaarheid
3. Zorgvuldigheid	(6. Geschiktheid)
4. Alternatieven	

Criteria 1 t/m 4 van versie 6
Aan het 1e criterium 'schadelijkheid' en 2e criterium 'verantwoordelijkheid' van versie 5 van mijn model kleven aspecten die ***empirisch*** kunnen worden onderzocht en aspecten die ***ideologisch*** worden bepaald. Met het oog op de rationaliteit van mijn model tracht ik deze aspecten uit elkaar te trekken. In versie 6 van mijn model splits ik daartoe het criterium 'schadelijkheid' op in een criterium ***'schadelijkheid'*** (dat empirisch kan worden onderzocht) en een criterium ***'noodzakelijkheid'*** (dat ideologisch wordt bepaald). Tevens splits ik daartoe het criterium 'verantwoordelijkheid' op in een criterium ***'oorzakelijkheid'*** (dat empirisch kan worden onderzocht) en een criterium ***'verantwoordelijkheid'*** (dat ideologisch wordt bepaald). Het belang van het onderscheid tussen beoordeling op basis van empirische informatie en ideologische opvattingen voor de rationaliteit van mijn model werk ik uit in het volgende hoofdstuk (§3.3).

In versie 6 van mijn model betreft het criterium 'schadelijkheid' de aard en omvang van de risico's op schade, op overlast en op schending van morele opvattingen, die men met de preventiemaatregel tracht te bestrijden. Het criterium 'noodzakelijkheid' betreft de noodzaak om deze risico's met preventiemaatregelen te bestrijden. Het criterium 'oorzakelijkheid' betreft het aandeel van verschillende determinanten in het ontstaan en de instandhouding van deze risico's. Het criterium 'verantwoordelijkheid' betreft de vraag in welke mate verschillende partijen voor het ontstaan en de instandhouding van deze risico's verantwoordelijk kunnen worden gehouden. Als kinderen bijvoorbeeld te dik worden doordat zij zelf teveel snoepen, kunnen misschien toch hun ouders hiervoor (deels) verantwoordelijk worden gehouden.

Criteria 5 en 6 van versie 6
In versie 6 van mijn model heeft het criterium '***gerichtheid'*** dezelfde betekenis als in versie 5. Het gaat om inzicht in de vraag of de preventiemaatregel wordt gericht op de juiste doelgroep of omgevingsfactoren (zie de bespreking van rij V van overzicht 5).

Het criterium 'geschiktheid' uit versie 3 en 4 van mijn model is in versie 5 onderdeel geworden van het criterium 'verantwoordelijkheid'. In versie 6 besluit ik het ***criterium 'geschiktheid'*** weer als afzonderlijk criterium te benoemen. De reden is dat de 'geschiktheid' niet beoordeeld kan worden voordat de 'gerichtheid' (5e criterium in versie 6 van het model) van de preventiemaatregel bekend is. Bij de beoordeling van de gerechtvaardigdheid van de preventiemaatregel aan de hand van het criterium 'geschiktheid' worden in versie 6 de volgende aspecten beoordeeld:

- Is de actor die de preventiemaatregel gaat uitvoeren daartoe wel bekwaam en bevoegd? (Mogen werkgevers hun personeel bijvoorbeeld verbieden om in hun vrije tijd te roken?);
- Is de actor wel geschikt voor de preventiemaatregel en is de preventiemaatregel wel geschikt voor de actor? (Is bijvoorbeeld de tabaksindustrie, die wel ‘bekwaam en bevoegd’ is om het roken onder jongeren terug te dringen, daarvoor wel geschikt?) (Is bijvoorbeeld de bestrijding van overgewicht wel een ‘geschikte’ taak voor scholen? Leidt dit niet tot een ongewenste medicalisering van het onderwijs?).

Criteria 7 en 8 van versie 6

Een begeleider van mijn promotieonderzoek dringt er op aan om de ‘indringendheid’ van de preventiemaatregel als afzonderlijk criterium ‘voorin’ mijn model op te nemen. Het ***criterium ‘indringendheid’*** betreft het binnendringen in het persoonlijke leven van mensen door toepassing van de preventiemaatregel. Omdat er bij preventiemaatregelen die trachten de leefstijl van mensen gezonder te maken lang niet altijd sprake is van ‘informed consent’ – de maatregelen kunnen zelfs tegen de wil van (een deel van) de doelgroep worden genomen – kan de indringendheid zeer ingrijpend zijn. Het criterium ‘indringendheid’ verdient daarom een zelfstandige, prominente (aan het begin van de rij criteria betreffende de effecten en neveneffecten) en zichtbare plaats binnen het model. Daarom moet het niet worden ‘verstopt’ in het criterium ‘lasten-batenverhouding’ dat pas volgt na het criterium ‘kosteneffectiviteit’. Omdat binnendringing van een preventiemaatregel in het persoonlijk leven van mensen zo ingrijpend kan zijn, heb ik besloten het criterium ‘indringendheid’ direct na het criterium ‘effectiviteit’ in het model op te nemen.

Het criterium ‘indringendheid’ kan breed en smal worden gedefinieerd:

- Heel breed gedefinieerd betreft de ‘indringendheid’ alle schade in de zin van het schadebeginsel, <u>en</u> overlast, <u>en</u> schending van morele opvattingen. Schade in de zin van het schadebeginsel is door mij geoperationaliseerd als gezondheidsschade, sociaal-economische schade, en schending van individuele mensenrechten (zie bijlage 5).
- Breed gedefinieerd betreft de ‘indringendheid’ alle schade in de zin van het schadebeginsel, exclusief overlast en exclusief schending van morele opvattingen.
- Smal gedefinieerd betreft de ‘indringendheid’ schending van individuele mensenrechten door de preventiemaatregel (aantasting van de lichamelijke integriteit, van de vrijheid, van de privacy, van de gelijke behandeling, van de eer en goede naam, van de waardigheid en respectvolle bejegening).

Het bijzondere van preventiemaatregelen ter bestrijding van leefstijlgerelateerde aandoeningen, is dat zij trachten de leefstijl van bepaalde mensen te beïnvloeden en daardoor een ongerechtvaardigde bemoeienis met het leven van mensen kunnen vormen. Een niet te rechtvaardigen bemoeienis met het leven van mensen betekent een schending van hun individuele mensenrechten. Daarom kies ik voor de smalle definitie van het begrip 'indringendheid': schending van de individuele mensenrechten.

Door bezig te zijn met het criterium 'indringendheid' realiseerde ik me dat leefstijlgerelateerde aandoeningen ook kunnen worden bestreden zonder leefstijlen te beïnvloeden. Teveel en ongezond eten en te weinig bewegen kunnen leiden tot een te hoge bloeddruk en een te hoog cholesterolgehalte. In plaats van gezonder te gaan eten en/of meer te gaan bewegen, kunnen op basis van informed consent bloeddrukverlagers en cholesterolremmers worden geslikt. Dit is niet het soort preventiemaatregelen ter bestrijding van leefstijlgerelateerde aandoeningen dat ik voor ogen had toen ik mijn onderzoeksvraag formuleerde. Mijn bedoeling was om een model te ontwerpen voor de beoordeling van de gerechtvaardigdheid van preventiemaatregelen die trachten de leefstijl van mensen te beïnvloeden (Scholtmeijer, 2006, p.222). Op grond van deze overwegingen besluit ik om mijn onderzoeksvraag nog preciezer te formuleren. Hieronder worden de oude (3e versie) en nieuwe formulering (4e versie) van mijn onderzoeksvraag weergegeven.

Wijziging van onderzoeksvraag

Oude formulering van de onderzoeksvraag (3e versie, 2006)
Kan een model worden ontwikkeld aan de hand waarvan de gerechtvaardigdheid van preventiemaatregelen ter bestrijding van leefstijlgerelateerde aandoeningen kan worden beoordeeld?

Nieuwe formulering van de onderzoeksvraag (4e versie, 2008)
Kan een model worden ontwikkeld aan de hand waarvan de gerechtvaardigdheid van preventiemaatregelen kan worden beoordeeld, die ter bestrijding van leefstijlgerelateerde aandoeningen ***trachten de leefstijl van mensen te beïnvloeden***?

Mijn onderzoek richt zich dus op de beoordeling van de gerechtvaardigdheid van ***leefstijlbeïnvloedende interventies***, zoals in het kader gedefinieerd. Ik maak onderscheid tussen leefstijlbeïnvloedende interventies en leefstijlinterventies:

- ***leefstijlinterventies*** zijn interventies die ***rechtstreeks*** in de leefstijl van mensen interveniëren en vormen een deelverzameling van leefstijlbeïnvloedende interventies;
- ***leefstijlbeïnvloedende interventies*** kunnen ***ook indirect*** de leefstijl van mensen beïnvloeden (bijvoorbeeld door te interveniëren in de media en door de reclame voor genotsmiddelen aan banden te leggen).

Preventiemaatregelen ter bestrijding van leefstijlgerelateerde aandoeningen, die de leefstijl van mensen in principe ongemoeid laten (cholesterolremmers, antibiotica tegen geslachtsziekten, e.d.), vragen om andere accenten in de keuze en operationalisering van beoordelingscriteria, en vallen buiten het kader van mijn onderzoek.

Het criterium ‘indringendheid’ betreft een deel van de neveneffecten van leefstijlbeïnvloedende interventies. Om te beoordelen of de indringendheid aanvaardbaar is, dient deze te worden gewogen tegen de effecten van de preventiemaatregel. Vergelijk dit met het criterium kosteneffectiviteit, waarbij de kosten worden gewogen tegen de effecten. Daarom kies ik er voor om het criterium ‘effectiviteit’ als afzonderlijk criterium, voorafgaand aan het criterium ‘indringendheid’, in mijn model op te nemen. De ***effectiviteit*** wordt hierdoor het ***7e criterium*** en de ***indringendheid*** het ***8e criterium*** van het model. Het afzonderlijk beoordelen van de effectiviteit levert geen meerwerk op, want de effectiviteit dient ook bekend te zijn voor het beoordelen van de kosteneffectiviteit en de lasten-batenverhouding.

Criteria 9 en 10 van versie 6

N.a.v. de introductie van de afzonderlijke criteria ‘effectiviteit’ en ‘indringendheid’ in mijn model heb ik overwogen om het criterium ‘kosteneffectiviteit te laten vervallen. De opeenstapeling van criteria die betrekking hebben op de (neven)effecten van de preventiemaatregel – effectiviteit, indringendheid, kosteneffectiviteit, lasten-batenverhouding – leek mij wat teveel van het goede. In geval van vrijheidsbeperkende preventiemaatregelen is er vaak sprake van wettelijke maatregelen (denk bijvoorbeeld aan het wettelijk rookverbod in de Nederlandse Horeca) en bij het beoordelen van de gerechtvaardigdheid van dergelijke maatregelen lijkt de kosteneffectiviteit een ondergeschikte rol te spelen. Bovendien maakt de kosteneffectiviteit, evenals de effectiviteit en indringendheid, sowieso onderdeel uit van de lastenbatenverhouding. Uiteindelijk heb ik besloten het criterium ‘kosteneffectiviteit’ toch te handhaven in versie 6 van het model. Bij de toetsing van het model aan de empirie wordt de relevantie van een criterium ‘kosteneffectiviteit’ beter zichtbaar als expliciet aan dit criterium wordt getoetst dan wanneer dit criterium alleen impliciet in de lasten-batenverhouding aanwezig is. Dit brengt mij op de gedachte, dat ik bij twijfel over het al dan niet samenvoegen van twee criteria tot één crite-

rium, ik deze criteria voorafgaand aan de toetsing aan de empirie beter (nog) niet kan samenvoegen.

Criteria 11 en 12 van versie 6
De criteria 'rechtvaardigheid' en 'rechtsgelijkheid' betreffen respectievelijk de distributieve en vergelijkende rechtvaardigheid. De betekenis van dit onderscheid is reeds toegelicht bij de bespreking van rij IX van overzicht 2 in §2.6.2. Ik twijfelde of ik deze criteria samengevoegd zou houden in een criterium 'rechtvaardigheid' (zoals in versie 5 van mijn model), of dat ik deze criteria toch afzonderlijk in het model zou opnemen. Omdat ik hierover twijfelde, heb ik besloten ze voorafgaand aan de toetsing aan de empirie als afzonderlijke criteria in versie 6 van het model op te nemen.

Criteria 13 en 14 van versie 6
Ik vervang het criterium 'aanvaardbaarheid' uit versie 5 van mijn model door het criterium 'draagvlak' in versie 6. De Preffi koppelt de term 'aanvaardbaarheid' aan de doelen van de preventiemaatregel (punt 5.3 van de Preffi). De aanvaardbaarheid betreft echter niet alleen de doelen (beoogde effecten), maar zeker ook de neveneffecten (indringendheid) van de preventiemaatregel. Wilson en Jungner spreken over de 'aanvaardbaarheid voor de bevolking'. Vanuit theoretisch perspectief vind ik het onduidelijk hoeveel aanvaardbaarheid c.q. aanvaarding van de preventiemaatregel nodig is om de maatregel te rechtvaardigen. De term 'maatschappelijk draagvlak' wordt gebruikt in het model van de RVZ (zie overzicht 4). Vanuit theoretisch perspectief vind ik het wel duidelijk hoeveel maatschappelijk draagvlak er nodig is om de preventiemaatregel te rechtvaardigen. Er dient zoveel maatschappelijk draagvlak te zijn, dat de maatregel succesvol kan worden ingevoerd en gehandhaafd. Soms kan voor de implementatie van de preventiemaatregel een ander draagvlak worden gebruikt dan een '*maatschappelijk* draagvlak'. Een geringer *maatschappelijk* draagvlak kan in theorie worden gecompenseerd door een groter draagvlak bij actoren die verantwoordelijk zijn voor de handhaving van de maatregel (bijvoorbeeld een breed draagvlak voor een verbod op openbaar alcoholgebruik bij de politie die dit verbod moet handhaven). Ik noem het criterium daarom sec 'draagvlak'.

Criteria 'zorgvuldigheid' en 'alternatieven' van versie 5
Het criterium 'zorgvuldigheid' betreft niet alleen de 'uitvoering' van de preventiemaatregel (zie versie 5 van mijn model in de linker kolom van overzicht 6), maar zowel de 'implementatie' als het 'ontwerp' van de preventiemaatregel (zie versie 6 in de rechter kolom van overzicht 6). Daarom besluit ik om het criterium 'zorgvuldigheid' niet als criterium in mijn model op te nemen, maar als aspect van de

beoordeling aan de hand van de criteria betreffende het ontwerp en de implementatie van de preventiemaatregel. Ook het criterium 'alternatieven' is op veel verschillende criteria van toepassing. Ik heb dit in versie 5 van mijn model tot uitdrukking gebracht door het criterium 'alternatieven' driemaal in dit model op te nemen. Ik besluit nu om het criterium 'alternatieven' als criterium te schrappen, omdat eigenlijk bij ieder criterium moet worden overwogen of er geen betere alternatieven zijn.

Zeefstructuur van versie 6

Geïnspireerd door de structuur van de Trechter van Dunning, waarbij ieder criterium een zeef in een trechter vormt, heb ik versie 5 van mijn model opgebouwd uit een drietal zeven[13]:

1. Doelstelling
2. Effecten
3. Uitvoering.

In versie 6 van mijn model heb ik de benaming van de zeven veranderd:

1. Logica van ontwerp
2. Effecten en neveneffecten
3. Implementatie.

De benaming 'doelstelling' uit versie 5 van mijn model vind ik, als overkoepelende term voor de eerste zes criteria van het model, bij nader inzien te smal. Met name het criterium 'geschiktheid' betreft meer het 'ontwerp' dan de 'doelstelling' van de preventiemaatregel. De eerste zes criteria van het model betreffen de 'logica van het ontwerp' van de preventiemaatregel (in §3.1 werk ik dit uit). De 'doelstelling van de preventiemaatregel' maakt onderdeel uit van 'de logica van het ontwerp van de preventiemaatregel'. Daarom vervang ik de overkoepelende term 'doelstelling' uit versie 5 van mijn model, in versie 6 door de overkoepelende term 'logica van het ontwerp'.

De benaming 'effecten' uit versie 5 van mijn model brengt onvoldoende tot uitdrukking dat het bij de beoordeling van de gerechtvaardigdheid van preventiemaatregelen ter bestrijding van leefstijlgerelateerde aandoeningen om de omvang en verdeling van effecten én neveneffecten gaat. Daarom noem ik de tweede zeef in versie 6 'effecten en neveneffecten'.

13. In hoofdstuk 3 ga ik uitgebreid in op de zeefstructuur van het model. In hoofdstuk 2 heb ik mij gericht op de criteria van het model.

Ook de benaming ‘uitvoering’ uit versie 5 van mijn model vind ik, als overkoepelende term voor de laatste twee criteria van het model (‘draagvlak’ en ‘uitvoerbaarheid’), bij nader inzien te smal. Het criterium ‘draagvlak’ betreft een randvoorwaarde voor de uitvoering en niet de ‘uitvoering’ zelf. Daarom vervang ik de overkoepelende term ‘uitvoering’ uit versie 5 door de overkoepelende term ‘implementatie’ in versie 6. De term ‘implementatie’ betreft zowel het benodigde ‘draagvlak’ voor uitvoering van de preventiemaatregel als de ‘uitvoerbaarheid’ (haalbaarheid en houdbaarheid) van de preventiemaatregel.

Empirische toetsing van versie 6 van mijn beoordelingsmodel

Versie 6 van mijn model heb ik gebruikt voor de toetsing van het model aan casus 1 (Clarian Health) in de hoofdstukken 5 t/m 9.

3 Structuur van het beoordelingsmodel (versie 6 van het model)

3.1 Volgorde en werking van de beoordelingszeven

Het idee voor de structuur van mijn model heb ik ontleend aan het model van Dunning. Het model van Dunning wordt gevormd door een trechter met een viertal zeven, waarbij ieder criterium een zeef vormt (versie 6 van het model in overzicht 7 bestaat uit een drietal zeven). De vier criteria van de 'trechter van Dunning' hoeven niet tegen elkaar te worden gewogen, maar kunnen achtereenvolgens worden beoordeeld en 'afgevinkt'. Hiermee worden vergelijkingsproblemen tussen de criteria omzeild. De modellen van het CVZ en de RVZ zijn afgeleid van het model van Dunning, maar hebben het idee van de 'trechter met zeven' bewust losgelaten (RVZ, 2006, p.35-36):

> *"De Commissie Dunning koos voor een trapsgewijs systeem: een behandeling moet én noodzakelijk én werkzaam én doelmatig zijn én niet voor eigen rekening en verantwoording zijn. Zij ging hierbij uit van een sequentiële toepassing van de criteria en voor een 'zwart-wit' – ja/nee – beoordeling per criterium: een behandeling is wel of niet noodzakelijk, wel of niet werkzaam etc. De werkelijkheid is evenwel niet zwart-wit: een behandeling kan meer of minder noodzakelijk, effectief etc. zijn. Bij een ja/nee aanpak is het niet mogelijk een zeer effectieve behandeling van een minder ernstige aandoening af te wegen tegen een matig effectieve behandeling van een ernstige aandoening. Beslissingen over behandelingen die duidelijk effectief en goedkoop of ineffectief en duur zijn, zijn gemakkelijk. Het probleem vormt het 'grijze gebied' daartussen. Helaas liggen veel, zo niet de meeste behandelingen in dit grijze gebied."*

Het CVZ en de RVZ zijn van mening dat de criteria binnen hun eigen model tegen elkaar moeten kunnen worden gewogen (Mastenbroek, 2006, p.30) (RVZ, 2006, p.22, 35-36). Als op één criterium laag wordt gescoord en op de andere criteria hoog, moet dat laatste de doorslag kunnen geven. Dit ben ik met het CVZ en de RVZ eens. Toch grijp ik terug op het principe van een 'trechter met zeven' van Dunning. De zeefstructuur van mijn model vormt een compromis tussen enerzijds het model van Dunning, waarbij ieder criterium een zeef vormt, en anderzijds de modellen van CVZ en RVZ, waarbij alle criteria tegen elkaar gewogen kunnen worden. In mijn model kunnen de criteria binnen een zeef tegen elkaar worden gewogen, maar is er wel sprake van een "zwart-wit - ja/nee- beoordeling" per zeef. Als de 'logica van het ontwerp' (1e zeef) van de preventiemaatregel niet deugt, is

het niet zinvol om de ‘effecten en neveneffecten’ (2e zeef) nader te onderzoeken. Als het saldo van effecten en neveneffecten negatief is, of de verdeling van effecten en neveneffecten onrechtvaardig is, is het niet zinvol om de ‘implementatie’ (3e zeef) verder te onderzoeken.

De eerste reden om toch gebruik te maken van het principe van een trechter met opeenvolgende zeven, is dat de volgorde waarin de beoordelingscriteria worden gehanteerd binnen het model van grote invloed is op de inhoud en daarmee op de validiteit van het beoordelingsproces. Door de clustering van de beoordelingscriteria in zeven wordt inzichtelijker waarom de volgorde van de beoordelingscriteria essentieel is. De tweede reden is dat, door gebruik te maken van het principe van een trechter met zeven die achtereenvolgens gepasseerd moeten worden, het aantal van veertien criteria beter hanteerbaar wordt. Als een bepaalde zeef niet wordt gepasseerd, hoeven de daaropvolgende criteria immers niet meer te worden onderzocht. Overzicht 7 toont de zeefstructuur van versie 6 van het beoordelingsmodel.

Overzicht 7: zeefstructuur van versie 6 van het beoordelingsmodel

preventiemaatregel

↓

Logica van ontwerp (1e zeef)
(criteria 1 t/m 6)

Effecten en neveneffecten (2e zeef)
(criteria 7 t/m 12)

Implementatie (3e zeef)
(criteria 13 en 14)

↓

Gerechtvaardigde preventiemaatregel

De beoordelingscriteria die samen een beoordelingszeef vormen (zie versie 6 van het model in §2.7), hebben een inhoudelijke samenhang. De betekenis en volgorde van de beoordelingszeven zal ik hieronder toelichten.

De ***eerste zeef*** betreft de logische redenering[1,2] achter het ontwerp van de preventiemaatregel. Voordat in het kader van de tweede en derde zeef de gevolgen van de invoering van de preventiemaatregel worden beoordeeld, dient eerst vast te staan dat de logische redenering achter de preventiemaatregel transparant en juist is (HM Treasury, 2003, p.11). Ik zal dit illustreren aan de hand van een voorbeeld.

> Stel dat een Gemeentelijke Gezondheidsdienst (GGD) overweegt om aan alle schoolkinderen met overgewicht een intensief trainingsprogramma om af te vallen aan te bieden. Voordat de effectiviteit en indringendheid (7e en 8e criterium) worden beoordeeld, moet eerst worden nagedacht of het wel een juiste keuze is om een dergelijk programma aan de betreffende kinderen aan te bieden:
> - Is het overgewicht van de betreffende kinderen wel zo schadelijk (1e criterium) dat het noodzakelijk (2e criterium) is dit met een preventiemaatregel te bestrijden? Of is hier sprake van een perfectionistisch streven van de GGD naar een ‘ideaalgewicht’ voor iedereen?
> - Is het wel terecht dat de preventiemaatregel op de kinderen wordt gericht (5e criterium)? Zijn de ouders van deze kinderen niet veel meer bepalend (oorzakelijkheid: 3e criterium) en verantwoordelijk (4e criterium) voor het overgewicht van hun kinderen dan de kinderen zelf?
> - Of kunnen de ouders niet verantwoordelijk (4e criterium) worden gehouden voor het overgewicht, omdat de voedingsmiddelenindustrie hun kinderen verleidt tot overconsumptie (oorzakelijkheid: 3e criterium)?
> - Zou de preventiemaatregel daarom niet beter op de marketingactiviteiten van de voedingsmiddelenindustrie kunnen worden gericht (5e criterium)?
> - Of is een GGD geen geschikte (geschiktheid: 6e criterium) partij om de marketingactiviteiten van deze industrie te beïnvloeden, omdat hun hiertoe de kennis en kunde ontbreekt en de industrie buiten hun invloedssfeer ligt?

De ***tweede zeef*** (effecten en neveneffecten) vormt de lakmoesproef voor de logica van het ontwerp van de preventiemaatregel. De effectiviteit, indringendheid en kosteneffectiviteit maken alle drie onderdeel uit van de lasten-batenverhouding. Het uitvoeren van een volledige lasten-batenanalyse is veel werk. Daarom is het verstandig de lasten-batenanalyse stap voor stap op te bouwen, zodat geen onnodig werk wordt verricht:

- stap 1: effectiviteit (7e criterium)
- stap 2: indringendheid (8e criterium)

1. Engels: ‘rationale’ (HM Treasury, 2003, p.11).
2. De “Preffi 2.0” spreekt over de ‘probleemanalyse’ (Molleman, 2003, p.16).

– stap 3: kosteneffectiviteit (9e criterium)
– stap 4: lasten-batenverhouding (10e criterium).

Als de maatregel evident onvoldoende effectief (stap 1) is, is het zetten van de stappen 2 t/m 4 niet meer nodig. Als de verhouding tussen de effectiviteit en indringendheid (stap 1 en 2) ongunstig uitvalt, is het zetten van de stappen 3 en 4 niet meer nodig. Als de kosteneffectiviteit (stap 3) ongunstig uitvalt, is het zetten van stap 4 niet meer nodig.

Als de effectiviteit, indringendheid, kosteneffectiviteit, of lasten-batenverhouding tegenvallen, dient het ontwerp (1e zeef) van de preventiemaatregel te worden heroverwogen. Het naar aanleiding van de baten en lasten heroverwegen van de oorspronkelijke uitgangspunten op zoek naar betere alternatieven, is geen zwaktebod, maar een regulier onderdeel van rationele beleidsontwikkeling. Om tunnelvisie te voorkomen, verdient het zelfs aanbeveling om altijd ook de baten en lasten van preventiemaatregelen met een alternatief ontwerp ter bestrijding van dezelfde leefstijlgerelateerde aandoening te onderzoeken (HM Treasury, 2003, p.5), (Molleman, 2003, p.39). Het beoordelen van de rechtvaardige verdeling van baten en lasten (11e en 12e criterium) is niet zinvol als de lasten-batenverhouding (10e criterium) nog niet is beoordeeld.

Bij de beoordeling van de rechtvaardige verdeling van baten en lasten (11e en 12e criterium) dient goed te worden gekeken naar de 'verantwoordelijkheid' voor de risico's die men met de preventiemaatregel tracht te bestrijden (4e criterium). Zijn de mensen of organisaties die met de lasten (en baten) van de preventiemaatregel worden geconfronteerd al dan niet verantwoordelijk voor de risico's op schade die men tracht te bestrijden. Als de lasten-batenverhouding (10e criterium) gunstig uitvalt, maar de verdeling van de baten en lasten (11e en 12e criterium) onrechtvaardig uitvalt, kan worden overwogen om benadeelde partijen (financieel) te compenseren, in plaats van het ontwerp van de preventiemaatregel aan te passen.

De overgang van de tweede naar de ***derde zeef*** betreft de overgang van de theorie naar de praktijk. Als de preventiemaatregel de eerste en tweede zeef is gepasseerd, is de preventiemaatregel in theorie rechtvaardig bevonden. Dan resteert nog één vraag: is de praktijk gereed voor implementatie van de maatregel? Het gaat hierbij om de kans van slagen voor implementatie van de maatregel, zoals deze bedoeld is. Sluit de preventiemaatregel aan op de bestaande praktijk? Bestaat er voldoende (maatschappelijk) draagvlak (13e criterium) voor de maatregel, zodat de uitvoering niet op (onoverkomelijke) tegenwerking stuit? Zijn er voldoende mensen en middelen beschikbaar om de maatregel adequaat uit te voeren (14^e criterium)?

3.2 Rol van denkkaders bij de beoordeling

Diverse auteurs wijzen erop dat de denkkaders van de bij de bestrijding van overgewicht betrokken partijen in de praktijk veelal van grotere invloed zijn op de keuze voor het al dan niet uitvoeren van bepaalde preventiemaatregelen dan de beschikbare wetenschappelijk kennis over de oorzaken van overgewicht en de kosteneffectiviteit van mogelijke maatregelen (Brownell, 2005, p.959-960) (Abigail, 2005) (Kersh, 2005, p.839-849) (Guerrieri, 2005, p.645) (Schwartz, 2007, p.79 en 81) (Barry, 2009, p.7-8 en 18-22). Ter illustratie worden in bijlage 6 voorbeelden van tegengestelde denkkaders beschreven die de beoordeling van de gerechtvaardigdheid van een preventiemaatregel in grote mate kunnen beïnvloeden.

Brownell acht het voor de bestrijding van overgewicht contraproductief dat de oorzaken van obesitas onderwerp zijn van een verhit moreel en politiek debat. Volgens haar dienen de oorzaken van obesitas op basis van wetenschappelijk onderzoek te worden vastgesteld (Brownell, 2005, p.960). In overeenstemming met deze stelling van Brownell dienen het 1e en 3e criterium van het model ertoe om respectievelijk het risico op en de oorzaken van gezondheidsschade wetenschappelijk te bepalen. De mogelijkheden om de gerechtvaardigdheid van preventiemaatregelen ter bestrijding van overgewicht uitsluitend op basis van wetenschappelijk onderzoek te beoordelen, moeten echter niet worden overschat (House of Lords, October 2006, p.4).

De (gedeeltelijke) onjuistheid of juistheid van een deel van de in bijlage 6 beschreven denkkaders kan met empirisch wetenschappelijk onderzoek worden aangetoond[3]. Welke van de genoemde sociaal-culturele denkkaders juist is (a. mensen eten en drinken vooral thuis; b. mensen eten en drinken vooral buitenshuis) lijkt bijvoorbeeld goed empirisch te onderzoeken. In het kader van mijn beoordelingsmodel mogen de denkkaders die worden gehanteerd ter rechtvaardiging van de preventiemaatregel niet strijdig zijn met de beschikbare empirische kennis. Empirische kennis gaat in mijn model boven niet op de empirie gestoelde opvattingen. In §3.3 werk ik dit nader uit.

3. Vaak is het eenvoudiger om de onjuistheid dan de juistheid van een bepaalde opvatting aan te tonen; meestal is het eenvoudiger om een theorie te ***falsificeren*** dan te bewijzen. (Chalmers, 1976, p.58-91)

3.3 Fasering van de argumentatie [4]

Het doel van mijn beoordelingsmodel is het rationaliseren en transparant maken van de beoordeling van de gerechtvaardigdheid van preventiemaatregelen. De achterliggende doelen die ik hiermee wil bereiken, zijn het verbeteren van de kwaliteit en toetsbaarheid van het beoordelingsproces en van de uitkomsten van de beoordeling. Bij het rationaliseren van het beoordelingsproces stuit ik op het probleem dat het begrip 'gerechtvaardigdheid', dat de kern vormt van mijn onderzoeksvraag, een construct (complex, onstoffelijk voortbrengsel van de menselijke geest) is en geen stoffelijk verschijnsel waarvan de eigenschappen via natuurwetenschappelijk onderzoek eenduidig kunnen worden vastgesteld. Aan een construct kunnen verschillende mensen een verschillende betekenis toekennen. Alle criteria van het model zijn een construct.

In het kader van mijn model maak ik onderscheid tussen enerzijds criteria (constructen) waaraan de meeste mensen ongeveer dezelfde betekenis toekennen en anderzijds criteria waaraan veel mensen een verschillende betekenis toekennen. De criteria van het model waaraan veel mensen een verschillende betekenis toekennen zijn met name:
- noodzakelijkheid;
- verantwoordelijkheid;
- geschiktheid;
- indringendheid;
- rechtvaardigheid;
- rechtsgelijkheid.

Voor de toetsing van de gerechtvaardigdheid van de preventiemaatregel aan criteria waaraan veel mensen een verschillende betekenis toekennen, moet (deels) worden teruggegrepen op argumenten waarvan de juistheid (nog) niet wetenschappelijk kan worden bewezen. Het gaat om argumenten op het morele, politiek-ideologische, sociaal-culturele, juridische en/of financieel-economische vlak (zie bijlage 6). Wellicht zijn er nog meer invalshoeken van het denken te bedenken (esthetisch, emotioneel, enz.). Voor de toetsing van de criteria waaraan de meeste mensen ongeveer dezelfde betekenis toekennen, kan in het algemeen wel gebruikt worden gemaakt van wetenschappelijk te bewijzen argumenten.

4. Als in deze paragraaf wordt verwezen naar de beoordelingscriteria of beoordelingszeven van het model wordt, tenzij anders aangegeven, gedoeld op versie 6.

In mijn visie is er in een rationeel beoordelingsproces pas plaats voor argumenten waarvan de juistheid niet wetenschappelijk kan worden bewezen, als de mogelijkheden om de beoordeling te baseren op wetenschappelijke (objectieve) argumenten zijn uitgeput. Dat kan hier het geval zijn, omdat bepaalde wetenschappelijke kennis nog niet beschikbaar is (er is bijvoorbeeld nog weinig empirische kennis over de effecten en neveneffecten van wetgeving ter bestrijding van overgewicht), maar ook omdat bepaalde zaken zich, zoals hierboven toegelicht, niet wetenschappelijk (objectief) laten bewijzen. Als bepaalde wetenschappelijke kennis nog niet beschikbaar is, kan het voorzorgsbeginsel (Engels: precautionary principle) een belangrijke rol spelen bij het rechtvaardigen van leefstijlbeïnvloedende interventies. Dit geldt met name als er sprake is van een groot risico op grote schade (Harris, 2009, p.221).

Bij een rationeel beoordelingsproces wordt zoveel mogelijk gebruik gemaakt van wetenschappelijke onderzoeksgegevens, die kwantitatief of kwalitatief van aard kunnen zijn. Met het oog op de objectiviteit, vergelijkbaarheid en generaliseerbaarheid van de beoordeling verdient het gebruik van kwantitatieve onderzoeksgegevens de voorkeur boven het gebruik van kwalitatieve onderzoeksgegevens (HM Treasury, 2003, p.4). Kwalitatieve gegevens dienen alleen te worden gebruikt als toereikende kwantitatieve gegevens redelijkerwijs niet te verkrijgen zijn. In overzicht 7 wordt mijn visie op de juiste volgorde van de argumentatie in een rationeel beoordelingsproces schematisch weergegeven.

Overzicht 8: fasering van de argumentatie in een rationeel beoordelingsproces

1. Argumenten op basis van *kwantitatieve* wetenschappelijke onderzoeksgegevens

↓

2. Argumenten op basis van *kwalitatieve* wetenschappelijke onderzoeksgegevens

↓

3. Niet-wetenschappelijk bewezen argumenten die niet in strijd zijn met wetenschappelijke onderzoeksgegevens of met de logica.

De RVZ pleit ervoor om beslissingen over het basispakket van zorgverzekeringen in twee fasen te nemen (RVZ, 2006, p.44) (Oortwijn, 2006, p.146). In de eerste fase, de ***assessment fase***, wordt op basis van kwantitatieve onderzoeksgegevens een principebesluit genomen. In de tweede fase, de ***appraisal fase***, wordt dit principe-

besluit maatschappelijk getoetst (Bal, 2006). Deze fasering heeft kenmerken van de fasering die ik heb weergegeven in overzicht 8. De fasering die de RVZ voorstelt, vormt echter een groot probleem voor de inhoudelijk logische volgorde van de criteria binnen mijn model. De reden hiervan is dat de inhoudelijke beoordeling van de criteria in mijn model afhankelijk is van de inhoudelijke beoordeling van de voorafgaande criteria, die deels worden bepaald door niet-wetenschappelijk te bewijzen opvattingen.

Voor de rationaliteit acht ik de inhoudelijk logische volgorde van de criteria (zie §4.3.4) nog belangrijker dan de logische fasering van de argumentatie conform overzicht 8. Als de criteria in de verkeerde volgorde worden gehanteerd, gaat dat sterk ten koste van de validiteit van de beoordeling, of gebeurt er werk voor niets (bv. als preventieve interventie niet noodzakelijk is). Ter illustratie enkele voorbeelden:

- het is onlogisch om de noodzakelijkheid (2e criterium) te beoordelen, voordat de schadelijkheid (1e criterium) is vastgesteld;
- het is onlogisch om de oorzakelijkheid (2e criterium) te beoordelen, voordat de noodzakelijkheid (1e criterium) is vastgesteld;
- het is onlogisch om de effectiviteit (9e criterium) te beoordelen, voordat de noodzakelijkheid (2e criterium) is vastgesteld;
- het is onlogisch om de rechtvaardigheid (11e criterium) te beoordelen, voordat de verantwoordelijkheid (4e criterium) is vastgesteld.

Bij het ontwerpen van de structuur van mijn model heb ik mij daarom primair laten leiden door de inhoudelijk logische volgorde van de argumentatie en secundair door de rationele fasering van een beoordelingsproces conform overzicht 8.

Om de logische fasering van de argumentatie conform overzicht 8 toch te verankeren in mijn model, heb ik het model opgebouwd uit 7 clusters van twee criteria:

- **Cluster 1:**
  1. Schadelijkheid
  2. Noodzakelijkheid
- **Cluster 2:**
  3. Oorzakelijkheid
  4. Verantwoordelijkheid
- **Cluster 3:**
  5. Gerichtheid
  6. Geschiktheid

- **Cluster 4:**
  7. Effectiviteit
  8. Indringendheid
- **Cluster 5:**
  9. Kosteneffectiviteit
  10. Lasten-batenverhouding
- **Cluster 6:**
  11. Rechtvaardigheid
  12. Rechtsgelijkheid
- **Cluster 7:**
  13. Draagvlak
  14. Uitvoerbaarheid

De criteria binnen een cluster kennen een inhoudelijke samenhang. Zowel de clusters als de criteria binnen een cluster kennen inhoudelijk gezien een logische volgorde. Omdat een onderverdeling tussen een eerste fase, assessment fase, en een tweede fase, appraisal fase, zoals de RVZ voorstelt, voor mijn model niet mogelijk is, heb ik getracht een vergelijkbaar onderscheid binnen ieder cluster terug te laten komen. In het algemeen is het eerste criterium binnen een cluster voor de beoordeling minder afhankelijk van niet-wetenschappelijk te bewijzen opvattingen dan het tweede criterium:

- Cluster 1:
  - Eerst wordt de schadelijkheid (1e criterium) van de leefstijl waarop de preventiemaatregel zich richt, zoveel mogelijk op basis van wetenschappelijke informatie beoordeeld. Het gaat hierbij om de aard en de omvang van de risico's op schade, op overlast en op schending van morele opvattingen, die men met de preventiemaatregel tracht te bestrijden.
  - Pas daarna wordt op basis van dit oordeel bepaald in hoeverre het noodzakelijk (2e criterium) is om deze risico's te bestrijden. Is de doelstelling van de preventiemaatregel niet ***perfectionistisch of moralistisch?*** Wordt met de preventiemaatregel niet een zinvollere doelstelling van het preventiebeleid verdrongen? Vooral bij de beoordeling van het perfectionistische en moralistische gehalte van de preventiemaatregel en de (on)wenselijkheid hiervan spelen niet-wetenschappelijk bewijsbare opvattingen een rol.
- Cluster 2:
  - Eerst worden de determinanten (3e criterium: oorzakelijkheid) van de risico's op schade, op overlast en op schending van morele opvattingen beschreven. Deze beschrijving wordt zoveel mogelijk gebaseerd op wetenschappelijke informatie. Het gaat hierbij om de risico's waarvan bij het 1e criterium is vastgesteld dat men die wil bestrijden, maar vooral om de risi-

co's waarvan bij het 2e criterium is vastgesteld dat bestrijding noodzakelijk is.
  - Pas daarna wordt op basis van deze beschrijving bepaald welke partijen verantwoordelijk (4e criterium) zijn voor het ontstaan en/of de instandhouding van deze risico's. Benadeelt men met de leefstijl zichzelf of derden? Dit is van belang om te beoordelen of er sprake is van ***paternalisme***. Vooral bij de beoordeling van het paternalistische gehalte van de preventiemaatregel en de (on)wenselijkheid hiervan spelen niet-wetenschappelijk bewijsbare opvattingen een rol.
- Cluster 3:
  - Eerst wordt beoordeeld of de preventiemaatregel is gericht (5e criterium) op de juiste doelgroep of omgevingsfactoren. De juiste gerichtheid wordt zoveel mogelijk logisch afgeleid van de voorafgaande criteria (schadelijkheid, noodzakelijkheid, oorzakelijkheid en verantwoordelijkheid).
  - Pas daarna wordt beoordeeld of de partij die is gekozen voor de uitvoering van de preventiemaatregel daarvoor geschikt (6e criterium) is. De geschiktheid van een bepaalde partij voor uitvoering van de preventiemaatregel kan slechts in beperkte mate worden afgeleid van de voorafgaande criteria. Het oordeel over de geschiktheid wordt in belangrijke mate bepaald door de opvattingen die men heeft over de bekwaamheid en (morele) intenties van de betreffende partij en door de politiek-ideologische voorkeur die men heeft voor zelfregulering, private regulering en/of overheidsregulering. Ter illustratie van opvattingen over (im)morele intenties: over de tabaksindustrie is bijvoorbeeld beweerd dat deze sigaretten met additieven opzettelijk extra verslavend heeft gemaakt (Bates, 1999).
- Cluster 4:
  - Eerst wordt de effectiviteit (7e criterium) van de preventiemaatregel zoveel mogelijk op basis van wetenschappelijke informatie beoordeeld. De effectiviteit kan kwantitatief worden uitgedrukt in bijvoorbeeld een toename van QALY's, DALY's, of een reductie van ziektelast.
  - Pas daarna wordt de indringendheid (8e criterium) van de preventiemaatregel beoordeeld, door mij gedefinieerd als risico op schending van individuele mensenrechten (aantasting van de lichamelijke integriteit, van de vrijheid, van de privacy, van de gelijke behandeling, van de eer en goede naam, van de waardigheid en respectvolle bejegening). Dit risico is moeilijker te kwantificeren en te objectiveren dan de effectiviteit van de preventiemaatregel. Bij het beoordelen van de indringendheid spelen niet-wetenschappelijk bewijsbare (morele, politiek-ideologische, sociaal-culturele, e.d.) opvattingen waarschijnlijk een grote rol.

- Cluster 5:
  - Eerst wordt de kosteneffectiviteit (9e criterium) van de preventiemaatregel zoveel mogelijk op basis van wetenschappelijke informatie beoordeeld. De effectiviteit is, in het kader van de 4e zeef, reeds zoveel mogelijk op basis van wetenschappelijke informatie beoordeeld. De kosten kunnen kwantitatief worden uitgedrukt in geld.
  - Daarna kan de lasten-batenverhouding (10e criterium) worden beoordeeld. De lasten-batenverhouding betreft naast de beoogde effecten ook alle neveneffecten (positief en negatief). Omdat neveneffecten niet beoogd zijn, zal niet altijd duidelijk zijn welke neveneffecten in het onderzoek moeten worden meegenomen (Hauck, 2004, p.7). Bovendien betreffen de neveneffecten vaak zaken waarvoor nog geen of slechts in beperkte mate objectieve c.q. kwantitatieve uitkomstmaten beschikbaar zijn (Baron J. 1997) (Hauck, 2004, p.7-9) (Rutten, 2006, p.53-56), zoals bijvoorbeeld de indringendheid van de preventiemaatregel. Hierdoor biedt een wetenschappelijke beoordeling van de lasten-batenverhouding (10e criterium) minder objectieve zekerheid dan van de kosteneffectiviteit (9e criterium).
- Cluster 6:
  - Eerst wordt de distributieve rechtvaardigheid (11e criterium: rechtvaardigheid) beoordeeld, die de vraag betreft of de baten en lasten van de preventiemaatregel rechtvaardig over de betrokken partijen worden verdeeld (zie voor een toelichting van het begrip 'distributieve rechtvaardigheid' de bespreking van 'Rij IX van overzicht 2 in §2.6.2). De distributieve rechtvaardigheid beperkt zich tot de rechtvaardige verdeling van de baten en lasten ten gevolge van de preventiemaatregel. Welke baten en lasten dit zijn, is vastgesteld bij de lasten-batenverhouding (10e criterium). Een groot probleem bij het beoordelen van de distributieve rechtvaardigheid is, dat er verschillende concepten van rechtvaardigheid bestaan, die met elkaar om voorrang strijden. Uit een literatuur-review in een discussienota ten behoeve van projecten van de Wereldbank worden maar liefst zeven concepten van rechtvaardigheid genoemd (Hauck, 2004, 23-36):
    1. Egalitarianism, which implies that everybody should have identical health status [gelijke gezondheid voor iedereen];
    2. Allocation according to need, which relies on an adequate definition of 'need' [allocatie van gezondheidszorg naar behoefte];
    3. Rule of rescue, which demands that it is an ethical duty to do everything possible to help individuals in immediate lifethreatening situations [plicht tot het afwenden van levensbedreigende situaties];

4. Equality of access, which is often used to operationalize the concept of equity but itself requires a definition of 'access' as well as 'need' [gelijke toegang tot gezondheidszorg voor iedereen];
5. Providing a decent minimum, which involves definition of an essential package of health services [beschikbaarheid van een basaal fatsoenlijk minimum aan gezondheidszorg voor iedereen];
6. Rawls' maximin principle, which demands that social policy should seek to maximize the position of the worst-off [het maximaliseren van de positie van de mensen die het slechtst af zijn];
7. Libertarianism, which favors a distribution of resources according to entitlement [gewaarborgd dient te worden dat iedereen krijgt waar hij recht op heeft; het herverdelen van middelen om gelijkheid te bevorderen, is geen taak voor de overheid].

In de wetenschap en in de samenleving bestaat nog geen consensus hoe de verschillende concepten van rechtvaardigheid bij de beoordeling van de verdeling van kosten en baten van preventiemaatregelen tegen elkaar gewogen moeten worden (Oortwijn, 2006, p.142). Hauck e.a. (2004, p.33-34) stellen:

> *"It is important to recognize that any notion of fairness is ultimately a personal construct and that no concept is in any sense more legitimate than another."*

Nagenoeg ieder mens hanteert meerdere (van de genoemde) concepten van rechtvaardigheid naast elkaar. Afhankelijk van het gewicht dat men op basis van morele en/of politiek-ideologische overwegingen aan verschillende concepten van rechtvaardigheid toekent, leidt de beoordeling van de distributieve rechtvaardigheid van de preventiemaatregel tot andere uitkomsten. Hauck e.a. (2004, p.2) stellen echter ook:

> *"However, most equity considerations fall into two broad headings: equity related to a concept of need and equity related to access to services and, in principle, equity concerns can be incorporated into an economic approach to priority setting with relative ease.".*

Dit betekent dat de beoordeling van de distributieve rechtvaardigheid relatief eenvoudig gekwantificeerd c.q. geobjectiveerd kan worden, als geëxpliciteerd is op basis van welk concept van rechtvaardigheid de distributieve rechtvaardigheid wordt beoordeeld. Bij de toetsing van de gerechtvaardigdheid van de preventiemaatregel aan het criterium 'distributieve rechtvaardigheid' zijn (tenminste) twee aspecten van belang: (1) de verdeling van

beschikbare middelen (verdeling van middelen naar behoeften) en (2) de verdeling van kansen (gelijke toegang tot voorzieningen).
  - Pas daarna wordt de vergelijkende rechtvaardigheid (12e criterium: rechtsgelijkheid) beoordeeld, die de vraag betreft of in vergelijkbare situaties vergelijkbaar wordt gehandeld (zie voor een toelichting van het begrip 'vergelijkende rechtvaardigheid' de bespreking van 'Rij IX van overzicht 2 in §2.6.2). Zou het bijvoorbeeld niet onrechtvaardig zijn om mensen met overgewicht (boulimia) wel financieel aansprakelijk te stellen voor hogere zorgkosten die hieruit voortvloeien en mensen met ondergewicht (anorexia) niet? De vergelijkende rechtvaardigheid kan betrekking hebben op zeer uiteenlopende gedragingen van mensen met risico op leefstijlgerelateerde aandoeningen: te veel of te weinig eten, te veel of te weinig bewegen, te veel of te weinig rust nemen, roken, overmatig alcohol- of drugsgebruik, enz. Evenals het bij de beoordeling van de lasten-batenverhouding moeilijk te bepalen is welke neveneffecten bij de beoordeling moeten worden meegenomen, is het bij het beoordelen van de vergelijkende rechtvaardigheid (zeer) moeilijk te bepalen 'waarmee' en 'hoe' vergeleken moet worden. De distributieve rechtvaardigheid speelt zich af binnen het kader van de baten en lasten van de preventiemaatregel, terwijl de vergelijkende rechtvaardigheid zich afspeelt binnen én buiten het kader van de baten en lasten van de preventiemaatregel. De vergelijkende rechtvaardigheid is m.i. daardoor moeilijker objectief te beoordelen dan de distributieve rechtvaardigheid.
- Cluster 7:
  - Het criterium 'draagvlak' betreft het heden. Het draagvlak is empirisch te onderzoeken door bijvoorbeeld mensen te interviewen over hun steun voor en/of bezwaar tegen de preventiemaatregel.
  - Het criterium 'uitvoerbaarheid' betreft de haalbaarheid en houdbaarheid en daarmee respectievelijk het heden en de toekomst. Met name de houdbaarheid is daardoor moeilijker op basis van wetenschappelijk onderzoek te bepalen dan het draagvlak (en dan de haalbaarheid).

De indeling van mijn beoordelingsmodel in 7 clusters van elk 2 criteria heb ik gebruikt voor het bepalen van de juiste volgorde van de beoordelingscriteria bij de bouw van het model. Om het model voor de gebruiker niet te complex te maken, kies ik er voor om deze clusters in het beoordelingsmodel niet expliciet zichtbaar te maken.

4 Wijze van beoordeling bij het model *(versie 6 van het model)*

4.1 Inleiding

In 2000 publiceert Norman Daniels een kort maar invloedrijk[1] artikel in de British Medical Journal onder de titel *"**Accountability for reasonableness**. Establishing a fair process for priority setting is easier than agreeing on principles"* (Daniels, 2000). De 'priority setting' waar Daniels op doelt, betreft de noodzakelijke keuzen in de gezondheidszorg[2], de keuzen van de overheid om bepaalde zorg wel en andere niet op te nemen in het basispakket. Dit met het oog op de grenzen aan de voor gezondheidszorg beschikbare middelen. Daniels acht het onmogelijk om consensus te bereiken over de inhoudelijke principes die bepalend zijn voor de keuzen in de zorg. Hij ontkent echter niet dat het mogelijk is om consensus te bereiken over een ***set criteria*** die een leidraad biedt bij het maken van keuzen in de gezondheidszorg. Acht jaar later, in 2008, schrijft hij hierover:

> *"… some people insist that process alone cannot tell us what is right or fair and we need other, substantive criteria. Sometimes we can agree on such criteria, and then we may not need an appeal to a fair process. The process does not rule out agreement, but no principles exist to resolve many of the disagreements noted above and we are unlikely to arrive at consensus on them in the near future."* (BMJ, 2008, p.905).

Denk bij een set criteria bijvoorbeeld aan de criteria van de Trechter van Dunning of aan de criteria van het beoordelingsmodel. Volgens Daniels is het echter niet mogelijk om in pluralistische (Westerse) samenlevingen consensus te bereiken over de morele of ideologische principes op basis waarvan keuzen worden gemaakt. In zijn artikel *"How many principles for public health ethics"* trekt Steven Coughlin een vergelijkbare conclusie:

> *"This article has considered general moral principles that play a prominent role in certain methods of moral reasoning in public health and biomedicine … None of the*

1. *"Accountability for reasonableness helped shape thinking about how the National Institute for Health and Clinical Excellence (NICE) should incorporate social value judgments into its recommendations to the National Health Service in England and Wales."* (Donaldson, 2008, p.904)
2. Denk hierbij aan de Commissie Keuzen in de zorg, die de 'Trechter van Dunning' heeft ontwikkeld.

principles … have been confirmed as "first principles" or "ultimate principles" that incontrovertibly fix the foundations of moral reasoning …" (Coughlin, 2008, p.15).

In een pluralistische samenleving denken mensen bijvoorbeeld verschillend over de juiste operationalisering van het principe van distributieve rechtvaardigheid (zie de bespreking van cluster 6 van het model in §3.3). Als het dan niet mogelijk is om consensus te bereiken over de inhoudelijke principes die bepalend zijn voor de keuzen in de zorg, kan volgens Daniels tenminste worden getracht om consensus te bereiken over de wijze waarop keuzen worden gemaakt, d.w.z. afspraken over een ***fair keuzeproces***. Daniels verwoordt dit als volgt (Daniels, 2000, p.1300):

"In pluralist societies we are likely to find reasonable disagreement about principles that should govern priority setting. For example, some will want to give more priority to the worst off, some less, some will be willing to aggregate benefits in ways that others are not. In the absence of consensus on principles, a fair process allows us to agree on what is legitimate and fair. Key elements of a fair process will involve [1] transparency about the grounds for decisions; [2] appeals to rationales that all can accept as relevant to meeting health needs fairly; and [3] procedures for revising decisions in light of challenges to them. Together these elements assure "accountability for reasonableness"."

De kernelementen van een fair keuzeproces die Daniels noemt, betreffen de ***argumentatie*** (de gebruikte informatie en gevolgde redeneringen) die ten grondslag ligt aan de besluitvorming en de ***besluitvormingsprocedure*** (welke partijen kunnen op welke moment welke invloed uitoefenen op de besluitvorming?). "Accountability for reasonableness" kan m.i. vrij worden vertaald met verantwoordelijkheid voor de redelijkheid en billijkheid van de argumentatie en van de besluitvormingsprocedure. De gerechtvaardigdheid van de besluitvormingsprocedure (Engels: procedural justice) valt buiten het kader van mijn model (zie §1.3). Deze zal ik daarom niet uitwerken. De gerechtvaardigdheid van de argumentatie vormt de kern van mijn model. Deze zal ik daarom wel uitwerken.

Van de drie kernelementen (key elements) van een fair keuzeproces die Daniels noemt, kunnen ten behoeve van mijn beoordelingsmodel, eisen aan de argumentatie worden afgeleid. Ik zal deze kernelementen en de betekenis voor mijn beoordelingsmodel achtereenvolgens bespreken, te weten:

- transparantie over de argumentatie die aan besluiten ten grondslag ligt (§4.2)
- gebruik van redeneringen die voor iedereen aanvaardbaar zijn (§4.3)
- procedures voor het herzien van besluiten als de onderbouwing onder druk staat (§4.4).

4.2 Transparantie over de argumentatie die aan besluiten ten grondslag ligt

Mijn model richt zich op de beoordeling van de gerechtvaardigdheid van de argumentatie die ten grondslag ligt aan de toepassing van leefstijlbeïnvloedende preventiemaatregelen. Onder de gerechtvaardigdheid van de argumentatie versta ik de redelijkheid en billijkheid van de argumentatie. De redelijkheid en billijkheid van de argumentatie kunnen alleen worden beoordeeld als de argumentatie, die ten grondslag ligt aan het besluit tot het al dan niet toepassen van de preventiemaatregel, transparant is. Daarom zie ik transparantie van de argumentatie in het kader van het model als een algemene voorwaarde voor de rechtvaardiging van een preventiemaatregel. De partij die verantwoordelijk is voor de toepassing van een leefstijlbeïnvloedende preventiemaatregel dient idealiter per criterium van het model gemaakte keuzes te onderbouwen en deze onderbouwing publiekelijk kenbaar te maken. Als de onderbouwing aantoonbaar onjuist is (strijdig met wetenschappelijke informatie of onlogisch) of verwijtbaar onvolledig is (bijvoorbeeld omdat beschikbare wetenschappelijke informatie wordt genegeerd), is de toepassing van de preventiemaatregel niet gerechtvaardigd. De toepassing van de preventiemaatregel is echter ook niet gerechtvaardigd als de genoemde partij tekortschiet in het publiekelijk kenbaar maken van de onderbouwing, omdat de juistheid en volledigheid van de argumentatie dan niet publiekelijk beoordeeld kunnen worden. Daniels (2000, p.1301) stelt in dit verband:

> *"A fair process requires publicity about the reasons and rationales that play a part in decisions. There must be no secrets where justice is involved, for people should not be expected to accept decisions that affect their well being unless they are aware of the grounds for those decisions."*

In 2002 publiceren James Childress e.a. een artikel over ethiek van de public health, waarin zij de ideeën van Daniels over een fair keuzeproces hebben verwerkt. Zij geven een opsomming van algemene morele overwegingen die relevant zijn voor de rechtvaardiging van preventiemaatregelen in de public health:

- a. *producing benefits;*
- b. *avoiding, preventing, and removing harms;*
- c. *producing the maximal balance of benefits over harms and other costs (often called utility);*
- d. *distributing benefits and burdens fairly (distributive justice) and ensuring public participation, including participation of affected parties (procedural justice);*
- e. *respecting autonomous choices and actions, including liberty of action;*
- f. *protecting privacy and confidentiality;*
- g. *keeping promises and commitments;*

h. *disclosing information as well as speaking honestly and truthfully (often grouped under transparency); and*
i. *building and maintaining trust.*

De reden dat ik dit artikel van Childress e.a. niet heb gebruikt bij de ontwikkeling van de criteria van het beoordelingsmodel in de voorafgaande hoofdstukken, is dat ik pas na afronding van hoofdstuk 3 op dit artikel ben gestuit. De hierboven opgesomde algemene morele overwegingen (a t/m i) geven echter geen aanleiding tot aanpassing van de criteria of structuur van versie 6 van het beoordelingsmodel (zie hoofdstuk 3 en 4). Ik noem het artikel van Childress e.a. in deze paragraaf vanwege het onderscheid dat zij maken tussen enerzijds *'d. procedural justice'* (die buiten het kader van het model valt) en anderzijds *'h. transparency'* (die voor wat betreft de transparantie van de argumentatie binnen het kader van het model valt). In de visie van Childress e.a. betekent transparantie niet alleen het openbaar maken van informatie (*disclosing information*), maar ook het eerlijk en waarheidsgetrouw spreken c.q. argumenteren (*speaking honestly and truthfully*). Dit betekent dat manipulatie van informatie, bijvoorbeeld het verstrekken van onvolledige informatie ter beïnvloeding van de publieke opinie, dient te worden afgewezen.

Het belang van transparantie van de argumentatie die ten grondslag ligt aan de besluitvorming over de toepassing van de preventiemaatregel, wordt ook door anderen erkend. Zie de eerste en vierde aanbeveling van Michelle Mello voor het ontwerp van gelegitimeerde 'incentive programs' in de public health (Mello, 2008, p.197)[3]:

Recommendations for the design of legally compliant wellness incentive programs:
1. *Document program goals and the reasons why medical inquiries are needed to achieve them and why the program is a reasonable means of achieving them.*
2. *Limit incentives to modest rewards that are activity-based, not achievement-based.*
3. *Provide accommodations and reasonable alternative standards for persons with disabilities or health conditions.*
4. *Make detailed, comprehensible information about the program available to potential participants, including procedures for obtaining an accommodation or alternative standard and for challenging decisions made by the program.*
5. *Retain a third party to manage medical information and maintain a firewall between that information and the employer.*
6. *Emphasize the voluntary nature of participation.*

3. Mello schrijft deze aanbevelingen te hebben overgenomen van Alvarez en Soltis (2008).

4.3 Gebruik van redeneringen die voor iedereen aanvaardbaar zijn

Voor mijn beoordelingsmodel geldt dat ik alleen gebruik wil maken van redeneringen die voor iedereen c.q. algemeen aanvaardbaar zijn. Dit stelt grenzen aan de mogelijkheden om preventiemaatregelen binnen het kader van het model te toetsen aan harde, meetbare criteria. Verderop in deze paragraaf licht ik dit toe.

In het kader van het model baseer ik de beoordeling van de gerechtvaardigdheid van de preventiemaatregel op de volgende algemeen aanvaardbare uitgangspunten:

- beoordeling in plaats van toetsing van gerechtvaardigdheid (§4.3.1);
- beoordeling aan de hand van een uitputtende lijst van beoordelingscriteria
- (§4.3.2);
- beoordeling van de kracht en transparantie van het bewijs (§4.3.3);
- beoordeling van de logische samenhang tussen beoordelingscriteria (§4.3.4);
- beoordeling met het schadebeginsel als richtinggevend principe (§4.3.5);
- beoordeling met falsificatiebeginsel als richtinggevend principe (§4.3.6).

Per uitgangspunt zal ik toelichten waarom ik dit algemeen aanvaardbaar acht.

4.3.1 Beoordeling in plaats van toetsing van gerechtvaardigdheid

Onder toetsing van de gerechtvaardigdheid van een preventiemaatregel versta ik toetsing aan de hand van criteria die hard en meetbaar zijn:

- hard: de toetsing is relatief zwart-wit; de preventiemaatregel voldoet wel of niet aan het criterium;
- meetbaar: objectief kan worden vastgesteld in hoeverre de preventiemaatregel voldoet aan het criterium.

Een voorwaarde voor toetsing is echter dat er binnen de samenleving consensus bestaat over de toetsingscriteria. In geval van toetsing van de gerechtvaardigdheid van de toepassing van leefstijlbeïnvloedende preventiemaatregelen is dat niet het geval. Ter illustratie geef ik een voorbeeld.

> ***Voorbeeld***
> Stel dat frequent gebruik van een bepaalde drug leidt tot een afname van de gezonde levensverwachting met 3 jaar. Dat is een kwantitatieve maatstaf van het 'risico op schade' (1e criterium van het model). In hoeverre is het in dat geval 'noodzakelijk' (2e criterium van het model) om leefstijlbeïnvloedende preventiemaatregelen te treffen: zeer noodzakelijk/ noodzakelijk/ enigszins noodzakelijk/ niet noodzakelijk? En is de gezonde levensverwachting wel de juiste uitkomstmaat (het juiste toetsingscriterium)? Gaat het in dit geval niet meer om het risico op

schade aan het welzijn van de druggebruiker of zijn gezin? Of om het risico op schade aan de arbeidsproductiviteit? Stel dat frequent gebruik leidt tot een afname van de arbeidsproductiviteit met 7%. In hoeverre is het dan noodzakelijk om leefstijlbeïnvloedende preventiemaatregelen te treffen? Over de juiste keuze en betekenis van toetsingscriteria bestaat binnen de samenleving geen consensus.

In pluralistische samenlevingen is het niet mogelijk om toetsingscriteria voor de beoordeling van de gerechtvaardigdheid van leefstijlbeïnvloedende preventiemaatregelen vast te stellen die algemeen aanvaardbaar zijn. Volgens Daniels is het zelfs niet mogelijk om in pluralistische samenlevingen consensus te bereiken over de morele of ideologische principes op basis waarvan keuzen in de zorg worden gemaakt (zie §4.1). Een fair (redelijk en billijk) besluitvormingsproces vormt volgens hem de beste waarborg voor gerechtvaardigde keuzen in de zorg. Ik ben dit met Daniels eens. Om niet de suggestie te wekken dat de gerechtvaardigdheid van preventiemaatregelen met behulp van mijn model wordt getoetst aan de hand van harde, meetbare criteria, spreek ik in het kader van het model over de beoordeling van de gerechtvaardigdheid van preventiemaatregelen en niet over toetsing van de gerechtvaardigdheid. Bij de beoordeling van de gerechtvaardigdheid van een preventiemaatregel worden in het kader van mijn model ***de redelijkheid en billijkheid van de argumentatie*** die ten grondslag ligt aan de besluitvorming beoordeeld. Hoe deze beoordeling plaatsvindt, beschrijf ik hieronder.

4.3.2 Beoordeling aan de hand van een uitputtende lijst van beoordelingscriteria

De gerechtvaardigdheid van een preventiemaatregel kan alleen worden vastgesteld, als de maatregel wordt beoordeeld op alle voor de gerechtvaardigdheid van de maatregel relevante aspecten. Het werken met een checklist c.q. uitputtende lijst van beoordelingscriteria geeft de meeste zekerheid dat alle relevante aspecten worden beoordeeld. Aan mijn beoordelingsmodel stelt dit de eis dat alle denkbare argumenten die pleiten voor of tegen de gerechtvaardigdheid van alle denkbare leefstijlbeïnvloedende preventiemaatregelen onder een criterium van het model kunnen worden geplaatst. In de hoofdstukken 7 t/m 10 wordt de volledigheid van de set beoordelingscriteria van het model getoetst aan de hand van praktijkcasussen. Als een of meer argumenten worden gevonden die niet onder een criterium van het model kunnen worden geplaatst, dient de lijst van beoordelingscriteria te worden aangepast c.q. uitgebreid (dit is dus wel een harde vorm van toetsing!). Om te voorkomen dat een onhanteerbaar grote lijst ontstaat, zijn criteria in het model geclusterd tot overkoepelende criteria. Het criterium ‘indringendheid’ – dat betrekking heeft op lichamelijke integriteit, vrijheid, privacy, gelijke behandeling,

eer en goede naam, waardigheid en respectvolle bejegening – vormt hiervan een voorbeeld.

4.3.3 Beoordeling van de kracht en transparantie van het bewijs

In §4.2 heb ik betoogd dat de partij die besluit over toepassing van de preventiemaatregel, de argumentatie die hieraan ten grondslag ligt transparant moet maken. In het kader van mijn model versta ik onder transparantie dat deze argumentatie publiekelijk kenbaar is, d.w.z. voor iedereen vrij en eenvoudig toegankelijk is (opvraagbaar of opzoekbaar)[4]. Idealiter worden per criterium van het model gemaakte keuzes met argumenten onderbouwd. Overigens betekent dit niet, dat als voor bepaalde keuzes de onderbouwing zwak is of ontbreekt, de toepassing van de preventiemaatregel niet gerechtvaardigd is. In het algemeen kan worden gesteld, dat naarmate de lasten van een preventiemaatregel groter zijn, de onderbouwing van de gemaakte keuzes sterker en transparanter dient te zijn:

> *"While all programs must be based on sound data rather than informal speculation, the quality and volume of existing data will vary. The question for policy and ethics analysis, then, is what quantity of data is enough to justify a program's implementation? As a rule of thumb, the greater the burdens posed by a program – for example, in terms of cost, constraints on liberty, or targeting particular, already vulnerable segments of the population – the stronger the evidence must be to demonstrate that the program will achieve its goals."* (Kass, 2001, p.1778-1779)

Sterkere onderbouwing

De onderbouwing wordt sterker naarmate deze meer wordt ondersteund door onderzoeksgegevens en de wetenschappelijke bewijskracht van deze gegevens hoger is[5]. Met het oog op de objectiviteit en controleerbaarheid verdienen kwantitatieve onderzoeksgegevens de voorkeur boven kwalitatieve (zie overzicht 8 van §3.3).

4. In het kader van de procedurele rechtvaardigheid zou men ondermeer de volgende aanvullende eisen aan de transparantie kunnen stellen: de partij die de leefstijlbeïnvloedende preventiemaatregel toepast, dient actief met de belanghebbende partijen over het ontwerp, de consequenties en de implementatie van de preventiemaatregel te communiceren en zich ervan te vergewissen dat de informatie is ontvangen en begrepen. De procedurele rechtvaardigheid valt echter buiten het kader van het model.
5. Op het terrein van de volksgezondheid en gezondheidszorg is het bepalen en toepassen van de wetenschappelijke bewijskracht (level of evidence) een onderzoeksveld op zich, dat ik met het oog op de uitvoerbaarheid van mijn onderzoek niet nader uitwerk.

Transparantere onderbouwing
De onderbouwing wordt transparanter naarmate meer informatie over de argumentatie die aan de toepassing van de preventiemaatregel ten grondslag ligt, vrij en eenvoudig verkrijgbaar is. Naarmate de lasten van de preventiemaatregel groter zijn, kunnen er ten behoeve van de rechtvaardiging van de preventiemaatregel hogere eisen aan de transparantie worden gesteld.

Algemene aanvaardbaarheid
In de ene samenleving worden hogere eisen gesteld aan wetenschappelijke bewijslast en transparantie dan in de andere (BMJ, 2008, p.905). In de ene situatie worden hogere eisen gesteld aan wetenschappelijke bewijslast en transparantie dan in de andere. De ene persoon stelt hogere eisen aan wetenschappelijke bewijslast en transparantie dan de andere. De beoordeling van de redelijkheid en billijkheid van de kracht van het wetenschappelijke bewijs en van de geboden transparantie is dus contextafhankelijk (Childress, 2008, p.161-162). De stelling – dat naarmate de lasten van een preventiemaatregel groter zijn, de onderbouwing van de gemaakte keuzes sterker en transparanter dient te zijn – is m.i. in iedere context geldig en voor niemand bezwaarlijk (mits men te goeder trouw is). Daarom acht ik deze stelling algemeen aanvaardbaar als uitgangspunt voor de beoordeling van de gerechtvaardigdheid van een preventiemaatregel in het kader van het model.

4.3.4 Beoordeling van de logische samenhang tussen beoordelingscriteria

In §3.1 en 3.3 heb ik de logische samenhang tussen de beoordelingscriteria van mijn model uitgewerkt. Door de dwingende structuur van het model – de criteria dienen in een vaste volgorde te worden beoordeeld – wordt de beoordelaar geholpen bij het beoordelen van de logische samenhang tussen de beoordelingscriteria. Inhoudelijk dient de volgende logische samenhang te bestaan tussen de criteria:

– *Schadelijkheid (1) – Noodzakelijkheid (2)*
  Is het toepassen van de preventiemaatregel wel noodzakelijk, als naar de ernst van de risico's wordt gekeken die men met de preventiemaatregel tracht te bestrijden? Is het treffen van de preventiemaatregel niet moralistisch of perfectionistisch?

– *Noodzakelijkheid (2) – Oorzakelijkheid (3)*
  Betreffen de oorzaken op basis waarvan de preventiemaatregel is ontworpen wel de risico's, waarvan men het noodzakelijk acht die te bestrijden? Als men het bijvoorbeeld alleen noodzakelijk acht om ernstig overgewicht te bestrijden,

moet men het ontwerp van de preventiemaatregel niet baseren op de oorzaken van overgewicht, maar op de oorzaken van ernstig overgewicht.

- *Oorzakelijkheid (3) – Verantwoordelijkheid (4)*
  Is bij het vaststellen van de verantwoordelijkheid voor het ontstaan van de risico's die men tracht te bestrijden wel rekening gehouden met alle bekende oorzaken c.q. determinanten van deze risico's? Is het treffen van de preventiemaatregel niet paternalistisch?

- *Oorzakelijkheid (3) – Gerichtheid (5)*
  Hangt de keuze van de doelgroep en/of leefstijlbeïnvloedende factoren waarop de preventiemaatregel zich richt logisch samen met de oorzaken c.q. determinanten van de risico's die men tracht te bestrijden?

- *Verantwoordelijkheid (4) – Gerichtheid (5)*
  Hangt de keuze van de doelgroep en/of leefstijlbeïnvloedende factoren waarop de preventiemaatregel zich richt logisch samen met de verantwoordelijkheid voor het ontstaan van de risico's die men tracht te bestrijden?

- *Gerichtheid (3) – Geschiktheid (6)*
  Hangt de keuze van de partij waaraan de uitvoering van de preventiemaatregel is toebedeeld logisch samen met de doelgroep en/of leefstijlbeïnvloedende factoren waarop de preventiemaatregel zich richt?

- *Gerichtheid (5) – Effectiviteit (7)*
  Is het niet effectiever om de preventiemaatregel te richten op een andere doelgroep en/of andere leefstijlbeïnvloedende factoren?

- *Geschiktheid (6) – Effectiviteit (7)*
  Is er geen geschiktere partij om de preventiemaatregel uit te voeren?

- *Effectiviteit (7) – Indringendheid (8)*
  Staat de indringendheid van de preventiemaatregel wel in verhouding tot de effectiviteit?

- *Effectiviteit (7) – Kosteneffectiviteit (9)*
  Staan de uitvoeringskosten van de preventiemaatregel wel in verhouding tot de effectiviteit?

- *Effectiviteit (7) & Indringendheid (8) & Kosteneffectiviteit (9) – Lasten-batenverhouding (10)*
  Is tenminste alles wat bekend is over de effectiviteit, indringendheid en kosteneffectiviteit meegenomen bij de beoordeling van de lasten-batenverhouding?

- *Verantwoordelijkheid (4) – Rechtvaardigheid (11)*
  Is in de verdeling van baten en lasten (distributieve rechtvaardigheid) verdisconteerd welke partijen verantwoordelijk zijn voor het ontstaan van de risico's die men tracht te bestrijden?

- *Lasten-batenverhouding (10) – Rechtvaardigheid (11)*
  Zijn in de rechtvaardige verdeling van baten en lasten (distributieve rechtvaardigheid) alle bekende baten en lasten verdisconteerd?

- *Rechtvaardigheid (11) – Rechtsgelijkheid (12)*
  Worden vergelijkbare partijen met vergelijkbare baten en lasten geconfronteerd (vergelijkende rechtvaardigheid)?

- *Rechtvaardigheid (11) & Rechtsgelijkheid (12) – Draagvlak (13)*
  Is de verdeling van baten en lasten (distributieve en vergelijkende rechtvaardigheid) acceptabel voor de doelgroep van de preventiemaatregel en voor de samenleving?

- *Draagvlak (13) – Uitvoerbaarheid (14)*
  Is de handhaafbaarheid van de preventiemaatregel in relatie tot het verwachte draagvlak meegewogen bij het beoordelen van de uitvoerbaarheid?

De hierboven beschreven samenhang tussen de criteria berust op logica en niet op morele of ideologische opvattingen:
- Dat geldt zelfs voor de samenhang tussen de criteria 'schadelijkheid' (1) en 'noodzakelijkheid' (2). Een partij kan er nog steeds voor kiezen dat de schending van morele opvattingen (bijvoorbeeld: homoseksualiteit is zondig) het treffen van een preventiemaatregel (bijvoorbeeld een wettelijk verbod op homoseksualiteit) noodzakelijk maakt. Het beoordelingsmodel dwingt de betreffende partij om een dergelijke keuze te expliciteren c.q. transparant te maken, maar niet om deze keuze te veranderen (dan zou het model niet neutraal zijn). Als een partij transparant maakt dat voornamelijk morele opvattingen de aanleiding vormen tot het treffen van de preventiemaatregel, is de kans wel groot dat hij hierop (met succes) wordt aangevallen. In Westerse samenlevingen wordt het in het algemeen niet aanvaard dat leefstijlbeïnvloeding

voornamelijk moreel gerechtvaardigd wordt. En al helemaal niet als er voor de betreffende morele opvattingen geen breed draagvlak bestaat.
- De 2e zeef van het model (effecten en neveneffecten) zou men als keuze voor een utilistische benadering van de rechtvaardiging van leefstijlbeïnvloeding kunnen interpreteren (bij een utilistische benadering vormt het nut van de interventie de rechtvaardiging).[6] De tweede zeef van het model past inderdaad in een utilistische benadering. In theorie kan een partij (bijvoorbeeld een overheid) kiezen voor een niet-utilistische, zuiver deontologische benadering van de rechtvaardiging van leefstijlbeïnvloeding (bij een deontologische benadering gaat het om de vraag of een handeling in zichzelf goed of slecht is). Bijvoorbeeld: harddrug- of alcoholgebruik is slecht en moet daarom worden verboden, ongeacht de effecten en neveneffecten van een dergelijk verbod. Het beoordelingsmodel dwingt de betreffende partij om een dergelijke keuze te expliciteren c.q. transparant te maken, maar niet om deze keuze te veranderen (dan zou het model moreel niet neutraal zijn). Als een partij transparant maakt, dat de effecten en neveneffecten van een preventiemaatregel voor hem van ondergeschikt belang of zelfs irrelevant zijn, is de kans wel groot dat hij hierop (met succes) wordt aangevallen. In Westerse samenlevingen wordt een dergelijke eenzijdige benadering in het algemeen niet aanvaard.

De structuur van het beoordelingsmodel is weliswaar dwingend voor de beoordelaar, maar moreel en ideologisch neutraal. Daarom acht ik de binnen het model vastgestelde samenhang tussen de beoordelingscriteria algemeen aanvaardbaar.

Zoals gezegd bestaat het model uit drie beoordelingszeven (zie overzicht 7 in §3.1):
- 1e zeef: logica van ontwerp (criteria 1 t/m 6)
- 2e zeef: effecten en neveneffecten (criteria 7 t/m 12)
- 3e zeef: implementatie (criteria 13 en 14).

In §3.1 heb ik beschreven dat de criteria van het model tegen elkaar gewogen mogen worden. Als op een bepaald criterium niet goed wordt gescoord, maar op een ander criterium wel, kan dat laatste de doorslag geven om de preventiemaatregel toch als gerechtvaardigd te beoordelen. De zeefstructuur impliceert dat alleen de criteria binnen eenzelfde zeef tegen elkaar gewogen mogen worden. M.i. vormt deze inperking geen aantasting van de morele en ideologische neutraliteit van het

6. Zie voor het onderscheid tussen een ***utilistische en deontologische benadering in bijlage 6*** onder het tweede voorbeeld van tegengestelde morele denkkaders (voorbeeld over drugsgebruik).

model, omdat deze inperking op logica en niet op morele en/of ideologische opvattingen berust.

Ik heb overwogen om binnen het kader van het model toe te staan dat 'de zeven als geheel' tegen elkaar gewogen mogen worden. Dat zou bijvoorbeeld kunnen betekenen dat wanneer de onderbouwing van de logica van het ontwerp (1e zeef) ontoereikend is, maar de effecten en neveneffecten (2e zeef) van de preventiemaatregel zeer positief zijn, dat laatste de doorslag zou kunnen geven om de preventiemaatregel toch als gerechtvaardigd te beoordelen. Ik heb echter besloten om hiervan af te zien:

- Als de doelstelling van de preventiemaatregel (onderdeel van de 1e zeef) niet gerechtvaardigd kan worden, kan logischerwijs niet worden beoordeeld of de effecten (2e zeef) positief zijn. De effecten betreffen immers (de bijdragen aan) het bereiken van de doelstelling.
- Als de implementatie (3e zeef) van de preventiemaatregel heel eenvoudig is, maar de effecten en neveneffecten (2e zeef) kunnen niet gerechtvaardigd worden, dan moet toch van implementatie worden afgezien. Desgewenst kan de preventiemaatregel als experiment worden geïmplementeerd (3e zeef), waarbij de effecten en neveneffecten (2e zeef) worden onderzocht.

Binnen een zuiver deontologische benadering kan men er wel toe beslissen om de preventiemaatregel te implementeren (3e zeef), als de doelstelling van de preventiemaatregel gerechtvaardigd is (1e zeef), maar de effecten en neveneffecten (2e zeef) de implementatie niet kunnen rechtvaardigen. Men bestempelt de effecten en neveneffecten (2e zeef) dan als irrelevant of van ondergeschikt belang, omdat men 'het goede' (bijvoorbeeld wat het geloof voorschrijft) wil doen, ongeacht de consequenties. Zoals eerder genoemd, wordt in Westerse samenlevingen een dergelijke eenzijdige benadering in het algemeen niet aanvaard.

Samenvattend

Inhoudelijk dient er een logische samenhang te bestaan tussen de criteria van mijn model. Het gaat hierbij meestal, maar hierop zijn uitzonderingen, om samenhang tussen opeenvolgende criteria: tussen criterium 1 en 2, tussen criterium 2 en 3, tussen criterium 3 en 4, enz. Mijn beoordelingsmodel kent drie zeven. Binnen een zeef mogen de criteria van deze zeef tegen elkaar worden gewogen. Een zwakke uitkomst van de beoordeling op een bepaald criterium kan worden gecompenseerd door een goede uitkomst op een andere criterium. Criteria afkomstig uit verschillende zeven mogen echter niet tegen elkaar worden gewogen. De drie beoordelingszeven mogen in hun geheel ook niet tegen elkaar worden gewogen.

4.3.5 *Beoordeling met schadebeginsel als richtinggevend principe*

Gostin stelt overeenkomstig het schadebeginsel (Gostin, 2007, p.830):

> *The risk of serious harm to other people is the most commonly asserted and well-accepted justification for public health regulation".*

Vrij vertaald stelt het schadebeginsel dat de vrijheid van mensen alleen mag worden ingeperkt om te voorkomen dat zij schade berokkenen aan derden. Schade die mensen zichzelf berokkenen, vormt volgens het schadebeginsel geen rechtvaardiging voor vrijheidsbeperkingen. Dit is een zeer liberaal uitgangspunt, dat veel mensen te ver gaat. Om het schadebeginsel toch toe te kunnen passen, wordt het schadebeginsel door deze mensen op twee manieren genuanceerd (Pope, 2005):

1. Zij rekken het begrip 'schade' op, zodat veel meer zaken er onder vallen (zie bijlage 5). Zij stellen bijvoorbeeld dat pornografie schade toebrengt aan de waardigheid van de vrouw (de waardigheid van de mens is een uitgangspunt van de mensenrechten) en dus verboden moet worden. Anderen vinden een verbod van pornografie moralistisch.
2. Zij stellen vergaande eisen aan de autonomie, keuzevrijheid, wilsbekwaamheid en geïnformeerdheid van mensen, voordat het schadebeginsel geldig is, d.w.z. paternalisme wordt afgewezen. Zij stellen bijvoorbeeld dat de *verplichte* bedenktijd van vijf dagen in de Wet afbreking zwangerschap (abortuswet) niet in strijd is met het schadebeginsel, omdat een ruime bedenktijd noodzakelijk is om tot een weloverwogen besluit te komen. Anderen vinden een verplichte bedenktijd bevoogdend (paternalistisch) en onnodig belastend voor de vrouw.

Dergelijke nuanceringen van het schadebeginsel zijn voor veel mensen noodzakelijk, omdat zij hun redeneringen baseren op een absolute opvatting van het schadebeginsel, d.w.z. dat paternalistische en moralistische vrijheidsbeperkingen rücksichtslos dienen te worden afgewezen. Een bezwaar van deze nuanceringen is dat het schadebeginsel hierdoor niet meer onderscheidend is, want met creatief nuanceren kunnen bijna alle vrijheidsbeperkingen onder het schadebeginsel worden gebracht. Voor het beoordelingsmodel gebruik ik een andere oplossing om het schadebeginsel algemeen aanvaardbaar te maken. Ik ga uit van een relatieve opvatting van het schadebeginsel. Dit betekent dat ik paternalistische en moralistische vrijheidsbeperkingen niet rücksichtslos afwijs, maar enkel stel dat deze minder sterk gerechtvaardigd zijn dan vrijheidsbeperkingen ter voorkoming van schade. Door het schadebeginsel relatief op te vatten, is het niet nodig om de definitie van het begrip 'schade' op te rekken, of om vergaande eisen aan de autonomie, keuzevrijheid, wilsbekwaamheid en geïnformeerdheid van mensen te stellen, om het schadebeginsel voor iedereen aanvaardbaar te maken. Op basis van een relatieve

opvatting van het schadebeginsel kom ik tot de volgende algemeen aanvaarde uitgangspunten voor de beoordeling van de gerechtvaardigdheid van vrijheidsbeperkingen (zie overzicht 9)[7]:

Overzicht 9: algemeen aanvaarde uitgangspunten voor beoordeling van aanvaardbaarheid van preventie

a. Preventie van ***schade aan rechtmatige belangen*** vormt een sterkere rechtvaardiging van een bepaalde leefstijlbeïnvloedende interventie dan ***bevordering van rechtmatige beleidsdoelen***[8]**.**
b. Preventie van ***schade of overlast*** vormt een sterkere rechtvaardiging van een bepaalde leefstijlbeïnvloedende interventie dan preventie van de ***schending van morele opvattingen***.
c. Preventie van schade die men berokkent ***aan derden*** vormt een sterkere rechtvaardiging van een bepaalde leefstijlbeïnvloedende interventie dan preventie van schade die men berokkent ***aan zichzelf*** (als het gaat om 'dezelfde' schade).
d. Preventie van schade die ***kinderen*** berokkenen aan zichzelf vormt een sterkere rechtvaardiging van een bepaalde leefstijlbeïnvloedende interventie dan preventie van schade die ***volwassenen*** berokkenen aan zichzelf (als het gaat om 'dezelfde' schade).
e. Naarmate de ***autonomie, keuzevrijheid, wilsbekwaamheid en geïnformeerdheid*** van mensen groter is, is paternalisme minder gerechtvaardigd.

In Westerse samenlevingen zijn de hierboven beschreven uitgangspunten (a t/m e) algemeen aanvaardbaar. In een samenleving zonder scheiding tussen kerk en staat (bijvoorbeeld een Islamitische staat) zou men geloofsvoorschriften, d.w.z. bepaalde morele opvattingen, als sterkste rechtvaardiging van een bepaalde leefstijl kunnen zien. In dat geval zijn deze uitgangspunten, met uitzondering van uitgangspunt a en b, algemeen aanvaardbaar. Binnen het kader van de 'relatieve opvatting van het schadebeginsel', zoals tot uitdrukking gebracht in de punten a t/m e, is een relatief smalle definitie van het begrip 'schade' voor iedereen aanvaardbaar. In het kader

7. Zie §2.6.2 voor een toelichting op de algemene aanvaardbaarheid van deze uitgangspunten.
8. Ter illustratie enkele voorbeelden van rechtmatige beleidsdoelen:
   – meer bewegen door de jeugd;
   – meer burenhulp voor bejaarden;
   – economische groei van 3% per jaar;
   – meer gelijkheid op de arbeidsmarkt tussen vrouwen en mannen en tussen allochtonen en autochtonen.

van het model heb ik het begrip ‘schade’ relatief smal gedefinieerd als gezondheidsschade, sociaal-economische schade, en inbreuk op het persoonlijke leven (zie §2.7.2). Als iedereen dezelfde smalle definitie van het begrip ‘schade’ hanteert, wordt het mogelijk om de gerechtvaardigdheid van de preventiemaatregel te beoordelen aan de hand van de uitgangspunten a t/m e.

4.3.6 Beoordeling met falsificatiebeginsel als richtinggevend principe

Het is heel moeilijk om met zekerheid vast te stellen dat een theorie juist is. Om de juistheid van een theorie te bewijzen, moet de juistheid van alle onderdelen van de theorie worden bewezen. Het is veel eenvoudiger om te bewijzen dat een theorie onjuist is. Om de onjuistheid van een theorie te bewijzen, hoeft enkel de onjuistheid van één onderdeel van de theorie te worden aangetoond. De wetenschap kan vooral vooruitgang boeken door in de empirie op zoek te gaan naar falsificaties van bestaande theorieën en door theorieën op basis van falsificaties aan te passen of te verwerpen. Dit is de kern van het falsificationisme van Karl Popper (Chalmers, 1976, p.58-91).

De gerechtvaardigdheid van toepassing van een leefstijlbeïnvloedende interventie is, evenals de juistheid van een wetenschappelijke theorie, moeilijk met zekerheid vast te stellen. De beoordeling van de gerechtvaardigdheid van toepassing van een leefstijlbeïnvloedende interventie komt daardoor in de praktijk neer op een zoektocht naar falsificatie van de gerechtvaardigdheid. Aan de hand van

- de vastgestelde criteria van het model,
- de vastgestelde samenhang tussen de criteria van het model,
- de kracht en transparantie van het bewijs, en
- het schadebeginsel als richtinggevend principe,

kan deze zoektocht systematisch plaatsvinden.

De uitspraak dat toepassing van een preventiemaatregel op basis van mijn model gerechtvaardigd is, betekent dus niet meer en niet minder dan dat het niet gelukt is om de gerechtvaardigdheid te falsificeren. Dit lijkt misschien teleurstellend, maar is het niet, want de wetenschap werkt niet anders. Bovendien zal het vaak wel mogelijk zijn om de gerechtvaardigdheid op onderdelen te falsificeren. Naar aanleiding daarvan kan de preventiemaatregel worden aangepast c.q. worden verbeterd, of worden verworpen. Het falsificatiebeginsel is moreel en ideologisch neutraal en daarom acht ik het algemeen aanvaardbaar.

4.4 Herzien van besluiten als de onderbouwing onder druk staat

Procedures voor het herzien van besluiten als de onderbouwing onder druk staat, betreffen primair de procedurele rechtvaardigheid van de besluitvorming over de toepassing van de preventiemaatregel. De procedurele rechtvaardigheid valt buiten het kader van mijn model. Mijn model betreft de gerechtvaardigdheid van de argumentatie waarmee de toepassing van de preventiemaatregel wordt onderbouwd.

Ik heb in de vorige paragraaf geconstateerd dat de beoordeling van de gerechtvaardigdheid van toepassing van een leefstijlbeïnvloedende interventie in de praktijk neerkomt op een zoektocht naar falsificatie van de gerechtvaardigdheid. In het kader van het beoordelingsmodel betekent dit de falsificatie van de (impliciete of expliciete) argumentatie waarmee de gerechtvaardigdheid van toepassing van de preventiemaatregel wordt onderbouwd. Deze argumentatie wordt gefalsificeerd als deze onjuist, onvolledig of onvoldoende transparant is. In het geval van falsificatie van de argumentatie zijn er twee opties:

- verbetering van de onderbouwing, bijvoorbeeld door beter gebruik te maken van beschikbare wetenschappelijke informatie of door meer transparantie te bieden;
- verbetering (van het ontwerp of de implementatie) van de preventiemaatregel.

Per criterium en per zeef van mijn model dient te worden overwogen of verbeteringen van de onderbouwing en/of verbeteringen van de preventiemaatregel mogelijk zijn. Als belangrijke verbeteringen niet worden doorgevoerd, terwijl zij wel haalbaar zijn (bijvoorbeeld het bieden van meer transparantie), is de toepassing van de preventiemaatregel niet gerechtvaardigd.

4.5 Samenvatting van de wijze van beoordeling

Mijn model richt zich op de beoordeling van de gerechtvaardigdheid van de argumentatie die ten grondslag ligt aan de toepassing van de preventiemaatregel. De procedurele rechtvaardigheid van de besluitvorming over toepassing van de preventiemaatregel valt buiten het kader van het model.

De gerechtvaardigdheid van een preventiemaatregel wordt in het kader van mijn model beoordeeld aan de hand van <u>achtereenvolgens</u> de volgende criteria en zeven van het model:

- criteria 1 t/m 6;
- 1e zeef;

- criteria 7 t/m 12;
- 2e zeef;
- criteria 13 en 14;
- 3e zeef.

Per criterium van het model worden de redelijkheid en billijkheid van de argumentatie die ten grondslag ligt aan de toepassing van de preventiemaatregel, beoordeeld. Deze beoordeling is eigenlijk een zoektocht naar falsificatie van de redelijkheid en billijkheid van de volgende aspecten van de argumentatie:

- ***Juistheid*** van de argumentatie (is de argumentatie niet strijdig met wetenschappelijke informatie of onlogisch?);
- ***Volledigheid*** van de argumentatie (is geen beschikbare wetenschappelijke informatie en zijn geen relevante argumenten over het hoofd gezien of genegeerd?);
- Kracht van het ***bewijs*** (is het bewijs voor de argumentatie sterk genoeg?);
- ***Transparantie*** van het bewijs (is de argumentatie voor iedereen vrij en eenvoudig toegankelijk?);
- ***Interne consistentie*** van de argumentatie (voldoet de preventiemaatregel aan de vastgestelde samenhang tussen de criteria van het model?);
- Verhouding tot het ***schadebeginsel*** (is de preventiemaatregel niet moralistisch, perfectionistisch, of paternalistisch?)
- Mogelijkheid van ***betere alternatieven*** (zijn geen betere alternatieven voor de vormgeving, het ontwerp, of de implementatie van de preventiemaatregel mogelijk?).

5 Inleiding bij casus 'Clarian Health' *(versie 6 van het model)*

'The proof of the pudding is in the eating'. Dat geldt ook voor het door mij ontwikkelde beoordelingsmodel. De juistheid, volledigheid en praktische toepasbaarheid van het model zal ik daarom toetsen door praktijkcases aan de hand van het model te beoordelen. Ten behoeve van deze beoordeling zal ik de criteria van het model in de komende hoofdstukken nader operationaliseren.

Aan de hand van de volgende criteria heb ik gezocht naar praktijkcases om het model aan te toetsen:

- de casus heeft betrekking op de preventie van leefstijlgerelateerde aandoeningen en er is sprake van drang of dwang;
- de casus is actueel (heeft niet in een ver verleden plaatsgevonden, zoals 'de drooglegging van Amerika');
- de casus is maatschappelijk relevant (preventie van de betreffende leefstijlgerelateerde aandoening is op dit moment maatschappelijk relevant);
- in de casus komen veel verschillende aspecten naar voren, die relevant zijn voor de gerechtvaardigdheid van interventies ter beïnvloeding van leefstijl (zie de toelichting op het derde deel van het literatuuronderzoek in §1.4.1);
- de casus is onderwerp van debat geweest (betrokken partijen hebben/ hadden kritiek op de maatregel);
- over de casus is niet alleen feitelijke informatie beschikbaar, maar er is ook informatie beschikbaar over de opvattingen van betrokken partijen over de maatregel (zie §3.3 over het belang van 'assessment' en 'appraisal');
- om bias te voorkomen, heeft 'onbewerkte informatie', die rechtstreeks uit de praktijk afkomstig is, de voorkeur (de opvattingen van mensen, zoals zij die zelf verwoorden, in plaats van de samenvatting van onderzoekers hiervan).

De zoektocht naar praktijkcases heeft plaatsgevonden via internet (PubMed, Picarta, Google), omdat daarmee over de hele wereld naar cases kon worden gezocht, die aan de bovenstaande criteria voldeden. De uitgebreide zoektocht via

internet, die in het najaar van 2007 en het voorjaar van 2008 beplaatsvond[1], heeft eigenlijk maar één casus opgeleverd die in grote mate aan alle criteria voldeed: het preventieplan van Clarian Health. Veel cases vielen af, omdat er geen sprake was van (substantiële) drang of dwang, of omdat ik via internet geen of weinig 'onbewerkte informatie' over de opvattingen van mensen over de preventiemaatregel kon vinden. Daarom heb ik het model getoetst aan het preventieplan van de Amerikaanse ziekenhuisketen Clarian Health (BusinessWeek, McGregor, 2007):

> ***"Being Unhealthy Could Cost You Money.*** *Clarian Health is taking a novel approach to reducing health-care costs: It's penalizing workers for indicators of poor health. In late June, the Indianapolis-based hospital system announced that starting in 2009, it will fine employees $10 per paycheck if their body mass index (BMI, a ratio of height to weight that measures body fat) is over 30. If their cholesterol, blood pressure, and glucose levels are too high, they'll be charged $5 for each standard they don't meet. Ditto if they smoke: Starting next year, they'll be charged another $5 in each check. Clarian has been making headlines for its aggressive and unusual approach to covering escalating health-care costs. Rather than taking the more common step of giving employees incentives for merely participating in its wellness programs, such as joining a smoking cessation group or using a health coach, Clarian is actually measuring outcomes. And unlike most employers, it is penalizing workers for poor health instead of rewarding them for taking healthy steps."*

De bovenstaande tekst vormt de inleiding van een artikel dat Jena McGregor in augustus 2007 heeft gepubliceerd in BusinessWeek. Deze inleiding geeft een korte samenvatting van het preventieplan dat de Amerikaanse ziekenhuisketen Clarian Health in 2009 wil invoeren (zie bijlage 7 voor het volledige artikel). Zoals Jena McGregor schrijft, heeft het preventieplan van Clarian tot veel commotie in de media geleid. Op internet is op nieuwspagina's en weblogs veel aandacht aan dit preventieplan besteed. Op weblogs hebben tientallen bezoekers hun mening gegeven. Voor mij boden de vele schriftelijke reacties een uitgelezen kans om het model ***empirisch te toetsen***. Aan de hand van de beoordelingscriteria van versie 6 van het model (zie §2.7.1) heb ik de volgende teksten geanalyseerd, die m.i. een goede afspiegeling vormen van alles wat in de media over dit preventieplan is bericht en van de reacties van burgers naar aanleiding van deze berichtgeving[2]:

- BusinessWeek, McGregor J. Being unhealthy could cost you money. August 2, 2007. ***Artikel***: 1282 woorden.

1. In het voorjaar van 2008 was het wettelijk rookverbod in de Nederlandse horeca (de 2e praktijkcasus waaraan ik het model zal toetsen) nog niet van kracht.
2. Naar mijn informatie is er bij de aanvang van de toetsing van het model in september 2008 nog niet wetenschappelijk over het preventieplan van Clarian Health gepubliceerd.

– Workforce Management, Wojcik J. Employer to fine unhealthy workers. Augustus 2007. ***Artikel***: 920 woorden.
– NBC5. Employer to obese employees: shape up or pay up. Indiana company charging employers who smoke, are obese. Augustus 10, 2007. ***Nieuwsbericht***: 527 woorden.
– FoxNews.com. Company to charge 'unhealthy' workers more for insurance. July 1, 2007. ***Nieuwsbericht***: 453 woorden.
– Big Fat Blog. Clarian Health, Others will dock your paycheck if you're fat. In de periode van 8 augustus 2007 t/m 9 september 2007. 'The fat acceptance ***weblog***': 32 reacties van bezoekers; 10.129 woorden. (www.bigfatblog.com).
– MSNBC, TodayShow. Company fines workers for being overweight. Augustus 10, 2007. 'The allDay ***weblog***': 35 reacties van bezoekers; 6.113 woorden.

Ten behoeve van de argumentatieve analyse van de zes bovenstaande teksten, heb ik deze tot één groot document samengevoegd en dit grote document vervolgens in inhoudelijk logisch samenhangende tekstdelen opgeknipt (in totaal 312 tekstdelen van gemiddeld 62 woorden). Voor ieder van deze tekstdelen heb ik beoordeeld op welke criteria van het model het betreffende tekstdeel betrekking heeft. Deze werkwijze levert opmerkelijke resultaten op. Er is verreweg het meest geschreven over het criterium 'indringendheid' (ruim 35% van de tekstdelen heeft hier betrekking op) en het minst over het criterium 'kosteneffectiviteit' (minder dan 1% van de tekstdelen heeft hier betrekking op). Het bronbestand (de zes teksten samengevoegd), mijn werkwijze en het door mij bewerkte bronbestand zijn besproken met en akkoord bevonden door mijn begeleiders (promotor en copromotor). Doordat er drie personen naar gekeken hebben in plaats van één persoon (de onderzoeker) is er sprake van een zeker mate van objectivering. Ondanks dat ik het bronbestand zorgvuldig heb bewerkt, en er sprake is van een zekere mate van objectivering, blijft de uitkomst van de gevolgde werkwijze mede afhankelijk van mijn persoonlijk oordeel. Ik ken daarom geen groot gewicht toe aan een kwantitatieve analyse van de resultaten van deze werkwijze en zal mij vooral richten op een kwalitatieve analyse. De beschreven werkwijze heeft de argumentatieve beoordeling van de zes genoemde teksten aan de hand van het beoordelingsmodel voor mij uitvoerbaar gemaakt. Belangrijk om op te merken is dat ik geen argumenten heb gevonden die niet onder een criterium van het model kunnen worden geplaatst. Dit is een aanwijzing dat de set criteria die ik heb ontwikkeld, volledig is[3].

3. Door intensief met de beoordeling van de casus Clarian Health aan de hand van het model bezig te zijn, kom ik in §7.5.3 tot de conclusie dat een criterium 'timing' aan het model moet worden toegevoegd. N.a.v. het bestuderen van de literatuur over de casus 'wettelijk rookverbod in de horeca' concludeer ik in §9.2 dat ook criterium 'flankerend beleid' aan het model moet worden toegevoegd.

De hoofdstukken 6, 7 en 8 betreffen achtereenvolgens de drie zeven van versie 6 van het beoordelingsmodel (zie overzicht 6 in §3.1):

- 1e zeef: logica van ontwerp (criteria 1 t/m 6);
- 2e zeef: effecten en neveneffecten (criteria 7 t/m 12);
- 3e zeef: implementatie (criteria 13 en 14).

In de hoofdstukken 6, 7 en 8 beoordeel ik de gerechtvaardigdheid van het preventieplan van Clarian aan de hand van het beoordelingsmodel. Ik volg hierbij de wijze van beoordeling, zoals samengevat in §4.5. In hoofdstuk 9 geef ik in §9.1 een overall beoordeling van de gerechtvaardigdheid van het preventieplan van Clarian. Ten slotte beoordeel ik in §9.2 de juistheid, volledigheid en bruikbaarheid van het model op basis van de voorafgaande paragrafen. Vanwege de beperkte omvang van deze inleiding (hoofdstuk 5) heb ik overwogen om deze inleiding als eerste paragraaf aan hoofdstuk 6 toe te voegen. Omdat deze inleiding ook betrekking heeft op de hoofdstukken 7, 8 en 9 heb ik hiervan afgezien.

6 Toetsing 1e zeef van het beoordelingsmodel aan casus 'Clarian Health' (versie 6 van het model)

In dit hoofdstuk beoordeel ik de gerechtvaardigdheid van het preventieplan van Clarian Health aan de hand van de criteria van de 1e zeef van het model (versie 6). De 1e zeef 'logica van het ontwerp' is samengesteld uit de criteria:
- schadelijkheid;
- noodzakelijkheid;
- oorzakelijkheid;
- verantwoordelijkheid;
- gerichtheid;
- geschiktheid.

Aan de hand van ieder afzonderlijk criterium tracht ik tot een eindoordeel over de gerechtvaardigdheid van het preventieplan van Clarian te komen. Dat is mogelijk als er doorslaggevende informatie over een criterium beschikbaar is. Als er weinig of geen doorslaggevende informatie over een criterium beschikbaar is, is dat niet mogelijk. Voor de bruikbaarheid van het model is dat geen probleem. Het model vereist dat op basis van de 1e zeef een uitspraak over de gerechtvaardigdheid (van het ontwerp) van de preventiemaatregel wordt gedaan, maar niet dat dit per criterium wordt gedaan. Binnen een zeef mogen de criteria immers tegen elkaar gewogen worden (zie §3.1). Achtereenvolgens bespreek in per criterium de operationalisering, de wijze van beoordeling en het oordeel over de casus.

6.1 Eerste criterium: schadelijkheid

6.1.1 Operationalisering en wijze van beoordeling

Operationalisering

Het criterium 'schadelijkheid' betreft de aard en omvang van de risico's op schade, op overlast en op schending van morele opvattingen, die men met de preventiemaatregel tracht te bestrijden (zie §2.7.2). Hierbij hanteer ik de definitie van het begrip 'risico', zoals die wordt gehanteerd in de verzekeringsbranche: risico = kans x gevolg. Ter verduidelijking van deze formule geef ik deze weer in een kruistabel.

Overzicht 10: risico = kans x gevolg

	**Kleine kans**	**Grote kans**
**Klein nadeel**	Beperkt risico	Risico
**Groot nadeel**	Risico	Ernstig risico

De aard van de risico's kent als invalshoeken:
- Wat voor soort schade, overlast of schending van morele opvattingen tracht men met de preventiemaatregel te bestrijden?
- Wie lopen risico op deze schade, overlast of schending van morele opvattingen:
  - personen met de ongezonde of onveilige leefstijl zelf of derden?
  - volwassenen of kinderen?

Ik onderscheid drie ***schadecategorieën*** (zie bijlage 5):
1. Gezondheidsschade (fysiek en psychisch)
2. Sociaal-economische schade:
   - schade aan sociaal-maatschappelijke belangen (bv. schade aan sociale contacten, aan maatschappelijke participatie, aan huisvesting);
   - schade aan sociaal-economische belangen (bv. schade aan huisvesting, aan bezit, aan opleiding, aan inkomen, aan arbeidsgeschiktheid, aan werk);
   - schade aan financieel-economische belangen (bv. schade aan bezit, aan inkomen, aan winst, aan concurrentiepositie).
3. Inbreuk op het persoonlijk leven (aantasting van: de lichamelijke integriteit, vrijheid, privacy, veiligheidsbeleving, gelijke behandeling, eer en goede naam, waardigheid, of respectvolle bejegening)[1].

Vooralsnog ga ik er niet toe over om de begrippen 'overlast' en 'schending van morele opvattingen' onder te verdelen in respectievelijk categorieën van overlast en categorieën van (schending van) morele opvattingen. Ik denk dat een dergelijke onderverdeling voor het model weinig toegevoegde waarde heeft en het model onnodig complex maakt.

1. In deze paragraaf geef ik de voorkeur aan de term ***'inbreuk op het persoonlijk leven'*** in plaats van de term 'schending van individuele mensenrechten' die ik in bijlage 5 heb gebruikt. De term 'inbreuk op het persoonlijk leven' verwijst niet naar een juridisch document (Universele Verklaring van de Rechten van de Mens) en is een preciezere benaming voor het soort schade waarop ik doel. Ook een kleine inbreuk op het persoonlijk leven, zoals bijvoorbeeld een lichte schending van de privacy, valt onder de criteria 'schadelijkheid' en indringendheid van het model, en om in dat geval te spreken van een schending van de mensenrechten, lijkt mij te zwaar. De term 'schending van individuele mensenrechten' is zo bezien een deelverzameling van de term 'inbreuk op het persoonlijk leven'. Ik heb ook overwogen om de term 'inbreuk op de persoonlijke levenssfeer' te gebruiken, maar deze term verwijst m.i. teveel naar de privésfeer, terwijl de inbreuk op het persoonlijk leven ook in de openbare ruimte kan plaatsvinden.

De omvang van de risico's die men met de preventiemaatregel tracht te bestrijden, dient zoveel mogelijk te worden geobjectiveerd c.q. gekwantificeerd (zie overzicht 7 in §3.1). Vaak kan dit door deze risico's in geld uit te drukken. Gezondheidsrisico's kunnen worden gekwantificeerd door deze uit te drukken in ***QALY's, DALY's, of reductie van ziektelast*** [2]. Voor het kwantificeren c.q. uitdrukken in geld van immateriële zaken – zoals overlast, inbreuk op het persoonlijk leven en schending van morele opvattingen – kan gebruik worden gemaakt van concepten als ***Willingness to Pay*** en ***Willingness to Accept*** [3]. Ook is het van belang om in kaart te brengen hoeveel mensen risico lopen op de betreffende schade of overlast en hoeveel mensen de morele opvattingen, die (mede) aanleiding vormen tot het treffen van de preventiemaatregel, aanhangen.

Bij de ontwikkeling van de criteria van het model (zie hoofdstuk 2) ben ik er aanvankelijk vanuit gegaan dat preventiemaatregelen ter bestrijding van leefstijlgerelateerde aandoeningen, primair gezondheidswinst tot doel hebben. Kostenbesparingen ten gevolge van de preventiemaatregel zijn in dat geval geen beoogd effect (criterium 'effectiviteit'), maar een gunstig neveneffect (criterium 'lasten-batenverhouding'). Uit de casus 'Clarian Health' blijkt dat de bestrijding van economische schade ook een doelstelling (beoogd effect) van een dergelijke preventiemaatregel kan zijn. Ten slotte is het mogelijk dat een dergelijke preventiemaatregel de bevordering van een bepaalde morele opvatting als doelstelling heeft. Denk bijvoorbeeld aan een preventiemaatregel die, ter preventie van geslachtsziekten, het hebben van wisselende seksuele contacten ontmoedigt. Een dergelijke maatregel kan (onder-meer) worden getroffen omdat men het hebben van wisselende seksuele contacten verwerpelijk acht.

Pas bij het 2e criterium 'noodzakelijkheid' van het model wordt beoordeeld of het bestrijden van de risico's waarop de preventiemaatregel zich richt, wel noodzake-

2. *"… Zo worden nieuwe geneesmiddelen toegelaten tot het verstrekkingenpakket wanneer de kosten per gewonnen* ***quality adjusted life year (QALY)*** *onder een drempelwaarde van 20.000 euro blijven. Over de hoogte van dit bedrag en de toepasbaarheid bij andere zorgvoorzieningen bestaat echter verschil van inzicht, niet in het minst vanwege grote internationale verschillen. In Australië geldt bijvoorbeeld een drempelwaarde van 25.000 euro terwijl in het Verenigd Koninkrijk de grens bij 44.000 euro is gelegd. Vanwege deze verschillen is op basis van een inventarisatie van alle willingness-to-pay benaderingen gezocht naar een gouden standaard voor de waarde van een QALY. Hoewel deze niet werd gevonden, is wel vast komen te staan dat gezondheid los van de bijdrage aan de economie via het human capital in de samenleving, een zelfstandige economische waarde heeft die minimaal 100.000 euro per QALY bedraagt. De drempelwaarden die in de praktijk worden gehanteerd zijn dus aan de lage kant."* (RIVM, 2008).
3. ***"Willingness to Accept (WTA)****: The amount that someone is willing to receive or accept to give up a good or service.* ***Willingness to Pay (WTA)****: The amount that someone is willing to give up or pay to acquire a good or service".* (HM Treasury, 2003, p.23 en 105)

lijk is. Bij het 1e criterium ‘schadelijkheid’ wordt uitsluitend beoordeeld of de doelstellingen van de preventiemaatregel helder omschreven en zo mogelijk gekwantificeerd zijn, zonder dat de beoordelaar zich bekommert over de (on)wenselijkheid van deze doelstellingen. De opvatting dat preventiemaatregelen ter bestrijding van leefstijlgerelateerde aandoeningen uitsluitend of primair gezondheidswinst als doelstelling dienen te hebben, acht ik met het oog op de waardeneutraliteit van het model geen geschikt uitgangspunt voor het model.

Wijze van beoordeling

Bij de beoordeling van de preventiemaatregel aan de hand van het criterium ‘schadelijkheid’ wordt beoordeeld of de beschikbare informatie een duidelijk beeld geeft van de aard en omvang van de risico’s die men met de preventiemaatregel tracht te bestrijden. Is deze informatie juist, volledig, voldoende gedetailleerd en voldoende gekwantificeerd? Als dat oordeel negatief uitvalt, wordt beoordeeld of de informatieverzameling over de risico’s die men tracht te bestrijden voldoende is geweest. Heeft men voldoende gebruik gemaakt van de mogelijkheden om deze risico’s te inventariseren en kwantificeren?

6.1.2 Beoordeling van casus Clarian Health aan de hand van criterium ‘schadelijkheid’

Gezondheidsschade (criterium schadelijkheid)

De geanalyseerde teksten bevatten kwantitatieve informatie over de (toekomstige) ongezondheid van de bevolking van Amerika, de bevolking van de staat Indiana, en van de werknemers van Clarian. NBC5 verwijst naar een studie van de Johns Hopkins School of Public Health:

> *“… by the year 2015, 75 percent of Americans will be overweight and over 40 percent will be obese.”*

NBC5 stelt verder:

> *“Indiana has the fourth-highest level of obesity and the second-highest smoking rate.”*

Fox News verwijst naar een studie van Thomson Health Care (Huse, 2007):

> *“… workers who were moderately obese had health-care costs that were 21 percent, or $670 a year, higher than workers with normal weight. For those with severe obesity, annual health-care costs rose 75 percent, or $2,441 per person.”*

Clarian schat dat momenteel 34% van haar werknemers obesitas heeft en dat 26% rookt (Businessweek, McGregor, 2007). Dr. Robert Goulet van Clarian stelt:

“The population of Indiana is one of the un-healthiest populations in the country.” "The ultimate goal is not to collect more money, the ultimate goal is create a healthier population.” (NBC5, 2007)

Volgens Robert Goulet, woordvoerder van Clarian, is het uiteindelijke doel van het preventieplan van Clarian dus niet financieel van aard, maar wil Clarian uiteindelijk een gezondheidsdoelstelling realiseren: *“a healthier population”*.

In de geanalyseerde teksten geeft Clarian de volgende objectieve (gekwantificeerde) informatie over de risico’s op gezondheidsschade die zij met haar preventieplan tracht te bestrijden:

- roken;
- ernstig overgewicht (BMI > 30);
- hoge bloeddruk (bloeddruk > 140/90 mmHg);
- hoog glucosegehalte (glucosegehalte > 120 mg/dL);
- hoog LDL cholesterolgehalte[4] (LDL > 130 mg/ dL).

De gezondheidsschade die Clarian met haar preventieplan tracht te bestrijden, betreft uitsluitend gezondheidsschade aan de persoon met de ongezonde leefstijl zelf. Roken kan in de vorm van passief meeroken weliswaar schade toebrengen aan de gezondheid van derden, maar de preventie van passief meeroken, is geen doelstelling van het preventieplan van Clarian.

Clarian kan niet meer dan een schatting geven van hoe haar werknemers op (enkele van) deze indicatoren scoren (34% obesitas; 26% rookt). Dat betekent dat Clarian niet meer dan een schatting kan geven van de risico’s op gezondheidsschade die haar werknemers lopen. Mogelijk vormt de privacy van de werknemers een belemmerende factor voor het verkrijgen van meer informatie. De bovenstaande indicatoren van ongezondheid leiden statistisch gezien tot een verhoogde zorgconsumptie en daarmee hogere zorgkosten, maar zijn op zichzelf nog geen aandoening (iemand die rookt, hoeft niet ziek te zijn). Voor een hoge bloeddruk, hoog glucosegehalte en hoog cholesterolgehalte geldt dat medicinale behandeling mogelijk is, die weliswaar leidt tot zorgkosten, maar waarmee gezondheidsschade kan worden voorkomen. Mogelijk geven werknemers er de voorkeur aan om bijvoorbeeld een cholesterolremmer of bloeddrukverlager te gebruiken in plaats van gezonder te gaan leven.

4. LDL Cholesterol = Low-Density Lipoprotein Cholesterol = ‘slecht’ cholesterol.

Geen van de teksten geeft informatie over de gezondheidsaandoeningen waaraan de werknemers van Clarian lijden, of over de zorgconsumptie van de werknemers van Clarian. In de teksten wordt alleen gesproken over het afrekenen van de werknemers op basis van de bovengenoemde indicatoren van ongezondheid en wordt een schatting gegeven van de score van de werknemers op enkele van deze indicatoren. In de wetenschappelijke literatuur kan worden opgezocht in welke mate de indicatoren van ongezondheid die Clarian hanteert een risico vormen voor het krijgen van bepaalde aandoeningen (bv. kanker, hart- en vaatziekten, klachten aan het bewegingsapparaat, enz.). In de media blijft Clarian in gebreke m.b.t. het leveren van informatie hierover. Ook journalisten die over het preventieplan van Clarian schrijven, geven deze informatie niet.

Gezondheidsschade kan worden uitgedrukt in een verlies aan QALY's (voor kwaliteit van leven gecorrigeerde levensjaren) of een toename van ziektelast. De kosten hiervan kunnen worden benaderd door de QALY of ziektelast uit te drukken in geld. In geen van de geanalyseerde artikelen wordt op deze manier over de gezondheidsschade gesproken. In de geanalyseerde teksten wordt alleen gesproken over de hogere zorgverzekeringspremies ten gevolge van gezondheidsschade, maar deze betreffen niet de gezondheidsschade zelf, maar financieel-economische schade voor de werkgever ten gevolge van de gezondheidsschade.

Conclusies m.b.t. gezondheidsschade
De gezondheidsschade die Clarian met haar preventieplan tracht te bestrijden, betreft uitsluitend schade aan de persoon met de ongezonde leefstijl zelf. De geanalyseerde teksten geven een duidelijk beeld (gedetailleerd en gekwantificeerd) van welke risico's op gezondheidsschade Clarian wil bestrijden: roken, ernstig overgewicht, hoge bloeddruk, hoog glucosegehalte en hoog LDL cholesterolgehalte. De geanalyseerde teksten geven geen duidelijk beeld van de risico's op gezondheidsschade onder de werknemers van Clarian. Deze onduidelijkheid betreft zowel de aandoeningen waarop zij risico lopen als de omvang van deze risico's. Of Clarian zelf een duidelijke indruk heeft van de risico's op gezondheidsschade onder haar werknemers, kan niet uit de geanalyseerde teksten worden opgemaakt. Mogelijk vormt de privacy van de werknemers een belemmerende factor voor het verkrijgen van meer informatie. De beperkte informatie die Clarian geeft over de gezondheidsschade die zij wil bestrijden is niet onjuist.

Sociaal-economische schade (criterium schadelijkheid)
Voor Clarian vormen de hoge en voortdurend stijgende zorgverzekeringspremies de aanleiding tot het treffen van de preventiemaatregelen:

"Employers generally pay the bulk of health care costs, and Clarian said its health insurance premiums increased 15.7 percent in 2007 and 12.9 percent in 2006." (FoxNews.com, 2007)

Zo bezien is de schade die voor Clarian de aanleiding vormt voor het treffen van preventiemaatregelen primair financieel-economisch van aard. De financieel-economische schade die Clarian met haar preventieplan tracht te bestrijden, betreft uitsluitend schade aan derden (schade aan de financieel-economische belangen van Clarian en van haar werknemers met een gezonde en daardoor 'kostenbesparende' leefstijl). De voortdurend stijgende zorgverzekeringspremies leiden weliswaar ook tot schade aan de financieel-economische belangen van de werknemers met een ongezonde leefstijl, maar de preventie van deze schade is geen doelstelling van het preventieplan van Clarian. Integendeel, Clarian tracht juist een deel van de financieel-economische schade die zij zelf ondervindt op haar werknemers met een 'kostenveroorzakende' leefstijl af te wentelen.

In principe zijn de aard en de omvang van de financiële schade die Clarian met haar preventieplan wil bestrijden, exact vast te stellen. In ieder geval is de omvang van de financiële schade (de kosten van de zorgverzekeringspremies) bij de zorgverzekeraar(s) en bij Clarian bekend. In hoeverre de aard van de financiële schade (de aandoeningen die de zorgkosten van de werknemers van Clarian veroorzaken) bij de zorgverzekeraar(s) en/of bij Clarian bekend is, valt niet uit de geanalyseerde teksten op te maken. Ook over de aard en de omvang van de economische schade die Clarian ondervindt door de hoge zorgverzekeringspremies (in hoeverre worden de concurrentiepositie en winstgevendheid van Clarian bedreigd) geven de geanalyseerde teksten geen nadere informatie.

Naast hogere zorgverzekeringspremies zou een ongezonde leefstijl van werknemers ook kunnen leiden tot productiviteitsverlies (hogere productiekosten) ten gevolge van een verminderde vitaliteit, een hoger ziekteverzuim en meer arbeidsongeschiktheid (Goetzel, 2008, p.305) (Gabel, 2009, p.47). Deze argumenten worden in de geanalyseerde teksten niet genoemd als doelstelling van het preventieplan van Clarian en worden daarom pas later beoordeeld als neveneffecten van het preventieplan bij het 10e criterium 'lasten-batenanalyse'.

Conclusies m.b.t. sociaal-economische schade

De sociaal-economische schade die Clarian met haar preventieplan tracht te bestrijden, betreft financieel-economische schade aan derden. De geanalyseerde teksten geven een duidelijk beeld van de aard van deze schade: voortdurend stijgende zorgverzekeringspremies. De geanalyseerde teksten geven geen duidelijk beeld van de omvang van deze schade. Mogelijk beschikt Clarian over veel (kwan-

titatieve) informatie m.b.t. de omvang van de financieel-economische schade die zij niet openbaar heeft gemaakt. De beperkte informatie die Clarian geeft over de sociaal-economische schade die zij wil bestrijden is niet onjuist.

Inbreuk op het persoonlijk leven (criterium schadelijkheid)
Uit de geanalyseerde teksten komt geen enkele informatie naar voren waaruit zou blijken dat werknemers van Clarian met hun ongezonde leefstijl inbreuk maken op het persoonlijk leven van derden.

Veroorzaken van overlast (criterium schadelijkheid)
Uit de geanalyseerde teksten komt geen enkele informatie naar voren waaruit zou blijken dat werknemers van Clarian met hun ongezonde leefstijl overlast veroorzaken.

Schending van morele opvattingen (criterium schadelijkheid)
Een aantal webloggers ziet in het preventieplan van Clarian een aanpak van het morele verval in de Amerikaanse samenleving. De Amerikaanse waarde van 'eigen verantwoordelijkheid' (voor de eigen gezondheid) is in hun ogen aan verval onderhevig. Het preventieplan van Clarian betekent voor hen een herstel van deze waarde:

> *"The USA is in this fix because we are into instant gratification. We all want what we want and want it now! I think the snowball is going downhill and it is getting bigger and there is no way to stop it. Sad, sad, sad. Everyone wants an easy fix."* (MSNBC, 2007)
>
> *"Responsibility and accountability. Hard to do but it is ultimately what will make us better people and a stronger country."* (MSNBC, 2007)

In de geanalyseerde teksten zijn geen aanwijzingen te vinden dat voor Clarian het hierboven genoemde morele verval aanleiding is tot het treffen van de preventiemaatregelen.

6.1.3 Eindoordeel (criterium schadelijkheid)

De gezondheidsschade die Clarian met haar preventieplan tracht te bestrijden, betreft uitsluitend schade aan de persoon met de ongezonde leefstijl zelf. De beschikbare informatie over de aard van de gezondheidsschade die Clarian tracht te bestrijden is volledig, voldoende gedetailleerd en voldoende gekwantificeerd. De beschikbare informatie over de omvang van de gezondheidsschade die Clarian tracht te bestrijden is onvolledig, onvoldoende gedetailleerd en onvoldoende gekwantificeerd. Mogelijk vormt de privacy van de werknemers een belemme-

rende factor voor het verkrijgen van meer informatie. De beperkte informatie die Clarian geeft over de gezondheidsschade die zij wil bestrijden is niet onjuist.

De sociaal-economische schade die Clarian met haar preventieplan tracht te bestrijden, betreft uitsluitend financieel-economische schade aan derden. De beschikbare informatie over de aard van de financieel-economische schade die Clarian tracht te bestrijden is volledig en voldoende gedetailleerd. De beschikbare informatie over de omvang van de financieel-economische schade die Clarian tracht te bestrijden is onvolledig, onvoldoende gedetailleerd en onvoldoende gekwantificeerd. Er lijken voldoende mogelijkheden te zijn om de omvang van de schade beter in beeld te brengen. Het is onduidelijk in hoeverre Clarian deze informatie achterhoudt of hier niet over beschikt. De beperkte informatie die Clarian geeft over de sociaal-economische schade die zij wil bestrijden, is niet onjuist.

De geanalyseerde teksten bevatten geen aanwijzingen dat Clarian met haar preventieplan ook andere vormen van schade, overlast of schending van morele opvattingen, tracht te bestrijden.

Overzicht 11 laat zien welke schade, overlast en schending van morele opvattingen Clarian met haar preventieplan tracht te bestrijden.

Overzicht 11: risico's die Clarian met het preventieplan tracht te bestrijden

Risicocategorieën	Risico's die Clarian tracht te bestrijden
1. Gezondheidsschade	
– aan zichzelf	ongezondheid van werknemers
– aan derden	
2. Sociaal-economische schade	
– aan zichzelf	
– aan derden	hoge zorgverzekeringspremies
3. Inbreuk op het persoonlijk leven	
4. Overlast	
5. Schending van morele opvattingen	

6.2 Tweede criterium: noodzakelijkheid

6.2.1 Operationalisering en wijze van beoordeling

Operationalisering

Het 2e criterium ‘noodzakelijkheid’ betreft de vraag in hoeverre het noodzakelijk is om de risico’s te bestrijden. Belangrijke aandachtspunten zijn:

- In hoeverre is de preventiemaatregel moralistisch? Tracht men risico’s op schade of overlast te bestrijden? Of tracht men de schending van morele opvattingen te bestrijden (het bestrijden van de schending van morele opvattingen is moralistisch)?
- In hoeverre is de preventiemaatregel perfectionistisch? Is er sprake van een substantieel risico op schade of overlast? Of overdrijft men de risicopreventie?
- Is er sprake van sancties die worden opgelegd aan personen of organisaties die de risico’s (mede) veroorzaken? Deze kunnen een indicatie geven van de noodzakelijkheid van preventie van de betreffende risico’s vanuit de optiek van de sanctionerende partij?
- Wordt met de preventiemaatregel niet een zinvollere doelstelling van het preventiebeleid ‘verdrongen’?

In het algemeen kan worden gesteld dat het treffen van een preventiemaatregel noodzakelijk is, als er sprake is van substantiële schade of overlast voor derden.

Wijze van beoordeling

Bij de beoordeling van een preventiemaatregel aan de hand van het criterium ‘noodzakelijkheid’ wordt beoordeeld of er overtuigend wordt beargumenteerd dat het toepassen van de leefstijlbeïnvloedende preventiemaatregel noodzakelijk is.

6.2.2 Beoordeling van casus Clarian Health aan de hand van criterium ‘noodzakelijkheid’

Twee soorten schade vormen voor Clarian aanleiding om preventiemaatregelen te treffen (zie het criterium ‘schadelijkheid’):

- gezondheidsschade;
- financieel-economische schade.

Voor deze schades tracht ik de noodzakelijkheid van het treffen van preventiemaatregelen te beoordelen.

Gezondheidsschade (criterium noodzakelijkheid)
Bij de analyse van het preventieplan van Clarian aan de hand van het criterium 'schadelijkheid' heb ik geconcludeerd dat de geanalyseerde teksten geen duidelijke beeld geven van de omvang van de gezondheidsschade die de aanleiding vormt voor het preventieplan. Daardoor is onduidelijk in hoeverre het preventieplan van Clarian noodzakelijk is om gezondheidsschade te voorkomen. De gezondheidsschade die Clarian met haar preventieplan tracht te bestrijden, betreft uitsluitend schade aan de persoon met de ongezonde leefstijl zelf (zie overzicht 11).

Financieel-economische schade (criterium noodzakelijkheid)
Uit de geanalyseerde teksten kunnen de volgende argumenten worden gedistilleerd, die aangeven waarom het noodzakelijk is, om preventiemaatregelen te treffen ter voorkoming van financieel-economische schade:
- Door te veel mensen met een ongezonde leefstijl wordt de gezondheidszorg in Amerika onbetaalbaar. Ook mensen met een gezonde leefstijl kunnen hier de dupe van worden.
- Door de hoge kosten van de zorgverzekeringspremies wordt de (internationale) concurrentiepositie van het Amerikaanse bedrijfsleven bedreigd.
- De staat Indiana waarin Clarian Health is gevestigd, behoort tot de staten met de ongezondste bevolking van Amerika. De ongezondheid van de bevolking in Indiana benadeelt de concurrentiepositie van Clarian t.o.v. andere Amerikaanse bedrijven.

Veel webloggers stellen de juistheid van deze argumenten ter discussie. Volgens een aantal webloggers kan Clarian de hoge zorgverzekeringspremies best betalen en wil Clarian alleen aan kostenreductie doen om nog meer winst voor de aandeelhouders te behalen. Anderen stellen dat de directiesalarissen bij Clarian te hoog en de bedrijfshuisvesting van Clarian te luxe zijn. Er zijn ook webloggers die vinden dat de kosten van de zorgverzekeringspremies inderdaad onaanvaardbaar hoog zijn voor Amerikaanse werkgevers. Zij leggen de oorzaak hiervan echter niet bij de ongezonde leefstijl van de Amerikaanse bevolking, maar bij het 'failliet' van het Amerikaanse gezondheidszorgsysteem en pleiten voor een stelselherziening. Wegens het ontbreken van financiële cijfers, is de juistheid van de, deels tegenstrijdige, argumenten die worden aangevoerd, niet te beoordelen. Daardoor blijft het onduidelijk in hoeverre het werkelijk noodzakelijk is om, ter voorkoming van financieel-economische schade, leefstijlbeïnvloedende preventiemaatregelen te treffen.

Is de preventiemaatregel moralistisch?
In de geanalyseerde teksten zijn geen aanwijzingen te vinden dat morele overwegingen voor Clarian aanleiding vormen tot het treffen van de preventiemaatregelen.

Is de preventiemaatregel perfectionistisch?
Volgens diverse webloggers wordt in ieder geval de noodzaak van het treffen van preventiemaatregelen tegen obesitas zwaar overdreven. Zij stellen dit echter in het algemeen en niet m.b.t. de werknemers van Clarian:

> *"… the health risks of being fat have been greatly exaggerated, as have the costs of providing health care to treat the illnesses that are correlated with being fat. The misinformation needs to be fought, and fought hard, because what I see happening in the US is that insurers are using fat people as a scapegoat and a diversion to prevent people from thinking too much about how much money they [the insurance companies] waste."* (Big Fat Blog, 2007)

Een boze weblogger bestempelt het preventieplan van Clarian als oplichterij van een perfectionist:

> *"So money cannot be thier [their] true issue... It has to be control, Some non-smoking, health nazi is pulling the ultimate scam [pull the scam = bedriegen, oplichten] and to cover his or her intentions they are including diabetics and c[h]olesterol patients to boot."* (MSNBC, 2007)

Gesuggereerd wordt dat rokers door Clarian worden behandeld als minderwaardige werknemers (associaties: Nazi – inferieur ras - Untermenschen). In de geanalyseerde teksten zijn voor een dergelijk pervers perfectionisme echter geen aanwijzingen te vinden.

De indicatoren van ongezondheid die Clarian hanteert, zijn op zichzelf niet erg perfectionistisch. Ook met suboptimale waarden (BMI onder 30; bloeddruk onder 140/90; cholesterol onder 130) voldoen werknemers aan de door Clarian gestelde normen. Wat betreft roken hanteert Clarian wel een strenge norm: wel of niet. Clarian hanteert de volgende normen voor ongezondheid:

– BMI > 30 (BMI tussen 18,5 en 25 duidt op normaal gewicht; BMI tussen 25 en 30 duidt op overgewicht; BMI > 30 duidt op ernstig overgewicht)[5];

---

5. *"… total annual healthcare costs per person were similar for normal [BMI 18,5-24,9] and overweight [BMI 25,0-29,9] individuals ($3,254 and $3,201, respectively) … Moderate obesity [BMI 30,0-34,9] … was associated with a $670 increase in costs (to $3,925). Severe obesity [BMI >35,0] ... was associated with a $2,441 increase (to $5,695), as compared with costs for persons of normal weight. ... Moderate and severe obesity were associated with 21% and 75% cost increases, respectively."* (Huse, 2007, p.3)

– bloeddruk > 140/90 mmHg (bloeddruk 120/80 is optimaal);
– glucosepeil > 120 mg/dL (glucosepeil tussen 80 en 120 is normaal; glucosepeil > 120 duidt op hyperglycemie/ ongezond verhoogd bloedsuikerpeil);
– LDL cholesterol > 130 mg/ dL (LDL < 100 is optimaal; LDL tussen 100 en 129 is boven normaal);
– roken (is in tegenstelling tot bijvoorbeeld alcoholgebruik zelfs in de laagste dosering schadelijk voor de gezondheid van de persoon zelf[6] en voor mensen die 'meeroken').

Aan de andere kant bestaat er consensus tussen wetenschappers dat in geval van overgewicht een doelstelling van 10% gewichtsverlies te verkiezen is boven een doelstelling van meer dan 10% gewichtsverlies (Puhl, 2010, p.1021):

> *"… there is recognition in the scientific community that existing dietary programs and medications can produce no more than an average of 10% weight loss. This evidence has prompted agreement among a number of expert panels and scientific groups (including the Institute of Medicine, World Health Organization, Preventive Task Force, Canadian Task Force of Preventive Health Care, and National Heart, Lung, and Blood Institute) that health care providers should counsel patients to set a goal of 10% reduction in total body weight rather than struggle to attain ideal body weight."*

Als 10% gewichtsverlies gemiddeld genomen het maximaal haalbare is, is de streefwaarde die Clarian hanteert van een BMI van 30 of minder voor een deel van de werknemers niet billijk. Clarian negeert deze op wetenschap gebaseerde informatie of is hiermee onbekend. Clarian zou als tegenargument kunnen aanvoeren dat meer dan 10% gewichtsverlies mogelijk wel haalbaar is met de negatieve incentives die Clarian hanteert. De reden van het toepassen van deze negatieve incentives is immers dat Clarian van opvatting is dat de reguliere preventieprogramma's onvoldoende werken. Op basis van dit mogelijke tegenargument beschouw ik de bovengenoemde consensus tussen wetenschappers over een gemiddeld maximaal haalbaar gewichtsverlies van 10% niet als een doorslaggevende falsificatie van de streefwaarde die Clarian hanteert van een BMI van 30 of minder.

Geven sancties een indicatie van de noodzakelijkheid?

> *"Some Clarian employees worry they'll have to pay up to $780 per year for some conditions they may not be able to correct."* (NBC5, 2007).

6. Roken heeft mogelijk een licht kalmerend effect, dat als positief zou kunnen worden bestempeld.

(M.i. bedraagt de boete maximaal $650 per jaar[7]). Dit bedrag van $780 (of $650) is een substantieel bedrag voor werknemers. De hoogte van dit bedrag maakt duidelijk dat het Clarian menens is. Willens en wetens loopt Clarian het risico op negatieve reacties van haar werknemers en in de media. Hieruit kan worden opgemaakt dat Clarian de preventie van de risico's op schade ten gevolge van de ongezonde leefstijl van haar werknemers noodzakelijk acht.

> *"Clarian, an Indiana hospital chain, will dock $5 off worker paychecks for smoking, $10 off for being overweight, and $5 each for high cholesterol, high blood sugar, and high blood pressure."* (MSNBC, 2007).

Als de hoogte van de sanctie een afspiegeling is van de 'noodzakelijkheid', acht Clarian het risico op schade ten gevolge van overgewicht (sanctie $10) twee keer zo groot als het risico op schade ten gevolge van roken (sanctie $5). De ratio achter de hoogte van de verschillende sancties is onduidelijk en wordt in de geanalyseerde teksten niet toegelicht.

> *"The process of determining the optimal incentive amount is an art end a science. Success with incentives requires understanding the culture of a company, the related benefit structure and other aspects of the covered population. There is no easy "one-size-fits-all" solution. To maximize its perceived value, an incentive should be designed so that participants' perceived value is high relative to the employer's actual cost."* (Hall, 2008, p.19)

Wordt een zinvollere doelstelling van het preventiebeleid verdrongen?

In de geanalyseerde teksten zijn geen aanwijzingen te vinden dat de preventiemaatregel ten koste gaat van ander beleid ter preventie van gezondheidsschade of financieel-economische schade.

De Amerikaanse overheid heeft in federale wetgeving vastgelegd, dat werkgevers in geval van een collectieve zorgverzekering alleen premiedifferentiatie op basis van gezondheidsfactoren mogen toepassen, als de zorgverzekering deel uitmaakt van een breder gezondheidsplan dat ook een welzijnsprogramma bevat[8]. Clarian com-

7. Het bedrag van $780 heeft de auteur van het artikel waarschijnlijk gebaseerd op een maximumboete van $30 per loonstrookje (4 boetes van $5 en 1 boete van $10). Werknemers van Clarian ontvangen iedere twee weken een loonstrookje (Wojcik, 2007) en dus 26 loonstrookjes per jaar (26 x $30 = $780). Om te voldoen aan de Amerikaanse antidiscriminatiewetgeving op het gebied van zorgverzekeringen heeft Clarian de boete per werknemer echter gemaximeerd op $25 per loonstrookje (Wojcik, 2007). Dit zou neerkomen op een maximale boete van $650 per jaar (26 x $25 = $650). Zie bijlage 8: toelichting op §f van de HIPAA.
8. Zie bijlage 8: toelichting op §f van de HIPAA.

bineert haar preventieplan met een uitgebreid welzijnsprogramma (Andrews, 2007):

> *"… the company [Clarian] offered free lifestyle and nutrition coaching, stop-smoking programs, fitness centers, and other wellness activities."*

Ik acht het onwaarschijnlijk dat het preventieplan van Clarian geld aan dit welzijnsprogramma onttrekt. Clarian verwacht immers dat de preventiemaatregel het bedrijf geld zal opleveren. Onder druk van het preventieplan zullen werknemers van Clarian waarschijnlijk eerder meer dan minder van het welzijnsprogramma van Clarian gebruik maken, om zodoende boetes voor een slechte score op gezondheidsindicatoren te ontlopen.

6.2.3 Eindoordeel (criterium noodzakelijkheid)

De argumentatie dat het noodzakelijk is om de gezondheidsschade met leefstijlbeïnvloedende preventiemaatregelen te bestrijden, is niet overtuigend. Zwak in de argumentatie is dat de omvang van de risico's op gezondheidsschade bij de werknemers van Clarian niet goed in kaart zijn gebracht en dat de gezondheidsschade geen schade aan derden betreft. Mogelijk vormt de privacy van de werknemers een belemmerende factor voor het verkrijgen van meer informatie.

De argumentatie dat het noodzakelijk is om de financieel-economische schade met leefstijlbeïnvloedende preventiemaatregelen te bestrijden, is enigszins overtuigend. Zwak in de argumentatie is met name dat de omvang van deze schade onvoldoende in kaart is gebracht.

De overwegingen die voor Clarian de aanleiding vormen tot het treffen van de preventiemaatregel zijn ***niet moralistisch***.

Er bestaat consensus tussen wetenschappers dat in geval van overgewicht een doelstelling van maximaal 10% gewichtsverlies de voorkeur verdient. Men kan redeneren dat deze consensus is gebaseerd op een situatie waarin niet wordt gewerkt met negatieve incentives en dat deze dus niet van toepassing is op de preventiemaatregel van Clarian. Zo bezien is de preventiemaatregel van Clarian ***niet perfectionistisch***.

Op basis van de hoogte van financiële sancties die Clarian haar werknemers wil opleggen, kan worden opgemaakt dat Clarian het treffen van de preventiemaatregel zeer noodzakelijk acht.

In de geanalyseerde teksten zijn geen aanwijzingen te vinden dat de preventiemaatregel ten koste gaat van ander beleid ter preventie van gezondheidsschade of financieel-economische schade.

6.3 Derde criterium: oorzakelijkheid

6.3.1 Operationalisering en wijze van beoordeling

Operationalisering

Het criterium 'oorzakelijkheid' betreft de vraag naar de determinanten van de risico's die men met de preventiemaatregel tracht te bestrijden. Achtereenvolgens dienen twee vragen te worden beantwoord:

1. In hoeverre zijn de leefstijlen waarop de preventiemaatregel zich richt bepalend voor de risico's die men met de preventiemaatregel tracht te bestrijden (zie het 1e criterium 'schadelijkheid')?
2. Welke factoren zijn bepalend voor de leefstijlen waarop de preventiemaatregel zich richt en in hoeverre zijn deze factoren bepalend?

Factoren die medebepalend zijn voor een bepaalde leefstijl (2e vraag) zijn bijvoorbeeld:

- persoonlijkheidsfactoren (bv. mate van impulsiviteit, afhankelijkheid, wilskracht);
- gezondheidsfactoren (bv. een bepaalde genetische aanleg, bepaalde aandoeningen);
- sociaal-economische factoren (bv. sociaal-economische status, werkloosheid);
- sociaal-culturele factoren (bv. de eetgewoonten van een bepaalde etnische groep, bij tieners de druk vanuit de 'peergroup' om te roken of alcohol te drinken);
- (overige) omgevingsfactoren (bv. de obesogene omgeving).

M.b.t. de factoren die medebepalend zijn voor een bepaalde leefstijl (2e vraag) kan men oneindig ver terug redeneren door eerst naar directe 'oorzaken' van een bepaalde leefstijl te zoeken, vervolgens naar achterliggende oorzaken, dan naar oorzaken achter deze achterliggende oorzaken, enz. Het aantal achterliggende oorzaken dat wordt gevonden, zal steeds verder toenemen, maar hoe verder de oorzaken naar achter liggen, hoe zwakker het causale verband met de leefstijl zal worden. Bij de analyse aan de hand van het criterium 'oorzakelijkheid' dient om reden van pragmatisme de focus uit te gaan naar de factoren die in grote mate bepalend zijn voor de leefstijl die men met de preventiemaatregel wil beïnvloeden.

In de literatuur wordt gesproken over een keten van oorzaken die leidt tot een leefstijlgerelateerde aandoening. Het woord keten wekt de suggestie dat het gaat om factoren die na elkaar komen, zoals de schakels van een ketting. Regelmatig treedt er echter een wisselwerking op tussen verschillende factoren en zijn bepaalde factoren zowel oorzaak als gevolg in het ontstaan van een leefstijlgerelateerde aandoening. Overgewicht kan er bijvoorbeeld toe leiden dat mensen minder gaan sporten en minder sporten kan leiden tot meer overgewicht. Ik spreek daarom bij voorkeur niet over een 'keten van oorzaken', maar over een ***'complex van factoren'*** dat aan het ontstaan van een leefstijlgerelateerde aandoening ten grondslag ligt.

Wijze van beoordeling
Bij de beoordeling van de preventiemaatregel aan de hand van het criterium 'oorzakelijkheid' wordt beoordeeld of de beschikbare informatie een duidelijk beeld geeft van de determinanten van 'de risico's die men tracht te bestrijden' en hun onderlinge wisselwerking. Is deze informatie juist, volledig, voldoende gedetailleerd en voldoende gekwantificeerd? Als dat oordeel negatief uitvalt, wordt beoordeeld of de informatieverzameling over de oorzakelijkheid voldoende is geweest. Heeft men voldoende gebruik gemaakt van de mogelijkheden om de determinanten van de risico's die men tracht te bestrijden (en hun wisselwerking) te inventariseren en kwantificeren?

6.3.2 Beoordeling van casus Clarian Health aan de hand van criterium 'oorzakelijkheid'

Twee vormen van schade vormen voor Clarian aanleiding om preventiemaatregelen te treffen (zie onder het criterium 'schadelijkheid'):
- gezondheidsschade;
- financieel-economische schade.

Daarom richt de beoordeling van de oorzakelijkheid zich op deze twee vormen van schade.

Vraag 1: in hoeverre zijn de leefstijlen bepalend voor de risico's op schade?

Gezondheidsschade (criterium oorzakelijkheid; vraag 1)
Een score op de indicatoren voor ongezondheid boven de afkappunten die Clarian hanteert (zie §6.2.2), vormt volgens de wetenschappelijke literatuur inderdaad een risico voor de gezondheid. Ook roken is een risico voor de gezondheid. De relatie tussen de leefstijl van de werknemers van Clarian en deze gezondheidsrisico's wordt in de geanalyseerde teksten echter niet toegelicht. Daardoor is het onduide-

lijk of deze gezondheidsrisico's (m.u.v. roken) bij de werknemers van Clarian vooral ontstaan door een ongezond dieet, door te weinig bewegen, of door een combinatie van deze twee. Ook is onduidelijk wat er eventueel mis is met het dieet van de werknemers van Clarian (te veel eten? te veel vet? te veel suiker? te veel zout? te veel alcohol? te weinig groenten en fruit?).

Voor- en tegenstanders van het preventieplan van Clarian debatteren over de vraag of overgewicht en diabetes een gevolg zijn van enerzijds een ongezonde leefstijl of anderzijds een aandoening en/of genetische aanleg. De tegenstanders van het preventieplan van Clarian voeren voorbeelden aan van patiënten waarbij ten onrechte door artsen niet werd onderkend dat hun overgewicht het gevolg was van een aandoening:

> *"There was a story on - I think it was "Mystery Diagnosis" - that ran today, about a woman with Cushing's Syndrome. She KNEW something was wrong but her doctors just kept harping on her to "lose weight, eat less, exercise more." It very nearly killed her … I wonder how many deaths attributed to "obesity" in this country are actually attributable to conditions or syndromes the doctors didn't bother to investigate …".* (Big Fat Blog, 2007)

De voorstanders van het preventieplan van Clarian stellen dat overgewicht ten gevolge van een aandoening statistisch gezien een uitzondering is:

> *"All of the examples you people are throwing out about statistically insignificant populations (hypochondriacs, people who 'can't' lose weight no matter how hard they try because it's ALL the fault of genetics, not their poor choices, etc) are sound-bites but they're not statistically significant."* (MSNBC, 2007)

Het debat over de vraag of overgewicht in het algemeen een gevolg is van enerzijds een ongezonde leefstijl (standpunt Clarian) of anderzijds een aandoening en/of genetische aanleg (standpunt van bepaalde tegenstanders van het preventieplan van Clarian) zie ik als een achterhoedegevecht, dat de tegenstanders van het preventieplan van Clarian gaan verliezen. Hun ongelijk in deze kwestie is met wetenschappelijk onderzoek aan te tonen. Tenzij zij zouden beweren dat de ongezonde leefstijl die tot overgewicht leidt (mede) wordt veroorzaakt door een aandoening en/of genetische aanleg, maar dat moeilijker te weerleggen argument wordt door hen niet aangevoerd.

Een tegenstander van het preventieplan van Clarian stelt dat het niet aan werkgevers is om de oorzaken van het overgewicht van werknemers te beoordelen:

> *"the determination of who is overweight as a result of poor eating habits and lack of exercise, compared to those who have genetic disorders or medical conditions that*

can't be wished away, is something that employers – most of whom are NOT medical professionals – are not qualified to make. . .” (MSNBC, 2007)

Ook dit argument van de tegenstanders van het preventieplan van Clarian is niet houdbaar. Het preventieplan van Clarian biedt werknemers immers de mogelijkheid om met een doktersverklaring aan te tonen dat hun ongezondheid primair het gevolg is van een aandoening en/of genetische aanleg en niet van een ongezonde leefstijl. Het opstellen van een dergelijke doktersverklaring laat Clarian over aan de behandelend arts van de werknemer, die onafhankelijk is van Clarian. Deze mogelijkheid wordt door de tegenstanders van het preventieplan genegeerd.

Financieel-economische schade (criterium oorzakelijkheid; vraag 1)
N.a.v. de aankondiging van het preventieplan van Clarian in de media, bericht FoxNews.com over een statistisch significant verband tussen overgewicht en zorgkosten:

“... a recent study by Thomson Health Care found that workers who were moderately obese had health-care costs that were 21 percent, or $670 a year, higher than workers with normal weight. For those with severe obesity, annual health-care costs rose 75 percent, or $2,441 per person.” (FoxNews.com, 2007)

De tegenstanders van het plan van Clarian stellen dat een hoge BMI geen indicator is van ongezondheid:

“BMI is NOT any kind of indicator of actual health. It is a holdover from the "social darwinism" of the Victorian era. It. Does. Not. Demonstrate. Health. Why don't they just charge "extra" when people actually have to go to the doctor?” (Big Fat Blog, 2007)

Ook dit debat zie ik als een achterhoedegevecht dat de tegenstanders van het preventieplan van Clarian gaan verliezen. Een statistisch significant verband (correlatie) tussen de indicatoren van ongezondheid die Clarian hanteert en ongezondheid is wetenschappelijk aan te tonen. Datzelfde geldt voor een significante correlatie tussen ongezondheid en hogere zorgkosten en tussen de indicatoren van ongezondheid die Clarian hanteert en hogere zorgkosten. Daaruit blijkt dat dit argument van de tegenstanders van het preventieplan van Clarian niet houdbaar is.

Veelvuldig wordt het argument aangevoerd dat de hoge zorgverzekeringspremies voor werkgevers niet worden veroorzaakt door de ongezonde leefstijl van de Amerikanen, maar door het failliet van het private zorgsysteem (zowel de Amerikaanse zorgverzekeraars als zorginstellingen hebben winstoogmerk). Diverse mensen pleiten voor een sociaal zorgsysteem zonder winstoogmerk, uitgevoerd door de overheid, om daarmee de solidariteit tussen zieken en gezonden te herstellen en de

hoge zorgkosten terug te dringen. Anderen bestrijden dat dit een oplossing is. Zij zijn beducht dat een publiek zorgsysteem de toegankelijkheid en keuzevrijheid zal aantasten, of beschuldigen de Amerikaanse artsen en overheid van een ongefundeerde 'vetonvriendelijke' attitude, die door een stelselherziening niet zal veranderen:

> *"And more specific to the problems for fat people - we won't be changing the doctors, we'll be moving the doctors we have to a new system. We won't be changing the system we have for training doctors. We won't be changing the attitudes in this country. Do you think that those attitudes won't affect the way that government system is run? Canada's government is more fat-friendly, so it only makes sense that a government-run health care system in Canada would be more fat-friendly. In this country, it is going the opposite way, which makes me shudder to think what might happen to my health care under a government-run system."* (Big Fat Blog, 2007)

Uit de geanalyseerde teksten wordt niet duidelijk in hoeverre de hoge zorgverzekeringspremies zijn toe te schrijven aan de ongezondheid van de bevolking of aan het Amerikaanse zorgsysteem. Tegenstanders van het preventieplan van Clarian zouden zich m.i. beter kunnen beroepen op het argument dat de oorzaken van een ongezonde leefstijl onvoldoende bekend zijn, maar dit argument wordt opmerkelijk genoeg in geen van de geanalyseerde teksten aangevoerd.

Vraag 2: welke factoren zijn in welke mate bepalend voor de ongezonde leefstijlen?

Het is opvalland dat de oorzaken van een ongezonde leefstijl in de geanalyseerde teksten grotendeels onbesproken blijven. Waarom rookt iemand? Is roken een verslaving? Waarom heeft iemand overgewicht? Is voedsel verslavend? (Sharma, 2009, p.1) Tegenstanders van het preventieplan van Clarian voeren in dit verband alleen het argument aan, dat de hoge werkdruk die werkgevers hun personeel opleggen, ongezondheid veroorzaakt:

> *"… high blood pressure and stress comes with the Job. What will the company pay us for the work schedule 365 days a year snd [and] divorce rate caused by some working enviro[n]ments."* (MSNBC, 2007)

Dit argument bevat wellicht een kern van waarheid, maar wordt door de voorstanders van het preventieplan van Clarian genegeerd. In de wetenschappelijke literatuur wordt de *"obesogene omgeving"* aangewezen als een belangrijke 'veroorzaker' van overgewicht. Daarom is het opmerkelijk dat niemand hierover spreekt.

6.3.3 Eindoordeel (criterium oorzakelijkheid)

Er bestaat duidelijk een oorzakelijk verband tussen de gezondheidsfactoren (roken, hoge BMI, hoge bloeddruk, hoog glucosepeil, hoog LDL cholesterol) waarop de preventiemaatregel zich richt en de risico's op gezondheidsschade en financieel-economische schade die Clarian wil bestrijden. Wat het aandeel is van de verschillende gezondheidsfactoren in het ontstaan van de risico's op gezondheidsschade en financieel-economische schade bij de werknemers van Clarian blijft onduidelijk. Mogelijk vormt de privacy van de werknemers een belemmerende factor voor het verkrijgen van meer informatie.

Clarian lijkt ervan uit te gaan dat werknemers volledig zelf verantwoordelijk zijn voor hun ongezonde leefstijl en negeert wetenschappelijke kennis die een rol toekent aan de genetische constitutie en een grote rol aan de obesogene omgeving. Op dit punt is de informatieverzameling van Clarian zeer onvolledig (Puhl, 2010, p.1021). Mogelijk vormen de instellingen van Clarian Health (ziekenhuisketen) zelf een obesogene omgeving voor de werknemers (Schulte, 2008, p.557-559).

6.4 Vierde criterium: verantwoordelijkheid

6.4.1 Operationalisering en wijze van beoordeling

Operationalisering
Het criterium 'verantwoordelijkheid' betreft de vragen:
1. Welke partijen worden verantwoordelijk gehouden voor het ontstaan en/of de instandhouding van de risico's die men met de preventiemaatregel tracht te bestrijden?
2. Is bij het vaststellen van de verantwoordelijkheid voor het ontstaan van deze risico's wel rekening gehouden met alle bekende oorzaken c.q. determinanten van deze risico's?
3. Is het treffen van de preventiemaatregel niet paternalistisch?

Het antwoord op bovenstaande vragen wordt bepaald door een combinatie van empirische feiten met ideologische opvattingen. De empirische feiten betreffen de determinanten van de risico's die men wil bestrijden (zie het criterium 'oorzakelijkheid'). De ideologische opvattingen betreffen de opvattingen die men heeft over:

- de verantwoordelijkheid van volwassenen en van kinderen voor het gedrag, voor de gezondheid, voor het welzijn en voor de omgeving van zichzelf en van derden (derden kunnen volwassenen en kinderen zijn);
- de verantwoordelijkheid van de overheid voor het gedrag, voor de gezondheid, voor het welzijn en voor de omgeving van volwassenen en van kinderen;
- de verantwoordelijkheid van private partijen (scholen, zorginstellingen, bedrijven)[9] voor het gedrag, voor de gezondheid, voor het welzijn en voor de omgeving van volwassenen en van kinderen;
- de fenomenen 'accumulerende schade' en 'collectieve verantwoordelijkheid' (bv. 'accumulerende schade' ten gevolge van de obesogene omgeving en 'collectieve verantwoordelijkheid' voor het ontstaan en de instandhouding van de obesogene omgeving) (Feinberg, 1984, p.225-232; Kernohan, 1993);
- de (on)wenselijkheid (van varianten) van paternalisme (bv. zacht paternalisme of libertair paternalisme).

De opvatting dat kinderen en psychiatrische patiënten tot op zekere hoogte tegen zichzelf dienen te worden beschermd, is algemeen aanvaard. Als men een verslaving ziet als een psychiatrische aandoening, dienen verslaafden wellicht ook tegen zichzelf te worden beschermd. De verantwoordelijkheid van volwassenen voor het eigen gedrag, wordt meestal gekoppeld aan keuzevrijheid. De vraag of en in hoeverre de mens keuzevrijheid heeft, is al eeuwen onderdeel van wetenschappelijk debat. Empirisch onderzoek heeft nog geen uitsluitsel gegeven (Barry, 2009, p.18-19):

> *"For this study, we examined the role of seven specific metaphors about the causes of obesity, derived from elite discourse and refined using indepth qualitative research methods. These metaphors are (1) obesity as sinful behavior (e.g.,sloth, gluttony); (2) obesity as a disability; (3) obesity as a form of eating disorder; (4) obesity as a food addiction; (5) obesity as a reflection of time crunch; (6) obesity as a consequence of manipulation by commercial interests; and (7) obesity as a result of a toxic food environment."*

De mate waarin mensen (financieel) aansprakelijk worden gesteld voor de kosten van de schade, wordt namelijk niet alleen bepaald door de mate waarin zij verantwoordelijk worden gehouden voor het ontstaan van de schade, maar bijvoorbeeld ook door opvattingen over een rechtvaardige lastenverdeling binnen de samenleving (Kaminisky, 2009, p.529):

9. Maatschappelijk Verantwoord Ondernemen: www.mvonederland.nl

“Attempts to solve the problem of obesity raise controversies regarding individual responsibility and solidarity. The arguments concern which burdens are to be carried privately and which are to be absorbed collectively.“

Een algemeen uitgangspunt zou bijvoorbeeld kunnen zijn, dat de sterkste schouders (werkgevers) de zwaarste (financiële) lasten moeten dragen en dat de zwakste schouders (zieke werknemers) ontzien moeten worden. De rechtvaardigheid van de lastenverdeling komt binnen het model echter pas bij het 9e criterium ‘rechtvaardigheid’ aan de orde. Op basis hievan besluit ik de (financiële) aansprakelijkheid voor de (kosten van) de schade en schadepreventie binnen het model pas bij het 9e criterium ‘rechtvaardigheid’ aan de orde te stellen.

Wijze van beoordeling

- Is duidelijk welke partijen verantwoordelijk worden gehouden voor het ontstaan en/of de instandhouding van de risico’s die men tracht te bestrijden?
- Is bij het vaststellen van de verantwoordelijkheid voor het ontstaan van deze risico’s voldoende rekening gehouden met alle bekende oorzaken c.q. determinanten van deze risico’s?
- Is het treffen van de preventiemaatregel niet (ongewenst) paternalistisch?

6.4.2 Beoordeling van casus Clarian Health aan de hand van criterium ‘verantwoordelijkheid’

Gezondheidsschade (criterium verantwoordelijkheid)

In de geanalyseerde teksten lijken zowel de voor- als de tegenstanders van het preventieplan van Clarian ervan uit te gaan, dat de werknemers van Clarian grotendeels[10] zelf verantwoordelijk zijn voor hun eigen leefstijl (zie onder het criterium ‘oorzakelijkheid’). Als dat het uitgangspunt is, zijn zij in principe ook grotendeels zelf verantwoordelijk voor alle gezondheidsschade ten gevolge van hun ongezonde leefstijl.

Bij het uitgangspunt dat mensen uitsluitend zelf verantwoordelijk zijn voor hun leefstijl, kunnen grote vraagtekens worden geplaatst. Is het wel redelijk en billijk om in een obesogene samenleving uitsluitend het individu verantwoordelijk te stellen voor de gezondheidsschade ten gevolge van overgewicht en daaraan gerelateerde gezondheidsklachten? De voedings- en genotsmiddelenindustrie en Horeca

---

10. Tegenstanders van het preventieplannen van Clarian voeren in dit verband alleen het argument aan, dat de hoge werkdruk die werkgevers hun personeel opleggen, stress en daarmee een hoge bloeddruk veroorzaakt (zie de uitwerking van vraag 2 onder het criterium ‘oorzakelijkheid’).

hebben ongetwijfeld ook een aandeel in het ontstaan of instandhouding van de obesogene omgeving, maar Clarian Health maakt geen onderdeel uit van deze bedrijfstakken. De geanalyseerde teksten bevatten geen aanwijzingen dat de werkomstandigheden bij Clarian in substantiële mate bijdragen aan de risico's op schade die Clarian met haar preventieplan wil bestrijden. Integendeel: Clarian heeft geprobeerd om middels het aanbieden van gezondheidsprogramma's, waaraan werknemers vrijwillig kunnen deelnemen, de gezondheid van haar werknemers te bevorderen. Op basis van de beschikbare informatie ligt het niet voor de hand om Clarian een grote verantwoordelijkheid toe te dichten voor de sanering van de obesogene omgeving. Van een aanbieder van ziekenhuiszorg, zoals Clarian, zou men misschien extra oog voor de gezondheid van de eigen werknemers mogen verwachten. Mogelijk is het preventieplan van Clarian hiervan een uiting.

Een tegenstander van het preventieplan van Clarian stelt dat mensen alleen voor zichzelf en hun familie een verplichting hebben om gezond te blijven en dat zij hierover geen verantwoording verschuldigd zijn aan de samenleving (medeburgers, werkgevers en overheid):

> *"The obligation an American citizen owes the country is to pay taxes, obey all laws, and to serve if legally called upon to do so. Maintaining your health is an obligation to yourself and your family, not to The State. Of course there have been some countries throughout history that required a health standard to be maintained; and we label them T-O-T-A-L-I-T-A-R-I-A-N."* (MSNBC, 2007)

Een woordvoerder van Clarian stelt dat het uiteindelijke doel van het preventieplan van Clarian niet financieel van aard is, maar dat Clarian uiteindelijk een gezondheidsdoelstelling wil realiseren: *"a healthier population"* (zie onder het criterium 'schadelijkheid'). Als dat werkelijk het geval is, en het preventieplan van Clarian vooral bedoeld is voor de bestwil van haar werknemers en de inwoners van Indiana, kan men het preventieplan van Clarian als paternalistisch (betuttelend) kenschetsen.

Financieel-economische schade (criterium verantwoordelijkheid)

Voor- en tegenstanders van het preventieplan van Clarian voeren argumenten aan waarom werknemers al dan niet verantwoordelijk kunnen worden gesteld voor de financieel-economische schade c.q. hogere zorgverzekeringspremies ten gevolge van een ongezonde leefstijl. Ook worden argumenten aangevoerd of het al dan niet terecht is dat Clarian *"agressieve"* preventiemaatregelen ter voorkoming van deze financieel-economische schade treft.

(1) Tegenstanders stellen: Clarian hanteert foutieve indicatoren

Tegenstanders stellen dat het statistisch verband (correlatie) tussen de indicatoren van ongezondheid die Clarian hanteert en extra zorgkosten onvoldoende is aangetoond:

> *"Because, like all conversations about health care, it is based not on fact, but on appearances and false assumptions. They look at us and SEE fat and ASSUME that we're unhealthy because of it."* (Big Fat Blog, 2007)

Zoals besproken onder het 3e criterium 'oorzakelijkheid' is dit argument waarschijnlijk niet houdbaar. Voor alle indicatoren van ongezondheid die Clarian hanteert kan waarschijnlijk een statistisch verband met hogere zorgkosten worden aangetoond.

(2) Tegenstanders stellen: geen oorzakelijk verband tussen leefstijl en indicatoren van ongezondheid

Tegenstanders stellen dat ook mensen met een gezonde leefstijl last kunnen krijgen van obesitas. Ook dit argument is, zoals besproken onder het criterium 'oorzakelijkheid', m.i. niet houdbaar. Enerzijds betreft dit statistisch gezien een uitzondering. Anderzijds kunnen mensen die lijden aan obesitas ten gevolge van genetische aanleg of een aandoening zich met een doktersverklaring van betaling vrijwaren. Wel zou men er bezwaar tegen kunnen maken dat Clarian de bewijslast, dat de ongezondheid van de werknemer niet (primair) het gevolg is van een ongezonde leefstijl, bij de werknemer legt. Bij het ontbreken van een doktersverklaring gaat de werkgever er automatisch vanuit dat de ongezondheid van de werknemer primair een gevolg is van zijn ongezonde leefstijl. Dit zou men kunnen interpreteren als omkering van de bewijslast. Het is de vraag of de behandelend arts van de werknemer en/of de medische beroepsgroep wel aan het opstellen van dergelijke verklaringen willen meewerken en of de werkgever met het opvragen van dergelijke medische verklaringen niet de privacy van de werknemer aantast. Het bezwaar dat Clarian de bewijslast bij de werknemer legt, wordt door de tegenstanders van Clarian echter niet aangevoerd.

(3) Tegenstanders stellen: voor obesitas is geen effectieve therapie beschikbaar

Tegenstanders stellen dat er voor obesitas geen effectieve therapie beschikbaar is (die voor iedereen werkt). Eigenlijk stelt men dus, omdat er geen effectieve therapie tegen obesitas is, kunnen mensen ook niet verantwoordelijk worden gehouden voor de kosten ten gevolge van obesitas. Om dit argument te beoordelen, is het van belang om onderscheid te maken tussen de verantwoordelijkheid voor het ontstaan van obesitas en de verantwoordelijkheid voor het 'genezen' van obesitas (afvallen). In dit verband poneer ik de volgende stellingen:

- Als mensen geen controle hebben over het ontstaan van obesitas en er is geen effectieve therapie beschikbaar, dan kan men hen niet verantwoordelijk houden voor de kosten ten gevolge van obesitas.
- Als mensen geen controle hebben over het ontstaan van obesitas, maar er is wel een effectieve therapie beschikbaar, dan kan men hen wel verantwoordelijk houden voor de kosten van het onnodig voortduren van de obesitas (als zij geen aanvaardbare reden hebben voor het weigeren van de therapie).
- Als mensen wel controle hebben over het ontstaan van obesitas en er is wel een effectieve therapie beschikbaar, dan kan men hen wel verantwoordelijk houden voor de kosten ten gevolge van obesitas (als zij geen aanvaardbare reden hebben voor het weigeren van de therapie).
- Als mensen wel controle hebben over het ontstaan van obesitas, maar er is geen effectieve therapie beschikbaar, dan kan men hen tijdelijk verantwoordelijk houden voor de kosten ten gevolge van obesitas. Iemand levenslang verantwoordelijk houden voor de gevolgschade van een eenmaal gemaakte 'fout', is disproportioneel. Iemand niet verantwoordelijk stellen voor een gemaakte 'fout' is wel heel vrijblijvend en kan de drempel verlagen om deze 'fout' te maken.

In het geval van obesitas is het discutabel of mensen controle hebben over het ontstaan van obesitas en of er een voor iedereen effectieve therapie beschikbaar is. Het lijkt eerder dat mensen enigszins controle hebben over het ontstaan en dat er enigszins effectieve therapieën beschikbaar zijn. De absolute uitspraken van de voorstanders van het preventieplan van Clarian, dat obesitas een gevolg is van luiheid en een gebrek aan wilskracht, passen hier niet bij. Datzelfde geldt voor de absolute uitspraken van de tegenstanders van het preventieplan van Clarian, dat mensen met obesitas niets kunnen doen aan het ontstaan van obesitas, noch aan het 'genezen' van obesitas. Het lijkt mij daarom redelijk om te stellen dat mensen gedeeltelijk verantwoordelijk kunnen worden gehouden voor het ontstaan van obesitas en gedeeltelijk ook voor het voortduren van obesitas. Daarmee zouden zij ook gedeeltelijk verantwoordelijk kunnen worden gesteld voor de zorgkosten die uit obesitas voortvloeien. Overigens geldt m.i. geldt ongeveer hetzelfde voor de zorgkosten die voortvloeien uit roken.

(4) Tegenstanders stellen: het private zorgsysteem veroorzaakt de hoge zorgverzekeringspremies

In de visie van tegenstanders van het preventieplan van Clarian is het falen van het private Amerikaanse zorgstelsel de oorzaak van de uitzonderlijk hoge zorgverzekeringspremies in Amerika. Zij redeneren dat als er geen falend zorgstelsel zou zijn, er geen noodzaak (voor werkgevers) zou bestaan om hoge zorgverzekeringspre-

mies agressief te bestrijden. Daarom is het agressieve preventieplan van Clarian niet gerechtvaardigd, omdat het niet de oorzaken van de hoge zorgverzekeringspremies bestrijdt, maar enkel aan symptoombestrijding doet. M.i. is deze stelling niet houdbaar. Wereldwijd worden hoge zorgkosten en daarmee samenhangende hoge zorgverzekeringspremies agressief bestreden. Alleen worden afhankelijk van de inrichting van het zorgstelsel (marktwerking versus overheidsregulering; privaat versus publiek) en de cultuur van het land andere instrumenten voor de kostenbeheersing ingezet, zoals regulering van de verzekeringsaanspraken, prijsbeleid, aanbodbudgettering, zorgsturing, eigen bijdragen, e.d. Het Amerikaanse zorgstelsel wordt gekenmerkt door relatief veel vrije marktwerking en een grote rol voor private partijen. Voor kostenbeheersing is men daarom in belangrijke mate afhankelijk van de sturingsmogelijkheden van private partijen die een eigen belang vertegenwoordigen, zoals bijvoorbeeld de werkgevers. Zo lang de Amerikaanse samenleving kiest voor de voordelen van een zorgstelsel dat wordt gedomineerd door vrije marktwerking en private partijen, kiest men ook voor de nadelen die hieruit kunnen voortvloeien, zoals private partijen die streven naar eigen winstmaximalisatie in plaats van de overheid die een maximaal presteren van ‘het systeem’ nastreeft. Dat het falende Amerikaanse zorgstelsel mogelijk de grootste veroorzaker van de hoge verzekeringspremies in Amerika is, acht ik daarom geen overtuigend argument tegen de gerechtvaardigdheid van het preventieplan van Clarian. In een privaat zorgstelsel met relatief veel vrije marktwerking moeten private partijen (zoals Clarian) in de gelegenheid worden gesteld om zelf de eigen kosten te bestrijden, want de overheid regelt dat niet voor hen. Het falen van het private Amerikaanse zorgstelsel doet niets af aan de veel hogere zorgverzekeringspremies voor werkgevers van werknemers met een ongezonde leefstijl. Samenvattend zou men de kritiek dat het falende Amerikaanse zorgsysteem leidt tot de hoge zorgverzekeringspremies kunnen zien als een overtuigend argument tegen de huidige inrichting van het Amerikaanse zorgstelsel, maar niet tegen het preventieplan van Clarian, omdat Clarian nu eenmaal binnen de (on)mogelijkheden van dit systeem moet functioneren.

(5) Voor- en tegenstanders stellen: zorgkosten zijn bij werkgevers verkeerd belegd

Tegen- en voorstanders stellen dat de verantwoordelijkheid voor de financiering van zorgkosten bij werkgevers verkeerd is belegd:

> *“The bigger question is: Why do we believe it is the responsibility of businesses to take care of employee health needs? At one time in our country's history, it was relatively easy and low cost for an employer to do this. But today, health insurance costs have risen to the point that they are negatively impacting many companies' survival, particularly small businesses. In many cases, businesses that cannot provide health insurance cannot find good employees, or those that can provide health insurance are*

shifting more and more of the costs onto the employees. American companies should exist to provide a product or service, not exist to provide health care and other social services. It is time for the government to remove this burden from our business community and assume its social responsibility by providing universal health care for ALL citizens. Sure, there will be a price to bear–but hopefully we can disperse the burden among all citizens, rather than lay it solely on the backs of our businesses. And along the way, perhaps do something about controlling skyrocketing health care costs." (MSNBC, 2007)

Zij lijken hiermee te suggereren dat werkgevers tot noodsprongen, zoals het agressieve preventieplan van Clarian, gedwongen worden, omdat de verantwoordelijkheid voor de financiering van gezondheidszorg onterecht bij werkgevers is belegd en bij de overheid thuishoort. Als dit klopt, moet de oplossing voor de hoge zorgverzekeringspremies die werkgevers betalen worden gezocht in een fundamentele stelselherziening. Echter, als men van opvatting is dat de hoge zorgkosten onterecht op werkgevers worden afgewenteld en dit ten koste gaat van de concurrentiepositie van bedrijven, en een fundamentele stelselherziening uitblijft, lijkt het redelijk dat werkgevers een deel van de hoge zorgkosten terugschuiven naar werknemers, met name als er sprake is van vermijdbare financieel-economische schade, bijvoorbeeld ten gevolge van leefstijlgerelateerde aandoeningen. Ik acht het argument dat zorgkosten bij werkgevers zijn belegd daarom geen overtuigend argument tegen de gerechtvaardigdheid van het preventieplan van Clarian.

(6) Voorstanders stellen: werkgevers mogen vermijdbare zorgschade op personeel verhalen

Voorstanders van het preventieplan van Clarian zijn van mening dat als werkgevers 'vrijwillig' aan de zorgkosten van hun personeel meebetalen, zij het recht hebben om onnodige zorgkosten te bestrijden en om mensen zelf te laten opdraaien voor extra zorgkosten ten gevolge van een ongezonde leefstijl (MSNBC, 2007):

"You can cry big brother all you want, but as long as your/my employers are kicking in the majority (in most cases, not all) of the costs of your/my health coverage, they absolutely have a right to demand that we as employees mitigate as many of the PREVENTABLE risks as possible, just as your car insurance company has the right to demand that you drive safely, that you do all you can to protect your car from damage/theft/etc."

Dit argument valt in twee deelargumenten uiteen. Een eerste deelargument is dat als werkgevers vrijwillig bijdragen aan zorgkosten, zij vrij zijn om de hoogte hiervan te bepalen, of dat de hoogte hiervan op zijn minst onderhandelbaar is. Dit eerste deelargument lijkt terecht. Een tweede deelargument is dat zij mogen discrimineren tussen werknemers, wat betreft hun bijdrage aan zorgverzekeringskosten, op basis van leefstijlindicatoren. Dit argument is discutabel, maar binnen nauw-

keurig omschreven randvoorwaarden is deze vorm van discriminatie tussen werknemers toegestaan binnen de Amerikaanse wetgeving (USDL, 2008). Een van deze randvoorwaarden is dat de premiekorting of -toeslag niet meer dan 20% mag bedragen van de totale premie. Blijkbaar acht de Amerikaanse wetgever het redelijk en billijk dat mensen met een ongezonde leefstijl beperkt (maximaal voor 1/5 deel) aansprakelijk worden gesteld voor hogere zorgkosten ten gevolge van een ongezonde leefstijl. Bij het vaststellen van dit maximumpercentage van 20% kunnen ondermeer ook overwegingen van inkomenssolidariteit een rol hebben gespeeld. Uit dit maximumpercentage kan daarom worden afgeleid dat de Amerikaanse wetgever (vermoedelijk) van opvatting is dat mensen met een ongezonde leefstijl op zijn minst voor 1/5 deel verantwoordelijk zijn voor de hogere zorgkosten ten gevolge van een ongezonde leefstijl. Ook kan de conclusie worden getrokken dat de Amerikaanse wetgever, evenals Clarian, een verband ziet tussen:

- een ongezonde leefstijl,
- een slechte score op leefstijlindicatoren,
- hogere zorgkosten,
- een hogere zorgverzekeringspremie.

Voorstanders van het preventieplan van Clarian die van opvatting zijn dat individuen volledig verantwoordelijk zijn voor hogere zorgkosten ten gevolge van een ongezonde leefstijl, gaan volledig voorbij aan de argumenten die hier tegenin kunnen worden gebracht, zoals de invloed van de obesogene omgeving, de onbekendheid met de precieze oorzaken van overgewicht en de afwezigheid van een voor iedereen werkzame therapie. De opvatting dat individuen volledig verantwoordelijk zijn voor de hogere zorgkosten ten gevolge van een ongezonde leefstijl, wordt niet met overtuigende argumenten onderbouwd.

(7) Voorstanders stellen: niet meebetalen aan slechte gewoonten van anderen

Voorstanders van het preventieplan van Clarian zeggen niet mee te willen betalen aan vermijdbare zorgkosten ten gevolge van een ongezonde leefstijl:

> *"I ask no one to pay for my bad habits, grow up and pull your own weight. I dont see any obesity in starving nations. You will feel so much better if you get in shape, mentally, physicaly and you wont be making excuses."* (MSNBC, 2007)

> *"Why should I pay extra for your laziness? Your health habits affect the rates I pay, why should I pay more than my share for your bad habits?"* (MSNBC, 2007)

Zij gaan volledig voorbij aan de oorzaken van een ongezonde leefstijl die buiten de keuzevrijheid van het individu liggen, zoals de obesogene omgeving en de verslavende werking van roken, alcohol en mogelijk ook voedingsmiddelen. Zoals eerder is betoogd, denk ik dat het bij de huidige stand van de wetenschap redelijker is om

mensen gedeeltelijk verantwoordelijk te stellen voor hogere zorgkosten ten gevolge van een ongezonde leefstijl.

Conclusies m.b.t. financieel-economische schade
Samenvattend kunnen op basis van de analyse van de zeven bovenstaande argumenten de volgende conclusies worden getrokken. Het streven van Clarian (de werkgever) om met het preventieplan financieel-economische schade terug te dringen, kan niet als paternalistisch worden bestempeld, want de werknemers brengen met hun ongezonde leefstijl financieel-economische schade toe aan derden. Bij de huidige stand van de wetenschap is er onvoldoende bewijs om werknemers volledig verantwoordelijk te houden voor de financieel-economische schade ten gevolge van een ongezonde leefstijl. De wetenschap draagt juist steeds meer bewijzen aan dat een deel van de oorzaken van bijvoorbeeld obesitas buiten de keuzevrijheid van het individu ligt (denk bijvoorbeeld aan de invloed van de obesogene omgeving en de mogelijk verslavende werking van bepaalde genots- en levensmiddelen). Wetenschappelijk bewijs dat werknemers niets kunnen doen aan hun ongezonde leefstijl ontbreekt echter ook en bovendien druist deze opvatting in tegen ‘het gezonde verstand’. Bij de huidige stand van de wetenschap lijkt het daarom redelijk dat werknemers gedeeltelijk verantwoordelijk worden gehouden voor de financieel-economische schade c.q. hogere zorgverzekeringspremies ten gevolge van een ongezonde leefstijl.

6.4.3 Eindoordeel (criterium verantwoordelijkheid)

De werknemers van Clarian met een ongezonde leefstijl worden volledig verantwoordelijk gehouden voor de gezondheidsschade en financieel-economische schade ten gevolge van hun ongezonde leefstijl. Dit is in strijd met empirisch wetenschappelijke kennis, die voor het ontstaan van overgewicht ook een rol toekent aan de genetische constitutie van mensen en een grote rol aan de obesogene omgeving. Het preventieplan van Clarian bestaat uit een combinatie van het aanbieden van een gezondheidsprogramma (bijvoorbeeld sportfaciliteiten) in combinatie met financiële sancties. Clarian acht de werknemers zelf verantwoordelijk voor het deelnemen aan preventieactiviteiten. Het opleggen van financiële sancties ter voorkoming van gezondheidsschade kan als paternalistisch (betuttelend) worden geïnterpreteerd, omdat de gezondheidsschade die Clarian wil voorkomen geen schade aan derden betreft.[11] Het opleggen van financiële sancties ter voorkoming

11. *“Another argument advanced to justify penalty programs is that, ultimately, their goal is to motivate behavior change that benefits the individual as well as the group. By itself, this “benign paternalism” is a weak justification, easily counterbalanced by the specter of coercion.”* (Pearson, 2009, p.847)

van financieel-economische schade is niet paternalistisch, omdat de financieel-economische schade die Clarian wil voorkomen wel schade aan derden betreft. Hoewel Clarian werknemers volledig verantwoordelijk acht voor hun ongezonde leefstijl, wordt dat niet tot uitdrukking gebracht in de hoogte van de financiële sancties. De federale overheid heeft deze gemaximeerd tot 20% van de zorgverzekeringspremie. Doordat de sancties van Clarian hier binnen blijven, worden werknemers de facto maar voor een beperkt deel verantwoordelijk gesteld voor de financieel-economische schade. Zo bezien zijn de financiële sancties ter bestrijding van de financieel-economische schade, vanuit het perspectief van de verantwoordelijkheidsverdeling, de facto niet ongerechtvaardigd.[12]

6.5 Vijfde criterium: gerichtheid

6.5.1 Operationalisering en wijze van beoordeling

Operationalisering
Het criterium 'gerichtheid' betreft de volgende vragen:

1. Hangt de keuze van de doelgroep en van de leefstijlbeïnvloedende factoren logisch samen met de determinanten van de risico's die men tracht te bestrijden en met de verantwoordelijkheid voor deze risico's?
2. In hoeverre worden personen binnen de doelgroep door de interventie gemist?
3. In hoeverre worden personen buiten de doelgroep door de interventie geraakt?

Ad 1.
De doelgroep van de leefstijlbeïnvloedende interventie definieer ik als de groep personen waarvan men ter voorkoming van leefstijlgerelateerde aandoeningen de leefstijl tracht te beïnvloeden. De keuze voor de doelgroep en/of leefstijlbeïnvloedende factoren waarop de preventieve interventie wordt gericht, dient logisch samen te hangen met de determinanten van de risico's die men tracht te bestrijden (criterium 'oorzakelijkheid') en de verantwoordelijkheid voor deze risico's (criterium 'verantwoordelijkheid').

Ad 2.
Een rookverbod in openbare gebouwen is een voorbeeld van een interventie die alleen de doelgroep (rokers in openbare gebouwen) raakt.

12. *"The primary justification for penalty programs must rest on the economic harms imposed on others when employees engage in unhealthy behavior."* (Pearson, 2009, p.847)

Ad 3.
Een accijnsverhoging op alcohol is een voorbeeld van een interventie die niet alleen de doelgroep (mensen met een (potentieel) alcoholprobleem) raakt, maar ook derden (mensen die geen (potentieel) alcoholprobleem hebben).

Wijze van beoordeling
1. Zijn er voor de keuze van de doelgroep en van de leefstijlbeïnvloedende factoren geen logischer alternatieven mogelijk?
2. Worden onevenredig veel of onnodig veel personen binnen de doelgroep door de interventie gemist?
3. Worden onevenredig veel of onnodig veel personen buiten de doelgroep door de interventie geraakt?

Ad 1.
Het overtuigend beargumenteren van de juistheid van de keuze van de doelgroep en leefstijlbeïnvloedende factoren is moeilijk. Hoe logisch moet een keuze zijn voordat deze gerechtvaardigd is? In het algemeen is het eenvoudiger om vast te stellen dat er geen overtuigende argumenten tegen een stelling of theorie zijn dan overtuigend te beargumenteren dat een stelling of theorie juist is. In dit geval kan dat door te toetsen of er geen logischer alternatieven mogelijk zijn. Met het oog op de uitvoerbaarheid van de toetsing kies ik hiervoor[13].

Ad 2 en 3.
De term ‘onevenredig’ vraagt om een toelichting. Ik doel op onevenredig veel ten opzichte van de omvang van de doelgroep.

6.5.2 Beoordeling van casus Clarian Health aan de hand van criterium ‘gerichtheid’

Vraag 1: Is de keuze van de doelgroep en/of van de leefstijlbeïnvloedende factoren logisch?
De doelgroep van de preventiemaatregel van Clarian wordt gevormd door de werknemers met het risico op hogere zorgkosten ten gevolge van een ongezonde leefstijl. De preventiemaatregel van Clarian is direct op deze doelgroep gericht. Voor een werkgever als Clarian lijkt het gerechtvaardigd om preventiemaatregelen ter voorkoming van hoge zorgverzekeringspremies ten gevolge van een ongezonde

13. Deze wijze van redeneren is afgeleid van het falsificationisme van Karl Popper. Een theorie wordt aangenomen als waar, als de mogelijkheid bestaat om de theorie te falsificeren en de theorie niet gefalsificeerd wordt. Zie §4.3.6.

leefstijl direct op haar eigen werknemers te richten. Op andere oorzakelijke factoren van een ongezonde leefstijl (obesogene omgeving, opvoeding, e.d.) en andere oorzaken van de hoge zorgverzekeringspremies (falend Amerikaans zorgstelsel) kan Clarian geen substantiële invloed uitoefenen.

Vraag 2: In hoeverre worden personen binnen de doelgroep door de interventie gemist?

Niet alle ongezonde of onveilige leefstijlen worden geraakt met het preventieplan van Clarian. Niet geraakt worden bijvoorbeeld ondervoeding (te weinig calorieën, vitaminen of mineralen, e.d.), alcoholmisbruik en onveilige seksuele contacten. Bij de beoordeling in het kader van het 10e criterium 'rechtsgelijkheid' dient hiermee rekening te worden gehouden. Het is een gebrek in de informatie die in de media over het preventieplan van Clarian beschikbaar komt, dat onduidelijk blijft welk percentage van de hoge zorgverzekeringspremies aan de indicatoren van ongezondheid die Clarian hanteert, kan worden toegeschreven.

Vraag 3: In hoeverre worden personen buiten de doelgroep door de interventie geraakt?

Ongezonde mensen die niets aan hun ongezondheid kunnen doen en mensen met een gezonde leefstijl behoren niet tot de doelgroep van het preventieplan van Clarian, maar worden wel door het preventieplan geraakt. Zij moeten immers maandelijks aantonen hoe zij scoren op de gezondheidscriteria die Clarian hanteert. Ten gevolge van het preventieplan moeten zij medische testen ondergaan en wordt mogelijk hun privacy aangetast. Dit is een moeilijk te rechtvaardigen punt van het preventieplan van Clarian, waaraan Clarian tegemoet zou moeten komen door de lasten die de uitvoering van het preventieplan oplevert voor werknemers die buiten de doelgroep vallen, zoveel mogelijk te beperken. Uit de bestudeerde teksten wordt niet duidelijk hoe Clarian hiermee omgaat.

6.5.3 Eindoordeel (criterium gerichtheid)

De gerichtheid van de preventiemaatregel van Clarian op haar eigen werknemers en de 'uitkomstindicatoren' van gezondheid die Clarian hanteert, beoordeel ik als niet onlogisch, d.w.z. dat ik geen logischer alternatieven zie. Ervan uitgaande dat de criteria van ongezondheid die Clarian hanteert niet onlogisch zijn, worden er waarschijnlijk ook niet onevenredig veel of onnodig veel personen binnen de doelgroep (mensen met een ongezonde leefstijl) door de interventie gemist. Wat betreft personen buiten de doelgroep die door de preventiemaatregel worden geraakt, is het beeld minder gunstig. M.i. worden er niet onnodig veel, maar wel onevenredig veel werknemers buiten de doelgroep door de preventiemaatregel van Clarian

geraakt. Het gaat om de werknemers met een gezonde leefstijl, die hun gezondheid keer op keer opnieuw moeten aantonen. M.i. is dit een moeilijk te rechtvaardigen punt van het preventieplan van Clarian, waarvoor zowel bij Clarian, als bij de criticasters van het preventieplan, onvoldoende aandacht is.

6.6 Zesde criterium: geschiktheid

6.6.1 Operationalisering en wijze van beoordeling

Operationalisering

Het criterium 'geschiktheid' betreft de geschiktheid van de partij die de preventiemaatregel uitvoert. Het gaat hierbij om de volgende vragen:

1. Hangt de keuze van de partij die de preventiemaatregel uitvoert logisch samen met de gerichtheid van de preventiemaatregel (doelgroep en leefstijlbeïnvloedende factoren)?
2. Is de partij die de preventiemaatregel uitvoert daartoe bekwaam en bevoegd?
3. Is de partij die de preventiemaatregel uitvoert daartoe geschikt?
4. Brengt uitvoering van de preventiemaatregel schade toe aan de aard of het imago van de partij die de maatregel uitvoert?

Naar aanleiding van de casus Clarian Health heb ik de term 'imago' (risico op imagoschade bij de uitvoerder) aan de vierde vraag toegevoegd.

Versie 2 van het model kende een criterium 'bekwaamheid en bevoegdheid', dat ik had geleend uit de Wet Big. Ten behoeve van het model neem ik het uitgangspunt van de Wet Big over, dat wie bekwaam is daardoor bevoegd is (tenzij er sprake is van een aan een bepaalde partij voorbehouden handeling).

Wijze van beoordeling

1. Is uitvoering van de preventiemaatregel door een andere partij niet logischer gezien de doelgroep en leefstijlbeïnvloedende factoren waarop de maatregel zich richt?
2. Is de partij die de preventiemaatregel uitvoert daartoe niet onbekwaam of onbevoegd?
3. Is de partij die de preventiemaatregel uitvoert daartoe niet ongeschikt?
4. Brengt de preventiemaatregel geen onaanvaardbare schade toe aan de aard of het imago van de partij die de maatregel uitvoert?

Bij de bespreking van de 'wijze van toetsing' van het criterium 'gerichtheid' heb ik gesteld, dat het in het algemeen eenvoudiger is om vast te stellen dat er geen overtuigende argumenten tegen een stelling of theorie zijn, dan overtuigend te beargumenteren dat een stelling of theorie juist is. Van dit principe (afgeleid van het falsificatiebeginsel) maak ik ook gebruik bij de toetsing van de preventiemaatregel aan de hand van het criterium 'geschiktheid'.

6.6.2 Beoordeling van casus Clarian Health aan de hand van criterium 'geschiktheid'

Vraag 1: Is de keuze van de uitvoerende partij logisch, gezien de doelgroep en de leefstijlbeïnvloedende factoren?

De werknemers van Clarian vormen de doelgroep van de preventiemaatregel. Clarian voert de preventiemaatregel uit. Clarian heeft als werkgever groot belang bij de gezondheid van haar werknemers met het oog op de arbeidsproductiviteit en het voorkomen van ziekteverzuim. Bovendien betaalt Clarian als werkgever de premie van het gezondheidsplan (health plan) en de zorgverzekering van haar werknemers. De preventiemaatregel is voornamelijk financieel van aard: het korten op vergoedingen van werknemers als zij niet voldoen aan bepaalde gezondheidscriteria. Inhoudelijk bemoeit Clarian zich er niet direct mee hoe werknemers dit moeten realiseren. Zo bezien is er niet direct een logischer partij dan Clarian mogelijk voor uitvoering van de preventiemaatregel.

Vraag 2: Is de partij die de preventiemaatregel uitvoert daartoe bekwaam en bevoegd?

Critici van het preventieplan van Clarian stellen de bevoegdheid van werkgevers, om te differentiëren in de hoogte van zorgverzekeringspremies op basis van gezond gedrag, ter discussie:

> *"Also, just as safe drivers pay for the actions of unsafe drivers, healthy workers on a group insurance plan pay for the actions of workers who take actions that are likely to damage their health. . . . – Which brings up a possibility: Insurance companies (NOT the employers; they have no right to look at your private medical records) could elect to reward healthier employess with lower rates – similar to the way auto insurers reward accident-free drivers; this might not seem fair to all, but it's a lot more fair than having your employer make a layman's judgment call as to how well you are taking care of yourself."* (MSNBC, 2007)

Op basis van het bovenstaande citaat onderscheid ik m.b.t. de bekwaamheid en bevoegdheid van Clarian twee discussiepunten:

a. In hoeverre is Clarian (zijn werkgevers) bekwaam en bevoegd om te differentiëren in de hoogte van zorgverzekeringspremies op basis van gezond gedrag?

b. In hoeverre is Clarian (zijn werkgevers) bekwaam en bevoegd om gezondheidsinformatie over werknemers te verzamelen en te gebruiken?

Afd. a.
De federale Amerikaanse overheid heeft in 2007 per wet geregeld dat werkgevers mogen differentiëren in de hoogte van zorgverzekeringspremies op basis van (indicatoren van) gezond gedrag:

> *"… regulations that became effective July 1 could prompt cautious employers to step off the sidelines. The federal government recently issued final rules on how wellness programs could comply with the nondiscrimination conditions of the Health Insurance Portability & Accountability Act (HIPAA)."* (BusinessWeek, McGregor, 2007)

Blijkbaar acht de federale overheid werkgevers bekwaam om te differentiëren in de hoogte van zorgverzekeringspremies op basis van gezond gedrag, want het ligt niet voor de hand dat zij bevoegdheden toekent aan onbekwame partijen. Het rationeel differentiëren in de hoogte van zorgverzekeringspremies op basis van (indicatoren van) gezond gedrag lijkt ook mij niet zo ingewikkeld dat werkgevers op dit punt evident onbekwaam zijn.

Afd. b.
Een grote witte vlek in de door mij bestudeerde publicaties (zie hoofdstuk 5) over het preventieplan van Clarian betreft de verzameling, de opslag en het gebruik van de gezondheidsinformatie door Clarian:
- Door wie, hoe en hoe vaak wordt vastgesteld hoe de werknemers van Clarian op de door Clarian vastgestelde gezondheidsindicatoren (roken, BMI, bloeddruk, glucosepeil en cholesterolpeil) scoren?
- Wie krijgen wel en geen inzage in deze informatie?
- Hoe en hoe lang bewaart en beschermt Clarian deze informatie (geheimhouding)?
- Waartoe kan en mag Clarian deze informatie wel en niet gebruiken?

M.i. is het zorgelijk dat in de media over deze vragen nauwelijks informatie beschikbaar is. In Workforce Management spreekt Wojcik over een jaarlijkse screening (2007):

> *"Following the annual assessment, employees who can later prove they have made changes to meet the standards can appeal to have the fees dropped."*

Door dit gebrek aan informatie kan niet worden beoordeeld of de verzameling, de opslag en het gebruik van gezondheidsinformatie door Clarian adequaat wordt

geregeld. Werkgevers zijn m.i. niet evident onbekwaam in de omgang met gezondheidsinformatie. Eventuele aan hulpverleners voorbehouden handelingen dienen aan hen te worden uitbesteed.

Vraag 3: Is de partij die de preventiemaatregel uitvoert daartoe geschikt?
De discussiepunten die ik heb besproken bij de beoordeling van de bekwaamheid en bevoegdheid van Clarian zijn ook relevant voor de beoordeling van de geschiktheid:

a. In hoeverre is Clarian (zijn werkgevers) geschikt om te differentiëren in de hoogte van zorgverzekeringspremies op basis van gezond gedrag?
b. In hoeverre is Clarian (zijn werkgevers) geschikt om gezondheidsinformatie over werknemers te verzamelen en te gebruiken?

Afd. a.
Het differentiëren in de hoogte van zorgverzekeringspremies op basis van gezond gedrag door werkgevers vind ik een heikel punt. Wanneer men dergelijke bevoegdheden toekent aan werkgevers (private partijen), liggen rechtsongelijkheid tussen werknemers van verschillende bedrijven en willekeur op de loer. In Amerika heeft de federale regering er tot mijn verbazing toe besloten om dit toe te staan. Het risico op rechtsongelijkheid en willekeur is in omvang begrensd, doordat de premieverschillen tussen werknemers op basis van gezondheidsverschillen volgens de wetgeving (HIPAA) niet meer dan 20% van de totale premie mogen bedragen. Ik vind het een opvallende omissie in de Amerikaanse wetgeving (HIPAA), dat de wetgever uitsluitend oog lijkt te hebben gehad voor het beperken van discriminatie tussen werknemers en niet voor de bemoeienis van werkgevers met het privéleven (het gezondheidsgedrag) van hun werknemers. Een criticus van het preventieplan van Clarian merkt in dit verband op:

> *"If we as Americans start letting the big name corporations tell us how we can and can't live then I think we have lost the battle to control our own destiny."* (MSNBC, 2007)

Het lijkt mij ongewenst dat werkgevers (private partijen) substantieel invloed uitoefenen op de invulling van het privéleven van hun werknemers wegens het gebrek aan democratische besluitvorming en controle. Als er wordt besloten tot premiedifferentiatie in de basiszorgverzekering op basis van gezondheidsverschillen lijkt mij dat een bevoegdheid van de nationale overheid (in Amerika wellicht de federale overheid) en niet van private partijen of decentrale overheden; dit ter voorkoming van rechtsongelijkheid tussen burgers en van willekeur. Volgens Ann Rives hebben zowel werkgevers als werknemers hier belang bij. Zo bezien zou het beter

zijn als de nationale overheid zou beslissen over de vormgeving van de preventiemaatregel en de werkgevers zich zouden beperken tot de uitvoering (Rives, 2006):

> "*This Note addresses the need for federal legislation, which would curtail private employers' ability to interfere in the off-duty conduct of their employees and thereby keep employers from infringing on their employees' privacy rights. Specifically, this legislation would protect an employee's right to engage in lawful recreational activities as well as an employee's right to lawfully use lawful products during nonworking hours. The legislation would also serve the additional purpose of standardizing employee privacy rights across state lines.*" (p.554)

> "*As evidenced by the example of the drug testing cases, different states have different privacy expectations, and relying on individual state courts to interpret privacy would result in employee privacy rights that varied depending on the state in question. At the same time, in light of economic globalization, employee protections provided by individual state statutes are no longer adequate. The requirements of multi-state employers become muddled as they must adapt their employment policies depending on the state in which they seek job applicants. An employee who lives in Michigan can be fired for smoking off duty, but if that same employee changes his residence to New York then he also changes his rights. Both employees and employers are entitled to some certainty as to what off-duty conduct will be protected; this can be accomplished by a clearly worded and unambiguous federal statute.*" (p.563-564)

Afd. b.

Werkgevers zijn eigenlijk geen geschikte partij om gezondheidsinformatie over werknemers te verzamelen, omdat de werknemer in een afhankelijke positie verkeert t.o.v. de werkgever. De werkgever zou de informatie kunnen misbruiken en het is moeilijk controleerbaar of dit al dan niet gebeurt. De werkgever kan direct of indirect inzicht krijgen in de gezondheid van de individuele werknemer zonder dat deze daarvoor toestemming heeft gegeven: direct, door de verzamelde gezondheidsinformatie te analyseren, en indirect, door kennis te nemen van de hoogte van de 'ongezondheidsboete' per werknemer. Het lijkt mij daarom logischer om de uitvoering van de preventiemaatregel bij zorgverzekeraars te leggen van wie werknemers veel minder afhankelijk zijn (zie het citaat onder vraag 2: "*Also, just as safe drivers pay …*"). Zorgverzekeraars kunnen dan geanonimiseerde informatie aan de werkgever verstrekken. In dit verband doet Michelle Mello de volgende aanbeveling voor het ontwerp van "*wellness incentive programs*" van werkgevers (Mello, 2008, p.197):

> "*Retain a third party to manage medical information and maintain a firewall between that information and the employer.*"

Pearson verwoordt dit als volgt (Pearson, 2009, p.851):

> *"Health records and information should be accessible to only third-party health plan administrators or other outside vendors authorized by the employee. … Employers in penalty programs will not be able to access this information or discriminate against employees on their health records."*

Vraag 4: Brengt uitvoering van de preventiemaatregel schade toe aan de aard of het imago van de partij die de maatregel uitvoert?

Worden de aard of het imago van Clarian als werkgever en ziekenhuisketen niet aangetast? Een nauwe betrokkenheid van Clarian Health bij de gezondheid van haar werknemers past bij de aard van Clarian als aanbieder van gezondheidszorg (ziekenhuisketen). Door het treffen van de preventiemaatregel kan Clarian als werkgever en als ziekenhuisketen echter imagoschade oplopen. Een belangrijke oorzaak is dat er niet democratisch (door de overheid) over de toepassing van de maatregel is besloten en dat andere werkgevers de maatregel niet toepassen of deze zelfs bekritiseren:

> *"Stuart Slutzky, vice president of product development at Destiny Health, a Chicago-based consumer-driven health care company, maintains incentives are a better way for employers to go. He says that in the last year, he has begun to see employers use both the carrot and the stick."* (Workforce Management, Wojcik, 2007)

Ook burgers, ongezond en gezond, bekritiseren de maatregel:

> *"So here's my question: who do we take this up with? Clearly, this action needs to be protested. So where do I fire off my angry letters? The ACLU [American Civil Liberties Union]? A consumer protection agency? …."* (Big Fat Blog, 2007)

Ongezonde en gezonde werknemers kunnen Clarian als werkgever gaan mijden. Patiënten kunnen Clarian als ziekenhuisketen gaan mijden. Clarian kan hier echter ook baat bij hebben, doordat er een vorm van risicoselectie kan optreden. Door de preventiemaatregel zou Clarian vooral werknemers en patiënten met een gezonde leefstijl kunnen aantrekken. Daar Clarian zelf tot het treffen van de preventiemaatregel heeft besloten en deze niet is opgedrongen, is de mogelijke (imago)beschadiging van Clarian geen reden om de maatregel als ongerechtvaardigd te beoordelen.

6.6.3 Eindoordeel (criterium geschiktheid)

Gezien de doelstelling van de preventiemaatregel, het bevorderen van de gezondheid en terugdringen van de zorgkosten van werknemers, is het niet onlogisch dat de werkgever een hoofdrol speelt in de uitvoering van de preventiemaatregel. Werkgevers zijn ***wel bekwaam en bevoegd*** om de preventiemaatregel uit te voeren

(zie het antwoord op vraag 2), ***maar*** in mijn ogen ***ongeschikt*** om de preventiemaatregel uit te voeren (zie het antwoord op vraag 3). Ter voorkoming van rechtsongelijkheid tussen werknemers van verschillende werkgevers en willekeur van de werkgever zou het m.i. beter zijn als de democratisch gekozen rijksoverheid zou beslissen over de vormgeving van de preventiemaatregel (zie het antwoord op vraag 3a). De uitvoering van de preventiemaatregel zouden werkgevers beter kunnen overlaten aan zorgverzekeraars (zie het antwoord op vraag 3b).

6.7 Eindoordeel over de 1e zeef: logica van het ontwerp

Clarian legitimeert haar preventiemaatregel door te wijzen op de gezondheidsschade en financieel-economische schade die zij tracht terug te dringen. De legitimering op basis van gezondheidsschade is niet perfectionistisch, maar wel paternalistisch en daarom niet overtuigend. De legitimering op basis van financieel-economische schade is niet perfectionistisch en niet paternalistisch en zou daarom overtuigend kunnen zijn. Om overtuigend te zijn dient Clarian meer kwantitatieve informatie te verschaffen over de financieel-economische schade die zij wil bestrijden en de noodzaak van de bestrijding van deze schade voor haar bedrijfsvoering.

Een zwak punt van het preventieplan is dat onevenredig veel werknemers buiten de doelgroep door het preventieplan worden geraakt, doordat jaarlijks hun score op alle gezondheidsindicatoren dient te worden vastgesteld. Omdat ik hiervoor geen beter alternatief zie, is dit geen reden om het ontwerp van het preventieplan als onlogisch te bestempelen. Pas bij het criterium 'indringendheid' (8e criterium) wordt vastgesteld, of dit zo bezwaarlijk (belastend/ indringend) is, dat toepassing van de preventiemaatregel ongerechtvaardigd is.

De belangrijkste kritiek op de logica van het ontwerp van de preventiemaatregel is te vinden onder het criterium 'geschiktheid'. Clarian c.q. werkgevers zijn in mijn ogen ongeschikt om de preventiemaatregel te ontwerpen en uit te voeren. Ter voorkoming van rechtsongelijkheid tussen werknemers van verschillende werkgevers en willekeur van de werkgever zou het m.i. beter zijn als de democratisch gekozen rijksoverheid zou beslissen over de vormgeving van de preventiemaatregel. De uitvoering van de preventiemaatregel kan beter worden overgelaten aan zorgverzekeraars. Werknemers zijn minder afhankelijk van hun zorgverzekeraar dan van hun werkgever. Dat geldt zeker voor de Nederlandse situatie, die een acceptatieplicht voor de Zorgverzekeringswet (Zvw) kent. In Nederland kunnen burgers c.q. werknemers wat betreft de Zvw eenvoudig van zorgverzekeraar wisselen, bijvoorbeeld als het premiebeleid van de zorgverzekeraar hun niet bevalt.

Omdat ik werkgevers zowel ongeschikt acht om te besluiten over de vormgeving van de preventiemaatregel als om de preventiemaatregel uit te voeren, verdient de preventiemaatregel in de huidige vorm geen navolging in Nederland. Zo bezien kan de preventiemaatregel van Clarian de eerste zeef van het model ('logica van het ontwerp') niet passeren.

Volgens de Amerikaanse wetgeving mogen werkgevers maximaal 20% premiekorting aan individuele werknemers toekennen op basis van gezondheidsfactoren (USDL, 2008). Als men dit democratisch genomen besluit van de Amerikaanse federale overheid als uitgangspunt neemt, kan de preventiemaatregel de eerste zeef van het model wel passeren, mits:

- de noodzaak van het treffen van de preventiemaatregel beter wordt onderbouwd (met name door de aard en omvang van de financiële schade beter te onderbouwen);
- het verzamelen, analyseren en opslaan van gezondheidsinformatie wordt uitbesteed aan een onafhankelijke derde partij.

7 Toetsing 2e zeef van het beoordelingsmodel aan casus 'Clarian Health' (versie 6 van het model)

In dit hoofdstuk beoordeel ik de gerechtvaardigdheid van het preventieplan van Clarian Health aan de hand van de criteria van de 2e zeef van het model (versie 6).

De 2e zeef 'effecten en neveneffecten' is samengesteld uit de criteria:
- effectiviteit;
- indringendheid;
- kosteneffectiviteit;
- lasten-batenverhouding;
- rechtvaardigheid;
- rechtsgelijkheid.

7.1 Zevende criterium: effectiviteit

7.1.1 *Operationalisering en wijze van beoordeling*

Operationalisering
De effectiviteit betreft de beoogde effecten van de preventiemaatregel op de doelgroep, waarbij de doelgroep is gedefinieerd als de groep personen waarvan men, ter voorkoming van leefstijlgerelateerde aandoeningen, de leefstijl tracht te beïnvloeden. Het is van belang om hierbij te onderscheiden voor welke risico's de preventie effectief is en of mensen met hun leefstijl zichzelf of anderen benadelen (zie overzicht 11 in §6.1). De effectiviteit kan kwantitatief worden uitgedrukt in bijvoorbeeld een toename van QALY's, DALY's of een reductie van ziektelast. Positieve (en negatieve) neveneffecten van de preventiemaatregel vallen niet onder het criterium 'effectiviteit', maar onder het criterium 'lasten-batenverhouding'.

Een belangrijk aandachtspunt is of de effectiviteit wordt bepaald aan de hand van proces- of uitkomstindicatoren. Procesindicatoren betreffen (positieve) veranderingen in het gedrag van de doelgroep. Bijvoorbeeld: mensen uit de doelgroep gaan vaker naar de sportschool, gaan vaker op de weegstaal staan, snoepen minder, enz. Het bezwaar is dat procesindicatoren slechts een indicatie geven van de beoogde (gezondheids)effecten. Mensen gaan weliswaar vaker naar de sportschool,

maar kletsen er misschien meer dan dat zij sporten. Ook kunnen substitutie-effecten optreden. Mensen gaan meer sporten, maar vergroten hun energie-inname navenant, zodat ze niet afvallen. Een voordeel van uitkomstindicatoren is dat de effectiviteit direct wordt gemeten. Mensen worden niet beloond als zij meer sporten, maar alleen als zij afvallen. Uiteindelijk kan dat mensen ook ontmoedigen en eerder doen afhaken. Dit laatste argument tegen uitkomstindicatoren acht ik echter niet doorslaggevend. De grote kracht en tegelijkertijd het grote bezwaar van uitkomstindicatoren is dat ze zo indringend zijn. In tegenstelling tot procesindicatoren bestrijken ze het hele leven (als bijvoorbeeld iemand de hele dag zijn best heeft gedaan om af te vallen, maar zich een uur voor het naar bed gaan overeet, is er toch sprake van een 'verloren' dag) en zijn ze niet slechts een momentopname. Als mensen worden beloond als ze naar de sportschool gaan, worden ze alleen op het moment dat ze naar de sportschool gaan in hun vrijheid beknot. Daarbuiten kunnen ze doen en laten wat ze willen. Als mensen worden beloond als ze afvallen, is hun gedrag gedurende de hele dag bepalend voor de beloning. Dat is wellicht effectiever, maar ook veel indringender.

Ten slotte is ook de duurzaamheid van de effecten een belangrijk aandachtspunt (Puhl, 2010, p.1021):

> *"… experts in the obesity field have concluded that weight regain occurs in practically all dietary and behavioral interventions, and other researchers have asserted that dieters who manage to sustain a weight loss are the rare exception, rather than the rule. Dieters who gain back more weight than they lost may very well be the norm, rather than an unlucky minority."*

Ook kan men zich de vraag stellen of financiële incentives, zoals in het preventieplan van Clarian, op den duur niet hun kracht verliezen (Mello, 2008, p.198):

> *"Of particular interest is whether the lifestyle changes and health-risk reductions that wellness programs inspire are sustained over time. Behavioral science literature suggests that although incentives may induce behavior change in the short term, in some cases they may also dampen intrinsic motivation, with negative long-term consequences for behavior."*

Wijze van beoordeling

Bij de toetsing van de preventiemaatregel aan de hand van het criterium 'effectiviteit' wordt beoordeeld of de preventiemaatregel effectief is in de beoogde risicobestrijding en of deze effecten duurzaam zijn. Is de informatie over de effectiviteit volledig, voldoende gedetailleerd en voldoende gekwantificeerd? Als dat oordeel negatief uitvalt, wordt beoordeeld of de informatieverzameling over de effectiviteit voldoende is geweest.

7.1.2 Beoordeling van casus Clarian Health aan de hand van criterium 'effectiviteit'

Twee vormen van schade vormen voor Clarian aanleiding om preventiemaatregelen te treffen (zie onder het criterium 'schadelijkheid'):
- gezondheidsschade;
- financieel-economische schade.

Daarom richt de beoordeling van de effectiviteit zich op deze twee vormen van schade.

Casus Clarian Health

Gezondheidsschade (criterium effectiviteit)

Een woordvoerder van Clarian stelt:

> *"We believe that by using this premium charge approach, where it shows up in employees' paychecks every two weeks, it will keep [costs] in front of the person, and we think it will be more effective at creating behavior change. It seems that a charge gets people's attention more readily than an incentive."* (Workforce Management, Wojcik, 2007)

Een alternatief is om werknemers te belonen voor gezond gedrag, in plaats van te straffen voor ongezond gedrag, zoals Clarian van plan is:

> *"Big companies like Dell, Kellogg's, IBM and Pitney Bowes now provide rewards, not penalties, for workers who participate in health and wellness programs. At Pitney Bowes, employees get up to $225 a year for doing things like exercising, eating healthy food, and not smoking."* (NBC5, 2007)

Een woordvoerder van Pitney Bowes stelt:

> *"We believe from a lot of testing over the last 15 years that the carrot is far more effective than the stick."* (NBC5, 2007)

In twee van de bovenstaande citaten heb ik het woord *"believe"* onderstreept, om daarmee aan te geven dat de keuze voor belonen en/of straffen meer op overtuiging dan op wetenschappelijk onderzoek lijkt te berusten. De geciteerde woordvoerder van Pitney Bowes spreekt weliswaar over *"a lot of testing over the last 15 years"*, maar aangezien Clarian volgens de geanalyseerde teksten de eerste werkgever is die van belonen overgaat op de combinatie van belonen en straffen, lijkt het mij onwaarschijnlijk dat met empirisch onderzoek is aangetoond dat belonen

in deze context veel effectiever is dan straffen[1]. Ook dient men te bedenken dat, zelfs als belonen veel effectiever is dan straffen, de combinatie van belonen en straffen nog steeds (veel) effectiever kan zijn dan enkel belonen.

Een woordvoerder van Clarian stelt dat belonen (een financiële tegemoetkoming) voor gezond gedrag onvoldoende leidt tot een gezondere leefstijl:

> *"We didn't just decide this. We have five years of wellness program history that included health risk appraisals, health coaching and other resources to help people reduce their risk factors."* (NBC5, 2007)

> *"But even with the wellness initiatives, Clarian's annual health benefit costs continued to climb, growing 12.9 percent in 2006 and 15.7 percent in 2007."* (NBC5, 2007)

Als bewijs voor het niet werken van de 'welness programs' worden de jaarlijks groeiende zorgkosten aangevoerd, maar onduidelijk blijft wat het effect van de *"wellness programs"* op de leefstijl van de werknemers van Clarian is geweest.

Een (waarschijnlijk dikke) weblogger op het Big Fat Blog vraagt zich af (2007):

> *"Is this what will finally moblize us? The fact that these companies are coming after our paycheck?"*

Ik denk dat een dergelijk vraag over de effectiviteit op dit moment het meest op zijn plaats is, omdat niemand lijkt te weten wat de effecten van het preventieplan van Clarian op de leefstijl en gezondheid van de werknemers van Clarian zullen zijn. Wat betreft het effect van het preventieplan van Clarian op de gezondheid van haar werknemers, kan het preventieplan van Clarian het best worden gezien als een eerste experiment, dat afhankelijk van de resultaten meer of minder navolging zal krijgen. De beoogde gezondheidswinst is door Clarian niet gekwantificeerd.

Toch is er wel enig wetenschappelijk bewijs dat financiële incentives gewichtsverlies kunnen bevorderen en dat financiële incentives effectiever zijn naarmate zij hoger zijn:

> *"To test the ability of two levels of modest financial incentives to encourage weight loss among overweight employees. Methods: This study used a randomized design with measurements at baseline, 3, and 6 months and two levels of financial incentives ($7 and $14 per percentage point of weight lost). … Results: At 3 months, partici-*

1. *"Psychological literature offers a variety of opinions on the most effective ways to motivate individuals, but most experts agree that pleasure or reward is a more effective long-term motivator than fear or punishment."* (Hall, 2008, p.15)

pants with no financial incentive lost 2 pounds, those in the $7 group lost approximately 3 pounds, and those in the $14 group lost 4.7 pounds. Between baseline and 6 months, when the financial gains were equalized, weight losses were similar across groups. Conclusion: This study revealed that modest financial incentives can be effective in motivating overweight employees to lose weight." (Finkelstein, 2007, p.981)

"In WHP [work site health promotion] programs, incentives have been offered for participation, compliance with behavior change recommendations, or achievement of certain health goals. Researchers have observed that an incentive valued at ~ $100 (in 2006 dollars) is necessary to encourage the majority of employees to complete an HRA [health risk appraisal]. … Anderson presented preliminary data at a recent conference showing that increasing incentives (typically through reductions in medical premiums) at $100 intervals (from a base of $100 in 2007 dollars) will result in incremental 10% improvements in HRA and program participation." (Goetzel, 2008, p.314)

Financieel-economische schade (criterium effectiviteit)

Voor zover het preventieplan van Clarian ertoe leidt dat werknemers een groter deel van hun zorgverzekeringspremie zelf gaan betalen, neemt de financieel-economische schade voor Clarian af. Om te voldoen aan de Amerikaanse anti-discriminatiewetgeving heeft Clarian de 'boete' voor werknemers in het kader van haar preventieplan gemaximeerd op $25 per tweewekelijks loonstrookje en daarmee op $650 per jaar[2]. Clarian kwantificeert niet welke reductie van zorgverzekeringskosten zij met haar preventieplan nastreeft. De effectiviteit van het preventieplan van Clarian zou men ook relatief kunnen bezien. Als de zorgverzekeringspremies van de werknemers van Clarian na invoering van het preventieplan minder hard stijgen dan de zorgverzekeringspremies van de werknemers van andere bedrijven, kan men ook van een positief effect spreken.

Proces- of uitkomstindicatoren

Clarian hanteert uitsluitend uitkomstindicatoren. Deze gelden voor de hele doelgroep. Ervan uitgaande dat de meeste werknemers het te duur vinden om niet aan het preventieplan deel te nemen, betekent dit dat de effectiviteit in ieder geval goed gemonitord kan worden en dat de druk op werknemers om positieve resultaten (effecten) te behalen, groot is. Het kan er echter ook toe leiden dat mensen die denken toch niet aan de criteria te kunnen voldoen, volledig afhaken.

2. Zie bijlage 9: toelichting op §f van HIPAA.

Duurzaamheid van de effecten
Vanuit het perspectief van duurzaamheid van de effecten is het een sterk punt van het preventieplan van Clarian dat de hoge negatieve financiële incentives blijvend zijn. De kans op terugval in ongezond gedrag lijkt hierdoor minder groot dan wanneer de incentives tijdelijk zouden worden toegepast.

7.1.3 Eindoordeel (criterium effectiviteit)

De effectiviteit van het preventieplan van Clarian als instrument ter preventie van gezondheidsschade van haar werknemers is onduidelijk. Een zwak punt is dat Clarian op het gebied van gezondheidswinst geen (openbare) kwantitatieve doelstellingen heeft geformuleerd aan de hand waarvan de effectiviteit kan worden getoetst. Als de effectiviteit van een preventiemaatregel nog onduidelijk is, is dat toch het minste wat men zou kunnen doen. De effectiviteit van het preventieplan van Clarian als instrument ter preventie van financieel-economische schade is evident voor zover het de extra eigen bijdragen van haar werknemers aan hun zorgverzekeringspremie betreft. In hoeverre het preventieplan van Clarian (op termijn) een gunstige invloed heeft op de hoogte van de zorgverzekeringspremies van haar werknemers, is nog onduidelijk. Een zwak punt is dat Clarian m.b.t. de ontwikkeling van de premiehoogte geen (openbare) kwantitatieve doelstellingen heeft geformuleerd, aan de hand waarvan de effectiviteit van haar preventieplan kan worden getoetst.

Volgens Hall is de werking van incentives contextafhankelijk (Hall, 2008, p.19):

> *"The process of determining the optimal incentive amount is an art end [and] a science. Success with incentives requires understanding the culture of a company, the related benefit structure and other aspects of the covered population. There is no easy "one-size-fits-all" solution. To maximize its perceived value, an incentive should be designed so that participants' perceived value is high relative to the employer's actual cost."*

Daar Clarian een van de eerste werkgevers is die voornemens is om hoge negatieve financiële incentives toe te passen, kan men niet van Clarian verwachten dat de effectiviteit van tevoren bekend is. Men zou van Clarian kunnen vragen om de effectiviteit te evalueren en met het toepassen van hoge negatieve financiële incentives te stoppen, als deze niet effectief blijken. Als van tevoren bekend is dat Clarian stopt met het toepassen van hoge negatieve financiële incentives als werknemers zich hier niets van aantrekken, kan dat echter een negatieve invloed hebben op de effectiviteit.

Alles overziend kom ik tot de slotsom dat op basis van het criterium 'effectiviteit' niet op voorhand kan worden geconcludeerd dat het preventieplan van Clarian ongerechtvaardigd is, mits het wordt gezien als een (interessant) experiment. De vraag of een dergelijk experiment (ethisch) aanvaardbaar is, komt aan bod bij de criteria die nog volgen (met name de criteria 'indringendheid' en 'lasten-batenverhouding').

7.2 Achtste criterium: indringendheid

7.2.1 Operationalisering en wijze van beoordeling

Operationalisering

Het criterium 'indringendheid' definieer ik, conform de derde schadecategorie die ik heb beschreven in §6.1.1, als de inbreuk die de preventiemaatregel maakt op het persoonlijk leven van mensen. Ter operationalisering van het criterium 'indringendheid' ga ik achtereenvolgens in op de:

I. Verschillende aspecten van het criterium 'indringendheid'
II. Vragen behorend bij de verschillende aspecten van het criterium 'indringendheid'
III. Mate van drang of dwang waarmee de preventiemaatregel wordt toegepast.

Ad I. Verschillende aspecten van het criterium indringendheid

In het kader van het model onderscheid ik zes aspecten van het criterium indringendheid[3,4] (Gostin, 2009):

1. Aantasting van de lichamelijke integriteit
2. Aantasting van de vrijheid
3. Aantasting van de privacy
4. Aantasting van de veiligheidsbeleving
5. Discriminatie (inbreuk op de gelijke behandeling)

---

3. *"Although a variety of burdens or harms might exist in public health programs, the majority will fall into 3 broad categories: risks to privacy and confidentiality, especially in data collection activities; risks to liberty and selfdetermination, given the power accorded public health to enact almost any measure necessary to contain disease; and risks to justice, if public health practitioners propose targeting public health interventions only to certain groups."* (Kass, 2001, p.1779)
4. *"Privacy—safeguarding personal information against unauthorized or unjustified disclosure—is a foundational individual good that respects personal dignity and protects patients from embarrassment, stigma, and discrimination."* (Gostin, April 2009, p.1373)

6. Stigmatisering (aantasting van de eer of goede naam, waardigheid, of respectvolle bejegening).

Hieronder bespreek ik de zes aspecten.

1. Aantasting van de lichamelijke integriteit

Het recht op lichamelijke integriteit kan worden gezien als aspect van het recht op privacy (Allen 2004):

> *"Another aspect of physical privacy is bodily integrity. Mandatory testing and health evaluation offend bodily integrity by forcing persons to submit their bodies to unwanted touching and visual inspection."*

Aantasting van de lichamelijke integriteit acht ik echter zo indringend, dat ik deze toch afzonderlijk wil benoemen. Ook in de Universele Verklaring van de Rechten van de Mens (UVRM) (VN, 1948) en de Nederlandse Grondwet (Grondwet, 1815) wordt – weliswaar in andere bewoordingen – het recht op lichamelijke integriteit afzonderlijk benoemd.

In navolging van Anita Allen (2009) wordt 'aantasting van de lichamelijke integriteit' door mij ruimer opgevat dan de letterlijke binnendringing van het lichaam. Bij de letterlijke binnendringing van het lichaam kan bijvoorbeeld worden gedacht aan een operatie, injectie, punctie, biopsie, oraal of anaal of vaginaal onderzoek, of het toedienen van medicatie. In het kader van het model valt direct onderzoek aan of van het lichaam ook onder 'aantasting van de lichamelijke integriteit'. Hierbij kan bijvoorbeeld worden gedacht aan een bloeddrukmeting, visustest, gehoortest, gewichtsmeting, lengtemeting, beeldvormend onderzoek (röntgen, echo, MRI). In het kader van het model valt ook onderzoek van lichaamsafscheidingen (bijvoorbeeld zweet, urine, ontlasting) of lichaamsweefsels (bijvoorbeeld haar, huidschilfers) onder 'aantasting van de lichamelijke integriteit'. Indirect onderzoek van de lichamelijke of psychische gesteldheid, bijvoorbeeld door mondeling of schriftelijk vragen te stellen, valt in het kader van het model niet onder 'aantasting van de lichamelijke integriteit', maar onder aantasting van de privacy.

2. Aantasting van de vrijheid

Onderscheid tussen negatieve en positieve vrijheid

Binnen de filosofie wordt onderscheid gemaakt tussen negatieve en positieve vrijheid (Carter, 2007):

> *"Negative liberty is the absence of obstacles, barriers or constraints. Positive liberty is the possibility of acting — or the fact of acting — in such a way as to take control of one's life and realize one's fundamental purposes. … The idea of distinguishing between a negative and a positive sense of the term 'liberty' goes back at least to Kant, and was examined and defended in depth by Isaiah Berlin in the 1950s and '60s."*

De titel van de beroemde inaugurele rede van Berlin – *Two concepts of liberty* – doelt op dit onderscheid (Berlin, 1958). De UVRM beschrijft zowel negatieve als positieve vrijheidsrechten.

Vrijheidsrechten zijn negatief als ze de afwezigheid van vrijheidsbeperkend gedrag (= negatief gedrag) van andere mensen borgen. Negatieve vrijheidsrechten kunnen als een verbod om de vrijheid van anderen te beperken worden geformuleerd. Voorbeelden van negatieve vrijheidsrechten in de UVRM zijn: verbod op aantasting van de lichamelijke integriteit (artikel 3), verbod op slavernij (artikel 4), verbod op willekeurige arrestatie, detentie of verbanning (artikel 9), en verbod op willekeurige inmenging in persoonlijke aangelegenheden (artikel 12). Negatieve vrijheidsrechten zijn ***afweerrechten***, waarmee mensen binnendringing (denk aan het criterium 'indringendheid') van anderen – met name de overheid – in hun persoonlijke leven kunnen afweren. Als men wil kan men anderen altijd negatieve vrijheid bieden, want men hoeft er niets voor te doen, maar alleen iets voor te laten (geen inbreuk maken op het persoonlijk leven).

Vrijheidsrechten zijn positief als ze de aanwezigheid van vrijheidsverruimend gedrag (= positief gedrag) van andere mensen borgen. Positieve vrijheidsrechten kunnen als een gebod om de vrijheid van anderen te verruimen worden geformuleerd. Voorbeelden van positieve vrijheidsrechten in de UVRM zijn: recht op arbeid voor iedereen (artikel 23), recht op gezondheidszorg voor iedereen (artikel 25) en recht op onderwijs voor iedereen (artikel 26). Artikel 22 van de UVRM gebiedt de maatschappij (met name de overheid) om mensen in hun streven naar zelfstandig functioneren en zelfontplooiing te ondersteunen. Dit kan bijvoorbeeld door het realiseren van een goede werkgelegenheid, goede gezondheidszorg en goed onderwijs. Positieve vrijheden zijn niet gericht op het afweren, maar juist op het betrekken van anderen – met name de overheid – bij het leven van mensen, om de voorwaarden voor zelfstandig functioneren en zelfontplooiing te realiseren. Zelfs als men wil kan men anderen niet altijd alle positieve vrijheden bieden waarop zij volgens de UVRM aanspraak hebben. Het 'recht op arbeid voor iedereen' is bijvoorbeeld moeilijk te realiseren tijdens een economische recessie. Voor de positieve vrijheidsrechten geldt daarom een ***inspanningsverplichting***, maar geen resultaatsverplichting. Dit komt mooi tot uitdrukking in artikel 22 van de UVRM:

> *"Een ieder heeft als lid van de gemeenschap recht op maatschappelijke zekerheid en heeft er aanspraak op, dat door middel van nationale inspanning en internationale samenwerking, en overeenkomstig de organisatie en de hulpbronnen van de betreffende Staat, de economische, sociale en culturele rechten, die onmisbaar zijn voor zijn waardigheid en voor de vrije ontplooiing van zijn persoonlijkheid, verwezenlijkt worden."*

Een aantasting van negatieve vrijheidsrechten (afweren van anderen) valt wel onder het criterium 'indringendheid' van het model. Een aantasting van positieve vrijheidsrechten (betrekken van anderen) valt niet onder het criterium 'indringendheid' (inbreuk op het persoonlijk leven) van het model.

Operationalisering van 'aantasting van negatieve vrijheid'
Binnen het kader van de mensenrechten is het 'recht op vrijheid' een centraal en overkoepelend recht. Zowel het 'recht op lichamelijke integriteit' als het 'recht op privacy', die binnen het model wel afzonderlijk worden benoemd, kunnen onder het 'recht op negatieve vrijheid' worden geschaard (DeCew, 2006):

> *"… privacy appears to be something we value to provide a sphere within which we can be free from interference by others …"*

Zoals besproken kan de 'aantasting van de lichamelijke integriteit' als een aspect van de 'aantasting van de privacy' worden opgevat. Onder het kopje *'(1) Aantasting van de lichamelijke integriteit'* heb ik beargumenteerd waarom ik de aantasting van de lichamelijke integriteit in het model afzonderlijk (los van aantasting van de privacy) benoem. De reden om aantasting van de privacy binnen het model afzonderlijk (los van aantasting van de vrijheid) te benoemen is drieledig. De eerste reden is dat naar mijn ervaring veel mensen het recht op privacy als nevengeschikt aan het recht op vrijheid zien en niet als onderdeel van het recht op vrijheid. De tweede reden is dat het privacyaspect van vrijheid bij leefstijlbeïnvloedende preventiemaatregelen relatief vaak in het geding is. Dit is met name het geval als de leefstijl of gezondheid van de doelgroep wordt gemonitord. De derde reden is dat er veel specifieke wet- en regelgeving van toepassing is op de vertrouwelijkheid van persoonlijke gezondheidsinformatie. Verderop in de tekst ga ik hier nader op in onder het kopje *'(3) Aantasting van de privacy'*.

Binnen het kader van het model vallen onder 'aantasting van de negatieve vrijheid':
- aantasting van de autonomie/ zelfstandigheid/ handelingsvrijheid;
- aantasting van de bewegingsvrijheid;
- aantasting van de keuzevrijheid/ keuzemogelijkheden.

John Christman definieert het begrip 'autonomie' als volgt (Christman, 2009):

> *"Individual autonomy is an idea that is generally understood to refer to the capacity to be one's own person, to live one's life according to reasons and motives that are taken as one's own and not the product of manipulative or distorting external forces."*

Autonomie betekent dat men eigen baas is over het eigen leven. Dat men zelfstandig beslissingen kan nemen. Dat men kan doen en laten (handelingsvrijheid) wat men wil, zo lang men anderen daarmee niet schaadt. Als men dronken wil worden, mag men dronken worden. Als men drugs wil gebruiken, mag men drugs gebruiken. Als men zich wil overeten, mag men zich overeten. Als men niet wil sporten, hoeft men niet te sporten. Enz. De enige voorwaarde is dat men anderen niet schaadt.

De aantasting van de ***bewegingsvrijheid*** benoem ik afzonderlijk vanwege het ruimtelijke aspect dat hieraan verbonden is (zie artikel 13 van de UVRM). Mensen kunnen in hun vrije beweging worden beperkt door hen af te zonderen, op te sluiten, of vast te binden (denk respectievelijk bijvoorbeeld aan een quarantaine-, isoleer-, of fixatiemaatregel), of door hen de toegang tot een bepaalde ruimte of een bepaald gebied te ontzeggen (denk respectievelijk bijvoorbeeld aan een Casinoverbod voor gokverslaafden of een buurtverbod voor stalkers). Op het moment dat men door iemand vast te binden aan het lichaam komt, is er ook sprake van een aantasting van de lichamelijke integriteit.

Een aantasting van de ***keuzevrijheid en keuzemogelijkheden*** betreft in het kader van de bestrijding van leefstijlgerelateerde aandoeningen middels leefstijlbeïnvloedende interventies vaak de aanschaf en consumptie van levens- en genotsmiddelen. Keuzevrijheid definieer ik als de mogelijkheid om vrij te kiezen uit de aanwezige keuzemogelijkheden. Keuzemogelijkheden definieer ik als het aantal opties waaruit gekozen kan worden. Een strikt onderscheid tussen keuzevrijheid en keuzemogelijkheden is m.i. niet mogelijk. Ter illustratie een voorbeeld. Een grote accijnsverhoging op sterke drank vormt volgens de bovengenoemde definitie een aantasting van de keuzevrijheid. Mensen voelen zich minder vrij om sterke drank te kopen. Het aantal verschillende drankproducten dat wordt aangeboden neemt door de accijnsverhoging echter niet af. Volgens de bovengenoemde definitie blijven de keuzemogelijkheden dus gelijk. Voor mensen met een kleine beurs is het kopen van sterke drank financieel mogelijk niet meer haalbaar. Voor hen neemt het aantal reële keuzemogelijkheden wel af.

Een beperking van de keuzevrijheid zou eerder tot ongelijke behandeling van groepen mensen kunnen leiden dan een beperking van de keuzemogelijkheden. Ter illustratie een voorbeeld. Als ter bestrijding van ernstig overgewicht mensen met ernstig overgewicht alleen nog suikervrije frisdrank mogen kopen, is dat een beperking van hun keuzevrijheid. Zij worden ongelijk behandeld ten opzichte van mensen die wel alle soorten frisdrank mogen kopen. Als ter bestrijding van ernstig overgewicht alleen nog suikervrije frisdrank mag worden verkocht, is dat een beperking van de keuzemogelijkheden. Er is echter geen sprake van ongelijke behandeling.

Door een overvloed aan keuzemogelijkheden in de moderne consumptiemaatschappij lopen mensen het risico te lijden aan keuzestress. Barry Schwartz (2004) spreekt in dit verband zelfs van *"Tyranny of choice"*. Als dat het geval is, zou een vermindering van het aantal (ongezonde) keuzemogelijkheden juist bevrijdend kunnen werken.

3. *Aantasting van de privacy*

> *"The term "privacy" is used frequently in ordinary language as well as in philosophical, political and legal discussions, yet there is no single definition or analysis or meaning of the term."* (DeCew, 2006)

Zowel de Universele Verklaring van de Rechten van de Mens (UVRM) (VN, 1948), als de Europese Verklaring van de Rechten van de mens (EVRM) (Raad van Europa, 1950), als ook de Nederlandse Grondwet (1815) kennen artikelen die het recht op privacy van het individu betreffen:

> *"Niemand zal onderworpen worden aan willekeurige inmenging in zijn persoonlijke aangelegenheden, in zijn gezin, zijn tehuis of zijn briefwisseling, noch aan enige aantasting van zijn eer of goede naam. Tegen een dergelijke inmenging of aantasting heeft een ieder recht op bescherming door de wet."* (UVRM, artikel 12)[5]

> *"Recht op eerbiediging van privé-, familie- en gezinsleven. Een ieder heeft recht op respect voor zijn privé leven, zijn familie- en gezinsleven, zijn woning en zijn correspondentie."* (EVRM, artikel 8)

> *"(1) Ieder heeft, behoudens bij of krachtens de wet te stellen beperkingen, recht op eerbiediging van zijn persoonlijke levenssfeer. (2) De wet stelt regels ter bescherming van de persoonlijke levenssfeer in verband met het vastleggen en verstrekken van persoonsgegevens. (3) De wet stelt regels inzake de aanspraken van personen op ken-*

5. In de Engelse versie van de UVRM wordt het woord 'privacy' gebruikt: *"No one shall be subjected to arbitrary interference with his privacy, family, home or correspondence, …"* (artikel 8)

nisneming van over hen vastgelegde gegevens en van het gebruik dat daarvan wordt gemaakt, alsmede op verbetering van zodanige gegevens." (Grondwet, artikel 10)

Binnen het kader van het model valt onder een 'aantasting van de privacy' het niet voldoen aan de volgende privacyeisen (Lee, 2009, p.83) (Mello, 2009, no.3 p.576-577) (Allen, 2009):

- de vertrouwelijkheid van het privéleven, familieleven en gezinsleven van het individu wordt gerespecteerd (vertrouwelijkheid van het privéleven);
- de hoeveelheid persoonlijke informatie die wordt verzameld wordt tot een minimum beperkt (minimale omvang van de dataverzameling);
- de wijze waarop persoonlijke informatie wordt verzameld en opgeslagen is veilig (veilige dataverzameling en -opslag);
- het aantal personen dat toegang heeft tot persoonlijke informatie wordt tot een minimum beperkt (minimale toegang tot de dataverzameling);
- de persoonlijke informatie die is verzameld, wordt alleen gebruikt voor het doel waarvoor deze is verzameld (doelbinding van datagebruik);
- personen die kennis hebben van persoonlijke informatie gaan daar vertrouwelijk mee om (vertrouwelijkheid van persoonlijke informatie);
- de personen die toegang hebben tot persoonlijke informatie zijn zoveel mogelijk onafhankelijk van de personen waarover informatie wordt verzameld (dataverzameling en –opslag onafhankelijk van privéleven en werk).

Lawrence Gostin geeft een heldere onderbouwing van het belang van eerbiediging van privacy (Gostin, April 2009, p.1373):

> *"Privacy – safeguarding personal information against unauthorized or unjustified disclosure – is a foundational individual good that respects personal dignity and protects patients from embarrassment, stigma, and discrimination. Privacy also has societal value because it encourages individuals to participate in socially desirable activities, including research and public health activities."*

4. Aantasting van de veiligheidsbeleving

Het criterium 'indringendheid' betreft de inbreuk die de preventiemaatregel maakt op het persoonlijk leven van mensen. Onveiligheid maakt een inbreuk op het persoonlijk leven van mensen, als de onveiligheid ertoe leidt dat mensen zich onveilig voelen. Het gaat hierbij niet om leven in angst vanwege een angststoornis. Het gaat om een terugkerend gevoel van onveiligheid, omdat men leeft in een omgeving die veiliger zou kunnen en behoren te zijn. Bij leven in een onveilige omgeving kan bijvoorbeeld worden gedacht aan leven in een oorlogsgebied, leven in een buurt met veel geweld, criminaliteit en vandalisme, of leven in een thuissituatie waarin

men wordt mishandeld of verwaarloosd. Leven in een onveilige omgeving kan het persoonlijk welzijn aantasten. Zelfs als men zelf nooit het directe slachtoffer is van de onveiligheid, maar bijvoorbeeld toeschouwer is van criminaliteit of er met regelmaat over leest in de krant.

Hoe kunnen leefstijlbeïnvloedende interventies ter bestrijding van leefstijlgerelateerde aandoeningen de veiligheids***beleving*** aantasten? Ter illustratie een voorbeeld. Stel dat de gemeentelijke overheid besluit, ter bestrijding van drugsverslaving, groepen verslaafden huisvesting in een woonwijk aan te bieden, terwijl zij eerst in het centrum van de stad op straat zwierven. Voor de bewoners van de woonwijk kan een dergelijke interventie een aantasting vormen van hun veiligheidsbeleving. Zij kunnen zich zorgen maken over toenemende criminaliteit in hun woonwijk en toenemende onveiligheid voor buiten spelende kinderen (rondslingerende injectiespuiten). Leefstijlbeïnvloedende interventies ter bestrijding van leefstijlgerelateerde aandoeningen zullen in het algemeen niet leiden tot een aantasting van de veiligheidsbeleving, maar het bovenstaande voorbeeld toont aan dat dit toch niet is uitgesloten.

5. Discriminatie

Binnen het model tracht ik tot een afbakening te komen tussen enerzijds het ‘recht op gelijke behandeling (verbod op discriminatie)’ dat onderdeel is van het criterium ‘indringendheid’ (7e criterium) en anderzijds de criteria ‘rechtvaardigheid’ (11e criterium) en ‘rechtsgelijkheid’ (12e criterium).

Recht op gelijke behandeling (verbod op discriminatie)
De Nederlandse Grondwet stelt in artikel 1:

> *“Allen die zich in Nederland bevinden, worden in gelijke gevallen gelijk behandeld. Discriminatie wegens godsdienst, levensovertuiging, politieke gezindheid, ras, geslacht of op welke grond dan ook, is niet toegestaan.”*

Op basis hiervan definieer ik ***discriminatie*** als ***ongelijke behandeling in gelijke gevallen***. Als mensen worden benadeeld ten opzichte van andere mensen en de reden hiervoor bestaat niet uitsluitend uit de (extra) schade, overlast of schending van morele opvattingen die zij veroorzaken, is er sprake van discriminatie. Mensen met een handicap vormen hierop een uitzondering. Zij worden (wettelijk) zoveel mogelijk beschermd tegen ‘ongelijke behandeling op basis van hun handicap’, terwijl hun handicap wel extra schade, overlast of schending van morele opvattin-

gen[6] met zich meebrengt (zij hebben recht op een gelijke behandeling, terwijl hun geval niet gelijk is). In Amerika is de extra bescherming van mensen met een handicap tegen ongelijke behandeling vastgelegd in de 'Americans with Disabilities Act' (ADA). Vooralsnog worden ernstig overgewicht en verslaving (aan roken, drank, of drugs) door de Amerikaanse rechters niet als handicap in de zin van de ADA geïnterpreteerd. Positieve discriminatie valt, evenals positieve vrijheid, niet onder het criterium 'indringendheid' van het model.

Rechtvaardigheid en rechtsgelijkheid

De criteria 'rechtvaardigheid' en 'rechtsgelijkheid' van het model betreffen alle vormen van ongelijke behandeling en ongelijke verdeling tussen mensen, inclusief discriminatie en positieve discriminatie.

Ter verduidelijking van de betekenis van het begrip 'discriminatie' binnen het model geef ik een aantal (fictieve) voorbeelden.

Wel discriminatie in het kader van het model:

a. De werkgever kort alleen vrouwelijke werknemers die roken op de vergoeding van hun zorgverzekeringspremie i.v.m. hogere zorgkosten.
b. Lichamelijk gehandicapten moeten extra betalen voor een plaats in het vliegtuig vanwege de extra ruimte die hun rolstoel inneemt, het extra gewicht dat hun rolstoel heeft en de extra zorg die zij nodig hebben.
c. Ter bestrijding van seksueel overdraagbare aandoeningen stelt de overheid alleen voor homoseksuele mannen condoomgebruik verplicht.
d. Alleen Antilliaanse gevangenen worden getest op drugsgebruik in de gevangenis.
e. Ter voorkoming van verdrinking mogen allochtone kinderen niet zonder direct toezicht van een volwassene met zwemdiploma zwemmen.

Geen discriminatie in het kader van het model:

f. De werkgever kort rokende werknemers op de vergoeding van hun zorgverzekeringspremie i.v.m. hogere zorgkosten.
g. Volwassenen met ernstig overgewicht moeten verplicht twee zitplaatsen betalen in het vliegtuig, maar kinderen met ernstig overgewicht niet.
h. Iedereen wordt verplicht om met een condoom te vrijen.
i. Drugsverslaafde gevangenen worden getest op drugsgebruik in de gevangenis.

6. Zij kunnen bijvoorbeeld moeite hebben om met mes en vork te eten, maar worden om die reden ook in een chique restaurant niet geweerd als klant.

j. Kinderen mogen alleen onder direct toezicht van een volwassene met zwem-diploma zwemmen.

Toelichting op ‘wel discriminatie in het kader van het model’:

a. Het onderscheid dat de werkgever maakt berust in substantiële mate op een man-vrouwverschil en niet uitsluitend op een verschil in zorgkosten tussen rokende vrouwelijke werknemers en rokende mannelijke werknemers.
b. Gehandicapten mogen niet worden benadeeld t.o.v. andere mensen op basis van de schade, overlast of schending van morele opvattingen die hun handicap met zich brengt.
c. Het onderscheid dat de overheid maakt berust in substantiële mate op een man-vrouwverschil en een verschil in seksuele geaardheid en niet uitsluitend op een verschil in het risico op het overdragen van seksuele aandoeningen.
d. Het onderscheid dat wordt gemaakt berust in substantiële mate op een verschil in etnische afkomst en niet uitsluitend op een verschil in drugsgebruik.
e. Het onderscheid dat wordt gemaakt berust in substantiële mate op een verschil in etnische afkomst en niet uitsluitend op een verschil in risico op verdrinking.

Toelichting op ‘geen discriminatie in het kader van het model’:

f. Dat alleen werknemers worden gekort en werklozen niet, benoem ik niet als discriminatie (criterium ‘indringendheid’), maar als rechtsongelijkheid (12e criterium). De werkgever discrimineert niet tussen werknemers en werklozen, maar heeft alleen met werknemers te maken.
g. Volwassenen met ernstig overgewicht veroorzaken meer overlast voor hun medepassagiers dan volwassenen zonder ernstig overgewicht en dan kinderen met ernstig overgewicht.
h. Een vrijheidsbeperking die thuishoort onder het criterium ‘indringendheid’ van het model, maar geen discriminatie.
i. Een vrijheidsbeperking die thuishoort onder het criterium ‘indringendheid’ van het model, maar geen discriminatie. Drugsverslaafde gevangen hebben een hoger risico op druggebruik dan niet drugsverslaafde gevangenen, verondersteld dat drugsgebruik buiten de gevangenis niet en binnen de gevangenis wel verboden is.
j. Er wordt onderscheid gemaakt tussen kinderen en volwassenen, maar het is algemeen aanvaard in onze maatschappij dat kinderen meer dan volwassenen (tegen zichzelf) moeten worden beschermd. Bovendien lopen kinderen waarschijnlijk een groter risico op verdrinking dan volwassenen.

6. Stigmatisering

Stigma betekent letterlijk 'brandmerk, schandvlek, merkteken' en heeft indien figuurlijk gebruikt meestal een ongunstige betekenis (MacLean, 2009, p.89):

> *"It is generally agreed that being obese, or even being overweight, is a highly stigmatized condition. There is a considerable discussion in the literature on the stigma of obesity and the way that obese people are treated in western society, from childhood teasing and bullying, avoidance by others, discriminatory hiring practices and misplaced humour. Rogge et al. used the term 'civilized oppression' to describe the pervasive pattern of ongoing, daily denigration and condemnation that constitutes living as an obese person. Stigma may worsen obesity through dynamics such as fear of going out, fear of ridicule while exercising, cycles of emotional eating and the development of eating disorders (Schwartz and Brownell, 2007). Further, stigmatization is correlated with significant health problems such as depression, hypertension, coronary heart disease and stroke (Major and O'Brien, 2005; Stuber et al., 2008). Thus, stigma itself may independently contribute to the health risks associated with obesity"*

Mensen die worden gestigmatiseerd, worden op basis van een afwijkend kenmerk stelselmatig met minachting of negatief door anderen benaderd. Discriminatie kan leiden tot stigmatisering, maar is niet hetzelfde als stigmatisering:
- discriminatie heb ik gedefinieerd als ongelijke behandeling in gelijke gevallen;
- ***stigmatisering*** definieer ik als ***onevenredig ongelijke benadering***.

Discriminatie kan plaatsvinden zonder dat er sprake is van stigmatisering, bijvoorbeeld als iemand een (onzichtbare) ziekte of handicap heeft. Een (onzichtbare) ziekte of handicap kan leiden tot onterechte achterstelling bij sollicitaties (= discriminatie), maar hoeft niet te leiden tot stigmatisering (ongelijke benadering) in het dagelijks leven.

Stigmatisering kan plaatsvinden zonder dat er sprake is van discriminatie. Denk bijvoorbeeld aan iemand met ernstig overgewicht die op basis hiervan door collega's op het werk stelselmatig wordt geminacht, belachelijk wordt gemaakt, of negatief wordt benaderd. In dat geval is er geen sprake van discriminatie, want er is geen sprake van 'ongelijke behandeling'. Wel is er sprake van een onevenredig ongelijke benadering. De schade, overlast of schending van morele opvattingen die iemand met ernstig overgewicht aan anderen (de samenleving) (en aan zichzelf) toebrengt, vormt geen rechtvaardiging voor de mate van afwijzing die iemand ten deel valt (Puhl, 2010, p1020-1021):

> *Experimental research in psychology consistently demonstrates that obese persons are stigmatized because their weight is perceived to be caused by factors within per-*

sonal control (e.g., overeating and lack of exercise). … Thus, the pervasiveness of the "personal responsibility" message plays a key role in stigmatization, and serves to justify stigma as an acceptable societal response. However, this prevailing message does not accurately reflect the science. Many significant contributors to obesity are beyond the control of individuals. In addition to the important role of genetic and biological factors regulating body weight, multiple social and economic influences have significantly altered the environment to promote and reinforce obesity."

In de literatuur worden discriminatie en stigmatisering vaak in één adem genoemd. Vanwege de door mij beschreven verschillen tussen 'discriminatie' en 'stigmatisering' heb ik besloten ze binnen het criterium 'indringendheid' van het model van elkaar te onderscheiden.

Ad II. Vragen behorend bij de verschillende aspecten van het criterium 'indringendheid'

Ter operationalisering van het criterium 'indringendheid' geef ik hieronder een overzicht van de te beantwoorden vragen bij het beoordelen van de indringendheid. Deze vragen zijn van toepassing op de hierboven beschreven zes aspecten van het criterium 'indringendheid':

a. Bij wie wordt een inbreuk gemaakt op het persoonlijk leven?
   - Alleen bij mensen binnen de doelgroep of ook bij mensen buiten de doelgroep?
   - Welke verantwoordelijkheid hebben de mensen bij wie een inbreuk wordt gemaakt op hun persoonlijk leven voor de risico's die men met de preventiemaatregel tracht te bestrijden:
     - verantwoordelijkheid voor de risico's die zijzelf lopen (als mensen tegen zichzelf worden beschermd is er sprake van paternalisme)?
     - verantwoordelijkheid voor de risico's die anderen lopen?
     - geen verantwoordelijkheid voor deze risico's c.q. de persoon is een buitenstaander in deze kwestie.

b. Door wie wordt een inbreuk gemaakt op het persoonlijk leven?
   - Wordt een inbreuk op het persoonlijk leven gemaakt door de overheid of door private partijen? In het algemeen geldt, dat hoe minder er sprake is van democratische controle, hoe groter het risico wordt op willekeur. De aan- of afwezigheid van willekeur wordt echter medebepaald door andere factoren dan de controle hierop; bijvoorbeeld door de kwaliteit en integriteit van een organisatie en de personen die er werken.

c. In welke mate wordt een inbreuk gemaakt op het persoonlijk leven?

- Bijvoorbeeld te beoordelen aan de hand van een vijfpuntsschaal:
  - in zeer grote mate;
  - in grote mate;
  - niet in grote/ niet in kleine mate;
  - in kleine mate;
  - in zeer kleine mate.

d. Welke reikwijdte heeft de inbreuk op het persoonlijk leven?
   - Bij hoeveel mensen wordt een inbreuk gemaakt op het persoonlijk leven?
   - Is de inbreuk beperkt in de tijd (eenmalig of herhaald; voor bepaalde of onbepaalde tijd)?
   - Is de inbreuk beperkt tot bepaalde situaties (alleen op het werk; alleen in de sportschool)?

e. Leidt het toestaan van de inbreuk op het persoonlijk leven tot precedentwerking?
   - De partij die de indringende preventiemaatregel toepast, kan in de verleiding komen om steeds meer ongezond gedrag te willen veranderen en/of steeds meer drang of dwang toe te passen bij het veranderen van ongezond gedrag.
   - Voor andere partijen wordt de drempel verlaagd om indringende preventiemaatregelen te treffen. Bijvoorbeeld: als de ene werkgever roken mag verbieden, mag ook de andere werkgever roken verbieden.

f. Hoe bezwaarlijk is de inbreuk op het persoonlijk leven?
   - Bijvoorbeeld te beoordelen aan de hand van een vijfpuntsschaal:
     - zeer bezwaarlijk;
     - bezwaarlijk;
     - niet bezwaarlijk/ niet onbezwaarlijk;
     - onbezwaarlijk;
     - zeer onbezwaarlijk.

Bij vraag f wordt eigenlijk gevraagd om een samenvattend oordeel op basis van de antwoorden op de voorafgaande vragen a t/m e.

Ad III. Mate van drang of dwang waarmee de preventiemaatregel wordt toegepast

De mate van drang of dwang waarmee de preventiemaatregel wordt toegepast, is in belangrijke mate bepalend voor de indringendheid van de preventiemaatregel. De vraag naar de drang of dwang waarmee de preventiemaatregel wordt toegepast,

is meestal van toepassing op de preventiemaatregel als geheel. Daarom wordt deze vraag afzonderlijk besproken (los van de vragen die behoren bij de zes aspecten van het criterium 'indringendheid').

In welke mate worden mensen gedrongen of gedwongen om de inbreuk op hun persoonlijk leven te ondergaan? Aan drang of dwang kan op verschillende manieren invulling worden gegeven. Een rangschikking van minder naar meer indringend zou er als volgt uit kunnen zien:

- moreel beroep (er wordt een moreel beroep op de persoon gedaan om bepaald gedrag te vertonen of na te laten);
- positieve financiële prikkels (de persoon wordt financieel beloond voor het vertonen of nalaten van bepaald gedrag);
- negatieve financiële prikkels (de persoon wordt financieel gestraft voor het vertonen of nalaten van bepaald gedrag);
- wettelijk verbod of gebod.

Deze volgorde, van minder naar meer indringend, is indicatief en niet absoluut. Een kind kan het morele beroep dat zijn ouders op hem doen om geen alcohol te gebruiken bijvoorbeeld als meer vrijheidsbeperkend ervaren dan de accijns (negatieve financiële prikkel) op sigaretten. De mate van drang of dwang wordt ook bepaald door de vraag of mensen zich aan de inbreuk op hun persoonlijk leven kunnen onttrekken. Bijvoorbeeld door niet deel te nemen aan het preventieprogramma, of door bepaalde situaties (als men autorijdt, loopt men het risico op alcoholgebruik te worden gecontroleerd) te vermijden.

Wijze van beoordeling

Bij het criterium 'indringendheid' (8e criterium) gaat het om de aanvaardbaarheid van de verhouding tussen bepaalde lasten en bepaalde baten van de preventiemaatregel, waarbij de lasten worden gevormd door de indringendheid en de baten door de effectiviteit. Is de verhouding tussen de indringendheid en effectiviteit van de preventiemaatregel aanvaardbaar? Bij het beoordelen van de aanvaardbaarheid maak ik gebruik van het falsificatiebeginsel. De preventiemaatregel passeert het criterium 'indringendheid' van het model, als niet kan worden aangetoond dat de verhouding tussen de indringendheid en effectiviteit onaanvaardbaar is.

De verschillende aspecten van het criterium indringendheid zijn:

1. aantasting van de lichamelijke integriteit;
2. aantasting van de vrijheid;
3. aantasting van de privacy;
4. aantasting van de veiligheidsbeleving;
5. discriminatie;

6. stigmatisering.

De vragen behorend bij deze aspecten zijn:
a. Bij wie (van wie[7]) wordt een inbreuk gemaakt op het persoonlijk leven?
b. Door wie wordt een inbreuk gemaakt op het persoonlijk leven?
c. In welke mate wordt een inbreuk gemaakt op het persoonlijk leven?
d. Welke reikwijdte heeft de inbreuk op het persoonlijk leven?
e. Leidt het toestaan van de inbreuk op het persoonlijk leven tot precedentwerking?
f. Hoe bezwaarlijk is de inbreuk op het persoonlijk leven?

De vragen (a t/m f) zijn van toepassing op alle aspecten (1 t/m 6) van het criterium 'indringendheid'. In totaal zijn er (6 x 6 =) 36 vragen te beantwoorden. In overzicht 12 is dit grafisch weergeven, doordat in de linker kolom de vragen (a t/m f) zijn geplaatst en in de bovenste rij de aspecten (1 t/m 6). In totaal levert dit een tabel met 36 lege cellen op, die ingevuld dienen te worden.

Overzicht 12: beoordeling van de indringendheid van de preventiemaatregel

		1	2	3	4	5	6
		Aantasting lichamelijke integriteit	Aantasting vrijheid	Aantasting privacy	Aantasting veiligheids-beleving	Discrimi-natie	Stigmati-sering
a	Van wie?						
b	Door wie?						
c	In welke mate?						
d	Reikwijdte?						
e	Precedent-werking?						
f	Hoe bezwaarlijk?						

Met het oog op de uitvoerbaarheid van de beoordeling pak ik de beoordeling van de indringendheid van de preventiemaatregel als volgt aan:
– Eerst wordt een algemene beoordeling gegeven van de 'indringendheid' van de preventiemaatregel aan de hand van de vragen a t/m f;

7. Van wie wordt de lichamelijke integriteit, vrijheid, privacy of veiligheidsbeleving aangetast? Discriminatie of stigmatisering van wie?

– Vervolgens wordt ingezoomd op de verschillende aspecten (1 t/m 6) van de indringendheid. Afhankelijk van de betreffende preventiemaatregel kan per aspect worden besloten om, al dan niet en meer of minder uitgebreid, op de zes bijbehorende vragen (a t/m f) in te gaan. Dat biedt de mogelijkheid om op de meest indringende kenmerken van de preventiemaatregel te focussen. Omdat op basis van het falsificatiebeginsel wordt beoordeeld of de preventiemaatregel het criterium 'indringendheid' passeert, acht ik focussen op de meest indringende kenmerken aanvaardbaar. Het eindoordeel luidt dat de beoordeling al dan niet heeft geleid tot falsificatie van de aanvaardbaarheid van de indringendheid. Een dergelijk oordeel is ook correct als niet voor alle aspecten (1 t/m 6) van het criterium indringendheid alle bijbehorende vragen (a t/m f) zijn beantwoord.
– Daarna wordt de mate van drang of dwang beoordeeld waarmee de preventiemaatregel wordt toegepast.

7.2.2 Beoordeling van casus Clarian Health aan de hand van criterium 'indringendheid'

Algemene beoordeling van de 'indringendheid' van de preventiemaatregel

a. Bij wie wordt een inbreuk gemaakt op het persoonlijk leven?
Bij alle werknemers van Clarian wordt een inbreuk gemaakt op het persoonlijk leven, doordat zij periodiek informatie over hun gezondheid aan de werkgever (of een tussenpersoon, dat wordt niet duidelijk) dienen te overleggen, als zij niet substantieel meer willen betalen voor hun zorgverzekering. Clarian schat dat momenteel 34% van haar werknemers obesitas heeft en dat 26% rookt (BusinessWeek, McGregor, 2007). De overige gezondheidsindicatoren – hoge bloeddruk, hoog glucosegehalte, hoog cholesterolgehalte – uit het preventieplan van Clarian correleren met obesitas en/of roken. Op basis hiervan schat ik dat het preventieplan van Clarian op meer dan 30% van de werknemers druk uitoefent om ongezonde aspecten van hun leefstijl aan te passen. Voor deze werknemers vormt het preventieplan van Clarian eigenlijke een dubbele inbreuk op hun persoonlijk leven. Enerzijds een inbreuk omdat zij persoonlijke gezondheidsinformatie aan de werkgever (of een tussenpersoon) dienen te verstrekken, en anderzijds een inbreuk omdat hun keuzevrijheid wordt aangetast, omdat zij financieel onder druk worden gezet om gezond te leven.

b. Door wie wordt een inbreuk gemaakt op het persoonlijk leven?
De werkgever – een private partij – beperkt de persoonlijke vrijheid van zijn werknemers om er een 'hedonistische' leefstijl op na te houden, die bestaat uit roken,

teveel en/of ongezond eten, te weinig bewegen, e.d. Werknemers zijn voor hun inkomsten afhankelijk van hun baan en verkeren daardoor in een afhankelijke positie t.o.v. hun werkgever. Bovendien is er sprake van een formeel hiërarchische relatie tussen de werkgever en de werknemers. De afhankelijkheid van werknemers van hun werkgevers is een belangrijk aspect om in het oog te houden bij de beoordeling van de indringendheid van de preventiemaatregel. De besluiten van de werkgever worden getoetst door het bestuur of de aandeelhouders, maar vallen niet onder een systematische democratische controle, zoals besluiten van de overheid. Het risico dat onredelijke of onbillijke besluiten niet door derden worden gecorrigeerd (zoals het parlement de regering controleert c.q. corrigeert), is daardoor groter.

c. In welke mate wordt een inbreuk gemaakt op het persoonlijk leven?

In §3.3 heb ik betoogd dat de ‘effecten en neveneffecten’ van preventiemaatregelen met het oog op de onderlinge vergelijkbaarheid en objectiviteit zoveel mogelijk gekwantificeerd dienen te worden. Het belang van kwantificering geldt uiteraard ook voor de indringendheid van de preventiemaatregel (inbreuk op het persoonlijk leven), omdat de indringendheid een neveneffect van de preventiemaatregel is. Voor het kwantificeren van de indringendheid kan gebruik worden gemaakt van de concepten ***‘Willingness to Pay’ en ‘Willingness to Accept’*** (zie §6.1.1). Werknemers van Clarian kunnen deelname aan de preventiemaatregel jaarlijks afkopen als zij de maximale jaarlijkse ‘boete’ betalen, die in 2009 $650 bedraagt[8]. Zo bezien zou men kunnen stellen dat de indringendheid c.q. de inbreuk op het persoonlijk leven een waarde van maximaal $650 per jaar vertegenwoordigt. Wat betekent een dergelijk bedrag voor een werknemer van Clarian? Het gemiddelde jaarinkomen per hoofd van de bevolking (inclusief niet-werkenden) in de Amerikaanse staat Indiana (in deze staat zijn de ziekenhuizen van Clarian zijn gevestigd) bedroeg $33.725 in 2009[9]. Een afkoopsom van $650 vormt hiervan 1,9%. Zo bezien lijkt een afkoopsom van $650 per werknemer per jaar substantieel, maar niet onoverkomelijk. Bij deze zienswijze dienen diverse kanttekeningen te worden geplaatst. Voor werknemers die in of op de rand van schuld leven, is iedere extra uitgave te veel:

> *"Yes, I do feel like this is punishment because I really can't afford to pay," said LaTonya Johnson, another Clarian employee.* (NBC5, 2007)

Voor mensen met een gezonde leefstijl, die aan alle (of de meeste) gezondheidscriteria van Clarian voldoen, lijkt een afkoopsom van $650, als zij hun gezondheid

8. Zie bijlage 8: toelichting op §f van de HIPAA.
9. USA States in profile. Stats Indiana. http://www.stats.indiana.edu/sip/

niet willen laten onderzoeken en/of geen gezondheidsinformatie over zichzelf willen verstrekken, onredelijk hoog. Zij moeten $650 betalen om te voorkomen dat hun lichamelijke integriteit en privacy worden aangetast, terwijl zij niet verantwoordelijk zijn voor de schade die Clarian wil bestrijden.

Een kwalitatieve benadering van de mate waarin de preventiemaatregel inbreuk maakt op het persoonlijk leven geeft het volgende beeld:

> *"But this is a serious civil rights issue"* (Big Fat Blog, rebelled, August 9, 2007).
>
> *"So here's my question: who do we take this up with? Clearly, this action needs to be protested. So where do I fire off my angry letters? The ACLU [American Civil Liberties Union]? A consumer protection agency? Who has made this legal, and why? I want to fight this, I'm just not clear on where."* (Big Fat Blog, fat chic, August 10, 2007).

Op internet nemen veel mensen de inmenging van Clarian in het persoonlijk leven van haar werknemers hoog op. Zij zien hierin een schending van de burgerrechten (Engels: civil rights[10]). Deze rechten zijn basaal en kunnen burgers alleen om (zeer) zwaarwegende redenen worden ontzegd. De schade die Clarian met haar preventieplan wil bestrijden kan een schending van de burgerrechten volgens genoemde mensen geenszins legitimeren. Sommigen waarschuwen zelfs voor aantasting van de democratie:

> *"If we as Americans start letting the big name corporations tell us how we can and can't live then I think we have lost the battle to control our own destiny."* (MSNBC, Casey, Hartselle, August 10, 2007).
>
> *"Maintaining your health is an obligation to yourself and your family, not to The State. Of course there have been some countries throughout history that required a health standard to be maintained; and we label them T-O-T-A-L-I-T-A-R-I-A-N."* (MSNBC, kingsXrulz, August 12, 2007).

d. Welke reikwijdte heeft de inbreuk op het persoonlijk leven?

Op basis van de door mij bestudeerde reacties op het preventieplan van Clarian, onderscheid ik drie aspecten aan de reikwijdte van het preventieplan, die ertoe

---

10. *"In contemporary political thought, the term 'civil rights' is indissolubly linked to the struggle for equality of American blacks during the 1950s and 60s. The aim of that struggle was to secure the status of equal citizenship in a liberal democratic state. Civil rights are the basic legal rights a person must possess in order to have such a status. They are the rights that constitute free and equal citizenship and include personal, political, and economic rights. No contemporary thinker of significance holds that such rights can be legitimately denied to a person on the basis of race, color, sex, religion, national origin, or disability."* (Altman, 2007)

leiden dat mensen de inbreuk van het preventieplan op het persoonlijk leven van werknemers als groot ervaren. In de eerste plaats noem ik het structurele karakter van het preventieplan. In principe houdt het plan nooit op, maar gaat het altijd door. Als men er niet in slaagt om aan de gezondheidscriteria van Clarian te voldoen, is men zo lang men bij Clarian werkt de pineut. In de tweede plaats is de reikwijdte van het preventieplan groot, omdat de gezondheidscriteria die Clarian hanteert, 24 uur per dag van toepassing zijn. Als men dagelijks hardloopt, is men de rest van de dag niet vrijgesteld van het preventieplan. Als men 's ochtends en 's middags zijn best heeft gedaan om gezond te eten, kan men 's avonds in een moment van 'zwakte' alsnog de inspanning van die dag teniet doen; als men de ene dag zijn best heeft gedaan om gezond te eten, kan men de volgende dag alsnog die inspanning teniet doen; als men de ene week … enz. Ten slotte is het ingrijpend dat de werkgever zich met het leven van zijn werknemers buiten werktijd bemoeit, zeker omdat deze bemoeienis, zoals hierboven toegelicht, onophoudelijk is (MSNBC, P. Evans, August 10, 2007):

> *"Why does the government and private companies feel the need to govern our lives, especially once we leave the work place?"*

e. Leidt het toestaan van de inbreuk op het persoonlijk leven tot precedentwerking?
Als de drang waarmee een preventiemaatregel wordt toegepast klein is, of als de inbreuk die de preventiemaatregel maakt op het persoonlijk leven klein is, kan toepassing van de preventiemaatregel toch ingrijpend zijn, als deze leidt tot precedentwerking. Dat wil zeggen dat toepassing van de preventiemaatregel de deur open zet tot grotere drang en grotere inmenging in het persoonlijk leven. Ter illustratie een fictief voorbeeld aan de hand van het preventieplan van Clarian. Stel dat de 'boetes' die werknemers moeten betalen, als zij niet voldoen aan de gezondheidscriteria van Clarian, worden omgezet van dollars in dollarcenten. De maximale jaarlijkse boete bedraagt dan $6,50 in plaats van $650 (zie antwoord op vraag c). Van de hoogte van dit bedrag zal geen werknemer wakker liggen. Toch kan het preventieplan van Clarian dan nog steeds als ingrijpend worden ervaren. Enerzijds omdat men vindt dat principes waarin men 'heilig gelooft', zoals de burgerrechten, worden geschonden. Dit zou van kwaad tot erger kunnen gaan. Anderzijds omdat het luttele bedrag van $6,50 geleidelijk verhoogd zou kunnen worden, zodat gewenning optreedt en heftig protest uitblijft. Denk hierbij aan het verhaal van de kikker die niet uit de pan met water springt, als deze geleidelijk aan de kook wordt gebracht. In beide gevallen - een kleine inbreuk op 'heilige principes' en (beginnen met) beperkte drang – is er sprake van precedentwerking, die toepassing van de preventiemaatregel (in de beleving van mensen) ingrijpend kan maken, zonder dat de maatregel op zichzelf ingrijpend is. Diverse mensen die op internet op het

preventieplan van Clarian hebben gereageerd, vinden het preventieplan van Clarian zowel ingrijpend op zichzelf, als ingrijpend vanwege de precedentwerking die ervan uitgaat:

> *"The problem that Gruber and other critics see is that programs like Clarian's collect personal medical data on employees. Today, it's information on blood pressure and cholesterol. Tomorrow, as Lauer wondered aloud, will it be asking single employees about their sexual habits and their use of condoms? "You can turn health care into a police state," Lauer said, asking Evans [Evans is CEO van Clarian] where the line is to be drawn."* (MSNBC.com, TodayShow, 2007)

> *"What's next, if someone is gay they are going to be charged? What about people with children...where will it stop?"* (MSNBC, Holly, August 10, 2007)

> *"Are we going to start charging pre-menopausal women more because they might get pregnant? Women more in general because they are more likely to get breast cancer? Employees who report working 70+ hours a week more because the stress might cause them to have HBP [high blood pressure] and other conditions? Asians, Hispanics and blacks more becaue they are more likely to develop diabetes? Where does it end?"* (Big Fat Blog, jmars, August 9, 2007)

> *"Clarion Health has some nerve taking money out of people's pockets purely because of their weight. This kind of mind-bending garbage has to stop before it becomes pervasive."* (Big Fat Blog, rebelled, August 9, 2007)

> *"I can appreciate Clarian wanting to keep costs in check, but as stated by many, privacy issues abound with this proposal. What other information are they gathering that can be used against the employee down the road...DNA tests to determine other potential illnesses? I agree this crosses the line"* (MSNBC, Pam K., August 11, 2007)

> *"I guess i feel so strongly about this particular issue because I see it as being both a beach head for anti fat legislation and because I feel it might be right on the cusp. …"* (Big Fat Blog, Bilt4Cmfrt, August 20, 2007)

> *"Any time one company is successful at this kind of discrimination, it opens the door for others to follow and we lose ground."* (Big Fat Blog, vesta44, September 9, 2007)

f. Hoe bezwaarlijk is de inbreuk op het persoonlijk leven?

De indringendheid van de preventiemaatregel van Clarian acht ik bezwaarlijk vanwege:

- het gebrek aan democratische controle op de 'indringendheid' van de preventiemaatregel (zie het antwoord op vraag b);
- de mate waarin de preventiemaatregel inbreuk maakt op het persoonlijk leven (zie het antwoord op vraag c en a);

- de reikwijdte van de inbreuk op het persoonlijk leven (zie het antwoord op vraag d);
- de precedentwerking die van het preventieplan kan uitgaan (bij de inrichting van de maatschappij moeten we m.i. niet een kant op waarbij werkgevers een grote invloed kunnen uitoefenen op de leefstijl van werknemers buiten werktijd) (zie het antwoord op vraag d en b).

Bovenal lijkt mij het gegeven dat mensen met een gezonde leefstijl, die aan alle (of de meeste) gezondheidscriteria van Clarian voldoen, $650 moeten betalen, als zij willen voorkomen dat hun werkgever hun lichamelijke integriteit en privacy aantast, niet te rechtvaardigen (zie het antwoord op vraag c).

Belangrijk is om ook oog te hebben voor de beleving van de inbreuk op het persoonlijk leven van de mensen die dit direct betreft. Zij kunnen de inbreuk op hun persoonlijk leven soms anders ervaren dan 'buitenstaanders'. Denk bijvoorbeeld aan preventief fouilleren in buurten met veel criminaliteit. Het is denkbaar dat juristen hier bezwaar tegen maken, terwijl de buurtbewoners die (onterecht) worden gefouilleerd het wel prima vinden. Op het internet zijn relatief weinig reacties van werknemers van Clarian op het preventieplan te vinden. Wellicht omdat zij hun werkgever niet schriftelijk en in het openbaar durven te bekritiseren.

De meeste reacties op internet over het preventieplan van Clarian zijn (zeer) negatief. Ter illustratie geef ik enkele voorbeelden:

> *"This really is scary."* (Big Fat Blog, jportnick, August 9, 2007)
>
> *"I am so outraged by Clarian's "punishment" of fat employees …"* (Big Fat Blog, Alyssa, August 10, 2007)
>
> *"I am so sick and tired of obesity being the only thing that it is still okay to discriminate against."* (Big Fat Blog, taylo500, August 14, 2007)
>
> *"This is absurd! The idea of being able to discriminate in this manner is sickening!"* (MSNBC, Holly, August 10, 2007)
>
> *"Enough is enough! Clarion is stomping on personnal rights. If allowed to continue this practice then they should be banned from Indiana and any other state."* (MSNBC, Brenda, Carolina, August 11, 2007)
>
> *"If my employer had this policy I would be so stressed out that I would get sick just because of the thought of it all."* (MSNBC, S Mck, August 11, 2007)

Ten slotte zijn het niet alleen mensen met een ongezonde leefstijl die ernstig verontrust zijn over het preventieplan van Clarian:

"I just watched the segment about Clarian Health and I was absolutly appalled. I am not a smoker nor do I have weight or blood pressure problems but if I did that would be my problem. For Clarian Health to tell their employees that they can either shape up or pay up seems to me like a violation of their rights." (MSNBC, Casey, Hartselle, August 10, 2007)

Hierboven heb ik de vragen a t/m f behorend bij het criterium 'indringendheid' besproken (zie overzicht 12). Hieronder bespreek ik de aspecten 1 t/m 6 behorend bij het criterium 'indringendheid' (zie overzicht 12).

Beoordeling van de zes aspecten van het criterium 'indringendheid'

1. *Indringendheid van de aantasting van de lichamelijke integriteit*

"Mandatory testing and health evaluation offend bodily integrity by forcing persons to submit their bodies to unwanted touching and visual inspection." (Allen 2004).

Dit is precies waarvan binnen het preventieplan van Clarian sprake is. Dat door periodiek onderzoek ook de lichamelijke integriteit van werknemers die voldoen aan alle gezondheidscriteria van Clarian wordt aangetast, heb ik een van de bezwaarlijkste aspecten van het preventieplan genoemd. Daarom bevreemdt het mij zeer dat uit de door mij bestudeerde teksten op geen enkele manier duidelijk wordt aan welke aantastingen van de lichamelijke integriteit de werknemers van Clarian worden blootgesteld.

2. *Indringendheid van de aantasting van de vrijheid*

Het preventieplan van Clarian tast de keuzevrijheid aan, maar niet de keuzemogelijkheden. De beperking van de keuzevrijheid kan twee kanten opgaan. De vrijheid om ongezond te leven wordt ingeperkt en/of de vrijheid om eigen geld naar eigen inzicht te besteden wordt ingeperkt. Als werknemers over veel geld zouden beschikken, zouden ze er misschien wel veel meer dan $650,- (de maximale boete per jaar) voor over hebben om vrij te kunnen beslissen over de eigen leefstijl. Hoe de inperking van de vrijheid om zelf te beslissen over de eigen leefstijl kwantitatief (financieel) en kwalitatief gewaardeerd moet worden, is daarom moeilijk in te schatten. Uit de reacties op internet ontstaat de indruk, dat veel mensen denken dat het preventieplan voor de werknemers van Clarian op het (onophoudelijk) betalen van boetes uit zal draaien:

"Clarion Health has some nerve taking money out of people's pockets purely because of their weight." (Big Fat Blog, rebelled, August 9, 2007)

"Get your hand out of my pocket! This is crazy. … Fat people are being mugged [beroofd], on the street, in broad daylight, with a cop standing right there providing

tips for the Efficient Mugger and there's 'not much we can do about it'?" (Big Fat Blog, Bilt4Cmfrt, August 20, 2007)

In hoeverre dit komt, doordat mensen hun ongezonde leefstijl niet willen aanpassen en/of niet kunnen aanpassen, kan niet uit de reacties worden opgemaakt.

3. Indringendheid van de aantasting van de privacy

In §7.2.1 heb ik aantasting van de privacy geoperationaliseerd als het niet voldoen aan de privacyeisen, die ik op basis van literatuur ten behoeve van het model heb vastgesteld (zie de linker kolom van overzicht 13).

Overzicht 13: voldoet het preventieplan van Clarian aan privacyeisen

**De privacy wordt aangetast als niet wordt voldaan aan de onderstaande privacyeisen**	**Voldoet het preventieplan van Clarian aan de betreffende privacyeis?**
De vertrouwelijkheid van het privéleven, familieleven en gezinsleven van het individu wordt gerespecteerd (vertrouwelijkheid van het privéleven).	Nee, het preventieplan maakt inbreuk op de vertrouwelijkheid van het privéleven, omdat werknemers informatie over hun leefstijl en gezondheid aan de werkgever dienen te verstrekken. Het is onduidelijk hoe groot deze inbreuk is, omdat onduidelijk is welke vragen over hun gezondheid en leefstijl werknemers moeten beantwoorden.
De hoeveelheid persoonlijke informatie die wordt verzameld, wordt tot een minimum beperkt (minimale omvang van de dataverzameling).	Onduidelijk, door gebrek aan informatie hierover (in de media wordt de indruk gewekt dat Clarian per werknemer heel veel informatie over de gezondheid en leefstijl verzamelt).
De wijze waarop persoonlijke informatie wordt verzameld en opgeslagen is veilig (veilige dataverzameling en -opslag).	Onduidelijk, door gebrek aan informatie hierover.
Het aantal personen dat toegang heeft tot persoonlijke informatie wordt tot een minimum beperkt (minimale toegang tot de dataverzameling).	Onduidelijk, door gebrek aan informatie hierover.
De persoonlijke informatie die is verzameld, wordt alleen gebruikt voor het doel waarvoor deze is verzameld (doelbinding van datagebruik).	Onduidelijk, door gebrek aan informatie hierover.
Personen die kennis hebben van persoonlijke informatie gaan daar vertrouwelijk mee om (vertrouwelijkheid van persoonlijke informatie).	Onduidelijk, door gebrek aan informatie hierover.
De personen die toegang hebben tot persoonlijke informatie zijn zoveel mogelijk onafhankelijk van de personen waarover informatie wordt verzameld (dataverzameling en –opslag onafhankelijk van privéleven en werk).	Onduidelijk, door gebrek aan informatie hierover (in de media wordt de indruk gewekt dat de informatie wordt verzameld door medewerkers die in dienst zijn van Clarian en dus niet volledig onafhankelijk zijn).

Op het punt van aantasting van de privacy kan het preventieplan van Clarian op basis van de door mij bestudeerde teksten niet worden gerechtvaardigd. De reden is onduidelijkheid over de mate waarin de privacy wordt geschonden (zie overzicht 13). Het is dan niet mogelijk om de aantasting van de privacy te wegen tegen de effectiviteit van de preventiemaatregel. Op basis van de reacties in de media over de aantasting van de privacy van werknemers kan in ieder geval worden geconcludeerd dat Clarian in de externe communicatie over de privacybescherming tekort is geschoten:

> *"While some employees were supportive, there's been "the other reaction that this is very personal," says Wantz*[11], with people asking, "How dare you? This is my personal space." There's been a lot of questions and confusion." *(BusinessWeek, McGregor, 2007)*

> *"These programs are highly invasive", he said. "They regulate private behavior. They amass huge amounts of health information. What's happening to this information? What type of employees' private behavior is being regulated? …"* (MSNBC.com. TodayShow).

4. Indringendheid van de aantasting van veiligheidsbeleving

De door mij bestudeerde teksten bevatten geen aanwijzing dat toepassing van het preventieplan van Clarian zou kunnen leiden tot aantasting van de veiligheidsbeleving.

5. Indringendheid van de discriminatie

Discriminatie heb ik gedefinieerd als ongelijke behandeling in gelijke gevallen. Volgens deze definitie is er sprake van discriminatie als mensen worden benadeeld ten opzichte van andere mensen en de reden hiervoor niet uitsluitend bestaat uit de (extra) schade, overlast of schending van morele opvattingen die zij veroorzaken. Volgens deze definitie is er bij het preventieplan van Clarian geen sprake van discriminatie van ongezonde mensen, die niet voldoen aan de gezondheidscriteria van Clarian. Er is weliswaar sprake van ongelijke behandeling, maar ook van ongelijke gevallen. De zorgkosten van mensen die niet voldoen aan de gezondheidscriteria van Clarian zijn gemiddeld genomen aantoonbaar (veel) hoger dan de mensen die wel aan deze criteria voldoen. Mensen die om medische redenen niet kunnen voldoen aan de gezondheidscriteria van Clarian, worden op basis van een doktersverklaring vrijgesteld van het betalen van de 'boetes'. Dat in het preventie-

11. Wantz is een woordvoerder van Clarian.

plan van Clarian rokers wel en alcoholisten geen boete hoeven te betalen voor hun ongezonde gedrag, valt in het kader van het model onder het criterium 'rechtsgelijkheid' (12e criterium), maar niet onder het aspect 'discriminatie' van het criterium 'indringendheid' (8e criterium).

Terugblikkend leidt het preventieplan van Clarian tot aantasting van de keuzevrijheid en mogelijk tot aantasting van de privacy en lichamelijk integriteit, maar niet tot discriminatie. Daarom vind ik het opmerkelijk dat in de door mij bestudeerde teksten juist de veronderstelde discriminatie zo dominant aanwezig is:

> *"Aside from the fact this practice is blatantly discriminatory (I dare them to show up in San Francisco with these standards) it is plainly punative and instituted solely to punish people who are fat."* (Big Fat Blog, jportnick, August 9, 2007)]
>
> *"I am not a lawyer, but articles like this make me wish I was. To me, it seems that already-existing discrimination law *could* be used to sue these companies."* (Big Fat Blog, Stefanie bee, September 7, 2007)
>
> *"This is absurd, you can't discriminate in this manner wake up to educating and helping your employees. I am an RNY GB patient and know how it feels to be discrim[in]ated."* (MSNBC, Salwan, August 11, 2007)
>
> *"…their attempts to do so will inevitably lead to discrimination lawsuits."* (MSNBC, kingsXrulz, August 12, 2007)
>
> *"Yale university professor Rebecca Puhl studies how society treats the obese and she says "these types of punitive programs are a form of weight discrimination.""* (NBC5, 2007)

6. Indringendheid van de stigmatisering

Stigmatisering heb ik gedefinieerd als onevenredig ongelijke benadering in ongelijke gevallen. Het woord 'benadering' duidt op de wijze waarop mensen tegemoet worden getreden in het dagelijks leven. Mensen die worden gestigmatiseerd, worden op basis van een afwijkend kenmerk stelselmatig met minachting of negatief door anderen benaderd. Hierbij kan worden gedacht aan het belachelijk maken van mensen (openlijk of achter hun rug grappen over hun afwijkende kenmerk maken), het kleineren van mensen ("Heb jij Dikkie Dik gezien in zijn zwembroek?"), het afwijzen van mensen ("Wij willen jou niet in ons voetbalteam, want jij bent te dik."), het uitschelden van mensen ("Vetzak", "vetklep", "dikzak", "bolle"), enz. De kans dat het preventieplan van Clarian bijdraagt aan stigmatisering van mensen die ongezond eten, te dik zijn en/of roken, acht ik vrij groot. Uit de reacties op internet blijkt in ieder geval dat verschillende mensen vrezen voor (verdere) stigmatisering ten gevolge van preventieplannen zoals die van Clarian:

> *"Health care is what people who care for each other do for each other when someone is sick or injured...and this society does not care about those whose BMI's do not fall within a "normal" range. If parents have 3 children and one is fat, they love that one the least. Society feels the same."* (Big Fat Blog, Skorch, August 21, 2007)
>
> *""There are better ways of addressing this issue than imposing additional prejudice and penalties on this population," she said."* (NBC5, 2007)
>
> *"We never cease to be amazed at how the obese are picked on, yet are 2/3 of the population. No one wants to be obese or morbidly obese and hard workers should not be penalized or embarrassed by being singled out this way. Everyone should be outraged by such dehumanizing and an invasion of something as personal as what the scale says."* (MSNBC. Parish Brandon. August 10, 2007)

In haar uitingen in de media geeft Clarian er geen blijk van een bezwaar te zien tegen stigmatisering van mensen met een ongezonde leefstijl en aandacht te besteden aan het voorkomen hiervan. Ik zie dit als een tekortkoming van het preventieplan van Clarian.

Beoordeling van de mate van drang of dwang waarmee de preventiemaatregel wordt toegepast

Door werknemers die niet voldoen aan de gezondheidscriteria van Clarian financieel te korten op de vergoeding die Clarian betaalt voor hun zorgverzekering, oefent Clarian drang uit op de werknemers om zich wel aan deze criteria te conformeren. Om te voldoen aan de Amerikaanse anti-discriminatiewetgeving heeft Clarian de 'boete' voor werknemers in het kader van haar preventieplan gemaximeerd op $25 per tweewekelijks loonstrookje en daarmee op $650 per jaar. De Amerikaanse wetgeving maakt het uitoefenen van deze vorm van drang door werkgevers op werknemers expliciet mogelijk. Zie bijlage 8 voor een toelichting op deze wetgeving (Toelichting op §f van de HIPAA).

Een zorgverzekering kan worden beschouwd als een noodzakelijke voorziening. Als een werknemer zich het betalen van de jaarlijkse maximale toeslag financieel niet kan permitteren, wordt deze werknemer min of meer gedwongen om zijn leefstijl te veranderen, omdat het onverantwoord is om de zorgverzekering op te zeggen. Dan is er dus geen sprake meer van drang, maar van dwang tot verandering van leefstijl. Daar de betaling van de toeslagen gespreid wordt over de tweewekelijkse loonstrookjes, zal bijna iedere werknemer de toeslagen echter kunnen betalen. De door mij bestudeerde teksten geven geen informatie over de inkomsten en vaste lasten van de werknemers van Clarian, waardoor dit niet met zekerheid is vast te stellen:

> “*Yes, I do feel like this is punishment because I really can't afford to pay," said LaTonya Johnson, another Clarian employee.*” (NBC5, 2007)

Op basis van de beschikbare informatie over de hoogte van de toeslagen kan er het best worden gesproken over het uitoefenen van sterke drang op werknemers, die ertoe moet leiden dat zij zich conformeren aan de gezondheidscriteria van Clarian. Op de door mij bestudeerde weblogs zien diverse mensen in deze sterke drang echter geen bezwaar:

> “*Pay ALL of your health care costs and don't leave it to anyone else to pick up the tab for your irresponsible and preventable behaviors. But as long as someone else (your employers, your coworkers) are shouldering some of that risk for you, expect to be held accountable for your actions in the form of increased premiums and a loss of personal freedom.*” (MSNBC, August 11, 2007)

Het verbaast mij dat de federale Amerikaanse overheid (Unites States Department of Labour) besluitvorming over de toepassing van sterke drang ter bevordering van de volksgezondheid en/of bestrijding van de kosten van ongezondheid toevertrouwt aan afzonderlijke werkgevers. De indringendheid van de preventiemaatregel, zoals beschreven in deze paragraaf (§7.2.2), toont m.i. aan dat deze keuze van de Amerikaanse overheid zeer discutabel is.

7.2.3 Eindoordeel (criterium indringendheid)

Bij het criterium ‘indringendheid’ (8e criterium) gaat het om de aanvaardbaarheid van de verhouding tussen bepaalde lasten en bepaalde baten van de preventiemaatregel, waarbij de lasten worden gevormd door de indringendheid en de baten door de effectiviteit. Is de verhouding tussen de indringendheid en effectiviteit van de preventiemaatregel aanvaardbaar? Bij het beantwoorden van deze vraag voor de casus Clarian Health maak ik onderscheid tussen twee groepen werknemers:

- groep 1: de werknemers die wel aan alle gezondheidscriteria van Clarian voldoen;
- groep 2: de werknemers die niet aan alle gezondheidscriteria van Clarian voldoen.

De werknemers die niet aan alle gezondheidscriteria van Clarian voldoen, maar die met een doktersverklaring kunnen worden vrijgesteld van een ‘boete’ vallen ten behoeve van mijn analyse onder groep 1. *5Groep 1*

Voor werknemers die wel aan alle gezondheidscriteria van Clarian voldoen, acht ik de indringendheid van het preventieplan niet te rechtvaardigen. Niet te rechtvaardigen acht ik het gegeven dat zij $650 (maximale jaarlijkse boete) moeten betalen, als zij willen voorkomen dat hun werkgever hun lichamelijke integriteit en privacy

aantast. De werknemers van groep 1 zijn immers niet verantwoordelijk voor de zorgkosten die Clarian met haar preventieplan wil bestrijden. De indringendheid van de preventiemaatregel (structurele aantasting van hun lichamelijke integriteit en privacy) vind ik daarom zwaarder wegen dan de door de werkgever beoogde kostenreductie. De door de werkgever beoogde gezondheidswinst acht ik bij de afweging van de indringendheid tegen de effecten van de preventiemaatregel niet relevant. De reden is dat de beoogde gezondheidswinst een paternalistische doelstelling betreft (zie onder het criterium ‘verantwoordelijkheid’).

Alle werknemers kunnen vrij kiezen om al dan niet deel te nemen aan het preventieplan, als van tevoren vaststaat dat de zorgpremie ten gevolge van het preventieplan voor alle werknemers met tenminste $650 per jaar zal dalen (of minder zal stijgen). Dat dit zal gebeuren, acht ik echter vrijwel uitgesloten. Enerzijds omdat de beoogde kostenreductie ten gevolge van het preventieplan in principe alleen wordt opgebracht door de werknemers van groep 2. Mogelijk is dit minder dan de helft van de werknemers van Clarian[12]. Anderzijds omdat het onwaarschijnlijk lijkt dat de zorgkosten van de werknemers in groep 2 ten gevolge van het preventieplan gemiddeld met meer dan $650 per jaar zullen dalen (of minder stijgen).[13]

Groep 2

De door de werkgever beoogde kostenreductie zou zwaarder kunnen wegen (zie de vorige voetnoot) dan de indringendheid van de preventiemaatregel, als de indringendheid van de preventiemaatregel kwantitatief wordt gewaardeerd op maximaal $650 per jaar (de maximale jaarlijkse ‘boete’). Een bezwaar hiertegen is dat sommige mensen de maximale ‘boete’ financieel misschien niet kunnen opbrengen, terwijl de ‘indringendheid’ van de preventiemaatregel voor hen een (veel) grotere waarde dan $650 vertegenwoordigt. Op basis van de beschikbare informatie kan ik niet concluderen dat de indringendheid van de preventiemaatregel voor groep 2 zwaarder weegt dan de beoogde kostenreductie. Geredeneerd volgens het falsificatiebeginsel kan het preventieplan van Clarian voor groep 2 daarom niet worden afgewezen op basis van het criterium ‘indringendheid’ van het model. Men kan hier tegenin brengen dat de mate waarin het preventieplan de lichamelijke integriteit en privacy aantast onduidelijk is. Daardoor is het niet mogelijk om de aantas-

12. Clarian schat dat momenteel 34% van haar werknemers obesitas heeft en dat 26% rookt (BusinessWeek, McGregor, 2007).
13. “*… total annual healthcare costs per person were similar for normal [BMI 18,5-24,9] and overweight [BMI 25,0-29,9] individuals ($3,254 and $3,201, respectively) … Moderate obesity [BMI 30,0-34,9] … was associated with a $670 increase in costs (to $3,925). Severe obesity [BMI >35,0] ... was associated with a $2,441 increase (to $5,695), as compared with costs for persons of normal weight.*” (Huse, 2007, p.3)

ting van de lichamelijke integriteit en privacy te wegen tegen de effectiviteit van de preventiemaatregel. Zo bezien kan de ‘indringendheid’ van de preventiemaatregel voor groep 2 pas gerechtvaardigd worden, als preciezere informatie over de aantasting van de lichamelijke integriteit en privacy beschikbaar komt.

7.3 Negende criterium: kosteneffectiviteit

7.3.1 *Operationalisering en wijze van beoordeling*

Operationalisering

Het criterium ‘kosteneffectiviteit’ betreft de verhouding tussen de uitvoeringskosten en de beoogde effecten van de interventie (zie de toelichting op rij II en III van overzicht 2 in §2.6.2). Als de beoogde effecten en uitvoeringskosten van de preventiemaatregel op dezelfde wijze worden gekwantificeerd, c.q. worden uitgedrukt in geld, kan de kosteneffectiviteit worden berekend door de waarde van de beoogde effecten te delen door de waarde van de uitvoeringskosten. Als de uitkomst van deze deling groter is dan één, dan is de maatregel kosteneffectief. De beoogde effecten zijn reeds in kaart gebracht bij het criterium ‘effectiviteit’. De uitvoeringskosten betreffen alle kosten die men moet maken om de preventiemaatregel te kunnen uitvoeren.

De duurzaamheid van de beoogde effecten en van de uitvoeringskosten vormen een belangrijk aandachtspunt. Het is denkbaar dat de implementatie van een preventiemaatregel hoge startkosten heeft en dat de uitvoeringskosten in de loop van de tijd afnemen, zonder dat de beoogde effecten navenant afnemen. Dit zou bijvoorbeeld het geval kunnen zijn bij de introductie van nieuwe wetgeving, zoals een rookverbod in de Horeca, waarbij de handhavingskosten in de loop van de tijd kunnen verminderen. Met dit voorbeeld raak ik gelijk een moeilijk punt. Met name in het geval van ge- of verbodswetgeving kunnen de handhavingskosten in grote mate bepalend zijn voor de uitvoeringskosten. Op hun beurt worden de handhavingskosten in grote mate bepaald door het draagvlak voor de preventiemaatregel. Het draagvlak voor de preventiemaatregel wordt echter pas bij het 13e criterium ‘draagvlak’ van het model beoordeeld. Bij het bepalen van de kosteneffectiviteit is het daarom van belang dat men zich er rekenschap van geeft in welke mate het draagvlak voor de preventiemaatregel van invloed is op eventuele handhavingskosten. Op basis van welke handhavingskosten de kosteneffectiviteit is bepaald, dient te worden geëxpliciteerd.

Wijze van beoordeling
Bij de toetsing van de preventiemaatregel aan het criterium 'kosteneffectiviteit' wordt beoordeeld of de preventiemaatregel kosteneffectief is en hoe de kosteneffectiviteit zich in de loop van de tijd ontwikkelt. De implementatie van preventiemaatregelen die niet kosteneffectief zijn, is niet gerechtvaardigd. Hieraan ligt het argument ten grondslag dat het geld nuttiger kan worden besteed. Tevens wordt beoordeeld of de informatie over de kosteneffectiviteit volledig, voldoende gedetailleerd en voldoende gekwantificeerd is. Als dat oordeel negatief uitvalt, wordt beoordeeld of de informatieverzameling over de kosteneffectiviteit voldoende is geweest.

7.3.2 Beoordeling van casus Clarian Health aan de hand van het criterium 'kosteneffectiviteit'

Het onderwerp kosteneffectiviteit is opvallend afwezig in de door mij bestudeerde documenten. Er is volstrekt onvoldoende informatie beschikbaar om de kosteneffectiviteit objectief te kunnen beoordelen. Het terugdringen van financiële schade is een hoofddoelstelling van Clarian. Pragmatisch geredeneerd kan daarom worden verondersteld, dat de uitvoeringskosten die Clarian verwacht (veel) lager liggen dan de beoogde (financiële) effecten. Clarian lijkt er hierbij van uit te gaan dat haar werknemers de uitvoering van het preventieplan niet zullen traineren of saboteren.

7.3.3 Eindoordeel (criterium kosteneffectiviteit)

Er is volstrekt onvoldoende informatie beschikbaar om de kosteneffectiviteit objectief te kunnen beoordelen. Naar verwachting is de preventiemaatregel van Clarian kosteneffectief.

7.4 Tiende criterium: lasten-batenverhouding

7.4.1 Operationalisering en wijze van beoordeling

Operationalisering
Het criterium 'lasten-batenverhouding' betreft de verhouding tussen alle baten en lasten van de preventiemaatregel (zie overzicht 14).

Overzicht 14: criterium lasten-batenverhouding

Lasten-batenverhouding	
**Alle lasten van de preventiemaatregel**	**Alle baten van de preventiemaatregel**
Inbreuk van de preventiemaatregel op het persoonlijk leven (zie criterium 'indringendheid')	Beoogde effecten van de preventiemaatregel (zie criterium 'effectiviteit')
Uitvoeringskosten van de preventiemaatregel (zie criterium 'kosteneffectiviteit')	
Overige lasten van de preventiemaatregel	Overige baten van de preventiemaatregel (alle positieve neveneffecten)

Alleen de overige lasten en baten (grijs gearceerd) zijn nog niet geanalyseerd. De andere lasten en baten zijn reeds geanalyseerd in het kader van de voorafgaande criteria 'effectiviteit', 'indringendheid' en 'kosteneffectiviteit'. Als alle lasten en baten op dezelfde wijze worden gekwantificeerd, c.q. worden uitgedrukt in geld, kan de lasten-batenverhouding worden berekend door de waarde van alle baten te delen door de waarde van alle lasten. Als de uitkomst van deze deling groter is dan één, dan is de lasten-batenverhouding gunstig. De duurzaamheid van de baten en lasten vormt een belangrijk aandachtspunt. Ook als het niet lukt om alle lasten en baten te kwantificeren, is het toch nuttig om alle lasten en baten in één overzichtelijk schema te plaatsen (Ogus, 2010):

> *"For the analytical framework, I propose to articulate the benefits and costs of paternalist interventions, although these will not be easy to quantify. Even in the absence of concrete figures, setting up a benefit–cost framework facilitates clear thinking about policy options.* (p.69) … *My benefit–cost analytical framework is not intended as a model which can easily reach definitive results, since quantification of many of the variables will be problematic. But it does suggest a useful way of ordering the relevant issues in a way which should facilitate good policy making."* (p.72)

Wijze van beoordeling

Bij de toetsing van de preventiemaatregel aan de hand van het criterium 'lasten-batenverhouding' wordt beoordeeld of deze verhouding gunstig is en hoe deze verhouding zich in de loop van de tijd ontwikkelt. De implementatie van preventiemaatregelen met een ongunstige lasten-batenverhouding is niet gerechtvaardigd. Hieraan ligt het argument ten grondslag dat het geld nuttiger kan worden besteed. Tevens wordt beoordeeld of de beschikbare informatie over de lasten-batenverhouding volledig, voldoende gedetailleerd en voldoende gekwantificeerd is. Als dat oordeel negatief uitvalt, wordt beoordeeld of de informatieverzameling over de lasten-batenverhouding voldoende is geweest.

In theorie is het mogelijk dat de beoordeling van de preventiemaatregel aan de hand van de criteria 'effectiviteit' en 'indringendheid' ongunstig uitvalt, terwijl de lasten-batenverhouding toch gunstig uitvalt. Dat kan het geval zijn als de positieve neveneffecten zeer groot zijn. M.i. is het onredelijk om een ongunstige beoordeling van de 'effectiviteit' en 'indringendheid' te compenseren met een gunstige beoordeling van de 'lasten-batenverhouding'. In plaats van de beoogde effecten vormen dan de positieve neveneffecten de basis voor de rechtvaardiging van de preventiemaatregel en dat is niet conform de doelstelling van de preventiemaatregel. Als men in dat geval toepassing van de preventiemaatregel vanwege de positieve neveneffecten toch wil rechtvaardigen, is een mogelijke oplossing om de positieve neveneffecten aan de doelstelling van de preventiemaatregel toe te voegen en de preventiemaatregel vanaf het 1e criterium opnieuw te beoordelen.

7.4.2 Beoordeling van casus Clarian Health aan de hand van criterium 'lasten-batenverhouding'

Alle lasten van de preventiemaatregel

Alle lasten van de preventiemaatregel betreffen (zie overzicht 14):

- inbreuk van de preventiemaatregel op het persoonlijk leven (zie criterium 'indringendheid');
- uitvoeringskosten van de preventiemaatregel (zie criterium 'kosteneffectiviteit');
- overige lasten van de preventiemaatregel (negatieve neveneffecten, exclusief inbreuk op het persoonlijk leven en exclusief uitvoeringskosten).

De 'inbreuk van de preventiemaatregel op het persoonlijk leven' en de 'uitvoeringskosten van de preventiemaatregel' heb ik bij voorafgaande criteria al in kaart gebracht en beoordeeld. Ten behoeve van het criterium 'lasten-batenanalyse' zal ik daarom in aanvulling hierop alleen de 'overige lasten van de preventiemaatregel' in kaart brengen en beoordelen:

- Voor de werkgever wordt het werven en behouden van personeel moeilijker:

  "People are not interested in making their employees unhappy," says Helen Darling, president of the National Business Group on Health, a nonprofit that represents large employers' health-care interests. "We're in a war for talent, … " (BusinessWeek, McGregor, 2007)

  "In my opinion if I were an employee of Clarian Health I would have a new job by 2009 fat or not fat...smoker or no smoker." (MSNBC, Casey, Hartselle, August 10, 2007)

– Medewerkers kunnen gedemotiveerd raken door negatieve incentives, wat ten koste kan gaan van hun productiviteit:

"… a price tag on your weight loss is not a way to motivate employees." (MSNBC, Casey, Hartselle, August 10, 2007)

– Medewerkers kunnen 'wraak' nemen door hun productiviteit te verminderen:

"But if I worked there and these new rules took effect, I think I'd slow my output in direct relationship to the amount the company is pinching from my paycheck." (MSNBC, Doug Wilson, August 12, 2007)

– Medewerkers kunnen (denken) in financiële problemen (te) raken:

"Yes, I do feel like this is punishment because I really can't afford to pay," said LaTonya Johnson, another Clarian employee." (NBC5, 2007)

– Klanten kunnen Clarian gaan boycotten:

"I will make sure not to purchase from Dell IBM Kelloggs and any of the other companies who do this to their employees." (MSNBC, S Mck, August 11, 2007)

– Er kan jaloezie en animositeit tussen medewerkers onderling en medewerkers en management ontstaan, wat ten koste kan gaan van de werksfeer en samenwerking (in teams):

"Incentives are better received in the workforce," Slutzky says. "Ultimately, what you're trying to do is improve camaraderie. …." (Workforce Management, Wojcik, 2007)

Voor alle bovengenoemde 'overige lasten' geldt, dat deze lasten (als ze ontstaan) waarschijnlijk structureel (duurzaam) zijn en niet eenmalig.

Alle baten van de preventiemaatregel

Alle baten van de preventiemaatregel betreffen (zie overzicht 14):

– beoogde effecten van de preventiemaatregel (zie criterium 'effectiviteit');
– overige baten van de preventiemaatregel (alle positieve neveneffecten).

De 'beoogde effecten van de preventiemaatregel' heb ik bij het voorafgaande criterium 'effectiviteit' al in kaart gebracht en beoordeeld. Ten behoeve van het criterium 'lasten-batenanalyse' zal ik daarom in aanvulling hierop alleen de 'overige baten van de preventiemaatregel' in kaart brengen en beoordelen:

– Stijging van de productiviteit door fittere werknemers met een lager ziekteverzuim:

  "… I'm sure they'd all love to have a workforce comprised of only young, thin, childless, pretty, perfectly healthy and able-bodied employees with no lives whatsoever who just live to work, work, work and don't care about anything else. …" (Big Fat Blog, Meowzer, August 10, 2007)

– Verbetering van de mentaliteit van de werknemers, hun gezinnen en andere mensen:

  "Responsibility and accountability. Hard to do but it is ultimately what will make us better people and a stronger country." (MSNBC, Alyssa P, August 10, 2007)

Voor alle bovengenoemde 'overige baten' geldt, dat deze baten (als ze ontstaan) waarschijnlijk structureel (duurzaam) zijn en niet eenmalig.

Het doel van het toetsen van de casus Clarian aan het model, is het beoordelen van de juistheid, volledigheid en praktische toepasbaarheid van het model en niet het beoordelen van de gerechtvaardigdheid van het preventieplan van Clarian. Daarom laat ik het achterwege om de 'overige lasten en baten' zoveel mogelijk te kwantificeren. Deze kwantificering kan ik namelijk niet rechtstreeks uit de door mij bestudeerde teksten over het preventieplan van Clarian halen.

7.4.3 Eindoordeel (criterium lasten-batenverhouding)

Ook als het niet lukt om alle lasten en baten te kwantificeren, is het toch nuttig om alle lasten en baten in één overzichtelijk schema te plaatsen (Ogus, 2010, p.69 en 72). Zie overzicht 15.

Overzicht 15 bevat de nodige vraagtekens, omdat de door mij bestudeerde teksten over de casus Clarian over de betreffende onderwerpen onduidelijk of niet overtuigend zijn. Bij het onderwerp 'aantasting van de privacy' heb ik geen vraagteken ingevuld, terwijl toch onduidelijk is in welke mate de privacy precies wordt aangetast. De reden is dat het wel evident is dat het preventieplan van Clarian de privacy substantieel aantast.

Overzicht 15: lasten-batenverhouding van het preventieplan van Clarian

**Lasten-batenverhouding**	
**Alle lasten van de preventiemaatregel**	**Alle baten van de preventiemaatregel**
Inbreuk op het persoonlijk leven: substantieel<br>– aantasting lichamelijke integriteit: ja?<br>– aantasting vrijheid: ja<br>– aantasting privacy: ja<br>– aantasting veiligheidsbeleving: nee<br>– discriminatie: nee<br>– stigmatisering: ja	***Beoogde effecten: mogelijk substantieel***<br>– beperking gezondheidsschade: ja?<br>– beperking financiële schade: ja
Uitvoeringskosten: naar verwachting beperkt t.o.v. verwachte effecten	
Overige lasten: mogelijk substantieel<br>– moeilijker werven en behouden personeel: ja?<br>– demotivering en 'wraak' werknemers en daardoor daling productiviteit: ja?<br>– financiële problemen van werknemers: ja?<br>– boycot van Clarian door klanten: ?<br>– negatieve invloed op werksfeer en samenwerking: ja?	***Overige baten: mogelijk substantieel***<br>– fitter personeel en minder ziekteverzuim en daardoor stijging productiviteit: ja?<br>– positieve mentaliteitsverandering binnen en buiten het bedrijf: ?

De overige lasten en baten kunnen m.i. substantieel zijn. Met name de effecten van het preventieplan op de productiviteit (inclusief het ziekteverzuim) zouden een grote invloed kunnen hebben op de lasten-batenverhouding. In de wetenschappelijke literatuur is er bijvoorbeeld bewijs dat obesitas leidt tot een verlaagde productiviteit[14]. Een verhoogde productiviteit ten gevolge van het preventieplan treedt echter alleen op als het preventieplan leidt tot een gezondere leefstijl en dat moet nog blijken in de praktijk.

Daar de overige lasten en baten substantieel kunnen zijn, is hierover meer informatie nodig, voordat het preventieplan gerechtvaardigd kan worden op basis van het criterium lasten-batenverhouding. Dit is nog steeds het geval, als het preventieplan wel gerechtvaardigd zou kunnen worden op basis van de criteria 'effectiviteit', 'indringendheid' en 'kosteneffectiviteit' (wat wegens een gebrek aan informatie op dit moment niet het geval is).

14. *"Conclusions: The relationship between BMI end presenteeism is characterized by a threshold effect, where extremely or moderately obese workers are significantly less productive than mildly obese workers."* (Gates, 2008, p.39)

7.5 Elfde criterium: rechtvaardigheid

7.5.1 Operationalisering en wijze van beoordeling

Operationalisering

Het criterium 'rechtvaardigheid' betreft de distributieve rechtvaardigheid. De distributieve rechtvaardigheid betreft de vraag of de baten en lasten van de preventiemaatregel rechtvaardig over de bij de preventiemaatregel betrokken partijen – mensen en organisaties – worden verdeeld. De vergelijking tussen enerzijds de baten en lasten voor bij de preventiemaatregel betrokken partijen en anderzijds de baten en lasten voor niet bij de preventiemaatregel betrokken partijen, vindt plaats bij het criterium 'rechtsgelijkheid'. Het criterium 'rechtsgelijkheid' betreft bijvoorbeeld de vraag of het wel rechtvaardig is dat het preventieplan van Clarian rokers wel financieel straft, maar alcoholisten niet.

De distributieve rechtvaardigheid kent de volgende aspecten:

1. ***Aspect van verantwoordelijkheid***
   In hoeverre worden de lasten van de preventiemaatregel over partijen verdeeld naar rato van hun verantwoordelijkheid voor de risico's die men met de preventiemaatregel tracht te bestrijden? Het principe achter dit aspect van rechtvaardigheid is: 'de vervuiler betaalt'. Volgens dit principe is het:
   - rechtvaardig dat men een groter (respectievelijk kleiner) deel van de lasten van de preventiemaatregel draagt, naarmate men verantwoordelijk is voor een groter (respectievelijk kleiner) deel van de risico's die men tracht te bestrijden;
   - onrechtvaardig als de lasten van de preventiemaatregel terechtkomen bij partijen die niet verantwoordelijk zijn voor de risico's die men tracht te bestrijden. Als dit toch het geval is, dienen deze partijen hiervoor te worden gecompenseerd door de baten van de preventiemaatregel en of door een (financiële) tegemoetkoming.
   - onrechtvaardig als men voor hetzelfde 'vergrijp' (hetzelfde ongezonde gedrag) meer dan eenmaal wordt 'gestraft'. Hiervan kan sprake zijn als er een correlatie bestaat tussen de gezondheidsindicatoren op basis waarvan de 'boete' voor ongezond gedrag wordt vastgesteld; in het preventieplan van Clarian bijvoorbeeld de correlatie tussen roken en een hoge bloeddruk. Als men (mede) ten gevolge van roken een te hoge bloeddruk heeft, wordt men twee keer beboet voor hetzelfde ongezonde gedrag: direct door een boete voor roken en indirect door een boete voor een te hoge bloeddruk.

2. *Aspect van gelijke behandeling*

In hoeverre worden partijen in absolute zin (zonder rekening te houden met draagkracht) gelijkelijk benadeeld of bevoordeeld door de preventiemaatregel?

> *"… it's important that an incentive program is equitable to all employees. Those who are already making responsible life choices deserve the same rewards and support as those who are just starting."* (Hall, 2008, p.15)

3. *Aspect van draagkracht*

In hoeverre houdt de preventiemaatregel rekening met de draagkracht van mensen (lichamelijk, psychisch, sociaal en financieel) en organisaties (organisatorisch, financieel en economisch)?

- Voor mensen met een hoog inkomen is dezelfde financiële boete minder belastend dan voor mensen met een laag inkomen. Zo bezien zou de hoogte van een boete inkomensafhankelijk moeten worden vastgesteld:

  > *"Fair magnitude of penalty. … Penalty amounts scaled to personal salary."* (Pearson, 2009, p.847)

- Gaan de lasten van de preventiemaatregel de draagkracht van partijen niet te boven? Het is belangrijk om hierbij oog te hebben voor een mogelijke stapeling van lasten bij bepaalde partijen:

  > *"As with smoking, social justice issues are prominent, given that obesity rates are highest in the poorest segments of the population."* (Brownell, 2009, p.261 op basis van Kumanyika, 2006)

- Men kan ook stellen dat het onrechtvaardig is, als in een samenleving de (financieel) zwakste partijen de zwaarste (financiële) lasten te dragen krijgen.

  > *"In the United States, many racial/ethnic minority populations are socially and politically disadvantaged […] and generally have higher rates of nutrition-related health problems than the majority population."* (Kumanyika, 2006, p.S9)

  Dit aspect van (on)rechtvaardigheid behandel ik bij het volgende criterium van het model: het criterium 'rechtsgelijkheid'.

- Staan de lasten van de preventiemaatregel, wanneer deze worden gewogen aan de hand van de draagkracht van partijen, nog in verhouding tot de risico's waarvoor men verantwoordelijk is?

4. ***Aspect van hardheid in specifieke gevallen***
   In hoeverre houdt de preventiemaatregel rekening met de persoonlijke omstandigheden van mensen of specifieke omstandigheden van organisaties?
   – In hoeverre houdt de preventiemaatregel rekening met persoonlijke omstandigheden van mensen?

     "Moreover, there was no apparent appeals process in place that would permit employees to opt out of the penalty program on medical or personal grounds." (Pearson, 2009a)

   – In hoeverre houdt de preventiemaatregel rekening met specifieke omstandigheden van organisaties? Denk bijvoorbeeld aan een rookverbod in de Horeca. Grote cafés kunnen wel een inpandige rookruimte realiseren, maar kleine cafés kunnen dat wegens ruimtegebrek niet, waardoor er sprake is van concurrentievervalsing.
   – Kent de preventiemaatregel een hardheidsclausule?

5. ***Aspect van gelijke toegang tot gezondheidszorg en preventie***
   Welke invloed heeft de preventiemaatregel op de gelijke kansen van betrokken partijen wat betreft de toegang tot gezondheidszorg en preventie?

   "Fair administration of penalty programs: … Fair magnitude of penalty … not as tools to drive low-income/ at-risk workers out of their health insurance" (Pearson, 2009, 850-851)

6. ***Aspect van verdeling naar behoefte van gezondheidszorg en preventie***
   Welke invloed heeft de preventiemaatregel op de verdeling van de voor gezondheidszorg en preventie beschikbare middelen naar behoefte van betrokken partijen?

   De toegang tot gezondheidszorg kan weliswaar gelijk zijn, maar de zorg waar men toegang toe heeft (waarvoor men verzekerd is) kan voor de ene partij genoeg en voor de andere partij te weinig zijn.

Er bestaat een zekere overlap tussen de bovenstaande aspecten van rechtvaardigheid. Voor het beoordelen van de distributieve rechtvaardigheid van de preventiemaatregel is dat geen bezwaar. Enige overlap tussen de aspecten die worden beoordeeld, maakt de kans dat alle aspecten van de distributieve rechtvaardigheid worden beoordeeld eerder groter dan kleiner.

Wijze van beoordeling (zie de bespreking van cluster 6 in §3.3)
Uit een literatuur-review in een discussienota t.b.v. projecten van de Wereldbank worden maar liefst zeven concepten van rechtvaardigheid genoemd (Hauck, 2004, 23-36):

1. Egalitarianism;
2. Allocation according to need;
3. Rule of rescue;
4. Equality of access;
5. Providing a decent minimum;
6. Rawls' maximin principle;
7. Libertarianism.

Binnen de wetenschap en samenleving bestaat nog geen consensus hoe deze concepten van rechtvaardigheid bij de beoordeling van de verdeling van kosten en baten van preventiemaatregelen tegen elkaar gewogen moeten worden (Oortwijn, 2006, p.142) (Hauck, 2004, p.33-34). Afhankelijk van het gewicht dat men, op basis van morele en/of politiek-ideologische overwegingen, aan verschillende concepten van rechtvaardigheid toekent, leidt de beoordeling van de distributieve rechtvaardigheid van de preventiemaatregel tot andere uitkomsten. Bij de bespreking van de 'wijze van toetsing' van het criterium 'gerichtheid' heb ik gesteld, dat het in het algemeen eenvoudiger is om vast te stellen dat er geen overtuigende argumenten tegen een stelling of theorie zijn, dan overtuigend te beargumenteren dat een stelling of theorie juist is. Van dit principe (afgeleid van het falsificatiebeginsel) maak ik ook gebruik bij de beoordeling van de 'distributieve rechtvaardigheid' van de preventiemaatregel. Beoordeeld wordt of toepassing van de preventiemaatregel vanuit het perspectief van de distributieve rechtvaardigheid niet evident onredelijk of onbillijk is.

7.5.2 Beoordeling van casus Clarian Health aan de hand van criterium 'rechtvaardigheid'

Aspect van verantwoordelijkheid

– Lasten naar rato van de risico's waarvoor men verantwoordelijk is

> *"Clarian, an Indiana hospital chain, will dock $5 off worker paychecks for smoking, $10 off for being overweight, and $5 each for high cholesterol, high blood sugar, and high blood pressure."* (MSNBC, 2007)

Als men alleen overgewicht heeft, leidt dat tot een jaarlijkse boete van 12 x $10 = $120. Het onderstaande citaat geeft een indicatie van de meerkosten op het gebied van zorg ten gevolge van overgewicht:

"… total annual healthcare costs per person were similar for normal [BMI 18,5-24,9] and overweight [BMI 25,0-29,9] individuals ($3,254 and $3,201, respectively) … Moderate obesity [BMI 30,0-34,9] … was associated with a $670 increase in costs (to $3,925). Severe obesity [BMI >35,0] ... was associated with a $2,441 increase (to $5,695), as compared with costs for persons of normal weight." (Huse, 2007, p.3)

Clarian brengt een boete in rekening bij een BMI > 30. Als personeelsleden voor 50% verantwoordelijk worden gehouden voor hun overgewicht, zouden zij voor 50% verantwoordelijk kunnen worden gehouden voor hun meerkosten op het gebied van zorg ten gevolge van overgewicht. Volgens het bovenstaande citaat leidt een BMI tussen 30 en 35 tot meerkosten op het gebied van zorg van $670. De helft (50%) hiervan bedraagt $335. Zo bezien is een jaarlijkse boete van $120 niet onredelijk.

– Lasten voor partijen niet verantwoordelijk voor het risico op schade
Voor werknemers die wel aan alle gezondheidscriteria van Clarian voldoen, acht ik de indringendheid van het preventieplan niet te rechtvaardigen. Concreter geformuleerd: het gegeven dat zij $650 (maximale jaarlijkse boete) moeten betalen, als zij willen voorkomen dat hun werkgever hun lichamelijke integriteit en privacy aantast, acht ik niet te rechtvaardigen. Zij zijn immers niet verantwoordelijk voor de zorgkosten die Clarian met haar preventieplan wil bestrijden. (Zie het eindoordeel bij het criterium 'indringendheid'.)
– Niet meer dan eenmaal beboet worden voor hetzelfde ongezonde gedrag
De gezondheidsindicatoren op basis waarvan Clarian haar personeel boetes oplegt, zijn: overgewicht, te hoog glucosegehalte, te hoog cholesterolgehalte, te hoge bloeddruk, en roken. Ik veronderstel het algemeen bekend dat er een correlatie bestaat tussen enerzijds overgewicht en anderzijds een te hoog glucosegehalte, te hoog cholesterolgehalte, en een te hoge bloeddruk; en tevens tussen roken en een te hoge bloeddruk. Het doel van de beoordeling van de casus Clarian Health aan de hand van criterium 'rechtvaardigheid' is de beoordeling van de bruikbaarheid van het model en niet de beoordeling van de casus Clarian Health. Daarom werk ik de correlaties tussen de gezondheidsindicatoren die Clarian hanteert niet nader uit. Ik baseer mijn beoordeling van het preventieplan van Clarian op het vermoeden dat de gezondheidsindicatoren die Clarian hanteert voldoende onderscheidend zijn, om te rechtvaardigen dat iedere 'overtreding' afzonderlijk wordt beboet. Bovendien heeft Clarian de boete per personeellid gemaximeerd op $25 per loonstrookje. Daardoor blijft de onrechtvaardigheid van het meer dan eenmaal beboet worden voor hetzelfde ongezonde gedrag in kwantitatieve zin beperkt. Op basis van de bovenstaande overwegingen kom ik tot de volgende conclusie: de mate waarin mensen meer dan

eenmaal worden beboet voor hetzelfde ongezonde gedrag is niet evident onredelijk of onbillijk.

Aspect van gelijke behandeling

In hoeverre worden partijen in absolute zin (zonder rekening te houden met draagkracht) gelijkelijk benadeeld of bevoordeeld door de preventiemaatregel? Alle werknemers van Clarian worden over één kam geschoren. Directie en managers worden voor zover bekend niet uitgezonderd van het betalen van boetes als zij niet voldoen aan de gezondheidsindicatoren die Clarian heeft vastgesteld. Als de premie van de zorgverzekeringen ten gevolge van het preventieplan de komende jaren minder hard stijgt, profiteren de werknemers van Clarian daar in gelijke mate van. In absolute zin worden partijen dus gelijkelijk benadeeld en bevoordeeld door het preventieplan. Als Clarian door het preventieplan betere financiële resultaten behaalt en met name de directie en eventueel leidinggevenden worden hiervoor beloond, dan is er geen sprake meer van gelijke bevoordeling van partijen. Uit de door mij bestudeerde teksten blijkt echter niet dat hiervan sprake zou zijn.

Aspect van draagkracht

- Inkomensafhankelijkheid van boetes
  De boetes die Clarian oplegt, zijn niet inkomensafhankelijk (geen percentage van het inkomen, maar een vast bedrag per ‘overtreding’). Ze zijn lager dan verkeersboetes, die ook niet inkomensafhankelijk zijn. Zo bezien is het niet evident onredelijk en onbillijk dat boetes van dergelijke hoogte niet inkomensafhankelijk worden vastgesteld. De boetes worden echter wel iedere maand opgelegd, waardoor het boetebedrag kan oplopen tot $650 per werknemer per jaar. Een bezwaar van het verlagen van de boetes voor de lagere inkomensgroepen kan zijn, dat de prikkel tot gedragsverandering hierdoor kan verminderen. Een andere mogelijkheid is om de boetes voor de hogere inkomensgroepen te verhogen. De hoogte van de boetes dient dan wel op een bepaald bedrag te worden gemaximeerd, want anders kan een wanverhouding ontstaan tussen het risico op extra zorgkosten waarvoor men verantwoordelijk is en de boete die men hiervoor betaalt. Een inkomensafhankelijke regeling leidt in het algemeen tot (veel) meer administratieve lasten en daarmee kosten voor de werkgever. Als werknemers stellen dat het onrechtvaardig is dat de boeteregeling niet inkomensafhankelijk is, hebben zij een punt. M.i. is de omvang van deze onrechtvaardigheid echter niet zo groot dat het preventieplan van Clarian hierdoor als evident onredelijk of onbillijk bestempeld kan worden.
- Lasten groter dan draagkracht
  Het volgende citaat vormt een aanwijzing dat de boetes die Clarian oplegt de draagkracht van sommige werknemers te boven kan gaan:

"Yes, I do feel like this is punishment because I really can't afford to pay," said LaTonya Johnson, another Clarian employee. (NBC5, 2007)

Clarian lijkt hier geen oog voor te hebben en te redeneren dat mensen hun ongezonde gedrag dan maar moeten aanpassen. Wetenschappelijke evidence dat mensen dat ook kunnen, wordt echter niet door Clarian aangedragen. Voor mensen die de jaarlijkse boete van $650 echt niet kunnen betalen en die niet in staat zijn om hun ongezonde gedrag aan te passen (en het is niet uitgesloten dat die combinatie voorkomt), is de hoogte van de boetes niet te billijken. Zij zullen hun zorgverzekering verliezen en dat is m.i. onaanvaardbaar.

– Verhouding tussen relatieve lasten en verantwoordelijkheid voor schade

"What is the average income of their employees? I would bet unter 30,000. How many of their employees are raising a family and may be singel parents?" (MSNBC, Sharon P, August 10, 2007)

"The middel income family is strugeling now. That $750 could make a big diffrence in their monthly budget. Where upper managementit is spending money." (MSNBC, Sharon P, August 10, 2007)

Deze citaten vormen een aanwijzing dat de boetes die Clarian oplegt een substantiële aanslag vormen op het maandelijkse budget van werknemers in de lagere inkomensgroepen. Amerikaanse werkgevers zijn echter niet verplicht om de zorgverzekering van hun werknemers te betalen. Gerelateerd aan de gemiddeld hogere zorgkosten van werknemers die niet aan de gezondheidscriteria voldoen, is de hoogte van de boetes niet onredelijk. Voor werknemers die de boetes kunnen betalen, acht ik de hoogte van de boetes niet evident onbillijk.

Aspect van hardheid in specifieke gevallen

"In addition, if employees have a medical condition that prevents them from meeting certain standards, they can obtain an exemption by providing a doctor's note." (Workforce Management, Wojcik, 2007)

Een medisch probleem lijkt de enige reden te zijn op basis waarvan werknemers zich aan deelname aan het preventieplan kunnen onttrekken. Voor de situatie dat mensen de boetes niet kunnen betalen en niet in staat zijn om hun ongezonde gedrag aan te pakken, lijkt niets geregeld. M.i. is het onaanvaardbaar als mensen hierdoor hun zorgverzekering verliezen. In dat geval valt toepassing van de preventiemaatregel te hard uit.

Aspect van gelijke toegang tot gezondheidszorg en preventie

Het preventieplan heeft in principe geen invloed op de gelijke kansen van betrokken partijen wat betreft hun toegang tot gezondheidszorg en preventie. Mensen

houden gelijke toegang, tenzij zij de hogere zorgpremie (inclusief boetes voor ongezond gedrag) van hun zorgverzekering niet meer kunnen betalen. In dat geval wordt hun toegang tot gezondheidszorg en preventie, weliswaar onbedoeld, sterk verminderd en dat is m.i. onaanvaardbaar.

Aspect van verdeling naar behoefte van gezondheidszorg en preventie

Door het preventieplan van Clarian worden de beschikbare gelden voor gezondheidszorg en preventie minder verdeeld naar behoefte en meer naar verantwoordelijkheid. Hoe meer men zelf verantwoordelijk wordt geacht voor de eigen gezondheidsproblemen, hoe meer men zelf voor gezondheidszorg en preventie moet betalen. Zo lang iedereen de eigen premie ((inclusief boetes voor ongezond gedrag) van de eigen zorgverzekering kan betalen, verandert het preventieplan niets aan de verdeling van gezondheidszorg en preventie naar behoefte. Men zou kunnen stellen dat mensen die worden beboet voor ongezond gedrag, minder geld overhouden om op eigen kosten aanvullende gezondheidszorg en preventie in te kopen. Ervan uitgaande dat de basiszorgverzekering die Clarian voor haar werknemers afsluit, voorziet in de noodzakelijke zorg, acht ik deze stelling van ondergeschikt belang.

7.5.3 Timing van de implementatie

Na analyse van alle door mij onderscheiden aspecten van het criterium 'rechtvaardigheid' resteren een aantal citaten uit de door mij bestudeerde literatuur, die door mij (ondermeer) zijn gekoppeld aan het criterium 'rechtvaardigheid', maar die in de analyse van dit criterium niet aan bod zijn gekomen. Ten behoeve van de analyse geef ik hieronder een opsomming van deze citaten:

> *"We didn't just decide this. We have <u>five years</u> of wellness program <u>history</u> that included health risk appraisals, health coaching and other resources to help people reduce their risk factors," he says.* (Workforce Management, Wojcik, 2007)
>
> *Clarian's decision to impose fees came <u>after several years</u> of operating a comprehensive wellness program, according to Steven Wantz, senior vice president for administration and human resources at Clarian, which has 13,000 employees working at five hospitals in the Indianapolis metropolitan area.* (Workforce Management, Wojcik, 2007)
>
> *But back at Clarian Health, executives said their program of surcharges will be fair. Employees have <u>18 months to get ready with</u> workplace exercise and health coaching from the company.* (NBC5, 2007)

> *Clarian said it is giving employees time to make improvements before most of the extra charges take effect and is providing them with free smoking-cessation and wellness programs.* (FoxNews.com, 2007)
>
> *The program will assess $5 per-paycheck fees on employees who do not meet minimum standards for body mass index, cholesterol, blood glucose, blood pressure and nonuse of tobacco. Although it will not be fully implemented until 2009, the effort was announced this year to give employees plenty of warning, Wantz says.* (Workforce Management, Wojcik, 2007)
>
> *"This kind of plan is not as unusual as it would have been five years ago. I've heard of employers considering getting more aggressive," says Sharon Cohen, group and health care benefits counsel at Watson Wyatt Worldwide in Washington. "I think more and more employers, now that there are final rules, want to take the next step. If they're not implementing this type of arrangement, it's on the table and they're discussing it."* (Workforce Management, Wojcik, 2007).

De bovenstaande citaten hebben allemaal te maken met de timing van de implementatie van het preventieplan:
- Is de tijd rijp voor toepassing van het betreffende preventieplan, of is toepassing van het betreffende preventieplan zeer ongebruikelijk?
- Is de doelgroep van het preventieplan geleidelijk op de gevraagde gedragsverandering voorbereid, of is de gevraagde gedragsverandering helemaal nieuw voor de doelgroep?
- Krijgt de doelgroep de tijd om zich aan de nieuwe regels aan te passen of worden de nieuwe regels plotseling ingevoerd?

Op basis van de bovenstaande citaten stel ik voor een nieuw criterium 'timing van de implementatie van de preventiemaatregel' in het model op te nemen. Deze timing hangt samen met de rechtvaardigheid van de preventiemaatregel (de citaten waren door mij (ondermeer) gekoppeld aan het criterium rechtvaardigheid), maar heeft ook consequenties voor het draagvlak voor de preventiemaatregel. Daarom plaats ik het nieuwe criterium 'timing' tussen de criteria 'rechtvaardigheid' en 'draagvlak' van het model. Door de criteria 'rechtvaardigheid' en 'rechtsgelijkheid' samen te voegen tot één criterium 'rechtvaardigheid', ontstaat hiervoor ruimte binnen het model.

7.5.4 Eindoordeel (criterium rechtvaardigheid)

De rechtvaardigheid van het preventieplan van Clarian schiet op (tenminste) twee punten tekort. In de eerste plaats acht ik voor de werknemers die wel aan alle gezondheidscriteria van Clarian voldoen, de indringendheid van het preventieplan niet te rechtvaardigen. Dit betreft het gegeven dat zij $650 (maximale jaarlijkse

boete) moeten betalen, als zij willen voorkomen dat hun werkgever hun lichamelijke integriteit en privacy aantast. In de tweede plaats acht ik het niet uitgesloten dat sommige werknemers hun zorgverzekering verliezen, omdat zij de boetes niet kunnen betalen en niet in staat zijn om hun leefstijl aan te passen. De lasten van het verlies van de zorgverzekering acht ik te groot in verhouding tot het risico op schade ten gevolge van een ongezonde leefstijl, waarvoor de betreffende werknemers verantwoordelijk zijn.

7.6 Twaalfde criterium: rechtsgelijkheid

7.6.1 Operationalisering en wijze van beoordeling

Operationalisering

Het criterium 'rechtsgelijkheid' betreft de vergelijkende rechtvaardigheid; de vraag of in vergelijkbare situaties vergelijkbaar wordt gehandeld. De vergelijkende rechtvaardigheid kan betrekking hebben op heel uiteenlopende gedragingen van mensen met risico op leefstijlgerelateerde aandoeningen: teveel of te weinig eten, teveel of te weinig bewegen, teveel of te weinig rust nemen, roken, overmatig alcohol- of drugsgebruik, enz. In dit verband geldt de volgende waarschuwing (Pearson, 2009, 849):

> *"A separate ethical concern for penalty programs lies in the risk that types of behavior will be selected as targets because of discrimination. The evidence linking behavior with poor health and increased costs must be evaluated in an even-handed way; penalty programs should not be based on moral judgments made by employers in a discriminatory and stigmatizing manner. The risk is that penalty programs will find it easiest to focus on behavior and outcomes that are generally viewed as "socially unacceptable within a workplace community or society at large. … Not all choices of illness are counted alike. Those that are targeted tend to be sins – sloth, gluttony, lust, to use their old-fashioned names- or to be behavior such as drug addiction, of the marginalized. Were other potentially costly types of behavior considered in the selection process? Is it for evidence, practically, or other reasons that penalties were not focused on excessive alcohol intake, failure to get cancer screening, engagement in risky outdoor sports, or other potentially unhealthy types of behavior that might have imposed unfair financial burdens on the workforce. … Thus, a central component of penalty programs must be that decisions regarding which types of behavior receive penalties are made with the participation of employees and use publicly accessible, evidence-based reasons as justification."*

Bij de beoordeling van het preventieplan van Clarian aan de hand van het criterium 'rechtvaardigheid' heb ik gesteld (zie §7.5.1 onder *"3. Aspect van draagkracht"*):

> *"Men kan ook stellen dat het onrechtvaardig is, als er juist bij de zwakste partijen en niet bij de sterkste partijen een stapeling van lasten optreedt. Dit aspect van (on) rechtvaardigheid behandel ik bij het volgende criterium van het model: rechtsgelijkheid."*

De vermeende onrechtvaardigheid van een stapeling van lasten bij de zwakste partijen betreft eigenlijk zowel de verdelende rechtvaardigheid (criterium 'rechtvaardigheid') als de vergelijkende rechtvaardigheid (criterium 'rechtsgelijkheid'). Een strikt onderscheid tussen verdelende en vergelijkende rechtvaardigheid is wellicht behulpzaam bij de theorievorming, maar sluit niet aan op de praktijk, waarin de verdelende en vergelijkende rechtvaardigheid door elkaar heen lopen. Naar aanleiding van de introductie van het criterium 'timing van de implementatie van de preventiemaatregel' (zie §7.5.3) heb ik besloten de criteria 'rechtvaardigheid' en 'rechtsgelijkheid' – na afronding van de toetsing van het model aan de hand van de casus Clarian – samen te voegen tot één criterium 'rechtvaardigheid'. Hierdoor verdwijnt binnen het model het strikte onderscheid tussen verdelende en vergelijkende rechtvaardigheid en sluit het model beter aan op de complexiteit van de praktijk.

Voor de beoordeling van de gerechtvaardigdheid van het preventieplan van Clarian aan de hand van het criterium 'rechtsgelijkheid' gebruik ik de volgende vragen:

- Draagt de preventiemaatregel eraan bij dat mensen met vergelijkbare ongezonde of risicovolle leefstijlen (leefstijlen die dezelfde risico's opleveren) hiervoor gelijkelijk worden belast?
- Draagt de preventiemaatregel eraan bij dat de sterkste schouders de zwaarste lasten dragen?

Beide vragen kunnen betrekking hebben op de doelgroep van de preventiemaatregel, bepaalde deelpopulaties van de samenleving (voorbeelden van deelpopulaties zijn: alle werknemers van een bepaalde werkgever, alle mensen in loondienst, alle vrouwen) en op de samenleving als geheel.

Wijze van beoordeling

Voor de beoordeling van de rechtsgelijkheid (vergelijkende rechtvaardigheid) geldt evenals voor de beoordeling van de rechtvaardigheid (verdelende rechtvaardigheid) dat afhankelijk van het gewicht dat men aan verschillende concepten van

rechtvaardigheid toekent (zie de bespreking van cluster 6 in §3.3) de beoordeling tot andere uitkomsten leidt. De wijze van beoordeling is bij het criterium 'rechtsgelijkheid' eveneens hetzelfde als bij het criterium 'rechtvaardigheid'. Bij het criterium 'rechtsgelijkheid' wordt beoordeeld of toepassing van de preventiemaatregel vanuit het perspectief van de rechtsgelijkheid niet evident onredelijk of onbillijk is.

7.6.2 Beoordeling van casus Clarian Health aan de hand van criterium 'rechtsgelijkheid'

Worden mensen met vergelijkbare leefstijlen gelijkelijk belast?

In de door mij bestudeerde reacties op het preventieplan van Clarian worden diverse voorbeelden aangevoerd van leefstijlen die eveneens tot extra zorgkosten kunnen leiden, maar die door Clarian niet worden beboet, zoals: alcoholgebruik of alcoholverslaving, niet gebruiken van autogordels, minder dan tweemaal per dag tanden poetsen, hebben van ondergewicht of anorexia, drugsverslaving, en krijgen van veel kinderen in een korte periode. In hoeverre is het redelijk en billijk dat deze gedragingen, die kunnen leiden tot hogere zorgkosten, niet door Clarian worden beboet? Ik zal deze gedragingen achtereenvolgens bespreken en vervolgens trachten tot een oordeel te komen. Uiteindelijk is het doel hiervan niet de beoordeling van de casus Clarian, maar de toetsing van de juistheid en praktische toepasbaarheid van het model. Daarom zal ik deze gedragingen bespreken op basis van mijn algemene kennis hierover en naar deze gedragingen geen nader literatuuronderzoek doen.

Alcoholgebruik of alcoholverslaving

Beperkt alcoholgebruik leidt waarschijnlijk niet tot hogere zorgkosten en kan zelfs een preventieve werking hebben m.b.t. het krijgen van hart- en vaatziekten. Alcoholverslaving leidt waarschijnlijk wel tot hogere zorgkosten. Het onderscheid tussen alcoholgebruik en alcoholverslaving is door middel van periodieke toetsing op alcoholgebruik m.i. moeilijk objectief aan te tonen. Alcoholgebruik op het werk wordt door de meeste werkgevers sowieso verboden. Als mensen onder de maat functioneren, kan dat tijdens functioneringsgesprekken aan de orde worden gesteld en kunnen daar consequenties aan worden verbonden. Het periodiek toetsen van werknemers op alcoholverslaving lijkt mij praktisch niet goed uitvoerbaar, omdat alcoholgebruik buiten werktijd wel is toegestaan.

Niet gebruiken van autogordels

Het niet gebruiken van autogordels is inderdaad risicovol gedrag. Ook hiervoor geldt dat het praktisch niet goed uitvoerbaar is om te toetsen of mensen hun autogordel altijd omdoen.

Minder dan tweemaal per dag tanden poetsen

Het minder dan tweemaal per dag tanden poetsen zou inderdaad tot hogere tandartskosten kunnen leiden. Het is echter de vraag of deze kosten wel tot de collectieve zorgverzekering behoren of dat mensen hiervoor individueel een verzekering afsluiten of deze kosten zelf betalen. Als tandartskosten tot de collectieve zorgverzekering behoren, zou de werkgever van werknemers kunnen vragen om hun gebit goed te verzorgen, bijvoorbeeld door hen tenminste tweemaal per dag te laten poetsen. Clarian zou dit kunnen controleren door werknemers te verplichten op het werk tweemaal per dag hun tanden te poetsen, of door regelmatig te controleren of het gebit van werknemers op een nader overeen te komen moment van de dag goed gereinigd is (bijvoorbeeld met behulp van een tandplakverklikker). Een dergelijke maatregel is echter zeer betuttelend en daardoor zeer indringend en lijkt mij daarom onaanvaardbaar. Op andere wijze is het toetsen of mensen dagelijks tenminste tweemaal hun tanden poetsen praktisch niet goed uitvoerbaar.

Hebben van ondergewicht of anorexia

Alleen ernstig overgewicht wordt door Clarian beboet. Analoog daaraan zou het redelijk zijn om alleen ernstig ondergewicht te beboeten. In de Amerikaanse samenleving hebben veel minder volwassenen ernstig ondergewicht (anorexia) dan ernstig overgewicht (obesitas)[15]. Wellicht wordt anorexia daarom minder als een maatschappelijk probleem ervaren dan obesitas[16]. Bovendien wordt anorexia door artsen erkend als psychiatrische aandoening en ongezondheid ten gevolge van ziekte wordt niet door Clarian beboet. Obesitas wordt door artsen niet als psychiatrische aandoening erkend. Obesitas kan (mede) het gevolg zijn van een (psychiatrische) aandoening, maar ook andere oorzaken hebben (gebrek aan wilskracht, obesogene omgeving, enz.). Op basis van deze overwegingen acht ik het niet evident onredelijk en onbillijk dat het hebben van ondergewicht buiten het kader van het preventieplan van Clarian valt.

Drugsverslaving

Beperkt drugsgebruik leidt waarschijnlijk niet tot hogere zorgkosten. Drugsverslaving leidt waarschijnlijk wel tot hogere zorgkosten. Het onderscheid tussen beperkt drugsgebruik en drugsverslaving is door middel van periodieke toetsing

15. De hoogste geschatte prevalentie van anorexia binnen de bevolking die ik in PubMed heb gevonden bedraagt 2.2% van de (Finse) bevolking (gemeten over het hele leven) (Keski-Rahkonen, 2007). Deze studie stelt dat veel anorexia binnen de gezondheidszorg niet wordt ondekt en dat andere studies daarom met veel te lage schattingen komen. Onder de volwassen bevolking van de US is de prevalentie van obesitas ruim 35% (Flegal, 2012).
16. De zoekterm { (anorexia OR anorexic) AND "united states" } levert in PubMed 2.542 hits op. De zoekterm { (obesity OR obese) AND "united states" } levert in PubMed 33.700 hits op.

op drugsgebruik voor zover ik weet moeilijk objectief aan te tonen. Omdat drugsgebruik in Amerika wettelijk verboden is, zou Clarian om die reden werknemers op 'drugsgebruik tijdens en buiten werktijd' kunnen toetsen. Een dergelijke toetsing past echter niet binnen het preventieplan van Clarian, omdat de relatie met het risico op gezondheidsschade en hogere zorgkosten, die Clarian met het preventieplan wil bestrijden, onduidelijk is. Daarom acht ik het niet evident onredelijk en onbillijk dat drugsverslaving buiten het preventieplan van Clarian valt.

Krijgen van veel kinderen in een korte periode
Bemoeienis van de werkgever met de gezinsvorming acht ik zeer indringend. Bovendien is zwangerschap geen ziekte. Om deze redenen acht ik het onredelijk en onbillijk als de werkgever in het kader van een preventieplan ter bevordering van de gezondheid werknemers zou beboeten voor het krijgen van 'veel kinderen' in een korte periode.

Dragen de sterkste schouders de zwaarste lasten?

> *"yeah, that's it... take money away from the lower income workers in the hospitals who most likely have a lot of these issues... that'll ensure they get the help they need. i know for a fact that a lot of these administrative folks aren't exactly models of perfection with regard to health."* (MSNBC, C. Boyle, August 10, 2007)
>
> *"It is proven that familys of lower income eat fatter foods to strech their budget."* (MSNBC, Sharon P, August 10, 2007)

De beoordeling van de casus Clarian aan de hand van het criterium 'rechtsgelijkheid van het model, baseer ik op de volgende veronderstelling: de verschillen in gezondheid en leefstijl tussen mensen met een lage en een hoge SES, zoals die voorkomen in Nederland (zie bijlage 9), zijn op hoofdlijnen ook van toepassing op de werknemers van Clarian. Het baseren van de beoordeling op deze veronderstelling acht ik aanvaardbaar, omdat het uiteindelijke doel van deze beoordeling niet is de beoordeling van de casus Clarian, maar de toetsing van de juistheid en praktische toepasbaarheid van het model aan de hand van de casus Clarian.

De werknemers van Clarian met een lage SES hebben gemiddeld genomen een minder goede gezondheid dan die met een hoge SES. Daardoor komen ten gevolge van het preventieplan van Clarian de zwaarste lasten waarschijnlijk terecht bij de werknemers met de financieel zwakste schouders (zij moeten naar verwachting het meest boete betalen). M.i. wordt het ongezonde gedrag van werknemers met een lage en met een hoge SES op dezelfde wijze bestraft. Zo bezien is er dus geen sprake van rechtsongelijkheid tussen werknemers met een lage en een hoge SES.

Desalniettemin leidt het preventieplan van Clarian gemiddeld genomen wel tot een toename van de inkomensverschillen tussen de werknemers met een lage en hoge SES. De reden is dat de werknemers met een lage SES gemiddeld genomen ongezonder zijn. M.i. is deze toename van inkomensverschillen niet evident onredelijk of onbillijk als deze wordt afgezet tegen het belang van Clarian om de sterk stijgende zorgverzekeringskosten te bestrijden. Deze toename van inkomensverschillen acht ik wel onbillijk, als werknemers (met een lage SES) door het preventieplan van Clarian hun zorgpremie niet meer kunnen betalen of anderszins serieus in de financiële problemen raken (dit heb ik reeds besproken onder het voorgaande criterium distributieve rechtvaardigheid).

7.6.3 Eindoordeel (criterium rechtsgelijkheid)

De keuze van de leefstijlen waartoe het preventieplan van Clarian zich beperkt, acht ik niet onredelijk en niet onbillijk. Ook de mate waarin het preventieplan van Clarian de (financieel) zwaarste lasten bij de (financieel) zwakste schouders (mensen met een lage SES) legt, acht ik niet onredelijk en niet onbillijk. Dat niet is uitgesloten dat mensen door het preventieplan van Clarian ernstig in de financiële problemen raken of hun zorgpremie niet meer kunnen betalen, acht ik wel onredelijk en onbillijk (zie het eindoordeel over het criterium 'rechtvaardigheid').

7.7 Eindoordeel over de 2e zeef: effecten en neveneffecten

Het preventieplan van Clarian kan, op basis van de door mij bestudeerde teksten, de 2e zeef 'effecten en neveneffecten' van het model niet passeren. In de eerste plaats acht ik de indringendheid van de preventiemaatregel (structurele aantasting van de lichamelijke integriteit en privacy) niet te rechtvaardigen voor de werknemers die wel aan de gezondheidscriteria van Clarian voldoen. Zie (het eindoordeel over) de criteria 'indringendheid' en 'rechtvaardigheid'. In de tweede plaats acht ik het niet te rechtvaardigen dat er zo weinig informatie beschikbaar is over de aantasting van de lichamelijke integriteit en van de privacy die de preventiemaatregel met zich kan brengen. Zie (het eindoordeel over) het criterium 'indringendheid'. Ten slotte acht ik het niet te rechtvaardigen dat werknemers van Clarian (en hun gezin) door het preventieplan ernstig in de financiële problemen kunnen raken of hun zorgpremie niet meer kunnen betalen. In die specifieke gevallen valt de preventiemaatregel te hard uit; misschien zou een hardheidsclausule uitkomst kunnen bieden. Zie (het eindoordeel over) de criteria 'rechtvaardigheid' en 'rechtsgelijkheid'.

Naar aanleiding van de toetsing van de casus Clarian aan de hand van de criteria van de 2e zeef van het model heb ik een ***criterium 'timing van de implementatie'*** aan het model toegevoegd. Om de clusterstructuur van het model te herstellen (7 clusters van 2 criteria) heb ik de criteria 'rechtvaardigheid' en 'rechtsgelijkheid' tot één criterium samengevoegd. Een strikt onderscheid tussen verdelende rechtvaardigheid (criterium 'rechtvaardigheid') en vergelijkende rechtvaardigheid (criterium 'rechtsgelijkheid') is wellicht behulpzaam bij de theorievorming, maar sluit niet aan op de complexiteit van de praktijk. Door binnen het model de criteria 'rechtvaardigheid' en 'rechtsgelijkheid' samen te voegen tot één criterium 'rechtvaardigheid' sluit het model beter aan op de complexiteit van de praktijk.

8 Toetsing 3e zeef van het beoordelingsmodel aan casus 'Clarian Health' (versie 6 van het model)

In dit hoofdstuk beoordeel ik de gerechtvaardigdheid van het preventieplan van Clarian Health aan de hand van de criteria van de 3e zeef van het model (versie 6 van het model). De 3e zeef 'implementatie' is samengesteld uit de criteria:
- draagvlak;
- uitvoerbaarheid.

8.1 Dertiende criterium: draagvlak

8.1.1 Operationalisering en wijze van beoordeling

Operationalisering
Het criterium 'draagvlak' betreft de volgende vragen:
- In hoeverre en op welke manier(en) is er aandacht besteed aan het verwerven van draagvlak voor de preventiemaatregel?
- Hoeveel draagvlak is er voor de preventiemaatregel?

Wijze van beoordeling
Het gaat hierbij om draagvlak bij alle betrokken partijen en in ieder geval bij de:
- doelgroep waarop de preventiemaatregel zich richt;
- belanghebbenden die door de preventiemaatregel worden beschermd;
- partijen die de preventiemaatregel uitvoeren en/of handhaven.

Indien relevant gaat het ook om maatschappelijk draagvlak. Afhankelijk van de inhoud van de preventiemaatregel zijn verschillende partijen bepalend voor het maatschappelijk draagvlak. Bij partijen kan bijvoorbeeld worden gedacht aan:
- algemene bevolking;
- pressiegroepen;
- werknemers;
- werkgevers;
- bedrijfsleven;
- aandeelhouders;
- overheid;

- politiek;
- wetenschap;
- media;
- mensen met een ongezonde leefstijl onder de bovengenoemde partijen.

Beoordeeld wordt of er voldoende draagvlak bestaat bij de betrokken partijen voor toepassing van de preventiemaatregel, zodat de handhaving en uitvoering niet onnodig moeilijk en/of onevenredig kostbaar worden. Vanuit dit perspectief wordt tevens beoordeeld of er voldoende aandacht is en wordt besteed aan het verwerven van draagvlak voor de preventiemaatregel.

8.1.2 Beoordeling van casus Clarian Health aan de hand van criterium 'draagvlak'

In hoeverre en hoe is draagvlak voor de preventiemaatregel verworven?
Uit de door mij bestudeerde teksten ontstaat de indruk dat door Clarian niet of nauwelijks aandacht is besteed aan het verwerven van draagvlak voor de preventiemaatregel. Clarian heeft de preventiemaatregel aangekondigd en is vervolgens overvallen door een storm van negatieve reacties onder het eigen personeel en in de media:

> *"Clarian Health admits that its program is aggressive by design, and that employee reaction has been mixed, with much debate on its internal message boards." (McGregor, 2007)*

> *"Clarian Health, an Indianapolis-based hospital system, drew media attention this summer when it announced that by 2009, employees whose body-mass index (a fatness indicator measured by a person's weight relative to height) was too high could expect deductions from their paychecks. The company recently backpedaled and said that weight loss would be voluntary and that a worker's pay would not be influenced by his failure to drop pounds."* (Baker, 2007)

Hoeveel draagvlak is er voor de preventiemaatregel?

Draagvlak bij de doelgroep waarop de preventiemaatregel zich richt
De doelgroep waarop de preventiemaatregel zich richt, zijn de werknemers van Clarian met een ongezonde leefstijl. Onder de doelgroep van het preventieplan zijn niet of nauwelijks voorstanders, maar wel veel tegenstanders van het preventieplan te vinden. In juni 2007 kondigt Clarian haar preventieplan aan. Al in het najaar van 2007 haalt Clarian bakzeil, vanwege de protesten onder haar werkne-

mers. In oktober 2007 schrijft Andrews hierover op de website van US News & World Report (Andrews, 2007):

> *"Healthy penalty. But incentive-based programs can be lightning rods [bliksemafleiders]. Clarian Health Partners, a large Indianapolis-based healthcare system, earlier this year found itself on the defensive after announcing that starting in 2009, 13,000 employees at five area hospitals would face a surcharge of up to $30 per pay period if they smoked, didn't fill out a health-risk questionnaire, or fell short of targets for weight, blood sugar, cholesterol, and blood pressure—a $5 penalty for each."*
>
> *"But employees rebelled, even though the company offered free lifestyle and nutrition coaching, stop-smoking programs, fitness centers, and other wellness activities. "We couldn't get people to hear the message because they were so stuck on the charges," says Ladd."*
>
> *"Clarian reversed course. Rather than being penalized for falling short of health targets, employees who meet them will receive up to $30 extra in every paycheck. "Now the package is more tolerable for employees to hear and digest," says Ladd."*

Draagvlak bij belanghebbenden die door de preventiemaatregel worden beschermd
De belanghebbende van het preventieplan is vooral Clarian zelf (bestrijding van hoge zorgverzekeringspremies, ziekteverzuim en arbeidsongeschiktheid). Indirect kunnen werknemers van Clarian met een gezonde leefstijl baat hebben bij het preventieplan. Geld dat de werkgever uitgeeft aan zorgverzekeringspremies kan de werkgever immers niet meer aan andere arbeidsvoorwaarden besteden. Bovendien kunnen werknemers met een gezonde leefstijl hinder ondervinden van meer ziekteverzuim en arbeidsongeschiktheid bij collega's met een ongezonde leefstijl. Een nadeel voor werknemers met een gezonde leefstijl is echter de aantasting van de privacy. Bovendien worden de collegiale verhoudingen onder druk gezet, als zij het preventieplan van Clarian openlijk steunen. Onder haar eigen werknemers is de (openlijke) steun die Clarian voor het preventieplan ontvangt klein en het verzet tegen het preventieplan groot. Ter illustratie een citaat:

> *"While some employees were supportive, there's been "the other reaction that this is very personal," says Wantz*[1]*, with people asking, "'How dare you? This is my personal space.' There's been a lot of questions and confusion."* (McGregor, 2007)

1. Wantz is een woordvoerder van Clarian.

Draagvlak bij partijen die de preventiemaatregel uitvoeren en/of handhaven
Clarian is de partij die de preventiemaatregel moet uitvoeren en handhaven. Clarian is tevens de partij die belang heeft bij de preventiemaatregel en het initiatief tot toepassing van de preventiemaatregel heeft genomen. Op dit punt lijkt het draagvlak voor de uitvoering en handhaving van de preventiemaatregel dus voldoende. Echter, werknemers van Clarian zullen een rol moeten spelen bij de uitvoering en handhaving van het preventieplan en dat zou problemen kunnen oproepen. Omdat Clarian niet tot uitvoering van het preventieplan in zijn oorspronkelijke vorm (met negatieve financiële incentives) is overgegaan, is hierover geen nadere informatie beschikbaar.

Maatschappelijk draagvlak
Uit de door mij bestudeerde teksten komen de volgende partijen naar voren als bepalend voor het maatschappelijk draagvlak voor het preventieplan van Clarian:
- algemene bevolking;
- werknemers;
- werkgevers;
- media;
- overheid;
- mensen met een ongezonde leefstijl onder de bovengenoemde partijen;
- pressiegroep van mensen met overgewicht (Big Fat Blog, 2007).

Waarschijnlijk is deze opsomming niet compleet. Het is bijvoorbeeld denkbaar dat artsen pas tegen het preventieplan gaan protesteren, als werknemers van Clarian hen gaan vragen om 'ongezondheidsverklaringen' af te geven. Zo ver is het echter nooit gekomen, omdat Clarian de negatieve financiële incentives van het preventieplan heeft ingetrokken.

De positieve reacties op het preventieplan van Clarian komen vooral van werkgevers, die benieuwd zijn of Clarian met haar preventieplan succes zal boeken. Slechts een enkele werkgever is Clarian voorgegaan met het toepassen van negatieve financiële incentives ter bestrijding van ongezond gedrag van werknemers. Een deel van de werkgevers staat negatief tegenover het preventieplan van Clarian. De werkgevers die positief staan tegenover het preventieplan van Clarian nemen een afwachtende houding aan, omdat zij beducht zijn voor protesten van werknemers en negatieve neveneffecten, zoals een verslechtering van de werksfeer en problemen bij het werven van nieuw personeel. Ter illustratie volgen enkele passages uit het artikel van McGregor uit augustus 2007:

> *"But some employment lawyers and wellness program administrators believe Clarian's approach may not be so unusual in coming years. … Garry Mathiason, a senior*

partner at employment law firm Littler Mendelson, says more than 300 companies have requested its assistance on mandatory wellness initiatives since it released a study on the topic in April. …

While Clarian's plan is highly unusual, a few other employers are using the stick approach, too. Western & Southern Financial Group, a Cincinnati-based insurance and financial-services provider, has been issuing employees with high BMIs a surcharge to their premiums since 2001. … Preventure, a provider of fitness and wellness programs for midsize to large employers, has one client in the manufacturing industry that rewards employees when their BMI falls from their baseline score and charges them more when their number rises. "We haven't seen a lot of companies do it," says Laura Gilligan, director of business development for Preventure, "but I sense it might be a trend." …

"The concept of penalizing for poor health is not well accepted, and a lot of employees would react badly to it," says Bruce Kelley, a consultant in the health-care practice of human resources advisory firm Watson Wyatt Worldwide. …

That's why some benefits consultants and health-care groups think many companies won't adopt a plan like Clarian's anytime soon. "People are not interested in making their employees unhappy," says Helen Darling, president of the National Business Group on Health, a nonprofit that represents large employers' health-care interests. "We're in a war for talent, and there are more than enough work-related challenges to manage people and performance that [penalizing] for something that is so complicated, so personal, so hard to deal with, just isn't the right thing to do at this point."

Still, Clarian's approach has already attracted interest. Wantz[2] says it has received calls from several local companies, along with other hospital systems, interested in their approach. "The most common response from other employers," he says, "has been 'thank you.'" "

In januari 2009 schrijft Gabel dat werknemers en werkgevers negatieve financiële incentives ter bestrijding van overgewicht onder werknemers afwijzen:

"Views on financial incentives. Both employers and employees favor positive financial incentives for participating in weight management programs and oppose financial penalties. … Employees strongly support discounts on health insurance or other monetary incentives and vehemently oppose financial penalties for participating or not participating in various workplace programs to address obesity and promote wellness." (p.52)

2. Wantz is een woordvoerder van Clarian.

Ook vakbonden kunnen voor werkgevers een belemmering vormen om ongezond gedrag van hun werknemers met negatieve financiële incentives te bestrijden (Pearson, 2009):

> *"Early reports and anecdotal information suggest that Financial incentives can effectively motivate employees to change healthy behavior, but employers are facing a backlash from organized unions and others who contend that workplace incentive programs, particularly penalty programs, are unethical."* (p.846)

Sommige mensen zijn echt boos over het preventieplan van Clarian:

> *"Boycott any company that forces this on their employees."* (Big Fat Blog, wriggle99, August 9, 2007)

> *"I will make sure not to purchase from Dell IBM Kelloggs and any of the other companies who do this to their employees."* (MSNBC, S Mck, August 11, 2007)

In 2009 schrijven Paerson e.a. (in een 'online data supplement' bij hun artikel):

> *"Due to fierce criticism from both employees and the media, Clarian ultimately retracted its proposal."*

8.1.3 Eindoordeel (criterium draagvlak)

Onder druk van protest van haar eigen personeel en onder druk van kritiek in de media heeft Clarian de negatieve financiële incentives uit haar preventieplan verwijderd. Op basis hiervan kan worden geconcludeerd dat er onvoldoende draagvlak bestond voor uitvoering van het preventieplan. Clarian had kunnen en moeten weten dat haar preventieplan controversieel was en tot heftige reacties van het eigen personeel en tot heftige reacties in de media zou kunnen leiden. Clarian lijkt voorafgaand aan de aankondiging van haar preventieplan weinig tot geen aandacht te hebben besteed aan het verwerven van draagvlak voor haar preventieplan. Op dit punt is Clarian tekortgeschoten. Ook in het van tevoren uitwerken en/of geven van voorlichting over haar preventieplan is Clarian tekortgeschoten. Uit de door mij bestudeerde documenten worden de gevolgen van het preventieplan voor de lichamelijke integriteit en privacy niet duidelijk (zie onder het criterium 'indringendheid'). Een betere uitwerking van het preventieplan en/of betere voorlichting over het preventieplan had in ieder geval een deel van de verwarring kunnen voorkomen:

> "*There's been a lot of questions and confusion."* (McGregor, 2007)

8.2 Veertiende criterium: uitvoerbaarheid

8.2.1 Operationalisering en wijze van beoordeling

Bij het operationaliseren van de voorafgaande criteria van het model heb ik goed gebruik kunnen maken van de door mij verzamelde teksten over het preventieplan van Clarian. Omdat Clarian haar preventieplan niet heeft geïmplementeerd, is er in de door mij verzamelde teksten ook maar weinig informatie over de uitvoerbaarheid van het preventieplan te vinden.

Operationalisering
Het criterium betreft de haalbaarheid en houdbaarheid (in de tijd) van de uitvoering van de preventiemaatregel.

Wijze van beoordeling
Beoordeeld wordt of redelijkerwijs kan worden aangenomen dat de uitvoering van de preventiemaatregel niet onhaalbaar en niet onhoudbaar is.

8.2.2 Beoordeling van casus Clarian Health aan de hand van criterium 'uitvoerbaarheid'

De uitvoerbaarheid van het preventieplan van Clarian is heel moeilijk te beoordelen, omdat Clarian nooit met de uitvoering is begonnen. Volgens de logica van het model hoeft de uitvoerbaarheid ook niet meer te worden beoordeeld, omdat het preventieplan van Clarian de 2e zeef 'effecten en neveneffecten' van het model niet heeft kunnen passeren. In het kader van mijn onderzoek is de doelstelling van de beoordeling van het preventieplan van Clarian aan de hand van het model echter niet de beoordeling van de gerechtvaardigdheid van het preventieplan van Clarian, maar de toetsing van de 'juistheid, volledigheid en praktische toepasbaarheid' van het model. Daarom zal ik toch enkele zaken over de uitvoerbaarheid van het preventieplan van Clarian opmerken.

De haalbaarheid en houdbaarheid van de uitvoering van het preventieplan van Clarian lijken meer te worden bepaald door het (gebrek aan) draagvlak voor het preventieplan dan door de voor de uitvoering benodigde mensen en (financiële) middelen. Wat betreft de voor de uitvoering benodigde mensen en middelen lijkt het preventieplan van Clarian haalbaar en houdbaar onder de voorwaarde dat er onder de werknemers van Clarian voldoende draagvlak voor de uitvoering van de preventiemaatregel bestaat. Dat een ander bedrijf zijn werknemers al meer dan 5

jaar een toeslag op de zorgpremie laat betalen, als hun BMI te hoog is, geeft een indicatie dat zoiets uitvoerbaar is:

> *Western & Southern Financial Group, a Cincinnati-based insurance and financial-services provider, has been issuing employees with high BMIs a surcharge to their premiums since 2001.* (Big Fat Blog, Paul, August 8, 2007)

8.2.3 Eindoordeel (criterium uitvoerbaarheid)

De haalbaarheid en houdbaarheid van de uitvoering van het preventieplan van Clarian lijken meer te worden bepaald door het (gebrek aan) draagvlak voor het preventieplan, dan door de voor de uitvoering benodigde mensen en (financiële) middelen. Wat betreft de voor de uitvoering benodigde mensen en middelen lijkt het preventieplan van Clarian haalbaar en houdbaar onder de voorwaarde dat er onder de werknemers van Clarian voldoende draagvlak voor de uitvoering van de preventiemaatregel bestaat.

8.3 Eindoordeel over de 3e zeef: implementatie

Het preventieplan van Clarian kan de 3e zeef 'implementatie' van het model niet passeren. De reden is dat Clarian onvoldoende aandacht heeft besteed aan het verwerven van draagvlak voor het preventieplan en dat er te weinig draagvlak voor implementatie van het preventieplan is.

9 Conclusies van toetsing aan casus 'Clarian Health' (versie 7 van het model)

In de hoofdstukken 6, 7 en 8 heb ik de gerechtvaardigdheid van het preventieplan van Clarian Health beoordeeld aan de hand van versie 6 van het beoordelingsmodel. Het doel hiervan is het toetsen van de juistheid, volledigheid en praktische toepasbaarheid van het model. In dit hoofdstuk geef ik eerst een eindoordeel over de gerechtvaardigdheid van het preventieplan van Clarian Health (§9.1). Vervolgens geef ik op basis van de toetsing van het model aan de hand van de casus Clarian Health een oordeel over de juistheid, volledigheid en praktische toepasbaarheid van het model (§9.2).

9.1 Eindoordeel over de gerechtvaardigdheid van het preventieplan van Clarian Health

Na afronding van de beoordeling van het preventieplan van Clarian aan de hand van de 2e zeef van het model, drong zich de conclusie op dat de zes door mij gebruikte teksten over het preventieplan van Clarian (zie hoofdstuk 5) onvoldoende informatie boden om tot een volledige beoordeling te komen. Van tevoren had ik dit niet aanzien komen. Voordat ik aan de beoordeling van het preventieplan van Clarian begon, verwachtte ik dat de zes door mij gebruikte teksten ruim voldoende informatie zouden bieden om de casus te beoordelen. De eerste zin van de inleiding bij de casus Clarian (hoofdstuk 5) luidt: *"The proof of the pudding is in the eating"*. Dat blijkt inderdaad het geval te zijn. Ik heb toen overwogen om bij Clarian extra informatie over het preventieplan op te vragen. Om de volgende redenen heb ik hiervan af gezien:

- Nieuwe informatie zou leiden tot uitbreiding van het 'bronbestand' en dan zou ik eigenlijk de beoordeling vanaf het eerste criterium opnieuw moeten doen.
- Het lag niet in de verwachting dat nieuwe informatie bij zou dragen aan de doelstelling van de beoordeling van het preventieplan van Clarian, die bestond uit de toetsing van de juistheid, volledigheid en praktische toepasbaarheid van het model.
- De tijd die ik zou besteden aan het perfectioneren van de casus Clarian, zou ik beter kunnen besteden aan de toetsing van het model aan een nieuwe casus,

waarin ik de lering die ik kon trekken uit de beoordeling van de casus Clarian zou verdisconteren.
- Juist door het ontbreken van volledige informatie over het preventieplan van Clarian, werd de rol van de 'beoordelingseis van transparantie' heel helder en die zou weer minder duidelijk worden als ontbrekende informatie zou worden aangevuld.

De gerechtvaardigdheid van het preventieplan van Clarian is dus door mij beoordeeld op basis van de informatie die via internet over het preventieplan beschikbaar was. Op basis van deze informatie zijn het ontwerp (1e zeef van het model), de effecten en neveneffecten (2e zeef) en de implementatie (3e zeef) van dit preventieplan aan de hand van het model niet te rechtvaardigen. In de eerste plaats, omdat er over het preventieplan, m.b.t. veel criteria van het model, onvoldoende informatie beschikbaar is om tot een betrouwbare beoordeling te kunnen komen. In de tweede plaats, omdat op basis van de informatie die wel beschikbaar is, geconcludeerd kan worden dat toepassing van het preventieplan van Clarian in de huidige vorm niet gerechtvaardigd is. De belangrijkste inhoudelijke punten van kritiek, die rechtvaardiging van het preventieplan van Clarian in de weg staan, zijn de volgende:
- De keuze van de Amerikaanse federale overheid om werkgevers in de gelegenheid te stellen om, weliswaar binnen een gegeven (financieel) kader[1], <u>naar eigen inzicht</u> de eigen werknemers financieel te straffen voor een ongezonde leefstijl binnen en <u>buiten werktijd</u>, acht ik zeer discutabel. Werkgevers zijn in mijn ogen ongeschikt om dergelijke preventiemaatregelen te ontwerpen en uit te voeren. Ter voorkoming van rechtsongelijkheid tussen werknemers van verschillende werkgevers en willekeur van werkgevers zou het beter zijn als de democratisch gekozen rijksoverheid zou beslissen over de vormgeving van dergelijke preventiemaatregelen. De uitvoering van dergelijke preventiemaatregelen kan beter worden overgelaten aan een 'trusted third party' (bijvoorbeeld de zorgverzekeraar). (Zie §6.7)
- De indringendheid van de preventiemaatregel (structurele aantasting van de lichamelijke integriteit en privacy) acht ik niet te rechtvaardigen voor de werknemers die wel aan de gezondheidscriteria van Clarian voldoen. (Zie §7.7)
- Dat werknemers van Clarian (en hun gezin) door het preventieplan ernstig in de financiële problemen kunnen raken of hun zorgpremie niet meer kunnen betalen acht ik niet te rechtvaardigen. Wellicht kan een hardheidsclausule op dit punt uitkomst bieden. (Zie §7.7)

1. Zie bijlage 8: Toelichting op §f van de HIPAA.

9.2 Oordeel over de juistheid, volledigheid en praktische toepasbaarheid van het model

De toetsing van de casus Clarian aan het model is zeer behulpzaam geweest voor het operationaliseren van de criteria en wijze van beoordeling van de criteria van het model. De beoordeling van het preventieplan van Clarian aan de hand van het criterium 'gerichtheid' heeft mij op het idee gebracht om het ***falsificatiebeginsel*** te introduceren als een van de uitgangspunten voor de beoordeling van preventiemaatregelen aan de hand van het model. Aan het schrijven van hoofdstuk 4 en 6 van het model heb ik parallel gewerkt. Pas tijdens het schrijven van hoofdstuk 6 heb ik het falsificatiebeginsel, als uitgangspunt voor de beoordeling, aan hoofdstuk 4 toegevoegd.

Naar aanleiding van de beoordeling van de casus Clarian aan de hand van het criterium 'rechtvaardigheid' van het model, heb ik een criterium ***'timing van de implementatie'*** aan het model toegevoegd (zie §7.5.3). Om de clusterstructuur van het model te herstellen (7 clusters van 2 criteria) heb ik de criteria 'rechtvaardigheid' en 'rechtsgelijkheid' tot één criterium samengevoegd. Door binnen het model de criteria 'rechtvaardigheid' en 'rechtsgelijkheid' samen te voegen tot één criterium 'rechtvaardigheid', sluit het model beter aan bij de praktijk (zie §7.7).

Nu ik bij het schrijven van deze paragraaf (alle criteria van) het model nog eens overzie, valt mij op dat het nieuwe criterium 'timing van de implementatie' niet thuishoort in de 2e zeef 'effecten en neveneffecten', maar in de 3e zeef 'implementatie' van het model. Omdat een (on)juiste timing medebepalend is voor het draagvlak voor de preventiemaatregel, plaats ik het criterium 'timing' voorafgaand aan het criterium 'draagvlak' in de 3e zeef van het model.

Naar aanleiding van de overheveling van het criterium 'timing' van de 2e naar de 3e zeef van het model, vraag ik mij af of voor meer (sub)criteria in de 2e zeef van het model geldt dat ze eigenlijk in de 3e zeef van het model thuishoren. Ten behoeve van hoofdstuk 6 van mijn proefschrift ben ik alvast begonnen met het lezen over de casus 'wettelijk rookverbod in de Horeca'. Wat me opvalt, is het belang dat politici toekennen aan flankerend beleid ter begeleiding van de implementatie van rookverboden. Bij het preventieplan van Clarian is er ook sprake van flankerend beleid (Andrews, 2007):

> *"But employees rebelled, even though the company [Clarian] offered free lifestyle and nutrition coaching, stop-smoking programs, fitness centers, and other wellness activities."*

Het is zelfs zo, dat de Amerikaanse overheid in federale wetgeving heeft vastgelegd, dat werkgevers in geval van een collectieve zorgverzekering alleen premiedifferentiatie op basis van gezondheidsfactoren mogen toepassen, als de zorgverzekering deel uitmaakt van een breder gezondheidsplan dat ook een welzijnsprogramma bevat (zie in bijlage 8 de toelichting op §f van de HIPAA). In dit verband kan het welzijnsprogramma als flankerend beleid bij de zorgverzekering worden opgevat.

In versie 6 van het model valt 'flankerend beleid' onder diverse criteria van de 2e zeef van het model. Met flankerend beleid – zoals voorlichting – kan de 'effectiviteit' van de preventiemaatregel worden vergroot. Met flankerend beleid – zoals privacybeschermende maatregelen – kan de 'indringendheid' van de preventiemaatregel worden verkleind. Met flankerend beleid – zoals financiële compensatie van onterecht benadeelden – kunnen 'onrechtvaardige' aspecten van de preventiemaatregel worden gecompenseerd. Vanwege het kennelijke belang van 'flankerend beleid' voor de rechtvaardiging van de toepassing van sommige preventiemaatregelen, kies ik ervoor om van 'flankerend beleid' een afzonderlijk criterium te maken. Aan de hand van de casus 'wettelijk rookverbod in de Horeca' zal ik in hoofdstuk 15 toetsen of dit terecht is.

Flankerend beleid is weliswaar van invloed op de effecten en neveneffecten (2e zeef van het model) van de preventiemaatregel, maar behoort zelf niet tot de effecten en neveneffecten. Het criterium 'flankerend beleid' betreft de implementatie van de preventiemaatregel en hoort dus thuis in de 3e zeef 'implementatie' van het model. Overigens geldt voor alle criteria van de 3e zeef van het model dat ze van invloed zijn op de effecten en neveneffecten (2e zeef) van de preventiemaatregel. Als bijvoorbeeld het draagvlak (criterium uit de 3e zeef) voor een preventiemaatregel groot is, heeft dat een gunstige invloed op de naleving en daarmee op de effectiviteit van de maatregel. Omdat de preventiemaatregel van Clarian niet daadwerkelijk is geïmplementeerd, is de casus Clarian minder geschikt om de interactie en het onderscheid tussen de criteria van de 2e en 3e zeef helder uit te werken. De volgende casus waaraan ik het model toets – het wettelijk rookverbod in de horeca – is wel daadwerkelijk geïmplementeerd. Bij de beoordeling van de gerechtvaardigdheid van de implementatie (3e zeef) van het wettelijk rookverbod in de horeca zal ik de interactie en het onderscheid tussen de criteria van de 2e en 3e zeef van het model uitwerken.

Het toevoegen van een ***criterium 'flankerend beleid'*** aan de 3e zeef van het model heeft als bijkomend voordeel dat ik hiermee de clusterstructuur van het model kan herstellen (iedere zeef van het model is opgebouwd uit clusters van twee criteria),

die door de overheveling van het criterium ‘timing’ van de 2e naar de 3e zeef van het model verstoord is.

Welk flankerend beleid nodig is, is in belangrijke mate afhankelijk van de ‘timing’ van en het ‘draagvlak’ voor de implementatie van de preventiemaatregel. Daarom plaats ik het nieuwe criterium ‘flankerend beleid’ na de criteria ‘timing’ en ‘draagvlak’ in de 3e zeef van het model. De ‘uitvoerbaarheid’ (haalbaarheid en houdbaarheid) van het flankerend beleid dient ook beoordeeld te worden. Daarom plaats ik het criterium ‘flankerend beleid’ voor het criterium ‘uitvoerbaarheid’ in de 3e zeef van het model.

Door het overhevelen van het criterium ‘timing’ van de 2e naar de 3e zeef van het model, is ook de clusterstructuur van de 2e zeef van het model verstoord (de 2e zeef van het model telt hierdoor een oneven aantal criteria, terwijl iedere zeef van het model is opgebouwd uit clusters van twee criteria). Dit los ik op door het samenvoegen van de criteria ‘kosteneffectiviteit’ en ‘lasten-batenverhouding’ tot één criterium ‘lasten-batenverhouding’. Een belangrijke overweging hierbij is, dat zowel bij de casus ‘Clarian Health’ als bij de casus ‘wettelijk rookverbod in de Horeca’ de kosteneffectiviteit van ondergeschikt belang blijkt[2].

In overzicht 16 (zie de volgende pagina) vergelijk ik het model voorafgaand en na afloop van de toetsing van het model aan de casus ‘Clarian Health’. In versie 7 van het model (zie overzicht 16) zijn alle bovenstaande wijzigingen verwerkt.

Met inachtneming van de in deze paragraaf (§9.2) besproken wijzigingen kom ik op basis van de beoordeling van de gerechtvaardigdheid van het preventieplan van Clarian Health aan de hand van het model tot de volgende conclusies over het model. De beoordeling van de casus Clarian aan de hand van het model heeft geen aanwijzingen opgeleverd dat het model onvolledig of onjuist is. Voor de beoordeling van de gerechtvaardigdheid van het preventieplan van Clarian is het model goed ‘praktisch toepasbaar’ gebleken. Naarmate het model aan meer verschillende casuïstiek wordt getoetst, ontstaat er meer zekerheid over de juistheid, volledigheid en praktische toepasbaarheid van het model.

2. Tijdens het schrijven van deze laatste paragraaf van hoofdstuk 9 ben ik inmiddels ver gevorderd met mijn ‘literatuurstudie’ over de casus ‘wettelijk rookverbod in de Horeca’.

Overzicht 16: vergelijking van versie 6 en 7 van het model

**Voor toetsing aan casus 'Clarian':**	**Na toetsing aan casus 'Clarian':**
**versie 6**	**versie 7**
**Eerste zeef: logica van het ontwerp**	**Eerste zeef: logica van het ontwerp**
Cluster 1	***Cluster 1***
Schadelijkheid	Schadelijkheid
Noodzakelijkheid	Noodzakelijkheid
Cluster 2	***Cluster 2***
Oorzakelijkheid	Oorzakelijkheid
Verantwoordelijkheid	Verantwoordelijkheid
Cluster 3	***Cluster 3***
Gerichtheid	Gerichtheid
Geschiktheid	Geschiktheid
**Tweede zeef: effecten en neveneffecten**	**Tweede zeef: effecten en neveneffecten**
Cluster 4	***Cluster 4***
Effectiviteit	Effectiviteit
Indringendheid	Indringendheid
Cluster 5	***Cluster 5***
Kosteneffectiviteit	
Lasten-batenverhouding	Lasten-batenverhouding
Cluster 6	
Rechtvaardigheid	Rechtvaardigheid
Rechtsgelijkheid	
**Derde zeef: implementatie**	**Derde zeef: implementatie**
Cluster 7	***Cluster 6***
	Timing
Draagvlak	Draagvlak
	Cluster 7
	Flankerend beleid
Uitvoerbaarheid	Uitvoerbaarheid

10 Inleiding bij casus ‘wettelijk rookverbod in horeca’ (versie 8 van het model)

10.1 Inleiding

Na toetsing van de juistheid, volledigheid en praktische toepasbaarheid van het model aan de casus Clarian Health (hoofdstuk 5 t/m 9) moest gekozen worden of ik het model zou toetsen aan nog één grote praktijkcasus of aan een aantal kleinere cases. In overleg met mijn begeleiders is gekozen voor nog één grote casus: het wettelijk rookverbod in de Nederlandse horeca. Deze keuze is gemaakt op basis van de volgende argumenten:

- Bij de toetsing van het model aan de casus Clarian heeft plaatsgevonden aan de hand van een groot bronbestand (zie hoofdstuk 5). Toch kwam ik tot de conclusie (zie §9.1) dat er nog informatie ontbrak die eigenlijk wel voor de beoordeling nodig was. Bij de toetsing van het model aan een volgende praktijkcasus wilde ik dit voorkomen.
- Uit de toetsing van het model aan de casus Clarian bleek dat een gedetailleerde beoordeling leidde tot nieuwe inzichten over het preventieplan van Clarian en ten behoeve van het model.
- Eén grote casus zou ik veel grondiger (gedetailleerder) kunnen analyseren dan een aantal kleine cases.
- Aan het begin van hoofdstuk 5 heb ik beschreven aan de hand van welke criteria ik heb gezocht naar praktijkcases om het model aan te toetsen. Toen ik in 2009 naar één of meer nieuwe praktijkcases zocht, bleek eigenlijk alleen het wettelijk rookverbod in de Nederlandse horeca, dat in juli 2008 van kracht was geworden, in grote mate aan alle criteria te voldoen.

In de hoofdstukken 10 t/m 14 wordt het model getoetst aan de casus ‘wettelijk rookverbod in de horeca’. Preciezer geformuleerd betreft deze casus de wettelijke bescherming van niet-rokers tegen blootstelling aan tabaksrook in de Nederlandse horeca. Dat de reikwijdte van dit rookverbod in de wet- en regelgeving is beperkt tot de bescherming van werknemers in de horeca tegen passief roken, wordt toegelicht in paragraaf 10.3. De bescherming van niet-rokers tegen de blootstelling aan

tabaksrook in de horeca kan in theorie worden gerealiseerd door (ondermeer[1]) de invoering van een rookverbod, de afzondering van rokers in een rookruimte en/of de zuivering van de lucht van tabaksrook via een ventilatiesysteem. Voor de horeca heeft de Nederlandse overheid vooralsnog gekozen voor een wettelijk rookverbod in combinatie met de mogelijkheid van een rookruimte voor rokers.

Het wettelijk rookverbod in de Nederlandse horeca is op 1 juli 2008 in werking getreden. Het is gebaseerd op het recht op een rookvrije werkplek voor werknemers, dat is vastgelegd in artikel 11a lid 1 van de Tabakswet. Het wettelijk recht op een rookvrije werkplek voor werknemers is op 1 januari 2004 van kracht geworden. Van 1 januari 2004 tot 1 juli 2008 waren werkgevers in de horeca echter vrijgesteld van de wettelijke verplichting om hun werknemers een rookvrije werkplek te bieden. Aan de invoering van het wettelijk recht op een rookvrije werkplek in 2004 zijn wettelijke rookverboden in overheidsgebouwen en gebouwen in het onderwijs en de gezondheidszorg voorafgegaan. Deze rookverboden zijn gebaseerd op de artikelen 10 en 11 van de Tabakswet en al op 1 maart 1990 in werking getreden[2]. Bij de overheid en in het onderwijs en de gezondheidszorg is een wettelijk rookverbod dus 14 jaar eerder ingevoerd dan in het bedrijfsleven en 18 jaar eerder dan in de horeca.

De invoering van het wettelijk rookverbod in de Nederlandse horeca kent een lange voorgeschiedenis, die relevant is voor de beoordeling van de gerechtvaardigdheid van dit rookverbod. In het literatuuronderzoek ten behoeve van de toetsing van het model aan de casus 'wettelijk rookverbod in de horeca' ben ik daarom teruggegaan naar de oorsprong van de eerste wettelijke rookverboden in Nederland ter bescherming van niet-rokers. Dit waren de eerder genoemde rookverboden in overheidsgebouwen en gebouwen in het onderwijs en de gezondheidszorg uit 1990. In haar analyse van de vormgeving en naleving van de wettelijke rookverboden in de horeca grijpt Van der Meij ook terug naar deze eerste wettelijke rookverboden uit 1990 (Meij van der, 2009, p.711). In de 'Handleiding invoering rookvrije horeca, sport en kunst/cultuur' grijpt het ministerie van Volksgezondheid Welzijn en Sport (VWS) eveneens terug naar deze eerste wettelijke rookverboden uit 1990 (VWS, 27 juni 2008, p.5).

Het vervolg van hoofdstuk 10 is als volgt opgebouwd. Het literatuuronderzoek dat ik heb gedaan naar het wettelijk rookverbod in de horeca wordt beschreven in

---

1. In theorie kunnen niet-rokers bijvoorbeeld ook een gasmasker opzetten en misschien kan er nog meer worden bedacht.
2. Staatsblad, 1988, nr.342.

§10.2. De totstandkoming van dit rookverbod wordt beschreven in §10.3. De na het literatuuronderzoek aangepaste versie van het model (versie 8) wordt beschreven in §10.4. In de hoofdstukken 11, 12 en 13 beoordeel ik de gerechtvaardigdheid van het wettelijk rookverbod in de horeca aan de hand van versie 8 van het beoordelingsmodel (zie overzicht 17 in §10.4). Ik volg hierbij de wijze van beoordeling zoals samengevat in §4.5. In hoofdstuk 14 geef ik in §14.1 een overall beoordeling van de gerechtvaardigdheid van het wettelijk rookverbod in de horeca. Ten slotte beoordeel ik in §14.2 de juistheid, volledigheid en praktische toepasbaarheid van het model op basis van de voorafgaande hoofdstukken.

10.2 Literatuuronderzoek naar wettelijk rookverbod in de horeca

Het wettelijk rookverbod in de horeca is ingesteld door de Nederlandse overheid. Daarom baseer ik de toetsing van het model aan deze casus op de formele documenten waarmee de Nederlandse overheid de totstandkoming en toepassing van dit rookverbod tot dusver heeft onderbouwd. Mijn literatuuronderzoek betreft een periode van ruim 25 jaar (november 1984 t/m juni 2011).

Ten behoeve van mijn onderzoek heb ik de volgende documenten gescreend op relevantie voor mijn onderzoek en – voor zover relevant – gelezen:

- van de website van het ministerie van Volksgezondheid Welzijn en Sport (VWS)[3]:
  - de ‘nieuwsberichten’ over het onderwerp ‘roken’ (juni 2007 t/m juni 2011)[4];
  - de ‘documenten en publicaties’ over het onderwerp ‘roken’ (mei 2007 t/m juni 2011)[5];
- van de website van de rijksoverheid[6]:
  - de parlementaire documenten[7] die worden gevonden met de zoektermen ‘tabaksontmoedigingsbeleid’ en ‘rookvrije’ (maart 1995 t/m juni 2011);
  - de wetten en wettelijke regelingen[8] die worden gevonden met de zoekterm ‘Tabakswet’ en de bijbehorende wetstechnische informatie (maart 1986 t/m juni 2011)[9];

3. http://www.rijksoverheid.nl/ministeries/vws
4. http://www.rijksoverheid.nl/onderwerpen/roken/nieuws
5. http://www.rijksoverheid.nl/onderwerpen/roken/documenten-en-publicaties
6. http://www.overheid.nl (deze website bevat parlementaire documenten vanaf 1995)
7. https://zoek.officielebekendmakingen.nl/zoeken/parlementaire_documenten
8. http://wetten.overheid.nl/zoeken/
9. http://www.rijksoverheid.nl/onderwerpen/roken/documenten-en-publicaties

- van de website van de nieuwe Voedsel en Waren Autoriteit (nVWA)[10]:
  - de 'nieuwsberichten' over het onderwerp 'naleefniveau rookverbod';
  - kwartaalrapportages over het naleefniveau van het rookverbod (Intraval 2008-2011);
- van de website van de Koninklijke Bibliotheek[11]:
  - enkele documenten uit de ontwerpperiode van de Tabakswet (1984);
- de volgende onderzoeksrapporten die in opdracht van de Nederlandse overheid zijn opgesteld ter onderbouwing van het rookbeleid in de horeca:
  - onderzoek naar de resultaten van de zelfregulering van het rookbeleid in de horeca (Nuijten. Mulier Instituut. 2007) en de resultaten van het rookbeleid in de sport (Diepen. TNS/NIPO. 2007);
  - onderzoek naar de gevolgen en de naleving van het rookverbod in de horeca (Gelder. RIVM. 2008) (Intraval. 2008-2011) (Intraval. 2008) (Intraval, 2009) (Diepen. TNS/NIPO. 2008) (Vogels. Trimbos. 2009) (Schieveen. TNS/NIPO. 2009) (Dekker. Regioplan. 2009);
  - onderzoek naar (alternatieven voor) rookvrije ruimten (Gids de/ TNO en Opperhuizen/ RIVM. 2004), (Talhout. RIVM. 2009) (Brunekreef. QANU. 2009) (Opperhuizen. RIVM. 2010);
- de belangrijkste internationale documenten waarnaar de rijksoverheid verwijst ter onderbouwing van de invoering van rookverboden:
  - Raad van de Europese Unie. Resolutie van de Raad en van de Ministers van Volksgezondheid betreffende het rookverbod in ruimten bestemd voor het ontvangen van publiek (18 juli 1989)[12];
  - WHO-Kaderverdrag inzake tabaksontmoediging (Genève, 21 mei 2003)[13];
  - Europese Commissie. Groenboek. Op weg naar een rookvrij Europa: beleidsopties op EU-niveau. (EC, 2007);
  - Raad van de Europese Unie. Aanbeveling van de Raad betreffende rookvrije ruimten (30 november 2009)[14];
- enkele belangrijke documenten waarnaar in de door mij gescreende en/of gelezen documenten wordt verwezen, waarvan de belangrijkste zijn:
  - onderzoeksrapporten van de Nederlandse Gezondheidsraad (2003) en de Surgeon General van de United States (USDHHS, 2006, 2010) over de gevolgen van passief roken voor de volksgezondheid;

---

10. http://www.vwa.nl
11. http://www.statengeneraaldigitaal.nl/uitgebreidzoeken
12. Publicatieblad van de Europese Unie. (89/C 189/01).
13. http://wetten.overheid.nl
14. Publicatieblad van de Europese Unie. (2009/C 296/02).

– het Stappenplan van Koninklijke Horeca Nederland (KHN) ter zelfregulering van het rookbeleid in de horeca (KHN, 2003)[15].

Samen geven de bovenstaande documenten m.i. een volledig overzicht van de argumenten waarmee de rijksoverheid – de achtereenvolgende ministers Borst-Eilers, Hoogervorst, Klink en Schippers van VWS – de totstandkoming en instandhouding van wettelijke rookverboden tot dusver heeft onderbouwd[16].

10.3 Totstandkoming van het wettelijk rookverbod in de horeca

In november 1984 sturen staatssecretarissen Van der Reijden van Welzijn Volksgezondheid en Cultuur (WVC) en Van Zeil van Economische Zaken (EZ) het wetsvoorstel voor de Tabakswet naar de Tweede Kamer (wetvoorstel betreffende 'Maatregelen ter beperking van het tabaksverbruik in het bijzonder ter bescherming van de niet-roker'). Artikel 9 en 10 van dit wetsvoorstel betreffen de bescherming van de niet-roker[17]:

> *"Artikel 9*
> *1. Voor de instellingen, diensten en bedrijven die door de Staat en de openbare lichamen worden beheerd, worden door het bevoegde orgaan zodanige maatregelen getroffen, dat van de daardoor geboden voorzieningen gebruik kan worden gemaakt en de werkzaamheden daarin kunnen worden verricht zonder dat daarbij hinder van het gebruik van tabaksprodukten wordt ondervonden.*
>
> *Artikel 9*
> *2. Bij algemene maatregel van bestuur worden, de Nationale Raad voor de Volksgezondheid gehoord, richtlijnen vastgesteld ten aanzien van het treffen van maatregelen als bedoeld in het eerste lid.*
>
> *Artikel 10*
> *1. Bij algemene maatregel van bestuur kan aan degenen die - anders dan in een hoedanigheid als bedoeld in artikel 9 - het beheer hebben over: a. inrichtingen voor gezondheidszorg of maatschappelijke dienstverlening; b. inrichtingen voor sport of sociaal-cultureel werk; c. inrichtingen voor onderwijs; voor zover die inrichtingen behoren tot bij de maatregel aangewezen categorieën, de verplichting worden opge-*

15. TK, 2003–2004, 29200 XVI, nr.278.
16. Van 22 juli 2002 tot 16 oktober 2002 was E. Bomhoff minister van VWS en van 16 oktober 2002 tot 27 mei 2003 A. de Geus. Naar mijn oordeel zijn beiden, vanwege hun korte periode als minister van VWS, niet van grote invloed geweest op de totstandkoming van de wettelijke rookverboden.
17. TK, 1984-1985, 18749, nr.1-2.

legd tot het treffen van maatregelen als bedoeld in artikel 9, eerste lid.
2. Artikel 9, tweede lid, is van overeenkomstige toepassing."

De memorie van toelichting bij dit wetsvoorstel stelt[18]:

"Hoe zwaar ook de belangen van volksgezondheid moeten wegen, het roken kan niet op stel en sprong uit onze maatschappij worden verbannen. Het nastreven daarvan op langere termijn zal ook grote moeilijkheden ondervinden. Feitelijk zal daarvoor op mondiaal niveau een grote mate van wilsovereenstemming moeten bestaan. De regering kiest daarom voor een beleid op langere termijn, waarvan de beperking van het tabaksgebruik en, in het bijzonder, de bescherming van de jeugd en van de niet-roker de hoofddoelstellingen zijn." (p.7-8)

"…Daar komt bij dat het recht op eerbiediging van de persoonlijke levenssfeer, zoals neergelegd in artikel 10, eerste lid, van de Grondwet, met zich brengt dat de overheid terughoudendheid moet betrachten bij het stellen van regels ter bescherming van de niet-roker. Zoals de niet-roker het recht heeft op bescherming van zijn lichamelijke integriteit heeft de roker er recht op dat overheidsbemoeienis op dit punt zich niet uitstrekt tot zijn persoonlijke levenssfeer. Dit betekent dat de overheid zich heeft te onthouden van het stellen van regels ten aanzien van het roken in de privésfeer met het oogmerk de niet-roker te beschermen." (p.12)

Belangrijk is om op te merken dat de bescherming van de niet-roker tegen passief roken een hoofddoelstelling is. Dit is een bredere doelstelling dan de bescherming van werknemers tegen passief roken. Daarom richt artikel 9 zich zowel op het gebruik van bepaalde voorzieningen (bescherming van klanten c.q. bezoekers) als op het verrichten van werkzaamheden in deze voorzieningen (bescherming van werknemers). De overheid definieert hier de persoonlijke levenssfeer als de privésfeer c.q. de thuissituatie van de roker.

In 1988 wordt de Tabakswet aangenomen (Meij van der, 2009):

"Op 10 maart 1988 werd het voorstel aangenomen voor de 'Wet maatregelen ter beperking van tabaksgebruik, in het bijzonder ter bescherming van de niet-roker' [19] *naar zijn datum van inwerkingtreding de Tabakswet 1990 genoemd. In artikel 10 lid 1 van deze wet werden de Staat en de openbare lichamen verplicht om voor alle instellingen, diensten en bedrijven die onder hun beheer vielen, maatregelen te treffen ter voorkoming van hinder van het gebruik van tabaksproducten. In lid 2 van dit artikel stond dat onder deze maatregelen werden verstaan 'het instellen en handhaven van een rookverbod' en dat bij Algemene Maatregel van Bestuur (AMvB) aanvullende regels konden worden gesteld. Deze aanvullende maatregelen werden gere-*

18. TK, 1984-1985, 18749, nr.3.
19. Staatsblad 1988, nr.342.

geld in het 'Besluit beperking verkoop en gebruik tabaksproducten'.[20] *In dit Besluit werd aangegeven dat het rookverbod in alle ruimten van de overheidsinstellingen moest gelden, behalve in de open lucht of in een daartoe aangewezen rookruimte. Ook moest daar waar het rookverbod gold, dit duidelijk zijn aangegeven."* (p.711)

In 1989 neemt de Raad van de Europese Unie een resolutie aan waarin de lidstaten worden opgeroepen tot het instellen van rookverboden in ruimten voor het ontvangen van publiek[21]:

"De Raad van de Europese Gemeenschappen en de ministers van Volksgezondheid van de Lid-Staten, … nodigen de Lid-Staten uit: door middel van wettelijke regelingen of anderszins conform de nationale praktijken en omstandigheden, de volgende maatregelen te nemen:

1. *rookverbod in gesloten ruimten, bestemd voor het ontvangen van publiek, van openbare of particuliere gebouwen als bedoeld in de bijlage. De Lid-Staten kunnen de lijst in de bijlage aanvullen;*
2. *rookverbod in alle middelen voor collectief vervoer;*
3. *zo nodig, reservering van duidelijk afgebakende ruimten voor rokers in bovengenoemde gebouwen en, zo mogelijk, in de middelen voor collectief vervoer, met name voor lange trajecten;*
4. *bij geschillen, buiten de voor rokers gereserveerde ruimten het recht op gezondheid van niet-rokers laten prevaleren boven het recht van rokers om te roken;"*

"In punt 1 van de resolutie bedoelde openbare en particuliere gebouwen (niet limitatieve lijst)

1. *Gebouwen waar, al of niet tegen betaling, diensten worden verleend aan het publiek, met inbegrip van de verkoop van goederen.*
2. *Ziekenhuizen en klinieken en alle andere gebouwen voor de gezondheidszorg.*
3. *Bejaardentehuizen.*
4. *Lagere scholen en andere plaatsen waar kinderen of jongeren worden opgevangen of gehuisvest.*
5. *Gebouwen waar hoger onderwijs of beroepsopleiding wordt verstrekt.*
6. *Gebouwen waar voorstellingen plaatsvinden (bioscopen, theaters, enz.) alsmede radio- en televisiestudio's waar publiek wordt ontvangen.*
7. *Gebouwen waar tentoonstellingen worden gehouden.*
8. *Gebouwen en gesloten ruimten waar sport wordt beoefend.*
9. *Gesloten ruimten in metrostations, spoorwegstations, havens en luchthavens."*

In 1990 treedt de Tabakswet in werking.

---

20. Staatsblad, 1989, nr.612.
21. Publicatieblad van de Europese Unie. (89/C 189/01).

In 1996 maakt minister Borst-Eilers van Volksgezondheid Welzijn en Sport (VWS) in een brief aan de Tweede Kamer onderscheid tussen rookverboden ter bescherming van het publiek en van werknemers[22]:

> *"Openbare ruimten*
> *… Bovendien zal ik nagaan welke juridische mogelijkheden er zijn om het rookverbod uit te breiden tot de voor het publiek toegankelijke ruimten van bedrijven met een publieksfunctie. Daarbij gaat het erom hoe de Tabakswet ware aan te passen ten einde rookverboden in bepaalde sectoren van het bedrijfsleven, zoals treinen, postkantoren, bankfilialen etcetera, wettelijk mogelijk te maken. Met deze maatregelen kan verder tegemoet worden gekomen aan de EU-resolutie van 18 juli 1989 inzake het instellen van een rookverbod in ruimten bestemd voor het ontvangen van publiek."* (p.6-7)

> *"Bedrijfsleven*
> *… In een adviesaanvraag uit 1992 aan de Stichting van de Arbeid is als uitgangspunt van het regeringsbeleid gesteld dat in Nederland een ieder zijn werk moet kunnen doen zonder daarbij hinder of gezondheidsschade te ondervinden van het roken door een collega in de onmiddellijke omgeving. … Het is in de eerste plaats de verantwoordelijkheid van werkgevers en werknemers op ondernemingsniveau om tot afspraken te komen ter voorkoming en/of beperking van blootstelling aan tabaksrook uit de omgeving. De Stichting van de Arbeid kon zich vinden in deze uitgangspunten en heeft een en ander vertaald in een Aanbeveling aan werkgevers en werknemers."* (p.7)

In april 1999 stuurt minister Borst-Eilers een wetsvoorstel tot wijziging van de Tabakswet aan de Tweede Kamer. Ten tijde van dit wetsvoorstel luidt de tekst van de Tabakswet, waarin de bescherming van niet-rokers tegen passief roken is geregeld, als volgt[23]:

> *"Artikel 10*
> 1. *Voor de instellingen, diensten en bedrijven die door de Staat en de openbare lichamen worden beheerd, worden door het bevoegde orgaan zodanige maatregelen getroffen, dat van de daardoor geboden voorzieningen gebruik kan worden gemaakt en de werkzaamheden daarin kunnen worden verricht zonder dat daarbij hinder van het gebruik van tabaksprodukten wordt ondervonden.*
> 2. *Tot de maatregelen, bedoeld in het eerste lid, behoort in ieder geval het instellen en handhaven van een verbod tabaksprodukten te gebruiken in ruimten, behorende tot bij algemene maatregel van bestuur, aangewezen categorieën. Op het*

22. TK, vergaderjaar 1995–1996, 24743, nr.1.
23. Tekst van artikel 10 en 11 van de Tabakswet geldend op 26 juli 1995 (http://lexius.nl/tabakswet/26-07-1995) en op 21 juni 2006 (http://lexius.nl/tabakswet/21-06-2000).

verbod kunnen, overeenkomstig bij de algemene maatregel van bestuur gestelde regelen, beperkingen worden aangebracht.

Artikel 11

1. *Bij algemene maatregel van bestuur kan aan degenen die - anders dan in een hoedanigheid als bedoeld in artikel 10 - het beheer hebben over inrichtingen voor gezondheidszorg, maatschappelijke dienstverlening, sport, sociaal-cultureel werk of onderwijs, voor zover die inrichtingen behoren tot bij algemene maatregel van bestuur aangewezen categorieën, de verplichting worden opgelegd tot het treffen van maatregelen als bedoeld in artikel 10, eerste lid.*
2. *Artikel 10, tweede lid, is van overeenkomstige toepassing."*

De artikelen 10 en 11 van de Tabakswet, zoals hierboven weergegeven, komen grotendeels overeen met respectievelijk de artikelen 9 en 10 uit het wetsvoorstel voor de Tabakswet uit 1984. Belangrijk is om op te merken dat het doel van artikel 10 van de Tabakswet, zoals hierboven weergegeven, tweeledig is. Enerzijds de bescherming van werknemers op de werkplek tegen passief roken en anderzijds de bescherming van gebruikers van openbare voorzieningen tegen passief roken. De reikwijdte van artikel 10 is beperkt tot overheidsorganisaties. Artikel 11 betreft een uitbreiding van de reikwijdte van artikel 10 met semi-overheidsorganisaties.

In het wetsvoorstel tot wijziging van de Tabakswet stelt minister Borst-Eilers voor om de reikwijdte van artikel 10 nog verder uit te breiden door een artikel 11a aan de Tabakswet toe te voegen. Het doel van het beoogde artikel 11a is tweeledig. Enerzijds heeft artikel 11a ten doel om ook werknemers die niet bij de (semi)overheid in dienst zijn te kunnen beschermen tegen passief roken. Anderzijds heeft artikel 11a ten doel om gebruikers van openbare voorzieningen, die niet door de (semi)overheid worden beheerd, tegen passief roken te kunnen beschermen. In het wetsvoorstel van minister Borst-Eilers luidt de formulering van conceptwetsartikel 11a als volgt[24]:

"Artikel 11a

1. *Bij algemene maatregel van bestuur <u>kunnen</u>:*
   a. *werkgevers worden verplicht zodanige maatregelen te treffen dat <u>werknemers</u> in staat worden gesteld hun werkzaamheden te verrichten zonder daarbij hinder of overlast van roken door anderen te ondervinden;*
   b. *exploitanten van middelen voor personenvervoer worden verplicht zodanige maatregelen te treffen dat <u>passagiers</u> in staat worden gesteld hun reis te volbrengen zonder daarbij hinder of overlast van roken te ondervinden;*
   c. *Nederlandse luchtvaartmaatschappijen worden verplicht zodanige maatrege-*

24. TK, 1998-1999, 26472, nrs.1-2.

len te treffen dat passagiers aan boord van hun vliegtuigen tijdens het gebruik voor de burgerluchtvaart op vluchten van en naar op Nederlands grondgebied gelegen luchthavens in staat worden gesteld hun reis te volbrengen zonder daarbij hinder of overlast van roken te ondervinden;

d. *diegenen die – anders dan in een hoedanigheid als bedoeld in artikel 10 of 11 – het beheer hebben over voor het publiek toegankelijke gebouwen, voor zover die gebouwen behoren tot bij algemene maatregel van bestuur aangewezen categorieën, worden verplicht tot het treffen van maatregelen als bedoeld in artikel 10, eerste lid.*

2. *Artikel 10, tweede lid, is van overeenkomstige toepassing."*

Conceptwetsartikel 11a richt zich zowel op werknemers als op gebruikers van voor het publiek toegankelijke voorzieningen:
- sub a richt zich op werknemers;
- sub b en c richten zich op gebruikers (passagiers);
- sub d richt zich op werknemers en gebruikers (sub d verwijst naar artikel 10 lid 1 en dat is gericht op werknemers en gebruikers).

Merk op dat zowel sub a als sub d van conceptwetsartikel 11a lid 1 kunnen worden toegepast op de horeca. Sub a ter bescherming van werknemers tegen passief roken. Sub d ter bescherming van werknemers en gebruikers (bezoekers) tegen passief roken.

In mei 2001 stelt Tweede Kamerlid Hermann in een amendement[25] op het '*Wetsvoorstel tot wijziging van de Tabakswet*' voor om conceptwetsartikel 11a meer verplichtend te maken door ondermeer het eerste zinsdeel van het door minister Borst-Eilers voorgestelde conceptwetsartikel 11a ("*Bij algemene maatregel van bestuur kunnen:*") te schrappen.

In april 2002 wordt de '*Wet van 18 april 2002 tot wijziging van de Tabakswet*' aangenomen. Ten gevolge van het bovengenoemde amendement van Tweede Kamerlid Hermann is artikel 11a verplichtend geformuleerd[26]:

Artikel 11a

1. *Werkgevers zijn verplicht zodanige maatregelen te treffen dat werknemers in staat worden gesteld hun werkzaamheden te verrichten zonder daarbij hinder of overlast van roken door anderen te ondervinden.*
2. *Exploitanten van middelen voor personenvervoer zijn verplicht zodanige maatregelen te treffen dat passagiers in staat worden gesteld hun reis te volbrengen zonder daarbij hinder of overlast van roken te ondervinden.*

25. TK, 200-2001, 26472, nr.13.
26. Staatsblad, 2002, nr.201.

3. *Nederlandse luchtvaartmaatschappijen zijn verplicht zodanige maatregelen te treffen dat passagiers aan boord van hun vliegtuigen tijdens het gebruik voor de burgerluchtvaart op vluchten van en naar op Nederlands grondgebied gelegen luchthavens in staat worden gesteld hun reis te volbrengen zonder daarbij hinder of overlast van roken te ondervinden.*
4. *Diegenen die – anders dan in een hoedanigheid als bedoeld in artikel 10 of 11 – het beheer hebben over voor het publiek toegankelijke gebouwen, voor zover die gebouwen behoren tot bij algemene maatregel van bestuur aangewezen categorieën, zijn verplicht tot het treffen van maatregelen als bedoeld in artikel 10, eerste lid.*
5. *Bij algemene maatregel van bestuur kunnen op de in dit artikel bedoelde verplichtingen beperkingen worden aangebracht. Zo kan worden bepaald dat de verplichtingen, bedoeld in het eerste lid, niet gelden voor bij die maatregel aangewezen:*
   a. *categorieën van werkgevers;*
   b. *ruimten in gebouwen;*
   c. *andere plaatsen waar werkzaamheden worden verricht.*

   Daarbij kunnen nadere regels worden gesteld."

Artikel 11a lid 1 wordt pas op 1 januari 2004 van kracht, zodat werkgevers, werknemers en de overheid de gelegenheid krijgen zich op de invoering van de rookvrije werkplek voor te bereiden.

Op 3 oktober 2003 schrijft minister Hoogervorst van VWS in een brief aan het bestuur van Koninklijke Horeca Nederland (KHN) dat een rookvrije horeca op termijn onvermijdelijk is en dat werknemers en gasten in de horeca tegen passief roken beschermd dienen te worden[27]:

> *"Wij kwamen gezamenlijk tot de conclusie dat een rookvrije horeca op termijn onvermijdelijk is. … Gezien de gezondheidsrisico's bij blootstelling aan tabaksrook zal het stappenplan maatregelen moeten bevatten die daadwerkelijk bijdragen aan het beschermen van de gezondheid van werknemers en gasten in de horeca."*

Op 30 oktober 2003 schrijft minister Hoogervorst aan de vaste commissie van de Tweede Kamer voor VWS dat ook bezoekers in de horeca tegen passief roken beschermd dienen te worden[28]:

> *[Vraag van de commissie] "Ongeveer 11 000 horecabedrijven hebben geen werknemers. Het ministerie van Volksgezondheid stelt dat tijdens het bestuurlijke overleg*

27. POG/GB 2.411.992. Afspraken bestuurlijk overleg 8 september 2003. Den Haag, 3 oktober 2003.
28. TK. 2003–2004, 29 200 XVI, nr.25.

van 8 september (VWS-KHN) is afgesproken dat ook die bedrijven worden meegenomen in het stappenplan, waarom?" (p.12)

[Antwoord van minister Hoogervorst] "Met de horeca heb ik afgesproken dat een rookvrije horeca op termijn onvermijdelijk is. De sector ziet dat zelf ook in. Op dit moment ligt de nadruk op de bescherming van de werknemers aangezien op 1 januari 2004 het recht op een rookvrije werkplek in werking treedt. Als het gaat om het beschermen van de gezondheid van de niet-roker tegen tabaksrook gaat het echter ook om de bezoekers van horecagelegenheden. Op termijn zullen daarom ook in horeca-inrichtingen zonder werknemers maatregelen genomen moeten worden." (p.12)

In december 2003 publiceert Koninklijke Horeca Nederland (KHN) de eerste versie van haar stappenplan 'Gewoon gastvrij' ter zelfregulering van het rookbeleid in de horeca (KHN, 2003). In dit stappenplan besteedt KHN extra aandacht aan de bescherming van kinderen (dit zijn meestal geen werknemers) tegen tabaksrook:

"Met minister Hoogervorst is afgesproken dat Koninklijk Horeca Nederland in zijn stappenplan nadrukkelijk aandacht zal besteden aan horecagelegenheden waar veel kinderen komen. IJsbedrijven, pannenkoekenrestaurants en horecagelegenheden in sport- en recreatiebedrijven voldoen aan dit criterium. Gestimuleerd moet worden dat kinderen in deze gelegenheden kunnen verblijven zonder overlast van rook te ervaren. De doelstellingen voor dit soort horecabedrijven zijn dan ook zeer scherp geformuleerd. Koninklijk horeca Nederland zal specifiek voorlichtingsmateriaal ontwikkelen voor horecabedrijven met als belangrijke doelgroep kinderen." (p.27)

Op 1 januari 2004 wordt artikel 11a lid 1 van de Tabakswet (de rookvrije werkplek voor werknemers) van kracht. De overheid acht de tijd echter nog niet rijp om de rookvrije werkplek voor werknemers ook wettelijk af te dwingen in de horeca. Om de horeca uit te zonderen van de wettelijke verplichting tot de invoering van de rookvrije werkplek wordt daarom per 1 januari 2004 ook het 'Besluit uitzonderingen rookvrije werkplek' van kracht[29]. Dit besluit regelt dat artikel 11a lid 1 van de Tabakswet (de rookvrije werkplek voor werknemers) niet van toepassing is op de horeca.

In september 2004 geeft minister Hoogervorst in een brief aan KHN zijn goedkeuring aan het door KHN bijgestelde stappenplan ter zelfregulering van het rookbeleid in de horeca over de periode 2005-2008[30].

29. Staatsblad, 2003, nr.561.
30. TK, 2003-2004, 29200 XVI, nr.278. Bijlagen bij dit Kamerstuk: brief van KHN aan minister Hoogervorst d.d. 26 augustus 2004; reactiebrief van minister Hoogervorst aan KHN, te raadplegen sinds 8 september 2004.

In juni 2007 schrijft minister Klink een brief aan de Tweede Kamer, over zijn besluit tot invoering van een rookvrije horeca zonder uitzonderingen. Volgens minister Klink is het doel van zijn besluit primair de bescherming van werknemers tegen passief roken in de horeca. De bescherming van bezoekers tegen passief roken in de horeca noemt hij een *"secundair"* doel. Minister Klink maakt niet duidelijk hoeveel waarde aan een *"secundair"* c.q. ondergeschikt doel gehecht moet worden[31]:

> *"Resultaten tweede jaar zelfregulering (2006). … De kwantitatieve doelen zijn – behoudens in enkele sectoren – gehaald, maar de resultaten zijn in kwalitatieve zin minder positief. Zeker de geringe omvang van de rookvrije delen en de – in bepaalde sectoren – weinig consequente toepassing maken duidelijk dat de rookvrije horeca en bescherming van de werknemer via zelfregulering niet bereikt zullen worden." (p.3)*

> *"In het coalitieakkoord van 7 februari 2007 staat dat «in samenspraak met de branche zal worden toegewerkt naar een rookvrije horeca in deze kabinetsperiode». Ik heb hierover gesproken met Koninklijk horeca Nederland (KHN), de Federatie van Podiumverenigingen, FNV horecabond en vakbond De Unie. KHN heeft toen, tegen de achtergrond van de passages uit het coalitieakkoord betreffende de rookvrije horeca, aangegeven dat de tijd rijp is voor een andere benadering en heeft voorgesteld om de sectoren hotels, restaurants en fastfood per 1 januari 2008 rookvrij te maken. Voor de sectoren cafés en discotheken wil KHN een apart traject dat op termijn, maar niet eerder dan 1 januari 2011, ook leidt tot een rookvrije situatie." (p.3)*

> *"Ik heb besloten om niet mee te gaan in het voorstel van KHN omdat: 1. dan niet alle werknemers (secundair: bezoekers) in de horeca beschermd worden tegen tabaksrook (ongelijke behandeling) en juist de werknemers die de hoogste blootstelling aan tabaksrook hebben (cafés, discotheken), worden uitgezonderd; …" (p.3)*

In juli 2007 stelt minister Klink van VWS in een algemeen overleg met de Tweede Kamer dat de horeca rookvrij gemaakt moet worden ter bescherming van werknemers en bezoekers tegen passief roken[32]:

> *"De doelstelling om in 40% van de cafés rookvrije zones te creëren, is gehaald. Die zones beslaan echter vaak niet meer dan 25% van de totale oppervlakte. Iemand zit dus in een rookvrije zone terwijl zijn buurman iets verderop aan het roken is. Juist vanwege de schade die meeroken veroorzaakt voor werknemers en bezoekers, moet de horeca rookvrij gemaakt worden. Als de horeca in overwegende mate ontoegankelijk is voor mensen met luchtwegenaandoeningen, kan niet worden volgehouden dat er een absolute keuzevrijheid is. … Bescherming van de gezondheid van de werkne-*

31. TK, 2006-2007, 30800 XVI, nr.149.
32. TK, 2006-2007, 30800 XVI, nr.182.

mer en van de gasten is de reden voor een rookverbod per 1 juli 2008 in de horeca, de kunst, de cultuur en de sport." (p.6-7)

Per 1 juli 2008 wordt het 'Besluit uitzonderingen rookvrije werkplek', dat sinds 1 januari 2004 van kracht is, ingetrokken[33]. Hierdoor treedt artikel 11a lid 1 van de Tabakswet – de rookvrije werkplek voor werknemers – ook in werking voor de horeca. Artikel 11a lid 1 is niet van toepassing op de horeca zonder personeel c.q. zonder werknemers. Om een gelijk speelveld te creëren tussen horeca met en zonder personeel wordt daarom tevens per 1 juli 2008 het 'Besluit uitvoering rookvrije werkplek, horeca en andere ruimten' van kracht. De nota van toelichting bij dit besluit stelt[34]:

"Dit besluit strekt er onder meer toe deze eenmanszaken in de horeca rookvrij te maken. Een belangrijk motief hiervoor is het vermijden van een tweedeling in de horeca: horecagelegenheden waar niet mag worden gerookt (om werknemers te beschermen tegen blootstelling aan tabaksrook) en horecagelegenheden waar dat wel zou mogen (omdat geen sprake is van werknemers). De grootste ondernemersorganisatie in de horecabranche, Koninklijk Horeca Nederland, acht een dergelijke tweedeling onwenselijk, omdat dit zou leiden tot een «ongelijk speelveld». … Dit zou onduidelijk zijn voor het publiek en leiden tot (een schijn van) concurrentievervalsing."

… Teneinde de eenmanszaken in de horeca rookvrij te maken, wordt hier voor het eerst gebruik gemaakt van artikel 11a, vierde lid, van de Tabakswet. Dit artikel biedt de rechtsgrondslag om al die categorieën van gebouwen rookvrij te maken, die – gelet op de artikelen 10 en 11 van de Tabakswet – niet onder de werkingssfeer van het Besluit beperking verkoop en gebruik tabaksprodukten gebracht kunnen worden en waarop ook artikel 11a, eerste lid, van de Tabakswet niet van toepassing is, omdat er niet altijd sprake hoeft te zijn van een relatie werkgever-werknemer."

Belangrijk is om op te merken dat in de bovenstaande nota van toelichting de bescherming van bezoekers tegen passief roken niet wordt genoemd als reden om eenmanshorecazaken zonder personeel rookvrij te maken.

In juni 2009 vindt in de Tweede Kamer een spoeddebat plaats over de gevolgen van een rechterlijke uitspraak over het wettelijk rookverbod in de horeca. In dit spoeddebat stelt minister Klink dat de doelstelling van dit rookverbod zich beperkt tot de bescherming van werknemers in de horeca tegen passief roken. Volgens minister Klink is de bescherming van bezoekers in de horeca tegen passief roken

33. Staatsblad, 2008, nr.123.
34. Staatsblad, 2008, nr.122.

geen doelstelling van dit rookverbod en zal dit wat hem betreft ook geen doelstelling worden[35]:

> *"De grondslag en het aangrijpingspunt voor en het oogmerk van deze wet- en regelgeving is om de werknemer te beschermen, niet meer en niet minder. Ik verwees naar de spin-off van het geheel, dus naar de effecten. De effecten zijn dat ook de horecabezoeker er uiteindelijk baat bij heeft. Ik zeg niet dat dit de reden is geweest voor de wet- en regelgeving. Dat zal het wat mij betreft ook niet worden. Het is wel een bijeffect van de maatregelen die wij hebben genomen. Ik geef maar even aan dat het een gelukkig bijeffect is, want het gaat hier om een miljoen mensen die aan astma lijden in ons land en die daadwerkelijk veel hinder ondervinden van het roken in een besloten ruimte."* (p.6623)

In juli 2009 schrijft minister Klink aan de Tweede Kamer echter weer wat anders. Nu wil hij werknemers en bezoekers in de horeca tegen passief roken beschermen[36]:

> *"Van begin af aan heb ik aangegeven dat ik hecht aan een gelijk speelveld voor de horeca en een gelijke behandeling van alle horeca. Dit om oneerlijke concurrentie zoveel mogelijk te voorkómen. Uw Kamer heeft dit uitgangspunt onderschreven. Met de uitspraken van de gerechtshoven in Den Bosch en Leeuwarden is een onduidelijke situatie ontstaan met betrekking tot de toepassing van de regelgeving door horecaondernemers zonder personeel. Die onduidelijkheid wil ik met genoemde uitgangspunten in het achterhoofd graag wegnemen. Concreet betekent dit dat ik op korte termijn voornemens ben om het Besluit uitvoering rookvrije werkplek, horeca en andere ruimten aan te passen. Het voornemen is er op gericht om voor alle horeca-inrichtingen een eensluidende verplichting te laten gelden, namelijk de verplichting om zodanige maatregelen te treffen dat van een horeca-inrichting gebruik kan worden gemaakt en de werkzaamheden daarin kunnen worden verricht zonder daarbij hinder of overlast van roken te ondervinden. … Verder beraad ik me op mogelijke wijziging van de huidige formulering van de rookverboden in de artikelen 10, 11 en 11a van de Tabakswet."* (p.2)

In september 2009 stuurt minister Klink het 'Ontwerpbesluit houdende verduidelijking van het Besluit uitvoering rookvrije werkplek, horeca en andere ruimten'[37] naar de Tweede Kamer. In de aanbiedingsbrief bij dit ontwerpbesluit schrijft minister Klink aan de Tweede Kamer dat het uitgangspunt van de Tabakswet (die in 1990 in werking is getreden) steeds heeft bestaan uit de bescherming van werknemers en bezoekers in openbare ruimten tegen passief roken. Daarom wil hij het

35. TK, 2008-2009, vergaderingnr.84, p.6613-6629 (dossiernr.22894).
36. TK, 2008–2009, 32011, nr.1.
37. Bijlage bij TK, 2008–2009, 32011, nr.2.

wettelijk rookverbod in de horeca niet langer uitsluitend baseren op het recht op een rookvrije werkplek (Tabakswet 11a lid 1), maar ook op de bescherming van bezoekers in de horeca tegen passief roken[38]:

> *"Uitgangspunt in de Tabakswet is steeds geweest dat in openbare ruimten kan worden gewerkt en verbleven zonder daarbij hinder of overlast van roken te ondervinden. Daarbij gaat het dus zowel om de bescherming van werknemers als om bescherming van bezoekers. Bij de rookvrije horeca speelde regelmatig de vraag op voor wie/welke kring van mensen de maatregelen moesten worden genomen. Horecaondernemers met personeel zijn immers verplicht primair hun personeel te beschermen, terwijl horecaondernemers zonder personeel primair het bezoekerspubliek beschermen. Ook dit pleit ervoor om het begrip «rookverbod» rechtstreeks in de Tabakswet op te nemen zodat duidelijk is dat in voor publiek toegankelijke gebouwen een rookverbod geldt. Kortom, een wijziging van de Tabakswet biedt mijns inziens ook voor de lange termijn de meeste rechtszekerheid, rechtsgelijkheid en duidelijkheid voor werkgevers, werknemers en bezoekers van de horeca."* (p.2-3)

In maart 2010, na de val van het kabinet Balkenende op 20 februari 2010, verklaart de Tweede Kamer het onderwerp 'rookvrije horeca' controversieel.[39]

In oktober 2010 breekt het regeerakkoord met het in 2009 door minister Klink voorgestelde nieuwe beleid om de doelstelling van het wettelijk rookverbod in de horeca uit te breiden met de bescherming van bezoekers in de horeca tegen passief roken. Het regeerakkoord stelt voor om kleine cafés zonder personeel van het wettelijk rookverbod in de horeca uit te zonderen[40]:

> *"Het huidige rookverbod wordt versoepeld. Het rookverbod werkt in de meeste horecagelegenheden goed. De horeca is voor het overgrote deel rookvrij geworden. Maar in veel kleine cafés, waar geen personeel in dienst is, is geen behoefte aan een rookverbod. Het kabinet is voornemens om horeca met minder dan 70 m2 bedrijfsruimte naar Duits voorbeeld vrij te stellen van het rookverbod."* (p.19)

In november 2010 schrijft minister Schippers van VWS een brief aan de Tweede Kamer dat zij uitvoering gaat geven aan de in het regeerakkoord overeengekomen uitzondering van kleine cafés zonder personeel van het wettelijk rookverbod in de horeca[41].

38. TK, 2008–2009, 32011, nr.2.
39. TK, 2009–2010, 32333, nr.1.
40. TK, 2010–2011, 32417, nr.5.
41. TK, 2010-2011, 22894, nr.289.

In december 2010 stuurt minister Schippers een nieuw 'Ontwerpbesluit, houdende verduidelijking van het besluit uitvoering rookvrije werkplek, horeca en andere ruimten' naar de Tweede Kamer. Tegelijkertijd trekt zij het ontwerpbesluit in dat minister Klink in september 2009 aan de Tweede Kamer heeft gestuurd. Het nieuwe ontwerpbesluit van minister Schippers zondert kleine cafés zonder personeel uit van het wettelijk rookverbod in de horeca en staat daarmee haaks op het eerdere ontwerpbesluit van minister Klink. In de nota van toelichting bij haar ontwerpbesluit schrijft minister Schippers[42]:

> *"Met behoud van de hoofdregel van de rookvrije werkplek voor werknemers in de horeca wordt aldus bewerkstelligd dat in (zeer) kleine buurtkroegjes, waar de kroeg een sociale functie vervult vergelijkbaar met die van de huiskamer in een huisgezin, roken wel mogelijk wordt gemaakt. Met het terugtreden van de regulering voor deze ruimten versterkt de wetgever de gedachte dat roken in de huiskamer niet een aangelegenheid is waar de overheid zich mee bemoeit. Het is een zaak voor de uitbater zelf, die zich daarbij ongetwijfeld mede zal laten leiden door datgene wat de klanten daarover te berde brengen."* (p.5)

Het bovenstaande argument van minister Schippers, dat de overheid zich niet moet bemoeien met roken in de privésfeer, komt overeen met een belangrijke overweging uit de memorie van toelichting bij het wetsvoorstel voor de Tabakswet uit ***november 1984*** (zie het begin van deze paragraaf)[43]:

> "*.. heeft de roker er recht op dat overheidsbemoeienis op dit punt zich niet uitstrekt tot zijn persoonlijke levenssfeer. Dit betekent dat de overheid zich heeft te onthouden van het stellen van regels ten aanzien van het roken in de privésfeer met het oogmerk de niet-roker te beschermen."* (p.12)

In januari 2011 stelt minister Schippers ter rechtvaardiging van de versoepeling van het rookverbod in de horeca die zij voorstelt, dat het uitgangspunt van dit rookverbod is de bescherming van werknemers (en dus niet van bezoekers) in de horeca tegen passief roken[44]:

> *"Minister Schippers: Voorzitter. Mijn beleid is heel helder en doet recht aan het uitgangspunt dat wij zelf hebben gekozen voor het rookverbod, namelijk de bescherming van werknemers tegen tabaksrook."* (p.20)

> *"Minister Schippers: Het uitgangspunt van de wet is het beschermen van de werknemers tegen tabaksrook. Waarom? Omdat sprake is van een hiërarchische relatie.*

42. Bijlage bij TK, 2010-2011, 32011 nr.9.
43. TK, 1984-1985, 18749, nr.3.
44. TK, 2010–2011, 32011, nr.15.

De werknemer kan wel denken «ik heb het eigenlijk liever niet», maar durft dat misschien niet te zeggen. Dan staat hij in de rook. Dat is dus het uitgangspunt.” (p.29)

10.4 Aanpassing van het model naar aanleiding van het voorbereidende literatuuronderzoek

Ter voorbereiding van de toetsing van versie 7 van het model aan de casus ‘wettelijk rookverbod in de horeca’ heb ik een literatuuronderzoek naar de totstandkoming van dit rookverbod gedaan (zie §10.2 en 10.3). Bij het screenen en doorlezen van alle documenten ben ik steeds bij mijzelf nagegaan of alle argumenten die ik las, m.b.t. de gerechtvaardigdheid van het wettelijk rookverbod, konden worden ondergebracht onder een of meer criteria van het beoordelingsmodel. Hiermee is de toetsing van versie 7 van het beoordelingsmodel aan de casus ‘wettelijk rookverbod in de horeca’ dus eigenlijk begonnen. Argumenten op de volgende gebieden kon ik niet goed onderbrengen onder de criteria van versie 7 van het beoordelingsmodel:
- handhaafbaarheid, en
- controleerbaarheid.

Hieronder zal ik toelichten hoe deze begrippen, in het kader van de beoordeling van de gerechtvaardigdheid van het wettelijk rookverbod in de horeca, dienen te worden geïnterpreteerd.

Handhaafbaarheid
In het kader van het ontwerp, de uitvoering en de evaluatie van het wettelijk rookverbod in de horeca is in de Tweede Kamer met grote regelmaat uitgebreid gedebatteerd over de handhaving van het wettelijk rookverbod in de horeca. Het ging hierbij om:
- de vormgeving van de handhaving (bestuurlijke versus strafrechtelijke handhaving, boete voor de roker en/of voor de eigenaar van de horecagelegenheid, hoogte van de boetes, straffen als boetes niet helpen, risicogerichte inspectie, ‘kliklijn’, e.d.),
- de resultaten van de handhaving (percentage horecagelegenheden waarin het rookverbod wordt nageleefd),
- de uitvoerbaarheid van de handhaving (benodigd aantal inspecteurs, te lange strafrechtelijke procedures).

In de uitgebreide en ingewikkelde discussies over de vormgeving van de handhaving zie ik aanleiding om hier een afzonderlijk criterium van te maken. Ter illustratie geef ik twee citaten[45]:

> *"Mevrouw Kant (SP): ... Hoe zit het boetesysteem in elkaar? Wordt alleen de caféhouder beboet of ook de persoon die ergens rookt waar het niet mag? Er moet een systeem komen waarbij het normatieve karakter vooropstaat en niet het straffen en beboeten. Dat zal echter niet eenvoudig zijn. Er moet geen rookpolitie komen."* (p.3)

> *"Antwoord van de minister [Klink]. … De cultuuromslag is vrij breed, zowel bij de ondernemers als bij het publiek. Het merendeel van de Nederlandse bevolking is voorstander van het rookvrij maken van de horeca. Dat is belangrijk voor de naleving. De sociale controle is ook vrij groot. De minister meent dat handhaving spontaan zal plaatsvinden."* (p.8).

Zonder voldoende draagvlak voor het wettelijk rookverbod onder de betrokken partijen (rokers en niet-rokers), is een wettelijk rookverbod niet te handhaven. Flankerend beleid kan bijdragen aan de (spontane) naleving van het rookverbod. Daarom volgt het criterium 'handhaafbaarheid' op de criteria 'draagvlak' en 'flankerend beleid'. Maatregelen in het kader van de 'handhaafbaarheid' van het rookverbod, kan men zien als een bijzondere vorm van 'flankerend beleid'. Daarom vormen de criteria 'flankerend beleid' en 'handhaafbaarheid' samen een beoordelingscluster.

Controleerbaarheid

Voordat de preventiemaatregel wordt geïmplementeerd, is vaak niet met zekerheid te voorspellen hoe de baten, lasten, handhaving en uitvoering van de preventiemaatregel in de praktijk zullen uitpakken. Als de baten, lasten, handhaving of uitvoering van de preventiemaatregel in de praktijk tegenvallen, moet kunnen worden bijgestuurd. Als bijvoorbeeld het beoogde effect uitblijft, is toepassing van de preventiemaatregel (in ongewijzigde vorm) niet langer gerechtvaardigd. Daarom is het noodzakelijk dat men bij implementatie van de preventiemaatregel voldoende zicht heeft op de ontwikkeling van de baten, lasten, handhaving en uitvoering. De Raad van de Europese Unie noemt in dit verband in een aanbeveling als beginsel voor de rookvrij-wetgeving[46]:

> *"De uitvoering van een rookvrij-wetgeving, haar handhaving en impact moeten gecontroleerd en beoordeeld worden."* (p.9)

45. TK, vergaderjaar 2006–2007, 30800 XVI, nr.182.
46. Publicatieblad van de Europese Unie. C 296, 5 december 2009, p.4-14.

Dit controleren en beoordelen kan door de baten, lasten, handhaving en uitvoering van de preventiemaatregel te monitoren, of door het (periodiek) uitvoeren van evaluatieonderzoek. Naarmate de implementatie van de preventiemaatregel met meer onzekerheden is omgeven en/of de lasten ten gevolge van implementatie groter zijn, kunnen hogere eisen worden gesteld aan de monitoring en/of evaluatie van de preventiemaatregel.

De criteria 'controleerbaarheid' en 'uitvoerbaarheid' vormen samen een beoordelingscluster. Ik kies er voor om het criterium 'controleerbaarheid' aan het criterium 'uitvoerbaarheid' vooraf te laten gaan. Een eerste argument hiervoor is dat de monitoring en/of evaluatie van de preventiemaatregel ook uitgevoerd moeten worden. Zijn de benodigde menskracht en middelen hiervoor beschikbaar? Een tweede argument hiervoor is dat ik het niet gerechtvaardigd acht om de preventiemaatregel uit te voeren, als de monitoring en/of evaluatie van de preventiemaatregel onvoldoende geregeld zijn. Een argument om het criterium 'uitvoerbaarheid' aan het criterium 'controleerbaarheid' vooraf te laten gaan, is dat ook gecontroleerd moet worden of de uitvoering van de preventiemaatregel volgens plan verloopt. De twee eerder gegeven argumenten om het criterium 'controleerbaarheid' aan het criterium 'uitvoerbaarheid' vooraf te laten gaan, vind ik echter zwaarder wegen.

Overzicht 17: vergelijking van versie 7 en 8 van het model

**Na toetsing aan casus 'Clarian':<br>versie 7 van het model**	**Toetsing aan casus 'horeca':<br>versie 8 van het model**
**Eerste zeef: logica van het ontwerp**	**Eerste zeef: logica van het ontwerp**
Cluster 1	***Cluster 1***
Schadelijkheid	Schadelijkheid
Noodzakelijkheid	Noodzakelijkheid
Cluster 2	***Cluster 2***
Oorzakelijkheid	Oorzakelijkheid
Verantwoordelijkheid	Verantwoordelijkheid
Cluster 3	***Cluster 3***
Gerichtheid	Gerichtheid
Geschiktheid	Geschiktheid
**Tweede zeef: effecten en neveneffecten**	**Tweede zeef: effecten en neveneffecten**
Cluster 4	***Cluster 4***
Effectiviteit	Effectiviteit
Indringendheid	Indringendheid

Cluster 5	***Cluster 5***
Lasten-batenverhouding	Lasten-batenverhouding
Rechtvaardigheid	Rechtvaardigheid
**Derde zeef: implementatie**	**Derde zeef: implementatie**
Cluster 6	***Cluster 6***
Timing	Timing
Draagvlak	Draagvlak
Cluster 7	***Cluster 7***
Flankerend beleid	Flankerend beleid
	Handhaafbaarheid
	Cluster 8
	Controleerbaarheid
Uitvoerbaarheid	Uitvoerbaarheid

Handhaafbaarheid en controleerbaarheid

Diverse politici achten handhaafbaarheid en controleerbaarheid van belang voor de rechtvaardiging van het wettelijk rookverbod in de horeca. Ik denk dat zij hierin gelijk hebben en voeg deze criteria daarom aan versie 7 van het model toe. Deze toevoeging leidt tot versie 8 van het model (zie overzicht 17).

Een mogelijke verklaring dat de nu toegevoegde criteria 'controleerbaarheid' en 'uitvoerbaarheid' een grote rol spelen bij de casus 'wettelijk rookverbod in de horeca' en minder bij de casus 'Clarian', is dat het wettelijk rookverbod in de horeca daadwerkelijk is geïmplementeerd, terwijl het preventieplan van Clarian door een gebrek aan draagvlak niet tot implementatie is gekomen. De toegevoegde criteria betreffen de 3e zeef 'implementatie' van het model.

In de hoofdstukken 11 t/m 13 wordt de gerechtvaardigdheid van het wettelijk rookverbod in de horeca getoetst aan de hand van de 16 criteria van versie 8 van het beoordelingsmodel. Voordat de beoordeling plaatsvindt, wordt van het betreffende criterium eerst een beknopte samenvatting van de 'operationalisering en wijze van beoordeling' van het betreffende criterium gegeven. Zie voor een uitgebreide beschrijving van de 'operationalisering en wijze van beoordeling' de bespreking van het betreffende criterium in de hoofdstukken 6, 7 en 8 (casus Clarian Health).

10.5 Onverwachte problemen bij toepassing van het model op casus 'wettelijk rookverbod in horeca'

Na afronding van de vorige paragraaf ben ik aan de slag gegaan met de toetsing van de gerechtvaardigdheid van het wettelijk rookverbod in de horeca aan versie 8 van het beoordelingsmodel (zie overzicht 17). De gedachte hierachter was dat ik de juistheid, volledigheid en praktische toepasbaarheid van het model het best zou kunnen onderzoeken door het model toe te passen op een daadwerkelijk geïmplementeerde en uitgebreid gedocumenteerde preventiemaatregel, zoals het wettelijk rookverbod in de horeca. Bij de toetsing van de gerechtvaardigdheid van het wettelijk rookverbod in de horeca aan versie 8 van het beoordelingsmodel, ben ik echter al bij het eerste criterium vastgelopen. De reden was, dat toepassing van het model niet goed mogelijk blijkt, als de doelstelling van de preventiemaatregel die wordt beoordeeld onduidelijk is. Bij nader inzien is dat ook logisch. Als de doelstelling onduidelijk of veranderlijk is, kan niet worden beoordeeld in hoeverre de doelstelling wordt behaald c.q. kan de effectiviteit van de preventiemaatregel niet worden beoordeeld.

Na afronding van de beschrijving van de totstandkoming van het wettelijk rookverbod in de horeca (zie §10.3) was voor mij nog steeds onduidelijk of, naast de bescherming van werknemers in de horeca tegen passief roken, ook de bescherming van bezoekers in de horeca tegen passief roken een doelstelling van het wettelijk rookverbod in de horeca was. Achtereenvolgende ministers van VWS hebben hierover namelijk een verschillend standpunt ingenomen. Minister Klink is hierover tijdens zijn bewindsperiode zelfs enkele keren van standpunt gewisseld. Hieronder geef ik op basis van §10.3 een samenvattend overzicht van de standpunten van verschillende ministers van VWS over de doelstelling van rookverboden in de horeca. Ik vermeld hierbij ook het standpunt van minister Hoogervorst, omdat het stappenplan ter zelfregulering van het rookbeleid in de horeca dat hij met KHN overeen is was gekomen, kan worden gezien als de aanloop naar het wettelijk rookverbod in de horeca dat minister Klink heeft ingevoerd.

Samenvattend overzicht van de wisselende standpunten van achtereenvolgende ministers van VWS over de doelstelling van het (wettelijk) rookverbod in de horeca:

- In oktober 2003 schrijft minister Hoogervorst aan de vaste commissie van de Tweede Kamer voor VWS dat de doelstelling van het stappenplan van KHN is een rookvrije horeca ter bescherming van <u>werknemers en bezoekers</u> in de horeca tegen passief roken.

- In juni 2007 (een jaar voor invoering van het wettelijk rookverbod in de horeca) stelt minister Klink in de Tweede Kamer dat de doelstelling (*"het oogmerk"*) van het wettelijk rookverbod in de horeca primair is de bescherming van werknemers in de horeca tegen passief roken en secundair de bescherming van bezoekers in de horeca tegen passief roken.
- In juli 2007 schrijft minister Klink aan de Tweede Kamer dat de bescherming van werknemers en bezoekers de reden is om per 1 juli 2008 een wettelijk rookverbod in de horeca in te voeren.
- In juni 2009 (een jaar na invoering van het wettelijk rookverbod in de horeca) stelt minister Klink in een spoeddebat in de Tweede Kamer dat alleen de bescherming van werknemers in de horeca de reden was om per 1 juli 2008 een wettelijk rookverbod in de horeca in te voeren en dat dit wat hem betreft zo blijft.
- In juli 2009 wil minister Klink het 'Besluit uitvoering rookvrije werkplek, horeca en andere ruimten' zodanig aanpassen, dat werknemers en bezoekers in de horeca tegen passief roken worden beschermd.
- In september 2009 stelt minister Klink dat het uitgangspunt van de Tabakswet steeds is geweest de bescherming van werknemers en bezoekers in openbare ruimten tegen passief roken. Hij wil dit uitgangspunt nu ook toepassen op de horeca.
- In januari 2011 stelt minister Schippers dat het uitgangspunt van de *"wet"* is het beschermen van werknemers tegen tabaksrook. De bescherming van bezoekers in de horeca tegen passief roken is volgens minister Schippers geen doelstelling van het wettelijk rookverbod in de horeca.

Voordat duidelijk is wat de doelstelling van het wettelijk rookverbod in de horeca is, kan ik de gerechtvaardigdheid van dit rookverbod niet toetsen aan de hand van het model. Daarom tracht ik in deze paragraaf (§10.5) eerst de doelstelling van het wettelijk rookverbod in de horeca duidelijk te krijgen. De hamvraag is hierbij of de bescherming van bezoekers in de horeca tegen passief roken ook tot de doelstelling van het wettelijk rookverbod in de horeca behoort.

Wat is het uitgangspunt van de Tabakswet?

Vanaf 1984 t/m juli 2007 (meer dan 20 jaar) zijn alle door de overheid bedongen rookverboden in voor het publiek toegankelijke, besloten ruimten (gebouwen, openbaar vervoer) gericht op de bescherming van werknemers en bezoekers tegen passief roken. Dat geldt voor:

- het wetvoorstel voor de Tabakswet van de staatssecretarissen Van der Reijden van WVC en Van Zeil van EZ uit 1984;
- de Tabakswet, zoals die in 1990 van kracht werd;

– het wetsvoorstel tot wijziging van de Tabakswet van minister Borst-Eilers uit 1999;
– de 'Wet van 18 april 2002 tot wijziging van de Tabakswet' die in april 2002 werd aangenomen;
– het beleid van minister Hoogervorst dat was gericht op het realiseren van een rookvrije horeca ter bescherming van werknemers en bezoekers;
– het Stappenplan 'Gewoon gastvrij' van KHN ter zelfregulering van het rookbeleid in de horeca, dat minister Hoogervorst met KHN overeen was gekomen[47];
– het besluit van minister Klink tot invoering van een rookvrije horeca, zoals hij dat in juli 2007 in een algemeen overleg in de Tweede Kamer heeft toegelicht.

In september 2009 stelt minister Klink dat het uitgangspunt van de Tabakswet steeds is geweest de bescherming van werknemers en bezoekers in openbare ruimten tegen passief roken. In januari 2011 stelt minister Schippers dat het uitgangspunt van de *"wet"* is het beschermen van de werknemers tegen tabaksrook.

Gezien de inhoud van de artikelen 10, 11 en 11a van de Tabakswet en de geschiedenis van de totstandkoming van de Tabakswet (zie §10.3) kom ik tot de volgende conclusie: het uitgangspunt van de Tabakswet is steeds geweest de bescherming van werknemers en bezoekers in openbare ruimten tegen passief roken. De eerste bewindspersoon die hiervan afweek was minister Klink toen hij in 2007, zonder zijn argumenten hiervoor publiekelijk kenbaar te maken, besloot om de doelstelling van het wettelijk rookverbod in de horeca te beperken tot de bescherming van werknemers tegen passief roken. Naar aanleiding van een aantal rechtszaken, waarin de rechtmatigheid van het rookverbod voor de horeca zonder personeel wordt aangevochten, stelt minister Klink in september 2009 voor om de doelstelling van het rookverbod in de horeca met personeel uit te breiden met de bescherming van bezoekers tegen passief roken. In februari 2010 valt het kabinet Balkenende en kan minister Klink dit voorstel niet meer ten uitvoer brengen. In december 2010 trekt minister Schippers dit voorstel van minister Klink in. Zij wil vasthouden aan het uitgangspunt dat de doelstelling van het rookverbod in de horeca zich beperkt tot de bescherming van werknemers tegen passief roken. Als minister Schippers in januari 2011 stelt dat het uitgangspunt van de *"wet"* is het beschermen van de werknemers tegen tabaksrook, neem ik aan dat zij hiermee het wettelijk rookverbod in de horeca bedoelt, en niet de gehele Tabakswet.

47. Overigens stelde KHN in een brief aan minister Hoogervorst d.d. 26 augustus 2004 dat de percentages in het stappenplan betrekking hadden op bedrijven met personeel (deze brief is een bijlage bij Kamerstuk 29200-XVI nr.278).

Wettelijk rookverbod in de horeca met personeel

Het wettelijk rookverbod in de horeca met personeel, dat per 1 juli 2008 door minister Klink is ingevoerd, is gebaseerd op artikel 11a lid 1 van de Tabakswet (het recht op een rookvrije werkplek), dat per 1 januari 2004 door minister Borst-Eilers is ingevoerd. De doelstelling van dit rookverbod is volgens minister Klink de bescherming van werknemers in de horeca tegen passief roken in de horeca (zie de uitspraak van minister Klink in de Tweede Kamer in juni 2009). Artikel 11a lid 1 van de Tabakswet stelt:

> *"Werkgevers zijn verplicht zodanige maatregelen te treffen dat werknemers in staat worden gesteld hun werkzaamheden te verrichten zonder daarbij hinder of overlast van roken door anderen te ondervinden."*

De aanduiding '*zonder daarbij hinder of overlast van roken door anderen te ondervinden*' biedt de ruimte om te zoeken naar gelijkwaardige alternatieven voor het rookverbod, bijvoorbeeld een ventilatiesysteem dat de lucht zuivert van tabaksrook, zodat werknemers geen hinder of overlast van de tabaksrook ondervinden.

Wettelijk rookverbod in de horeca zonder personeel

Het wettelijk rookverbod in de horeca zonder personeel, dat per 1 juli 2008 door minister Klink is ingevoerd, is gebaseerd op artikel 11a lid 4 van de Tabakswet, dat verwijst naar artikel 10 lid 1 van de Tabakswet, dat verwijst naar artikel 10 lid 2 van de Tabakswet. De doelstelling van dit rookverbod is volgens minister Klink het creëren van een gelijk (economisch) speelveld tussen de horeca met en zonder personeel (zie de nota van toelichting bij het Besluit uitvoering rookvrije werkplek, horeca en andere ruimten). Artikel 10 lid 2 van de Tabakswet stelt:

> *"Tot de maatregelen, bedoeld in het eerste lid, behoort in ieder geval het instellen, aanduiden en handhaven van een rookverbod in ruimten, behorende tot bij algemene maatregel van bestuur, aangewezen categorieën. Op het verbod kunnen, overeenkomstig bij de algemene maatregel van bestuur gestelde regelen, beperkingen worden aangebracht."*

De aanduiding '*instellen, aanduiden en handhaven van een rookverbod*' biedt niet de ruimte om te zoeken naar gelijkwaardige alternatieven voor het rookverbod.

Conclusies over de doelstelling van het wettelijk rookverbod in de horeca

- Eigenlijk kent de horeca twee verschillende wettelijke rookverboden:
  - een rookverbod voor de horeca met personeel;
  - een rookverbod voor de horeca zonder personeel;
- Het wettelijk rookverbod voor de horeca met personeel heeft een andere doelstelling (bescherming van werknemers tegen passief roken) dan het wettelijk

rookverbod voor de horeca zonder personeel (creëren van een gelijk speelveld tussen de horeca met en zonder personeel);
- Het wettelijk rookverbod voor de horeca met personeel is minder streng (gelijkwaardige alternatieven voor het rookverbod zijn toegestaan) dan het wettelijk rookverbod voor de horeca zonder personeel (gelijkwaardige alternatieven voor het rookverbod zijn niet toegestaan).

Ontbrekende argumentatie over beperkte doelstelling van wettelijk rookverbod in horeca

Tijdens de bestudering van het wettelijk rookverbod in de horeca heb ik lang abusievelijk in de veronderstelling verkeerd dat de doelstelling hiervan tweeledig was, namelijk de bescherming van werknemers en van bezoekers in de horeca tegen passief roken. Net als Tweede Kamerlid Joldersma van het CDA (de partij van minister Klink) dacht ik dat de manier waarop minister Klink juridisch vorm had gegeven aan het wettelijk rookverbod in de horeca niet aansloot op de beleidsmatige doelstellingen van het wettelijk rookverbod in de horeca. Ik verkeerde in de veronderstelling dat minister Klink aan de bescherming van niet-rokers c.q. werknemers en bezoekers in de horeca tegen passief roken juridisch vorm had gegeven door de horeca niet langer uit te zonderen van het recht op een rookvrije werkplek (artikel 11a lid 1 van de Tabakswet). Tweede Kamerlid Joldersma verwoordt dit in mei 2009 als volgt[48]:

> *"Mevrouw Joldersma (CDA): Voorzitter. Het CDA hecht zeer aan rookvrije horeca zonder uitzonderingen. We moeten tegelijkertijd constateren dat het realiseren van rookvrije horeca tot nu toe niet echt over rozen is gegaan. We hebben regelmatig met de minister van gedachten gewisseld en het is heel terecht dat we dat vanavond weer doen, na de forse aantijgingen in de uitspraak van het gerechtshof in Den Bosch. Het hof in Den Bosch lijkt te zeggen dat er geen juridische basis is voor een rookverbod in cafés zonder personeel. De uitspraak van de rechtbank in Groningen staat daar haaks op. Wij zijn dus zelf maar eens in al die uitspraken en in de voorgeschiedenis van de Tabakswet gedoken. Als we dit op ons laten inwerken, is onze voorlopige conclusie dat er eigenlijk met de wetgeving niet zo heel veel mis is. De uitsp[r]aak van het hof te Den Bosch is gebaseerd op artikel 11a, lid 1, van de Tabakswet. Dit artikel gaat over het realiseren van een rookvrije werkplek. De uitspraak van de rechtbank in Groningen is gebaseerd op hetzelfde artikel 11a, maar dan op lid 4. Lid 4 gaat over publieksruimten. … Het grote verschil tussen lid 1 en lid 4 van artikel 11a is dat het in lid 1 alleen gaat over werknemers. Op basis daarvan kan ik de redenering van het hof in Den Bosch volgen. Artikel 10 gaat ook over bezoekers*[49]. Op basis

48. TK, 2008-2009, vergaderingnr.84, p.6613-6629 (dossiernr.22894).
49. Artikel 11a, lid 4 van de Tabakswet verwijst naar artikel 10 van de Tabakswet.

> daarvan kan ik de redenering volgen van de rechtbank in Groningen, die zegt dat het niet uitmaakt of een café-eigenaar personeel heeft, omdat het in alle gevallen om een publieke ruimte gaat die rookvrij zou moeten zijn." *(p.6616-6617)*

> "*Kijkend naar de verschillende uitspraken is mijn conclusie dat het probleem niet in de wetgeving zit, maar in het uitvoeringsbesluit dat de minister heeft gemaakt. Dat uitvoeringsbesluit hinkt op zowel lid 1 als op lid 4, maar eigenlijk vooral op de rookvrije werkplek. Klopt het dat als je het uitvoeringsbesluit aanpast, er geen probleem meer is en er rookvrije horeca kan worden gecreëerd zonder enige uitzondering?*" (p.6617)

In hetzelfde debat ontkent minister Klink echter dat het wettelijk rookverbod in de horeca ook tot doel heeft bezoekers in de horeca tegen passief roken te beschermen:

> *"De grondslag en het aangrijpingspunt voor en het oogmerk van deze wet- en regelgeving is om de werknemer te beschermen, niet meer en niet minder. Ik verwees naar de spin-off van het geheel, dus naar de effecten. De effecten zijn dat ook de horecabezoeker er uiteindelijk baat bij heeft. Ik zeg niet dat dit de reden is geweest voor de wet- en regelgeving. Dat zal het wat mij betreft ook niet worden. Het is wel een bijeffect van de maatregelen die wij hebben genomen. Ik geef maar even aan dat het een gelukkig bijeffect is, want het gaat hier om een miljoen mensen die aan astma lijden in ons land en die daadwerkelijk veel hinder ondervinden van het roken in een besloten ruimte."* (p.6623)

Deze ontkennende reactie van minister Klink leidt tot verbazing en verwarring bij zijn partijgenoot in de Tweede Kamer (het wettelijk rookverbod in de horeca is dan al bijna een jaar van kracht!):

> *"Mevrouw Joldersma (CDA): Ik snap de uitspraak van de minister niet als hij zegt: wij hebben het hier over de rookvrije werkplek. In het uitvoeringsbesluit waar deze wijziging op gebaseerd is, wordt er verwezen naar wetsartikel 11a en naar lid vier en lid vijf. Lid vier gaat over wat ik hier net heb zitten betogen over de publieke ruimte. Dat verwijst terug naar artikel 10 en artikel 10 gaat over de publieke ruimte. Het gaat niet alleen om bescherming tegen rook van de werknemer, maar ook van de bezoeker. Dan snap ik niet waarom de minister nu alleen naar de werkplek verwijst en niet naar wat ik net heb proberen te zeggen over de publieke ruimte. Als de minister immers zo redeneert, gaat hij in wezen mee met de redenering van het hof. Daarmee laat hij eigenlijk zien dat hij vindt dat het hof gelijk heeft. Ik snap dat niet helemaal."* (p.6623)

De uitspraak van minister Klink is echter klip en klaar. Het wettelijk rookverbod in de horeca heeft tot doel de werknemers in de horeca tegen passief roken te beschermen, niet meer en niet minder. Dat betekent niet dat het overheidsbeleid niet meer ten doel heeft om bezoekers in de horeca tegen passief roken te bescher-

men, maar dat er blijkbaar onvoldoende politiek draagvlak was om de bescherming van bezoekers via het instrument van een wettelijk rookverbod te regelen. Of onvoldoende politiek draagvlak werkelijk de reden was om het wettelijk rookverbod te beperken tot enkel de bescherming van werknemers in de horeca tegen passief roken, kan ik uit de door mij bestudeerde parlementaire documenten niet opmaken.

Voor zover ik het kan beoordelen heeft minister Klink verzuimd om publiekelijk kenbaar te maken waarom hij de doelstelling van het wettelijk rookverbod in de horeca heeft beperkt tot de bescherming van werknemers in de horeca tegen passief roken. In §4.2 heb ik betoogd dat ***transparantie van de argumentatie*** in het kader van het beoordelingsmodel een algemene voorwaarde is voor de rechtvaardiging van een preventiemaatregel. De toepassing van de preventiemaatregel is niet gerechtvaardigd, als de onderbouwing niet publiekelijk kenbaar is gemaakt, omdat de juistheid en volledigheid van de argumentatie dan niet publiekelijk beoordeeld kunnen worden. Daniels (2000, p.1301) stelt in dit verband:

> *"A fair process requires publicity about the reasons and rationales that play a part in decisions. There must be no secrets where justice is involved, for people should not be expected to accept decisions that affect their well being unless they are aware of the grounds for those decisions."*

Voor de beoordeling van de gerechtvaardigdheid van het wettelijk rookverbod in de horeca zijn twee vragen relevant:

1. Is het gerechtvaardigd dat de doelstelling van het wettelijk rookverbod in de horeca is beperkt tot de bescherming van werknemers tegen passief roken?
2. Is het wettelijk rookverbod in de horeca gerechtvaardigd, indien de doelstelling van bescherming van werknemers tegen passief roken als uitgangspunt wordt genomen?

In het kader van mijn proefschrift heeft de beoordeling van de gerechtvaardigdheid van het wettelijk rookverbod in de horeca tot doel om de juistheid, volledigheid en praktische toepasbaarheid van het model te toetsen aan de praktijk. Alleen het wettelijk rookverbod zoals aangeduid in de tweede vraag, is in de praktijk geïmplementeerd, zodat het model alleen aan de hand van de tweede vraag aan de praktijk getoetst kan worden. Daarom richt ik de toetsing van het model aan de casus 'rookverbod in de horeca' op de tweede vraag: *'Is het wettelijk rookverbod in de horeca gerechtvaardigd, indien de doelstelling van bescherming van werknemers tegen passief roken als uitgangspunt wordt genomen?'*.

Voordat ik overga tot de toetsing van het beoordelingsmodel aan de casus 'wettelijk rookverbod in de horeca', zal ik in de volgende paragraaf (10.6) eerst ingaan op de eerste vraag: *'Is het gerechtvaardigd dat de doelstelling van het wettelijk rookverbod in de horeca is beperkt tot de bescherming van werknemers tegen passief roken?'.* Als deze doelstelling niet te rechtvaardigen is, kan ik wellicht beter op zoek gaan naar een andere casus om het model aan te toetsen. Het is immers zinloos om de gerechtvaardigdheid van alle aspecten van een preventiemaatregel te beoordelen, als de doelstelling van de preventiemaatregel niet te rechtvaardigen is.

10.6 Beoordeling van de gerechtvaardigdheid van de doelstelling van het wettelijk rookverbod in de horeca

10.6.1 Inleiding

In paragraaf 10.5 heb ik geconcludeerd dat het uitgangspunt van de Tabakswet is de bescherming van werknemers en bezoekers in openbare ruimten tegen passief roken. Na afronding van het voorbereidende literatuuronderzoek (zie §10.2) vraag ik mij af of de doelstelling van het wettelijk rookverbod in de horeca niet nog breder zou moeten zijn dan dit uitgangspunt van de Tabakswet, namelijk de bescherming van niet-rokers in de horeca tegen passief roken. Niet-rokers in de horeca zijn:

1. werknemers in de horeca;
2. bezoekers in de horeca;
3. bedrijfseigenaars in de horeca.

De gerechtvaardigdheid van het wettelijk rookverbod in de horeca ter bescherming van (1) werknemers tegen passief roken, komt uitgebreid aan bod bij de toetsing van de casus 'wettelijk rookverbod in de horeca' aan versie 8 van het beoordelingsmodel (zie de hoofdstukken 13 t/m 15). In deze paragraaf (§10.6) tracht ik antwoord te geven op de vraag of het wettelijk rookverbod in de horeca zich niet ook op de bescherming van (2) bezoekers en (3) bedrijfseigenaars zou moeten richten.

10.6.2 Bescherming van bezoekers in de horeca tegen passief roken

Schadelijkheid van passief roken voor bezoekers

Het Groenboek 'Op weg naar een rookvrij Europa' van de Europese Commissie uit januari 2007 stelt, mede op basis van het rapport van de Surgeon General uit 2006 (USDHHS)[50]:

> *"De dosis-effectrelatie voor hartziekten is niet-lineair. Iemand die passief rookt heeft maar liefst bijna half zoveel risico een hartziekte te krijgen als iemand die twintig sigaretten per dag rookt. Ook heel kleine doses tabaksrook kunnen een onmiddellijk effect hebben op het ontstaan van stremmingen in de bloedsomloop en trombose, alsmede een langetermijneffect op de ontwikkeling van arteriosclerosis – allemaal belangrijke factoren bij hartziekten. … Er is geen veilig niveau van blootstelling aan omgevingstabaksrook geconstateerd, en naar verwachting zal verder onderzoek een dergelijk niveau ook niet weten te identificeren."*

Dit kan worden opgevat als het (indirecte) bewijs dat passief roken schadelijk is voor bezoekers in de Horeca die incidenteel aan passief roken worden blootgesteld. Daarom was er, toen minister Klink in juni 2007 een brief aan de Tweede Kamer schreef over zijn besluit tot de invoering van een rookverbod in Horeca, voldoende wetenschappelijk bewijs dat passief roken een substantieel risico oplevert op gezondheidsschade voor bezoekers in de Horeca.

In mei 2007 onderschrijft de Nederlandse regering de juistheid en volledigheid van de cijfers in het Groenboek van de Europese Commissie[51]:

> *"Het Nederlandse kabinet constateert dat de Europese Commissie met het door haar uitgebrachte Groenboek de onderhavige problematiek in al zijn facetten adequaat, correct en volledig beschrijft. De Commissie vestigt langs deze weg nog eens de aandacht op het belang van een rookvrij publiek domein (voor zover het besloten ruimten betreft)." (p.1)*

De afgelopen jaren is de wetenschappelijke evidence over de schadelijkheid van passief roken voor bezoekers in de Horeca alleen maar sterker geworden. Het rapport van de Surgeon General uit 2010 over de gezondheidsrisico's van roken stelt (USDHHS, executive summary, 2010)[52]:

50. Het rapport van de Surgeon General uit 2006 is een zeer uitgebreide reviewstudie. Het rapport telt ruim 2000 literatuurverwijzingen.
51. Reactie van Nederland op de vragen van de Europese Commissie in het Groenboek 'Op weg naar een rookvrij Europa. Brief van Minister Klink aan de Eerste Kamer d.d. 15 mei 2007.
52. Het rapport van de Surgeon General uit 2010 is een zeer uitgebreide reviewstudie. Het rapport telt ruim 4500 literatuurverwijzingen.

"In 2006, the Surgeon General reported that the scientific evidence was sufficient to conclude that there is no risk-free level of exposure to secondhand smoke (U.S. Department of Health and Human Services 2006). This report provides a more detailed review of the mechanisms that validate that conclusion. A risk-free level of exposure to tobacco smoke (whether mainstream or from secondhand smoke) cannot be defined." (p.2)

Aantasting van de keuzevrijheid van bezoekers

In mei 2000 onderschrijft minister Borst-Eilers de stelling dat iedere burger aan alle activiteiten die tot het normale maatschappelijk leven behoren, moet kunnen deelnemen zonder de kans te lopen rook van een ander in te ademen[53]:

"De leden van de fractie van de PvdA halen de stelling van het Astma Fonds aan dat iedere burger aan alle activiteiten, die tot het normale maatschappelijk leven behoren, moet kunnen deelnemen zonder de kans te lopen rook van een ander in te ademen. Zij horen graag of en in hoeverre de regering ook deze stelling huldigt en zo ja, welke consequenties daaraan worden verbonden wat betreft de instelling van rookverboden. De regering onderschrijft uiteraard deze stelling. Dit uitgangspunt vormt namelijk sinds medio jaren tachtig een van de pijlers van het tabaksontmoedigingsbeleid. De bescherming van niet-rokers is ook een hoofddoelstelling van de Tabakswet. De onvoldoende maatschappelijke acceptatie van het recht op schone lucht voor niet-rokers en de in te geringe mate aanwezige tolerantie bij rokers blijkt in de dagelijkse praktijk niet voldoende ruimte te bieden voor een daadwerkelijk volwaardige bescherming van de meerderheid van niet-rokers in ons land. Daarom is het onvermijdelijk om wettelijke regels te gaan stellen voor die plaatsen waar een ieder vrij moeten kunnen komen of vertoeven zonder hinder van andermans rook. … De regering geeft inhoud aan voornoemd uitgangspunt door geleidelijk aan steeds meer plaatsen, ruimten en tijdstippen aan te wijzen waar niet meer gerookt kan worden. … Tegelijkertijd moeten we met z'n allen ook realistisch blijven. In absolute zin kan de stelling van het Astma Fonds nu eenmaal niet direct letterlijk worden waargemaakt. De daaraan verbonden consequentie zou namelijk zijn dat er bijna overal een rookverbod zou moeten komen, of anders gesteld: dat een roker nooit meer kan roken als er een niet-roker in de buurt is. Dit is als streven een prima doel, dat ik ook van harte onderschrijf, maar het is natuurlijk niet van de ene op de andere dag haalbaar noch te verwezenlijken." (p.35)

Op basis van het bovenstaande citaat kan worden geconcludeerd dat volgens minister Borst-Eilers iedere Horecagelegenheid in principe voor iedere burger probleemloos toegankelijk zou moeten zijn. Hierbij ga ik ervan uit dat gebruik maken van de Horeca behoort tot de activiteiten *"die tot het normale maatschappelijke*

53. TK, 1999–2000, 26472, nr.6.

leven behoren". Onder probleemloos versta ik dat niet-rokers geen risico lopen om aan passief roken te worden blootgesteld.

In december 2003 kiest Koninklijke Horeca Nederland (KHN) keuzevrijheid voor bezoekers als uitgangspunt voor haar stappenplan ter zelfregulering van het rookbeleid in de Horeca (KHN, 2003):

> *"Koninklijk Horeca Nederland heeft met minister Hoogervorst afgesproken dat dit stappenplan concrete doelstellingen zou bevatten die in overeenstemming zijn met het maatschappelijk draagvlak voor niet-roken in de horeca. Maatschappelijk draagvlak kan bij uitstek gemeten worden via marktwerking. Maar om deze marktwerking goed op gang te laten komen, is het noodzakelijk dat consumenten wel een duidelijke keuze hebben tussen horecagelegenheden met en zonder rookbeleid."(p.14)*

In mijn ogen is het vreemd om de toegankelijkheid van openbare voorzieningen (voor iedereen toegankelijke voorzieningen), i.c. de Horeca, voor mensen met een ziekte of handicap afhankelijk te maken van marktwerking. Dat lijkt een keuze voor het recht van de sterkste, i.c. de mensen zonder ziekte of handicap. Vermoedelijk interpreteert minister Hoogervorst de keuzevrijheid waarvoor KHN pleit als een tussenstap op weg naar een rookvrije Horeca. In oktober 2003 schreef hij immers in een brief aan het bestuur van KHN dat een rookvrije horeca op termijn onvermijdelijk is:

> *"Wij kwamen gezamenlijk tot de conclusie dat een rookvrije horeca op termijn onvermijdelijk is. … Gezien de gezondheidsrisico's bij blootstelling aan tabaksrook zal het stappenplan maatregelen moeten bevatten die daadwerkelijk bijdragen aan het beschermen van de gezondheid van werknemers en gasten in de horeca."*

In juli 2007 vindt een algemeen overleg plaats in de Tweede Kamer over het besluit van Minister Klink om per 1 juli 2008 een algeheel wettelijk rookverbod in de horeca in te voeren. Tweede Kamerlid Agema is tegen een algemeen rookverbod en pleit voor keuzevrijheid voor bezoekers[54]:

> *"Mevrouw Agema (PVV) is tegen een algemeen rookverbod in de horeca, ondanks dat algemeen bekend is dat roken slecht is voor de gezondheid. … Er zijn inmiddels voldoende rookvrije horecabedrijven. De mensen hebben een keuze. (p.2)*

Na juli 2007 kiest minister Klink ervoor, zonder dit publiekelijk met argumenten te onderbouwen, om de doelstelling van het wettelijk rookverbod in de horeca te beperken tot de bescherming van werknemers tegen passief roken.

54. TK, 2006-2007, 30800 XVI, nr.182.

In 2010 lijkt minister Schippers het uitgangspunt van de Tabakswet van een probleemloze toegankelijkheid voor iedereen van voor het publiek toegankelijke voorzieningen gedeeltelijk uit het oog te hebben verloren. Zonder dat onderzoek is uitgevoerd naar het belang dat mensen, met en zonder een aandoening die slecht reageert op tabaksrook, hechten aan rookvrije buurtkroegjes, stelt zij[55]:

> *"… Het huidige rookverbod wordt versoepeld. … in veel kleine cafés, waar geen personeel in dienst is, is geen behoefte aan een rookverbod. … Met deze uitzondering kom ik tegemoet aan de sociale functie van deze buurtkroegjes (of huiskamerkroegjes) waar een substantieel deel van de bezoekers net als in zijn eigen huiskamer wil kunnen roken, … Door de uitzondering wordt recht gedaan aan de keuzevrijheid van de consument,…)"* (p.1)

Een onderzoek naar het belang dat mensen hechten aan rookvrije buurtkroegjes moet natuurlijk niet alleen worden uitgevoerd onder de bezoekers van deze buurtkroegjes, maar ook onder de 'thuiszitters', die vanwege hun gezondheid het buurtkroegje niet of beperkt bezoeken. Mensen (met een aandoening die slecht reageert op tabaksrook) zullen een afweging maken tussen hun behoefte aan sociale contacten, waarin wordt voorzien door het bezoek aan een buurtkroegje, en hun gezondheid. Maar is het wel terecht dat zij een dergelijke afweging moeten maken? Uit het onderstaande citaat blijkt dat sommige bezoekers van zogenaamde *"huiskamerkroegjes"* de versoepeling van het rookverbod voor kleine cafés zonder personeel ervaren als vrijheidsbeperkend[56]:

> *"Mevrouw Bouwmeester (PvdA): … Hebben mensen nu hun buurtkroeg terug? Nee, die hebben ze niet terug. Ik kreeg verscheidene mails, bijvoorbeeld van een vrouw die dart en van een man die biljart. Zij schreven mij dat zij konden darten of biljarten omdat dat gebeurde in een rookvrije buurtkroeg. Nu er weer wordt gerookt, kunnen deze mensen hun lidmaatschap opzeggen. Zij schrijven: een ander heeft zijn vrijheid terug om overlast te veroorzaken, en ik kan mijn eigen kroeg niet meer in. Vrijheid? Het is maar hoe je dat begrip interpreteert."* (p.3)

Ter onderbouwing van het rookbeleid in de Horeca heeft in opdracht van de overheid veel onderzoek plaatsgevonden (zie §10.2). Onderzoek naar het belang dat mensen hechten aan een rookvrije Horeca lijkt echter te ontbreken. De overheid heeft niet laten onderzoeken welk belang mensen hechten aan de toegankelijkheid voor iedereen van iedere Horecagelegenheid; dus ook voor mensen met een aandoening van de luchtwegen. Een dergelijk onderzoek dient niet alleen plaats te vinden onder de bezoekers van een niet-rookvrije Horeca, maar juist ook onder de

55. TK, 2010-2011, 22894, nr.289.
56. TK, 2010-2011, 32011, nr.15.

bezoekers die een niet-rookvrije Horeca mijden. De overheid heeft ook niet laten onderzoeken welk belang mensen hechten aan keuzevrijheid tussen rookvrije en niet-rookvrije Horecagelegenheden. Met hoeveel keuzevrijheid nemen mensen genoegen? Ook dit onderzoek dient niet alleen plaats te vinden onder de bezoekers van niet-rookvrije Horeca, maar juist ook onder de mensen die bezoek aan niet-rookvrije Horeca mijden.

Analyse en conclusie

Passief roken is schadelijk voor de gezondheid van bezoekers in de horeca. Daarom is volgens mij de hamvraag of het voldoende is als burgers kunnen kiezen tussen rookvrije en niet-rookvrije horeca, of dat alle horeca voor iedere burger probleemloos toegankelijk dienen te zijn. Met probleemloos bedoel ik in dit geval dat bezoekers niet het risico lopen te worden blootgesteld aan passief roken.

Wanneer is er sprake van (ernstige) aantasting van de vrijheid van burgers?

- Is er aantasting van de vrijheid van burgers als zij niet rookvrij gebruik kunnen maken van iedere Horecagelegenheid?
- Is er pas aantasting van de vrijheid van burgers als zij er niet voor kunnen kiezen om van een rookvrije Horecagelegenheid gebruik te maken?
- Is er helemaal geen sprake van aantasting van de vrijheid van burgers als zij niet rookvrij gebruik kunnen maken van de Horeca, omdat men zou kunnen stellen dat het gebruik maken van cafés en restaurants geen basisbelang vertegenwoordigt?

Voor de gedachtevorming wil ik een vergelijking maken met de rolstoeltoegankelijkheid van de Horeca. Ik vermoed dat het uitgangspunt dat ieder restaurant en café in principe rolstoeltoegankelijk moet zijn, op breed draagvlak onder de Nederlandse bevolking kan rekenen. Zou analoog daaraan niet ieder restaurant en café probleemloos toegankelijk moeten zijn voor alle mensen die met het oog op hun gezondheid niet aan passief roken willen worden blootgesteld, waaronder ook longpatiënten, hartpatiënten, (ex-)kankerpatiënten, enz.?

In het kader van het politieke debat over het rookverbod in de Horeca is de discussie over het belang van een probleemloze toegankelijkheid van openbare voorzieningen voor iedereen, onvoldoende gevoerd. Gezien de voorgeschiedenis van de totstandkoming van het rookverbod in de horeca en het risico gezondheidsschade voor bezoekers in de horeca ten gevolge van passief roken, was het logisch geweest als minister Klink het wettelijk rookverbod in de horeca ook had gericht op de bescherming van bezoekers tegen passief roken. Uiteraard kunnen er ‘goede’ argumenten zijn om de doelstelling van het rookverbod in de horeca te beperken tot de

bescherming van werknemers tegen passief roken. Bijvoorbeeld het willen bieden van keuzevrijheid aan bezoekers tussen rookvrije en niet-rookvrije horeca, of het ontbreken van politiek of maatschappelijk draagvlak voor een rookverbod ter bescherming van bezoekers in de horeca. In een democratie dient men zulke argumenten wel te expliciteren, zodat ze getoetst kunnen worden. De overheid (de ministers Klink en Schippers) heeft haar keuze om bezoekers in de horeca niet wettelijk tegen passief roken te beschermen, onvoldoende beargumenteerd.

10.6.3 Bescherming van bedrijfseigenaars in de horeca tegen passief roken

Met uitzondering van het rookverbod in de horeca kennen de rookverboden op basis van de Tabakswet[57] in voor het publiek toegankelijke, besloten ruimten, een tweeledige doelstelling, namelijk de bescherming van werknemers én van bezoekers tegen passief roken. Rookverboden op basis van de Tabakswet bieden geen bescherming tegen passief roken aan bedrijfseigenaars die in hun eigen zaak werken. Niet-rokende bedrijfseigenaars kunnen schade of overlast ondervinden ten gevolge van passief roken, doordat hun werknemers of klanten roken. Blijkbaar is de wetgever ervan uitgegaan dat bedrijfseigenaars over de vrijheid beschikken om zelf een rookverbod voor hun eigen bedrijf in te stellen, als zij daar behoefte aan hebben. Meestal zal deze redenering opgaan, maar voor commerciële bedrijven met een publieksfunctie, zoals de horeca, is een kritische kanttekening op zijn plaats.

Tweede Kamerleden die tegenstander zijn van het rookverbod in kleine cafés zonder personeel, hebben betoogd dat deze cafés failliet kunnen gaan, als er niet mag worden gerookt. Ter illustratie enkele schriftelijke vragen van Tweede Kamerlid Verdonk aan minister Klink[58]:

> *"Bent u bekend met de situatie van vele kleine cafébedrijven die niet in staat zijn zich aan te passen aan de nieuwe situatie die ontstaan is door het rookverbod en failliet dreigen te gaan? Bent u bereid hier maatregelen tegen te nemen?"*
>
> *"Deelt u de opvatting dat een café-eigenaar zelf een keus kan maken om rookvrij of niet rookvrij te zijn?"*

Als café-eigenaars failliet kunnen gaan als in hun zaak een rookverbod geldt, zoals mevrouw Verdonk stelt, is het de vraag of zij wel de vrijheid hebben om op eigen gezag een rookverbod in te stellen of dat zij zich economisch gedwongen voelen

57. Er kan bijvoorbeeld ook een rookverbod gelden in verband met de brandveiligheid.
58. TK. Kamervragen. Aanhangsel 2008-2009, 1867. 20 maart 2009.

om hun eigen gezondheid op te offeren ten behoeve van het bedrijfsbelang. Ook kan ik mij voorstellen dat juist in het geval van zogenaamde 'huiskamerkroegjes' klanten het de eigenaar zeer kwalijk nemen als hij een rookverbod instelt. De sociale druk om dat niet te doen en dus de eigen gezondheid op te offeren, zou zeer groot kunnen zijn. Ik acht het een tekortkoming van het politieke debat over het rookverbod in de horeca, dat aan dit argument vooralsnog helemaal geen aandacht is besteed. In mijn visie kan het nodig zijn dat bedrijfseigenaars van gebouwen met een publieksfunctie wettelijk worden beschermd tegen passief roken door hun klanten.

Voordat men beslist de rookverboden in de Tabakswet te baseren op het uitgangspunt dat bedrijfseigenaars vrij zijn om in hun eigen bedrijf zelf een rookverbod in te stellen, zou men eerst wetenschappelijk moeten onderzoeken of dat in de praktijk wel het geval is. Naar mijn informatie heeft een dergelijk onderzoek nog niet plaatsgevonden. In theorie is dit een tekortkoming van alle rookverboden op basis van de Tabakswet die betrekking hebben op voor het publiek toegankelijke, besloten ruimten in het bedrijfsleven. In de praktijk is dit vraagstuk alleen actueel in kleine cafés zonder personeel.

10.6.4 Eindoordeel over de gerechtvaardigdheid van de doelstelling van het wettelijk rookverbod in de horeca

Er zijn goede argumenten om het wettelijk rookverbod in de horeca zowel op de bescherming van werknemers als bezoekers te richten en misschien zelfs op de bescherming van bedrijfseigenaars. De doelstelling van het wettelijk rookverbod in de horeca is achtereenvolgens door de ministers Klink en Schippers echter beperkt tot de bescherming van werknemers. Hiervoor kunnen goede argumenten zijn, maar deze dienen in een democratie wel te worden geëxpliciteerd, zodat ze kunnen worden getoetst. Dat is onvoldoende gebeurd.

Toch zie ik in de ontbrekende argumentatie over de beperkte doelstelling van het wettelijk rookverbod in de horeca (alleen de bescherming van werknemers tegen passief roken) onvoldoende reden om deze doelstelling, en daarmee het wettelijk rookverbod in de horeca, ongerechtvaardigd te noemen. Ik heb hiervoor de volgende argumenten:

- de ontbrekende argumentatie over de beperkte doelstelling van de preventiemaatregel betreft niet de wettelijke bescherming die wordt toegepast (de bescherming van werknemers in de horeca tegen passief roken), maar de wettelijke bescherming die niet wordt toegepast (de bescherming van bezoekers in de horeca tegen passief roken);

- werknemers in de horeca lopen meestal een (nog) groter risico op schade en overlast ten gevolge van passief roken dan bezoekers, omdat werknemers veelal langduriger en frequenter in de horeca aanwezig zijn dan bezoekers;
- als in een horecagelegenheid wordt gerookt, is het voor werknemers moeilijker om van baan te veranderen dan voor bezoekers om naar een andere horecagelegenheid te gaan;
- de wettelijke bescherming van werknemers in de horeca tegen passief roken vormt in principe geen belemmering om in de toekomst desgewenst ook bezoekers in de horeca tegen passief roken te beschermen.

In de hoofdstukken 11 t/m 14 wordt de gerechtvaardigdheid van het wettelijk rookverbod in de horeca beoordeeld aan de hand van versie 8 van het beoordelingsmodel (zie overzicht 17 in §10.4). Uitgangspunt bij deze beoordeling is dat de doelstelling van het wettelijk rookverbod in de horeca zich beperkt tot de bescherming van werknemers in de horeca tegen passief roken. In het kader van deze beoordeling wordt de bescherming van bezoekers in de horeca tegen passief roken niet gezien als een doelstelling c.q. beoogd effect, maar als een positief neveneffect van het wettelijk rookverbod in de horeca.

11 Toetsing 1e zeef van het beoordelingsmodel aan casus ‘wettelijk rookverbod in horeca’ (versie 8 van het model)

In dit hoofdstuk toets ik de gerechtvaardigdheid van het wettelijk rookverbod in de horeca aan de hand van de criteria van de 1e zeef van het model (versie 8).

11.1 Eerste criterium: schadelijkheid

11.1.1 Operationalisering en wijze van beoordeling

Het criterium ‘schadelijkheid’ betreft de aard en omvang van de risico’s op:
- schade:
  - gezondheidsschade;
  - sociaaleconomische schade;
  - inbreuk op het persoonlijk leven;
- overlast;
- schending van morele opvattingen.

Het gaat hierbij om de risico’s die men met de preventiemaatregel tracht te bestrijden. Ook gaat het om de vraag of men met de leefstijl zichzelf en/of derden benadeelt.

Beoordeeld wordt of de beschikbare informatie een duidelijk beeld geeft van de bovengenoemde risico’s. Als de beschikbare informatie geen duidelijk beeld geeft, wordt beoordeeld of de informatieverzameling over de risico’s voldoende is geweest. Heeft men voldoende gebruik gemaakt van de mogelijkheden om de risico’s te inventariseren en kwantificeren?

11.1.2 Beoordeling van casus ‘wettelijk rookverbod in horeca’ aan de hand van criterium ‘schadelijkheid’

Het wettelijk rookverbod heeft tot doel werknemers in de horeca te beschermen tegen de blootstelling aan tabaksrook (zie §10.5 en 10.6.4), niet meer en niet minder. De schade en overlast die werknemers kunnen ondervinden ten gevolge van

passief roken in de horeca vormt daarom het kader voor de beoordeling van de preventiemaatregel aan de hand van het criterium 'schadelijkheid'.

Gezondheidsschade (criterium schadelijkheid)

In 1995 verschijnt de kabinetsnota 'Gezond en wel'[1]. In deze nota stelt het kabinet (minister Borst-Eilers is minister van VWS):

> *"Roken is zeer schadelijk voor de gezondheid. Het veroorzaakt vooral longkanker en hart- en vaatziekten. … Daarnaast ontstaat er ook hinder en gezondheidsschade bij degenen in de directe omgeving van de roker. … Daarom wil het kabinet: … het rookverbod in openbare ruimten aanscherpen, waarbij sancties worden overwogen …" (p.23)*

In april 1999 stuurt minister Borst-Eilers een wetsvoorstel tot wijziging van de Tabakswet aan de Tweede Kamer. Dit voorstel bevat ondermeer een uitbreiding van de wettelijke bescherming van niet-rokers tegen passief roken. Bij het wetsvoorstel behoort een memorie van toelichting, waarin de minister de gronden waarop het wijzigingsvoorstel rust, beschrijft[2]:

> *"Het roken is niet alleen schadelijk voor de gezondheid van de roker. Ook niet-rokers kunnen gezondheidsschade ondervinden door tabaksrook van anderen, zo blijkt uit wetenschappelijke bevindingen. Dit geldt met name voor de werkplek waar rokers en niet-rokers gedwongen zijn om door de week vrijwel dagelijks voor langere tijd met elkaar samen te zijn. Daarnaast veroorzaakt tabaksrook hinder en overlast voor niet-rokers, vooral wanneer zij lijden aan aandoeningen van de ademhalingswegen, zoals astma, of als het om kinderen gaat." (p.1)*

> *"Het regelmatig blootstaan aan tabaksrook heeft een nadelige invloed op de gezondheid en het welbevinden, in het bijzonder voor de meest kwetsbare groepen, zoals CARA-patiënten, zwangere vrouwen en kinderen. Tabaksrook vormt op de arbeidsplaats, in openbare gelegenheden en thuis een zeer groot, zo niet het grootste binnenmilieuprobleem. In tabaksrook zitten ruim 50 kankerverwekkende bestanddelen. Voor deze stoffen bestaan, in tegenstelling tot andere schadelijke stoffen, geen drempelwaarden, waaronder ze geen gevaar met zich zouden brengen en niet meer schadelijk (onder meer kankerverwekkend) voor de gezondheid zouden zijn. Passief roken kan nauwelijks vermeden worden en elke mate van blootstelling aan tabaksrook geeft een verhoogd risico op onder meer longkanker."* (p.7)

1. TK, 1994-1995, 24126, nrs.1-2.
2. TK, 1998-1999, 26472, nr.3.

Van de bovenstaande citaten van minister Borst-Eilers kan worden afgeleid dat zij van opvatting is dat passief roken leidt tot gezondheidsschade bij werknemers op de werkplek, omdat zij hieraan dagelijks voor langere tijd zijn blootgesteld.

In april 2000 wordt de schadelijkheid voor de gezondheid van passief roken op de werkplek door de rechter in Breda bevestigd. Deze rechterlijke uitspraak staat nog steeds[3]:

> *"In dit verband heeft de rechter te Breda op 25 april 2000 uitgesproken dat niet-rokers ten principale recht hebben op een rookvrije werkplek. Het recht op bescherming van de lichamelijke integriteit, de gezondheid, is een grondrecht. Als uitgangspunt voor deze rechterlijke uitspraak gold dat er voor een kankerverwekkende stof als tabaksrook geen veilige ondergrens qua blootstelling is te bepalen. Daarom is een algeheel rookverbod in kantoorgebouwen en andere besloten werkruimten in het algemeen aangewezen, aldus dit vonnis. …" (p.34)*

In mei 2003 wordt het 'WHO-Kaderverdrag inzake tabaksontmoediging' vastgesteld. Het is in 2005 door Nederland geratificeerd en voor Nederland in werking getreden. Dit verdrag stelt in de preambule:

> *" … dat wetenschappelijk bewijs onomstotelijk heeft aangetoond dat tabaksconsumptie en blootstelling aan tabaksrook dood, ziekte en arbeidsongeschiktheid veroorzaken, en dat er geruime tijd ligt tussen de blootstelling aan roken en andere gebruikswijzen van tabaksproducten en het begin van tabaksgerelateerde ziekten, …."*

In september 2003 schrijft Minister Hoogervorst van VWS in een brief over het 'zelfreguleringstraject in het kader van een rookvrije horeca' aan de Tweede Kamer[4]:

> *"Tabaksrook is schadelijk voor de gezondheid. Niet alleen voor de gezondheid van de roker zelf, maar het staat ook onomstotelijk vast dat het inademen van andermans tabaksrook (meeroken) tot gezondheidsschade leidt. Bescherming van de niet-roker is dan ook een van de pijlers van het tabaksontmoedigingsbeleid. … Werkgevers in de horecasector zijn voorlopig vrijgesteld van de verplichting om hun werknemers een rookvrije werkplek te garanderen. Een betere bescherming van werknemers in de horeca tegen tabaksrook is echter wel gewenst."* (p.1)

In oktober 2003 schrijft minister Hoogervorst in een brief aan het bestuur van Koninklijke Horeca Nederland[5]:

3. TK, 1999-2000, 26472, nr.6.
4. TK, 2003-2004, 29200 XVI, nr.278.
5. POG/GB 2.411.992. Afspraken bestuurlijk overleg 8 september 2003. Den Haag, 3 oktober 2003.

“Wij kwamen gezamenlijk tot de conclusie dat een rookvrije horeca op termijn onvermijdelijk is. … Gezien de gezondheidsrisico's bij blootstelling aan tabaksrook zal het stappenplan maatregelen moeten bevatten die daadwerkelijk bijdragen aan het beschermen van de gezondheid van werknemers en gasten in de horeca.”

***In november* 2003** publiceert de Gezondheidsraad het rapport ‘Volksgezondheidsschade door passief roken’ (Gezondheidsraad, 2003). De belangrijkste conclusies in dit rapport, relevant voor het beoordelen van gezondheidsschade door passief roken in de horeca, zijn:

“De belangrijkste conclusies, die gelden voor een doorsnee blootstelling aan omgevingstabaksrook, zijn als volgt:
- *Er is voldoende bewijs geleverd dat passief roken longkanker kan veroorzaken. De risicoverhoging bedraagt ongeveer 20 procent. …*
- *Het is overtuigend aangetoond dat passief roken tot een hogere kans op hartaandoeningen leidt. De risicoverhoging is 20 à 30 procent.*
- *Kinderen hebben gemiddeld een lager gewicht en een geringere lengte bij de geboorte als de aanstaande moeders roken of meeroken. De risicoverhoging bedraagt ongeveer 20 tot 40 procent. …*
- *Er zijn aanwijzingen dat zowel prenatale als postnatale blootstelling aan omgevingstabaksrook bepaalde cognitieve vaardigheden (zoals taal- en leervermogen) en gedragskenmerken (zoals activiteit en concentratievermogen) van kinderen negatief beïnvloedt. De mate waarin dit het geval is, kan nog niet goed worden beoordeeld. …*
- *Er zijn aanwijzingen dat passief roken de kans op chronische luchtwegklachten bij volwassenen (in het bijzonder astmatici) vergroot.”* (p.7-8)

“Op basis van becijferingen uit de VS valt volgens de commissie wel een uitspraak te doen over de orde van grootte van de sterfte- en ziektelast. Naar schatting is passief roken in ons land jaarlijks de oorzaak van:
- *enkele honderden sterfgevallen door longkanker*
- *enkele duizenden sterfgevallen door hartaandoeningen …*
- *vele tienduizenden gevallen van (meer of minder ernstige) luchtwegaandoeningen bij kinderen”* (p.8)

Bij deze cijfers over sterfte en ziektelast ten gevolge van passief roken plaatst de Gezondheidsraad wel een kritische kanttekening:

“De commissie heeft zich vervolgens de vraag gesteld wat deze relatieve risico’s betekenen voor het aantal ziekte- en sterfgevallen dat jaarlijks in ons land aan passief roken te wijten is. Om die vraag te kunnen beantwoorden moet men beschikken over incidentiecijfers voor (sterfte door) de betrokken aandoeningen, gespecificeerd naar leeftijd, geslacht en rookstatus. Ook zijn prevalentiecijfers nodig voor blootstelling aan omgevingstabaksrook, gespecificeerd naar leeftijd, geslacht en locatie. De commissie heeft geconstateerd dat veel van de benodigde cijfers niet zonder meer beschik-

baar zijn. Voor een belangrijk deel lijken ze wel te achterhalen, maar dat vergt nader onderzoek. (p.8)

In juni 2006 publiceert de Surgeon General van de United States (US) het rapport *'The health consequences of involuntary exposure to tobacco smoke'* (USUDH, 2006). De belangrijkste conclusies, relevant voor het beoordelen van gezondheidsschade door passief roken bij werknemers in de horeca, zijn[6]:

> *"The reviews in this report reaffirm and strengthen the findings of the 1986 report. With regard to the involuntary exposure of nonsmokers to tobacco smoke, the scientific evidence now supports the following major conclusions*
>
> 1. *Secondhand smoke causes premature death and disease in children and in adults who do not smoke. growth in their children.*
> 3. *Exposure of adults to secondhand smoke has immediate adverse effects on the cardiovascular system and causes coronary heart disease and lung cancer.*
> 4. *The scientific evidence indicates that there is no risk-free level of exposure to secondhand smoke.*
> 6. *Eliminating smoking in indoor spaces fully protects nonsmokers from exposure to secondhand smoke. Separating smokers from nonsmokers, cleaning the air, and ventilating buildings cannot eliminate exposures of nonsmokers to secondhand smoke."* (p.11)

In januari 2007 brengt de Europese Commissie het Groenboek 'Op weg naar een rookvrij Europa' uit (EC, 2007):

> *"De meeste voor de gezondheid nadelige gevolgen van blootstelling aan omgevingstabaksrook vertonen een lineaire dosis-effectrelatie. Met andere woorden: het risico neemt in gelijke mate toe met de blootstelling. Het individuele risico is kleiner dan bij actief roken (bijvoorbeeld 1,2 resp. 20 voor longkanker). Niettemin is de ziektelast aanzienlijk door het feit dat grote aantallen mensen worden blootgesteld. De dosis-effectrelatie voor hartziekten is niet-lineair. Iemand die passief rookt heeft maar liefst bijna half zoveel risico een hartziekte te krijgen als iemand die twintig sigaretten per dag rookt. Ook heel kleine doses tabaksrook kunnen een onmiddellijk effect hebben op het ontstaan van stremmingen in de bloedsomloop en trombose, alsmede een langetermijneffect op de ontwikkeling van arteriosclerosis – allemaal belangrijke factoren bij hartziekten. Dit heeft een enorme impact: hartziekten vormen de meest voorkomende doodsoorzaak in de EU onder zowel rokers als niet rokers."* (p.7)
>
> *"Er is geen veilig niveau van blootstelling aan omgevingstabaksrook geconstateerd, en naar verwachting zal verder onderzoek een dergelijk niveau ook niet weten te identificeren."* (p.5)

6. Zoals reeds eerder in een voetnoot vermeld, is het rapport van de Surgeon General uit 2006 een zeer uitgebreide reviewstudie. Het rapport telt ruim 2000 literatuurverwijzingen.

"Volgens de meest recente – conservatieve – schattingen door het partnerschap van de European Respiratory Society, Cancer Research UK en het Franse Institut National du Cancer overlijden er ieder jaar meer dan 79 000 volwassenen in de 25 landen van de EU als gevolg van passief roken. Er zijn bewijzen dat passief roken op het werk in de EU in 2002 geleid heeft tot 7 000 sterfgevallen. Blootstelling aan omgevingstabaksrook thuis was verantwoordelijk voor nog eens 72 000 doden. In deze schattingen zijn opgenomen: sterfgevallen door hartziekten, longkanker en enkele ademhalingsaandoeningen die worden veroorzaakt door passief roken. Niet opgenomen zijn sterfgevallen door toedoen van andere omstandigheden die in verband staan met blootstelling aan omgevingstabaksrook (zoals longontsteking), overleden kinderen en de vele andere ernstige, zowel acute als chronische, aandoeningen die worden veroorzaakt door passief roken" (p.6)

De conclusies in het Groenboek zijn ondermeer gebaseerd op de conclusies van het bovengenoemde rapport van de Surgeon General uit juni 2006. Opvallend in het Groenboek is m.i. de verhouding in 2002 tussen het aantal doden ten gevolge van omgevingstabaksrook thuis (72.000) en omgevingstabaksrook op het werk (7.000), namelijk een factor 10. Dit verschil kan echter mede worden veroorzaakt door de rookverboden op het werk, die in veel landen in 2002 al gedeeltelijk waren ingevoerd. Door het tabaksontmoedigingsbeleid, waarbij verschillende maatregelen gelijktijdig worden ingevoerd, zijn de cijfers over sterfte en ziektelast ten gevolge van omgevingstabaksrook moeilijk te interpreteren.

In mei 2007 onderschrijft de Nederlandse regering de juistheid en volledigheid van de cijfers in het Groenboek van de Europese Commissie[7]:

"Het Nederlandse kabinet constateert dat de Europese Commissie met het door haar uitgebrachte Groenboek de onderhavige problematiek in al zijn facetten adequaat, correct en volledig beschrijft." (p.1)

In juni 2007 schrijft minister Klink van VWS in zijn brief aan de Tweede Kamer over zijn besluit tot invoering van een rookvrije horeca zonder uitzonderingen[8]:

"Ik heb besloten om niet mee te gaan in het voorstel van KHN omdat: 1. dan niet alle werknemers (secundair: bezoekers) in de horeca beschermd worden tegen tabaksrook (ongelijke behandeling) en juist de werknemers die de hoogste blootstelling aan tabaksrook hebben (cafés, discotheken), worden uitgezonderd; …" (p.3)

---

7. Reactie van Nederland op de vragen van de Europese Commissie in het Groenboek 'Op weg naar een rookvrij Europa. Brief van Minister Klink aan de Eerste Kamer d.d. 15 mei 2007.
8. TK, 200602007, 30800 XVI, nr.149.

In juli 2007 stelt minister Klink in een algemeen overleg met de Tweede Kamer over zijn besluit tot invoering van een rookvrije horeca[9]:

> *"… Juist vanwege de schade die meeroken veroorzaakt voor werknemers en bezoekers, moet de horeca rookvrij gemaakt worden. … In discotheken gaat het vaak om 600–1000 microgram schadelijke deeltjes en in een café en in een restaurant om gemiddeld 200 microgram, terwijl in de openbare ruimte slechts 50 microgram getolereerd wordt."* (p.6-7)

In december 2008 schrijft minister Klink in een brief aan de Tweede Kamer[10]:

> ***"****De ervaringen in andere landen laten zien dat de luchtkwaliteit in de horeca na de invoering van een rookverbod substantieel verbetert en dat de blootstelling van werknemers aan tabaksrook sterk daalt. … In enkele landen is onderzoek gedaan naar veranderingen in het aantal hartaanvallen onder de bevolking voor en na de invoering van rookverboden. Deze onderzoeken uit de staat New York, Italië en Schotland laten dalingen in het aantal ziekenhuisopnames voor hartaanvallen zien van respectievelijk 8, 11 en 17 procent."* (p.2)

In 2009 herbevestigt een wetenschappelijk onderzoek (een second opinion), dat heeft plaatsgevonden in opdracht van het ministerie van VWS, dat er geen veilig niveau van blootstelling aan omgevingstabaksrook bestaat (Brunekreef. QANU, 2009):

> *"There are no standards with which the amount of second hand smoke (SHS) or maximum levels that can be found in smoke-free rooms can be compared. This is because second hand smoke causes cancer and other adverse effects on health, and no safe levels of exposure exist other than absence."* (p.3)

In 2010 publiceert de Surgeon General van de US het rapport 'How tobacco smoke causes disease. The biology and behavioral basis for smoking-attributable disease' (USUDH, 2010):

> *"As mentioned previously, the substantial CVD [cardiovascular disease] risk associated with involuntary exposure to cigarette smoke indicates that the risks estimated in most studies of active smoking are biased downward because the control groups generally included large numbers of persons with exposure to secondhand smoke."* (p.363)

De 'Executive summary' bij dit rapport stelt:

> *"In 2006, the Surgeon General reported that the scientific evidence was sufficient to conclude that there is no risk-free level of exposure to secondhand smoke (U.S.*

9. TK, 2006-2007, 30800 XVI, nr.182.
10. TK, vergaderjaar 2008–2009, 22894, nr.206.

Department of Health and Human Services [USDHHS] 2006). This report [2010] provides a more detailed review of the mechanisms that validate that conclusion. A risk-free level of exposure to tobacco smoke (whether mainstream or from secondhand smoke) cannot be defined." (p.2)

Onderzoek ter onderbouwing van het rookbeleid in de horeca
Ter onderbouwing van het rookbeleid in de horeca heeft in opdracht van de Nederlandse overheid veel onderzoek plaatsgevonden (zie §10.2). Zo is onderzoek uitgevoerd naar de resultaten van de zelfregulering van het rookbeleid in de horeca en naar de resultaten van het rookbeleid in de sport. Ook zijn de gevolgen en de naleving van het rookverbod in de horeca en (alternatieven voor) rookvrije ruimten onderzocht. Daarnaast is onderzoek uitgevoerd naar de gevolgen van passief roken voor de volksgezondheid.

Conclusies inzake gezondheidsschade
Inzake de gezondheidsschade die aanleiding vormt tot de invoering van het wettelijk rookverbod in de horeca kunnen ondermeer de volgende conclusies worden getrokken:

- het gaat om risico's op gezondheidsschade die niet-rokende werknemers in de horeca lopen, doordat zij tijdens hun werkzaamheden in de horeca worden blootgesteld aan passief roken;
- onder bovengenoemde risico's vallen ook de risico's verbonden aan prenatale blootstelling aan passief roken van kinderen van zwangere werknemers;
- de afgelopen 15 jaar is het wetenschappelijk bewijs dat passief roken schadelijk is voor de gezondheid steeds sterker geworden;
- er is voldoende wetenschappelijk bewijs dat passief roken leidt tot een substantieel risico op ernstige gezondheidsschade bij werknemers in de horeca;
- de gezondheidsschade die niet-rokers door passief roken kunnen oplopen, betreft schade toegebracht door derden;
- er zijn nationaal en internationaal grote wetenschappelijke inspanningen gedaan om de schade ten gevolge van passief roken te objectiveren c.q. kwantificeren en hierdoor is veel beleidsrelevante kwantitatieve informatie beschikbaar gekomen;
- de Nederlandse overheid heeft zelf ook veel onafhankelijk onderzoek laten uitvoeren ter onderbouwing van het wettelijk rookverbod in de horeca;
- de Nederlandse overheid heeft bij de onderbouwing van het wettelijk rookverbod in de horeca zeer goed gebruik gemaakt van de nationaal en internationaal beschikbare wetenschappelijke informatie.

Het meeste wetenschappelijke onderzoek dat heeft plaatsgevonden naar de schadelijkheid van passief roken, betreft de schadelijkheid van passief roken in het algemeen en niet de schadelijkheid van passief roken voor werknemers in de Nederlandse horeca. Op basis van de beschikbare wetenschappelijke informatie kunnen geen harde conclusies worden getrokken m.b.t. het aantal ziekte- en sterftegevallen onder werknemers in de Nederlandse horeca ten gevolge van passief roken, voorafgaand aan de invoering van het wettelijk rookverbod. Dergelijke cijfers zijn ook in andere landen niet beschikbaar en m.i. zou het een onevenredig grote onderzoeksinspanning vergen om dergelijke cijfers vast te stellen. Het aantal factoren dat deze cijfers beïnvloedt, is te groot. Ter illustratie noem ik er een aantal:

- variatie in aantal werkuren, werktijden en werkzaamheden van werknemers;
- variatie in concentratie van omgevingstabaksrook in verschillende horecagelegenheden;
- passief roken door niet-rokende werknemers buiten werktijd;
- (on)gezonde leefstijl van niet-rokende werknemers;
- eigen rookbeleid van horecagelegenheden (bv. rookverboden, rookvrije delen in restaurants, ventilatie);
- invloed van andere preventiemaatregelen van de overheid in het kader van het tabaksontmoedigingsbeleid op rookgedrag en blootstelling aan passief roken.

In 1999 benoemt minister Borst-Eilers een aantal kwetsbare groepen bij wie blootstelling aan tabaksrook extra risico's voor de gezondheid en het welbevinden met zich brengt: CARA-patiënten, zwangere vrouwen en kinderen. Het stappenplan ter zelfregulering van het rookbeleid van KHN uit 2003 besteedt extra aandacht aan de bescherming van kinderen tegen tabaksrook in de horeca. Nadat minister Klink in 2007 heeft besloten tot de invoering van een algeheel wettelijk rookverbod in de horeca, wordt niet meer gesproken over extra bescherming van kwetsbare groepen, zoals CARA-patiënten, zwangere vrouwen en kinderen, tegen passief roken in de horeca. Het streven van minister Klink is immers om een <u>algeheel</u> wettelijk rookverbod in de horeca in te voeren. Extra aandacht voor de bescherming van kwetsbare groepen tegen passief roken in de horeca is daarom overbodig. Op het moment dat er <u>geen algeheel</u> wettelijk rookverbod meer is, wordt extra aandacht voor de bescherming van kwetsbare groepen weer relevant. Dit moment ontstaat als minister Schippers in 2010 beslist om het wettelijke rookverbod voor kleine cafés zonder personeel op te heffen. Aan de consequenties hiervan voor de gezondheid van kwetsbare groepen besteedt zij geen aandacht (ik heb hierover van haar geen uitspraak kunnen vinden).

Sociaaleconomische schade (criterium schadelijkheid)
In 1995 stelt het kabinet in de nota 'Gezond en wel'[11] dat het voorkomen van economische schade geen doelstelling is van het preventiebeleid op het terrein van de volksgezondheid (inclusief het tabaksontmoedigingsbeleid). Het kabinet geeft als reden aan dat het per saldo goedkoper is, als mensen minder oud worden:

> *"In aanmerking genomen dat op latere leeftijd een grote kans bestaat op «vervangende ziekten», zal preventie de gezondheidszorg zelden goedkoper maken, zeker niet op langere termijn gezien. De «gespaarde» individuen hebben immers op latere leeftijd gemiddeld een relatief hoge medische consumptie: 75-plussers gebruiken per jaar gemiddeld voor ruim f 8000 aan zorgvoorzieningen, terwijl degenen die de 40 nog niet gepasseerd zijn gemiddeld jaarlijks voor minder dan f 2000 consumeren. Gezondheidsbevordering en preventie van ziekten zullen op den duur dus de totale vraag naar zorgvoorzieningen in de samenleving doen toenemen. Echter, het is de verbetering van gezondheid en welbevinden die de verdere uitbouw van preventie rechtvaardigt, en niet de eventuele besparingen."* (p.20)

In 2002 zegt minister Borst-Eilers tijdens de behandeling van het wetsvoorstel voor wijziging van de Tabakswet in de Eerste Kamer echter[12]:

> *"Minister Borst-Eilers: ... De zaak kan ook op een macroeconomische manier bekeken worden. De algemene conclusie van de economen is dat roken ook slecht is voor de economie. De Wereldbank heeft dit in een rapport toegelicht: in macro-economisch opzicht kost roken meer dan het oplevert. Ook vanuit dat oogpunt is de boodschap aan overheden: doe er wat aan."* (p.1257)

In 2007 gebruikt de Europese Commissie in het Groenboek 'Op weg naar een rookvrij Europa' wel economische argumenten ter rechtvaardiging van rookverboden (EC, 2007):

> *"Uit gegevens uit lidstaten (Verenigd Koninkrijk, Ierland) en uit landen van buiten de EU komt naar voren dat de blootstelling aan omgevingstabaksrook tot torenhoge private en sociale kosten leidt … Tot de belasting voor de economie als geheel behoren de directe kosten voor hogere uitgaven voor de gezondheidszorg met betrekking tot tabaksgerelateerde ziekten. Ook worden er indirecte kosten gemaakt in verband met productiviteitsverliezen en worden er minder belastingen en socialeverzekeringsbijdragen geïnd bij actieve en passieve rokers die anders betaalde arbeid hadden verricht. De economische belasting is vooral hoog voor werkgevers, bijvoorbeeld door rookpauzes, meer ziekmeldingen en de daarmee gepaard gaande lagere productiviteit van deze werknemers. Ook brandschade, extra schoonmaakkosten en noodzakelijke opknapbeurten van het interieur leiden tot hogere kosten. In Canada werden de*

11. TK, 1994-1995, 24126, nrs.1-2.
12. EK, 2001-2002, vergaderingnr.24, p.1236-1275 (dossiernr.26472).

jaarlijkse kosten per rokende werknemer, vergeleken met de kosten voor een vergelijkbare niet-rokende werknemer, geschat op $2 565 in Canadese dollars (1995). …" (p.7-8)

In het Groenboek worden aansluitend echter ook onbedoelde economische gevolgen van rookverboden genoemd, die de economische voordelen voor een deel teniet doen:

> *"Een "rookvrij"-beleid zal sommige rokers motiveren om te stoppen met roken of om minder te gaan roken. Dat kan leiden tot lagere winsten voor de tabaksindustrie en dientengevolge tot vermindering van de werkgelegenheid in de tabaksector. … Beperking van het actieve roken zal eveneens betekenen dat de lidstaten minder belastinginkomsten (accijnzen, BTW) genereren uit sigaretten. Er kan enig productieverlies worden verwacht van verstokte rokers die op dit moment nog op het werk mogen roken, en die rookpauzes buiten het gebouw zullen nemen."* (p.8-9)

De Europese commissie weet de omvang van deze economische nadelen overtuigend te relativeren (EC, 2007, p.8-9). Het grootste bezwaar tegen de rechtvaardiging van rookverboden op basis van economische overwegingen door de Europese Commissie acht ik echter, dat de Commissie onvolledig is in het opsommen van de economische nadelen van rookverboden. Ik denk dat het Nederlandse kabinet 1995 gelijk had, toen het in de kabinetsnota 'Gezond en wel'[13] stelde dat preventie op het terrein van de volksgezondheid op de lange termijn niet leidt tot kostenbesparingen, omdat mensen ten gevolge van deze preventie ouder worden. Het kabinet stelde dat de kosten van gezondheidszorg hierdoor per saldo hoger zouden worden. Als daar ook de extra pensioenkosten nog bij betrokken worden, wordt het onwaarschijnlijk dat rookverboden op macroniveau per saldo zullen leiden tot kostenbesparingen. Uit een onderzoek van het RIVM naar de kosteneffectiviteit van tabaksontmoedigingsbeleid in Nederland blijkt dat zelfs een accijnsverhoging van tabaksproducten tot hogere zorgkosten leidt (dit is dus nog exclusief hogere pensioenkosten), omdat mensen minder gaan roken en als gevolg daarvan ouder worden (RIVM, 2005, p.3).

Uit het totaal van de door mij bestudeerde documenten blijkt dat de Nederlandse regering preventie van economische schade in het algemeen niet aanvoert als rechtvaardiging voor rookverboden; dus ook niet als rechtvaardiging van een rookverbod in de horeca. Daar het onwaarschijnlijk is dat rookverboden op macroniveau tot kostenbesparingen leiden, acht ik dit een terechte keuze. In het

13. TK, 1994-1995, 24126, nrs.1-2.

kader van het model neem ik de preventie van economische schade daarom niet op als doelstelling van het rookverbod in de horeca.

De preventie van ***immateriële maatschappelijke kosten is in 1995*** wel een uitgangspunt van het tabaksontmoedigingsbeleid van de regering, blijkt uit de onderstaande citaten die op elkaar aansluiten:

> *"Uitgangspunt voor de regering is en blijft dat roken schade voor de volksgezondheid oplevert en hoge maatschappelijke kosten veroorzaakt. Succesvolle preventie van roken betekent dan ook een grote gezondheidswinst en veel maatschappelijk rendement."* (p.1)[14]

> *"In verband met de passages in de brief [zie het citaat hierboven] over de hoge maatschappelijke kosten van het roken herinnerde zij aan de uitspraak van de minister van VWS … dat zowel voor de Verenigde Staten als voor Nederland is uitgerekend dat de kosten van de volksgezondheid aanzienlijk zouden stijgen als iedere roker morgen met roken zou stoppen. Ook het NEI-rapport komt tot die conclusie. Tegen die achtergrond vroeg mevrouw Van Blerck wat nu precies moet worden verstaan onder «hoge maatschappelijke kosten»."* (p.7)[15]

> *"Onder «maatschappelijke kosten» verstond de minister ook de immateriële maatschappelijke kosten, bijvoorbeeld in de gevallen waarin mensen op een jonge leeftijd ernstig ziek worden en overlijden en een jong gezin achterlaten. Voor de gezondheidszorgkosten geldt inderdaad dat deze aanzienlijk zouden stijgen als iedere roker morgen met roken zou stoppen, maar dit komt geheel anders te liggen op het moment dat longkanker en andere vormen van kanker goed behandelbaar worden. Het draaipunt is hier dat rokers nu nog vaak op jonge leeftijd overlijden aan een ziekte die vrij kort duurt en dan worden inderdaad maar weinig gezondheidszorgkosten gemaakt."* (p.20)

Bij immateriële maatschappelijke kosten kan ook worden gedacht aan het leed dat arbeidsongeschiktheid veroorzaakt:

> *"Dat werknemers ziek worden van de rook van collega's en vervolgens in de WAO belanden vinden de leden van de D66-fractie onacceptabel. Helaas gebeurt dit nog steeds. Een werkgever dient ervoor te zorgen dat een werknemer in een rookvrije ruimte kan werken, zo menen zij."* (p.22)[16]

14. TK, 1995-1996, 24743, nr.1.
15. TK, 1996-1997, 24743, nr.3.
16. TK, 1998-1999, 26472, nr.5.

Inbreuk op het persoonlijk leven (criterium schadelijkheid)
Een inbreuk op het persoonlijk leven heb ik t.b.v. het model geoperationaliseerd als een aantasting van de:

– lichamelijke integriteit;
– vrijheid;
– privacy;
– gelijke behandeling;
– eer en goede naam/ waardigheid/ respectvolle bejegening.

In juli 1999 stelt de PvdA in een brief van de vaste commissie voor VWS aan minister Borst-Eilers[17]:

> *"Intussen zijn wel vele duizenden werknemers dag in dag uit blootgesteld aan rook-overlast. En is het voor astma- en COPD-patiënten in veel gevallen onmogelijk betaalde arbeid te verrichten. … Deze leden zijn van mening dat aan de behoeften van werknemers op een betere wijze tegemoet moet worden gekomen dan mogelijk is, door nog langer te wachten op de totstandkoming van convenanten. … Zij bepleiten tenslotte dat de AMvB die werkgevers verplicht een rookverbod in te stellen op de gemeenschappelijke werkplek dwingend wordt voorgeschreven."* (p.19-20)

De PvdA wijst erop dat blootstelling aan tabaksrook op de werkplek leidt tot uitsluiting van bepaalde werknemers van (een deel van) de arbeidsmarkt. Ik interpreteer dit als een aantasting van de keuzevrijheid van werknemers om daar te werken (solliciteren) waar men wil.

In mei 2000 antwoordt minister Borst-Eilers op de bovengenoemde brief[18]:

> *"Dag in dag uit worden vele duizenden werknemers onvrijwillig blootgesteld aan hinder, overlast en schade door passief meeroken. Voor astma- en COPD/CARA-patiënten is het om deze reden in veel gevallen onmogelijk betaalde arbeid te verrichten, aldus deze leden, … Met deze leden is ook de regering van mening dat aan de wensen en behoeften van niet rokende werknemers op een betere en andere wijze tegemoet zal moeten worden gekomen dan tot nu toe mogelijk is gebleken via zelfregulering en het wachten op de totstandkoming van convenanten."* (p.33)

> *"In dit verband heeft de rechter te Breda op 25 april 2000 uitgesproken dat niet-rokers ten principale recht hebben op een rookvrije werkplek. Het recht op bescherming van de lichamelijke integriteit, de gezondheid, is een grondrecht. Als uitgangspunt voor deze rechterlijke uitspraak gold dat er voor een kankerverwekkende stof als tabaksrook geen veilige ondergrens qua blootstelling is te bepalen. Daarom is een*

17. TK, 1998–1999, 26472, nr.5
18. TK, 1999–2000, 26472, nr.6

> *algeheel rookverbod in kantoorgebouwen en andere besloten werkruimten in het algemeen aangewezen, aldus dit vonnis. …"* (p.34)

In het bovenstaande citaat worden de bescherming van de lichamelijke integriteit en gezondheid in een adem genoemd. Dit duidt erop dat er overlap zit in de subcriteria 'gezondheidsschade' en 'aantasting van de lichamelijke integriteit' van het model. Toch acht ik het van belang om onderscheid te maken tussen deze subcriteria. Bij aantasting van de lichamelijk integriteit is er sprake van aanranding of binnendringing van het lichaam van derden. Dit kan direct via lichamelijk contact, maar ook indirect, bijvoorbeeld in de vorm van passief roken. Gezondheidsschade ten gevolge van het gedrag van derden kan ook optreden zonder dat er sprake is van aantasting van de lichamelijke integriteit. Denk bijvoorbeeld aan een werkgever die een werknemer verplicht te lang achtereen door te werken zonder rustpauze wat tot gezondheidsklachten kan leiden. Of denk aan ouders die kinderen te weinig groente en fruit te eten geven. Omdat aantasting van de lichamelijke integriteit in het algemeen ernstig wordt gevonden, acht ik het van belang deze vorm van schade te onderscheiden van gezondheidsschade in het algemeen. Bovendien kan er sprake zijn van een aantasting van de lichamelijke integriteit, zonder dat er sprake hoeft te zijn van gezondheidsschade. Denk bijvoorbeeld aan verkrachting. Verkrachting leidt niet automatisch tot gezondheidsschade, maar vormt wel een ernstige aantasting van de lichamelijke integriteit c.q. een ernstige inbreuk op het persoonlijk leven. Bij het subcriterium 'gezondheidsschade' ligt de focus op (de onaanvaardbaarheid van) de gezondheidsschade die wordt toegebracht. Bij het subcriterium 'aantasting van de lichamelijke integriteit' ligt de focus op (de onaanvaardbaarheid van) het grensoverschrijdend gedrag van derden.

In juli 2007 vindt een algemeen overleg plaats in de Tweede Kamer over het besluit van Minister Klink om per 1 juli 2008 een algeheel wettelijk rookverbod in de horeca in te voeren[19]:

> *"Mevrouw Agema (PVV) is tegen een algemeen rookverbod in de horeca, ondanks dat algemeen bekend is dat roken slecht is voor de gezondheid. … Er zijn inmiddels voldoende rookvrije horecabedrijven. <u>De mensen hebben een keuze.</u> (p.2)*

> *"Mevrouw Schippers (VVD) … In het VVD-voorstel <u>kan men kiezen</u> tussen het bezoeken van een kroeg waar wordt gerookt en die goed wordt geventileerd of een rookvrije kroeg." (p.3)*

De bovenstaande uitspraken van de Tweede Kamerleden Agema en Schippers staan haaks op de uitspraak van de rechter in april 2000 dat werknemers ter

19. TK, 2006-2007, 30800 XVI, nr.182.

bescherming van hun lichamelijke integriteit recht hebben op en rookvrije werkplek.

Overlast (criterium schadelijkheid)

De 'Wet van 18 april 2002 tot wijziging van de Tabakswet' stelt in artikel I H[20]:

> *"Artikel 10 wordt als volgt gewijzigd: 1. In het eerste lid wordt «zonder dat daarbij hinder van het gebruik van tabaksprodukten wordt ondervonden» vervangen door: zonder daarbij hinder of overlast van roken te ondervinden."*

Artikel 11a lid 1 van de Tabakswet luidt[21]:

> *"Werkgevers zijn verplicht zodanige maatregelen te treffen dat werknemers in staat worden gesteld hun werkzaamheden te verrichten zonder daarbij hinder of overlast van roken door anderen te ondervinden."*

Daarom voeg ik aan de vierde risicocategorie 'overlast' ten behoeve van het model het begrip ***'hinder'*** toe.

In de door mij bestudeerde documenten wordt het begrip hinder vooral gekoppeld aan de persoon (benauwdheid, hoofdpijn, duizeligheid, irritatie, verminderd smaakvermogen) en het begrip overlast aan de omgeving (stankoverlast, geluidsoverlast). In het kader van de toetsing van het model aan de casus 'rookverbod' in de horeca, voert het te ver om het onderscheid tussen hinder en overlast nader uit te diepen.

In de door mij bestudeerde documenten komen de begrippen 'hinder' en 'overlast' ten gevolge van tabaksrook, zowel afzonderlijk als tezamen, regelmatig voor. Ter illustratie worden achtereenvolgens Minister Borst-Eilers (1999) en de Gezondheidsraad (2003) geciteerd:

> *"Daarnaast veroorzaakt tabaksrook hinder en overlast voor niet-rokers, vooral wanneer zij lijden aan aandoeningen van de ademhalingswegen, zoals astma, … " (p.1) … "Behalve schadelijk is passief roken ook een bron van ongemak en een bedreiging van het welbevinden en functioneren van de werknemer. Recent onderzoek laat zien dat maar liefst twee derde van de niet rokende werknemers soms tot vaak hinder ondervindt van tabaksrook op het werk. Dit kan variëren van hardnekkig aanhoudende stank tot irritatie van ogen, neus en keel. Tabaksrook vermindert het smaakvermogen en kan hoofdpijn, misselijkheid en duizeligheid veroorzaken."* (p.8)[22]

20. Staatsblad, 2002, nr.201.
21. Tabakswet, geldend op 26 maart 2011.
22. TK, 1998–1999, 26472, nr.3.

> *"… passief roken … Verder doet hinder door stank en irritatie zich op grote schaal voor."* (Gezondheidsraad, 2003, p.9)

Een belangrijk aandachtpunt bij het vaststellen van de omvang en ernst van hinder en overlast, is dat de ervaren hinder en overlast mede worden bepaald door individuele gevoeligheid. Astmapatiënten kunnen bijvoorbeeld hinder ondervinden van tabaksrook omdat zij het benauwd krijgen, terwijl mensen zonder astma het alleen maar vinden stinken of zeggen nergens last van te hebben. In dit verband is het van belang dat er binnen de Nederlandse bevolking consensus lijk te bestaan dat tabaksrook hinderlijk is voor alle niet-rokers:

> *"Vier op de vijf mensen [Nederlanders] zijn van mening dat niet-rokers doorgaans hinder ondervinden van tabaksrook. Ook de rokers zèlf vinden dat."* (p.8)[23]

Schending van morele opvattingen (criterium schadelijkheid)
Uit de door mij bestudeerde documenten is niet gebleken dat morele opvattingen zijn aangevoerd ter rechtvaardiging van het rookverbod in de horeca in Nederland.

11.1.3 Eindoordeel (criterium schadelijkheid)

Overzicht 18 geeft een overzicht van de 'risicocategorieën' op basis waarvan de achtereenvolgende kabinetten de totstandkoming van het rookverbod in de horeca hebben gerechtvaardigd. De schade, hinder en overlast ten gevolge van passief roken voor bezoekers, die in ieder geval t/m juli 2007 voor de overheid een belangrijke reden vormde om een rookvrije horeca na te streven, wordt buiten beschouwing gelaten. De reden is dat minister Klink het rookverbod in de horeca alleen heeft ingevoerd ter bescherming van werknemers in de horeca tegen passief roken en niet ter bescherming van bezoekers tegen passief roken (zie §10.5). Ook de schade, hinder en overlast voor bedrijfseigenaars, ten gevolge van passief roken in hun horecazaak, wordt buiten beschouwing gelaten. Preventie van deze schade, hinder en overlast vormt geen doelstelling van het wettelijk rookverbod in de horeca (zie §10.6.3).

23. TK, 1998–1999, 26472, nr.3.

Overzicht 18: aanleiding tot het rookverbod in de horeca

Risicocategorieën	*Risico's die de overheid tracht te bestrijden*
**Gezondheidsschade**	
aan zichzelf	Nee
aan derden	Risico op gezondheidsschade bij werknemers ten gevolge van passief roken
**Sociaaleconomische schade**	
aan zichzelf	Nee
aan derden	Risico op immateriële maatschappelijke kosten voor werknemers ten gevolge van passief roken (leed door ziekte, arbeidsongeschiktheid, of voortijdig overlijden)
**Inbreuk op het persoonlijk leven**	Aantasting van de lichamelijke integriteit van werknemers. Aantasting van de keuzevrijheid van werknemers om daar te werken (solliciteren) waar zij willen. Uitsluiting van patiënten met astma of COPD van (een deel van) de arbeidsmarkt ten gevolge van passief roken.
**Hinder of overlast**	Hinder en overlast voor werknemers ten gevolge van passief roken (benauwdheid hoofdpijn, duizeligheid, irritatie, verminderd smaakvermogen, stank)
**Schending van morele opvattingen**	Nee

De beschikbare informatie geeft een duidelijk beeld van de aard en omvang van de risico's op gezondheidsschade, die aanleiding vormen tot het wettelijk rookverbod in de horeca. Deze risico's zijn voldoende gekwantificeerd en wetenschappelijk onderbouwd. Het aantal ziekte- en sterftegevallen onder niet-rokers ten gevolge van passief roken in de (Nederlandse) horeca is onbekend en redelijkerwijs niet wetenschappelijk vast te stellen, omdat het van zoveel verschillende factoren afhankelijk is.

De beschikbare informatie geeft een duidelijk beeld van de aard van de risico's op sociaaleconomische schade, die aanleiding vormen tot het rookverbod in de horeca. Het blijkt te gaan om leed door ziekte, arbeidsongeschiktheid, of voortijdig overlijden. Er is geen reden om eraan te twijfelen dat deze schade zich voordoet. De omvang van deze schade is echter onvoldoende gekwantificeerd.

Dat passief roken een ernstige aantasting vormt van de lichamelijk integriteit van werknemers in de horeca is evident en bevestigd in een rechterlijke uitspraak. Mede op basis hiervan is in 2008 een rookverbod in de horeca ingevoerd, waardoor werknemers in de horeca niet meer aan passief roken op hun werkplek worden blootgesteld.

Dat passief roken leidt tot uitsluiting van ondermeer astma- en COPD-patiënten van de arbeidsmarkt in de horeca, is aannemelijk. De omvang van deze schade is echter onvoldoende gekwantificeerd. Dit geldt zowel voor de arbeidsmarkt als geheel als voor de arbeidsmarkt van de horecasector.

De beschikbare informatie geeft een duidelijk beeld van de aard van de risico's op hinder en overlast, die aanleiding vormen tot het rookverbod in de horeca. Het blijkt te gaan om benauwdheid, hoofdpijn, duizeligheid, irritatie, verminderd smaakvermogen en stank. Er is geen reden om eraan te twijfelen dat deze hinder en overlast zich voordoen. De omvang van deze hinder en overlast, die werknemers ervaren, is echter onvoldoende gekwantificeerd.

11.1.4 Gevolgen van de beoordeling voor het model (criterium schadelijkheid)

Uit de beoordeling van het wettelijk rookverbod in de horeca aan de hand van het criterium 'schadelijkheid' is duidelijk geworden, dat het voor de toepassing van het model essentieel is om eenduidig vast te stellen welke schade aanleiding vormt tot het treffen van de preventiemaatregel. Als daarover onduidelijkheid bestaat, is de gerechtvaardigdheid van de preventiemaatregel aan de hand van het model niet te beoordelen, omdat alle volgende criteria van het model de preventie van deze schade als vertrekpunt hebben. Bij de beoordeling van het wettelijk rookverbod in de horeca aan de hand van het criterium 'schadelijkheid' liep ik ertegenaan, dat voor mij onduidelijk was of de bescherming van bezoekers in de horeca tegen gezondheidsschade ten gevolge van passief roken al dan niet een doelstelling van het wettelijk rookverbod was. Het wettelijk rookverbod in de horeca leidt immers wel tot de bescherming van bezoekers tegen deze schade. Als de bescherming van bezoekers wel een doelstelling van het rookverbod is, is deze bescherming medebepalend voor de 'effectiviteit' (7e criterium van het model) van het rookverbod. Als de bescherming van bezoekers geen doelstelling van het rookverbod is, is deze bescherming 'slechts' een 'positief neveneffect' (onderdeel van het 9e criterium 'kosten-baten-verhouding'). Enigszins tot mijn verbazing ben ik tot de conclusie gekomen dat de bescherming van bezoekers in de horeca tegen passief roken geen doelstelling is van het wettelijk rookverbod. Zie voor een nadere toelichting §10.5.

Aan de vierde schadecategorie 'overlast' van het model voeg ik het begrip 'hinder' toe. De vierde schadecategorie van het model luidt in het vervolg ***'hinder of overlast'***.

11.2 Tweede criterium: noodzakelijkheid

11.2.1 Operationalisering en wijze van beoordeling

Onder het 1e criterium 'schadelijkheid' is beoordeeld wat de aard en omvang is van de risico's die men tracht te bestrijden. Het 2e criterium 'noodzakelijkheid' betreft de vraag in hoeverre het noodzakelijk is om deze risico's te bestrijden. Belangrijke aandachtspunten zijn:

- In hoeverre is de preventiemaatregel moralistisch (tracht men de schending van morele opvattingen te bestrijden)?
- In hoeverre is de preventiemaatregel perfectionistisch (overdrijft men de risicopreventie)?
- Is er sprake van sancties die worden opgelegd aan personen of organisaties die de risico's (mede) veroorzaken? Deze kunnen een indicatie geven van de noodzakelijkheid van de risicopreventie vanuit de optiek van de sanctionerende partij.

Beoordeeld wordt of overtuigend wordt beargumenteerd dat het toepassen van de preventiemaatregel noodzakelijk is.

11.2.2 Beoordeling van casus 'wettelijk rookverbod in horeca' aan de hand van criterium 'noodzakelijkheid'

De redenen voor het instellen van een wettelijk rookverbod in de horeca zijn (zie overzicht 18 in §11.1.3):

- Gezondheidsschade:
  - risico op gezondheidsschade bij werknemers ten gevolge van passief roken;
- Sociaaleconomische schade:
  - risico op immateriële maatschappelijk kosten voor werknemers ten gevolge van passief roken (leed door ziekte, arbeidsongeschiktheid, of voortijdig overlijden);
- Inbreuk op het persoonlijk leven:
  - Aantasting van de lichamelijke integriteit van werknemers;
  - Aantasting van de keuzevrijheid van werknemers om daar te werken (solliciteren) waar zij willen. Uitsluiting van patiënten met astma of COPD van (een deel van) de arbeidsmarkt ten gevolge van passief roken;
  - Hinder of overlast:
  - hinder en overlast voor werknemers ten gevolge van passief roken (benauwdheid, hoofdpijn, duizeligheid, irritatie, verminderd smaakvermogen, stank).

Daarom tracht ik voor deze kwesties de noodzakelijkheid van het treffen van preventiemaatregelen te beoordelen.

Gezondheidsschade (criterium noodzakelijkheid)

In 2000 schrijft Minister Borst-Eilers aan de Tweede Kamer[24]:

> *"In dit verband heeft de rechter te Breda op 25 april 2000 uitgesproken dat niet-rokers ten principale recht hebben op een rookvrije werkplek. Het recht op bescherming van de lichamelijke integriteit, de gezondheid, is een grondrecht. Als uitgangspunt voor deze rechterlijke uitspraak gold dat er voor een kankerverwekkende stof als tabaksrook geen veilige ondergrens qua blootstelling is te bepalen. Daarom is een algeheel rookverbod in kantoorgebouwen en andere besloten werkruimten in het algemeen aangewezen, aldus dit vonnis. Kortom, het grondrecht op gezondheid kan langs privaatrechtelijke weg worden gewaarborgd, maar om te voorkomen dat nog vele andere niet-rokers zich tot de rechter moeten wenden, is het zaak hiervoor publiekrechtelijke waarborgen te scheppen. Het is tegen deze achtergrond niet alleen wenselijk, maar ook noodzakelijk om te komen tot een besluit inzake een rookvrije werkplek, …" (p.34)*

In mei 2003 stelt de World Health Organization in artikel 3 van het Kaderverdrag inzake tabaksontmoediging (WHO, 2003):

> *"Het doel van dit Verdrag en de protocollen daarbij is de huidige en toekomstige generaties te beschermen tegen de verwoestende gezondheidseffecten en sociale, milieu- en economische gevolgen van tabaksconsumptie en blootstelling aan tabaksrook door een kader te bieden voor maatregelen ten behoeve van tabaksontmoediging die door de Partijen op nationaal, regionaal en internationaal niveau moeten worden uitgevoerd om het wijdverbreide tabaksgebruik en de blootstelling aan tabaksrook permanent en substantieel te verminderen."*

In oktober 2003 schrijft minister Hoogervorst in een brief aan het bestuur van Koninklijke Horeca Nederland[25]:

> *"Wij kwamen gezamenlijk tot de conclusie dat een rookvrije horeca op termijn onvermijdelijk is. Tevens gaf u aan dat het draagvlak bij uw achterban nu nog niet voldoende is om dit met ingang van 1 januari 2005 te realiseren. Vervolgens bood ik u aan om deze ingangsdatum uit de AMvB te halen op de uitdrukkelijke voorwaarde dat Koninklijk horeca Nederland (KHN) een stappenplan ontwikkelt met als einddoel een rookvrije horeca. Dit stappenplan dient voor 1 december 2003 gereed te mijn en een duidelijk tijdpad te bevatten voor het geleidelijk rookvrij worden van de horeca. Gezien de gezondheidsrisico's bij blootstelling aan tabaksrook zal het stap-*

24. TK, 1999-2000, 26472, nr.6.
25. POG/GB 2.411.992. Afspraken bestuurlijk overleg 8 september 2003. Den Haag, 3 oktober 2003.

penplan maatregelen moeten bevatten die daadwerkelijk bijdragen aan het beschermen van de gezondheid van werknemers en gasten in de horeca."

In 2004 schrijft Minster Hoogervorst in een brief aan de Tweede Kamer[26]:

"Bovenstaande sterfte- en ziektecijfers [cijfers: Gezondheidsraad, 2003] door passief roken zijn buitengewoon ernstig te noemen. Het gaat hierbij immers om jaarlijks optredende én eenvoudig vermijdbare sterfte en ziekte." (p.2)

"Bescherming van de niet-roker wordt echter helaas nog niet door iedereen als vanzelfsprekend gezien. Er zijn belangrijke terreinen, zoals (delen van) sport, kunst en cultuur en de horeca, waar maatregelen tegen blootstelling aan tabaksrook op verzet stuiten. Toch zal het ook voor deze sectoren onvermijdelijk zijn om goede maatregelen te nemen tegen blootstelling aan tabaksrook. We kunnen onze ogen immers niet meer sluiten voor de omvangrijke gezondheidsschade door meeroken. Ook werknemers en bezoekers van genoemde sectoren verdienen bescherming tegen tabaksrook." (p.3)

"De grote aantallen qua sterfte en ziektelast door passief roken die blijken uit het advies van de Gezondheidsraad vormen bovendien een aanleiding – zo niet een verplichting – voor overheidsingrijpen op die terreinen waar bescherming van de niet-roker onvoldoende gerealiseerd wordt." (p.3)

"In het advies geeft de Gezondheidsraad cijfers van de sterfte- en ziektelast door passief roken op bevolkingsniveau. De genoemde aantallen geven een indicatief beeld op basis van de tot nu toe bekende wetenschappelijke literatuur. De raad geeft aan dat nader onderzoek nodig is voor een preciezere kwantificering van de effecten in de Nederlandse populatie. Ik deel deze aanbeveling van de Gezondheidsraad." (p.4)

"De becijfering die de Gezondheidsraad in het huidige advies geeft, toont echter onomstotelijk aan dat niet gewacht kan worden met maatregelen ter bescherming van de niet-roker aangezien hiermee directe gezondheidswinst kan worden behaald. Een preciezere kwantificering zal aan deze hoofdconclusie weinig veranderen. In mijn ogen is een verdiepend onderzoek dan ook niet nodig ter onderbouwing van de beslissing om al dan niet maatregelen te nemen." (p.5)

In juni 2007 schrijft Minister Klink een brief aan de Tweede Kamer, over zijn besluit tot invoering van een rookvrije horeca zonder uitzonderingen[27]:

"Ik heb besloten om niet mee te gaan in het voorstel van KHN omdat: [1] dan niet alle werknemers (secundair: bezoekers) in de horeca beschermd worden tegen tabaksrook (ongelijke behandeling) en juist de werknemers die de hoogste blootstelling aan tabaksrook hebben (cafés, discotheken), worden uitgezonderd"

26. TK, 2003-2004, 29200 XVI, nr.215.
27. TK, 200602007, 30800 XVI, nr.149.

In juli 2007 stelt Minister Klink tijdens een algemeen overleg in de Tweede Kamer over zijn besluit om per 1 juli 2008 een algeheel wettelijk rookverbod in de horeca in te voeren[28]:

> *"De doelstelling om in 40% van de cafés rookvrije zones te creëren, is gehaald. Die zones beslaan echter vaak niet meer dan 25% van de totale oppervlakte. Iemand zit dus in een rookvrije zone terwijl zijn buurman iets verderop aan het roken is. Juist vanwege de schade die meeroken veroorzaakt voor werknemers en bezoekers, moet de horeca rookvrij gemaakt worden. Als de horeca in overwegende mate ontoegankelijk is voor mensen met luchtwegenaandoeningen, kan niet worden volgehouden dat er een absolute keuzevrijheid is. … Bescherming van de gezondheid van de werknemer en van de gasten is de reden voor een rookverbod per 1 juli 2008 in de horeca, de kunst, de cultuur en de sport."* (p.6-7)

M.i. hebben de achtereenvolgende bewindspersonen van het ministerie van VWS overtuigend aangetoond dat het noodzakelijk is om de gezondheid van werknemers in de horeca te beschermen tegen passief roken.

Sociaaleconomische schade (criterium noodzakelijkheid)

De preventie van immateriële maatschappelijke kosten als aanleiding voor rookverbonden lijkt vooral te berusten op een 'verschrijving' van minister Borst-Eilers in een brief aan de Tweede Kamer[29]:

> *"Uitgangspunt voor de regering is en blijft dat roken schade voor de volksgezondheid oplevert en hoge maatschappelijke kosten veroorzaakt. Succesvolle preventie van roken betekent dan ook een grote gezondheidswinst en veel maatschappelijk rendement."* (p.1)

In reactie op deze uitspraak volgen kritische vragen vanuit de Tweede Kamer aan minister Borst-Eilers, of de minister soms van opvatting is dat de kosten van de gezondheidszorg zullen dalen, als er minder wordt gerookt[30]:

> *"In verband met de passages in de brief over de hoge maatschappelijke kosten van het roken herinnerde zij aan de uitspraak van de minister van VWS in een overleg op 15 december jl., namelijk dat zowel voor de Verenigde Staten als voor Nederland is uitgerekend dat de kosten van de volksgezondheid aanzienlijk zouden stijgen als iedere roker morgen met roken zou stoppen. Ook het NEI-rapport komt tot die conclusie. Tegen die achtergrond vroeg mevrouw Van Blerck wat nu precies moet worden verstaan onder «hoge maatschappelijke kosten»."* (p.7)

---

28. TK, 2006-2007, 30800 XVI, nr.182.
29. TK, 1995-1996, 24743, nr.1.
30. TK, 1995-1996, 24743, nr.3.

Minister Borst redt zich eruit door te stellen dat zij doelde op immateriële maatschappelijke kosten[31]:

> *"Onder «maatschappelijke kosten» verstond de minister ook de immateriële maatschappelijke kosten, bijvoorbeeld in de gevallen waarin mensen op een jonge leeftijd ernstig ziek worden en overlijden en een jong gezin achterlaten. Voor de gezondheidszorgkosten geldt inderdaad dat deze aanzienlijk zouden stijgen als iedere roker morgen met roken zou stoppen, maar dit komt geheel anders te liggen op het moment dat longkanker en andere vormen van kanker goed behandelbaar worden. Het draaipunt is hier dat rokers nu nog vaak op jonge leeftijd overlijden aan een ziekte die vrij kort duurt en dan worden inderdaad maar weinig gezondheidszorgkosten gemaakt."* (p.20)

Als men doelt op het menselijk leed ten gevolge van ziekte, arbeidsongeschiktheid en overlijden is het duidelijker om te spreken over een aantasting van het welzijn dan over maatschappelijke kosten en maatschappelijk rendement. Wanneer men spreekt over kosten en rendement wordt snel in termen van geld gedacht. Na 1995 worden *"immateriële maatschappelijke kosten"* in het politieke debat niet meer als argument aangevoerd ter rechtvaardiging van rookverboden. Na 1995 is in het politieke debat over het rookverbod in de horeca echter ook niet expliciet aandacht voor de aantasting van het welzijn ten gevolge van passief roken. De focus in dit debat ligt op de preventie van gezondheidsschade.

Inbreuk op het persoonlijk leven (criterium noodzakelijkheid)

In overzicht 18 bevat de risicocategorie 'inbreuk op het persoonlijk leven' twee vormen van schade die aanleiding vormen tot de invoering van een rookverbod in de Horeca:

- aantasting van de lichamelijke integriteit van werknemers;
- aantasting van de keuzevrijheid van werknemers om daar te werken (solliciteren) waar zij willen. Uitsluiting van patiënten met astma of COPD van (een deel van) de arbeidsmarkt ten gevolge van passief roken.

Aantasting van lichamelijke integriteit

De aantasting van de lichamelijke integriteit weegt het zwaarst en hiernaar wordt ook door de rechter verwezen[32]:

> *"In dit verband heeft de rechter te Breda op 25 april 2000 uitgesproken dat niet-rokers ten principale recht hebben op een rookvrije werkplek. Het recht op bescherming van de lichamelijke integriteit, de gezondheid, is een grondrecht." (p.34)*

31. TK, 1995-1996, 24743, nr.3.
32. TK, 1999-2000, 26472, nr.6. [14]

Dat een onvrijwillige aantasting van de lichamelijke integriteit ten gevolge van passief roken onaanvaardbaar is en dat werknemers hiertegen dienen te worden beschermd, staat in het politieke debat momenteel niet ter discussie.

Uitsluiting van werknemers van de arbeidsmarkt
In de door mij bestudeerde documenten ben ik dit argument niet tegengekomen ter rechtvaardiging van het wettelijk rookverbod in de horeca. In het politieke debat is dit argument gehanteerd ter rechtvaardiging van de invoering van het wettelijk recht op een rookvrije werkplek voor werknemers per 1 januari 2004 door minister Borst-Eilers. Van 2004 tot 2008 werd de sector horeca uitgezonderd van dit wettelijk recht op een rookvrije werkplek. Als reden voor deze uitzondering werd niet aangevoerd dat een rookvrije werkplek voor werknemers in de horeca minder noodzakelijk was dan voor werknemers buiten de horeca. De reden die werd aangevoerd was het ontbreken van voldoende draagvlak voor (de handhaving van) een wettelijk rookverbod in de horeca. Daarom werd de horeca in de gelegenheid gesteld om via zelfregulering in enkele jaren naar een rookvrije horeca toe te groeien. In 2007 heeft minister Klink besloten om een wettelijk rookverbod in de horeca in te voeren. Hij heeft hieraan uitvoering gegeven door de horeca vanaf 1 juli 2008 niet langer uit te zonderen van het wettelijk recht op een rookvrije werkplek voor werknemers dat in 2004 door minister Borst-Eilers was ingevoerd. Daarom kan men redeneren dat de rechtvaardiging, die ten grondslag ligt aan de invoering van het wettelijk recht op een rookvrije werkplek in 2004, ook van toepassing is op het wettelijk rookverbod in de horeca, dat sinds juli 2008 van kracht is. De noodzaak van een rookvrije werkplek ter bescherming van werknemers in de horeca wordt door de achtereenvolgende ministers van VWS – Hoogervorst, Klink en Schippers – onderschreven.

Hinder en overlast (criterium noodzakelijkheid)
Naarmate de wetenschappelijke evidence over de schadelijkheid van passief roken voor de gezondheid van werknemers en bezoekers in de horeca groter werd, is het argument van hinder en overlast van passief roken voor werknemers en bezoekers in de horeca steeds meer op de achtergrond geraakt in het politieke debat. Momenteel wordt in het politieke debat niet of nauwelijks over hinder en overlast van passief roken gesproken.

Is de preventiemaatregel moralistisch?
Het rookverbod in de horeca is niet moralistisch, want het dient ter bestrijding van gezondheidsschade aan derden. Het doel is dus schadepreventie en niet het opdringen van een gezonde leefstijl.

Is de preventiemaatregel perfectionistisch?
Het rookverbod in de horeca is niet perfectionistisch, want het dient ter bestrijding van ernstige gezondheidsschade. Bovendien worden er geen overdreven hoge eisen aan de bescherming van werknemers tegen passief roken gesteld. Rookruimten worden toegestaan en zelfs roken zal worden toegestaan, indien ventilatiesystemen werknemers voldoende tegen passief roken kunnen beschermen.

Geven sancties een indicatie van de noodzakelijkheid?
Het rookverbod in de horeca wordt gehandhaafd door de Voedsel en Waren Autoriteit (VWA). Bij overtreding van de regelgeving zal de VWA eerst een waarschuwing uitdelen en de ondernemer de gelegenheid geven het beleid op orde te maken. Als bij hercontrole nog steeds sprake is van overtreding, kan de VWA een boete opleggen. De boetes kunnen hoog oplopen. In december 2008 schrijft minister Klink aan de Tweede Kamer[33]:

> *"Op 17 en 26 november 2008 heb ik u gemeld dat de VWA in nauwe samenwerking met het Openbaar Ministerie (OM) gebruik zal gaan maken van de Wet op de Economische Delicten (WED). De controleurs zullen in ieder geval proces-verbaal opmaken in die gevallen waarin horecaondernemers reeds tweemaal of vaker een bestuurlijke boete is aangezegd. Deze zaken zullen zo spoedig mogelijk voor de economische politierechter worden gebracht. De officier van justitie kan een boete eisen van maximaal € 18.500,-, al dan niet in combinatie met een voorwaardelijke stillegging. Voor deze aanpak is gekozen omdat er in delen van de horeca sprake lijkt van een min of meer gecoördineerde actie om het rookverbod te overtreden. In een rechtstaat is niet te verdedigen dat democratisch vastgestelde wetten bewust worden overtreden. … Tijdens het spoed algemeen overleg op 27 november 2008 bleek dat uw Kamer deze aanpak in meerderheid steunt."* (p.4)

Hieruit blijkt dat de overheid (de handhaving van) het rookverbod serieus neemt.

In januari 2011 stelt minister Schippers van VWS in een overleg met de Tweede Kamer echter[34]:

> *"Minister Schippers: … Ook wordt er gevraagd om handhaving door hogere boetes te garanderen. Ik heb aangekondigd dat ik met fors hogere boetes kom. Als wij een wet maken, moeten wij die handhaven. Een boete van € 300 is dan volstrekt een lachertje. Ik zal mij er dus voor inzetten dat de boetes fors worden verhoogd." (p.5)*

Het bovenstaande citaat duidt erop dat minister Schippers handhaving van het wettelijk rookverbod in de horeca noodzakelijk acht.

---

33. TK, vergaderjaar 2008–2009, 22894, nr.206.
34. TK, 2010-2011, vergaderingnr.44, itemnr.6 (dossiernr.32011).

11.2.3 Eindoordeel (criterium noodzakelijkheid)

Overzicht 19: is de noodzaak van preventie aangetoond?

Risico's die de overheid met het wettelijk rookverbod tracht te bestrijden	*Noodzaak van preventie aangetoond?*
**Gezondheidsschade**<br>a. Risico op gezondheidsschade bij werknemers ten gevolge van passief roken	**Gezondheidsschade**<br>a. Noodzaak is aangetoond.
**Sociaaleconomische schade**<br>b. Risico op immateriële maatschappelijke kosten voor werknemers ten gevolge van passief roken (leed door ziekte, arbeidsongeschiktheid of voortijdig overlijden)	**Sociaaleconomische schade**<br>b. Argument heeft nauwelijks een rol gespeeld in het politieke debat.
**Inbreuk op het persoonlijk leven**<br>c. Aantasting van de lichamelijke integriteit van werknemers.<br>d. Aantasting van de keuzevrijheid van werknemers om daar te werken (solliciteren) waar zij willen. Uitsluiting van patiënten met astma of COPD van (een deel van) de arbeidsmarkt ten gevolge van passief roken.	**Inbreuk op het persoonlijk leven**<br>c. Noodzaak is aangetoond.<br>d. Noodzaak is aangetoond.
**Hinder of overlast**<br>e. Hinder en overlast voor werknemers ten gevolge van passief roken (benauwdheid hoofdpijn, duizeligheid, irritatie, verminderd smaakvermogen, stank)	**Hinder of overlast**<br>e. Noodzaak is aangetoond.

In het politieke debat ligt de focus op de preventie van gezondheidsschade. De beoordeling van de gerechtvaardigdheid van de preventiemaatregel zal ik vanaf het criterium 'oorzakelijkheid' (3e criterium van het model) niet meer baseren op de preventie van sociaaleconomische schade, omdat dit argument nauwelijks een rol heeft gespeeld in het politieke debat.

11.2.4 Gevolgen van de beoordeling voor het model (criterium noodzakelijkheid)

De beoordeling van het wettelijk rookverbod in de horeca aan de hand van het criterium 'noodzakelijkheid' heeft voor het model geen nieuwe inzichten opgeleverd.

11.3 Derde criterium: oorzakelijkheid

11.3.1 Wijze van beoordeling

Het criterium 'oorzakelijkheid' betreft de vraag naar de determinanten van de risico's die men met de preventiemaatregel tracht te bestrijden. Achtereenvolgens dienen twee vragen te worden beantwoord:

1. In hoeverre zijn de leefstijlen waarop de preventiemaatregel zich richt bepalend voor de risico's die men tracht te bestrijden (zie criterium 'schadelijkheid')?
2. Welke factoren zijn bepalend voor de leefstijlen waarop de preventiemaatregel zich richt en in hoeverre zijn deze factoren bepalend?

Beoordeeld wordt of de beschikbare informatie een duidelijk beeld geeft van de determinanten van 'de risico's die men tracht te bestrijden' en hun onderlinge wisselwerking. Als de beschikbare informatie geen duidelijk beeld geeft, wordt beoordeeld of de informatieverzameling over de determinanten voldoende is geweest.

11.3.2 Beoordeling van casus 'wettelijk rookverbod in horeca' aan de hand van criterium 'oorzakelijkheid'

Vraag 1: In hoeverre zijn de leefstijlen waarop de preventiemaatregel zich richt bepalend voor de risico's die men tracht te bestrijden?

De preventiemaatregel is gericht op het voorkomen van de blootstelling van werknemers in de horeca aan passief roken. Roken in afgesloten ruimten leidt tot de blootstelling van niet-rokers in dezelfde ruimte aan tabaksrook; het zogenaamde passief roken. Het oorzakelijk verband tussen roken in afgesloten ruimten en passief roken is overduidelijk en wetenschappelijk bewezen, en wordt in het politieke debat niet ter discussie gesteld. Het oorzakelijk verband tussen passief roken en risico's op gezondheidsschade bij werknemers in de horeca is wetenschappelijk bewezen en wordt in het politieke debat niet (meer) ter discussie gesteld. Zelfs een kleine dosis tabaksrook die men via passief roken inademt, kan leiden tot gezondheidsschade (met name hart- en vaatziekten). Er heeft zeer veel wetenschappelijk onderzoek plaatsgevonden om de bovengenoemde oorzakelijke verbanden aan te tonen. De overheid heeft bij de onderbouwing van het rookverbod in de horeca goed van dit wetenschappelijk onderzoek gebruikt gemaakt. Het oorzakelijk verband tussen roken in afgesloten ruimten en hinder of overlast voor niet-rokers is evident.

Vraag 2: Welke factoren zijn bepalend voor de leefstijlen waarop de preventiemaatregel zich richt en in hoeverre zijn deze factoren bepalend?

Het roken van werknemers en van bezoekers in de horeca leidt tot blootstelling van niet-rokende werknemers in de horeca aan passief roken. Waarom roken werknemers en waarom roken bezoekers in de horeca? En welke factoren zijn medebepalend voor het roken in de horeca? Deze vragen zijn voorafgaand aan de invoering van het rookverbod in de horeca in 2007 eigenlijk nauwelijks onderzocht. Was dat wel gebeurd, dan had de overheid wellicht beter kunnen anticiperen op het grootschalige verzet tegen het rookverbod in zogenaamde 'huiskamerkroegjes'. Ik vermoed dat dergelijk kleine buurtkroegjes vooral worden bezocht door mensen met gemiddeld genomen een lagere sociaaleconomische status (SES). Het zou inderdaad zo kunnen zijn dat de buurtkroeg voor deze mensen functioneert als een verlengstuk van hun huiskamer, terwijl dat minder het geval is voor het kroegbezoek in het kader van het 'uitgaansleven'. Minister Schippers stelt in dit verband[35]:

> *"… wordt aldus bewerkstelligd dat in (zeer) kleine buurtkroegjes, waar de kroeg een sociale functie vervult vergelijkbaar met die van de huiskamer in een huisgezin, roken wel mogelijk wordt gemaakt. Met het terugtreden van de regulering voor deze ruimten versterkt de wetgever de gedachte dat roken in de huiskamer niet een aangelegenheid is waar de overheid zich mee bemoeit."* (p.5)

Voor zover mij bekend, is deze stellingname van minister Schippers niet op wetenschappelijk onderzoek gebaseerd.

11.3.3 Eindoordeel (criterium oorzakelijkheid)

De preventiemaatregel is gericht op het voorkomen van de blootstelling van werknemers in de horeca aan passief roken. Het oorzakelijk verband tussen roken in afgesloten ruimten en passief roken is uitgebreid onderzocht en overtuigend bewezen. Dat geldt ook voor het oorzakelijk verband tussen passief roken en risico op gezondheidsschade bij werknemers in de horeca. Het oorzakelijk verband tussen roken in afgesloten ruimten en hinder of overlast voor niet-rokers is evident.

Naar de factoren die mede bepalend zijn voor het roken in de horeca heeft onvoldoende onderzoek plaatsgevonden. Bij de invoering van het rookverbod in de horeca is aan deze factoren te weinig aandacht besteed.

35. Bijlage bij Tweede Kamerstuk 32011 nr.9, vergaderjaar 2010–2011.

11.3.4 Gevolgen van de beoordeling voor het model (criterium oorzakelijkheid)

De beoordeling van het wettelijk rookverbod in de horeca aan de hand van het criterium ‘oorzakelijkheid’ heeft voor het model geen nieuwe inzichten opgeleverd.

11.4 Vierde criterium: verantwoordelijkheid

11.4.1 Operationalisering en wijze van beoordeling

Het criterium ‘verantwoordelijkheid’ betreft de vragen:
1. Welke partijen worden verantwoordelijk gehouden voor het ontstaan en/of de instandhouding van de risico’s die men tracht te bestrijden?
2. In hoeverre is bij het vaststellen van de verantwoordelijke partijen rekening gehouden met de determinanten van de risico’s die men tracht te bestrijden?
3. In hoeverre is de preventiemaatregel paternalistisch?

Beoordeeld wordt of:
- duidelijk is welke partijen verantwoordelijk worden gehouden?
- bij het vaststellen van de verantwoordelijke partijen voldoende rekening is gehouden met alle bekende determinanten van de risico’s die men tracht te bestrijden?
- de preventiemaatregel niet ongewenst paternalistisch is?

11.4.2 Beoordeling van casus ‘wettelijk rookverbod in horeca’ aan de hand van criterium ‘verantwoordelijkheid’

Vraag 1: Welke partijen worden verantwoordelijk gehouden voor het ontstaan en/of de instandhouding van de risico’s die men tracht te bestrijden?
Alle rokers – werkgevers, werknemers en bezoekers – in de horeca worden verantwoordelijk gehouden voor het ontstaan en de instandhouding van de risico’s op schade, hinder en overlast ten gevolge van passief roken. Werknemers in de horeca, die op hun werkplek worden blootgesteld aan passief roken, worden hiervoor op geen enkele manier verantwoordelijk gehouden. Er wordt dus niet gesteld dat zij maar een andere baan moeten zoeken, als zij op hun werkplek niet (incidenteel) aan passief roken willen worden blootgesteld.

Bezoekers die in de horeca worden blootgesteld aan passief roken, worden wel mede verantwoordelijk gehouden voor de risico’s op schade, hinder en overlast

ten gevolge van passief roken, die zij lopen. Minister Schippers stelt, dat het voldoende is als zij kunnen kiezen tussen horeca-inrichtingen, die al dan niet rookvrij zijn. De bescherming van bezoekers in de horeca tegen passief roken is volgens de ministers Klink en Schippers geen doelstelling van het wettelijk rookverbod in de horeca.

In 2000 schrijft minister Borst-Eilers aan de Tweede Kamer ter rechtvaardiging van de rookverboden die zij voorstelt[36]:

> *"Hinder, overlast en gezondheidsschade. De leden van de fractie van de PvdA overwegen dat een volwassen roker in beginsel ooit zelf de keuze gemaakt heeft om te gaan roken, onder welke invloed dan ook. Zij menen dat hij of zij daardoor, afhankelijk van de kennis over de schadelijkheid van roken, een zekere eigen verantwoordelijkheid heeft, hoe klein of groot dan ook, voor de gezondheidsschade die daarvan het gevolg is. Dat geldt volgens de PvdA-fractie daarentegen niet voor diegenen, de niet-rokers, die onvrijwillig worden blootgesteld aan tabaksrook. … Ik ben het eens met deze redenering, maar wil hier graag een paar opmerkingen aan toevoegen, dit ter aanscherping van het betoog. De eerste opmerking is dat bijna alle volwassen rokers op minderjarige leeftijd zijn begonnen, vaak al op (zeer) jonge leeftijd; de vraag rijst dan ook of zij toentertijd volledig in staat waren te komen tot een weloverwogen rationele keuze. Ten tweede is het zo dat tabak een uitermate verslavende stof is; rokers zijn dus veelal als minderjarige verslaafd geraakt. Overigens onderschrijf ik geheel de opvatting van deze leden dat niet-rokers dikwijls gedwongen zijn om onvrijwillig tabaksrook in te ademen, bijvoorbeeld op het werk of in de horeca."*

In het bovenstaande citaat nuanceert minister Borst-Eilers de eigen verantwoordelijkheid die rokers hebben voor de gezondheidsschade die zijzelf ondervinden ten gevolge van hun rookgedrag. In dit citaat nuanceert zij echter niet de verantwoordelijkheid die rokers hebben om hinder, overlast en gezondheidsschade ten gevolge van passief roken bij niet-rokers te voorkomen (hierover spreekt zij zich niet uit).

Vraag 2: In hoeverre is bij het vaststellen van de verantwoordelijke partijen rekening gehouden met de determinanten van de risico's die men tracht te bestrijden?

De focus in het politieke debat ligt op het bewijzen van de schadelijkheid van passief roken. Over de determinanten van het 'roken in de horeca' is in het politieke debat niet of nauwelijks gesproken.

36. TK, 1999–2000, 26472, nr.6.

Wel wordt in het politieke debat gewezen op het verslavende effect van roken. Verslaving geeft drang tot roken en die drang houdt niet op als men een horecagelegenheid betreedt. Misschien neemt die drang zelfs toe, bijvoorbeeld na het nuttigen van een warme maaltijd en/of alcoholische drank. Aan de relatie tussen 'rookverslaving – omgevingsfactoren – drang tot roken' wordt in het politieke debat echter geen aandacht besteed.

Vraag 3: In hoeverre is de preventiemaatregel paternalistisch?

Alleen een rookverbod biedt werknemers in de horeca adequate bescherming tegen passief roken op hun werkplek. De reden is dat zelfs hele kleine doses tabaksrook schadelijk kunnen zijn voor de gezondheid en dat ventilatiesystemen onvoldoende bescherming bieden (EC, 2007). Men zou echter kunnen redeneren dat werkgevers en werknemers dit onderling wel kunnen regelen en dat het paternalistisch is als de overheid dit wettelijk regelt. Er zijn echter goede argumenten om hier niet vanuit te gaan.

In 2000 schrijft minister Borst-Eilers aan de Tweede Kamer ter rechtvaardiging van de rookverboden die zij voorstelt[37]:

> *"De regering deelt niet de inschatting van deze leden [Groen Links] dat de huidige rokers deze maatregelen als een inbreuk op hun vrijheid zullen ervaren. Immers, een algemeen aanvaard principe is toch dat de vrijheid van de een (van de roker om te roken) eindigt waar die van de ander (van de niet-roker met het recht op schone lucht) begint. Wel moet worden erkend dat het alle rokers niet altijd lukt dit principe overal in de praktijk te brengen, want rokers zijn nu eenmaal verslaafd aan tabak."* (p.4)

> *"Ik wil hier nog aan toevoegen dat er bij passief roken sprake is van (potentiële) schade en hinder of overlast voor derden; ook dat is een algemene grond voor overheidsinterventie."* (p.4)

> *"Het is goed om eveneens te kunnen vaststellen dat deze leden [Groen Links] het toejuichen om werkgevers, vervoersexploitanten, luchtvaartmaatschappijen en beheerders van openbare instellingen stringenter verantwoordelijk te maken voor het niet roken, omdat hierdoor de niet-roker door de verantwoordelijke instantie wordt beschermd en niet meer in de huidige moeilijke situatie wordt gebracht om bij collega's, medebezoekers of personeel te moeten vragen om gevrijwaard te worden van ongezonde tabaksrook."* (p.4)

37. TK, 1999–2000, 26472, nr.6.

> *"De onvoldoende maatschappelijke acceptatie van het recht op schone lucht voor niet-rokers en de in te geringe mate aanwezige tolerantie bij rokers blijkt in de dagelijkse praktijk niet voldoende ruimte te bieden voor een daadwerkelijk volwaardige bescherming van de meerderheid van niet-rokers in ons land. Daarom is het onvermijdelijk om wettelijke regels te gaan stellen voor die plaatsen waar een ieder vrij moeten kunnen komen of vertoeven zonder hinder van andermans rook. … De rookverboden zullen verder moeten worden uitgebreid en aangescherpt."* (p.35)

In 2011 stelt minister Schippers ter rechtvaardiging van het rookverbod in de horeca[38]:

> *"Het uitgangspunt van de wet is het beschermen van de werknemers tegen tabaksrook. Waarom? Omdat sprake is van een hiërarchische relatie. De werknemer kan wel denken «ik heb het eigenlijk liever niet», maar durft dat misschien niet te zeggen. Dan staat hij in de rook. Dat is dus het uitgangspunt."* (p.29)

Uit de bovenstaande citaten kunnen diverse argumenten worden gedistilleerd waarom de wettelijke bescherming van werknemers (in de horeca) tegen passief roken op de werkplek niet paternalistisch is:
- rokers zijn verslaafd aan tabak; als de werkgever zelf rookt en/of de meeste werknemers roken, is de kans reëel dat de werkgever een verzoek van een werknemer om een rookverbod in te stellen, niet zal inwilligen;
- bij passief roken is er sprake voor hinder en overlast voor derden;
- het recht op schone lucht voor niet-rokers is nog onvoldoende maatschappelijk geaccepteerd;
- rokers zijn in te geringe mate tolerant tegenover niet-rokers;
- het kan moeilijk zijn voor werknemers om bij collega's, medebezoekers of personeel te moeten vragen om gevrijwaard te worden van tabaksrook, met name omdat er sprake is van een hiërarchische relatie tussen werkgever en werknemers (en werknemers onderling).

Bovendien verwachten veel werkgevers in de horeca dat een rookverbod in hun zaak zal leiden tot omzetverlies en sommigen verwachten zelfs dat zij hierdoor failliet kunnen gaan. Dit maakt voor werknemers de drempel hoger om hun werkgever om een rookverbod te verzoeken en voor werkgevers de drempel hoger om een rookverbod in te stellen.

Samen maken deze argumenten het aannemelijk dat men de bescherming van werknemers in de horeca tegen passief roken niet aan werknemers en werkgevers

38. TK, 2010–2011, 32011, nr.15.

kan overlaten, c.q. dat het niet ongewenst paternalistisch is, als de overheid een wettelijk rookverbod in de horeca instelt.

11.4.3 Eindoordeel (criterium verantwoordelijkheid)

Het is duidelijk welke partijen verantwoordelijk worden gehouden voor het risico op schade, hinder en overlast voor werknemers ten gevolge van passief roken. Alle rokers in de horeca – werkgevers, werknemers en bezoekers – worden verantwoordelijk gehouden voor dit risico. Bij het toewijzen van de verantwoordelijkheid hiervoor heeft de overheid geen rekening gehouden met de determinanten van het rookgedrag dat wordt verboden. Omdat de preventiemaatregel het roken niet volledig verbiedt, maar alleen in de horeca, is het niet onredelijk dat alle verantwoordelijkheid voor het risico op schade, hinder en overlast bij de rokers wordt gelegd. Buiten de horeca kunnen rokers 'gewoon' aan hun verslaving toegeven. Er zijn goede argumenten om het wettelijk rookverbod in de horeca niet paternalistisch te noemen.

11.4.4 Gevolgen van de beoordeling voor het model (criterium verantwoordelijkheid)

De beoordeling van het wettelijk rookverbod in de horeca aan de hand van het criterium 'verantwoordelijkheid' heeft voor het model geen nieuwe inzichten opgeleverd.

11.5 Vijfde criterium: gerichtheid

11.5.1 Operationalisering en wijze van beoordeling

Het criterium 'gerichtheid' betreft de vragen:

1. Is de keuze van de doelgroep en leefstijlbeïnvloedende factoren logisch, gezien de determinanten van de risico's die men tracht te bestrijden en de verantwoordelijkheid voor deze risico's?
2. In hoeverre worden personen binnen de doelgroep door de interventie gemist?
3. In hoeverre worden personen buiten de doelgroep door de interventie geraakt?

Beoordeeld wordt of:

- voor de doelgroep- en leefstijlbeïnvloedende factoren geen logischer alternatieven mogelijk zijn;

– onevenredig of onnodig veel personen binnen de doelgroep door de interventie worden gemist;
– onevenredig of onnodig veel personen buiten de doelgroep door de interventie worden geraakt.

11.5.2 *Beoordeling van casus 'wettelijk rookverbod in horeca' aan de hand van criterium 'gerichtheid'*

Vraag 1: Is de keuze van de doelgroep en leefstijlbeïnvloedende factoren logisch?
Het doel van het wettelijk rookverbod in de horeca is de bescherming van werknemers op hun werkplek in de horeca tegen blootstelling aan tabaksrook. De doelgroep van de preventiemaatregel wordt daarom gevormd door rokers in horecagelegenheden met werknemers (getracht wordt om hun leefstijl c.q. rookgedrag te beïnvloeden). Om dit te bewerkstelligen wordt een algeheel rookverbod ingevoerd, dat zich richt op alle mogelijke rokers in de horeca: werkgevers, werknemers en bezoekers. Gezien de doelstelling van het wettelijk rookverbod, is deze keuze logisch. Gezien de doelstelling van het wettelijk rookverbod is het echter niet logisch om het wettelijk rookverbod ook in te voeren in horecagelegenheden zonder werknemers. Als reden om het wettelijk rookverbod ook in horecagelegenheden zonder werknemers in te voeren, noemt minister Klink het belang van een gelijk speelveld voor horecaondernemingen met en zonder werknemers, maar ook duidelijkheid voor bezoekers en het voorkomen van definitie- en afbakeningskwesties (is bijvoorbeeld de partner van de kroegeigenaar, als de partner meehelpt in de kroeg, een werknemer van de kroegeigenaar?)[39]:

> *"Artikel 11a van de Tabakswet heeft alleen betrekking op werkgevers, maar lid 4 van datzelfde artikel biedt de mogelijkheid om categorieën van voor publiek toegankelijke gebouwen aan te wijzen waar een rookverbod wordt ingesteld. Er moet een gelijk speelveld komen voor alle horecaondernemingen. ...*
>
> *Per 1 juli 2008 geldt een rookverbod voor de hele sector. Dat biedt duidelijkheid voor iedereen. Er is geen sprake van fasering voor de natte horeca en de restaurants en er zijn geen definitie- en afbakeningskwesties meer."* (p.7)

Vraag 2: In hoeverre worden personen binnen de doelgroep door de interventie gemist?
De doelgroep van de preventiemaatregel wordt gevormd door rokers in horecagelegenheden met werknemers. Per 1 januari 2008 heeft minister Klink een algeheel

39. TK, 2006–2007, 30800 XVI, nr.182.

wettelijk rookverbod in de horeca ingevoerd. Met een algeheel rookverbod in de horeca wordt niemand uit de doelgroep door de preventiemaatregel gemist.

In november 2010 heeft minister Schippers een brief aan de Tweede Kamer geschreven over haar voornemen tot opheffing van het wettelijke rookverbod voor kleine cafés zonder personeel. Ook in geval van opheffing van het wettelijke rookverbod voor kleine cafés zonder personeel wordt niemand uit de doelgroep door de preventiemaatregel gemist.

Vraag 3: In hoeverre worden personen buiten de doelgroep door de interventie geraakt?

Ook niet-rokende werkgevers en werknemers, die geacht worden de preventiemaatregel te handhaven, kunnen door de preventiemaatregel worden geraakt. Zij kunnen bijvoorbeeld worden geconfronteerd met boze gasten en met omzetverlies. Vraag 3 van het criterium 'gerichtheid' van het model dient er echter toe om vast te stellen of de preventiemaatregel ook ingrijpt op de leefstijl (in dit geval het rookgedrag) van mensen buiten de doelgroep, en niet om vast te stellen of mensen buiten de doelgroep op andere wijze door de preventiemaatregel worden geraakt. Daarom ga ik ertoe over om vraag 3 nog iets preciezer te formuleren: *'In hoeverre wordt de leefstijl van personen binnen de doelgroep door de interventie gemist?'*. Analoog aan deze precisering komt vraag 2 dan te luiden: *'In hoeverre wordt de leefstijl van personen buiten de doelgroep door de interventie geraakt?'*.

Het algeheel rookverbod dat minister Klink heeft ingevoerd, raakt ook de leefstijl van personen die niet tot de doelgroep van de preventiemaatregel behoren. Het wettelijke rookverbod geldt namelijk ook voor bezoekers en eigenaars van horecagelegenheden zonder personeel, terwijl zij misschien zouden willen roken. Minister Schippers komt deze mensen tegemoet door voor te stellen om kleine cafés zonder personeel uit te zonderen van het wettelijke rookverbod in de horeca. Opmerkelijk is dat zij deze uitzondering beperkt tot kleine cafés zonder personeel en niet uitbreidt tot bijvoorbeeld snackbars en ijssalons. Als enige reden voor deze beperking geeft zij aan dat het aantal grensgevallen zo beperkt blijft[40]:

> *"De regels voor de andere sectoren in de horeca, zoals restaurants, grotere cafés, discotheken, fastfoodsector, evenementensector en de sport blijven onverminderd van kracht. Door deze scherpe afbakening van de uitzondering blijft het aantal grensgevallen beperkt."* (p.2)

40. TK, 2010–2011, 22894, nr.289.

Het voorkomen van definitie- en afbakeningskwesties was ook een van de redenen van minister Klink om een algeheel wettelijk rookverbod in te voeren (zie onder vraag 1 van het criterium 'gerichtheid').

11.5.3 *Eindoordeel (criterium gerichtheid)*

Vraag 1: Is de keuze van de doelgroep en leefstijlbeïnvloedende factoren logisch?
Met inachtneming van de doelstelling van de preventiemaatregel (bescherming van werknemers in de horeca tegen passief roken) zijn er m.i. geen logischer alternatieven mogelijk voor de keuze van de doelgroep en leefstijlbeïnvloedende factoren. Dat geldt zowel voor het algeheel wettelijk rookverbod dat minister Klink per 1 januari 2008 heeft ingevoerd, als voor de versoepeling van het wettelijk rookverbod voor kleine cafés zonder personeel, die minister Schippers in 2010 heeft voorgesteld. Klink acht het voor de handhaafbaarheid en uitvoerbaarheid van het wettelijk rookverbod in de horeca van belang dat de kleine cafés zonder personeel er ook onder vallen. Schippers acht het voor het draagvlak voor en daarmee de handhaafbaarheid van het wettelijk rookverbod in de horeca van belang dat de kleine cafés zonder personeel er niet onder vallen. Beide ministers stellen dat zij gelijk hebben, maar de wetenschappelijke onderbouwing van beide stellingnamen is zwak.

Vraag 2: In hoeverre worden personen binnen de doelgroep door de interventie gemist?
Met het algeheel wettelijk rookverbod in de horeca, dat door minister Klink is ingevoerd, wordt niemand uit de doelgroep door de preventiemaatregel gemist. Ook in geval van opheffing van het wettelijke rookverbod voor kleine cafés zonder personeel, zoals minister Schippers voorstelt, wordt niemand uit de doelgroep door de preventiemaatregel gemist.

Vraag 3: In hoeverre worden personen buiten de doelgroep door de interventie geraakt?
Volgens minister Klink leidt een algeheel wettelijk rookverbod in de horeca tot een betere handhaafbaarheid en uitvoerbaarheid van dit rookverbod. Volgens minister Schippers leidt de uitzondering van kleine cafés zonder personeel van het wettelijk rookverbod in de horeca tot meer draagvlak voor en daarmee een betere handhaafbaarheid van dit rookverbod. Als minister Schippers gelijk heeft, raakt een algeheel wettelijk rookverbod in de horeca onnodig veel personen buiten de doelgroep. Als de handhaving van het wettelijk rookverbod, in de horeca met personeel, echter substantieel verslechtert door de uitzondering die minister Schippers voorstelt, kan niet worden volgehouden dat een algeheel wettelijk rookverbod de leefstijl van onnodig veel personen buiten de doelgroep raakt. Blijkbaar lukt het dan alleen om

tot adequate bescherming van werknemers in de horeca tegen passief roken te komen middels een algeheel wettelijk rookverbod. Zowel minister Klink als minister Schippers beschikten niet over wetenschappelijk onderzoek dat hun gelijk aantoont. Het antwoord op vraag 3 is dus onduidelijk.

11.5.4 Gevolgen van de beoordeling voor het model (criterium gerichtheid)

De beoordeling van het wettelijk rookverbod in de horeca aan de hand van het criterium 'gerichtheid' van het model leidt tot een kleine aanpassing van de vragen waarmee ik het criterium 'gerichtheid' operationaliseer en beoordeel. Aan de 2e en 3e vraag wordt het woord 'leefstijl' toegevoegd:
1. In hoeverre wordt de leefstijl van personen binnen de doelgroep door de interventie gemist?
2. In hoeverre wordt de leefstijl van personen buiten de doelgroep door de interventie geraakt?

11.6 Zesde criterium: geschiktheid

11.6.1 Operationalisering en wijze van beoordeling

Het criterium 'geschiktheid' betreft de geschiktheid van de partij die de preventiemaatregel uitvoert. Het gaat hierbij om de volgende vragen:
1. Is de keuze van de partij die de preventiemaatregel uitvoert logisch, gezien de doelgroep en leefstijlbeïnvloedende factoren waarop de maatregel zich richt?
2. Is de partij die de preventiemaatregel uitvoert daartoe bekwaam en bevoegd?
3. Is de partij die de preventiemaatregel uitvoert daartoe geschikt?
4. Brengt uitvoering van de preventiemaatregel schade toe aan de aard of het imago van de partij die de maatregel uitvoert?

Beoordeeld wordt of:
- uitvoering van de preventiemaatregel door een andere partij niet logischer is;
- de partij die de preventiemaatregel uitvoert daartoe niet onbekwaam of onbevoegd is;
- de partij die de preventiemaatregel uitvoert daartoe niet ongeschikt is;
- uitvoering van de preventiemaatregel geen onaanvaardbare schade toebrengt aan de aard of het imago van de partij die de maatregel uitvoert.

11.6.2 Beoordeling van casus 'wettelijk rookverbod in horeca' aan de hand van criterium 'geschiktheid'

Bij het rookverbod in de horeca zijn verschillende uitvoerende partijen betrokken:
- de overheid, die de wetgeving invoert;
- de werkgevers (en werknemers), die geacht worden het rookverbod te handhaven, en
- de Voedsel en Waren Autoriteit (VWA), die geacht wordt de naleving van het rookverbod te controleren en zo nodig boetes op te leggen aan werkgevers.

In de vragen behorend bij het criterium 'geschiktheid' vervang ik het enkelvoud 'partij' daarom door het meervoud 'partijen'. Ik zal de geschiktheid van deze uitvoerende partijen achtereenvolgens bespreken.

Vraag 1: Is de keuze van de uitvoerende partijen logisch, gezien de doelgroep en leefstijlbeïnvloedende factoren?

Invoering van wetgeving door de overheid

Minister Hoogervorst is begonnen met stringente regulering van het rookbeleid in de horeca door hierover in de vorm van resultaatsverplichtingen kwantitatieve afspraken met Koninklijke Horeca Nederland (KHN) te maken. Zijn opvolger, minister Klink, heeft besloten om van zelfregulering door de horecasector over te gaan op verbodswetgeving, omdat de resultaten van de zelfregulering naar zijn mening in kwantitatieve zin wel, maar in kwalitatieve zin niet, voldoende waren. Minister Klink stelde in dit verband[41]:

> *"Resultaten tweede jaar zelfregulering (2006). … De kwantitatieve doelen zijn – behoudens in enkele sectoren – gehaald, maar de resultaten zijn in kwalitatieve zin minder positief. Zeker de geringe omvang van de rookvrije delen en de – in bepaalde sectoren – weinig consequente toepassing maken duidelijk dat de rookvrije horeca en bescherming van de werknemer via zelfregulering niet bereikt zullen worden." (p.3)*

De vraag waar het om draait, is of het terecht is dat de rijksoverheid heeft besloten de regie over het rookbeleid in de horeca van KHN over te nemen door over te gaan van zelfregulering op wetgeving. Deze vraag kan vanuit inhoudelijk en procedureel perspectief worden beoordeeld. Op basis van de in kwalitatieve zin teleurstellende resultaten van de zelfregulering, denk ik dat minister Klink inhoudelijk gezien gelijk had dat de rookvrije horeca en de bescherming van de werknemers

41. TK, 2006-2007, 30800 XVI, nr.149.

tegen passief roken, via zelfregulering niet bereikt zouden worden of dat dit onaanvaardbaar lang zou duren.

In april 2005 schrijft minister Hoogervorst aan de Tweede Kamer dat hij in het kader van de zelfregulering van het rookbeleid in de horeca de volgende afspraken met KHN heeft gemaakt[42]:

> *"Ik ben met Koninklijk Horeca Nederland (KHN) overeengekomen dat de monitoring van het stappenplan gezamenlijk zal worden uitgevoerd. Daartoe zal gezamenlijk een onafhankelijk onderzoeksbureau worden geselecteerd dat in overleg met KHN en VWS de onderzoeksopzet en onderzoeksmethode verder zal vormgeven."* (p.1)

> *"Het stappenplan beslaat een periode van vier jaar (2005, 2006, 2007 en 2008) en bevat voor elk jaar concrete streefcijfers. Na afloop van elk jaar zal de voortgang van de invoering van de afgesproken maatregelen worden gemeten. Er zal dus over elk jaar een aparte voortgangsrapportage verschijnen waarin wordt teruggekeken op het voorgaande jaar of de voorgaande jaren. Aan het eind van de totale periode zal een eindevaluatie worden opgesteld."* (p.1)

> *"De planning ziet er nu als volgt uit.*
>
> - *Eerste meting (over 2005) in het eerste kwartaal van 2006; de rapportage daarover zal medio 2006 verschijnen; …;*
> - *Vierde meting (over 2008) in het eerste kwartaal van 2009; de rapportage daarover en de eindevaluatie (over de gehele periode 2005–2008) zal medio 2009 verschijnen." (p.1-2)*

KHN heeft zich ingespannen om de zelfregulering een succes ter maken en de afgesproken resultaten, die nu eenmaal kwantitatief waren, zijn behaald. Toch heeft minister Klink de overeenkomst tussen de overheid (minister Hoogervorst) en KHN over de zelfregulering van het rookbeleid in de horeca eenzijdig opgezegd en vervolgens eenzijdig beslist tot de invoering van een algeheel wettelijk rookverbod. Daarbij heeft hij het voorstel van KHN om de invoering van het wettelijk rookverbod in de horeca te faseren, naast zich neergelegd. Deze gang van zaken lijkt moeilijk te rijmen met de toezegging in het regeerakkoord dat in samenspraak met KHN aan een rookvrije horeca gewerkt zal worden[43]:

> *"Mevrouw Schippers (VVD): … In het regeerakkoord staat dat in samenspraak met de branche wordt gewerkt aan een rookvrije horeca. De minister kondigt echter een-*

42. TK, 2004–2005, 29800 XVI, nr.162.
43. TK, 2006–2007, 30800 XVI, nr.182.

zijdig aan het rookverbod 2,5 jaar eerder te laten ingaan. Dat heeft geleid tot veel ongenoegen. Waaruit bestaat die samenspraak dan eigenlijk?"

"Mevrouw Joldersma (CDA) meent dat de minister de tijdgeest goed aanvoelt door te kiezen voor een rookvrije horeca per 1 juli 2008. Het maatschappelijk draagvlak is toegenomen. Dat is niet in de laatste plaats te danken aan de horeca zelf. Het stappenplan heeft bijgedragen tot steeds meer maatregelen om de werknemer en de niet-roker te beschermen. De doelstellingen zijn grotendeels gehaald, maar de ambities waren niet al te groot. De afspraak rond rookvrije horeca was dat een en ander in samenspraak met Koninklijke Horeca Nederland zou plaatsvinden. Koninklijke Horeca Nederland spreekt echter van een onbetrouwbare overheid. Hoe heeft die samenspraak dan plaatsgevonden? (p.4)

KHN spreekt niet geheel ten onrechte van een onbetrouwbare overheid en dat kan minister Klink parten hebben gespeeld bij de handhaving van het rookverbod in kleine cafés zonder personeel. KHN is hem begrijpelijkerwijs toen niet te hulp geschoten.

In paragraaf 4.1 heb ik gesteld:

"De kernelementen van een fair keuzeproces … betreffen de ***argumentatie*** *(de gebruikte informatie en gevolgde redeneringen) die ten grondslag ligt aan de besluitvorming en de* ***besluitvormingsprocedure*** *(welke partijen kunnen op welke moment welke invloed uitoefenen op de besluitvorming?). …* ***De gerechtvaardigdheid van de besluitvormingsprocedure*** *(Engels: procedural justice)* ***valt buiten het kader van het model*** *(zie paragraaf 1.3). … De gerechtvaardigdheid van de argumentatie vormt de kern van het model."*

M.i. heeft minister Klink overtuigende argumenten gebruikt ter onderbouwing van zijn besluit tot de invoering van een algeheel wettelijk rookverbod. De besluitvormingsprocedure die hij heeft gevolgd, verdient echter geen schoonheidsprijs. De gerechtvaardigdheid van de besluitvormingsprocedure valt echter buiten het kader van het model.

Handhaving van het rookverbod door werkgevers

De keuze van handhaving van het rookverbod in de horeca door werkgevers (en door werknemers in opdracht van de werkgever) is eigenlijk in 2000 al gemaakt door minister Borst-Eilers bij het besluit tot invoering van de rookvrije werkplek, dat onderdeel was van haar Wetsvoorstel tot wijziging van de Tabakswet[44]. Zij heeft m.i. overtuigend beargumenteerd waarom het de voorkeur verdient om

44. TK, 1998-1999, 26472, nrs.1-2.

werkgevers verantwoordelijk te maken voor de handhaving van de rookvrije werkplek en om niet de roker maar de werkgever te beboeten, als het recht op de rookvrije werkplek wordt overtreden. Zij heeft hiervoor verschillende argumenten aangevoerd[45]:

> *"Het is goed om eveneens te kunnen vaststellen dat deze leden [Groen Links] het toejuichen om werkgevers, vervoersexploitanten, luchtvaartmaatschappijen en beheerders van openbare instellingen stringenter verantwoordelijk te maken voor het niet roken, omdat hierdoor de niet-roker door de verantwoordelijke instantie wordt beschermd en niet meer in de huidige moeilijke situatie wordt gebracht om bij collega's, medebezoekers of personeel te moeten vragen om gevrijwaard te worden van ongezonde tabaksrook."* (p.4)

> *"Deze leden [PvdA] vragen ook waarom niet wordt overwogen een rechtstreekse boete te geven aan de roker die een rookverbod overtreedt en wat eigenlijk het principiële verschil is tussen handhaving van het verbod op wildplassen en handhaving van het rookverbod. … Evenals de leden van deze fractie [PvdA] heb ik mij in eerste instantie eveneens afgevraagd of niet ook de individuele overtreder van het rookverbod, de roker, bestraft zou moeten worden. Mede gelet op de plaatsen waar zulke overtredingen plaatsvinden, zal het echter niet goed mogelijk zijn om zo een wettelijke bepaling in redelijke mate te handhaven. Het voor zo'n overtreding uitdelen van een bestuurlijke boete door het personeel of de beheerder van de instelling is niet mogelijk. Verder schat ik in dat bij het bestraffen van de roker zelf er een aannemelijke kans bestaat op een negatieve uitwerking. Uiteraard dient de individuele overtreder wel terstond door het personeel van de instelling, de beheerder zelf of het bezoekende publiek te worden aangesproken op zijn rookgedrag, omdat hij of zij niet alleen een maatschappelijke norm overtreedt, maar ook de huisregels van de instelling die voor een deel wettelijk zijn vastgelegd. Het verschil met wildplassen is dat dit veelal op de openbare weg gebeurt en dat toezicht op de naleving van dit verbod bij de politie ligt, die regelmatig surveilleert en direct tot actie kan overgaan bij constatering van een overtreding."* (p.53)

> *"In het licht van de eigen verantwoordelijkheid van de roker vragen de leden van de fractie van de VVD de regering om nog eens in het algemeen een onderbouwing te geven van de reden waarom alleen de verkoper van tabaksproducten (en niet ook de jongere onder de 18 jaar) en uitsluitend de beheerder van een openbaar gebouw waar een rookverbod geldt (en niet ook de roker aldaar) met sancties wordt aangepakt. Het antwoord hierop is dat de regering niet wil dat groepen jongeren en rokers «gecriminaliseerd » zouden worden.* (p.56)

45. TK, 1999–2000, 26472, nr.6.

Samenvattend geeft minister Borst-Eilers de volgende argumenten om werkgevers/ exploitanten verantwoordelijk te maken voor de handhaving van het rookverbod en om niet de roker maar de werkgever/ exploitant te beboeten, als het rookverbod wordt overtreden:

- werkgevers zijn verantwoordelijkheid voor de veiligheid van hun personeel;
- voor niet-rokers kan het moeilijk zijn om bij collega's, medebezoekers of personeel te moeten vragen om gevrijwaard te worden van tabaksrook;
- het roken in besloten ruimten zal zich vaak aan de waarneming van de politie onttrekken, maar niet aan de waarneming van werkgevers of werknemers die in die ruimten werkzaam zijn;
- bij het bestraffen van de roker zelf bestaat er een aannemelijke kans op een negatieve uitwerking (minister Borst-Eilers licht niet toe wat zij met een negatieve uitwerking bedoelt; agressie?);
- voorkomen van criminalisering van de roker.

Ten tijde van de invoering van het wettelijke rookverbod in de horeca door minister Klink, wordt in de tweede Kamer het debat over het beboeten van de werkgever en/of de roker, als het rookverbod in de horeca wordt overtreden, opnieuw gevoerd:

> *"Mevrouw Bouwmeester (PvdA): … Mevrouw Bouwmeester vindt dat de uitbater strafbaar gesteld moet worden als hij de regels overtreedt, maar de horeca wil de rokers zelf strafbaar stellen. Dat criminaliseert de rokers en dat is niet de bedoeling. Er is ook sprake van rechtsongelijkheid, want iemand die rookt op kantoor, krijgt geen boete."* (p.1)

> *"Mevrouw Kant (SP): … Hoe zit het boetesysteem in elkaar? Wordt alleen de cafébhouder beboet of ook de persoon die ergens rookt waar het niet mag? Er moet een systeem komen waarbij het normatieve karakter vooropstaat en niet het straffen en beboeten. Dat zal echter niet eenvoudig zijn. Er moet geen rookpolitie komen."* (p.3)

In 2011 betoogt minister Schippers dat het vanwege de hiërarchische relatie voor werknemers moeilijk is om bij hun werkgever te vragen om gevrijwaard te worden van tabaksrook[46]:

> *"Het uitgangspunt van de wet is het beschermen van de werknemers tegen tabaksrook. Waarom? Omdat sprake is van een hiërarchische relatie. De werknemer kan wel denken «ik heb het eigenlijk liever niet», maar durft dat misschien niet te zeggen. Dan staat hij in de rook. Dat is dus het uitgangspunt."* (p.29)

46. TK, 2010–2011, 32011, nr.15.

Controle van handhaving van het rookverbod door de VWA

In de Memorie van toelichting behorend bij het Wetsvoorstel tot wijziging van de Tabakswet beargumenteert minister Borst-Eilers zeer grondig waarom zij kiest voor bestuursrechtelijke in plaats van strafrechtelijke handhaving van de voorgestelde rookverbod. Het strafrecht blijft enkel als ultimum remedium van toepassing[47]:

> *"Het huidige kabinetsbeleid inzake de rechtshandhaving, …, houdt onder meer een versterking van de bestuurlijke handhaving in. Handhaving van bestuursrechtelijke wetgeving is primair de verantwoordelijkheid van de bestuursorganen die met de uitvoering daarvan zijn belast. Het beleid is er op gericht de handhaving meer integraal deel te laten uitmaken van bestuurlijke beslissingen rond vaststelling en uitvoering van wetgeving. Tevens geldt dat het bestuursorgaan belast met de uitvoering van vaak zeer specifieke wetgeving qua deskundigheid en ervaring in beginsel beter dan andere overheidsinstellingen is toegerust om de handhaving efficiënt en effectief te laten zijn. Een dergelijk bestuursorgaan – vaak ook belast met het toezicht op de naleving – is veelal goed op de hoogte van de werkwijze van «het veld»,de ontwikkelingen, de actoren en natuurlijk inhoud en doelstelling van het overtreden voorschrift."* (p.14)

> *"Op basis van het primaat van de bestuurlijke handhaving heeft het kabinet er voor gekozen nieuwe handhavingsinstrumenten te ontwikkelen. Daaronder valt ook de bestuurlijke boete. De bestuurlijke boete kan er toe bijdragen dat het bestuur de eigen verantwoordelijkheid voor de handhaving beter kan waarmaken dan thans het geval is. Voorts geldt dat een systeem van bestuurlijke boeten veelal een snellere sanctionering mogelijk maakt ten aanzien van overtreders. De strafrechtelijke handhaving van vooral het economische ordeningsrecht blijft thans – mede in verband met capaciteitsproblemen bij het openbaar ministerie – vaak onderbelicht. Het gevolg daarvan is dat in een aantal gevallen handhaving achterwege blijft of te lang op zich laat wachten."* (p.14)

> *"Na het rapport van de Commissie voor de toetsing van wetgevingsprojecten is de regering tot de conclusie gekomen dat diverse geboden en verboden bij of krachtens de Tabakswet voor handhaving door bestuurlijke boeten in aanmerking komen. Voor veel voorschriften geldt dat zij voldoen aan de door de Commissie gehanteerde criteria voor bestuursrechtelijke handhaving. … Ik stel dan ook voor het instrument van de bestuurlijke boetes te introduceren voor de reclamebeperkingen, alsmede de verkoop- en rookverboden. Zodoende zal de inspectie W & V ter zake slagvaardiger en adequater kunnen opereren. Overigens zij benadrukt dat de Wet op de economische delicten als ultimum remedium van toepassing blijft."* (p.14-15)

47. TK, 1998–1999, 26472, nr.3.

"Het karakter van de vigerende en voorgestelde voorschriften is een belangrijke overweging om het instrument van de bestuurlijke boete in de Tabakswet te introduceren. Overtreding pleegt in de regel geen (direct) letsel aan personen of schade aan goederen toe te brengen, noch een direct gevaar daarvoor op te leveren. Voor een veroordeling door de strafrechter op deze grond is slechts zelden aanleiding. Dit strookt met de inzichten verkregen uit de strafrechtelijke handhavingspraktijk van de Tabakswet. Op jaarbasis worden niet of nauwelijks processen-verbaal opgemaakt voor overtredingen van de bij of krachtens de Tabakswet gestelde voorschriften. Het komt dan ook niet of nauwelijks tot vervolging door het openbaar ministerie. … Een en ander leidt tot de conclusie dat het enkele overtreden van de norm veelal niet onmiddellijk een situatie in het leven roept, die de rechtsorde ernstig schokt. In dat licht bezien, is een strafrechtelijke sanctie – afgezien van bijzondere situaties – niet de meest passende reactie." (p.15)

"Niettemin kan het overtreden van een wettelijke norm, zeker in combinatie met andere overtredingen, wel degelijk situaties betreffen die op termijn schadelijk zijn voor de volksgezondheid, dan wel leiden tot maatschappelijk onaanvaardbare gedragingen richting de consument. Dit betekent dat adequate handhaving van de normen van de Tabakswet van groot belang is, zelfs in het geval dat het overtreden van een enkele norm niet onmiddellijk tot ernstig gevaar lijkt te leiden, noch omvangrijke economische benadeling van consument en concurrentie tot gevolg heeft. Een strafrechtelijke reactie kan dan echter als onevenredig zwaar worden beschouwd en ligt in zo'n geval daarom minder voor de hand. Daar komt bij dat het huidige handhavingsniveau voor verbetering vatbaar is." (p.15)

Het openbaar ministerie blijkt in de huidige strafrechtelijke handhavingspraktijk niet altijd even optimaal te kunnen reageren waar het gaat om grote aantallen overtredingen die een aanzienlijke mate van technisch inzicht op het gebied van levensmiddelen en niet-levensmiddelen vereisen. Een ten allen tijde adequate reactie op dergelijke overtredingen is ook gegeven de huidige omstandigheden – gezien de eerder aangegeven brede taakstelling van het openbaar ministerie – niet mogelijk. Bovendien speelt ook de tijdsduur van de procedure een rol. De strafrechtelijke handhavingspraktijk laat zien dat tussen het moment van geconstateerde overtreding en uiteindelijke afdoening door een vonnis door de economische politierechter, veel tijd verstrijkt. Van een «lik op stuk»-beleid, waarbij de overtreder direct wordt geconfronteerd met de voor hem onaangename gevolgen van de door hem begane overtreding waarmee wordt bijgedragen aan zijn bewustwording van normovertredend gedrag, komt aldus weinig terecht. De invoering van de bestuurlijke boete in de Tabakswet kan de handhaving van deze wet en de daarop gebaseerde wettelijke voorschriften vanuit dit perspectief bezien, versterken." (p.15-16)

In geval van bestuurlijke handhaving van het rookverbod in de horeca is de Voedsel en Waren Autoriteit (VWA), die toezicht houdt op de uitvoering van de tabakswetgeving, de aangewezen partij.

Samenvattend geeft minister Borst-Eilers de volgende argumenten voor bestuurlijke handhaving c.q. handhaving door de Voedsel en Waren Autoriteit (voorheen: Keuringsdienst van Waren) van de rookverboden op basis van de Tabakswet:

- bestuursorganen beschikken over meer deskundigheid over en ervaring met het ‘veld’ en kennen de beleidsdoelstellingen van de overheid beter, wat een voordeel is bij de handhaving van wetgeving;
- stafrechtelijke handhaving is een te zwaar instrument in relatie tot de ernst van de overtreding;
- ‘lik op stuk beleid’ is effectiever als gedragsverandering wordt beoogd en is in geval van bestuursrechtelijke handhaving beter te realiseren.

Vraag 2: Zijn de partijen die de preventiemaatregel uitvoeren daartoe bekwaam en bevoegd?

Alle bij de uitvoering van het rookverbod in de horeca betrokken partijen – de overheid, werkgevers (en werknemers), en de VWA – zijn daartoe bekwaam en bevoegd. Zie het antwoord op vraag 1.

Vraag 3: Zijn de partijen die de preventiemaatregel uitvoeren daartoe geschikt?

Alle bij de uitvoering van het rookverbod in de horeca betrokken partijen zijn daartoe geschikt. Zie het antwoord op vraag 1.

Vraag 4: Brengt uitvoering van de preventiemaatregel schade toe aan de aard of het imago van de partijen die de maatregel uitvoeren?

Uitvoering van het rookverbod in de horeca kan schade toebrengen aan de aard van met name cafés, omdat ‘cafés – alcohol – roken’ van oudsher met elkaar verbonden zijn. Volgens minister Schippers tast het rookverbod het karakter van kleine cafés zonder personeel aan, omdat bezoekers dergelijke cafés als hun huiskamer beschouwen, waarbinnen zij moeten kunnen roken.

Alle partijen die de preventiemaatregel uitvoeren lopen risico op imagoschade, omdat werkgevers/ eigenaars, werknemers en bezoekers zich boos kunnen maken omdat er niet meer mag worden gerookt, of juist omdat het rookverbod onvoldoende wordt nageleefd.

11.6.3 Eindoordeel (criterium geschiktheid)

Vraag 1

Is uitvoering van de preventiemaatregel door andere partijen niet logischer gezien de doelgroep en leefstijlbeïnvloedende factoren waarop de maatregel zich richt?

Antwoord op vraag 1
Nee, met name minister Borst-Eilers heeft de keuze van de uitvoerende partijen grondig onderbouwd.

Vraag 2
Zijn de partijen die de preventiemaatregel uitvoeren daartoe niet onbekwaam of onbevoegd?

Antwoord op vraag 2
Nee, integendeel (zie de onderbouwing van het antwoord op vraag 1).

Vraag 3
Zijn de partijen die de preventiemaatregel uitvoeren daartoe niet ongeschikt?

Antwoord op vraag 3
Nee, integendeel (zie de onderbouwing van het antwoord op vraag 1).

Vraag 4
Brengt de preventiemaatregel geen onaanvaardbare schade toe aan de aard of het imago van de partijen die de maatregel uitvoeren?

Antwoord op vraag 4
Met uitzondering van de kleine cafés zonder personeel – de zogenaamde huiskamerkroegjes – is dat niet het geval. De schade aan de aard en het imago van huiskamerkroegjes moet worden afgewogen tegen het negatieve effect op de handhaafbaarheid van het rookverbod in de horeca, als deze kroegjes van dit rookverbod worden uitgezonderd.

11.6.4 Gevolgen van de beoordeling voor het model (criterium geschiktheid)

De beoordeling van het wettelijk rookverbod in de horeca aan de hand van het criterium 'geschiktheid' van het model leidt tot een kleine aanpassing van de vragen waarmee ik het criterium 'geschiktheid' operationaliseer en beoordeel. In deze vragen vervang ik het enkelvoud 'partij' door het meervoud 'partij<u>en</u>'.

11.7 Eindoordeel over de 1e zeef: logica van het ontwerp

11.7.1 Beoordeling van de logica van het ontwerp van het wettelijk rookverbod in de horeca

Beoordeeld wordt de logica van het ontwerp van het wettelijk rookverbod in de horeca op basis van het uitgangspunt dat de doelstelling van dit rookverbod zich beperkt tot de bescherming van werknemers in de horeca tegen passief roken. De schadelijkheid (1e criterium) van passief roken in de horeca voor werknemers en de noodzaak (2e criterium) van preventie zijn overtuigend aangetoond. De doelgroep (alle rokers in de horeca: werkgevers, werknemers en bezoekers) waarop de preventiemaatregel zich richt (5e criterium) is logisch en de gehele doelgroep wordt door de preventiemaatregel geraakt. De geschiktheid (6e criterium) van de overheid (wetgeving), werkgevers (handhaving rookverbod) en VWA (controle op handhaving) als uitvoerende partijen, is goed beargumenteerd.

Bij het toewijzen van de verantwoordelijkheid (4e criterium) voor de schade, hinder en overlast ten gevolge van passief roken in de horeca, heeft de overheid geen rekening gehouden met de determinanten (3e criterium) van het rookgedrag dat wordt verboden. Omdat de preventiemaatregel het roken niet volledig verbiedt, maar alleen in de horeca, is het niet onredelijk dat alle verantwoordelijkheid voor het risico op schade, hinder en overlast bij de rokers wordt gelegd.

Als het uitgangspunt wordt gehanteerd dat de doelstelling van het wettelijk rookverbod in de horeca zich beperkt tot de bescherming van werknemers tegen passief roken, zijn er goede argumenten voor (gelijk speelveld, handhaafbaarheid), maar ook goede argumenten tegen (geen werknemers), om ook aan kleine cafés zonder personeel een wettelijk rookverbod op te leggen.

De stelling van minister Schippers dat er voor kleine cafés zonder personeel geen behoefte bestaat aan een wettelijk rookverbod, acht ik onvoldoende onderbouwd. Enerzijds zouden eigenaars van dergelijke cafés behoefte kunnen hebben aan een wettelijk rookverbod (zie §10.3.2). Anderzijds zouden bezoekers van dergelijke cafés behoefte kunnen hebben aan een wettelijk rookverbod. Ter illustratie hiervan een citaat uit een algemeen overleg in de Tweede Kamer over het voorstel van minister Schippers om het wettelijk rookverbod op te heffen voor kleine cafés zonder personeel[48]:

48. TK, 2010–2011, 32011, nr.15.

"Hebben mensen nu hun buurtkroeg terug? Nee, die hebben ze niet terug. Ik kreeg verscheidene mails, bijvoorbeeld van een vrouw die dart en van een man die biljart. Zij schreven mij dat zij konden darten of biljarten omdat dat gebeurde in een rookvrije buurtkroeg. Nu er weer wordt gerookt, kunnen deze mensen hun lidmaatschap opzeggen. Zij schrijven: een ander heeft zijn vrijheid terug om overlast te veroorzaken, en ik kan mijn eigen kroeg niet meer in. Vrijheid? Het is maar hoe je dat begrip interpreteert." (p.3)

Deze kritiek op de argumentatie van minister Schippers valt echter buiten het kader van de preventiemaatregel die wordt beoordeeld, omdat de doelstelling van deze maatregel zich beperkt tot de bescherming van werknemers in de horeca tegen passief roken.

De preventiemaatregel is niet moralistisch, niet perfectionistisch en niet paternalistisch. De preventiemaatregel kan de eerste zeef van het model kan passeren: ***het ontwerp van de preventiemaatregel is niet onlogisch.***

11.7.2 Gevolgen van de beoordeling voor het model (1e zeef: logica van het ontwerp)

De beoordeling van het wettelijk rookverbod in de horeca aan de hand van de criteria van de 1e zeef van het model heeft geleid tot een aantal aanpassingen aan en conclusies over het model. Hieronder worden deze besproken.

Belang van transparantie van de doelstelling
Bij de beoordeling van het rookverbod aan de hand van het criterium 'schadelijkheid' ben ik aanvankelijk vastgelopen, omdat de doelstelling van dit rookverbod uit de parlementaire verslagen niet eenduidig valt op te maken. Voor de toepassing van het model blijkt het essentieel te zijn, dat duidelijk is welke risico's aanleiding vormen tot het treffen van de preventiemaatregel. Als daarover onduidelijkheid bestaat, is de gerechtvaardigdheid van de preventiemaatregel aan de hand van het model niet te beoordelen, omdat alle volgende criteria van het model de preventie van deze risico's als vertrekpunt hebben.

Aanvulling op operationalisering van schadelijkheid
Aan de risicocategorie 'overlast' van het criterium schadelijkheid is het begrip 'hinder' toegevoegd (zie §11.1.2).

Precisering van operationalisering van gerichtheid
Aan twee vragen behorend bij het criterium 'gerichtheid' is het woord 'leefstijl' toegevoegd (zie §11.5.4).

Precisering van operationalisering van geschiktheid
In de vragen behorend bij het criterium 'geschiktheid' is het enkelvoud 'partij' vervangen door het meervoud 'partijen' (zie §11.6.2).

Conclusies over juistheid, volledigheid en praktische toepasbaarheid
Met inachtneming van het bovenstaande kunnen de volgende conclusies worden getrokken. De beoordeling van het wettelijk rookverbod in de horeca aan de hand van de 1e zeef van het model leidt niet tot aanwijzingen dat de criteria van de 1e zeef onjuist, onvolledig, of praktisch niet toepasbaar zijn. Het blijkt mogelijk om op basis van de criteria van de 1e zeef tot een beoordeling van de 'logica van het ontwerp' van het wettelijk rookverbod in de horeca te komen.

12 Toetsing 2e zeef van het beoordelingsmodel aan casus 'wettelijk rookverbod in horeca' (versie 8 van het model)

In dit hoofdstuk beoordeel ik de gerechtvaardigdheid van het wettelijk rookverbod in de horeca aan de hand van de criteria van de 2e zeef van het model (versie 8; zie §10.4).

12.1 Zevende criterium: effectiviteit

12.1.1 Operationalisering en wijze van beoordeling

In §7.1.1 heb ik het criterium 'effectiviteit' als volgt gedefinieerd:

> *"De effectiviteit betreft de beoogde effecten van de preventiemaatregel op de doelgroep, waarbij de doelgroep is gedefinieerd als de groep personen waarvan men ter voorkoming van leefstijlgerelateerde aandoeningen de leefstijl tracht te beïnvloeden."*

Naar aanleiding van de beoordeling van de effectiviteit van het wettelijk rookverbod in de horeca, realiseer ik mij dat deze definitie onvolledig is. De effectiviteit betreft niet alleen de beoogde effecten van de preventiemaatregel op de doelgroep (de rokers in de horeca), maar ook de beoogde effecten op derden (de werknemers in de horeca die men tegen passief roken tracht te beschermen). Daarom pas ik de definitie van het criterium 'effectiviteit' als volgt aan:

> *"De effectiviteit betreft de beoogde effecten van de preventiemaatregel op de doelgroep of op derden. De doelgroep is hierbij gedefinieerd als de groep personen waarvan men de leefstijl tracht te beïnvloeden ter voorkoming van leefstijlgerelateerde aandoeningen bij de doelgroep zelf of bij derden."*

Mijn eerdere definitie van het criterium 'effectiviteit' vervalt hiermee.

Belangrijke aandachtspunten bij het beoordelen van de effectiviteit zijn:
- effectiviteit per risico dat men tracht te bestrijden;
- kwantificering van de effectiviteit;
- gebruik van proces- en uitkomstindicatoren;
- duurzaamheid van de effecten.

Beoordeeld wordt of de preventiemaatregel effectief is en of de effectiviteit duurzaam is. Is de informatie over de effectiviteit volledig, voldoende gedetailleerd en voldoende gekwantificeerd? Als onvoldoende informatie beschikbaar is, wordt beoordeeld of de informatieverzameling over de effectiviteit voldoende is geweest.

12.1.2 Beoordeling van casus 'wettelijk rookverbod in horeca' aan de hand van criterium 'effectiviteit'

Diverse vormen van schade, hinder en overlast maken het volgens de overheid noodzakelijk om een wettelijk rookverbod in de horeca in te voeren (zie overzicht 19 in §11.2.3):

- risico op gezondheidsschade bij werknemers ten gevolge van passief roken;
- aantasting van de lichamelijke integriteit van werknemers ten gevolge van passief roken;
- aantasting van de keuzevrijheid van werknemers om daar te werken (solliciteren) waar zij willen ten gevolge van passief roken;
- hinder en overlast voor werknemers ten gevolge van passief roken (benauwdheid, hoofdpijn, duizeligheid, irritatie, verminderd smaakvermogen, stank).

Een algeheel rookverbod is in theorie 100% effectief in het voorkomen van passief roken en daarmee in het voorkomen van alle bovengenoemde vormen van schade, hinder en overlast.

Het rookverbod in de horeca heeft er niet toe geleid dat werknemers in de horeca helemaal niet meer worden blootgesteld aan passief roken. Enerzijds doordat mensen het rookverbod overtreden, anderzijds doordat de regelgeving toestaat dat in de horeca in afgesloten rookruimten wordt gerookt. Omdat de deuren van afgesloten rookruimten af en toe geopend moeten worden, om mensen naar binnen en buiten te laten, kan er vanuit de rookruimte tabaksrook in de rookvrije ruimte naar binnen lekken, die dan niet meer volledig rookvrij is. Ook dienen horecawerknemers eventuele rookruimten soms te betreden, b.v. om schoon te maken. In het kader van het politieke debat, is er veel over gediscussieerd of moderne ventilatiesystemen een alternatief voor het rookverbod (of voor rookruimten) kunnen vormen. Samenvattend zijn bepalend voor de blootstelling aan passief roken in horecagelegenheden waar een rookverbod geldt:

- het al dan niet toestaan van rookruimten;
- het al dan niet toestaan van ventilatiesystemen als alternatief voor het rookverbod (of voor rookruimten);
- de naleving van het rookverbod.

Wat betreft de naleving zal ik in deze paragraaf alleen ingaan op de mate waarin het rookverbod wordt nageleefd. Op de oorzaken van de mate van naleving, zoals het draagvlak voor het rookverbod en de handhaving van het rookverbod, zal ik ingaan bij de criteria 'draagvlak' en 'handhaafbaarheid' die onderdeel zijn van de 3e zeef van het model.

In oktober 2009 verschijnt het onderzoeksrapport 'Even uitblazen. Eén jaar rookvrije horeca' van Regioplan Beleidsonderzoek (Regioplan, 2009). Dit rapport is een review van de onderzoeksrapporten van andere organisaties (TNS NIPO, INTRAVAL, Synovate, Foodstep, RIVM, enz.) over de gevolgen van het rookverbod in de horeca. Het ministerie van VWS heeft Regioplan opdracht gegeven tot dit reviewonderzoek. Op 1 december 2009 rapporteert minister Klink in een brief aan de Tweede Kamer over de resultaten van één jaar rookvrije horeca[1]. Hij baseert zich hierbij voornamelijk op het reviewrapport van Regioplan en op de onderzoeksrapporten waarop dit rapport is gebaseerd.

Al dan niet toestaan van rookruimten

In 2007 heeft minister Klink besloten tot het instellen van een rookverbod in de horeca in combinatie met de mogelijkheid tot het instellen van rookruimten. Minister Klink was echter niet de eerste minister die besloot tot de invoering van een rookverbod in combinatie met de mogelijkheid tot het instellen van rookruimten. De meeste rookverboden op basis van de Tabakswet kennen de mogelijkheid tot het instellen van rookruimten. In 2007 kon minister Klink, gezien de voorgeschiedenis, bijna niet anders dan besluiten om ook in de horeca rookruimten toe te staan. Ter onderbouwing van deze stelling geef ik een overzicht van de mogelijkheden om rookruimten in te richten in gebouwen waarvoor een rookverbod op basis van de Tabakswet geldt over de periode 1996 tot heden (juni 2011)[2].

Het 'Besluit beperking verkoop en gebruik tabaksprodukten' luidt op 1 april 1996 als volgt:

> "*Artikel 2*
>
> 1. *Als categorieën van ruimten waarin ingevolge artikel 10, tweede lid, van de Tabakswet (Stb. 1988, 342), een verbod om tabaksprodukten te gebruiken ingesteld en gehandhaafd dient te worden, worden aangewezen: … b. wach-*

1. TK, 2009–2010, 32011, nr.3.
2. De website www.overheid.nl gaat m.b.t. de tekst van het 'Besluit beperking verkoop en gebruik tabaksprodukten' niet verder terug dan 1 april 1996. Voor de onderbouwing van mijn stelling heeft het weinig toegevoegde waarde om verder terug te gaan in de tijd.

truimten; … h. recreatie- en soortgelijke ruimten; i. overige ruimten, voor zover deze voor het publiek toegankelijk zijn.

2. *Indien meer dan één ruimte, behorend tot een van de categorieën, genoemd onder b, h of i, in de inrichting aanwezig is, kan het bevoegde orgaan besluiten per categorie ten hoogste de helft van dit aantal ruimten van dit verbod uit te zonderen. Het verbod is in dat geval van toepassing op de ruimte of gezamenlijke ruimten met de grootste vloeroppervlakte. …*
3. *Indien slechts één ruimte, behorend tot een van de categorieën, genoemd onder b, h of i, in de inrichting aanwezig is, kan het bevoegde orgaan besluiten ten hoogste eenderde van de oppervlakte van die ruimte van dit verbod uit te zonderen, mits in het andere deel van die ruimte geen hinder wordt ondervonden van het gebruik van tabaksprodukten.*
4. *Indien slechts één ruimte, behorend tot een van de categorieën, genoemd onder b, h of i, in de inrichting aanwezig is en het derde lid geen toepassing vindt, kan het bevoegde orgaan besluiten per categorie de gehele ruimte van het verbod uit te zonderen gedurende ten hoogste een derde van de tijd van openstelling van die ruimte, volgend op of voorafgaand aan de periode dat het verbod van toepassing is, mits buiten eerstgenoemde tijd in die ruimte geen hinder van het gebruik van tabaksprodukten wordt ondervonden.*

Artikel 3

5. *Degenen die – anders dan in een hoedanigheid als bedoeld in artikel 10 van de Tabakswet – het beheer hebben over inrichtingen voor gezondheidszorg, maatschappelijke dienstverlening, sport, sociaal-cultureel werk of onderwijs, voor zover die inrichtingen behoren tot de in het tweede lid aangewezen categorieën, zijn verplicht maatregelen te treffen als bedoeld in artikel 10, eerste lid, van de Tabakswet."*

In 1998 vervallen het 3e en 4e lid van artikel 2 van het 'Besluit beperking verkoop en gebruik tabaksprodukten'[3]. Dit betekent een aanscherping van de dan geldende rookverboden in overheidsgebouwen en in de in artikel 3 van het 'Besluit' genoemde sectoren.

Op 1 januari 2004 is het recht op een rookvrije werkplek (Tabakswet, artikel 11a lid 1) van kracht geworden. Op dat moment golden er al rookverboden in ondermeer overheidsgebouwen en gebouwen in de sectoren gezondheidszorg en onderwijs. Op basis van het 'Besluit beperking verkoop en gebruik tabaksprodukten' was het

3. Staatsblad 1998, nr.572.

toegestaan om in overheidsgebouwen en in gebouwen in de sectoren gezondheidszorg en onderwijs rookruimten in te stellen. In 2003 kon daarom bijna niet anders besloten worden dan, om tegelijk met de invoering van het recht op een rookvrije werkplek in het bedrijfsleven, ook het instellen van rookruimten in het bedrijfsleven toe te staan. Minister Hoogervorst heeft het toestaan van rookruimten in bedrijven geregeld in het 'Besluit uitzonderingen rookvrije werkplek' dat per 1 januari 2004 van kracht is geworden. In de nota van toelichting bij dit besluit schrijft minister Hoogervorst[4]:

> "*... rookvrij betekent ook echt rookvrij. Vooralsnog sta ik op het standpunt dat het installeren en in werking stellen van ventilatie- en luchtzuiveringsapparatuur geen duurzaam alternatief biedt, omdat dergelijke apparatuur nauwelijks bescherming biedt tegen schadelijke tabaksrook (...). Het creëren van speciale rookruimten ... blijft niettemin mogelijk. Het scheiden van horeca-inrichtingen in gedeelten voor rokers en niet-rokers is geen optie vanuit een oogpunt van volksgezondheid en handhaving.*" (p.7)

> "*... Werkgevers kunnen besluiten tot het inrichten en aanwijzen van speciale, afgesloten rookruimten. De speciale rookruimten dienen afgesloten te zijn, zodat eventuele hinder of overlast van tabaksrook buiten deze ruimten tot een minimum beperkt blijft. Het is immers onvermijdelijk dat de toegangsdeur tot de rookruimte met enige regelmaat geopend wordt. Om d[t]e bewerkstelligen dat elders in een gebouw geen hinder of overlast wordt ondervonden van de tabaksrook in de rookruimte, staat een rookruimte idealiter niet in verbinding met het algemene ventilatiesysteem van een gebouw. De speciale afgesloten rookruimten zijn allereerst, en in de meeste gevallen, bestemd om rokers in een bedrijf de gelegenheid te bieden toch af en toe te roken zonder dat zij hiermee anderen hinder of overlast bezorgen. Niet-rokers hoeven deze ruimten in beginsel niet te betreden. Dit neemt niet weg dat er ook mensen zijn die in de rookruimten werkzaamheden moeten verrichten. Hierbij kan bijvoorbeeld worden gedacht aan schoonmaak- en onderhoudspersoneel. Ook wanneer zij hun schoonmaak- en onderhoudswerkzaamheden verrichten wanneer er niet wordt gerookt in de rookruimten, dan nog zal er sprake zijn van blootstelling aan tabaksrook. Deze blijft immers nog vele uren hangen nadat er is gerookt. ...*" (p.10)

Minister Hoogervorst realiseert zich dat het toestaan van rookruimten een negatieve invloed heeft op de effectiviteit van het rookverbod. Enerzijds omdat het onvermijdelijk is dat de toegangsdeur tot de rookruimte met enige regelmaat geopend wordt. Anderzijds omdat (niet-rokend) personeel de rookruimte soms zal moeten betreden.

4. Staatsblad 2003, nr.561.

Per 1 juli 2008 vervangt minister Klink het 'Besluit uitzonderingen rookvrije werkplek' door het 'Besluit uitvoering rookvrije werkplek, horeca en andere ruimten'. In de nota van toelichting bij dit besluit schrijft minister Klink over de rookruimten[5]:

> *"Werkgevers kunnen besluiten tot het inrichten en aanwijzen van afsluitbare, voor het roken van tabaksproducten aangewezen en als zodanig aangeduide ruimten. De rookruimten dienen afsluitbaar te zijn, zodat eventuele hinder of overlast van tabaksrook buiten deze ruimten tot een minimum beperkt blijft. De rookruimten zijn eerst en vooral bestemd om rokers in een bedrijf de gelegenheid te bieden toch af en toe te roken zonder dat zij hiermee anderen hinder of overlast bezorgen. Niet-rokers hoeven deze ruimten in beginsel niet te betreden. Dit neemt niet weg dat er ook mensen zijn die in de rookruimten werkzaamheden moeten verrichten. Hierbij kan bijvoorbeeld worden gedacht aan schoonmaak- en onderhoudspersoneel. …"*

> *"Een nadere toelichting verdient het schrappen van het woord «speciaal» uit artikel 2, onderdeel h van het Besluit uitzonderingen rookvrije werkplek. … De huidige formulering brengt tot uitdrukking dat rookruimten weliswaar bestemd zijn om in te roken, maar tevens dat in rookruimten meer is toegestaan dan alleen roken. Speciaal met betrekking tot de horeca betekent dit dat men er ook een consumptie mee naar binnen mag nemen. Daarentegen is wel duidelijk dat het personeel deze ruimten niet mag betreden in het kader van het uitoefenen van de normale horecawerkzaamheden, zoals het bedienen van klanten."* (p.7)

In juni 2011[6] is naast het 'Besluit uitvoering rookvrije werkplek, horeca en andere ruimten' ook het 'Besluit beperking verkoop en gebruik tabaksprodukten' nog steeds van kracht. Dit laatstgenoemde besluit heeft in de loop der jaren een aantal veranderingen ondergaan en is inhoudelijk geharmoniseerd met het 'Besluit uitvoering rookvrije werkplek, horeca en andere ruimten'.

In november 2009 beantwoordt een internationale commissie van deskundigen ondermeer de volgende vraag van het ministerie van VWS (Brunekreef. QANU. 2009):

> *"Vraag: Zijn er andere opties dan het inrichten van aparte rookruimtes die garanderen dat de concentratie omgevingstabaksrook beneden een bepaald maximum niveau blijft?*
> *Antwoord: Aangezien er geen gezondheidkundig verantwoord maximum concentratie omgevingstabaksrook anders dan afwezigheid kan worden vastgesteld, zijn er*

5. Staatsblad 2008, nr.122.
6. In juni 2011 heb ik de casus 'wettelijk rookverbod in de horeca' beoordeeld aan de hand van het criterium 'effectiviteit' van het model.

> *geen andere opties dan een rookverbod om ervoor te zorgen dat de concentratie omgevingstabaksrook voldoende laag blijft. De beschikbare wetenschappelijke gegevens laten zien dat in ruimtes waar roken is toegestaan, het praktisch onmogelijk is om uit oogpunt van gezondheid aanvaardbaar lage concentraties omgevingstabaksrook te bewerkstelligen."* (p.5)

Belangrijk is om op te merken dat het antwoord luidt dat alleen een rookverbod werknemers in de horeca voldoende tegen passief roken kan beschermen. Hieruit volgt impliciet dat rookruimten onvoldoende (garantie op) bescherming van werknemers tegen passief roken bieden.

In 2010 stelt het onderzoeksrapport "Zwerfrook en Alternatieven voor Rookruimten" van RIVM en TNO ondermeer (Opperhuizen. RIVM. 2010):

> *"De aanwezigheid van rookruimten in horecagelegenheden zorgt ervoor dat concentraties omgevingstabaksrook in de rookvrije ruimten ongeveer een factor 10 tot 100 lager zijn dan in gelegenheden zonder rookverboden. Rookruimten in de negentien onderzochte Nederlandse horecagelegenheden kunnen de concentraties omgevingstabaksrook in de rookvrije ruimte niet op het niveau brengen van volledig rookvrije horeca. De bezetting bij grote drukte, het gebruik van klapdeuren en het wegstromen van vervuilde lucht uit de rookkamer richting rookvrije ruimte door het aangelegde ventilatiesysteem, kunnen bijdragen aan aanzienlijke lekkage van zwerfrook, en dus belasting van de rookvrije ruimte. De gevonden concentraties in de rookvrije ruimten zijn ongeveer een factor 10 hoger dan in volledig rookvrije horeca."* (p.3)

Rookruimten leiden tot zwerfrook in rookvrije ruimten en ondermijnen daarmee de effectiviteit van het rookverbod. In november 2008 heeft een lid van de vaste commissie voor volksgezondheid nog gepleit voor subsidiering van de bouw/inrichting van rookruimten[7]:

> *"De heer Van Gerven (SP): ... Wat vindt de minister van een subsidieregeling voor rookruimtes? Wij hebben dit al eerder naar voren gebracht. Hier moet serieus naar gekeken worden."* (p.6)

Minister Klink ziet geen noodzaak voor een subsidieregeling:

> *"Een subsidieregeling voor het maken van rookruimten, zo vraagt de heer Van Gerven. Ik heb van meet af aan gezegd niet al te veel eisen te verbinden aan de rookruimte. Zo jagen wij horecaondernemers niet per definitie op hoge kosten. De afweging is aan de ondernemer. In de knelpuntenanalyse nemen wij wellicht mee in hoeverre er sprake is van een belemmering. Ik zie voorshands geen noodzaak voor een subsidieregeling."* (p.15)

7. TK, 2008–2009, 22894, nr.207.

Naar aanleiding van het rapport van het RIVM en TNO uit 2010 'Zwerfrook en Alternatieven voor Rookruimten' zouden er met het oog op de effectiviteit van het rookverbod misschien juist wel eisen moeten worden gesteld aan rookruimten.

In zijn brief aan de Tweede Kamer d.d. 1 december 2009 over de resultaten van één jaar rookvrije horeca schrijft minister Klink over het aantal rookruimten in horecagelegenheden[8]:

> *"… Een aanzienlijk deel van de horecaondernemers heeft aanpassingen aan de horecagelegenheid gedaan om aan de bepalingen van de Tabakswet te voldoen: 72% beschikt over een rookvoorziening (binnen en/of buiten). Van deze horecagelegenheden heeft 38% een rookruimte binnen en 85% heeft buiten een voorziening (terras, tuin, asbakken of afdakjes). Slecht een klein deel van de ondernemers is van plan om komend jaar nog aanpassingen te doen in hun horecagelegenheid.*

Al dan niet toestaan van ventilatiesystemen

In mei 2004 schrijft minister Hoogervorst in een brief aan de Tweede Kamer, naar aanleiding van het onderzoeksrapport 'Reductie van blootstelling aan omgevingstabaksrook in de horeca door ventilatie en luchtreiniging' van TNO en RIVM[9]:

> *"Ventilatie kan dus bijdragen aan het reduceren van de blootstelling aan tabaksrook en daarmee aan het verminderen van de gezondheidsrisico's van meeroken, maar het is niet waarschijnlijk dat een vermindering van de blootstelling een evenredige reductie van de gezondheidsrisico's betekent. Een volledige reductie van de gezondheidsrisico's is met ventilatie niet mogelijk. Dat betekent dat ventilatie wel kan bijdragen aan een comfortabel binnenklimaat, maar dat een – vanuit het perspectief van meeroken – gezond binnenklimaat met ventilatie niet te realiseren is.*
>
> *Daarenboven brengt de introductie van de meest effectieve ventilatietechnieken voor de horeca prohibitieve investeringen met zich mee. Gezien deze gezondheidskundige overwegingen en bovenstaande praktische voorbehouden kom ik tot het oordeel dat ventilatie niet gezien moet worden als een effectieve oplossing om werknemers en bezoekers te beschermen tegen omgevingstabaksrook."* (p.2)

In juni 2006 schrijft minister Hoogervorst opnieuw een brief aan de Tweede Kamer, waarin hij toelicht waarom ventilatiesystemen niet kunnen worden gezien als een effectieve oplossing om werknemers en bezoekers te beschermen tegen omgevingstabaksrook[10]:

8. TK, 2009–2010, 32011, nr.3.
9. TK, 2003–2004, 29200 XVI, nr.233.
10. TK, 2005–2006, 30300 XVI, nr.144.

"Recent heeft de gezaghebbende «American Society of Heating, Refrigerating and Air-Conditioning Engineers (ASHRAE) een publicatie uitgegeven over ventilatie en omgevingstabaksrook … ASHRAE concludeert hierin dat:

- *een rookverbod op dit moment de enige effectieve manier is om de gezondheidsrisico's als gevolg van blootstelling aan omgevingstabaksrook uit te bannen, en;*
- *«engineering approaches» niet gezien kunnen worden als oplossing voor de problematiek van omgevingstabaksrook in ruimten waar wordt gerookt.*

Deze conclusies zijn mijns inziens volkomen helder en volledig in lijn met hetgeen het RIVM in zijn oordeel over het TNO-rapport heeft aangegeven. De conclusies van ASHRAE laten wederom zien dat ventilatie en eventuele andere technische maatregelen niet gezien kunnen worden als effectieve oplossing om werknemers en bezoekers te beschermen tegen omgevingstabaksrook. Een rookverbod is de enige effectieve manier om bescherming te bieden tegen de gezondheidsrisico's als gevolg van blootstelling aan omgevingstabaksrook." (p.2)

In juli 2007 geeft minister Klink in een algemeen overleg in de Tweede Kamer een toelichting op zijn voornemen om ventilatie- en verdringingssystemen niet toe te staan als alternatief voor het rookverbod[11]:

"Volgens 50 000 ventilatiedeskundigen verenigd in ASHRAE zijn de huidige ventilatie- en verdringingssystemen niet toereikend voor de bescherming van de werknemers en de gasten. Er blijven te veel schadelijke stoffen achter. Het RIVM concludeert dat er een reductiemogelijkheid is van 50%-79% en dat is ontoereikend. Discotheken en horecagelegenheden blijven ver boven het niveau dat uit gezondheidsoogpunt toelaatbaar is. Ook in het buitenland worden kritische kanttekeningen gezet bij ventilatiesystemen. Het gaat om grote hoeveelheden schadelijke stoffen die buitengewoon moeilijk te meten zijn. Een tweede probleem wordt veroorzaakt door het feit dat de intensiteit binnen de ruimtes die geventileerd worden, enorm varieert. Mochten er in de toekomst systemen komen die wel voldoende resultaat geven, dan zijn die natuurlijk bespreekbaar. De minister neigt naar een nulgrens. In het buitenland zijn twee opties: óf rookvrij met een afgeschermde rookplek óf een generiek verbod. Frankrijk heeft op basis van de bevindingen van ASHRAE besloten niet over te gaan tot introductie van ventilatiesystemen omdat deze ontoereikend zijn." (p.7)

In 2007 heeft de Europese Commissie in het Groenboek 'Op weg naar een rookvrij Europa' op basis van wetenschappelijk onderzoek geconcludeerd dat er geen veilig niveau van omgevingstabaksrook bestaat. Ook bij kleine dosis tabaksrook bestaat er een substantieel risico op hart- en vaatziekten. Daarom is het voor de effectivi-

11. TK, 2006–2007, 30800 XVI, nr.182.

teit van rookverboden essentieel dat minister Klink in 2007 heeft besloten om ventilatie- en verdringingssystemen niet toe te staan als alternatief voor het rookverbod.

In november 2010 schrijft minister Schippers een brief aan de Tweede Kamer, waarin zij aangeeft het rookverbod in de horeca voor kleine cafés zonder personeel te gaan opheffen. In deze brief schrijft zij over ventilatietechnieken als alternatief voor een rookverbod[12]:

> *"Ventilatie. Naar aanleiding van het RIVM-briefrapport «Handhaving van een rookvrij binnenmilieu» heeft mijn voorganger [Klink] u per brief van 8 juli 2009 geïnformeerd over zijn voornemen om nogmaals nauwkeurig te laten kijken naar de vraag of ventilatietechnieken een gelijkwaardig alternatief zouden kunnen zijn voor de rookvrije horeca. Daarop volgend heeft mijn voorganger u op 2 juli jl. twee rapportages gestuurd. Eén rapportage betrof een second opinion van de Commissie van Toezicht (CvT) van het RIVM over het RIVM-briefrapport «Handhaving van een rookvrij binnenmilieu»*[13]. Het tweede rapport betrof een aanvullend onderzoek van het RIVM en TNO naar ventilatietechnieken en de gehalten aan zwerfrook in de rookvrije gedeelten van horecagelegenheden met een rookruimte. Mijn voorganger heeft een besluit over vervolgacties naar aanleiding van dit onderzoek overgelaten aan een nieuw kabinet.
>
> *De voornaamste reden voor dit traject was een alternatief te kunnen bieden aan kleine cafés, die geen mogelijkheid hadden om een rookruimte te creëren. Voor deze categorie wordt nu een uitzondering op het rookverbod gemaakt. Bovendien volgt uit het rapport van de CvT [een second opinion] en het rapport van RIVM en TNO dat ventilatietechnieken voorlopig geen goed alternatief zijn voor de huidige rookruimtes, zowel qua gezondheidsbescherming, toepassing als handhaving. Ik ben daarom niet voornemens om de mogelijkheid van ventilatietechnieken als alternatief voor de rookvrije horeca, op korte termijn verder te verkennen."* (p.3)

Het briefrapport van het RIVM waarop minister Schippers zich baseert, stelt ondermeer (de 'second opinion' van de Commissie van Toezicht van het RIVM geeft geen aanleiding om aan de onderstaande conclusie te twijfelen):

> *"Conclusie: Luchtgordijnen kunnen verschillende technische ontwerpen hebben en hun functionaliteit wordt door veel factoren bepaald. Grote verstoringen kunnen optreden door menselijke beweging in ruimten met luchtgordijnen, evenals het onoordeelkundig gebruik. Toezicht op de functionele werking zal daarom arbeidsintensief en technisch ingewikkeld worden. Dat geldt in zekere zin ook voor vaste*

12. TK, 2010–2011, 22894, nr.289.
13. Brunekreef. QANU. 2009.

afscheidingen en voor rookruimten met deuren, maar de kans op onjuist gebruik door de roker (bijvoorbeeld half in de open rookvoorziening plaatsnemen) of de eigenaar (slecht onderhoud of manipulatie van de bediening) is hierbij kleiner." (p.11)

Naleving van het rookverbod

In zijn brief aan de Tweede Kamer d.d. 1 december 2009 over de resultaten van één jaar rookvrije horeca schrijft minister Klink over de naleving van het rookverbod in de horeca[14]:

> *In de herfst van 2008, de winter van 2008/2009 en het voorjaar van 2009 lag de naleving voor de gehele horeca op respectievelijk 93, 90 en 85%. Als gekeken wordt naar de resultaten van representatieve observaties door bureau INTRAVAL in opdracht van de VWA blijkt dat in de winter van 2008/2009, het voorjaar 2009, de zomer 2009 en het najaar van 2009 geen rokers zijn aangetroffen in respectievelijk 94, 97, 96 en 95% van de horecagelegenheden. Als gekeken wordt naar de deelsectoren valt op dat bij restaurants, cafetaria's, hotels en recreatie, sportkantines en kunst/ cultuur er nauwelijks veranderingen zijn: daar blijft het percentage gelegenheden waar geen rokers en geen asbakken zijn waargenomen zeer hoog (tussen 97 en 100%). Bij cafés en discotheken is er een daling waarneembaar van het percentage cafés en discotheken waar geen rokers (van 83 naar 73%) en geen asbakken (van 83 naar 69%) zijn waargenomen. De daling is het grootst bij cafés waar tijdens de observatie maar één personeelslid aanwezig was (bij een aanzienlijk deel daarvan zal het gaan om eenmanszaken). De meest voor de handliggende verklaringen voor deze dalingen zijn de uitspraken van de gerechtshoven in Den Bosch en Leeuwarden en mijn beslissing in reactie daarop om de handhaving voor horeca zonder personeel voorlopig op te schorten."* (p.2)

Eind 2010 daalt de naleving van het rookverbod in cafés en discotheken plotseling sterk. In januari 2011 stellen diverse leden van de vaste commissie voor Volksgezondheid van de Tweede Kamer in een algemeen overleg met de minister dat de oorzaak hiervan wordt gevormd door de versoepeling van het rookverbod in kleine cafés zonder personeel, die minister Schippers in november 2010 heeft aangekondigd. Ter illustratie een citaat van Tweede Kamerlid Van Gerven[15]:

> *"Een kwalijke zaak is de manier waarop de minister van Volksgezondheid de versoepeling van het rookverbod heeft aangekondigd. Met haar mantra van antibetutteling, leek zij meer op een echo van de tabakslobby dan op een minister van Volksgezondheid. Het gevolg was dat de handhaving van het rookverbod in drie maanden in een vrije val lijkt te zijn beland. Bij discotheken en cafés wordt het verbod nu*

14. TK, 2009–2010, 32011, nr.3.
15. TK, 2010–2011, 32011, nr.15.

massaal overtreden. Het laatste schokkende cijfer dat wij hoorden was dat 51% van de horecagelegenheden het rookverbod overtreedt. Drie maanden geleden lag dat percentage nog op 25. Om het draagvlak onder het rookverbod niet nog verder te ondermijnen, is handhaving van cruciaal belang." (p.6)

Effecten op de luchtkwaliteit

In december 2008 schrijft minister Klink in een brief aan de Tweede Kamer over de effecten van het rookverbod op de luchtkwaliteit[16]:

"De ervaringen in andere landen laten zien dat de luchtkwaliteit in de horeca na de invoering van een rookverbod substantieel verbetert en dat de blootstelling van werknemers aan tabaksrook sterk daalt. Zo waren in New York City, Ierland, Schotland en Engeland de gemiddelde concentraties fijn stof (PM2.5) in horecagelegenheden na de invoering respectievelijk 6, 9, 10 en 12 keer lager dan daarvoor. In Nederland hebben de Voedsel en Waren Autoriteit (VWA) en het Rijksinstituut voor Volksgezondheid en Milieu (RIVM) hier onderzoek naar gedaan. De concentratie fijn stof (PM2.5) in horecagelegenheden blijkt na invoering van de rookvrije horeca negen keer lager te zijn dan daarvoor. Deze verbetering van de luchtkwaliteit is dus goed vergelijkbaar met die in andere landen met een rookvrije horeca."

In zijn brief aan de Tweede Kamer d.d. 1 december 2009 over de resultaten van één jaar rookvrije horeca schrijft minister Klink over de luchtkwaliteit in de horeca[17]:

"Metingen van het RIVM in opdracht van de VWA laten zien dat de luchtkwaliteit in de horeca na de invoering aanzienlijk is verbeterd. Zo is de concentratie fijn stof na de invoering negen keer lager dan daarvoor. De rookvrije horeca heeft daarmee een positieve invloed gehad op de blootstelling van horecamedewerkers (en secundair bezoekers) aan tabaksrook. Horecawerknemers hebben dus een gezondere werkomgeving gekregen. (p.1)

Het reviewrapport van Regioplan uit oktober 2009 over de resultaten van één jaar rookvrije horeca plaatst bij deze resultaten echter de volgende kritische kanttekening, die minister Klink in zijn brief niet noemt:

"Fijn stof wordt in grote hoeveelheden geproduceerd tijdens het roken en is een belangrijke component van omgevingstabaksrook. Toch is het constateren van deze 'zwevende deeltjes' voor binnenruimten onvoldoende om de relatie met kankergerelateerde risico's vast te stellen. Fijn stof is een van de bestanddelen in omgevingstabaksrook, maar het is nog niet duidelijk of daarmee een representatief beeld wordt gegeven van de toxicologisch relevante stoffen." (p.5)

16. TK, 2008–2009, 22894, nr.206.
17. TK, 2009–2010, 32011, nr.3.

Effecten op de gezondheid (en lichamelijke integriteit) van werknemers
In december 2008 schrijft minister Klink in een brief aan de Tweede Kamer over de effecten van het rookverbod op de gezondheid van werknemers[18]:

> *"Een … afname van de blootstelling aan tabaksrook door de invoering van de rookvrije horeca blijkt ook uit metingen van de concentratie cotinine in lichaamsvloeistoffen (bijvoorbeeld speeksel). Cotinine is een afbraakproduct van nicotine en wordt vaak gebruikt om iemands blootstelling aan tabaksrook vast te stellen. In New York, Schotland en Engeland nam de gemiddelde concentratie cotinine bij niet-rokende werknemers na het rookverbod af met respectievelijk 85, 89 en 76 procent ten opzichte van de concentraties voor het rookverbod. Op basis van de gevonden verbetering in luchtkwaliteit mag verwacht worden dat in Nederland de daling van concentraties cotinine bij niet-rokende horecawerknemers in dezelfde orde van grootte zal liggen als in de genoemde landen. In enkele landen is onderzoek gedaan naar veranderingen in het aantal hartaanvallen onder de bevolking voor en na de invoering van rookverboden. Deze onderzoeken uit de staat New York, Italië en Schotland laten dalingen in het aantal ziekenhuisopnames voor hartaanvallen zien van respectievelijk 8, 11 en 17 procent."* (p.2)

Het reviewrapport van Regioplan uit oktober 2009 stelt over de effecten van het rookverbod in de horeca op de gezondheid van werknemers ondermeer:

> *"Het CPB heeft een poging gedaan om met behulp van verschillende aannames een schatting te maken van de veranderende levensverwachting van horecamedewerkers als gevolg van de invoering van het rookverbod. In onderstaand kader is de berekening van deze schatting weergegeven.*

> ***Levensverwachting van horecamedewerkers***
> *In de horeca werkten in 2004 volgens het CBS 299.000 personen met in totaal 192.000 arbeidsjaren, waarvan 79.000 mannen in arbeidsjaren en 113.000 vrouwen.*
>
> *Van de 104.000 niet-rokers (54% van de horecamedewerkers) werd 69% blootgesteld aan omgevingstabaksrook en van de 88.000 rokers tachtig procent. Voor de Nederlandse horeca betekent dit dat ruim 29.000 niet-rokende mannen na de invoering van het rookverbod gemiddeld 0,19 jaar langer leven, bijna 27.000 rokende mannen leven 0,53 jaar langer en bijna drieduizend mannen stoppen met roken en leven 7,7 jaar langer. Onder de vrouwen leven ruim 42.000 niet-rokers 0,20 jaar langer, ruim 38.000 rokers leven gemiddeld*

18. TK, 2008–2009, 22894, nr.206.

0,39 jaar langer en bij ruim 4300 vrouwen die als gevolg van het rookverbod stoppen, stijgt de levensverwachting met 6,3 jaar gemiddeld.

Hierbij is gebruik gemaakt van gegevens uit de literatuur dat in 2004 van de 192.000 horecamedewerkers (in arbeidsjaren) 41 procent man is, dat 54 procent niet rookt, dat een rookverbod op de werkplek een reductie in het percentage rokers met 3,8 procent teweeg brengt en dat het percentage dat blootgesteld is aan omgevingstabaksrook onder de niet-rokende horecamedewerkers 69 procent bedraagt en onder de rokende tachtig procent."

Strikt geredeneerd neemt de effectiviteit van het rookverbod toe als het rookverbod in de horeca ertoe leidt dat 3,8% van de werknemers stopt met roken. De 'nieuwe' niet-rokende werknemers worden door het rookverbod namelijk ook beschermd tegen de blootstelling aan passief roken in de horeca. Medewerkers in de horeca ertoe overhalen om te stoppen met roken is echter geen doelstelling van het rookverbod in de horeca. Het instellen van een rookverbod in de horeca om medewerkers in de horeca over te halen om te stoppen met roken, zou men als te betuttelend (te paternalistisch) kunnen zien. Het is daarom discutabel in hoeverre de bescherming van de genoemde 'nieuwe' niet-rokende werknemers meegerekend mag worden bij de bepaling van de effectiviteit van het rookverbod in de horeca.

In zijn brief aan de Tweede Kamer d.d. 1 december 2009 over de resultaten van één jaar rookvrije horeca schrijft minister Klink over de (lange-termijn)effecten op de gezondheid van werknemers (en bezoekers)[19]:

> *De daadwerkelijke (lange termijn) effecten op de gezondheid van werknemers (en bezoekers) zijn slechts één jaar na invoering lastig vast te stellen. In het buitenland zijn positieve effecten gerapporteerd op het aantal (ziekenhuisopnames voor) hartaanvallen: zo daalden deze in Schotland na de invoering van «smoke free public places» met zo'n 17%. Voor de Nederlandse situatie zijn hierover geen specifieke cijfers beschikbaar."* (p.1-2)

Op 2 december 2009 schrijft minister Klink in een brief met antwoorden op Kamervragen aan de Tweede Kamer[20]:

> *"[Vraag] Is bekend of de invoering van het rookverbod in de horeca effect heeft gehad op de algemene gezondheid of het zorggebruik? [Antwoord] Uit metingen van het*

19. TK, 2009–2010, 32011, nr.3.
20. TK, 2009–2010, 32011, nr.4.

RIVM in opdracht van de VWA is gebleken dat de luchtkwaliteit in de horeca na de invoering van het rookverbod aanmerkelijk is verbeterd … Het Centraal Plan Bureau heeft gecalculeerd dat de levensverwachting voor zowel de rokende als niet-rokende werknemer in de horeca gestegen is. Er is inmiddels veel Europees onderzoek naar de gezondheidseffecten van het rookverbod, maar deze onderzoeken zijn soms lastig te vergelijken, omdat in veel landen het rookverbod in de horeca gelijktijdig werd ingevoerd met de rookvrije werkplek en een rookverbod in publieke gebouwen. Op basis van onderzoeken in binnen- en buitenland kan voorzichtig geconcludeerd worden dat een rookverbod in de horeca de levensverwachting verlengt en het aantal hart- en vaatziekten vermindert. De daadwerkelijke langetermijneffecten op de gezondheid en het zorggebruik van (niet-)rokende werknemers en bezoekers kunnen pas op langere termijn na de invoering worden bepaald." (p.5-6)

Effecten op keuzevrijheid van werknemers en hinder en overlast voor werknemers
Over de effecten van het rookverbod in de horeca op de keuzevrijheid van werknemers om daar te werken waar ze willen, en op de hinder en overlast die werknemers ondervinden ten gevolge van passief roken (zie overzicht 19 in §11.2.3), heb ik in de door mij bestudeerde parlementaire documenten geen informatie gevonden. Uiteraard geven de naleving van het rookverbod en de effecten van het rookverbod op de luchtkwaliteit en op de gezondheid van werknemers wel een indicatie van de effecten op de keuzevrijheid van werknemers en de hinder en overlast voor werknemers.

Niet-lineaire dosis–effectrelatie
Bij een preventiemaatregel die tot doel heeft om schade, hinder en overlast ten gevolge van passief roken te voorkomen, vallen procesindicatoren (er wordt niet gerookt) en uitkomstindicatoren (er treedt geen schade, hinder en overlast op ten gevolge van passief roken) ongeveer samen. Dat maakt het relatief eenvoudig om de effectiviteit van een rookverbod vast te stellen. Door de niet-lineaire dosis-effectrelatie tussen de blootstelling aan passief roken en de risico's op gezondheidsschade, is het echter onjuist om de daling in de blootstelling aan passief roken gelijk te stellen aan de afname van gezondheidsrisico's. Het Groenboek 'Op weg naar een rookvrij Europa' van de Europese Commissie uit 2007 stelt, mede op basis van het rapport van de Surgeon General uit 2006 (USDHHS), in dit verband:

"De dosis-effectrelatie voor hartziekten is niet-lineair. Iemand die passief rookt heeft maar liefst bijna half zoveel risico een hartziekte te krijgen als iemand die twintig sigaretten per dag rookt. Ook heel kleine doses tabaksrook kunnen een onmiddellijk effect hebben op het ontstaan van stremmingen in de bloedsomloop en trombose, alsmede een langetermijneffect op de ontwikkeling van arteriosclerosis – allemaal belangrijke factoren bij hartziekten. … Er is geen veilig niveau van blootstelling aan

omgevingstabaksrook geconstateerd, en naar verwachting zal verder onderzoek een dergelijk niveau ook niet weten te identificeren."

Alleen een volledig rookverbod geeft de zekerheid dat werknemers in de horeca geen gezondheidsrisico's ten gevolge van passief roken op hun werk lopen. Vanuit dit perspectief is het belangrijk dat 'slechts' 27% van de horecagelegenheden over een rookruimte binnen beschikt (38% van 72% is 27%)[21]. Rookruimten leiden immers tot zwerfrook in de rookvrije ruimten en ondermijnen daarmee de effectiviteit van het rookverbod.

Analyse van de effectiviteit van het rookverbod

In de eerste plaats valt op dat alleen voor de gezondheidseffecten vele pogingen zijn ondernomen om deze te kwantificeren. Voor zover mij bekend is niet geprobeerd de effectiviteit van het rookverbod in de horeca, als instrument om de keuzevrijheid van werknemers om daar te werken waar zij willen te verbeteren, en om de hinder en overlast voor werknemers ten gevolge van passief roken te verminderen, te kwantificeren. Dit had gekund (en kan nog steeds) door gebruik te maken van benaderingen zoals 'Willingness to Accept' (WTA) en 'Willingness to Pay' (WTP).

Wat verder opvalt, is dat het rookverbod in de horeca niet is ontworpen op basis van de op dit moment beschikbare wetenschappelijke kennis, maar dat het in een periode van ruim twintig jaar is gegroeid tot wat het nu is (zie §10.3). Eenmaal genomen besluiten, zoals het toestaan van rookruimten, kunnen moeilijk worden teruggedraaid. Dit betekent dat de vormgeving van het rookverbod niet steeds wordt aangepast aan de laatste stand van de wetenschap. De ministers van VWS – Hoogervorst en Klink – die aan de totstandkoming van het rookverbod in de horeca hebben gewerkt, hebben voortgebouwd op en/of hebben tenminste te maken gehad met besluiten over rookverboden van hun voorgangers.

Rookruimten in niet-horeca-bedrijven, die in 2004 zijn toegestaan bij de invoering van het recht op een rookvrije werkplek, liggen vaak ver af van de werkkamers van het personeel. Ik vermoed dat deze rookruimten in de praktijk tot minder zwerfrook in de beoogde rookvrije ruimten (de werkvloer en werkkamers) leiden dan rookruimten in de horeca. Ook maakt het verschil of rookruimten worden ingesteld voor werknemers (die gedurende een hele werkdag in een gebouw verblijven) of voor bezoekers (die een kortere tijd in een horecagelegenheid verblijven). Het is opmerkelijk dat deze argumenten in het wetenschappelijke en politieke debat over het rookverbod in de horeca niet zijn aangevoerd en meegewogen. In het politieke

21. Zie het laatste citaat onder het kopje '*Al dan niet toestaan van rookruimten*'.

debat is het toestaan van rookruimten in de horeca onterecht niet beargumenteerd en onterecht niet ter discussie gesteld.

Het besluit tot invoering van een rookverbod in de horeca is in 2007 genomen door minister Klink, als het laatste in een rij van rookverboden. Met dit besluit bouwde minister Klink voort op de zelfregulering van het rookbeleid in de horeca die minister Hoogervorst met KHN overeen was gekomen, en op het recht op een rookvrije werkplek waartoe minister Borst-Eilers had besloten. Toch worden besluiten uit het verleden over de vormgeving van rookverboden soms wel veranderd of teruggedraaid. In 1998 heeft minister Borst-Eilers de dan geldende rookverboden aangescherpt door het aantal plaatsen waar mag worden gerookt, in gebouwen waar een rookverbod geldt, te beperken. In 2011 heeft minister Schippers besloten om het rookverbod in de horeca te versoepelen, door kleine cafés zonder personeel vrij te stellen van het rookverbod.

Als men ten tijde van de besluitvorming over de eerste wettelijke rookverboden (eind jaren '80) had beschikt over de huidige wetenschappelijke kennis over de risico's van zwerfrook vanuit rookruimten, vermoed ik dat aan rookruimten stringente eisen gesteld zouden worden, of dat rookruimten in gebouwen waar een rookverbod geldt, verboden zouden worden. Toch zijn er ook argumenten die pleiten voor het instellen van rookruimten. In de eerste plaats het recht van rokers om te roken, als zij daarmee geen hinder of overlast veroorzaken. In de tweede plaats kan een rookruimte mogelijk een deel van de (vermeende) economische schade voorkomen die uitbaters van horecagelegenheden van een rookverbod verwachten, omdat het horecabezoek zou kunnen teruglopen. Ten slotte, en dat is in de praktijk misschien het belangrijkst, kan het toestaan van rookruimten het draagvlak voor rookverboden onder de bevolking, de naleving van rookverboden door rokers en de handhaving van rookverboden door personeel en bedrijfseigenaars gunstig beïnvloeden. Daarom zou het toestaan van rookruimten per saldo gunstig kunnen zijn voor de effectiviteit van rookverboden. Hoewel rookruimten in beperkte mate tot zwerfrook in beoogde rookvrije ruimten kunnen leiden, en werknemers rookruimten soms moeten betreden, kan daarom niet worden geconcludeerd, dat het toestaan van rookruimten afbreuk doet aan de effectiviteit van rookverboden in het algemeen en het rookverbod in de horeca in het bijzonder.

Als de keus moet worden gemaakt tussen enerzijds het toestaan van rookruimten en anderzijds het toestaan van ventilatiesystemen in plaats van een rookverbod, is (bij de huidige stand van de techniek) de keuze voor rookruimten het best te rechtvaardigen. Uit diverse wetenschappelijke onderzoeken blijkt dat in de praktijk de kans op zwerfrook in beoogde rookvrije ruimten bij het gebruik van een ventilatie-

systeem beduidend groter is dan bij het gebruik van een rookruimte. In de praktijk zijn rookruimten effectiever dan ventilatiesystemen in het voorkomen van de blootstelling van werknemers aan tabaksrook, omdat het gebruik minder complex is en minder snel fout gaat.

Van 2009 tot op heden (juni 2011) concentreert het politieke en maatschappelijke debat zich op de naleving van het rookverbod, die met name in kleine cafés zonder personeel onder druk staat. Als het rookverbod in kleine cafés zonder personeel niet wordt nageleefd, heeft dat echter geen negatieve invloed op de bescherming van werknemers in de horeca tegen passief roken. De beoogde effecten (= de effectiviteit) van het rookverbod worden dan niet negatief beïnvloed (de bescherming van eigenaars of bezoekers van cafés tegen passief roken zijn geen beoogde effecten, maar gunstige neveneffecten). De naleving van het rookverbod is 'slechts' een indicator van de effectiviteit. Naleving zegt veel over de blootstelling aan tabaksrook, maar minder over gezondheidswinst. Als er een niet-lineaire dosis-effect-relatie bestaat tussen de blootstelling aan tabaksrook en het risico op gezondheidsschade (zoals bij hart- en vaatziekten het geval is), kan de naleving een (sterk) vertekend beeld geven van de effectiviteit van het rookverbod. In dat geval geeft naleving van het rookverbod in 90% van de tijd een reductie van het risico op gezondheidsschade van veel minder dan 90%.

In het algemeen zijn de effecten van een rookverbod duurzaam. Naarmate de acceptatie en daarmee de handhaving van het rookverbod met de tijd toeneemt, neemt ook de effectiviteit toe (het omgekeerde kan natuurlijk ook het geval zijn). In dit verband is het van belang dat het rookverbod geen geïsoleerde activiteit is, maar onderdeel uitmaakt van een groot aantal preventiemaatregelen in het kader van het tabaksontmoedigingsbeleid van de overheid.

12.1.3 Eindoordeel (criterium effectiviteit)

Er zijn veel pogingen ondernomen om de gezondheidseffecten van het rookverbod in de horeca te kwantificeren. Voor zover mij bekend is (onterecht) niet geprobeerd de effectiviteit van het rookverbod in de horeca, als instrument om de keuzevrijheid van werknemers te verbeteren en om de hinder en overlast voor werknemers te verminderen, te kwantificeren.

Opvallend, maar begrijpelijk, is dat de vormgeving van het rookverbod in de horeca niet steeds wordt aangepast aan de laatste stand van de wetenschap. Voor de preventie van aandoeningen die werknemers ten gevolge van passief roken kunnen oplopen, wordt dit blijkbaar aanvaardbaar geacht, terwijl dit voor de behande-

ling van deze aandoeningen niet aanvaardbaar wordt geacht. Op basis van de wetenschappelijke onderzoeken die hiernaar zijn uitgevoerd kan m.i. worden geconcludeerd dat het rookverbod in de horeca effectief is.

In de praktijk wordt de effectiviteit van het rookverbod in de horeca het meest bedreigd doordat het rookverbod te weinig wordt nageleefd. In 2011 geldt dit met name voor cafés en discotheken. Zelfs met het huidige niveau van naleving is het rookverbod in de horeca m.i. nog steeds effectief, omdat heel veel werknemers in de horeca wel worden beschermd tegen de blootstelling aan passief roken. Wel dient de naleving c.q. handhaving in met name cafés en discotheken te worden verbeterd. In februari 2011 heeft minister Schippers aangekondigd hiertoe maatregel te gaan treffen[22].

Op termijn dient de effectiviteit van het rookverbod in de horeca te worden verbeterd door eisen te stellen aan de effectiviteit van rookruimten. Verbetering van de naleving heeft echter meer prioriteit, omdat de huidige gebrekkige naleving in cafés en discotheken een grotere bedreiging vormt voor de effectiviteit van het rookverbod en zelfs de houdbaarheid van het rookverbod in de horeca (met name in cafés en discotheken) onder druk zet.

12.1.4 Gevolgen van de beoordeling voor het model (criterium effectiviteit)

Naar aanleiding van de beoordeling aan de hand van het criterium ‘effectiviteit’ heb ik de definitie van het criterium ‘effectiviteit’ aangepast. De nieuwe definitie luidt:

> *“De effectiviteit betreft de beoogde effecten van de preventiemaatregel op de doelgroep <u>of op derden</u>. De doelgroep is hierbij gedefinieerd als de groep personen waarvan men de leefstijl tracht te beïnvloeden ter voorkoming van leefstijlgerelateerde aandoeningen bij de doelgroep zelf <u>of bij derden</u>.”*

Het aspect ‘naleving van de preventiemaatregel’ komt bij verschillende criteria van het model aan bod. Naar aanleiding van de beoordeling aan de hand van het criterium ‘effectiviteit’ heb ik m.b.t. het aspect ‘naleving’ het volgende onderscheid gemaakt tussen de criteria ‘effectiviteit, draagvlak en handhaafbaarheid’. Bij het criterium ‘effectiviteit’ wordt ingegaan op de mate waarin de preventiematregel wordt nageleefd. Bij de criteria ‘draagvlak’ en ‘handhaafbaarheid’ wordt ingegaan

22. TK, 2010–2011, 32011, nr.15, p.20-21.

op de oorzaken van de mate van naleving, respectievelijk het draagvlak voor en de handhaving van de preventiemaatregel.

12.2 Achtste criterium: indringendheid

12.2.1 Operationalisering en wijze van beoordeling

Het criterium 'indringendheid' betreft de verhouding tussen de effectiviteit en de indringendheid van de preventiemaatregel. De indringendheid van de preventiemaatregel betreft de inbreuk van de preventiemaatregel op het persoonlijk leven van mensen. De indringendheid wordt in drie stappen beoordeeld (zie overzicht 12 in §7.2.1):

I. Algemene beoordeling van de indringendheid:
   1. Bij wie wordt een inbreuk gemaakt op het persoonlijk leven?
   2. Door wie wordt een inbreuk gemaakt op het persoonlijk leven?
   3. In welke mate wordt een inbreuk gemaakt op het persoonlijk leven?
   4. Welke reikwijdte heeft de inbreuk op het persoonlijk leven?
   5. Leidt het toestaan van de inbreuk op het persoonlijk leven tot precedentwerking?
   6. Hoe bezwaarlijk is de inbreuk op het persoonlijk leven?

II. Beoordeling van verschillende aspecten van de indringendheid:
   a. aantasting van de lichamelijke integriteit;
   b. aantasting van de vrijheid;
   c. aantasting van de privacy;
   d. aantasting van de veiligheidsbeleving;
   e. discriminatie;
   f. stigmatisering.

III. Beoordeling van de mate van drang of dwang die wordt toegepast.

Bij stap II kan afhankelijk van de betreffende preventiemaatregel per aspect (a t/m f) worden besloten om, al dan niet en meer of minder uitgebreid, op de zes vragen (1 t/m 6) in te gaan.

De preventiemaatregel passeert het criterium 'indringendheid', als niet kan worden aangetoond dat de verhouding tussen de effectiviteit en indringendheid onaanvaardbaar is.

12.2.2 Beoordeling van casus 'wettelijk rookverbod in horeca' aan de hand van criterium 'indringendheid'

Stap I: algemene beoordeling gegeven van de indringendheid

Bij wie wordt een inbreuk gemaakt op het persoonlijk leven?
Met het rookverbod in de horeca wordt een inbreuk gemaakt op het persoonlijk leven van rokers in de horeca: bezoekers, werknemers en eigenaars van horecagelegenheden.

Door wie wordt een inbreuk gemaakt op het persoonlijk leven?
Door de rijksoverheid die het wettelijke rookverbod in de horeca heeft vastgesteld.

In welke mate wordt een inbreuk gemaakt op het persoonlijk leven?
Deze vraag bespreek ik onder stap II.

Welke reikwijdte heeft de inbreuk op het persoonlijk leven?
De inbreuk op de individuele vrijheid van rokers ten gevolge van het wettelijk rookverbod in de horeca is locatiegebonden. Direct buiten de horecagelegenheid en in een eventuele rookruimte mag wel worden gerookt. Daardoor is een rookverbod in de horeca veel minder indringend dan een algeheel rookverbod.

Leidt het toestaan van de inbreuk op het persoonlijk leven tot precedentwerking?
Van het rookverbod in de horeca wordt geen precedentwerking verwacht, omdat dit rookverbod het sluitstuk vormt van de invoering van de rookvrije werkplek, die buiten de horeca al in 2004 heeft plaatsgevonden.

Hoe bezwaarlijk is de inbreuk op het persoonlijk leven?
Deze vraag bespreek ik onder stap II.

Stap II: beoordeling van verschillende aspecten van de indringendheid

De rookverboden in het kader van de Tabakswet leiden niet tot:
- aantasting van de lichamelijke integriteit;
- aantasting van de privacy;
- aantasting van de veiligheidsbeleving;
- discriminatie.

De rookverboden in het kader van de Tabakswet leiden wel tot:
- aantasting van de vrijheid;
- stigmatisering.

Daarom zal ik deze twee aspecten van indringendheid uitwerken.

Aantasting van de vrijheid
Met het rookverbod in de horeca wordt een inbreuk gemaakt op de vrijheid van rokers in de horeca. Rokers zouden dit als een grote inbreuk kunnen ervaren, omdat roken verslavend is en zij daarom naar het roken kunnen hunkeren. Minister Borst-Eilers relativeert in het onderstaande citaat de inbreuk die (ondermeer) een rookverbod op de vrijheid van rokers maakt. In mei 2000 schrijft minister Borst-Eilers over haar Wetsvoorstel tot wijziging van de Tabakswet (waarin ondermeer het recht op een rookvrije werkplek wordt voorgesteld)[23]:

> *"De regering deelt niet de inschatting van deze leden [Groen Links] dat de huidige rokers deze maatregelen als een inbreuk op hun vrijheid zullen ervaren. Immers, een algemeen aanvaard principe is toch dat de vrijheid van de een (van de roker om te roken) eindigt waar die van de ander (van de niet-roker met het recht op schone lucht) begint. Wel moet worden erkend dat het alle rokers niet altijd lukt dit principe overal in de praktijk te brengen, want rokers zijn nu eenmaal verslaafd aan tabak. De regering onderschrijft de opvatting van de leden van de fractie van GroenLinks dat de overheid inbreuk mag maken op de individuele vrijheid als daardoor het algemeen belang overtuigend gediend wordt. Gezien de bewijzen van de negatieve invloed van roken op de gezondheid, bewijzen die in de laatste dertig jaar steeds talrijker en sterker zijn geworden, is het goed om te constateren dat de leden van de fractie van GroenLinks menen dat hier inderdaad sprake is van bevordering van het algemeen belang, waarvoor de individuele keuzevrijheid mag worden ingeperkt. Ik wil hier nog aan toevoegen dat er bij passief roken sprake is van (potentiële) schade en hinder of overlast voor derden; ook dat is een algemene grond voor overheidsinterventie. Bovendien gaat het in dit wetsvoorstel óók om de bescherming van minderjarigen, wederom een algemeen aanvaarde legitimatiegrond voor ingrijpen door de Staat."* (p.4)

Interessant is dat minister Borst-Eilers onderscheid maakt tussen enerzijds de inbreuk op hun vrijheid die rokers ervaren (ondermeer ten gevolge van het rookverbod op de werkplek) en anderzijds de feitelijke inbreuk op de individuele vrijheid die een rookverbod vormt. Volgens minister Borst-Eilers ervaren rokers een

23. TK, 1999-2000, 26472, nr.6.

rookverbod niet als een inbreuk op hun vrijheid, als zij door te roken (risico op) schade, hinder of overlast voor derden veroorzaken.

In maart 2002 stelt Eerste Kamerlid Van Heukelum bij de bespreking van het Wetsvoorstel tot wijziging van de Tabakswet in de Eerste Kamer[24]:

> *"Ik wil niet verhelen dat binnen mijn fractie geworsteld wordt met dit wetsvoorstel. … waar het bij mijn fractie ten diepste om gaat, is de vraag hoe ver de rol en de verantwoordelijkheid van de overheid gaat. Hoe verhouden die rol en de verantwoordelijkheid van de overheid zich tot de vrijheid en de verantwoordelijkheid van de individuele burger. Wij leven in een vrij land en tabak is nog steeds een legaal product. Als burgers daarvan gebruik willen maken, is dat hun volste recht. Zij hebben de vrijheid, wel of niet te roken. Maar een rotsvast liberaal adagium is ook dat de vrijheid van het ene individu ophoudt waar de onvrijheid van de ander begint. Mijn fractie kan dan ook alleszins instemmen met het geformuleerde doel van het wetsvoorstel, te weten het beschermen van de niet-roker. … Maar de in dit wetsvoorstel voorgestelde verscheidene maatregelen vergen wel een uiterst zorgvuldige afweging tussen bescherming van maatschappelijke belangen en volksgezondheidsbelangen enerzijds en vrijheid van consumptie en eigen verantwoordelijkheid anderzijds. Uiteraard heeft de overheid in dit geheel een belangrijke taak, maar zij moet door middel van haar maatregelen niet verder willen springen dan haar polsstok lang is."*
> (p.1252)

Eerste Kamerlid Van Heukelum geeft aan dat zijn partij (VVD) worstelt met de rookverboden die minister Borst-Eilers voorstelt. De punten waarmee zijn partij worstelt, zijn (1) de inbreuk op de vrijheid van consumptie en (2) de inbreuk op de eigen verantwoordelijkheid van rokers (en niet rokers) die een rookverbod vormt. De eigen verantwoordelijkheid waarover de heer Van Heukelum spreekt, betreft volgens mij de vraag of rokers en niet-rokers dit niet zelf onderling kunnen regelen. In de praktijk is dat volgens minister Borst-Eilers en later volgens de ministers Hoogervorst en Klink niet het geval. Dit citaat van de heer Van Heukelum bevestigt mijn indruk dat de indringendheid van het rookverbod wordt bepaald door de inbreuk op de vrijheid van consumptie en de inbreuk op de eigen verantwoordelijkheid van rokers, om niet te roken als anderen daar schade, hinder of overlast van kunnen ondervinden.

De inbreuk op de keuzevrijheid en eigen verantwoordelijkheid van rokers acht ik niet groot, want als rokers hun eigen verantwoordelijkheid zouden nemen wat betreft de bescherming van de gezondheid van derden (bezoekers, werknemers en

24. EK, 2001-2002, vergaderingnr.24, p.1236-1275 (dossiernr.26472).

eigenaars van horecagelegenheden) en het voorkomen van hinder en overlast voor derden, zouden zij feitelijk nauwelijks ergens in de horeca kunnen roken. Tabaksrook blijft immers lang in ruimten hangen en ook geringe concentraties tabaksrook zijn schadelijk. Bezoekers, werknemers en eigenaars kunnen schade, hinder en overlast van roken ondervinden, zonder dat zij hiertegen adequaat kunnen of durven optreden. Bovendien kan op ieder moment een niet-rokende bezoeker binnenkomen.

Het gegeven dat roken verslavend is, kan maken dat rokers het rookverbod in de horeca als zwaar ervaren. Als rokers het rookverbod in de horeca ten gevolge van hun verslaving als zwaar ervaren, vind ik dat geen reden om deze inbreuk op hun keuzevrijheid en eigen verantwoordelijkheid, om die reden, als bezwaarlijk te bestempelen. Het is immers niet zo dat men wel schade, hinder of overlast voor anderen mag veroorzaken, als men verslaafd is.

Eigenaars van horecagelegenheden zonder personeel zouden kunnen stellen dat een wettelijk rookverbod in hun eigen zaak een grote inbreuk is op hun keuzevrijheid en eigen verantwoordelijkheid. Uit de door mij bestudeerde parlementaire documenten komt niet naar voren dat dit in de praktijk een rol van betekenis speelt. Eigenaars van horecagelegenheden zonder personeel protesteren weliswaar heftig tegen een wettelijk rookverbod in hun zaak. De reden die zij aanvoeren is echter niet de inbreuk op hun persoonlijk leven (b.v. dat zij zelf graag in hun eigen zaak willen roken), maar vermeende omzetdaling ten gevolge van een rookverbod.

Stigmatisering
Al in 2000 heeft minister Borst-Eilers besloten om werkgevers verantwoordelijk te maken voor de handhaving van de rookvrije werkplek en om niet de roker maar de werkgever te beboeten, als het recht op de rookvrije werkplek wordt overtreden. Een van de redenen is dat zij niet wil dat rokers gecriminaliseerd c.q. gestigmatiseerd worden[25]:

> *"In het licht van de eigen verantwoordelijkheid van de roker vragen de leden van de fractie van de VVD de regering om nog eens in het algemeen een onderbouwing te geven van de reden waarom alleen de verkoper van tabaksproducten (en niet ook de jongere onder de 18 jaar) en uitsluitend de beheerder van een openbaar gebouw waar een rookverbod geldt (en niet ook de roker aldaar) met sancties wordt aangepakt. Het antwoord hierop is dat de regering niet wil dat groepen jongeren en rokers «gecriminaliseerd » zouden worden.* (p.56)

25. TK, 1999–2000, 26472, nr.6.

In een debat in de tweede kamer over de invoering van het wettelijk rookverbod in de horeca wordt opnieuw gesteld dat rokers niet gecriminaliseerd c.q. gestigmatiseerd mogen worden[26]:

> *"Mevrouw Bouwmeester (PvdA): … Mevrouw Bouwmeester vindt dat de uitbater strafbaar gesteld moet worden als hij de regels overtreedt, maar de horeca wil de rokers zelf strafbaar stellen. Dat criminaliseert de rokers en dat is niet de bedoeling…."* (p.1)

Stap III: beoordeling van de mate van drang of dwang die wordt toegepast.

De mate van dwang die wordt toegepast is groot. Er is sprake van intensieve inspectie door de Voedsel en Warenautoriteit, boetes en de mogelijkheid tot stillegging van het horecabedrijf[27].

12.2.3 Eindoordeel (criterium indringendheid)

Roken vertegenwoordigt geen kwaadaardig belang

In bijlage 5 heb ik beschreven dat kwaadaardige belangen volgens Feinberg geen basisbelangen zijn in de zin van het schadebeginsel. Kwaadaardige belangen (bv. de wens om iemand te vermoorden) zijn niet te billijken en kunnen daarom niet als basisbelang worden aanvaard. Een aantasting van kwaadaardig belangen (bv. het voorkomen van een moord) mag daarom niet als schade (aan een basisbelang van de potentiële moordenaar) worden opgevat. Een inbreuk op het persoonlijk leven van mensen, die leidt tot de aantasting van een kwaadaardig belang (iemand verhinderen om een moord te plegen), valt daarom niet onder het criterium 'indringendheid' van het model. De vraag is nu in hoeverre roken in een openbare ruimte, zoals de horeca, als een kwaadaardig belang kan worden opgevat. Roken kan immers ernstige schade toebrengen aan derden en hinder en overlast veroorzaken. Passief roken kan dodelijk zijn en tot ernstige aandoeningen leiden (zie §11.1).

Een essentieel verschil tussen een moordenaar en een roker is, dat een moordenaar de intentie heeft (gehad) om een ander schade toe te brengen en een roker niet. De vraag is nu of alleen de intentie bepalend is voor de beantwoording van de vraag of een belang kwaadaardig is, of dat ook gedrag dat onbedoeld tot schade, hinder of overlast leidt een kwaadaardig belang kan vertegenwoordigen. Als iemand er een

26. TK, 2006–2007, 30800 XVI, nr.182.
27. TK, 2008–2009, 22894, nr.206.

kick van krijgt om in Nederland met 160 km per uur met een auto op de snelweg te racen, zonder dat hij de intentie heeft om anderen letsel toe te brengen, is er sprake van roekeloosheid, maar niet van kwaadaardigheid (zijn aard is niet kwaad; hij wil geen letsel toebrengen). De redenering, dat gedrag dat onbedoeld tot schade, hinder of overlast leidt een kwaadaardig belang kan vertegenwoordigen, gaat daarom mank. Net zoals zonder voorbedachte rade geen sprake is van moord, is zonder de intentie om schade toe te brengen m.i. geen sprake van een kwaadaardig belang. Wel kan er sprake zijn van verwijtbare roekeloosheid, maar dat is iets anders dan kwaadaardige opzet.

In 1984 stellen de staatssecretarissen Van der Reijden van WVC en Van Zeil van EZ in de memorie van toelichting bij het wetvoorstel betreffende 'maatregelen ter beperking van het tabaksverbruik in het bijzonder ter bescherming van de niet-roker' (zie §10.3):

> *"…Daar komt bij dat het recht op eerbiediging van de persoonlijke levenssfeer, zoals neergelegd in artikel 10, eerste lid, van de Grondwet, met zich brengt dat de overheid terughoudendheid moet betrachten bij het stellen van regels ter bescherming van de niet-roker. Zoals de niet-roker het recht heeft op bescherming van zijn lichamelijke integriteit heeft de roker er recht op dat overheidsbemoeienis op dit punt zich niet uitstrekt tot zijn persoonlijke levenssfeer. Dit betekent dat de overheid zich heeft te onthouden van het stellen van regels ten aanzien van het roken in de privésfeer met het oogmerk de niet-roker te beschermen."* (p.12)

In 2010 stelt minister Schippers in het ontwerpbesluit ter versoepeling van het rookverbod in kleine cafés zonder personeel (zie §10.3):

> *"Met behoud van de hoofdregel van de rookvrije werkplek voor werknemers in de horeca wordt aldus bewerkstelligd dat in (zeer) kleine buurtkroegjes, waar de kroeg een sociale functie vervult vergelijkbaar met die van de huiskamer in een huisgezin, roken wel mogelijk wordt gemaakt. Met het terugtreden van de regulering voor deze ruimten versterkt de wetgever de gedachte dat roken in de huiskamer niet een aangelegenheid is waar de overheid zich mee bemoeit. Het is een zaak voor de uitbater zelf, die zich daarbij ongetwijfeld mede zal laten leiden door datgene wat de klanten daarover te berde brengen."* (p.5)

Op basis van de bovenstaande citaten concludeer ik dat de overheid een wettelijk rookverbod in de privésfeer zeer indringend vindt. Blijkbaar vindt de overheid dat een rookverbod in de privésfeer inbreuk maakt op een basisbelang van de roker. Dit zou betekenen dat roken in de thuissituatie geen kwaadaardig belang vertegenwoordigt; ook niet als roken in de thuissituatie leidt tot risico op schade, hinder of overlast bij derden (zoals de partner of kinderen van de roker).

Op basis van de bovenstaande overwegingen kom ik tot de volgende conclusies. Gedrag dat wordt gedreven door de ***intentie*** om een ander schade toe te brengen, vertegenwoordigt een kwaadaardig belang. Een inperking of verbod van dit gedrag valt niet onder het criterium indringendheid van het model. Gedrag dat een ander ***onbedoeld*** schade kan toebrengen, zoals roken, vertegenwoordigt geen kwaadaardig belang. Een inperking of verbod van dit gedrag valt wel onder het criterium indringendheid van het model. In dat geval moet de ***belangenafweging*** worden gemaakt tussen enerzijds het recht van het individu (de roker) dat geen inbreuk wordt gemaakt op zijn persoonlijk leven en anderzijds het recht van anderen ('meerokers') op bescherming tegen schade, hinder en overlast.

Aantasting van de vrijheid
Opmerkelijk is dat de aantasting van de vrijheid van rokers ten gevolge van het rookverbod (in de horeca) niet wetenschappelijk is onderzocht. Wel lijkt brede politieke consensus te bestaan dat het belang van werknemers om te worden beschermd tegen passief roken in de horeca veel zwaarder weegt dan het belang van rokers om te roken in de horeca. Ik ben het daarmee eens. Daarbij weeg ik mee dat de reikwijdte van het rookverbod in de horeca beperkt is tot de horecagelegenheid. Rokers in de horeca mogen in een rookruimte en direct buiten de horecagelegenheid wel roken (mits het terras niet teveel is afgesloten). Eigenaars van kleine cafés zonder personeel, die tijdens werktijd niet mogen roken in hun eigen zaak, zouden dit als een grote inbreuk op hun keuzevrijheid en eigen verantwoordelijkheid kunnen ervaren. Het lijkt mij dat zij moeilijk af en toe weg kunnen lopen om bijvoorbeeld buiten een sigaretje te roken. In de parlementaire verslaglegging van het debat over de problemen die kleine cafés zonder personeel met het rookverbod hebben, wordt dit argument echter niet aangevoerd.

Stigmatisering
Opmerkelijk is dat de stigmatisering van rokers ten gevolge van het rookverbod (in de horeca) niet wetenschappelijk is onderzocht. De overheid heeft onderkend dat een rookverbod op de werkplek stigmatiserend kan zijn. Daarom heeft de wetgever werkgevers verantwoordelijk gemaakt voor de handhaving van het rookverbod op de werkplek en worden niet de rokers, maar de werkgever beboet bij overtreding van het rookverbod. De kritiek die ik in parlementaire documenten heb gevonden op het wettelijk rookverbod in de horeca betrof niet de stigmatisering van de rokers, maar de vermeende negatieve economische effecten.

Dwang
De mate van dwang die wordt toegepast is groot. Er is sprake van intensieve inspectie, boetes en de mogelijkheid tot stillegging van het horecabedrijf. De boetes

worden opgelegd aan het bedrijf en niet aan natuurlijke personen en vormen om die reden geen inbreuk op het persoonlijk leven.

Samenvattend
De indringendheid van het rookverbod in de horeca wordt bepaald door de inbreuk van het rookverbod op de vrijheid van consumptie en eigen verantwoordelijkheid van rokers. Bij de beoordeling van de gerechtvaardigdheid van het rookverbod in de horeca aan de hand van het criterium 'indringendheid' moet een afweging worden gemaakt tussen enerzijds het belang van bescherming van werknemers tegen passief roken en anderzijds het belang van rokers om te roken. Binnen de politiek bestaat consensus dat de bescherming van werknemers het zwaarst weegt.

12.2.4 Gevolgen van de beoordeling voor het model (criterium indringendheid)

De beoordeling van het wettelijk rookverbod in de horeca aan de hand van het criterium 'indringendheid' heeft geleid tot een verheldering van het begrip 'kwaadaardig belang'. Gedrag dat wordt gedreven door de ***intentie*** om een ander schade toe te brengen, vertegenwoordigt een kwaadaardig belang. Een inperking of verbod van dit gedrag valt niet onder het criterium indringendheid van het model. Gedrag dat een ander ***onbedoeld*** schade kan toebrengen vertegenwoordigt geen kwaadaardig belang. Een inperking of verbod van dit gedrag valt wel onder het criterium indringendheid van het model.

12.3 Negende criterium: lasten-batenverhouding

12.3.1 Operationalisering en wijze van beoordeling

Het criterium 'lasten-batenverhouding' betreft de verhouding tussen de lasten en baten van de preventiemaatregel (zie overzicht 20). De kosteneffectiviteit van de preventiemaatregel betreft de verhouding tussen de uitvoeringskosten en de effectiviteit van de preventiemaatregel (zie §7.3.1). Het verdient aanbeveling om de uitvoeringskosten wel afzonderlijk zichtbaar te maken, maar van de kosteneffectiviteit is geen afzonderlijk criterium gemaakt, omdat de kosteneffectiviteit zowel bij de casus 'Clarian health' als bij de casus 'rookverbod in de horeca' van ondergeschikt belang blijkt. De lasten van de preventiemaatregel worden veel meer bepaald door de indringendheid van de preventiemaatregel, en bij het rookverbod in de horeca door (vermeende) economische neveneffecten, dan door de uitvoeringskosten.

Overzicht 20: criterium lasten-batenverhouding

Lasten-batenverhouding	
Lasten van de preventiemaatregel	***Baten van de preventiemaatregel***
Indringendheid van de preventiemaatregel (8e criterium)	Effectiviteit van de preventiemaatregel (7e criterium)
Overige lasten van de preventiemaatregel (inclusief uitvoeringskosten)	Overige baten van de preventiemaatregel (positieve neveneffecten)

Alleen de overige lasten en baten (grijs gearceerd) zijn nog niet geanalyseerd. De indringendheid (8e criterium) en effectiviteit (7e criterium) zijn al geanalyseerd. Als alle lasten en baten op dezelfde wijze worden gekwantificeerd c.q. worden uitgedrukt in geld, kan de lasten-batenverhouding worden berekend door de waarde van alle baten te delen door de waarde van alle lasten. Als de uitkomst van deze deling groter is dan één, dan is de lasten-batenverhouding gunstig. Als deze kleiner is dan één, is toepassing van de preventiemaatregel niet gerechtvaardigd. De duurzaamheid van de baten en lasten vormt een belangrijk aandachtspunt. Ook als het niet lukt om alle lasten en baten te kwantificeren, is het toch nuttig om alle lasten en baten in één overzichtelijk schema te plaatsen (Ogus, 2010).

Overzicht 21: lasten en baten van wettelijk rookverbod in horeca

Lasten van de preventiemaatregel	***Baten van de preventiemaatregel***
Indringendheid van de preventiemaatregel (8e criterium)	Effectiviteit van de preventiemaatregel (7e criterium)
Overige lasten van de preventiemaatregel (negatieve neveneffecten):<br>– Uitvoeringskosten<br>– Omzetdaling van horecabedrijven<br>– Lasten van realiseren van rookvoorzieningen<br>– Overlast door horecabezoekers die buiten staan<br>– Substitutie van roken in horeca naar roken thuis<br>– Ontstaan van verzet tegen wetgeving en agressie tegen wetshandhavers<br>– Afname van de keuzevrijheid van bezoekers	Overige baten van de preventiemaatregel (positieve neveneffecten):<br>– Vermindering van risico op gezondheidsschade bij bezoekers<br>– Vergroting van keuzevrijheid van bezoekers<br>– Vermindering van hinder en overlast voor bezoekers<br>– Bezoekers en werknemers zijn minder gaan roken of gestopt met roken<br>– Omzetstijging van horecabedrijven<br>– Ontstaan van een sociaal klimaat waarin niet-roken de norm is

12.3.2 *Beoordeling van casus 'wettelijk rookverbod in horeca' aan de hand van criterium 'lasten-batenverhouding'*

Overzicht 21 geeft een overzicht van de lasten en baten van het wettelijk rookverbod in de horeca, zoals die uit de door mij bestudeerde parlementaire documenten naar voren komen.

Om tot een beoordeling te komen van de lasten-batenverhouding zal ik de lasten en baten, zoals die uit de door mij bestudeerde parlementaire documenten naar voren komen, eerst achtereenvolgens (zie overzicht 21) bespreken.

Uitvoeringskosten (overige lasten)
In de door mij bestudeerde parlementaire documenten (zie §10.2) heb ik nauwelijks informatie over de uitvoeringskosten en de daaruit voortvloeiende kosteneffectiviteit van het wettelijk rookverbod in de horeca kunnen vinden[28]. Hieronder geef ik een overzicht van de informatie die ik over de uitvoeringskosten en kosteneffectiviteit van (rook)verboden op basis van de Tabakswet heb aangetroffen.

In 2002 schrijft minister Borst-Eilers aan de Eerste Kamer in een 'Nadere memorie van antwoord' op vragen over haar 'Wetsvoorstel tot wijziging van de Tabakswet'[29]:

> *"Wetgeving is en blijft ook een kosteneffectief instrument binnen het totale samenhangende tabaksontmoedigingsbeleid van de regering."* (p.10)

De stelling van minister Borst-Eilers dat wetgeving een kosteneffectief instrument is binnen het totale samenhangende tabaksontmoedigingsbeleid van de regering, wordt door haar echter niet met argumenten of cijfers onderbouwd. Ten tijde van deze uitspraak van minister Borst-Eilers (2002) is er echter nog geen sprake van een wettelijk rookverbod in de horeca.

Uit het onderstaande citaat blijkt dat de Algemene Rekenkamer in 2003 heeft geconstateerd dat voor de beleidsinstrumenten 'wetgeving' en 'handhaven wetgeving' op het gebied van het niet-rokenbeleid geen gegevens over de kosteneffectiviteit beschikbaar waren[30]:

---

28. Kosteneffectiviteit: verhouding tussen de uitvoeringskosten en de effectiviteit van de preventiemaatregel.
29. EK, 2001–2002, 26472, nr.59c.
30. TK, 2003–2004, 29300, nrs. 1–2.

"De Algemene Rekenkamer heeft van oktober 2002 tot en met juli 2003 een onderzoek uitgevoerd naar enkele onderdelen van de preventieve gezondheidszorg. Gekeken is naar het beleid ter bestrijding van: (1) sociaal-economische gezondheidsverschillen, (2) roken en (3) te weinig bewegen." (p.8)

Voor de verwezenlijking van het niet-rokenbeleid van het Ministerie van VWS worden de volgende tien beleidsinstrumenten gehanteerd:

1. *wetgeving;*
2. *handhaven wetgeving;*
3. *massamediale campagnes;*
4. *marketingexperiment niet-roken;*
5. *subsidies voor ontwikkeling voorlichtingsmaterialen;*
6. *ondersteuning bij stoppen voor verslaafde rokers;*
7. *adequate zelfregulering;*
8. *accijnsverhoging;*
9. *internationale initiatieven;*
10. *aansturing van onderzoek, instrumentontwikkeling en bevorderen van toepassing van instrumenten.*

De Algemene Rekenkamer heeft de tien beleidsinstrumenten aan de normen voor de beleidsinrichting getoetst. Deze normen houden in dat keuze van de beleidsinstrumenten onderbouwd moet zijn (op basis van een probleemanalyse, beleidstheorie, verwachte effecten en kosteneffectiviteit) en dat de taakverdeling tussen actoren duidelijk omschreven moet zijn. …

Gegevens over kosteneffectiviteit zijn beschikbaar voor de massamediale campagnes, het marketingexperiment niet-roken en de ondersteuning voor stoppen bij verslaafde rokers." (p.27)

juli 2007 verschijnt de eindrapportage 'Gezond gedrag bevordert' van de werkgroep 'IBO preventie'. In deze rapportage stelt de werkgroep (IBO, 2007)[31]:

"Het juridische instrument van ge- en verboden wordt toegepast bij zowel alcohol als roken. Roken. Na het invoeren van de rookvrije werkplek, het rookvrije personenvervoer, een forse accijnsverhoging, een grote massamediale campagne en daaraan voorafgaand de invoering van een leeftijdsgrens, reclame- en sponsoringsverbod, meer handhaving en gezondheidswaarschuwingen op de verpakking in de periode 2001-2004 is de totale consumptie van rookwaren met 9 % en het aantal rokers met 10% (400.000 personen) verlaagd. Ook is het aantal situaties waarbij niet-rokers worden blootgesteld aan meeroken flink gedaald. Rookverboden hebben daarmee bijna zeker een aanzienlijk effect gehad op de incidentie van zowel roken als meeroken. Er zijn geen studies uitgevoerd over de effectiviteit (gezondheidswinst) of de

31. Bijlage bij TK, 2007–2008, 22894, nr.134.

kosteneffectiviteit van deze verboden, zodat hierover niet met zekerheid conclusies zijn te trekken. Het is waarschijnlijk echter een kosteneffectief instrument: de kosten zijn relatief laag, terwijl de baten, in termen van minder rokers en minder overlast voor niet -rokers aanzienlijk zijn." (p.109)

In een algemeen overleg in de Tweede Kamer op 19 januari 2011 dient mevrouw Bouwmeester (PvdA) de volgende motie in[32]:

"De Kamer, gehoord de beraadslaging, …

- *overwegende dat het onderscheid in horeca waar wel en niet gerookt mag worden extra bureaucratie veroorzaakt, en toezicht en handhaving daardoor extra mensen vergen;*
- *constaterende dat sinds de aankondiging van versoepeling van het rookverbod in de horeca er momenteel in 51% van de kroegen wordt gerookt;*
- *constaterende dat de nVWA veel te weinig mensen heeft om de controle en handhaving uit te voeren, aangezien er in totaal 40 teams van twee mensen zijn die alle 17.000 kroegen in Nederland moeten controleren;*
- *constaterende dat de regering geen extra controleurs inzet en geen concreet voorstel heeft gedaan voor hogere boetes;– constaterende dat alleen een hoge pakkans in samenhang met hoge boetes afschrikwekkend zal werken;*
  verzoekt de regering, het wettelijk rookverbod te handhaven door voldoende toezicht en handhaving en hogere boetes te garanderen, en hiermee het aantal overtredingen zo veel mogelijk terug te brengen; …". (p.2)

Minister Schippers reageert ondermeer als volgt op de bovenstaande motie:

"In het verzoek aan de regering staat dat er voldoende toezicht moet zijn. Misschien vindt mevrouw Bouwmeester het niet voldoende, maar ik vind dat wel. Ook wordt er gevraagd om handhaving door hogere boetes te garanderen. Ik heb aangekondigd dat ik met fors hogere boetes kom. … Daar komt bij dat het altijd gemakkelijk is om te zeggen dat het aantal handhavers moet worden uitgebreid. Wij hebben echter ook een huishoudboekje op orde te brengen. Daarvoor moeten heel serieuze en moeilijke keuzes worden gemaakt. Ik maak die keuzes blijkbaar anders dan de PvdA-fractie dat doet." (p.5)

Uit de bovenstaande citaten blijkt dat de kosten van de handhaving van de preventiemaatregel primair bestaan uit de kosten van 80 inspecteurs (40 teams van twee inspecteurs) van de nieuwe Voedsel en Waren Autoriteit (nVWA). In hoeverre de kosten van deze inspecteurs aan de handhaving van het rookverbod in de horeca kunnen worden toegeschreven, wordt uit de door mij bestudeerde documenten niet duidelijk. Minister Schippers stelt niet dat het aanstellen van meer inspecteurs

32. TK, 2010-2011, vergaderingnr.44, itemnr.6 (dossiernr.32011).

niet kosteneffectief is. Zij stelt dat meer inspecteurs niet passen bij de (algemene) bezuinigingsdoelstellingen van het kabinet.

In oktober 2009 verschijnt het onderzoeksrapport 'Even uitblazen. Eén jaar rookvrije horeca' van Regioplan. Dit rapport is een review van de onderzoeksrapporten van andere organisaties (TNS NIPO, INTRAVAL, Synovate, Foodstep, RIVM, enz.) over de gevolgen van het rookverbod in de horeca. Het ministerie van VWS is opdrachtgever van deze reviewstudie. De onderzoeksvraag van deze reviewstudie luidt als volgt (Regioplan, 2009):

> *"Het onderzoek richt zich op het krijgen van inzicht in het effect van de invoering van de rookvrije horeca op diverse aspecten, bekeken over een periode van (ten minste) twaalf maanden vóór en twaalf maanden na de invoering van het rookverbod (periode 1-7-2007 tot 1-7-2009).*
>
> *Dit leidt tot de volgende onderzoeksvragen, op drie thema's:*
> 1. *Volksgezondheid …*
> 2. *Naleving …*
> 3. *Economische effecten …." (p.1-2)*

Opmerkelijk is dat het ministerie van VWS aan Regioplan niet heeft gevraagd om te rapporteren over de kosteneffectiviteit van het rookverbod in de horeca. In het reviewrapport van Regioplan staat dan ook niets over kosteneffectiviteit.

Omzetdaling van horecabedrijven (overige lasten)

Op 16 december 2008 informeert minister Klink de Tweede Kamer in een brief over de praktische implicaties van de invoering van de rookvrije horeca (verder te noemen: brief van minister Klink in december 2008 over de implicaties van invoering)[33]:

> *"Volgens het CBS is een veelheid aan factoren (mede) bepalend voor de omzet: prijzen, weersomstandigheden, economie, consumentenvertrouwen, etc. CBS geeft aan geen direct verband te zien met de rookvrije horeca: er is sinds het derde kwartaal 2007 sprake van een negatieve omzettrend voor de horeca. Deze lijn zet zich na de invoering van de rookvrije horeca op 1 juli jl. voort."* (p.6)
>
> *"Aantal faillissementen. Uit een overzicht van horecafaillissementen blijkt dat het aantal cafés en restaurants dat failliet ging sinds de invoering van de rookvrije horeca is afgenomen waarmee de dalende trend sinds 2007 zich voortzet. In totaal gingen 175 horecagelegenheden failliet. Middelgrote en grote zaken met twee of meer*

33. TK, 2008–2009, 22894, nr.206.

man personeel vormden het leeuwendeel van de faillissementen." [http://www.curatoren.nl/fo/nieuws.php?id=2754]

Op 1 december 2009 rapporteert minister Klink op basis van diverse wetenschappelijke onderzoeken in een brief aan de Tweede Kamer over de resultaten van één jaar rookvrije Horeca (verder te noemen: brief van minister Klink in december 2009 over één jaar rookvrije horeca). Hij schrijft ondermeer[34]:

> *"De invoering van de rookvrije horeca heeft voor zowel horecaondernemers als gemeenten een aantal knelpunten meegebracht. Horecaondernemers geven de omzetdaling en de onwil van klanten aan. De mate waarin de ondernemers knelpunten ervaren is in 2009 lager dan het jaar ervoor."* (p.2)

> *"De ontwikkelingen in de horecasector zijn niet uniek; uit analyse blijkt dat de horecasector geen afwijkende ontwikkelingen heeft doorgemaakt in vergelijking met andere sectoren of de gehele economie. De stijging van het aantal faillissementen in de horeca is ook vergelijkbaar met andere sectoren. Vanuit bedrijfseconomische aspecten, macro-economische ontwikkelingen en vergelijkingen met andere sectoren, lijkt het erop dat de recessie meer invloed heeft gehad op de omzet in de horeca, dan de invoering van het rookbod. Het consumentenvertrouwen blijkt sterk samen te hangen met de omzetontwikkelingen in de totale sector. Horecaondernemers zelf noemen ook in toenemende mate de economische crisis als oorzaak van de omzetdalingen."* (p.3)

Lasten van realiseren van rookvoorzieningen (overige lasten)

In de 'Memorie van antwoord' behorend bij het 'Wetsvoorstel tot wijziging van de Tabakswet' schrijft minister Borst-Eilers in december 2001[35]:

> *"De kosten die voor ondernemingen voortvloeien uit het realiseren van een rookvrije werkplek vallen in principe erg mee. Het rookbeleid moet binnen de onderneming goed worden gecommuniceerd. De praktijk leert verder dat nogal wat bedrijven, wanneer zij de rookvrije werkplek invoeren, medewerkers gaan stimuleren om te stoppen met roken (vergoeding van stoppen met roken-cursussen of andere hulpmiddelen). Tot de te maken kosten behoort verder eventueel het inrichten van een speciale rookruimte (een tegemoetkoming aan de verstokte rokers). Al deze kosten zijn in beginsel eenmalig."* (p.15)

34. TK, 2009–2010, 32011, nr.3.
35. EK, 2001–2002, 26472, nr.59a.

In oktober 2008 stelt Tweede Kamerlid Joldersma in een spoeddebat over de handhaving van het rookverbod in de horeca (verder te noemen: spoeddebat over de handhaving in oktober 2008) enkele praktische knelpunten aan de orde[36]:

> *"Een ander knelpunt waarover wij horen, is dat sommige cafés heel moeilijk aan een gemeentelijke vergunning kunnen komen voor een buitenterras. Ook hebben wij begrepen dat het voor een klein café heel moeilijk is om een rookruimte te realiseren, door de eisen die worden gesteld aan de omvang van het vloeroppervlak in de bouw- en de horecaregelgeving. Ik vraag de minister om aandacht hiervoor. Is er ruimte om ook een klein café dat zich aan de regels wil houden, toch een rookruimte te laten realiseren?"* (p.668)

Uit de brief van minister Klink in december 2008 over de implicaties van invoering[37]:

> *"Van de cafés zegt 72% een rookvoorziening te hebben. Een rookvoorziening kan zijn een rookruimte of rookkamer binnen, een terras (met of zonder terrasverwarming) of tuin. De omzetontwikkeling is niet afhankelijk van het wel of niet hebben van een rookvoorziening."* (p.9)

> *"De helft van de restaurants heeft een rookvoorziening, de andere helft niet. Net als bij de cafés zien we ook bij de restaurants geen significante verschillen in de omzetveranderingen tussen restaurants met of zonder rookvoorziening. Wel valt op dat juist de restaurants met een rookruimte significant vaker een omzetdaling melden dan gemiddeld."* (p.9)

> *"Bij 87 procent van de ondervraagde gemeenten is een vergunning nodig voor een inpandige verbouwing. Bij 32 procent van de gemeenten waarbij dit nodig is, is er sinds de invoering van het rookverbod in de horeca een aanvraag geweest. Het ging dan gemiddeld om 3,4 aanvragen per gemeente. In 76 procent van de gevallen werd de vergunning toegewezen. Bij de 24 procent die werd afgewezen, ging het in vrijwel alle gevallen (91 procent) omdat de aanvraag in strijd was met het gemeentelijke bestemmingsplan."* (p.13)

> *"Bij 88 procent van de ondervraagde gemeenten is een vergunning nodig voor een rookfaciliteit buiten. Het ging in 91 procent van de gevallen om een luifel of afdakje, bij 5 procent om gevelasbakken, bij 14 procent om statafels en 8 procent om een andere voorziening. Ondanks dat de meeste gemeenten voor een luifel of afdakje een vergunningsplicht hanteren, werd deze sinds 1 juli slechts bij 39 procent van de gemeenten aangevraagd. Het gemiddeld aantal aanvragen betrof 3,1 per gemeente. In 57 procent van de gevallen werd de vergunning toegewezen, in 39 procent van de gevallen afgewezen."* (p.13)

---

36. TK, 2008-2009, vergaderingnr.10, p.662-678 (dossiernr.22894).
37. Zie ook de reviewstudie van Regioplan Beleidsonderzoek uit oktober 2009.

"Conclusie vergunningen en overlast. In het onderzoek van TNS NIPO meldt 64 procent van de horecaondernemers dat zij een rookvoorziening hebben. Een rookvoorziening kan zijn een rookruimte of rookkamer binnen, een terras (met of zonder terrasverwarming) of een tuin. Daar waar horecaondernemers initiatieven nemen om binnen of buiten een rookvoorziening te creëren, is er geen reden om aan te nemen dat dit op grote knelpunten stuit." (p.14-15)

"Op basis van de gegevens uit het onderzoek van Intraval concludeer ik dat het merendeel van de vergunningaanvragen wordt toegekend. Een afwijzing geschiedt meestal omdat een aanvraag strijdig is met het plaatselijke bestemmingsplan. Niettemin zie ik hier wel de mogelijkheid om gemeenten te verzoeken om te bezien of er flexibiliteit getoond kan worden bij vergunningaanvragen." (p.15)

"Uit de onderzoeksgegevens en de informatie die hierboven is toegelicht, blijkt dat er op dit moment geen grond is om vast te stellen dat de rookvrije horeca voor bepaalde sectoren of (sub)groepen buiten proportioneel uitwerkt." (p.15)

Uit de brief van minister Klink in december 2009 over één jaar rookvrije horeca:

"Een aanzienlijk deel van de horecaondernemers heeft aanpassingen aan de horecagelegenheid gedaan om aan de bepalingen van de Tabakswet te voldoen: 72% beschikt over een rookvoorziening (binnen en/of buiten). Van deze horecagelegenheden heeft 38% een rookruimte binnen en 85% heeft buiten een voorziening (terras, tuin, asbakken of afdakjes). Slecht een klein deel van de ondernemers is van plan om komend jaar nog aanpassingen te doen in hun horecagelegenheid." (p.2)

Overlast door horecabezoekers die buiten staan (overige lasten)

Uit de brief van minister Klink in december 2008 over de implicaties van invoering:

"Gemeenten rapporteren over het algemeen (67 procent) geen knelpunten zoals overlastsituaties, vergunningverstrekking e.d. met betrekking tot de invoering van het rookverbod in de horeca. Bij de 29 gemeenten die wel knelpunten ervaren, gaat het vooral om (geluids)overlast van rokers buiten (27 procent) en om problemen met de voorzijden van horecapanden of het stads-/straataangezicht (25 procent).
Het ministerie van Binnenlandse Zaken en Koninkrijksrelaties heeft op mijn verzoek navraag gedaan bij de politiekorpsen. De reacties die eind week 50 binnen zijn, laten zien dat er in het algemeen enige toename van de overlast is. Dit beeld is in de verschillende gebieden/regio's wel zeer divers en varieert van geen of weinig toename tot enige of een duidelijke toename van de overlast." (p.14)

Uit de brief van minister Klink in december 2009 over één jaar rookvrije horeca:

"De invoering van de rookvrije horeca heeft voor … gemeenten een aantal knelpunten meegebracht. Gemeenten ervaren knelpunten ten aanzien van geluidsoverlast

van rokende horecaklanten en problemen met het straatgezicht. Ook rapporteren zij knelpunten rond terrasvergunningen voor de winter, rookruimtes in kleine horeca en de naleving. De mate waarin gemeenten knelpunten ondervinden is voor 2009 nagenoeg gelijk aan 2008." (p.2)

Substitutie van roken in horeca naar roken thuis (overige lasten)
Uit de brief van minister Klink in december 2009 over één jaar rookvrije horeca:

> *"Tot slot valt op dat er geen verplaatsing van het rookgedrag plaatsgevonden; rokers zijn thuis niet méér gaan roken."* (p.2)

Ontstaan van verzet tegen wet en agressie tegen handhavers (overige lasten)
In het spoeddebat over de handhaving in oktober 2008 stelt Tweede Kamerlid Agema:

> *"… Sinds een week wordt er namelijk direct een prent uitgedeeld aan horecaondernemers die zich niet houden aan het rookverbod. Ik heb daarom een aantal vragen aan de minister. In de eerste plaats vraag ik of hij zijn belofte aan de horecaondernemers nakomt. … De minister heeft de horecaondernemers beloofd dat zij altijd eerst een waarschuwing zouden krijgen voordat er een boete wordt uitgedeeld. In de tweede plaats vraag ik of er een relatie is met het geweld tegen de controleurs van de Voedsel en Waren Autoriteit, die sinds een week direct een prent uitdelen. … In de derde plaats vraag ik waar de tijd is gebleven waarin wij zeiden: wij komen er samen wel uit. Waar is de tijd gebleven waarin op vrijwillige basis eigenaars van veel horecagelegenheden besloten het roken niet toe te staan of in ieder geval een gedeelte van de zaak rookvrij te maken? Moet de minister niet op z'n minst kleine horecabedrijven de gelegenheid bieden om alsnog roken toe te staan? … In de vierde plaats vraag ik de minister waarom hij dit pad is ingeslagen. Wij zijn uitgegaan van een situatie waarin gold: wij komen er samen wel uit. Wij zijn nu echter beland in een situatie waarin de kloof tussen burger en politiek zo groot is geworden, dat wij kunnen spreken van een Grand Canyon." (p.662)*

In het spoeddebat over de handhaving in oktober 2008 stelt Tweede Kamerlid Van Gent:

> *"Een klein groepje voert nu een soort anarchistische actie met het plaatsen van asbakken op de bar. Ook in die cafés zal gewoon gehandhaafd moeten worden. … Als er massaal en opzichtig in een café wordt gerookt, vind ik het uitermate terecht, mijnheer Zijlstra, dat je de wet handhaaft en een boete uitdeelt. U zou het met mij erg moeten vinden dat mensen van de Voedsel en Waren Autoriteit hun werk niet kunnen doen, omdat ze bedreigd worden en lastig worden gevallen. Ik begreep vanochtend dat dat het geval is en daar zou u zich ook eens zorgen om moeten maken."* (p.667)

> *"…95% van de ondernemers handhaaft het rookverbod gewoon. Slechts 5% doet dat niet. 95% moet bloeden voor de 5% die het verbod niet handhaaft, dat is pas oneerlijke concurrentie! ... Ik vind dat wij het rookverbod in de horeca gewoon moeten handhaven. ... Ook voor degenen die wel handhaven is het goed dat de overtreders worden aangepakt. Vanuit mijn eigen stad Groningen schijnt de anarchie aangevoerd te worden als het gaat om roken in cafés. Daar moet mee opgehouden worden. Wij moeten de zaak niet overdrijven. Sinds het rookverbod zijn er ruim 6700 bedrijven bezocht. Er zijn 22 boetes uitgedeeld voor 1 oktober en nog eens 22 sindsdien. Wij moeten dus niet net doen alsof het allemaal heel erg is." (p.666)*

Afname van de keuzevrijheid van bezoekers (overige lasten)
In het spoeddebat over de handhaving in oktober 2008 stelt Tweede Kamerlid Zijlstra:

> *"… Mevrouw Bouwmeester zegt steeds: gezondheid, gezondheid, gezondheid. Ik begrijp daaruit dat de PvdA-fractie vindt dat de politiek gaat bepalen wat een burger gezondheidstechnisch moet doen. Volgens de PvdA-fractie mag de burger niet langer ervoor kiezen om naar een café te gaan, waar hij al dan niet gaat roken, omdat gezondheid zo belangrijk is dat die keuzevrijheid volledig weggenomen moet worden. Dat is echt goed ouderwets socialistisch denken, maar volgens mij niet langer goed voor deze maatschappij."* (p.666)

Vermindering van risico op gezondheidsschade bij bezoekers (overige baten)
Het reviewrapport van Regioplan uit oktober 2009 over de resultaten van één jaar rookvrije horeca stelt:

> *"De luchtkwaliteit is na de invoering van het rookverbod aanmerkelijk verbeterd. De concentratie fijn stof (PM2.5) is negen keer lager. De blootstelling aan tabaksrook is drastisch gedaald onder niet-rokers. … Op basis van onderzoeken in binnen- en buitenland kunnen we voorzichtig concluderen dat een rookverbod in de horeca de levensverwachting verlengt en het aantal hart- en vaatziekten vermindert."* (p.3)

Vergroting van keuzevrijheid van bezoekers (overige baten)
Na invoering van het rookverbod per 1 juli 2008 wordt hier in het parlementaire debat eigenlijk niet of nauwelijks meer over gesproken, terwijl dit toch een belangrijk positief neveneffect zou kunnen zijn.

Vermindering van hinder en overlast voor bezoekers (overige baten)
Na invoering van het rookverbod per 1 juli 2008 wordt hier in het parlementaire debat eigenlijk niet of nauwelijks meer over gesproken, terwijl dit toch een belangrijk positief neveneffect zou kunnen zijn.

Bezoekers en werknemers zijn minder gaan roken of gestopt met roken (overige baten)

Het reviewrapport van Regioplan uit oktober 2009 over de resultaten van één jaar rookvrije horeca stelt:

> *"Door de invoering van het rookverbod in de horeca is één op de tien rokers minder gaan roken. Rokers hebben door het rookverbod in de horeca vaker een stoppoging ondernomen en zijn daarin vaker succesvol geweest."* (p.3)

Omzetstijging van horecabedrijven (overige baten)

In juli 2007 stelt Tweede Kamerlid Bouwmeester in een algemeen overleg met minister Klink over zijn besluit tot invoering van een rookverbod in de horeca[38]:

> *"Mevrouw Bouwmeester (PvdA): De horeca is bang voor neveneffecten: overlast, lawaai, stank, sigarettenpeuken op straat en onveiligheid. Uit buitenlandse ervaringen blijkt echter dat handhaving goed mogelijk, dat er draagvlak is en dat de mensen over het algemeen positief zijn. De omzet blijkt zelfs toe te nemen. Hoe zorgt de minister ervoor dat dit ook in Nederland zal gebeuren?"* (p.1)

In het spoeddebat over de handhaving in oktober 2008 stelt Tweede Kamerlid Zijlstra:

> *"Ik haal de minister aan. Net als de antirooklobby heeft hij de wet altijd verdedigd met de mogelijkheden die zouden ontstaan door een rookvrije horeca. De klanten zouden toestromen. Ik ben daarover eerlijk gezegd een beetje cynisch. Als dit zo zou zijn, zullen grote kroegen deze mogelijkheid willen benutten. Kleine kroegen zullen soms de mogelijkheid benutten om rookvrij te zijn. Zij hebben echter de keuze. De VVD wil deze keuzevrijheid."* (p.664)

Ontstaan van een sociaal klimaat waarin niet-roken de norm is (overige baten)

In 1996 schrijft minister Borst-Eilers aan de Tweede Kamer onder de tussenkop *"Rookverboden. Openbare ruimten."*[39]:

> *"Het Besluit beperking verkoop en gebruik tabaksprodukten (1990) levert een bijdrage aan de hoofddoelstellingen van het tabaksontmoedigingsbeleid, namelijk de beperking van de consumptie van tabaksprodukten en de bescherming van de jeugd en niet-roker. Daarnaast worden rokers nadrukkelijk geconfronteerd met een maatregel die ten opzichte van het roken een negatief oordeel inhoudt. Hierdoor kan de motivatie toenemen om geheel te stoppen met roken of de consumptie ook in andere*

38. TK, 2006–2007, 30800 XVI, nr.182.
39. TK, 1995–1996, 24743, nr.1.

omstandigheden dan tijdens het verblijf in een openbare ruimte verminderen. Omdat een aantal inrichtingen waar rookverboden zijn ingesteld veelvuldig wordt bezocht door jongeren, kan een sociaal klimaat ontstaan, waarin niet-roken de norm is en het niet beginnen of stoppen met roken het gevolg is." (p.6)

In maart 2002 stelt minister Borst-Eilers in een overleg met de Eerste Kamer over haar Wetsvoorstel tot wijziging van de Tabakswet[40]:

"Een tweede voorbeeld is dat als je probeert rokers tot stoppen aan te zetten, dit meer effect zal hebben – dat hoor je ook van de betreffende mensen – als zij merken dat zij op steeds meer plekken niet meer mogen roken. Hoewel wettelijk vastgelegde rookverboden primair voortkomen uit het motief dat wij de niet-roker moeten beschermen, hebben zij het bijkomend voordeel dat zij méér rokers overhalen om nu toch maar te stoppen." (p.1258).

Uit de brief van minister Klink in december 2009 over één jaar rookvrije horeca:

"Daarnaast heeft de invoering van de rookvrije horeca positieve effecten gehad op het rookgedrag: rokers zijn vaker gestimuleerd een stoppoging te ondernemen en deze stoppogingen zijn ook vaker succesvol geweest." (p.2)

Analyse van de lasten en baten van het rookverbod
In het politieke debat speelt de kosteneffectiviteit (de verhouding tussen de uitvoeringskosten en effectiviteit) van het rookverbod in de horeca geen enkele rol. De (structurele) kosten van de uitvoering van de preventiemaatregel lijken grotendeels te bestaan uit de kosten van de handhaving door de nVWA. Deze kosten worden in de door mij bestudeerde parlementaire documenten niet geëxpliciteerd. Er zijn geen aanwijzingen dat het rookverbod in de horeca niet kosteneffectief is. In 2002 heeft minister Borst-Eilers gesteld: "*Wetgeving is en blijft ook een kosteneffectief instrument binnen het totale samenhangende tabaksontmoedigingsbeleid van de regering."*. Ik vermoed dat de regering en Kamerleden hier impliciet vanuit gaan, ook wat betreft het rookverbod in de horeca.

Het politieke debat over de neveneffecten van het rookverbod in de horeca wordt sterk gedomineerd door de (vermeende) economische effecten. Op basis van het reviewonderzoek van Regioplan uit oktober 2009 kan worden geconcludeerd dat het onwaarschijnlijk is dat het rookverbod in de horeca voor de horecasector als geheel tot grote omzetdalingen heeft geleid. M.i. kan op basis van de beschikbare onderzoeksgegevens echter niet worden uitgesloten dat bepaalde (sub)sectoren van de horeca ten gevolge van het rookverbod wel door grote omzetdalingen

40. EK, 2001-2002, vergaderingnr.24, p.1236-1275 (dossiernr.26472).

getroffen zijn. De ongelijke verdeling van lasten over partijen valt echter niet onder het criterium 'lasten-batenverhouding' (9e criterium), maar onder het criterium 'rechtvaardigheid' (10e criterium) van het model.

Bij de beoordeling van de verhouding tussen baten en lasten dienen alle baten te worden vergeleken met alle lasten. Minister Klink heeft wel onderzoek laten uitvoeren naar alle lasten van het rookverbod in de horeca, maar heeft m.i. verzuimd om alle baten te laten onderzoeken. Belangrijke baten die niet zijn onderzocht zijn:

- vergroting van keuzevrijheid van bezoekers (althans van degenen die niet roken);
- vermindering van hinder en overlast voor bezoekers.

In het politieke debat over baten en lasten van het rookverbod in de horeca worden de twee bovengenoemde positieve neveneffecten m.i. onterecht stelselmatig genegeerd.

12.3.3 Eindoordeel (criterium lasten-batenverhouding)

Naar de lasten en baten van het rookverbod in de horeca is veel onderzoek gedaan en daardoor is hierover veel informatie beschikbaar. Deze informatie bevat geen aanwijzingen dat er sprake is van een negatieve lasten-batenverhouding voor de horecasector als geheel.

De informatie die beschikbaar is over de lasten-batenverhouding van het rookverbod in de horeca is echter toch onvolledig:

- de uitvoeringskosten c.q. kosteneffectiviteit van het rookverbod in de horeca zijn niet publiekelijk kenbaar gemaakt (ik tref ze niet aan in de parlementaire documenten);
- belangrijke positieve neveneffecten zijn niet onderzocht.

Ik acht het onwaarschijnlijk dat volledige informatie over de lasten-batenverhouding van het rookverbod in de horeca zal leiden tot een ongunstiger beeld van de lasten-batenverhouding, dan het huidige beeld dat is gebaseerd op onvolledige informatie.

Op basis van de bovenstaande overwegingen kom ik tot de conclusie dat beoordeling van het rookverbod in de horeca aan de hand van het criterium 'lasten-batenverhouding' van het model geen aanleiding vormt om dit rookverbod ongerechtvaardigd te verklaren. De informatieverzameling over de lasten-batenverhouding van het rookverbod in de horeca acht ik niet zo gebrekkig, dat de onvolledigheid

van deze informatieverzameling aanleiding vormt om toepassing van de preventiemaatregel ongerechtvaardigd te verklaren.

12.3.4 Gevolgen van de beoordeling voor het model (criterium lasten-batenverhouding)

Op basis van alle door mij bestudeerde literatuur ben ik tot de volgende conclusie gekomen. Bij de beoordeling van de gerechtvaardigdheid van leefstijlbeïnvloedende interventies ter bestrijding van leefstijlgerelateerde aandoeningen, speelt de indringendheid van de preventiemaatregel veelal een grotere rol dan de kosteneffectiviteit. Naar aanleiding hiervan heb ik ervoor gekozen om van het aspect ‘indringendheid’ een afzonderlijk criterium te maken en om het aspect ‘kosteneffectiviteit’ onder te brengen onder het criterium ‘lasten-batenverhouding’. De beoordeling van het wettelijk rookverbod in de horeca aan de hand van het criterium ‘lasten-batenverhouding’ bevestigt het ondergeschikte belang van de kosteneffectiviteit bij de beoordeling van de gerechtvaardigdheid van leefstijlbeïnvloedende interventies ter bestrijding van leefstijlgerelateerde aandoeningen.

12.4 Tiende criterium: rechtvaardigheid

12.4.1 Operationalisering en wijze van beoordeling

Het criterium ‘rechtvaardigheid’ betreft de
- distributieve rechtvaardigheid,
- vergelijkende rechtvaardigheid, en
- legitimiteit,

van de preventiemaatregel.

Distributieve rechtvaardigheid
De distributieve rechtvaardigheid betreft de vraag of de baten en lasten van de preventiemaatregel rechtvaardig over partijen worden verdeeld.

Vergelijkende rechtvaardigheid
De vergelijkende rechtvaardigheid betreft de vraag of partijen in vergelijkbare situaties vergelijkbaar worden behandeld. Hierbij kan ook een vergelijking worden gemaakt tussen partijen die wel en niet bij de preventiemaatregel zijn betrokken.

Legitimiteit
De legitimiteit van de preventiemaatregel betreft de vraag of de preventiemaatregel niet strijdig is met het vigerende recht (wet- en regelgeving, nationale en internationale verdragen, rechtsgeldige overeenkomsten, e.d.). Het vigerende recht vormt in een democratie immers een afspiegeling van (vroegere) opvattingen over rechtvaardigheid in de samenleving. Als een beoogde preventiemaatregel strijdig is met het vigerende recht, dient de preventiemaatregel niet te worden uitgevoerd, of dient het recht (wet- en regelgeving, nationale en internationale verdragen, rechtsgeldige overeenkomsten, e.d.) te worden aangepast. Ter illustratie van dit laatste onderstreepte zinsdeel volgt een citaat van Tweede Kamerlid Rouvoet uit een overleg in september 1996 van de vaste commissies voor VWS en EZ met minister Borst-Eilers over haar beleidsvoornemens op het gebied van het tabaksontmoedigingsbeleid[41]:

> *"Als argument om af te zien van het instellen van een rookverbod op internationale passagiersvluchten wordt genoemd dat de Tabakswet hiervoor geen grondslag biedt. Dat kan geen bezwaar zijn, want deze wet kan gewijzigd worden."* (p.10)

Ik heb gekozen voor de toevoeging van het aspect van 'legitimiteit' aan het 10e criterium 'rechtvaardigheid' van het model, omdat de legitimiteit van het wettelijk rookverbod in de horeca diverse malen ter discussie is gesteld. Twee gerechtshoven zijn tot de conclusie gekomen dat de Tabakswet onvoldoende rechtsgrond bood voor een rookverbod in horecabedrijven zonder personeel[42]. Dit is van grote invloed geweest op het draagvlak voor en de handhaafbaarheid van het rookverbod (draagvlak en handhaafbaarheid zijn criteria uit de 3e zeef van het model). Ter illustratie een deel van een nieuwsbericht van de website van het ministerie van VWS d.d. 23 februari 2010:

> *"De Hoge Raad heeft vandaag uitspraak gedaan over het rookverbod in de horeca. Minister Klink van Volksgezondheid, Welzijn en Sport (VWS) reageert verheugd op de uitspraak. De Hoge Raad komt tot de conclusie dat het rookverbod in de horeca, geldend sinds 1 juli 2008, ook geldt voor horecagelegenheden zonder personeel.*
>
> *Afgelopen zomer [2009] werd de handhaving van het rookverbod bij horeca zonder personeel tijdelijk opgeschort naar aanleiding van uitspraken van de gerechtshoven in Den Bosch en Leeuwarden. Het OM (Openbaar Ministerie) ging in cassatie. Met de uitspraak is een einde gekomen aan een periode van onduidelijkheid over de reikwijdte van de wet. Het rookverbod geldt voor de gehele horeca."*

Het criterium rechtvaardigheid kent de volgende aspecten:

---

41. TK, 1996–1997, 24743, nr3.
42. TK, 2008-2009, 32011, nr.1.

1. Aspect van verantwoordelijkheid:
2. In hoeverre worden de lasten van de preventiemaatregel over partijen verdeeld naar rato van hun verantwoordelijkheid voor het risico op schade, hinder en overlast?
3. Aspect van gelijke behandeling
   Worden partijen in gelijke gevallen gelijk behandeld (zonder rekening te houden met draagkracht)?
4. Aspect van draagkracht
   In hoeverre houdt de preventiemaatregel rekening met de draagkracht van mensen (dragen de sterkste schouders de zwaarste lasten)?
5. Aspect van hardheid in specifieke gevallen
   In hoeverre houdt de preventiemaatregel rekening met de persoonlijke omstandigheden van mensen of specifieke omstandigheden van organisaties?
6. Aspect van gelijke toegang tot gezondheidszorg en preventie
   Welke invloed heeft de preventiemaatregel op de gelijke kansen van betrokken partijen wat betreft de toegang tot gezondheidszorg en preventie?
7. Aspect van verdeling naar behoefte van gezondheidszorg en preventie
   Welke invloed heeft de preventiemaatregel op de verdeling van de voor de gezondheidszorg en preventie beschikbare middelen naar behoefte van de betrokken partijen?
8. Aspect van legitimiteit
   Is de preventiemaatregel niet strijdig met het vigerende recht (wet- en regelgeving, nationale en internationale verdragen, rechtsgeldige overeenkomsten, e.d.)?

Beoordeeld wordt of toepassing van de preventiemaatregel vanuit het perspectief van de distributieve en vergelijkende rechtvaardigheid niet onredelijk of onbillijk is (1e t/m 6e aspect) en niet strijdig is met het vigerende recht (7e aspect).

12.4.2 *Beoordeling van casus 'wettelijk rookverbod in horeca' aan de hand van criterium 'rechtvaardigheid'*

Aspect van verantwoordelijkheid
In hoeverre worden de lasten van de preventiemaatregel over partijen verdeeld naar rato van hun verantwoordelijkheid voor het risico op schade, hinder of overlast?

De lasten van de preventiemaatregel bestaan uit (zie overzicht 21 in §12.3.2)[43]!!:
– indringendheid van de preventiemaatregel;
– uitvoeringskosten;

– omzetdaling van horecabedrijven;
– lasten van realiseren van rookvoorzieningen;
– overlast door horecabezoekers die buiten staan;
– ontstaan van verzet tegen wetgeving en agressie tegen wetshandhavers;
– afname van de keuzevrijheid van bezoekers.

Rokers worden zelf verantwoordelijk gehouden voor hun rookgedrag (zie het criterium 'verantwoordelijkheid' in §11.4). De verantwoordelijkheid voor de handhaving van het rookverbod wordt echter toegewezen aan de eigenaar van de horecagelegenheid, die vaak ook werkgever is. Als rokers het rookverbod overtreden worden zij niet beboet, maar kan de eigenaar van de horecagelegenheid worden beboet, wegens het niet handhaven van het wettelijk rookverbod. Over deze keuze heeft de politiek uitgebreid gedebatteerd voorafgaand aan de invoering van het wettelijk recht op een rookvrije werkplek, dat op 1 januari 2004 van kracht is geworden. In het kader van het wettelijk rookverbod in de horeca wordt deze keuze echter niet of nauwelijks opnieuw ter discussie gesteld. De keuze om de eigenaar van de horecagelegenheid in plaats van de roker te beboeten, wordt niet onrechtvaardig gevonden. De argumenten voor deze keuze licht ik daarom niet toe onder het criterium 'rechtvaardigheid', maar onder het criterium 'handhaafbaarheid' (§13.4).

Het politieke debat over de rechtvaardigheid van de preventiemaatregel spitst zich toe op de rechtvaardigheid c.q. aanvaardbaarheid van de vermeende grote omzetdalingen in kleine horecabedrijven zonder personeel. Blijkbaar wordt de toewijzing en verdeling van de overige lasten (anders dan omzetdalingen) aan partijen aanvaardbaar geacht.

Aspect van gelijke behandeling
In de Tweede Kamer is frequent en uitgebreid gedebatteerd over de gelijke behandeling van horecaondernemers in het kader van het wettelijk rookverbod in de horeca. Het debat spitst zich toe op de vraag of er sprake is van concurrentievervalsing, omdat kleine kroegen niet over de ruimte beschikken om een rookruimte in te richten.

In oktober 2008 stelt Tweede Kamerlid Zijlstra in een spoeddebat over de handhaving van het rookverbod[44]:

43. Van substitutie van roken in de horeca naar roken thuis blijkt geen sprake te zijn.
44. TK, 2008-2009, vergaderingnr.10, p.662-678 (dossiernr.22894).

"… wij zijn … tegen het rookverbod in de huidige vorm. Wij vinden dat te rigide en wij zien de resultaten daarvan. Er komen steeds meer signalen dat er forse omzetdalingen bij de horeca zijn. Dat speelt vooral bij kleine cafés. … Feitelijk is er sprake van concurrentievervalsing. … Een kleine kroeg heeft niet de mogelijkheid om een rookruimte in te richten, simpelweg omdat daarvoor de vierkante meters ontbreken. … Wij streven naar een aanpassing van de Tabakswet, maar dat moeten wij doen op het moment dat de omzetcijfers beschikbaar zijn." (p.663)

In november 2008 debatteren de vaste commissie van de Tweede Kamer voor VWS en minister Klink over de (vermeende) concurrentievervalsing tussen grote en kleine cafés[45]:

"De heer Zijlstra (VVD): Voorzitter. Het rookverbod zet de horeca in vuur en vlam. Het probleem ligt met name bij de kleine cafés. Er is sprake van oneerlijke concurrentie. Grote horeca kan een rookruimte maken. Kleine horeca kan dat fysiek niet. Het grappige is dat horeca zonder personeel destijds onder de wet moest vallen om te voorkomen dat er onjuiste concurrentie zou optreden. In de praktijk treedt die dus wel degelijk op. Het zijn in de praktijk de kleine kroegen die de pisang zijn. In de NRC van 22 november schrijft een oud-lid van de Raad van State, die verstand van zaken heeft, dat de kleine horeca uitgezonderd moet worden. … Wij moeten een keuze maken: of een algeheel rookverbod of er is een uitzondering mogelijk. De VVD kiest voor het laatste en kiest voor een uitzondering voor horeca zonder personeel." (p.1)

[Minister Klink] "Op voorhand heb ik geen enkele aanleiding om tot compensatie over te gaan in financiële zin, zoals de heer Van Gerven bedoelde, of te definiëren vanaf welke oppervlakte cafés gevrijwaard zijn van het rookverbod. Dat zou namelijk betekenen – een van de punten in het overleg met de Koninklijke Horeca Nederland in de aanloop naar het verbod – dat een aantal middelgrote bedrijven en bedrijven die net boven het aantal vierkante meters uitkomen een concurrentienadeel ondervinden. Het was met het oog op het «level playing field» dat wij hebben gekozen voor een generiek rookverbod. Inderdaad, degenen zonder personeel zouden strikt genomen niet verplicht dienen te worden om een rookvrije werkplek te realiseren. Gegeven concurrentieoverwegingen hebben wij een integrale keuze gemaakt. Daar liep de Koninklijke Horeca Nederland niet voor weg. Sterker nog, zij bepleitte dat ook, zij het met de kanttekening dat men een periode van overgang voor de kleine horeca wenste. Uiteindelijk zou over de gehele linie hetzelfde verbod geïmplementeerd dienen te worden." (p.13)

"Minister Klink: Nee, de maatregelen staan op papier. Daar vloeien feiten uit voort die de heer Zijlstra en ik niet kennen. Ik constateer dat 16% van de kroegen een rookruimte heeft gemaakt. De heer Zijlstra constateert nu al dat er een nadeel bestaat

45. TK, 2008–2009, 22894, nr.207.

voor kroegen die geen rookruimte kunnen inrichten. Dat zegt hij op basis van vooronderstellingen en niet op basis van de feiten. Ik ga de feiten bekijken. De Kamer krijgt in, … , nog voor die met reces gaat een knelpuntenanalyse. Ik hoop en verwacht dat wij daar dan al enig inzicht kunnen bieden. Ik loop daar echter niet op vooruit. Zo-even is gesproken over wijs besturen. Wijs besturen is je baseren op feiten en niet op vermoedens." (p.14)

In maart 2009 stelt Tweede Kamerlid Agema tijdens een overleg van de vaste Tweede Kamercommissie voor VWS met minister Klink impliciet dat het concurrentievoordeel van de aanwezigheid van een rookruimte beperkt is[46]:

"Bij de cafés waar niet wordt gerookt, is heel duidelijk sprake van een enorme omzetdaling. Dat geldt ook voor de cafés met een rookruimte, want dat is gewoon veel minder gezellig." (p.2)

In ditzelfde overleg (maart 2009) beargumenteert Tweede Kamerlid van Gerven dat het wel meevalt met de concurrentievervalsing, als de kleine cafés zonder personeel worden vrijgesteld van het rookverbod:

"Het mogelijke concurrentienadeel is begrensd, want een kroeg zonder werknemers kan maar heel beperkt groeien. Als er heel veel gasten komen, zullen ze personeel in dienst moeten nemen en dan moeten zij direct rookvrij worden, dus ik denk dat het wel mee zal vallen met die concurrentie." (p.9)

Het reviewrapport van Regioplan uit oktober 2009 over de resultaten van één jaar rookvrije horeca stelt:

"Bijna driekwart (72%) van de horecagelegenheden heeft een rookvoorziening. Hiervan heeft een derde van de horecagelegenheden binnen een rookruimte (evenveel als vorig jaar), 85% heeft buiten een voorziening om te roken." (p.11)

"Ongeveer de helft van de horecaondernemers heeft te maken met omzetdalingen … Omzetdalingen zijn in de gehele sector zichtbaar zowel bij grote als bij kleine ondernemingen. Het aantal werknemers in een horecaonderneming heeft geen significant verband met gerapporteerde omzetdalingen. Horecagelegenheden zonder rookvoorziening ervaren vergelijkbare omzetontwikkelingen als horecagelegenheden met rookvoorziening." (p.23)

"Een derde van de gemeenten meldt knelpunten vanwege de rookvrije horeca. In 2008 ondervonden gemeenten met name last van buiten rokende horecaklanten en problemen met het straatgezicht. In 2009 kwamen daar terrasvergunningen voor de winter, rookruimtes in de kleine horeca en naleving bij." (p.41)

46. TK, 2008–2009, 22894, nr.216.

In het najaar van 2008 werd duidelijk dat vooral horecaondernemers van kleine cafés (zonder personeel) meenden grote omzetdalingen ten gevolge van het rookverbod in de horeca te ondervinden. Daarom vind ik het verbazingwekkend dat de onderzoeken die in 2009 zijn uitgevoerd naar de gevolgen van de invoering van de rookvrije horeca (zie voor een overzicht het reviewrapport van Regioplan uit oktober 2009) voornamelijk zijn gericht op de horecasector als geheel of op horecasectoren als geheel (cafés, restaurants, discotheken, enz.) en dat niet of nauwelijks is ingezoomd op de vermeende grote (omzet)dalingen van kleine cafés (zonder personeel).

Het overgrote deel van de rookvoorzieningen in de horeca bestaat niet uit een rookruimte binnen, maar uit een rookvoorziening buiten (terras met eventueel verwarming en/of luifel). Ik acht het denkbaar dat het voor kleine cafés (zonder bestaand terras) veel moelijker is om een aantrekkelijke rookvoorziening buiten te realiseren dan voor grote cafés (met bestaand terras). Ook is het mogelijk dat de klantenkring van kleine cafés (mogelijk een lagere SES en daardoor een andere attitude t.a.v. roken en gezondheid; mogelijk ouder; mogelijk ongezonder) het bezwaarlijker vindt om buiten te roken dan de klantenkring van grote cafés (mogelijk een hogere SES; mogelijk jonger; mogelijk gezonder). Al deze aspecten zijn naar mijn informatie niet onderzocht.

Of er al dan niet sprake is van concurrentievervalsing kan het best worden geobjectiveerd op basis van kwantitatieve informatie over wijzigingen in de omzet van kleine cafés en grote cafés na invoering van het rookverbod. Op basis van het reviewonderzoek van Regioplan uit oktober 2009 kan worden geconcludeerd dat het onwaarschijnlijk is dat het rookverbod in de horeca voor de horecasector als geheel tot grote omzetdalingen heeft geleid (zie voor een toelichting de *'Analyse van de lasten en baten van het rookverbod'* in §12.3.2). Op basis van de beschikbare onderzoeksgegevens kan echter niet worden uitgesloten dat kleine cafés zonder personeel ten gevolge van het rookverbod wel door grote omzetdalingen getroffen zijn. In deze paragraaf (12.4.2) ga ik daar onder het kopje *'Aspect van hardheid in specifieke gevallen'* nader op in.

Ten slotte speelt wat betreft het onderwerp 'gelijke behandeling' ook nog de juridische kwestie van de mogelijkheid tot het installeren van luchtzuiveringsinstallaties als alternatief voor het rookverbod. Formeel juridisch bestaat deze mogelijkheid wel voor cafés met personeel die moeten voorkomen dat werknemers hinder of overlast van tabaksrook ondervinden, maar niet voor cafés zonder personeel waarvoor een rookverbod geldt. Vooralsnog is dit een formeel juridische kwestie, omdat luchtzuiveringsinstallaties als alternatief voor het rookverbod in de praktijk

nog niet zijn toegestaan. Daarom bespreek ik deze kwestie in deze paragraaf (12.4.2) onder het kopje *'Aspect van legitimiteit'*.

Aspect van draagkracht

In november 2008 debatteren de vaste commissie van de Tweede Kamer voor VWS en minister Klink over de (vermeende) concurrentievervalsing tussen grote en kleine cafés[47]:

> *"De heer Zijlstra (VVD): … Er is sprake van oneerlijke concurrentie. Grote horeca kan een rookruimte maken. Kleine horeca kan dat fysiek niet. … Wij moeten een keuze maken: of een algeheel rookverbod of er is een uitzondering mogelijk. De VVD kiest voor het laatste en kiest voor een uitzondering voor horeca zonder personeel."* (p.1)

> *"De heer Van der Ham (D66): In de kleine horeca bestaat een grote diversiteit. Dat levert direct problemen op als wij doen wat de heer Zijlstra wil. Stel je voor dat alle kleine horecagelegenheden en cafés, die onder de 70 vierkante meter, een rookcafé mogen zijn. Dan heeft de daarnaast gelegen caféhouder met een café van 80 vierkante meter en dus net wel ruimte voor een rokersplek enorm grote problemen, een enorm concurrentienadeel ten opzichte van dat net wat kleinere café. Het concurrentienadeel dat de heer Zijlstra voor de kleinere cafés ziet klopt voor een deel. Een oplossing zoals hij het voorstelt, betekent direct een enorm groot nadeel voor de middelgrote caféhouder. Daar houdt hij geen rekening mee."* (p.8)

> *"De heer Zijlstra (VVD): De heer Van der Ham zegt dat er bij mijn oplossing een ander concurrentienadeel optreedt. Daarom is de grens wanneer er een fatsoenlijke rokersruimte aangelegd kan worden belangrijk. Er moet fysiek de mogelijkheid toe bestaan. De heer Van der Ham wil dat de groep met het minste spek op de botten, de kleine horecaondernemer, de nadelen draagt. Wij vinden dat het nadeel beter bij de grote kroegen dan bij de kleine neergelegd kan worden."* (p.8)

In maart 2009 stelt Tweede Kamerlid Agema tijdens een overleg van de vaste Tweede Kamercommissie voor VWS met minister Klink[48]:

> *"Minister Klink schrijft dat een vermindering van de omzet met 4% hem bescheiden voorkomt, maar dat begrijp ik niet goed. Meestal gaat het zo in een horecabedrijf: de omzet van de eerste week ben je kwijt aan pacht en personeel, van de tweede week aan de toeleveranciers, van de derde week aan verzekeringen, gas, water en elektriciteit en al die andere rekeningen. Als er aan het einde van de rit nog wat overblijft, dan is dat je inkomen. Als dat maar 15 of 20% is en er gaat 4% vanaf, dan valt een heel groot deel van het inkomen weg. Daardoor kunnen horecaondernemers in de*

47. TK, 2008–2009, 22894, nr.207.
48. TK, 2008–2009, 22894, nr.216.

problemen komen, en dan vooral door het rookverbod. 70% van de cafés tot 100 m2 heeft een omzetdaling. De minister weet niet hoeveel ondernemingen extra leningen hebben aangevraagd als gevolg hiervan. Hij stelt in de brief dat er geen relatie is met het rookverbod. Het zijn echter vertroebelde cijfers, die ontvlecht zouden moeten worden. Ik heb hier gegevens over 64 cafés, onderverdeeld in roken, rookruimte en niet roken. Bij de cafés waar niet wordt gerookt, is heel duidelijk sprake van een enorme omzetdaling. Dat geldt ook voor de cafés met een rookruimte, want dat is gewoon veel minder gezellig." (p.2)

In de bovenstaande citaten vragen in november 2008 de heer Zijlstra (VVD) en in maart 2009 mevrouw Agema (PVV) aandacht voor de beperkte financiële draagkracht van kleine cafés (ten opzichte van grote cafés). In de onderzoeken die in opdracht van het ministerie van VWS zijn uitgevoerd naar de gevolgen van de invoering van de rookvrije horeca (zie voor een overzicht het reviewrapport van Regioplan uit oktober 2009) wordt aan mogelijke verschillen in financiële draagkracht tussen grotere en kleinere cafés echter geen aandacht besteed.

Aspect van hardheid in specifieke gevallen

Het debat in de Tweede Kamer over de economische gevolgen van het rookverbod in de horeca spitst zich in 2008 en 2009 toe op de vraag of kleine cafés zonder personeel financieel gezien al dan niet onevenredig hard door het rookverbod worden geraakt.

In oktober 2008 stelt Tweede Kamerlid Zijlstra in een spoeddebat over de handhaving van het rookverbod[49]:

"Het CDA is er toch niet voor dat de handhaving van een wet erin uitmondt dat honderden en misschien wel 1000 ondernemers in dit land het loodje leggen? Dat kan toch nooit de bedoeling zijn? … De ondernemers die op dit moment van doen hebben met het rookverbod en hieraan een draai proberen te geven, doen dit uit lijfsbehoud. Daarom zegt de VVD: op dit moment moeten wij eerst wachten tot wij de cijfers hebben over de omzetten en pas dan besluiten of wij aanscherping van de handhaving op zijn plaats vinden." (p.664)

Minister Klink toont echter geen begrip voor het verzet van de kleine cafés zonder personeel tegen het rookverbod in de horeca. In november 2008 stelt hij in een overleg met de vaste commissie van de Tweede Kamer voor VWS[50]:

"… De slechte naleving in met name de kleine horeca en cafés was toen de reden om het toezicht te verschuiven naar de cafés. De Kamer had mij aanvankelijk gevraagd

49. TK, 2008-2009, vergaderingnr.10, p.662-678 (dossiernr.22894).
50. TK, 2008–2009, 22894, nr.207.

om bij de handhaving proportioneel op te treden en een gewenningsperiode te laten ontstaan. Er werd begonnen met een waarschuwing en pas daarna zou een boete worden opgelegd. … Ik heb op 7 oktober gezegd het anders te gaan doen. … Wij gingen van een waarschuwing naar een bestuurlijke boete. Het boeteregime is vervolgens uitgebreid. Aan de oproep tot strengere handhaving, die overigens ook van Koninklijke Horeca Nederland kwam, een paar weken geleden, heb ik ook gehoor gegeven. De overheid is dan geen knip voor de neus waard als het de regels niet handhaaft. Om die reden is in de loop van november, …, besloten om samen te werken met het OM in het kader van de Wet op de economische delicten. … Het regime voor de Wet economische delicten is overigens een stuk strenger dan het bestuurlijke handhavingstraject. Men kan overgaan tot een boete van maximaal € 18 000 en tijdelijke sluiting van een horecagelegenheid." (p.11)

Op 16 december 2008 informeert minister Klink de Tweede Kamer in een brief over de praktische implicaties van de invoering van de horecavrije horeca[51]:

"Volgens het CBS is een veelheid aan factoren (mede) bepalend voor de omzet: prijzen, weersomstandigheden, economie, consumentenvertrouwen, etc. CBS geeft aan geen direct verband te zien met de rookvrije horeca: er is sinds het derde kwartaal 2007 sprake van een negatieve omzettrend voor de horeca. Deze lijn zet zich na de invoering van de rookvrije horeca op 1 juli jl. voort." (p.6)

"Uit een overzicht van horecafaillissementen blijkt dat het aantal cafés en restaurants dat failliet ging sinds de invoering van de rookvrije horeca is afgenomen waarmee de dalende trend sinds 2007 zich voortzet. In totaal gingen 175 horecagelegenheden failliet. Middelgrote en grote zaken met twee of meer man personeel vormden het leeuwendeel van de faillissementen." (p.9-10)

"Uit de onderzoeksgegevens en de informatie die hierboven is toegelicht, blijkt dat er op dit moment geen grond is om vast te stellen dat de rookvrije horeca voor bepaalde sectoren of (sub)groepen buiten proportioneel uitwerkt. Een reden om voor bepaalde sectoren of groepen een uitzondering of buitengewone ondersteuning (compensatie dan wel subsidie) te overwegen, dient zich op basis van deze gegevens niet aan." (p.15)

In oktober 2009 stelt het reviewrapport van Regioplan over de economische gevolgen van het rookverbod in de horeca:

"Vanaf het tweede kwartaal van 2007 stagneren de omzetten in de horeca. Ook in 2008 zette de omzetdaling door. Deze ontwikkeling was (iets later) ook in de detailhandel te zien. Ongeveer de helft van de horecaondernemers heeft te maken met omzetdalingen. Omzetdalingen zijn in de gehele sector zichtbaar zowel bij grote als kleine ondernemingen. Het aantal werknemers in een horecaonderneming heeft geen

51. TK, 2008–2009, 22894, nr.206.

significant verband met de gerapporteerde omzetdalingen. Horecagelegenheden zonder rookvoorziening ervaren vergelijkbare omzetontwikkelingen als horecagelegenheden met rookvoorziening. … Bedrijfseconomische aspecten, macro-economische ontwikkelingen en vergelijkingen met andere sectoren, duiden erop dat de recessie meer invloed heeft gehad op de omzet in de horeca, dan de invoering van het rookverbod. Horecaondernemers noemden in 2008 de invoering van het rookverbod het meest (75%) als oorzaak voor omzetverlies. In 2009 wordt de economische situatie het meest genoemd als oorzaak." (p.23)

In december 2009 rapporteert minister Klink in een brief aan de Tweede Kamer over de resultaten van één jaar rookvrije Horeca[52]:

"Tot slot is duidelijk dat door de economische recessie ook een sector als de horeca forse tegenwind heeft. De (omzet)ontwikkelingen in de horecasector zijn echter niet uniek; uit de rapportages blijkt dat de horecasector geen afwijkende ontwikkelingen heeft doorgemaakt in vergelijking met andere sectoren of de gehele economie. Net als in mijn brief van 16 december 2008 is mijn conclusie dat er vooralsnog geen grond is om vast te stellen dat de rookvrije horeca voor bepaalde sectoren of (sub)groepen buiten proportioneel zou uitwerken." (p.5)

In november 2010 schrijft minister Schippers in een brief aan de Tweede Kamer[53]:

"In het regeerakkoord is het volgende overeengekomen met betrekking tot het rookverbod: «Het huidige rookverbod wordt versoepeld. …" (p.1)

"De voornaamste reden voor dit traject was een alternatief te kunnen bieden aan kleine cafés, die geen mogelijkheid hadden om een rookruimte te creëren. Voor deze categorie wordt nu een uitzondering op het rookverbod gemaakt." (p.3)

In januari 2011 stelt Tweede Kamerlid Uitslag in een algemeen overleg met minister Schippers[54]:

"… Als de kleine horeca het rookverbod opgelegd krijgt, draaien we dit type bedrijven de nek om. Dat is niet de bedoeling. De mensen die gebruikmaken van de kleine horeca, zien deze voorziening als een soort huiskamer. De praktijk is nu dat, als er een sigaretje moet worden gerookt, het hele café leeg gaat. Men gaat buiten een sigaret roken en komt dan weer binnen. Maar los daarvan zal ik er niet omheen draaien; er is hierbij sprake van een deal in de huidige coalitie. Daarom staat het nu in het regeerakkoord." (p.9)

Voor zover ik het kan beoordelen is minister Klink op basis van het rapport van TNS NIPO (2009) in zijn brief d.d. 1 december 2009 tot de volgende conclusie

52. TK, 2009–2010, 32011, nr.3.
53. TK, 2010-2011, 22894, nr.289.
54. TK, 2010–2011, 32011, nr.15.

gekomen: vooralsnog is er geen grond om vast te stellen dat de rookvrije horeca voor bepaalde sectoren of (sub)groepen buiten proportioneel uitwerkt. Deze conclusie van minister Klink is meer gebaseerd op het ontbreken van gedetailleerde onderzoekscijfers, waardoor hierover geen conclusies kunnen worden getrokken (er is geen grond om vast te stellen), dan op duidelijke aanwijzingen dat kleine cafés zonder personeel niet buitenproportioneel door het rookverbod worden geraakt.

Op basis van het rapport van TNS NIPO (2009) kan niet worden geconcludeerd of het rookverbod in de horeca al dan niet buitenproportioneel uitwerkt voor bepaalde (sub)groepen c.q. kleine cafés zonder personeel. De reden dat dit niet kan, is dat de cijfers in dit rapport over veranderingen in de omzetontwikkeling daarvoor te globaal zijn (zie voor de onderbouwing van deze stelling bijlage 10). Overigens kan op basis van het rapport van TNS NIPO (2009), wegens het ontbreken van gedetailleerde cijfers, ook niet worden geconcludeerd dat kleine cafés zonder personeel wel buiten proportioneel door het rookverbod worden geraakt.

Aspect van gelijke toegang tot gezondheidszorg en preventie
Dit is niet van toepassing.

Aspect van verdeling naar behoefte van gezondheidszorg en preventie
Dit is niet van toepassing.

Aspect van legitimiteit
Het aspect van legitimiteit betreft de vraag of de preventiemaatregel niet strijdig is met het vigerende recht:
- wet- en regelgeving;
- nationale en internationale verdragen;
- rechtsgeldige overeenkomsten/ afspraken;
- e.d..

In geval van de preventiemaatregel 'wettelijk rookverbod in de horeca' betreft het aspect van legitimiteit m.i. de vraag of deze preventiemaatregel niet in strijd is met:

A. de Tabakswet;
B. het WHO-Kaderverdrag inzake tabaksontmoediging (2003);
C. de afspraken van minister Hoogervorst met KHN over het stappenplan ter zelfregulering van het rookbeleid in de horeca (2004);
D. de Aanbeveling van de Raad van Europa betreffende rookvrije ruimten (2009).

A. Congruentie van het wettelijk rookverbod in de horeca met de Tabakswet

In een brief aan de Tweede Kamer d.d. 8 juli 2009 legt minister Klink uit waarom het volgens de gerechtshoven in Den Bosch en Leeuwarden niet legitiem is om op basis van de Tabakswet aan horeca-inrichtingen zonder personeel een rookverbod op te leggen. Minister Klink besluit naar aanleiding van deze rechterlijke uitspraken het rookverbod in cafés zonder personeel op te schorten. Wel gaat het Openbaar Ministerie tegen deze uitspraken van de gerechtshoven in den Bosch en Leeuwarden in cassatie bij de Hoge Raad.[55] :

> ***"Uitspraken gerechtshof Den Bosch en gerechtshof Leeuwarden***
> *Het Gerechtshof in Den Bosch concludeerde op 12 mei 2009 in het hoger beroep van het Openbaar Ministerie tegen de uitspraak van de rechtbank in Breda inzake café Victoria dat artikel 3 van het Besluit uitvoering rookvrije werkplek, horeca en andere ruimten (Besluit Uitvoering) bij gebreke van een deugdelijke wettelijke grondslag onverbindend is voor zover het verplicht tot het instellen, aanduiden en handhaven van een rookverbod in horeca-inrichtingen zonder personeel. Naar het oordeel van het hof biedt de uit artikel 11a, vierde lid, van de Tabakswet voortvloeiende bevoegdheid om gebouwen aan te wijzen waar maatregelen als bedoeld in artikel 10, eerste lid, van de Tabakswet dienen te worden getroffen onvoldoende grondslag om gebouwen aan te wijzen waar specifiek een rookverbod moet worden ingesteld, aangeduid en gehandhaafd. Het Openbaar Ministerie (OM) is in cassatie gegaan tegen de uitspraak van het gerechtshof in Den Bosch.*
>
> *Het gerechtshof in Leeuwarden sprak café De Kachel in het hoger beroep tegen de uitspraak van de rechtbank in Groningen op 3 juli 2009 vrij. Het gerechtshof is van oordeel dat artikel 10, lid 1 en/of 2, van de Tabakswet café De Kachel niet de verplichting oplegt om een rookverbod in te stellen. De verwijzing in artikel 11a, vierde lid, van de Tabakswet naar artikel 10, eerste lid, van de Tabakswet biedt onvoldoende grond om een verplichting op te leggen een rookverbod in te stellen. Het OM stelt ook tegen dit arrest cassatie in bij de Hoge Raad.*
>
> ***Welke consequentie verbind ik aan de uitspraken?***
> *Van begin af aan heb ik aangegeven dat ik hecht aan een gelijk speelveld voor de horeca en een gelijke behandeling van alle horeca. Dit om oneerlijke concurrentie zoveel mogelijk te voorkómen. Uw Kamer heeft dit uitgangspunt onderschreven. Met de uitspraken van de gerechtshoven in Den Bosch en Leeuwarden is een onduidelijke situatie ontstaan met betrekking tot de toepassing van de regelgeving door horecaondernemers zonder personeel. Die onduidelijkheid wil ik met genoemde uitgangspunten in het achterhoofd graag wegnemen.*

55. TK, 2008–2009, 32011, nr.1.

Concreet betekent dit dat ik op korte termijn voornemens ben om het Besluit uitvoering rookvrije werkplek, horeca en andere ruimten aan te passen. Het voornemen is er op gericht om voor alle horeca-inrichtingen een eensluidende verplichting te laten gelden, namelijk de verplichting om zodanige maatregelen te treffen dat van een horeca-inrichting gebruik kan worden gemaakt en de werkzaamheden daarin kunnen worden verricht zonder daarbij hinder of overlast van roken te ondervinden. Bij de afwegingen die met dit voornemen zijn gemoeid, zal ik de overwegingen en de uitkomsten van rechterlijke uitspraken betrekken. Ik zal u zo spoedig mogelijk ingevolge de voorhangprocedure ex artikel 12 van de Tabakswet een algemene maatregel van bestuur op basis van de Tabakswet te doen toekomen ter wijziging van het Besluit uitvoering rookvrije werkplek, horeca en andere ruimten. … Verder beraad ik me op mogelijke wijziging van de huidige formulering van de rookverboden in de artikelen 10, 11 en 11a van de Tabakswet.

Welke consequenties heeft dit voor de handhaving?
De handhaving voor horecabedrijven mét personeel zal uiteraard doorgaan omdat op die categorie de rechterlijke uitspraken niet van toepassing zijn. De handhaving voor horecabedrijven zonder personeel wordt tijdelijk opgeschort. Dit betekent dat de VWA voorlopig geen horecabedrijven zonder personeel zal controleren op naleving van het rookverbod. Voor wat betreft boetes die reeds zijn opgelegd, zal ik de procedures ook tijdelijk opschorten." (p.1-2)

Op 11 september 2009 stuurt minister Klink een brief aan de Tweede Kamer met als bijlage het 'Ontwerpbesluit, houdende verduidelijking van het Besluit uitvoering rookvrije werkplek, horeca en andere ruimten'. In deze brief schrijft hij ondermeer[56]:

"Wijziging Tabakswet
… Echter ook na aanpassing van het Besluit uitvoering zou er nog steeds grond kunnen zijn voor voortgaande discussie. Ik acht dat ongewenst. Uitgangspunt in de Tabakswet is steeds geweest dat in openbare ruimten kan worden gewerkt en verbleven zonder daarbij hinder of overlast van roken te ondervinden. Daarbij gaat het dus zowel om de bescherming van werknemers als om bescherming van bezoekers. Bij de rookvrije horeca speelde regelmatig de vraag op voor wie/welke kring van mensen de maatregelen moesten worden genomen. Horecaondernemers met personeel zijn immers verplicht primair hun personeel te beschermen, terwijl horecaondernemers zonder personeel primair het bezoekerspubliek beschermen. Ook dit pleit ervoor om het begrip «rookverbod» rechtstreeks in de Tabakswet op te nemen zodat duidelijk is dat in voor publiek toegankelijke gebouwen een rookverbod geldt. Kortom, een wijziging van de Tabakswet biedt mijns inziens ook voor de lange termijn de meeste rechtszekerheid, rechtsgelijkheid en duidelijkheid voor werkgevers, werknemers en

56. TK, 2008–2009, 32011, nr.2.

bezoekers van de horeca. Het biedt duidelijkheid over de bedoeling van de wetgever, over wie verantwoordelijk is voor het nemen van maatregelen tegen tabaksrook en over wat die maatregelen moeten zijn. … Daarom zet ik, naast de aanpassing van het Besluit uitvoering, een wijziging van de Tabakswet in gang." (p.2-3)

In de nota van toelichting bij het Ontwerpbesluit ter aanpassing van het 'Besluit uitvoering' (bijlage bij TK, 2008-2009, 32011, nr.2) geeft minister Klink een samenvatting van de problemen m.b.t. de legitimiteit van het rookverbod in cafés zonder personeel:

> *"Het debat in het afgelopen jaar rondom artikel 3, eerste lid, aanhef en onder a, van het Besluit uitvoering is langs diverse lijnen gevoerd, maar lijkt zich toe te spitsen op het antwoord op de vraag of artikel 11a, vierde lid, van de Tabakswet voldoende grondslag biedt om in het Besluit uitvoering een rookverbod voor de horeca zonder personeel af te kondigen. Het debat over deze vraag is nog niet ten einde. Zoals bekend heeft het Openbaar Ministerie tegen een aantal van de hierboven genoemde rechterlijke uitspraken cassatie bij de Hoge Raad ingesteld. Ook de wetenschap is verdeeld. Een deel zit op dezelfde lijn als de eerder bedoelde uitspraken, terwijl in het andere deel - meestal op wetshistorische gronden – de conclusie wordt getrokken dat er geen valide aanleiding bestaat om het rookverbod in de horeca onverbindend te achten wegens het ontbreken van een toereikende wettelijke grondslag."* (p.3-4)

Uiteindelijk beslist de Hoge Raad dat het rookverbod in de horeca, zoals dat geldt sinds 1 juli 2008, ook rechtsgeldig (legitiem) is in de horeca zonder personeel. Het ministerie van VWS schrijft hierover op 23 februari 2010 in een nieuwsbericht op haar website:

> *"De Hoge Raad heeft vandaag uitspraak gedaan over het rookverbod in de horeca. Minister Klink van Volksgezondheid, Welzijn en Sport (VWS) reageert verheugd op de uitspraak. De Hoge Raad komt tot de conclusie dat het rookverbod in de horeca, geldend sinds 1 juli 2008, ook geldt voor horecagelegenheden zonder personeel.*
>
> *Afgelopen zomer [2009] werd de handhaving van het rookverbod bij horeca zonder personeel tijdelijk opgeschort naar aanleiding van uitspraken van de gerechtshoven in Den Bosch en Leeuwarden. Het OM (Openbaar Ministerie) ging in cassatie. Met de uitspraak is een einde gekomen aan een periode van onduidelijkheid over de reikwijdte van de wet. Het rookverbod geldt voor de gehele horeca."*

A. Nadere analyse van congruentie van het wettelijk rookverbod in de horeca met de Tabakswet (vervolg van A.)

Indien een preventiemaatregel bestaat uit een wettelijk verbod kunnen problemen met de legitimiteit (rechtsgeldigheid) van de preventiemaatregel zowel de inhoud als de vorm van de wetgeving betreffen. De legitimiteitsproblemen van het wettelijk rookverbod in de horeca betreffen zowel de inhoud als de vorm van betref-

fende wet- en regelgeving. Overigens beperken deze problemen zich tot de toepassing van het wettelijk rookverbod in horecagelegenheden zonder personeel (en in de praktijk tot kleine cafés zonder personeel).

De discussie over de legitimiteit van het wettelijk rookverbod in de horeca concentreert zich rond drie problemen:
1. De doelstelling van het wettelijk rookverbod in de horeca is beperkt tot de bescherming van werknemers in de horeca tegen passief roken, terwijl het uitgangspunt van de Tabakswet is de bescherming van werknemers en bezoekers van openbare ruimten tegen de blootstelling aan tabaksrook.
2. Artikel 11a van de Tabakswet, op basis waarvan het rookverbod in horeca zonder personeel is geregeld, betreft het recht op een rookvrije werkplek. Het recht op een rookvrije werkplek is een onlogisch uitgangspunt voor een rookverbod in de horeca zonder personeel.
3. De Tabakswet schrijft voor de horeca met personeel geen rookverbod voor, maar regelt dat het personeel zonder hinder of overlast van tabaksrook zijn werkzaamheden moet kunnen verrichten. Het 'Besluit uitvoering rookvrije werkplek, horeca en andere ruimten' (verder te noemen 'uitvoeringsbesluit') regelt dat in de horeca zonder personeel een rookverbod geldt. In theorie is een goed functionerend ventilatiesysteem in de horeca met personeel wel toegestaan (geen hinder of overlast), maar in de horeca zonder personeel niet (rookverbod). Dit impliceert rechtsongelijkheid.

Ad.1
Uit het hierboven genoemde eerste probleem vloeien het hier bovengenoemde tweede en derde probleem voort. Het is inderdaad niet in overeenstemming met het uitgangspunt van de Tabakswet, dat de bescherming van bezoekers in de horeca tegen passief roken, geen doelstelling is van de Tabakswet. Echter, de Tabakswet regelt de bescherming van werknemers en bezoekers in openbare ruimten tegen passief roken. De Tabakswet verplicht de overheid echter niet om bezoekers in alle openbare ruimten – waaronder de horeca – tegen passief roken te beschermen. Daarom acht ik de beperkte doelstelling van het wettelijk rookverbod in de horeca (enkel de bescherming van werknemers tegen passief roken), weliswaar niet in lijn met de Tabakswet, maar ook niet strijdig met de Tabakswet. In §10.6 heb ik reeds betoogd waarom de beperkte doelstelling van het wettelijk rookverbod in de horeca niet ongerechtvaardigd is.

Ad. 2
Het tweede probleem kan worden gezien als een onvolkomenheid in de vormgeving of als een fundamentele inhoudelijke fout. Als men het tweede probleem

opvat als een onvolkomenheid in de vormgeving, betekent dit dat men van opvatting is dat het rookverbod in de horeca zonder personeel uitsluitend een economisch doel dient, namelijk het waarborgen van een gelijk economisch speelveld tussen de horeca zonder en met personeel. In dat geval zou men het rookverbod in de horeca zonder personeel eigenlijk moeten baseren op een economische wet en niet op de Tabakswet, of zou men voor het rookverbod in de horeca zonder personeel een plaats in de Tabakswet moeten inruimen buiten artikel 11a dat het recht op een rookvrije werkplek regelt. De Hoge Raad vindt echter niet dat het rookverbod in de horeca zonder personeel door deze onvolkomenheid zijn rechtsgeldigheid verliest.

Minister Klink maakt er echter een fundamenteel inhoudelijk probleem van door in zijn brief d.d. 11 september 2009 te stellen dat het rookverbod in de horeca ook tot doel heeft om bezoekers tegen passief roken te beschermen. De bescherming van bezoekers tegen passief roken in de horeca is niet geregeld in de Tabakswet en niet in het 'Besluit uitvoering' en daarom beslist minister Klink dat beiden aangepast moeten worden. Mogelijk realiseert minister Klink zich dat een wettelijk rookverbod in de horeca zonder personeel, uitsluitend om een gelijk economisch speelveld te realiseren, op termijn niet houdbaar is. Door aan de horeca zonder personeel een rookverbod op te leggen, lijkt immers in de praktijk juist economische ongelijkheid te ontstaan. Als het wettelijk rookverbod in de horeca ook tot doel krijgt om de bezoekers in de horeca tegen passief roken te beschermen, betekent dit een grote inhoudelijke wijziging van het rookverbod met gevolgen voor (bijna) alle voorgaande criteria van het model. Het is dan niet meer dezelfde preventiemaatregel (zie §10.6). Minister Schippers (de opvolgster van minister Klink) besluit echter deze wijziging niet door te voeren.

Ad. 3
Het derde probleem betreft de juridische ongelijkheid tussen enerzijds de horeca met personeel, die moet voorkomen dat het personeel hinder of overlast ten gevolge van passief roken ondervindt, en anderzijds de horeca zonder personeel, die moet voorkomen dat er wordt gerookt (er geldt een rookverbod). Dit zie ik niet als een fundamenteel inhoudelijk probleem, maar als een onvolkomenheid in de vormgeving c.q. formulering van de wetstekst. De wetgever heeft niet de bedoeling gehad om op dit punt ongelijkheid tussen de horeca met en zonder personeel te bewerkstelligen. Integendeel, minister Klink heeft zich juist ingespannen om een (economisch) gelijk speelveld voor de horeca met en zonder personeel te realiseren. Een wijziging van de formulering van het 'Besluit uitvoering' en/of de Tabakswet, waarbij op dit punt voor de horeca met en zonder personeel dezelfde verplichtingen gaan gelden, acht ik daarom geen ingrijpende wijziging van de pre-

ventiemaatregel. Omdat in horeca met personeel op dat moment ook nog geen ventilatiesystemen als alternatief voor het rookverbod waren toegepast, was er in de praktijk op dit punt eigenlijk nog geen sprake geweest van rechtsongelijkheid.

B. Congruentie van het wettelijk rookverbod in de horeca met de het WHO-Kaderverdrag inzake tabaksontmoediging

Het 'WHO-Kaderverdrag inzake tabaksontmoediging' is vastgesteld in mei 2003[57]. In januari 2005 is het door Nederland geratificeerd en in april 2005 voor Nederland in werking getreden[58]. Relevant voor het wettelijk rookverbod in de horeca is vooral artikel 8:

> *"Artikel 8. Bescherming tegen de blootstelling aan tabaksrook*
> 1. *De Partijen erkennen dat wetenschappelijk bewijsmateriaal onomstotelijk heeft aangetoond dat blootstelling aan tabaksrook leidt tot dood, ziekte en arbeidsongeschiktheid.*
> 2. *Elke Partij neemt binnen de bestaande nationale rechtsbevoegdheid zoals bepaald in het nationaal recht doeltreffende wetgevende, uitvoerende, bestuurlijke en/of andere maatregelen aan, voert deze uit, en bevordert deze maatregelen op andere niveaus van rechtsbevoegdheid. Deze maatregelen voorzien in bescherming tegen blootstelling aan tabaksrook in binnen gebouwen gelegen werkplekken, het openbaar vervoer, binnen openbare gebouwen en, naar gelang van het geval, op andere openbare plaatsen."*

Van artikel 8 kan worden afgeleid dat ook bezoekers in de horeca tegen passief roken dienen te worden beschermd. De horeca valt immers onder de aanduiding "*binnen openbare gebouwen en, naar gelang van het geval, op andere openbare plaatsen*". Artikel 8 verplicht echter niet tot het instellen van een wettelijk rookverbod ter bescherming van bezoekers in de horeca tegen passief roken. In de eerste plaats spreekt artikel 8 niet over een rookverbod, maar over *"bescherming tegen blootstelling aan tabaksrook"*. In de tweede plaats spreekt artikel 8 niet over wettelijke maatregelen, want *"andere maatregelen"* zijn ook toegestaan.

In juni 2007 schrijft Minister Klink in een brief aan de Tweede Kamer dat hij met het wettelijk rookverbod in de horeca, dat hij voorstelt, primair werknemers en secundair bezoekers in de horeca tegen passief roken wil beschermen[59]:

> *"Ik heb besloten om niet mee te gaan in het voorstel van KHN omdat: dan niet alle werknemers (secundair: bezoekers) in de horeca beschermd worden tegen tabaksrook*

57. http://wetten.overheid.nl
58. Tractatenblad van het Koninkrijk der Nederlanden. Jaargang 2005, nr.72.
59. TK, 200602007, 30800 XVI, nr.149.

(ongelijke behandeling) en juist de werknemers die de hoogste blootstelling aan tabaksrook hebben (cafés, discotheken), worden uitgezonderd …"

De bovenstaande motivering van minister Klink van het wettelijk rookverbod in de horeca past goed in het kader voor het tabaksontmoedigingsbeleid van het 'WHO-kaderverdrag inzake tabaksontmoediging'.

In juni 2009 vindt in de Tweede Kamer een spoeddebat plaats over de gevolgen van een rechterlijke uitspraak over het wettelijk rookverbod in de horeca. In dit debat stelt minister Klink dat de bescherming van bezoekers in de horeca tegen passief roken geen doelstelling is van het rookverbod[60]:

> *"De grondslag en het aangrijpingspunt voor en het oogmerk van deze wet- en regelgeving is om de werknemer te beschermen, niet meer en niet minder. Ik verwees naar de spin-off van het geheel, dus naar de effecten. De effecten zijn dat ook de horecabezoeker er uiteindelijk baat bij heeft. Ik zeg niet dat dit de reden is geweest voor de wet- en regelgeving. Dat zal het wat mij betreft ook niet worden. Het is wel een bijeffect van de maatregelen die wij hebben genomen. Ik geef maar even aan dat het een gelukkig bijeffect is, want het gaat hier om een miljoen mensen die aan astma lijden in ons land en die daadwerkelijk veel hinder ondervinden van het roken in een besloten ruimte."* (p.6623)

Deze formulering van minister Klink van de doelstelling van het wettelijk rookverbod in de horeca betekent een afzwakking van zijn formulering van deze doelstelling in juni 2007. Deze afzwakking is niet in de geest van het 'WHO-Kaderverdrag inzake tabaksontmoediging' en de consequentie is dat minister Klink zich genoodzaakt ziet om de handhaving van het wettelijk rookverbod in cafés zonder personeel op te schorten. Strijdig met de geest van het WHO-kaderverdrag is in dit geval echter niet de preventiemaatregel die wel wordt toegepast (wettelijke bescherming van werknemers in de horeca tegen passief roken), maar de preventiemaatregel die niet wordt toegepast (wettelijke bescherming van bezoekers in de horeca tegen passief roken).

Bij versie 5 van mijn model heb ik beargumenteerd dat het model wel betrekking heeft op de vraag ***'moet de preventiemaatregel wel worden uitgevoerd?'***, maar niet op de vraag ***'welke preventiemaatregelen moeten worden uitgevoerd?'***. De kritiek die men op basis van het WHO-Kaderverdrag op de afzwakking van de doelstelling van het wettelijk rookverbod door minister Klink kan hebben, betreft de tweede vraag: 'welke preventiemaatregelen moeten worden uitgevoerd (c.q. welke

60. TK, 2008-2009, vergaderingnr.84, p.6613-6629 (dossiernr.22894).

preventiemaatregelen worden onterecht niet uitgevoerd)?'. Binnen het kader (de reikwijdte) van het model is deze afzwakking daarom geen reden om (de doelstelling van) het wettelijk rookverbod in de horeca ongerechtvaardigd te noemen.

In november 2010 stelt minister Schippers[61]:

> *"… Door de uitzondering wordt recht gedaan aan de keuzevrijheid van de consument, terwijl ook de handhaafbaarheid en de acceptatie van de norm zal worden vergroot."* (p.1-2)
>
> *"Om de herkenbaarheid van buitenaf voor potentiële klanten en toezichthouders te bevorderen zullen rookcafés» verplicht worden op of bij de ingang via een bordje aan te geven dat zij een rookcafé vormen. Deze aanduiding geeft de bezoekers de vrijheid zelf te kiezen of zij een café waar nog gerookt wordt, willen betreden of niet."* (p.2)

De uitzondering waarover minister Schippers spreekt, betreft de uitzondering van kleine cafés zonder personeel van het wettelijk rookverbod in de horeca.

Minister Schippers wil bezoekers van cafés beschermen tegen passief roken door hen keuzevrijheid te bieden tussen cafés waar wel en niet mag worden gerookt. Dit is niet (evident) strijdig met artikel 8 van het WHO-kaderverdrag inzake tabaksontmoediging. Wetstechnisch is het geen achteruitgang in bescherming van bezoekers ten opzichte van het wettelijk rookverbod in de horeca dat door minister Klink was ingesteld. De tekst van de Tabakswet is immers alleen gericht op de bescherming van werknemers in de horeca tegen passief roken.

C. Afspraken van minister Hoogervorst van VWS met KHN over het stappenplan ter zelfregulering van het rookbeleid in de horeca (2004)

Bij de beoordeling van het wettelijk rookverbod in de horeca aan de hand van het criterium 'geschiktheid' heb ik geschreven dat de wijze waarop minister Klink het wettelijk rookverbod in de horeca heeft aangekondigd en ingevoerd geen schoonheidsprijs verdient. De voorganger van minister Klink – minister Hoogervorst – had namelijk met KHN afgesproken dat de zelfregulering van het rookbeleid in de horeca in ieder geval t/m 2008 zou duren, als de horeca de doelstellingen van het afgesproken 'stappenplan ter zelfregulering van het rookbeleid in de horeca' zou realiseren. De horeca heeft deze doelstellingen behaald en toch heeft minister Klink eenzijdig de afspraken over de zelfregulering van het rookbeleid tussen de overheid en KHN opgezegd.

---

61. TK, 2010–2011, 22894, nr.289.

Bij de beoordeling van het wettelijk rookverbod aan de hand van het criterium 'geschiktheid' heb ik gesteld, dat de wijze waarop minister Klink het rookverbod heeft aangekondigd en ingevoerd de procedurele rechtvaardigheid betreft en dat de beoordeling hiervan buiten het kader van het model valt. Bij de beoordeling van het wettelijk rookverbod in de horeca loop ik er echter tegenaan dat de wijze waarop minister Klink dit rookverbod heeft aangekondigd en ingevoerd, van invloed is op de legitimiteit van dit rookverbod[62]. De reden is dat de legitimiteit van de preventiemaatregel zowel inhoudelijke als procedurele voorschriften of afspraken kan betreffen.

De reden dat ik er in hoofdstuk 4 voor heb gekozen om de ***procedurele rechtvaardigheid geen onderdeel*** te maken van het model, is dat de ontwikkeling van een beoordelingskader voor de procedurele rechtvaardigheid een studie op zich vergt. Het proces van ontwikkeling en implementatie van een vrijheidsbeperkende leefstijlbeïnvloedende interventie kan jaren duren en kent vele informele en formele momenten (zie §10.3). De ontwikkel- en implementatieprocedure betreffen een beschrijving van de 'ijkpunten' in dit informele en formele ontwikkel- en implementatieproces. Belangrijke vragen zijn hierbij op welke momenten en op welke wijze deskundigen en belanghebbenden inspraak krijgen bij de ontwikkeling en implementatie van de preventiemaatregel en hoe het best vorm kan worden gegeven aan een rationele en democratische besluitvorming over de vorm en implementatie van de preventiemaatregel.

In de praktijk is het mogelijk dat de ontwikkel- en besluitvormingsprocedure op basis waarvan de preventiemaatregel tot stand komt ernstige gebreken vertoont, terwijl alle betrokken deskundigen en belanghebbenden toch tevreden zijn over het eindresultaat: tevredenheid over het ontwerp (1e zeef van het model), over de effecten en neveneffecten (2e zeef) en over de implementatie (3e zeef) van de preventiemaatregel. Dit kan bijvoorbeeld het geval zijn als bij de ontwikkeling en implementatie van de preventiemaatregel nauwelijks iemand inspraak heeft gehad (er is sprake geweest van een ondemocratisch proces), maar alle betrokkenen wel tevreden zijn met het eindresultaat. In dat geval kan de inhoud van de preventiemaatregel aan de hand van het model gerechtvaardigd worden, terwijl de ontwikkel- en besluitvormingsprocedure niet gerechtvaardigd kunnen worden.

Het is echter ook mogelijk dat de inhoudelijke beoordeling van de preventiemaatregel aan de hand van het model en de ontwikkel- en besluitvormingsprocedure

62. Het aspect 'legitimiteit' is onderdeel van het 10e criterium 'rechtvaardigheid' van het model.

interacteren. Gebreken in de besluitvormingsprocedure kunnen bijvoorbeeld leiden tot problemen met de legitimiteit van de preventiemaatregel (legitimiteit is een aspect van het criterium 'rechtvaardigheid' van het model) of problemen met het draagvlak voor de preventiemaatregel (draagvlak is een criterium van het model). Op basis van deze overweging kies ik ervoor om de ontwikkel- en besluitvormingsprocedure te betrekken bij de beoordeling van de preventiemaatregel aan de hand van het model, voor zover deze procedures van invloed zijn op de inhoudelijke beoordeling.

Bij de beoordeling van de gerechtvaardigdheid van de preventiemaatregel aan de hand van het model ***kunnen procedurele aspecten worden meegewogen***. Afhankelijk van de casus dient per criterium te worden afgewogen in hoeverre procedurele aspecten relevant zijn voor de inhoudelijke beoordeling van de gerechtvaardigdheid van de preventiemaatregel. Ik vermoed dat dit vooral bij het criterium 'rechtvaardigheid' en bij de criteria van de 3e zeef van het model het geval kan zijn.

In augustus 2004 stuurt KHN een brief aan minister Hoogervorst met daarin een aangepast stappenplan ter zelfregulering van het rookbeleid in de horeca. Dit aangepaste plan betreft de periode 2005 t/m 2008[63]. In reactie op deze brief schrijft minister Hoogervorst aan KHN met dit aangepaste stappenplan in te stemmen[64]:

> *"Aan het Bestuur van Koninklijk Horeca Nederland … Ook in Nederland zal de horeca onder invloed van bovengenoemde maatschappelijke ontwikkelingen op termijn rookvrij worden. … In overleggen over dit onderwerp met onder andere de Tweede Kamer en Koninklijk Horeca Nederland (KHN) heb ik duidelijk gemaakt dat bescherming van werknemers tegen tabaksrook ook in de horeca noodzakelijk is, maar dat – gegeven genoemde overgangssituatie – een zelfreguleringtraject met maatregelen die de horeca op korte termijn kan uitvoeren, de voorkeur verdient. In december 2003 heeft u hiervoor een eerste voorstel ingediend. Hierover is op een open en constructieve wijze overleg gevoerd tussen KHN en mijn medewerkers. Het resultaat van dat overleg heeft u in uw brief van 26 augustus 2004 toegelicht. Dit doet mij deugd en ik kan u mededelen in te kunnen stemmen met dit aangepaste stappenplan. … Hoogachtend, de Minister van Volksgezondheid, Welzijn en Sport, H. Hoogervorst".*

M.i. kan deze brief worden gezien als een rechtsgeldige afspraak tussen de overheid en KHN over de (periode van) zelfregulering van het rookbeleid in de horeca. Als minister Klink in juni 2007 de Tweede Kamer informeert over zijn voornemen tot

63. Brief van KHN over rookbeleid, d.d. 26 augustus 2004. Bijlage bij Kamerstuk 29200-XVI nr.278.
64. Brief aan KHN over Stappenplan rookvrije horeca, te raadplegen sinds 8 september 2004. Bijlage bij Kamerstuk 29200-XVI nr.278.

invoering van een wettelijk rookverbod in de horeca per 1 juli 2008, gaat hij voorbij aan de afspraak over de periode van zelfregulering (2005 t/m 2008) tussen de overheid en KHN. KHN heeft wat betreft de zelfregulering van het rookbeleid in de horeca een grote inspanning geleverd en de afgesproken kwantitatieve doelstellingen behaald. In 2003 schreef minister Hoogervorst in dit verband aan het bestuur van KHN[65]:

> *"Begin december heb ik het stappenplan naar een rookvrije horeca van Koninklijk Horeca Nederland (KHN) ontvangen. Ik wil u complimenteren met het feit dat KHN dit plan in zo'n korte tijd tot stand heeft weten te brengen. Daarmee voldoet u aan de afspraak die in het bestuurlijk overleg van 8 september 2003 is gemaakt om voor kerst 2003 een stappenplan naar een rookvrije horeca op te stellen. Ik ben verheugd te zien dat KHN in deze zijn verantwoordelijkheid neemt en aan het werk is gegaan om de mogelijkheden voor stappen richting een rookvrije horeca voor de verschillende subsectoren binnen de horeca in kaart te brengen."*

In plaats van dat KHN door de overheid voor haar inspanningen wordt beloond, worden de behaalde resultaten door minister Klink bekritiseerd en beslist minister Klink eenzijdig tot het impliciet openbreken van het overeengekomen stappenplan door per 1 juli 2008 een wettelijk rookverbod voor de hele horeca in te voeren. In een debat met minister Klink citeren Tweede Kamerleden KHN, die naar aanleiding hiervan van een *"onbetrouwbare overheid"* spreekt (zie §13.6.2). Daarom verbaast het mij dat de Tweede Kamer met de invoeringsdatum van het wettelijk rookverbod in de horeca per 1 juli 2008 heeft ingestemd. M.i. is het eenzijdig vaststellen van ***deze invoeringsdatum*** door de overheid ***niet legitiem***, omdat de overheid een afspraak die zij met KHN heeft gemaakt niet nakomt. Het besluit van minister Klink, om na afronding van het stappenplan ***van zelfregulering*** van het rookbeleid in de horeca over te gaan ***naar*** een ***wettelijk*** rookverbod in de horeca, acht ik ***wel legitiem***. De voorganger van minister Klink – minister Hoogervorst – heeft immers van een *"overgangssituatie"* gesproken en voortdurend gesteld dat het einddoel van het overheidsbeleid een rookvrije horeca is.

D. Aanbeveling van de Raad van Europa betreffende rookvrije ruimten (2009)

In november 2009 doet de Raad van de Europese Unie aan haar lidstaten (waaronder Nederland) de volgende aanbeveling[66]:

65. Afschrift brief min VWS aan KHN over stappenplan rookvrije horeca. Bijlage bij Kamerstuk 29200-XVI nr.157.
66. Publicatieblad van de Europese Unie (2009/C 296/02).

"De Raad van de Europese Unie, ... Overwegende hetgeen volgt: ... (6) Maatregelen op basis van vrijwilligheid op nationaal niveau hebben naar gebleken is weinig of geen effect gehad wat het verminderen van de blootstelling aan tabaksrook betreft. Bindende nationale wetgeving, op de naleving waarvan strikt wordt toegezien, is een effectieve manier om mensen adequaat te beschermen tegen de gezondheidsrisico's van secundaire tabaksrook. (7) Alle burgers hebben recht op een hoog niveau van gezondheidsbescherming en dienen beschermd te worden tegen blootstelling aan tabaksrook. ...

Beveelt aan dat de lidstaten: 1. Zorgen voor effectieve bescherming tegen blootstelling aan tabaksrook in afgesloten werkplekken, afgesloten openbare ruimten, openbare vervoermiddelen, en waar van toepassing ook in andere openbare ruimten, zoals bepaald in artikel 8 van de Kaderovereenkomst van de WHO voor de bestrijding van tabaksgebruik en op basis van de bijgevoegde richtsnoeren inzake de bescherming tegen blootstelling aan tabaksrook die zijn goedgekeurd door de tweede conferentie van de partijen bij de kaderovereenkomst, binnen vijf jaar na het van kracht worden van de overeenkomst voor de betreffende lidstaat, of uiterlijk binnen drie jaar na de goedkeuring van deze aanbeveling; ...".

Deze aanbeveling volgt artikel 8 van het WHO-Kaderverdrag inzake tabaksontmoediging en de daarbij gevoegde richtsnoeren (zie hierboven onder B.). Het gaat hierbij echter om een aanbeveling en de lidstaten zijn niet (wettelijk) verplicht om een aanbeveling op te volgen.

12.4.3 Eindoordeel (criterium rechtvaardigheid)

Het politieke debat over de rechtvaardigheid van het rookverbod in de horeca concentreert zich op de toepassing van het rookverbod in kleine cafés zonder personeel. In november 2008 stelt minister Klink in een overleg met de vaste commissie van de Tweede kamer voor VWS:

"Inderdaad, degenen zonder personeel zouden strikt genomen niet verplicht dienen te worden om een rookvrije werkplek te realiseren. Gegeven concurrentieoverwegingen hebben wij een integrale keuze gemaakt."

In datzelfde overleg stelt Tweede Kamerlid Zijlstra terecht dat de kleine cafés zonder personeel door deze keuze nu juist de dupe worden van concurrentievervalsing, omdat zij wegens ruimtegebrek geen rookruimte kunnen maken:

"Het probleem ligt met name bij de kleine cafés. Er is sprake van oneerlijke concurrentie. Grote horeca kan een rookruimte maken. Kleine horeca kan dat fysiek niet. Het grappige is dat horeca zonder personeel destijds onder de wet moest vallen om te voorkomen dat er onjuiste concurrentie zou optreden. In de praktijk treedt die dus wel degelijk op. Het zijn in de praktijk de kleine kroegen die de pisang zijn."

Als het doel van een maatregel is het voorkomen van concurrentievervalsing, maar de betreffende maatregel leidt juist tot concurrentievervalsing, dan staat de rechtvaardigheid van de toepassing van deze maatregel onder druk. Dan moet worden beoordeeld of de maatregel meer concurrentievervalsing voorkomt dan concurrentievervalsing oplevert. Problematisch aan de redenering van Zijlstra is dat hij stelt dat de concurrentievervalsing wordt veroorzaakt door het al dan niet hebben van een rookruimte binnen. Dit blijkt in de praktijk niet het geval. Tweede Kamerlid Agema, fel tegenstander van het rookverbod in de horeca, stelt in maart 2009:

> "*Bij de cafés waar niet wordt gerookt, is heel duidelijk sprake van een enorme omzetdaling. Dat geldt ook voor de cafés met een rookruimte, want dat is gewoon veel minder gezellig.*"

Het reviewrapport van Regioplan stelt in oktober 2009:

> "*Horecagelegenheden zonder rookvoorziening ervaren vergelijkbare omzetontwikkelingen als horecagelegenheden met rookvoorziening.*"

In de loop van 2009 wordt steeds duidelijker dat het al dan niet beschikken over een rookruimte binnen niet bepalend is voor de omzet van cafés. Dat betekent echter niet dat kleine cafés zonder personeel niet onevenredig hard door het rookverbod geraakt kunnen worden. Er zijn alternatieve verklaringen mogelijk voor de vermeende grote omzetdalingen in deze cafés ten gevolge van het rookverbod. Hierbij kan bijvoorbeeld worden gedacht aan specifieke kenmerken van de klantenkring van deze cafés, een andere gebruiksfunctie van deze cafés (meer een huiskamer dan uitgaansgelegenheid) of problemen van deze cafés met het realiseren van een aantrekkelijke rookvoorziening buiten vanwege het ontbreken van een (reeds bestaand) terras. Teleurstellend is m.i. dat in geen van de onderzoeken die naar de gevolgen van het rookverbod in de horeca zijn uitgevoerd aan deze 'inhoudelijke' aspecten aandacht is besteed. Daarom rest er weinig anders dan het beleid te baseren op omzetcijfers zonder de achterliggende oorzaken te kennen.

Minister Klink stelt in december 2008 en opnieuw in november 2009, dat er geen grond is om vast te stellen dat de rookvrije horeca voor bepaalde sectoren of (sub) groepen buitenproportioneel uitwerkt. Voor wat betreft de subgroep 'kleine cafés zonder personeel' is die grond er inderdaad niet, omdat er onvoldoende (omzet) cijfers over deze subgroep bekend zijn. Op basis van zijn stelling 'dat er geen grond is om vast te stellen dat de rookvrije horeca voor bepaalde sectoren of (sub)groepen buitenproportioneel uitwerkt' besluit minister Klink al in november 2008 om het handhavingsbeleid te verscherpen door te gaan samenwerken met het Openbaar Ministerie (OM) in het kader van de Wet op de economische delicten. Het OM kan vanwege overtreding van het wettelijk rookverbod een boete van maxi-

maal € 18.000 en tijdelijke sluiting van een horecagelegenheid eisen. Kamerlid Zijlstra stelt dat kleine cafés zonder personeel zich tegen het rookverbod verzetten uit ***lijfsbehoud*** en vraagt minister Klink het boeteregime niet te verscherpen, totdat meer cijfers over de omzetdalingen in kleine cafés zonder personeel beschikbaar zijn. Dat minister Klink geen gehoor geeft aan dit verzoek acht ik ***onredelijk en onbillijk c.q. onrechtvaardig***, gegeven het feit dat hij in november 2008 zelf aangeeft dat er nog niet genoeg onderzoeksgegevens beschikbaar zijn om tot een gedegen oordeel te komen:

> *"Nee, de maatregelen staan op papier. Daar vloeien feiten uit voort die de heer Zijlstra en ik niet kennen. Ik constateer dat 16% van de kroegen een rookruimte heeft gemaakt. De heer Zijlstra constateert nu al dat er een nadeel bestaat voor kroegen die geen rookruimte kunnen inrichten. Dat zegt hij op basis van vooronderstellingen en niet op basis van de feiten. Ik ga de feiten bekijken. De Kamer krijgt in, in antwoord op de vraag van de voorzitter, nog voor die met reces gaat een knelpuntenanalyse. Ik hoop en verwacht dat wij daar dan al enig inzicht kunnen bieden. Ik loop daar echter niet op vooruit. Zo-even is gesproken over wijs besturen. Wijs besturen is je baseren op feiten en niet op vermoedens."*

Als ik het belang van minister Klink (voorkomen van concurrentievervalsing) weeg tegen het belang van de kleine horeca zonder personeel (voorkomen van financiële problemen of faillissement) lijkt het mij redelijk en billijk om de kleine cafés zonder personeel het voordeel van de twijfel te gunnen, totdat er degelijke informatie over de omzetdaling in kleine cafés beschikbaar is. Een overweging daarbij is ook het argument, dat ondermeer is aangevoerd door de Tweede Kamerleden Zijlstra (november 2008) en Agema (maart 2009), dat kleine cafés financieel niet draagkrachtig zijn en financieel minder draagkrachtig zijn dan de grotere cafés waarmee zij zouden concurreren.

Mede naar aanleiding van uitspraken van de gerechtshoven in Den Bosch en Leeuwarden over de legitimiteit van het rookverbod in de horeca komt minister Klink uiteindelijk zelf ook tot de conclusie dat het voorkomen van concurrentievervalsing tussen kleine cafés zonder personeel en (grote) cafés met personeel een (te) wankele basis is om kleine cafés zonder personeel een rookverbod op te leggen. In september 2009 stuurt hij een brief aan de Tweede Kamer waarin hij aangeeft de doelstelling van het wettelijk rookverbod in de horeca te willen verbreden naar de bescherming van bezoekers in de horeca tegen passief roken. M.i. zou dit wel een goede rechtvaardigingsgrond voor het opleggen van een rookverbod aan kleine cafés zonder personeel kunnen zijn. Bij een dergelijke ingrijpende verbreding van de doelstelling van de preventiemaatregel is er echter sprake van een heel andere preventiemaatregel, waarvan de gerechtvaardigdheid opnieuw vanaf het eerste criterium 'schadelijkheid' van het model getoetst zou moeten worden.

In november 2010 schrijft minister Schippers een brief aan de Tweede Kamer, waarin zij aangeeft kleine cafés zonder personeel te gaan uitzonderen van het rookverbod in de horeca. Over de reden van dit besluit schrijft zij:

> *"De voornaamste reden voor dit traject was een alternatief te kunnen bieden aan kleine cafés, die geen mogelijkheid hadden om een rookruimte te creëren. Voor deze categorie wordt nu een uitzondering op het rookverbod gemaakt. Bovendien volgt uit het rapport van de CvT en het rapport van RIVM en TNO dat ventilatietechnieken voorlopig geen goed alternatief zijn voor de huidige rookruimtes, zowel qua gezondheidsbescherming, toepassing als handhaving."*

Dit besluit acht ik ***deels onredelijk*** (de onderbouwing is deels irrationeel) ***maar billijk***. Onredelijk (irrationeel) aan bovengenoemd besluit van minister Schippers is dat het al dan niet beschikken over een rookruimte binnen, in de praktijk waarschijnlijk geen grote invloed heeft op de omzet van cafés. Tevens is onredelijk dat in november 2010 nog steeds geen (omzet)cijfers beschikbaar zijn op basis waarvan met voldoende zekerheid objectief kan worden vastgesteld in hoeverre kleine cafés zonder personeel al dan niet onevenredig hard door het rookverbod worden geraakt. Als men, zoals minister Schippers, van opvatting is dat de doelstelling van het wettelijk rookverbod in de horeca beperkt dient te worden tot de bescherming van werknemers tegen passief roken, is het echter redelijk (rationeel) om cafés zonder personeel van het rookverbod uit te zonderen[67]. Als het doel van het opleggen van een rookverbod aan kleine cafés zonder personeel is het voorkomen van concurrentievervalsing, maar het gevolg is dat vooral deze cafés door het rookverbod in de financiële problemen komen, dan schiet het rookverbod voor deze cafés zijn doel voorbij. Bovendien blijkt het in de praktijk met de concurrentievervalsing wel mee te vallen. Tweede Kamerlid Van Gerven heeft dit overtuigend beargumenteerd door te stellen dat als cafés, doordat er gerookt mag worden, meer klanten gaan trekken, ze vanzelf personeel in dienst moeten nemen, waarna het rookverbod weer van toepassing is.

Mijn eindoordeel over de legitimiteit van het wettelijk rookverbod in de horeca luidt als volgt. Op basis van de vigerende wet- en regelgeving en internationale verdragen kan niet worden geconcludeerd dat het wettelijk rookverbod in de horeca niet legitiem is. Wel kan worden gesteld dat de vrijstelling door minister Schippers van kleine cafés zonder personeel van het wettelijk rookverbod in de horeca niet in de geest is van de Tabakswet, het WHO-Kaderverdrag inzake tabaksontmoediging en de Aanbeveling van de Raad van Europa betreffende rook-

67. Minister Schippers heeft het voorstel van minister Klink, om de doelstelling van het rookverbod te verbreden met de bescherming van bezoekers in de horeca tegen passief roken, ingetrokken.

vrije ruimten. Dat minister Klink de invoeringsdatum van het wettelijk rookverbod voor de hele horeca zonder instemming van KHN heeft vastgesteld op 1 juli 2008 acht ik ongerechtvaardigd c.q. niet legitiem in de zin van het model, omdat hij hiermee een afspraak tussen de overheid en KHN heeft geschonden.

12.4.4 Gevolgen van de beoordeling voor het model (criterium rechtvaardigheid)

Tijdens de beoordeling van de 'legitimiteit' (aspect van het criterium 'rechtvaardigheid' van het model) van het wettelijk rookverbod in de horeca ben ik tot de volgende conclusie gekomen. Bij de beoordeling van de gerechtvaardigdheid van de preventiemaatregel aan de hand van het model kunnen procedurele aspecten worden meegewogen. Afhankelijk van de casus dient per criterium te worden afgewogen in hoeverre procedurele aspecten relevant zijn voor de inhoudelijke beoordeling van de gerechtvaardigdheid van de preventiemaatregel. Ik vermoed dat dit vooral bij het criterium 'rechtvaardigheid' en bij de criteria van de 3e zeef 'implementatie' van het model het geval kan zijn. Op zichzelf valt de beoordeling van de procedurele rechtvaardigheid buiten het kader van het model.

12.5 Eindoordeel over de 2e zeef: effecten en neveneffecten

12.5.1 Beoordeling van de effecten en neveneffecten van het wettelijk rookverbod in de horeca

Het wettelijk rookverbod in de horeca, zoals minister Klink dat per 1 juli 2007 heeft ingevoerd, kan de 2e zeef van het beoordelingsmodel passeren, met uitzondering van:

- het rookverbod in kleine cafés zonder personeel (zie het eindoordeel onder het 10e criterium 'rechtvaardigheid');
- de invoeringsdatum 1 juli 2008 (zie het eindoordeel onder het 10e criterium 'rechtvaardigheid').

Deze uitzonderingen acht ik zwaarwegend genoeg om te concluderen dat het wettelijk rookverbod in de horeca, zoals minister Klink dat per 1 juli 2007 heeft ingevoerd, de 2e zeef van het beoordelingsmodel niet kan passeren. Het huidige wettelijk rookverbod in de horeca, waarvan kleine cafés zonder personeel zijn uitgezonderd, kan de 2e zeef van het model wel passeren. Hieronder zal ik dit toelichten.

Het aspect 'hardheid in specifieke gevallen' van het criterium 'rechtvaardigheid' vormt het breekpunt van de gerechtvaardigdheid van het rookverbod in kleine cafés zonder personeel. In verhouding tot de doelstelling van het rookverbod in kleine cafés zonder personeel – het voorkomen van concurrentievervalsing met cafés met personeel – vallen de financiële gevolgen voor deze cafés mogelijk onevenredig en onaanvaardbaar hard uit. Ik schrijf 'mogelijk' omdat een degelijke financiële onderbouwing ontbreekt. Hierbij weeg ik mee dat de concurrentievervalsing in de praktijk wel meevalt

Dat minister Klink de invoeringsdatum van het wettelijk rookverbod voor de hele horeca zonder instemming van KHN heeft vastgesteld op 1 juli 2008 acht ik ongerechtvaardigd, omdat hij hiermee een afspraak tussen de overheid en KHN over de termijn van zelfregulering heeft geschonden. Het eenzijdig opzeggen van de afspraak over de termijn van zelfregulering was niet noodzakelijk, waardoor er sprake lijkt van een onbetrouwbare overheid, wat een ernstige zaak is.

Men kan het voorstel van minister Schippers uit november 2010, om kleine cafés zonder personeel vrij te stellen van het rookverbod in de horeca, zien als een reparatie van de onrechtvaardigheid dat het rookverbod wordt toegepast op kleine cafés zonder personeel, zonder dat er voldoende bekend is over de (vermeende) negatieve economische effecten. Discutabel is dat minister Schippers niet heeft besloten de economische effecten van het rookverbod op kleine cafés diepgaander te onderzoeken. In plaats daarvan heeft zij, ook op basis van onvoldoende geobjectiveerde informatie over de economische effecten voor kleine cafés zonder personeel, besloten om kleine cafés zonder personeel van het rookverbod uit te zonderen. Dit besluit is echter niet evident onrechtvaardig, omdat het concurrentievoordeel voor kleine cafés zonder personeel gering is.

Bij haar besluit om kleine cafés zonder personeel uit te zonderen van het rookverbod in de horeca, lijkt minister Schippers te weinig rekening te hebben gehouden met de gevolgen van haar besluit voor de handhaafbaarheid van het wettelijk rookverbod in cafés met personeel. De ***handhaving*** is hierdoor mogelijk ***suboptimaal*** geworden. In hoeverre de handhaving optimaal of suboptimaal is geregeld, wordt pas beoordeeld in de 3e zeef van het beoordelingsmodel:

- in de ***1e zeef*** van het model wordt aan de hand van het criterium 'geschiktheid' beoordeeld of de partijen die verantwoordelijk zijn voor de ***handhaving*** van de preventiemaatregel daarvoor geschikt zijn;
- in de ***2e zeef*** van het model wordt beoordeeld of de ***handhaving***, vanuit het perspectief van de criteria 'effectiviteit, indringendheid, kosten-batenverhouding en rechtvaardigheid' voldoende is.

– in de ***3e zeef*** van het model wordt beoordeeld in hoeverre de ***handhaving*** optimaal of suboptimaal is geregeld. Hierbij wordt de handhaving beoordeeld in relatie tot de criteria: timing, draagvlak, flankerend beleid, controleerbaarheid, en uitvoerbaarheid.

12.5.2 Gevolgen van de beoordeling voor het model (2e zeef: effecten en neveneffecten)

Precisering van operationalisering van effectiviteit
Naar aanleiding van de beoordeling van het rookverbod aan de hand van het criterium 'effectiviteit' heb ik de definitie van dit criterium aangepast. De nieuwe definitie luidt (zie §12.1.4):

> *"De effectiviteit betreft de beoogde effecten van de preventiemaatregel op de doelgroep of op derden. De doelgroep is hierbij gedefinieerd als de groep personen waarvan men de leefstijl tracht te beïnvloeden ter voorkoming van leefstijlgerelateerde aandoeningen bij de doelgroep zelf of bij derden."*

'Effectiviteit, draagvlak en handhaafbaarheid' in relatie tot naleving
Naar aanleiding van de beoordeling van het rookverbod aan de hand van het criterium 'effectiviteit' heb ik m.b.t. het aspect 'naleving' het volgende onderscheid gemaakt tussen de criteria 'effectiviteit, draagvlak en handhaafbaarheid'. Bij het criterium 'effectiviteit' wordt ingegaan op de mate waarin de preventiemaatregel wordt nageleefd. Bij de criteria 'draagvlak' en 'handhaafbaarheid' wordt ingegaan op de achterliggende oorzaken van de mate waarin de preventiemaatregel wordt nageleefd, respectievelijk het draagvlak voor en de handhaving van de preventiemaatregel. (Zie §12.1.4)

Ondergeschikt belang van kosteneffectiviteit
De beoordeling van het rookverbod aan de hand van het criterium 'kosten-batenverhouding' bevestigt het ondergeschikte belang van de kosteneffectiviteit bij de beoordeling van de gerechtvaardigdheid van leefstijlbeïnvloedende interventies ter bestrijding van leefstijlgerelateerde aandoeningen. (Zie §12.3.4)

Aanvulling op operationalisering van rechtvaardigheid
Naar aanleiding van de beoordeling van het rookverbod aan de hand van het criterium 'rechtvaardigheid' heb ik het ***aspect 'legitimiteit'*** aan het criterium 'rechtvaardigheid' toegevoegd. De legitimiteit van de preventiemaatregel betreft de vraag of de preventiemaatregel niet strijdig is met het vigerende recht (wet- en regelge-

ving, nationale en internationale verdragen, rechtsgeldige overeenkomsten, e.d.). (Zie §12.4.1)

Mogelijke rol voor procedurele rechtvaardigheid

Tijdens de beoordeling van de 'legitimiteit' van het rookverbod ben ik tot de volgende conclusie gekomen. Bij de beoordeling van de gerechtvaardigdheid van de preventiemaatregel aan de hand van het model kunnen procedurele aspecten worden meegewogen. Afhankelijk van de casus dient per criterium te worden afgewogen in hoeverre procedurele aspecten relevant zijn voor de inhoudelijke beoordeling van de gerechtvaardigdheid van de preventiemaatregel. Op zichzelf valt de beoordeling van de procedurele rechtvaardigheid nog steeds buiten het kader van het model. (Zie §12.4.4)

Conclusies over juistheid, volledigheid en praktische toepasbaarheid

Met inachtneming van het bovenstaande kunnen de volgende conclusies worden getrokken. De beoordeling van het wettelijk rookverbod in de horeca aan de hand van de 2e zeef van het model leidt niet tot aanwijzingen dat de criteria van de 2e zeef van het model onjuist, onvolledig of praktisch niet toepasbaar zouden zijn. Het blijkt mogelijk om op basis van de criteria van de 2e zeef van het model tot een beoordeling van de gerechtvaardigdheid van de 'effecten en neveneffecten' van het wettelijk rookverbod in de horeca te komen.

13 Toetsing 3e zeef van het beoordelingsmodel aan casus ‘wettelijk rookverbod in horeca’ (versie 8 van het model)

In dit hoofdstuk toets ik de gerechtvaardigdheid van het wettelijk rookverbod in de horeca aan de hand van de criteria van de 3e zeef van het beoordelingsmodel (versie 8 van het model).

13.1 Elfde criterium: timing

13.1.1 Operationalisering en wijze van beoordeling

Het criterium ‘timing van de implementatie’ betreft de volgende vragen:

- In hoeverre is de tijd rijp om de beoogde leefstijlverandering na te streven door het toepassen van drang of dwang?
- In hoeverre zijn mensen en organisaties die door de preventiemaatregel worden geraakt geleidelijk op de preventiemaatregel voorbereid?
- In hoeverre zijn de actuele politieke, economische en maatschappelijke omstandigheden geschikt voor implementatie?
- In hoeverre krijgen mensen en organisaties die door de preventiemaatregel worden geraakt de tijd om zich aan de nieuwe regels aan te passen?

Beoordeeld wordt of de timing van de implementatie niet onredelijk (onlogisch, irrationeel) of onbillijk (onrechtvaardig) is.

13.1.2 *Beoordeling van casus 'wettelijk rookverbod in horeca' aan de hand van criterium 'timing'*

In hoeverre is de tijd rijp om de beoogde leefstijlverandering na te streven door het toepassen van drang of dwang?

In 1984 sturen staatssecretarissen Van der Reijden van WVC en Van Zeil van EZ het wetsvoorstel voor de Tabakswet naar de Tweede Kamer. De memorie van toelichting stelt[1]:

> *"Hoe zwaar ook de belangen van volksgezondheid moeten wegen, het roken kan niet op stel en sprong uit onze maatschappij worden verbannen. Het nastreven daarvan op langere termijn zal ook grote moeilijkheden ondervinden. Feitelijk zal daarvoor op mondiaal niveau een grote mate van wilsovereenstemming moeten bestaan. De regering kiest daarom voor een beleid op langere termijn, waarvan de beperking van het tabaksgebruik en, in het bijzonder, de bescherming van de jeugd en van de niet-roker de hoofddoelstellingen zijn."* (p.7-8)

In mei 2000 schrijft minister Borst-Eilers aan de Tweede Kamer in reactie op vragen over haar Wetsvoorstel tot wijziging van de Tabakswet[2]:

> *"De Tabakswet van 10 maart 1989, die al enkele jaren eerder door de regering was opgesteld, ging er nog vanuit dat het roken in onze samenleving diep geworteld en sociaal geaccepteerd is. Toen al maakte de regering daarbij de kanttekening dat er tekenen waren dat de maatschappelijke acceptatie van roken aan het verminderen was. De regering beseft dat rookverboden de vrijheid van rokers tijdelijk enigszins beperken; de vrijheid van mensen (rokers) eindigt nu eenmaal daar waar door hun (rook)gedrag de vrijheid van anderen (niet-rokers) wordt belemmerd. Hierbij wil de regering duidelijk – en meer dan tot nu toe – voorrang geven aan niet-rokers."* (p.31)

> *"Tegelijkertijd moeten we met z'n allen ook realistisch blijven. In absolute zin kan de stelling van het Astma Fonds nu eenmaal niet direct letterlijk worden waargemaakt. De daaraan verbonden consequentie zou namelijk zijn dat er bijna overal een rookverbod zou moeten komen, of anders gesteld: dat een roker nooit meer kan roken als er een niet-roker in de buurt is. Dit is als streven een prima doel, dat ik ook van harte onderschrijf, maar het is natuurlijk niet van de ene op de andere dag haalbaar noch te verwezenlijken."* (p.35)

> *"De niet-rokers zijn zich geleidelijk aan bewuster geworden van de schadelijke effecten die tabaksrook veroorzaakt, vandaar dat zij steeds vaker en in grotere getale hun recht op schone lucht opeisen. Dat speelt in het bijzonder op de werkplek, want daar*

1. TK, 1984-1985, 18749, nr.3.
2. TK, 1999–2000, 26472, nr.6.

moet de niet rokende werknemer verplicht vele uren per dag doorbrengen in de rook van collega's." (p.36)

In mei 2001 stelt Tweede Kamerlid Oudkerk in een vergadering van de Tweede Kamer over het Wetvoorstel tot wijziging van de Tabakswet[3]:

"Nederland wordt altijd gezien als gidsland, maar zowel binnen Europa als wereldwijd loopt Nederland helemaal niet voorop als het gaat om het nemen van maatregelen om het roken te beperken. Wij moeten daarbij overigens ook naar onszelf kijken. In 1995 en 1996 hebben wij met het kabinet over de Tabaksnota gesproken en pas vijf jaar later zijn alle zaken waar de Kamer toen akkoord mee is gegaan, rijp voor wetgeving." (p.5176)

In diezelfde vergadering in mei 2001 stelt Tweede Kamerlid Kant:

"In datzelfde jaar [1975] was er een rapport van de Gezondheidsraad, die in 1975 een aantal maatregelen voorstelde die ons vandaag misschien bekend voorkomen: rookverboden in openbare gebouwen, het drastisch beperken van het aantal verkooppunten, het invoeren van een algemeen reclameverbod en het beschermen van de nietroker op zijn werkplek. … De vraag is hoe het mogelijk is dat dit 25 jaar heeft geduurd." (p.5192)

In december 2001 schrijft minister Borst-Eilers over haar Wetsvoorstel tot wijziging van de Tabakswet aan de Eerste Kamer[4]:

"Naar aanleiding van een brief van Koninklijke Horeca Nederland vragen de leden van de fractie van GroenLinks of het recht van werknemers op een rookvrije werkplek automatisch een rookvrije horeca betekent. De regering is niet op voorhand tegen het in eerste instantie tijdelijk uitzonderen van een enkele sector op het komende recht op een rookvrije werkplek. Een consequent doorgevoerd recht van werknemers op een rookvrije werkplek impliceert logischerwijs uiteindelijk inderdaad een rookvrije horeca (met bijvoorbeeld speciale rookruimten). Het mag echter duidelijk zijn dat werkgevers in de horeca, als ze goede argumenten aanvoeren, in aanmerking komen om voorlopig te worden uitgezonderd van de in artikel 11a, eerste lid, vastgelegde verplichting. Een dergelijke uitzondering zal echter altijd aan een duidelijke tijdslimiet gebonden zijn en zal werkgevers in de horeca nooit van de verplichting ontslaan om op basis van zelfregulering een verandering ten goede te bewerkstelligen." (p.15)

3. TK, 2000-2001, vergaderingnr.82, p.5175-5208 (dossiernr.26472).
4. EK, 2001–2002, 26472, nr.59a.

In juli 2007 overlegt de vaste commissie voor VWS van de Tweede Kamer met minister Klink over zijn besluit tot invoering van het rookverbod in de horeca per 1 juli 2008[5]:

"Mevrouw Joldersma (CDA) meent dat de minister de tijdgeest goed aanvoelt door te kiezen voor een rookvrije horeca per 1 juli 2008. Het maatschappelijk draagvlak is toegenomen. Dat is niet in de laatste plaats te danken aan de horeca zelf. Het stappenplan heeft bijgedragen tot steeds meer maatregelen om de werknemer en de niet-roker te beschermen.

"Minister Klink: … Veel landen zijn Nederland voorgegaan in het instellen van een rookverbod. (p.7)

In hoeverre is de doelgroep van de preventiemaatregel geleidelijk op de gevraagde gedragsverandering voorbereid?

In juli 1999 stellen leden van de PvdA-fractie in een overleg van de vaste commissie voor VWS van de Tweede Kamer over het Wetsvoorstel tot Wijziging van de Tabakswet[6]:

"De leden van de fractie van de PvdA constateren dat de horeca nog steeds een ontwikkelingsgebied is wat betreft rookvrije restaurants en cafés. Er zijn slechts weinig restaurants en nauwelijks cafés die een rookvrij gedeelte hebben. Deze leden betreuren dat ten zeerste, en vragen de regering beleid te ontwikkelen dat hier op een creatieve manier op inspeelt." (p.21)

In mei 2000 schrijft minister Borst-Eilers aan de Tweede Kamer in reactie op vragen over haar Wetsvoorstel tot wijziging van de Tabakswet[7]:

"De onvoldoende maatschappelijke acceptatie van het recht op schone lucht voor niet-rokers en de in te geringe mate aanwezige tolerantie bij rokers blijkt in de dagelijkse praktijk niet voldoende ruimte te bieden voor een daadwerkelijk volwaardige bescherming van de meerderheid van niet-rokers in ons land. Daarom is het onvermijdelijk om wettelijke regels te gaan stellen voor die plaatsen waar een ieder vrij moeten kunnen komen of vertoeven zonder hinder van andermans rook. De regering geeft inhoud aan voornoemd uitgangspunt door geleidelijk aan steeds meer plaatsen, ruimten en tijdstippen aan te wijzen waar niet meer gerookt kan worden. De rookverboden zullen verder moeten worden uitgebreid en aangescherpt." (p.35)

5. TK, 2006–2007, 30800 XVI, nr.182.
6. TK, 1998–1999, 26472, nr.5.
7. TK, 1999–2000, 26472, nr.6.

In december 2001 schrijft minister Borst-Eilers over haar Wetsvoorstel tot wijziging van de Tabakswet aan de Eerste Kamer[8]:

> *"De leden van de PvdA-fractie gaan in op de inwerkingtredingstermijnen van de voorgestelde wettelijke bepalingen. De mate van succes van het wetsvoorstel wordt naast de handhaafbaarheid inderdaad ook bepaald door het draagvlak in de samenleving. Het verdient overweging bepaalde sectoren van de samenleving nog enige tijd te gunnen om via zelfregulering alvast vooruit te lopen op de gewenste en beoogde situatie: een rookvrije omgeving op het werk, in het openbaar vervoer en in de voor publiek toegankelijke gebouwen. Als dat lukt, dan vindt nadien via het in werking laten treden van artikel 11a codificatie van een al gegroeide sociale norm in de Tabakswet plaats. Zo'n scenario heeft veruit mijn voorkeur."* (p.2)

In september 2004 schrijft minister Hoogervorst in een brief aan de Tweede Kamer over zijn afspraken met KHN over het zelfreguleringtraject in het kader van een rookvrije horeca[9]:

> *"Als gevolg van de maatschappelijke ontwikkelingen in ons land en de landen om ons heen zal de horeca op termijn rookvrij worden. Er is dus momenteel sprake van een overgangssituatie. Tegen deze achtergrond ben ik met KHN de stappen overeengekomen … Enerzijds krijgen werknemers in de horeca – bij goede uitvoering van onderstaande stappen – op korte termijn een betere bescherming tegen tabaksrook dan in de huidige situatie. Anderzijds stellen deze stappen werkgevers, werknemers en horecabezoekers in de gelegenheid om te wennen aan een rookbeleid in de horeca waardoor de stap naar een rookvrije horeca op termijn makkelijker gezet kan worden."* (p.2)

> *"Bij rookvrije zones is het belangrijk om te beseffen dat deze, in tegenstelling tot rookvrije sectoren, geen garantie bieden ten aanzien van bescherming van de gezondheid van de werknemers. In die zin moeten rookvrije zones niet beschouwd worden als de eindsituatie maar veel meer als een overgangsfase waarin bewustwording van werkgever, werknemer en gast zal toenemen en roken in de horeca steeds minder vanzelfsprekend zal worden."* (p.3)

In hoeverre zijn de actuele politieke, economische en maatschappelijke omstandigheden geschikt voor implementatie?

In april 2005 schrijft minister Hoogervorst in een brief aan de Tweede Kamer over het stappenplan dat hij met KHN overeen is gekomen[10]:

8. EK, 2001–2002, 26472, nr.59a, p.2.
9. TK, 2003–2004, 29200 XVI, nr.278.
10. TK, 2004–2005, 29800 XVI, nr.162.

"Ik ben met Koninklijk Horeca Nederland (KHN) overeengekomen dat de monitoring van het stappenplan gezamenlijk zal worden uitgevoerd. Daartoe zal gezamenlijk een onafhankelijk onderzoeksbureau worden geselecteerd dat in overleg met KHN en VWS de onderzoeksopzet en onderzoeksmethode verder zal vormgeven." (p.1)

"Het stappenplan beslaat een periode van vier jaar (2005, 2006, 2007 en 2008) en bevat voor elk jaar concrete streefcijfers. Na afloop van elk jaar zal de voortgang van de invoering van de afgesproken maatregelen worden gemeten. Er zal dus over elk jaar een aparte voortgangsrapportage verschijnen waarin wordt teruggekeken op het voorgaande jaar of de voorgaande jaren. Aan het eind van de totale periode zal een eindevaluatie worden opgesteld." (p.1)

"De planning ziet er nu als volgt uit.
- *Eerste meting (over 2005) in het eerste kwartaal van 2006; de rapportage daarover zal medio 2006 verschijnen; …;*
- *Vierde meting (over 2008) in het eerste kwartaal van 2009; de rapportage daarover en de eindevaluatie (over de gehele periode 2005–2008) zal medio 2009 verschijnen."* (p.1-2)

Op basis van deze planning kan pas eind 2009 worden besloten hoe het invoeringstraject om te komen tot een rookvrije horeca verder vorm zal worden gegeven. Toen minister Klink in juni 2007 zijn besluit tot invoering van de rookvrije horeca per 1 juli 2008 kenbaar maakte, was dat ***twee jaar voor de afgesproken eindevaluatie medio 2009.*** Dit zou een omstandigheid kunnen zijn die vraagt om uitstel van implementatie. Bijvoorbeeld door het rookverbod in aansluiting op de afgesproken eindevaluatie per 1 januari 2010 in te laten gaan.

In juni 2007 schrijft Minister Klink een brief aan de Tweede Kamer, over zijn besluit tot invoering van een rookvrije horeca zonder uitzonderingen[11]:

"Resultaten tweede jaar zelfregulering (2006). … De kwantitatieve doelen zijn – behoudens in enkele sectoren – gehaald, maar de resultaten zijn in kwalitatieve zin minder positief. Zeker de geringe omvang van de rookvrije delen en de – in bepaalde sectoren – weinig consequente toepassing maken duidelijk dat de rookvrije horeca en bescherming van de werknemer via zelfregulering niet bereikt zullen worden." (p.3)

"In het coalitieakkoord van 7 februari 2007 staat dat «in samenspraak met de branche zal worden toegewerkt naar een rookvrije horeca in deze kabinetsperiode». Ik heb hierover gesproken met Koninklijk horeca Nederland (KHN), … KHN heeft toen, tegen de achtergrond van de passages uit het coalitieakkoord betreffende de rookvrije horeca, aangegeven dat de tijd rijp is voor een andere benadering en heeft

11. TK, 2006-2007, 30800 XVI, nr.149.

voorgesteld om de sectoren hotels, restaurants en fastfood per 1 januari 2008 rookvrij te maken. Voor de sectoren cafés en discotheken wil KHN een apart traject dat op termijn, maar niet eerder dan 1 januari 2011, ook leidt tot een rookvrije situatie." (p.3)

"Ik heb besloten om niet mee te gaan in het voorstel van KHN omdat:
1. *werknemers (secundair: bezoekers) in de horeca beschermd worden tegen tabaksrook (ongelijke behandeling) en juist de werknemers die de hoogste blootstelling aan tabaksrook hebben (cafés, discotheken), worden uitgezonderd;*
2. *in sommige gevallen er op papier wel maar in de praktijk eigenlijk geen bescherming is (alleen rookvrij als de keuken geopend is);*
3. *niet alle horecasectoren op dezelfde manier behandeld worden (ongelijk speelveld);*
4. *de kans daarom groot is dat deze oneerlijke concurrentiepositie juridisch aangevochten zal worden;*
5. *andere sectoren onmiddellijk aansluiting zullen zoeken bij cafés en discotheken (in ieder geval podia, sport, zorginstellingen, buurtcafés);*
6. *het onderscheid in de praktijk moeilijk hanteerbaar zal zijn en zal leiden tot «ontwijkgedrag» door ondernemers: wat is zitten?, roken voor en na sluitingstijd keuken, vallen barmaaltijden onder «zittend eten»?,enzovoorts;*
7. *het ingewikkelde onderscheid en de vele grensgevallen de handhaving bemoeilijken en aanleiding zullen zijn voor het ter discussie stellen van het optreden van de VWA en het aanvechten van opgelegde boetes;*
8. *de ervaringen in bijvoorbeeld België leren dat een onderscheid tussen subsectoren ingewikkeld is1 en in de praktijk veel onduidelijkheid geeft bij horecaondernemers, de handhavende instantie en het publiek."* (p.3-4)

In juli 2007 overlegt de vaste commissie voor VWS van de Tweede kamer met minister Klink over zijn besluit tot invoering van het rookverbod in de horeca per 1 juli 2008[12]:

"Mevrouw Schippers (VVD): … In het regeerakkoord staat dat in samenspraak met de branche wordt gewerkt aan een rookvrije horeca. De minister kondigt echter eenzijdig aan het rookverbod 2,5 jaar eerder te laten ingaan. Dat heeft geleid tot veel ongenoegen. Waaruit bestaat die samenspraak dan eigenlijk?" (p.3-4)

"Mevrouw Joldersma (CDA) … De afspraak rond rookvrije horeca was dat een en ander in samenspraak met Koninklijke Horeca Nederland zou plaatsvinden. Koninklijke Horeca Nederland spreekt echter van een onbetrouwbare overheid. Hoe heeft die samenspraak dan plaatsgevonden?" (p.4)

12. TK, 2006–2007, 30800 XVI, nr.182.

*"Minister Klink: … Er is gesproken met de horeca, de vakbonden, de werknemers en NOC*NSF. Hun argumenten zijn serieus genomen. De invoering is met een halfjaar uitgesteld. Fasering bleek echter buitengewoon lastig en daarom is besloten tot een verbod voor iedereen per 1 juli 2008."* (p.7-8)

In hoeverre krijgt de doelgroep de tijd om zich aan de nieuwe regels aan te passen?

In maart 2002 stelt minister Borst-Eilers bij de behandeling van het Wetsvoorstel tot wijziging van de Tabakswet in de Eerste Kamer[13]:

"Bij het invoeren van zo'n rookbeleid in een onderneming is het draagvlak onder het personeel een belangrijke factor. Als je dat van de ene op de andere dag afkondigt zonder dat je eerst een draagvlak hebt gecreëerd, dan zal het succes niet erg groot zijn. Ook daar geldt dus het principe: eerst veel overleg en dan pas daadwerkelijk invoering." (p.1260)

In september 2003 schrijft Minister Hoogervorst in een brief over het 'zelfreguleringstraject in het kader van een rookvrije horeca' aan de Tweede Kamer[14]:

"Ik heb reeds eerder benadrukt dat de horeca op termijn rookvrij zal zijn. Ik zie mij daarin bevestigd door de ontwikkelingen in andere Europese landen, zoals Ierland, Noorwegen en Zweden. Er is dus sprake van een overgangssituatie waarbinnen ik de horeca de gelegenheid wil geven om zelf bepaalde stappen te zetten. Ik ben in overleg met KHN over hun stappenplan om te bezien in hoeverre binnen dit kader overeenstemming over zelfregulering bereikt kan worden." (p.2)

In juli 2007 stelt minister Klink in een algemeen overleg met de Tweede Kamer over zijn besluit tot invoering van een rookvrije horeca[15]:

"Per 1 juli 2008 geldt een rookverbod voor de hele sector. Dat biedt duidelijkheid voor iedereen. Er is geen sprake van fasering voor de natte horeca en de restaurants en er zijn geen definitie- en afbakeningskwesties meer. De horeca heeft nog een jaar de tijd om te wennen, zich voor te bereiden en eventueel vergunningen voor verbouwingen aan te vragen. Omdat een en ander tijd vraagt, is niet gekozen voor invoering per 1 januari 2008. Bovendien biedt invoering in de zomer de bezoeker meer gelegenheid om eraan te wennen. Hij kan immers naar buiten lopen om te roken of eventueel op een terras roken. Er komt een invoeringscampagne, gericht op werkgevers, werknemers en het publiek." (p.7)

13. EK, 2001-2002, vergaderingnr.24, p.1236-1275 (dossiernr.26472).
14. TK, 2003–2004, 29200 XVI, nr.233.
15. TK, 2006–2007, 30800 XVI, nr.182.

In december 2008 schrijft minister Klink in een brief aan de Tweede Kamer over de praktische implicaties van de invoering van de rookvrije horeca[16]:

> *"Ik heb er, in goed overleg met de betrokken sectoren, voor gekozen om direct vanaf het begin te handhaven maar in eerste instantie alleen waarschuwingen uit te delen. Dit heb ik niet alleen besloten om het onterechte beeld weg te nemen dat de Voedsel en Waren Autoriteit (VWA) bij iedere brandende sigaret direct een boete zou opleggen, maar ook omdat ik vind dat als iemand het rookverbod overtreedt hij of zij in de gelegenheid moet worden gesteld om de overtreding op te heffen. … Niettemin kon de VWA boetes opleggen als bij hercontrole zou blijken dat er nog steeds sprake was van een overtreding."* (p.2-3)

Analyse van de timing van de implementatie van het rookverbod

Uit de analyse van de citaten over timing van de implementatie blijkt dat politici veel oog voor deze timing hebben. In het algemeen kiezen de achtereenvolgende ministers van VWS bij de implementatie van rookverboden voor de weg van de geleidelijkheid. Daardoor kunnen betrokken partijen aan de rookverboden wennen en zich erop instellen.

Minister Borst-Eilers heeft ervoor gekozen om de horeca tijdelijk uit te zonderen van het wettelijk recht op een rookvrije werkplek, omdat de tijd nog niet rijp was voor implementatie van dit recht in de horeca. Minister Hoogervorst is in het kader van de zelfregulering van het rookbeleid in de horeca met KHN een stappenplan overeengekomen. Onderdeel van dit stappenplan was dat een steeds groter deel van de horeca rookvrij zou worden. Minister Klink heeft in juni 2007 zijn besluit kenbaar gemaakt om de horeca een jaar tijd te geven om zich op de invoering van het rookverbod in de horeca per 1 juli 2008 voor te bereiden. Daarbij heeft hij er ook rekening mee gehouden dat invoering in de zomer minder problemen geeft dan invoering in de winter, omdat horecabezoekers in de zomer makkelijker buiten kunnen roken.

Minister Klink is echter ook de eerste minister die van de weg van de geleidelijkheid is afgeweken. Het stappenplan om toe te groeien naar een rookvrije horeca, dat minister Hoogervorst met KHN was overeengekomen, betrof de periode 2005 tot en met 2008. In dit plan waren voornamelijk kwantitatieve doelstellingen afgesproken. Ondanks dat de horeca deze kwantitatieve doelstellingen in 2007 nagenoeg volledig had gerealiseerd, heeft minister Klink deze overeenkomst tussen de overheid en KHN eenzijdig opgezegd. KHN heeft toen voorgesteld om het wette-

16. TK, 2008–2009, 22894, nr.206.

lijk rookverbod in de horeca, waartoe in het regeerakkoord was besloten, gefaseerd in te voeren, waarbij cafés en discotheken in 2011 als laatste aan de beurt zouden komen. Minister Klink heeft dit voorstel van KHN tot fasering naast zich neergelegd en besloten om het wettelijk rookverbod voor alle horeca tegelijk in te voeren.

In zijn brief[17] aan de Tweede Kamer van 8 juni 2007 geeft minister Klink maar liefst acht redenen waarom hij niet op de fasering van de implementatie van het rookverbod, zoals voorgesteld door KHN, ingaat. Naar mijn oordeel zijn deze redenen overtuigend. Feit blijft echter dat minister Klink eerst eenzijdig de overeenkomst (het stappenplan) met KHN heeft opgezegd en vervolgens de door KHN voorgestelde fasering naast zich neer heeft gelegd. Deze gang van zaken heeft wrevel opgeroepen bij ondermeer KHN. Terugkijkend zou men kunnen concluderen dat dit misschien wel heeft geleid tot een vertrouwensbreuk tussen de horeca en de overheid op het gebied van het rookbeleid in de horeca. Mogelijk heeft deze gang van zaken minister Klink ernstig parten gespeeld toen de handhaving van het rookverbod in kleine cafés zonder personeel een probleem begon te worden. In ieder geval kan worden geconstateerd dat KHN toen niet naar voren is getreden om minister Klink te helpen om de naleving van het rookverbod in deze cafés te verbeteren. M.i. heeft minister Klink een procedurefout gemaakt, door de afspraken met KHN eenzijdig open te breken.

13.1.3 Eindoordeel (criterium timing)

Met uitzondering van de invoeringsdatum van het wettelijk rookverbod in de horeca kan de wijze waarop de overheid is omgegaan met de invoering van (wettelijke) rookverboden redelijk en billijk worden genoemd. De invoeringsdatum van het wettelijk rookverbod in de horeca was redelijk (logisch, rationeel), maar deels onbillijk (onrechtvaardig). (Zie voor een toelichting op de onbillijkheid ook onder het aspect ‘legitimiteit’ in §12.4.2.)

Toen minister Klink in juni 2007 zijn besluit tot invoering van de rookvrije horeca per 1 januari 2008 kenbaar maakte, was de tijd daarvoor rijp. De horeca kreeg ruim voldoende tijd (een jaar) om zich op de implementatie voor te bereiden (er was immers al een stappenplan). Zo bezien was de timing van de implementatie redelijk en billijk. Toch voelde KHN zich overvallen door het besluit. De reden hiervan was voornamelijk dat minister Klink een procedurele c.q. bestuurlijke fout heeft

17. TK, 30800 XVI, nr.149.

gemaakt door eenzijdig de overeenkomst (het stappenplan) met KHN open te breken en de verantwoordelijkheid naar zich toe te trekken.

13.1.4 Gevolgen van de beoordeling voor het model (criterium timing)

In §12.4.4 heb ik geconcludeerd dat per criterium van het model beoordeeld dient te worden of procedurele aspecten relevant zijn voor de beoordeling van de preventiemaatregel aan de hand van het betreffende criterium. De invoeringsdatum (procedureel aspect) blijkt van belang te (kunnen) zijn voor de beoordeling van de preventiemaatregel aan de hand van het criterium 'timing' van de implementatie.

13.2 Twaalfde criterium: draagvlak

13.2.1 Operationalisering en wijze van beoordeling

Het criterium 'draagvlak' betreft de volgende vragen:
- In hoeverre en op welke manier(en) is er aandacht besteed aan het verwerven van draagvlak voor de preventiemaatregel?
- Hoeveel draagvlak is er voor de preventiemaatregel?

Het gaat hierbij om draagvlak bij alle betrokken partijen en in ieder geval bij de:
a. doelgroep waarop de preventiemaatregel zich richt;
b. belanghebbenden die door de preventiemaatregel worden beschermd tegen schade, hinder of overlast;
c. partijen die de preventiemaatregel uitvoeren en/of handhaven;
d. partijen die voor- of nadeel ondervinden van de preventiemaatregel.

Er zit overlap tussen deze partijen. Voor het model is deze overlap geen bezwaar. Het gaat erom dat bij het beoordelen van het draagvlak aan alle voor het draagvlak relevante partijen wordt gedacht. Indien relevant gaat het ook om maatschappelijk draagvlak (draagvlak onder de bevolking) en politiek draagvlak.

Ter illustratie geef ik voorbeelden van partijen die in theorie voor- of nadeel van een wettelijk rookverbod in de horeca kunnen ondervinden (en niet tot de partijen a, b of c behoren):
- voordeel:
  - leveranciers van luchtzuiveringsinstallaties (als deze zouden worden toegestaan);
- nadeel:

– mensen die in de buurt van een café wonen en geluidsoverlast ondervinden van buiten rokende bezoekers;
– producenten of leveranciers van tabaksproducten die hun verkoop zien afnemen.

Beoordeeld wordt of er voldoende draagvlak bestaat bij de betrokken partijen voor toepassing van de preventiemaatregel, zodat de handhaving en uitvoering niet onnodig moeilijk en/of onevenredig kostbaar worden. Vanuit dit perspectief wordt tevens beoordeeld of er voldoende aandacht is en wordt besteed aan het verwerven van draagvlak voor de preventiemaatregel en of het draagvlak niet onnodig is of wordt ondermijnd.

13.2.2 Beoordeling van casus 'wettelijk rookverbod in horeca' aan de hand van criterium 'draagvlak'

Ik begin de beoordeling met het geven van een overzicht van citaten die te maken hebben met het criterium draagvlak. Vervolgens zal ik (mede) aan de hand van deze citaten trachten de vragen behorend bij het criterium draagvlak te beoordelen.

In 1996 vond een algemeen overleg plaats tussen de vaste commissie voor VWS van de Tweede Kamer en de ministers Borst-Eilers van VWS en Wijers van EZ over het tabaksontmoedigingsbeleid[18]:

> *"Mevrouw Oedayraj Singh Varma (GroenLinks): ...Toch hield zij vast aan haar voorkeur voor een reclameverbod, want reclame heeft altijd invloed op jongeren en niet-rokers, terwijl juist niet-roken de norm zou moeten zijn en roken abnormaal gevonden zou moeten worden."* (p.6)

> *"De heer Marijnissen (SP) was in grote lijnen blij met de brief van 28 mei. Ook hij vond dat speerpunt van het beleid moet zijn dat zo min mogelijk mensen met roken beginnen en dat roken steeds meer als «abnormaal gedrag» wordt gezien."* (p.14)

> *"De heer Marijnissen (SP): ... Al met al onderschreef hij de in de brief genoemde maatregelen, ook die waar vraagtekens kunnen worden gezet bij de praktische haalbaarheid. Hij deed dat vooral met het oog op de passage op blz. 6 in de brief, waarin staat dat door dit soort maatregelen een sociaal klimaat kan ontstaan waarin niet-roken de norm is en het niet beginnen of stoppen met roken het gevolg kan zijn."* (p.15)

18. TK, 1996–1997, 24743, nr.3.

In 2001 schrijft minister Borst-Eilers in reactie op opmerkingen en vragen van Eerste Kamerleden over haar wetvoorstel tot wijziging van de Tabakswet in een memorie van antwoord[19]:

> *"De regering heeft met belangstelling kennis genomen van de opmerkingen en vragen van de leden van de vaste commissie voor Volksgezondheid, Welzijn en Sport. … De brede steun voor het tabaksontmoedigingsbeleid doet mij goed."* (p.1)

> *"De regering heeft geen bericht ontvangen van de Europese Commissie in het kader van de notificatie-procedure. Dit betekent dat er geen bezwaren kenbaar zijn gemaakt."* (p.2)

> *"De leden van de PvdA-fractie gaan in op de inwerkingtredingstermijnen van de voorgestelde wettelijke bepalingen. De mate van succes van het wetsvoorstel wordt naast de handhaafbaarheid inderdaad ook bepaald door het draagvlak in de samenleving. Het verdient overweging bepaalde sectoren van de samenleving nog enige tijd te gunnen om via zelfregulering alvast vooruit te lopen op de gewenste en beoogde situatie: een rookvrije omgeving op het werk, in het openbaar vervoer en in de voor publiek toegankelijke gebouwen. Als dat lukt, dan vindt nadien via het in werking laten treden van artikel 11a codificatie van een al gegroeide sociale norm in de Tabakswet plaats. Zo'n scenario heeft veruit mijn voorkeur."* (p.2)

> *"Wat betreft het draagvlak voor een wettelijk vastgelegd recht op een rookvrije werkplek merk ik het volgende op: de werkgeversorganisaties VNO-NCW en MKB blijven gekant tegen een wettelijke regeling van het recht op een rookvrije werkplek. In een brief d.d. 28 augustus jl. aan uw Kamer hebben zij dat nog eens bevestigd. De Stichting van de Arbeid is dezelfde mening toegedaan. Wanneer men echter kijkt naar het draagvlak onder de bevolking, dan komt een ander beeld naar voren: volgens een vrij recente NIPO-enquête vindt circa 65% van de bevolking dat er op de werkplek een wettelijk rookverbod moet worden ingesteld (overigens in combinatie met speciale rookruimten)."* (p.16)

In februari 2002 schrijft minister Borst-Eilers in een nadere memorie van antwoord (zie de memorie van antwoord hierboven)[20]:

> *"Daarnaast komen er ook steeds meer volwassen mensen die er prijs op stellen om uit eten te kunnen gaan in een rookvrij restaurant, of in elk geval zonder al te veel hinder of overlast van tabaksrook te ondervinden. Willen we dat (uiteindelijk) wettelijk mogelijk maken of laten we het (vooralsnog) over aan de zelfregulering? Al dit soort vragen zullen in de loop der jaren keer op keer opnieuw moeten worden bezien. Immers, het gaat hierbij eerst en vooral ook om draagvlak in de samenleving. Als steeds meer burgers niet langer tabaksrook in willen ademen, dan is er reden om*

19. EK, 2001–2002, 26472, nr.59a.
20. EK, 2001–2002, 26472, nr.59c.

maatregelen tegen hinder en overlast van tabaksrook te nemen, hetzij via zelfregulering, hetzij in de vorm van regelgeving." (p.5)

In maart 2002 stelt Eerste Kamerlid van Heukelum tijdens de behandeling van het wetsvoorstel tot wijziging van de Tabakswet in de Eerste Kamer[21]:

"… Mijn fractie kan dan ook alleszins instemmen met het geformuleerde doel van het wetsvoorstel, te weten [1] het beschermen van de niet-roker. Ook [2] het terugbrengen van roken onder jongeren achten wij een goede zaak. Overigens heerst er over beide doelstellingen naar mijn mening in onze samenleving al een brede consensus." (p.1252)

In juni 2006 schrijft minister Hoogervorst aan de Tweede Kamer over zijn visie op een toekomstige rookvrije horeca[22]:

"Er zijn ook internationale ontwikkelingen die mij bevestigen in mijn eerder ingenomen standpunt dat ook in Nederland de horeca op termijn rookvrij zal zijn. Het Nederlandse tabaksontmoedigingsbeleid is in belangrijke mate internationaal ingekaderd. Zo heeft Nederland begin 2005 het Kaderverdrag inzake tabaksontmoediging van de Wereldgezondheidsorganisatie (WHO FCTC) geratificeerd. Tijdens de eerste Conference of the Parties (COP – februari 2006) ingevolge de WHO FCTC is door ruim 120 landen, waaronder Nederland, besloten om een richtlijn op te stellen ten aanzien van artikel 8 van het verdrag. Dit artikel verplicht Partijen maatregelen te treffen om mensen te beschermen tegen blootstelling aan (omgevings)tabaksrook. Uitgangspunt voor de op te stellen richtlijn is onder meer: «From a public health perspective, no «safe» levels of second-hand smoke exist. And there is conclusive evidence that engineering approaches do not protect against exposure to tobacco smoke.» Daarnaast kiest een aantal landen, wereldwijd maar vooral ook in Europa, voor een wettelijk rookverbod, ook in de horeca, ter bescherming van werknemers tegen omgevingstabaksrook. Dit aantal neemt snel toe. Het gaat hier om cultureel zeer uiteenlopende landen. Recentelijk hebben Engeland en Uruguay (als eerste Zuid-Amerikaans land) gekozen voor het wettelijk rookverbod, ook in de horeca. Al deze landen hebben ventilatie afgewezen als alternatief voor het rookverbod. Bovenstaande (inter)nationale context biedt mijns inziens overtuigend bewijs voor mijn standpunt dat ventilatie niet gezien kan worden als effectieve oplossing om werknemers en bezoekers te beschermen tegen omgevingstabaksrook." (p.2-3)

In oktober 2009 stelt het reviewrapport van Regioplan over het draagvlak onder de Nederlandse bevolking voor het rookverbod in de horeca:

21. EK, 2001-2002, vergaderingnr.24, p.1236-1275 (dossiernr.26472).
22. TK, 2005–2006, 30300 XVI, nr.144.

"De meerderheid van de bevolking [55%] vindt de rookvrije horeca een (zeer) goede zaak. Bijna driekwart is het ook (helemaal) eens met de stelling dat werknemers in de horeca het recht hebben op een rookvrije werkplek." (p.11)

"In 2005 heeft de Europese Commissie onderzoek gedaan naar de attitude van de bevolking ten aanzien van rookvrij horecabeleid. Een meerderheid van de Europeanen was voorstander van rookvrije cafés (61%) en van rookvrije restaurants (77%). Het draagvlak was het grootst in landen waar op dat moment al en rookvrije horeca was ingevoerd. Vergeleken met andere Europese landen was in Nederland het draagvlak erg laag; meer dan de helft van de Nederlandse bevolking was tegen de invoering van een rookverbod in cafés." (p.19)

Het reviewrapport van Regioplan geeft de volgende percentages voor het 1e kwartaal van 2009 (n=32550) (figuur 3.3, pagina 20):

"Mening van de Nederlandse bevolking over de stelling 'Een rookvrije horeca vind ik een …':
- *zeer goede zaak: 29%*
- *goede zaak: 26%*
- *noch slechte, noch goede zaak: 21%*
- *slechte zaak: 14%*
- *zeer slechte zaak: 9%."*

"De houding van horecaondernemers (van discotheken, restaurant en cafés) ten aanzien van het rookverbod is divers. Eind 2008 is meer dan de helft (55%) overwegend tot zeer negatief ten aanzien van het verbod, een kwart is overwegend tot zeer positief en een vijfde staat er neutraal tegenover. In juni 2009 zijn de ondernemers echter iets positiever gestemd. Minder dan de helft heeft een negatieve houding (46%). Net als vorig jaar is de meerderheid van de horecaondernemers van cafés in 2009 negatief over het rookverbod (59%) alsook de discotheekhouders (75%). Van de restaurantondernemers heeft 23 procent een negatieve houding ten aanzien van het rookverbod." (p.21)

"De houding van horecaondernemers (van discotheken, restaurant en cafés) ten aanzien van het rookverbod is divers. Eind 2008 is meer dan de helft (55%) overwegend tot zeer negatief ten aanzien van het verbod, een kwart is overwegend tot zeer positief en een vijfde staat er neutraal tegenover. In juni 2009 zijn de ondernemers echter iets positiever gestemd. Minder dan de helft heeft een negatieve houding (46%). Net als vorig jaar is de meerderheid van de horecaondernemers van cafés in 2009 negatief over het rookverbod (59%) alsook de discotheekhouders (75%). Van de restaurantondernemers heeft 23 procent een negatieve houding ten aanzien van het rookverbod." (p.21)

Op 1 december 2009 schrijft minister Klink in een brief aan de Tweede Kamer over het draagvlak voor het rookverbod in de horeca[23]:

> *"Duidelijk is dat de invoering van de rookvrije horeca vrijwel niemand onberoerd heeft gelaten. De meerderheid van de Nederlandse bevolking vindt de rookvrije horeca een goede zaak. Het draagvlak onder de ondernemers verschuift ook: de stemming is in 2009 positiever dan in 2008. De naleving is in het algemeen hoog, maar kan nog duidelijk verbeterd worden bij de sectoren cafés en discotheken."* (p.4)

In januari 2010 schrijft minister Klink een brief aan de Tweede Kamer over de resultaten van de invoeringscampagne van het rookverbod in de horeca[24]:

> *"In 2008 heeft een invoeringscampagne plaatsgevonden om iedereen zo goed mogelijk te informeren over de rookvrije horeca, kunst/cultuur en sport…. Het percentage onder het algemeen publiek dat positief staat tegenover een rookvrije horeca (51%) is door de campagne niet toegenomen. … Onder de rokers bleef 63% tegen de invoering van een rookvrije horeca."* (p.4)

In januari 2011 overlegt de vaste commissie van de Tweede Kamer voor VWS met minister Schippers over het voorstel van de minister om kleine cafés zonder personeel uit te zonderen van het rookverbod[25]:

> *"Mevrouw Voortman (GroenLinks): … Is er dan minder draagvlak gekomen voor het rookverbod? Nee, het draagvlak is juist toegenomen. Een deel van de mensen dacht eerst: moet dit nu wel? Sinds de invoering van het rookverbod constateren velen dat er toch voordelen zijn. De kleren stinken niet meer en de mogelijkheid om uit te gaan is gegroeid. Het is daarom niet verwonderlijk dat uit onderzoek van TNS NIPO is gebleken dat een meerderheid van de Nederlandse bevolking voor het rookverbod is."* (p.7)

> *"Minister Schippers: … Als er één onderwerp is waarbij de meningen gigantisch gepolariseerd zijn, is het wel bij dit onderwerp. Enkele woordvoerders hebben al gesproken over de mails die zij krijgen. Ik weet daar uit eigen ervaring alles van: de meeste dreigmails gaan over dit onderwerp. Een groep mensen vindt dat heel Nederland rookvrij moet zijn en een groep mensen vindt dat altijd en overal gerookt mag worden. Ik ben op zoek naar een manier om dit te depolariseren en om die enorme felheid uit deze discussie te halen door het zo te regelen dat men weet bij wat voor café men naar binnen gaat en dat er geen werknemers zijn die acht uur in de rook staan. Ik ben op zoek naar een manier om hier een beetje anders mee om te gaan dan met de enorme felheid waarmee deze discussie tot nu toe wordt gevoerd. Een aantal horecagelegenheden valt niet onder deze uitzondering, maar daar staan de asbakken*

23. TK, 2009–2010, 32011, nr.3.
24. TK, 2009–2010, 32011, nr.5.
25. TK, 2010–2011, 32011, nr.15.

wel op tafel. Dat zijn er veel te veel. Een wet is er om nageleefd te worden. Dat betekent dat je moet handhaven.." (p.20)

In hoeverre en hoe is draagvlak voor de preventiemaatregel verworven?

Bij het toewerken naar een rookvrije horeca heeft de politiek zeer veel rekening gehouden met het (verwerven van) draagvlak voor deze preventiemaatregel bij de bevolking en horecaondernemers. Allereerst heeft minister Borst-Eilers ervoor gekozen om de horeca voorlopig uit te zonderen van het wettelijk recht op een rookvrije werkplek dat op 1 januari 2004 van kracht is geworden. Daarna heeft minister Hoogervorst in het kader van de zelfregulering van het rookbeleid in de horeca voor de periode 2005 t/m 2008 een stappenplan met KHN afgesproken, waarbij ieder jaar steeds meer horecagelegenheden rookvrij zouden worden. In juni 2007 heeft minister Klink gebroken met de geleidelijke invoering van de rookvrije horeca door de overeenkomst met KHN (het stappenplan) eenzijdig op te zeggen en de door KHN voorgestelde fasering van de invoering van het wettelijk rookverbod naast zich neer te leggen (§13.3.1). Het kan haast niet anders dan dat dit ten koste is gegaan van het draagvlak voor het wettelijk rookverbod in de horeca (met name wat betreft cafés en discotheken) bij horecaondernemers en KHN.

Hoeveel draagvlak is er voor de preventiemaatregel?

Draagvlak bij de doelgroep waarop de preventiemaatregel zich richt De doelgroep van de preventiemaatregel bestaat uit alle rokers in de horeca: horecahouders, horecawerknemers en horecabezoekers. Van deze doelgroep is alleen de opvatting van de horecahouders over het rookverbod afzonderlijk onderzocht. (De horecahouders, horecawerknemers en horecabezoekers maken allen deel uit van de bevolking, waarvan de opvatting over het rookverbod wel is onderzocht). In 2008 is 55% van de horecahouders negatief over het rookverbod in de horeca. In 2009 is dit gedaald tot 46%. In 2009 heeft 59% van de caféhouders, 75% van de discotheekhouders en 23% van de restauranthouders een negatieve houding ten aanzien van het rookverbod. (Regioplan, oktober 2009, p.21)

Draagvlak bij belanghebbenden die worden beschermd tegen schade, hinder of overlast
Het doel van de preventiemaatregel is de bescherming van niet-rokende werknemers tegen passief roken in de horeca. Het is zeer opmerkelijk dat hun opvatting over het rookverbod in de horeca niet afzonderlijk is onderzocht.

Draagvlak bij partijen die de preventiemaatregel uitvoeren en/of handhaven
In de praktijk dienen de horecahouders en horecawerknemers het rookverbod te handhaven. Daarom is het opmerkelijk dat het draagvlak voor het rookverbod onder werknemers in de horeca niet afzonderlijk is onderzocht. (De horecawerknemers maken deel uit van de bevolking, waarvan de opvatting over het rookverbod wel is onderzocht).

Draagvlak bij partijen die voor- of nadeel ondervinden van de preventiemaatregel
Hierover is geen informatie beschikbaar die een aanvulling vormt op wat hierboven als is vermeld.

Draagvlak bij de bevolking
De meerderheid van de bevolking staat positief tegenover het rookverbod in de horeca (Regioplan, oktober 2009, figuur 3.3, p.20) (n=32550):
- 55% vindt het een goede tot zeer goede zaak;
- 21% staat er neutraal tegenover;
- 23% vindt het een slechte tot zeer slechte zaak.

Draagvlak bij de politiek
In november 2008 debatteren de vaste commissie van de Tweede Kamer voor VWS en minister Klink over de (vermeende) concurrentievervalsing tussen grote en kleine cafés[26]:

> *"Mevrouw Joldersma (CDA): … Ik ben bereid om te kijken naar degenen die iets niet kunnen. Ik ben niet bereid om degenen te helpen die niet willen, die gewoon hebben afgewacht tot 1 juli 2008, niets hebben gedaan en niet mee willen denken over een rookvrije horeca. De heer Zijlstra [VVD] voedt deze ondernemers en voedt de burgerlijke ongehoorzaamheid."*

Hoewel er ten tijde van de invoering van het rookverbod in de horeca hiervoor een politieke meerderheid was, was de politiek toch erg verdeeld. Twee grotere politieke partijen – VVD en PVV – hebben bijna voortdurend publiekelijk kritiek gehad op het wettelijk rookverbod in de horeca en met name op de toepassing van dit rookverbod op kleine cafés zonder personeel. Dit heeft het draagvlak voor en de handhaafbaarheid van het rookverbod mogelijk negatief beïnvloed, maar dit is niet onderzocht.

26. TK, 2008–2009, 22894, nr.207.

Overige opmerkingen naar aanleiding van de citaten over draagvlak

Uit het citaat van minister Borst-Eilers blijkt dat 'geen bezwaar' soms ook een vorm van draagvlak is:

> *"De regering heeft geen bericht ontvangen van de Europese Commissie in het kader van de notificatie-procedure. Dit betekent dat er geen bezwaren kenbaar zijn gemaakt."*

Uit het citaat van de Tweede Kamerlid Marijnissen blijkt dat draagvlak te maken heeft met normen en waarden:

> *"…dat door dit soort maatregelen een sociaal klimaat kan ontstaan waarin niet-roken de norm is en het niet beginnen of stoppen met roken het gevolg kan zijn."*

Dit blijkt ook uit het citaat van minister Borst-Eilers:

> *"… Het verdient overweging bepaalde sectoren van de samenleving nog enige tijd te gunnen om via zelfregulering alvast vooruit te lopen op de gewenste en beoogde situatie: een rookvrije omgeving op het werk, in het openbaar vervoer en in de voor publiek toegankelijke gebouwen. Als dat lukt, dan vindt nadien via het in werking laten treden van artikel 11a codificatie van een al gegroeide sociale norm in de Tabakswet plaats."*

Als het draagvlak voor een preventiemaatregel beperkt is, kan flankerend beleid nodig zijn, om normen en waarden te veranderen en zo het draagvlak te vergroten. Dit is een onderbouwing van mijn keuze om het criterium 'draagvlak' van het model vooraf te laten gaan aan het criterium 'flankerend beleid'.

Uit het citaat van minister Hoogervorst blijkt dat de internationale inkadering van de rookvrije horeca belangrijk is voor het (politieke) draagvlak. In het algemeen kan aansluiting van een preventiemaatregel op het beleid van stakeholders (bv. overheden, branchegenoten, cliëntenorganisaties, beroepsorganisaties en ideële organisaties) bijdragen aan het politieke of maatschappelijke draagvlak voor de maatregel. Het aspect 'aansluiting van de preventiemaatregel op het beleid van stakeholders' ontbrak nog aan de operationalisering van het criterium 'draagvlak' van het model en zal ik hieraan toevoegen.

13.2.3 Eindoordeel (criterium draagvlak)

Het draagvlak voor het rookverbod in de horeca bij de bevolking lijkt groot genoeg om de implementatie van de preventiemaatregel per 1 juli 2008 te rechtvaardigen. Ook wordt het draagvlak voor implementatie van de preventiemaatregel versterkt

door de internationale inkadering van het streven van de Nederlandse overheid om tot een rookvrije horeca te komen.

Horecaondernemers spelen een cruciale rol bij de handhaving van het rookverbod in de horeca en daarom is het essentieel dat er bij hen voldoende draagvlak voor het rookverbod bestaat. Het draagvlak voor het rookverbod in de horeca bij de horecaondernemers is in 2008 echter zorgwekkend klein. In 2008 is ruim de helft (55%) van de horecahouders negatief over het rookverbod in de horeca. In 2007 en 2008 had de overheid zich moeten inspannen om het in de voorafgaande jaren opgebouwde draagvlak bij KHN en horecaondernemers voor het rookverbod in de horeca te behouden en vergroten. Dat is niet gebeurd. In plaats daarvan heeft minister Klink het draagvlak (onbedoeld) ondermijnd door de overeenkomst met KHN (het stappenplan) eenzijdig op te zeggen en de door KHN voorgestelde fasering van de invoering van het wettelijk rookverbod naast zich neer te leggen (§13.3.1).

Uit de door mij bestudeerde documenten komt het beeld naar voren dat de overheid bij (de voorbereiding van) de implementatie van het rookverbod in de horeca in 2007 en 2008 weinig aandacht heeft besteed aan het verkrijgen van inzicht in en het versterken van het draagvlak voor de preventiemaatregel bij de verschillende stakeholders. Zo is niet bekend hoe groot het draagvlak voor de preventiemaatregel bij (niet-rokende) werknemers in de horeca is (om wie alles is begonnen).

Op basis van de bovenstaande overwegingen kom ik tot de conclusie dat bij (de voorbereiding van) de implementatie van het rookverbod in de horeca in 2007 en 2008 onvoldoende aandacht is besteed aan het draagvlak voor de implementatie van het wettelijk rookverbod.

13.2.4 Gevolgen van de beoordeling voor het model (criterium draagvlak)

De aansluiting van de preventiemaatregel op het beleid van stakeholders kan van belang zijn voor het draagvlak van de preventiemaatregel. Dit aspect voeg ik daarom toe aan het criterium draagvlak van het model.

In §12.4.4 (criterium 'rechtvaardigheid') heb ik geconcludeerd dat per criterium van het model beoordeeld dient te worden of procedurele aspecten relevant zijn voor de beoordeling van de preventiemaatregel aan de hand van het betreffende criterium. De invoeringsprocedure (in dit geval zonder consensus met KHN vaststellen van de invoeringsdatum door minister Klink) blijkt van belang te (kunnen)

zijn voor de beoordeling van de preventiemaatregel aan de hand van het criterium ‘draagvlak’.

13.3 Dertiende criterium: flankerend beleid

13.3.1 Operationalisering en wijze van beoordeling

Flankerend beleid is beleid bedoeld om de uitvoering van een maatregel of wet te ondersteunen[27]. Dit kan zowel door stimulerende maatregelen (bijvoorbeeld het stimuleren van het draagvlak voor het rookverbod in de horeca door voorlichting aan de bevolking over de schadelijke effecten van passief roken) als door het wegnemen van belemmeringen (bijvoorbeeld een soepele opstelling van gemeenten bij de aanvraag van terrasvergunningen door cafés). Over drie vormen van flankerend beleid wil ik enkele nadere opmerkingen maken:
- voorlichtings- of invoeringscampagne;
- inventarisatie en oplossing van knelpunten;
- handhavingsbeleid.

Een voorlichtingscampagne over of invoeringscampagne voor de preventiemaatregel kan men interpreteren als flankerend beleid, maar ook als onderdeel van de preventiemaatregel. Er is sprake van flankerend beleid, omdat heel veel beleid wordt doorgevoerd zonder (uitgebreide) voorlichtings- of invoeringscampagne. Mensen of organisaties komen vanzelf achter de beleidswijzigingen als ze er in de praktijk mee geconfronteerd worden. Zo bezien is een voorlichtings- of invoeringscampagne iets extra’s ter ondersteuning van de beleidsmaatregel. De kosten voor een voorlichtings- of invoeringscampagne kunnen worden toegerekend aan de betreffende preventiemaatregel. In dat geval kan men ook redeneren dat de voorlichtings- of invoeringscampagne onderdeel is van de preventiemaatregel en dus geen flankerend beleid vormt. Ten behoeve van het model kies ik voor een ruime definitie van het criterium ‘flankerend beleid’, omdat het minder bezwaarlijk is als bepaalde onderdelen van de preventiemaatregel bij diverse criteria worden beoordeeld (meer dan eens worden beoordeeld) dan dat bepaalde onderdelen van de preventiemaatregel aan de beoordeling ‘ontsnappen’ (niet worden meegenomen bij de beoordeling van de gerechtvaardigdheid van de preventiemaatregel). Op basis van deze redenering vallen een ‘voorlichtings- of invoeringscampagne’, de ‘inventarisatie en oplossing van knelpunten’ en het ‘handhavingsbeleid’ binnen

27. http://www.thesauruszorgenwelzijn.nl/flankerendbeleid.htm

het kader van het model ook onder het flankerend beleid bij de preventiemaatregel.

Vanwege het grote belang en de complexiteit van het handhavingsbeleid kent het model ten behoeve van deze vorm van flankerend beleid een afzonderlijk criterium 'handhaafbaarheid'. Daarom wordt het handhavingsbeleid niet onder het criterium 'flankerend beleid', maar onder het criterium 'handhaafbaarheid' besproken. Omdat het handhavingsbeleid kan worden gezien als onderdeel van het flankerend beleid heb ik ervoor gekozen om de beoordelingscriteria 'flankerend beleid' (13e criterium) en handhaafbaarheid' (14e criterium) samen in één beoordelingscluster onder te brengen.

Bij de beoordeling van flankerend beleid verdient het m.i. aanbeveling om in ieder geval aan de volgende aspecten aandacht te besteden:
- inbedding in breder preventiebeleid;
- voorlichting over de preventiemaatregel aan betrokken partijen;
- knelpunten die de implementatie van de preventiemaatregel belemmeren en knelpunten ten gevolge van de preventiemaatregel;
- handhavingsbeleid (zoals toegelicht wordt het handhavingsbeleid pas beoordeeld in de volgende paragraaf, bij het criterium 'handhaafbaarheid').

Optimalisatiebeginsel als aanvullend uitgangspunt voor de beoordeling
De wijze van beoordeling van het criterium 'flankerend beleid' heeft mij heel wat hoofdbrekens gekost. Ik heb geworsteld met de vraag hoe kan worden beoordeeld of het flankerend beleid al dan niet voldoende is (geweest).

De beoordeling van de preventiemaatregel aan de hand van de 3e zeef (implementatie) van het model vindt pas plaats nadat de preventiemaatregel de 1e zeef (ontwerp) en 2e zeef (effecten en neveneffecten) is gepasseerd. Dit betekent dat, voorafgaand aan de beoordeling van de preventiemaatregel aan de hand van de 3e zeef, al is vastgesteld dat het ontwerp (1e zeef) en de effecten en neveneffecten (2e zeef) gerechtvaardigd zijn. Stel dat de preventiemaatregel de 2e zeef (effecten en neveneffecten) is gepasseerd op basis van de effecten en neveneffecten die zijn opgetreden na implementatie van de preventiemaatregel. Dan betekent dat dat de implementatie (3e zeef) voldoende is geweest om de effecten en neveneffecten (2e zeef) te rechtvaardigen. Dit hoeft echter niet te betekenen dat er sprake is geweest van een optimaal implementatietraject. Ook een suboptimaal implementatietraject (3e zeef) kan immers tot aanvaardbare effecten en neveneffecten (2e zeef) leiden. Bij de 3e zeef (implementatie) gaat het dus niet alleen om de aanvaardbaarheid van de implementatie (de implementatie leidt of heeft geleid tot aanvaardbare effecten en

neveneffecten), maar ook om de optimalisatie van de implementatie (de implementatie leidt of heeft geleid tot optimale effecten en neveneffecten).

Voor de beoordeling van de preventiemaatregel aan de hand van de 1e en 2e zeef van het model heb ik het schadebeginsel en het beginsel van 'redelijkheid en billijkheid' gekozen. In aanvulling hierop kies ik het 'optimalisatiebeginsel' als uitgangspunt voor de beoordeling van de preventiemaatregel aan de hand van de 3e zeef van het model.

Optimalisatie als uitgangspunt voor de beoordeling van de gerechtvaardigdheid van de 'implementatie van de preventiemaatregel' komt neer op een perfectionistische wijze van beoordelen en leidt tot verschillen van inzicht tussen mensen. Ter illustratie van een dergelijk verschil van inzicht geef ik een al eerder door mij vermeld citaat van minister Schippers over de beschikbare middelen voor de 'handhaving' (14e criterium van het model) van het wettelijk rookverbod in de horeca[28]:

> *"In het verzoek aan de regering staat dat er voldoende toezicht moet zijn. Misschien vindt mevrouw Bouwmeester het niet voldoende, maar ik vind dat wel. … Daar komt bij dat het altijd gemakkelijk is om te zeggen dat het aantal handhavers moet worden uitgebreid. Wij hebben echter ook een huishoudboekje op orde te brengen. Daarvoor moeten heel serieuze en moeilijke keuzes worden gemaakt. Ik maak die keuzes blijkbaar anders dan de PvdA-fractie dat doet."* (p.5)

Ik heb mij ten doel gesteld om een algemeen aanvaard beoordelingsmodel te ontwikkelen. Een perfectionistische wijze van beoordelen dien ik daarom zoveel mogelijk te vermijden. Daarom heb gezocht naar een operationalisering van het begrip 'optimalisatie' die voor iedereen aanvaardbaar is. Onachtzaamheid (onoplettendheid; onvoldoende aandacht besteden aan problemen met het risico op negatieve gevolgen) en nalatigheid (verzuim; binnen de gegeven mogelijkheden zet men zich onvoldoende in om tot optimale effecten en neveneffecten te komen) zijn m.i. vormen van suboptimaal gedrag die algemeen worden afgekeurd. Daarom gebruik ik de begrippen 'onachtzaamheid en nalatigheid' voor het operationaliseren van de (on)gerechtvaardigdheid van de 'implementatie van de preventiemaatregel'.

De implementatie van de preventiemaatregel (3e zeef van het model) acht ik ongerechtvaardigd:
- als de vormgeving of uitvoering van de implementatie onredelijk (onlogisch) of onbillijk (onrechtvaardig) zijn;

28. TK, 2010-2011, vergaderingnr.44, itemnr.6 (dossiernr.32011).

- als er sprake is van onachtzaamheid of nalatigheid bij de vormgeving of uitvoering van de implementatie.

Door bij het beoordelen van de 'optimalisatie van de implementatie' uit te gaan van 'de gegeven mogelijkheden' tracht ik een perfectionistische invalshoek bij het beoordelen van de implementatie zoveel mogelijk te voor komen.

Aanvulling op de wijze van beoordeling bij de criteria 'timing' en 'draagvlak'
Op basis van de bovenstaande overwegingen dient de wijze van beoordeling van de voorgaande criteria 'timing' (11e criterium) en 'draagvlak' (12e criterium) te worden aangevuld. Aan de beoordeling van het criterium 'timing' wordt de vraag toegevoegd of er geen sprake is (geweest) van onachtzaamheid of nalatigheid bij de timing van de implementatie. Aan de beoordeling van het criterium 'draagvlak' wordt de vraag toegevoegd of er geen sprake is (geweest) van onachtzaamheid of nalatigheid bij het verwerven van draagvlak voor de implementatie. Deze aanvullingen hebben geen noemenswaardige invloed op het resultaat van de reeds door mij uitgevoerde beoordeling van 'het rookverbod in de horeca' aan de hand van de criteria 'timing' en draagvlak'. Daarom zal ik het wettelijk rookverbod in de horeca niet opnieuw aan de hand van deze criteria beoordelen.

Wijze van beoordeling bij het criterium 'flankerend beleid'
Op basis van de voorgaande overwegingen kom ik tot de volgende wijze van beoordeling van het criterium 'flankerend beleid':
- beoordeeld wordt of de vormgeving en uitvoering van het flankerend beleid niet onredelijk (onlogisch) of onbillijk (onrechtvaardig) zijn (geweest);
- beoordeeld wordt of er geen sprake is (geweest) van onachtzaamheid of nalatigheid bij de vormgeving en uitvoering van het flankerend beleid.

Bij de beoordeling van het flankerend beleid dient in ieder geval aandacht te worden besteed aan:
- inbedding in breder preventiebeleid;
- voorlichting over de preventiemaatregel aan betrokken partijen;
- inventarisatie en oplossing van knelpunten (het gaat hierbij zowel om knelpunten die de implementatie van de preventiemaatregel belemmeren, als om knelpunten ten gevolge van de preventiemaatregel).

13.3.2 *Beoordeling van casus 'wettelijk rookverbod in horeca' aan de hand van criterium 'flankerend beleid'*

Ik begin de beoordeling met het geven van een overzicht van citaten die te maken hebben met het criterium 'flankerend beleid'. Vervolgens zal ik (mede) aan de hand van deze citaten de implementatie van de preventiemaatregel aan de hand van het criterium 'flankerend beleid' beoordelen.

Inbedding in breder preventiebeleid

De vaste commissies voor VWV en EZ hebben in september 1996 overleg gevoerd met minister Borst-Eilers van VWS en Wijers van EZ over het tabaksontmoedigingsbeleid[29]:

> *"Mevrouw Oedayraj Singh Varma (GroenLinks): ...Toch hield zij vast aan haar voorkeur voor een reclameverbod, want reclame heeft altijd invloed op jongeren en niet-rokers, terwijl juist niet-roken de norm zou moeten zijn en roken abnormaal gevonden zou moeten worden. Nu wordt nog steeds een groot deel van het preventiebeleid weer teniet gedaan door reclameuitingen."* (p.6)

In de Memorie van toelichting bij haar voorstel tot wijziging van de Tabakswet stelt minister Borst-Eilers[30]:

> *"In algemene zin valt uit deze effecttoetsing op te maken dat het succes van het tabaksontmoedigingsbeleid in hoge mate afhankelijk is van de coherentie van het pakket van maatregelen. Slechts in onderlinge samenhang zullen maatregelen het gewenste effect kunnen sorteren. Maatregelen zullen elkaar versterken, maar het achterwege laten van bepaalde aspecten kan ook het effect van een andere maatregel ondermijnen en bovendien leiden tot tegenstrijdigheid in het beleid."* (p.12)

In november 2003 stelt een rapport van de Algemene Rekenkamer[31]:

> *"Voor de verwezenlijking van het niet-rokenbeleid van het Ministerie van VWS worden de volgende tien beleidsinstrumenten gehanteerd:*
> 1. *wetgeving;*
> 2. *handhaven wetgeving;*
> 3. *massamediale campagnes;*
> 4. *marketingexperiment niet-roken;*
> 5. *subsidies voor ontwikkeling voorlichtingsmaterialen;*
> 6. *ondersteuning bij stoppen voor verslaafde rokers;*
> 7. *adequate zelfregulering;*

29. TK, 1996–1997, 24743, nr.3.
30. TK, 1998-1999, 26472, nr.3.
31. TK, 2003–2004, 29300, nrs. 1–2.

8. *accijnsverhoging;*
9. *internationale initiatieven;*
10. *aansturing van onderzoek, instrumentontwikkeling en bevorderen van toepassing van instrumenten.*

… De tien instrumenten worden zoveel mogelijk in samenhang met elkaar ingezet. Dat de effectiviteit van de instrumenten dan toeneemt, komt naar voren in de Gezondheidseffectenrapportage uit 1998. Deze rapportage is mede gebaseerd op een uitgebreid literatuuronderzoek." (p.27)

In september 2009 schrijft staatssecretaris Timmermans van Buitenlandse Zaken een brief aan de Tweede Kamer over een voorstel van de Europese Commissie om te komen tot een aanbeveling van de Europese Raad aan de lidstaten van de Europese Unie betreffende rookvrije ruimten[32]:

Dit voorstel sluit aan bij het eerder uitgebracht Groenboek «Op weg naar een rookvrij Europa: beleidsopties op EU-niveau» en bij het WHO Kaderverdrag voor bestrijding van tabaksgebruik (FCTC), in het bijzonder bij artikel 8. De voorgestelde aanbeveling is hoofdzakelijk gericht op de effectieve tenuitvoerlegging van dit artikel binnen de EU.

Artikel 8 van het WHO FCTC-verdrag betreft effectieve bescherming tegen blootstelling aan tabaksrook in afgesloten werkplekken, afgesloten openbare ruimten, openbare vervoermiddelen en waar van toepassing ook in overige openbare ruimten. Nederland is samen met nog 25 EU-lidstaten verdragspartij (in totaal 166 verdragspartijen) en dient de verplichtingen voorvloeiende uit voornoemd artikel 8 op te volgen. Er bestaan zowel tussen als binnen EU-lidstaten nog veel verschillen in niveau van bescherming.

Bescherming tegen blootstelling van tabaksrook is zowel vanuit financieel als ook vanuit gezondheidperspectief effectief en zinvol. De Commissie stelt dat een breed «rookvrij»-beleid (reeds bestaand in diverse lidstaten en landen buiten de EU), tabaksgerelateerde lasten kan verlichten zonder daarbij de economie te schaden.

Het voorstel heeft als doel om lidstaten te ondersteunen bij de implementatie van WHO FCTC artikel 8. Om dit voorstel te concretiseren, doet de Commissie, aanvullend op FCTC artikel 8, een viertal aanbevelingen. Deze zijn: …

2. *flankerende maatregelen zoals effectief beleid om rokers te helpen te stoppen en waarschuwende afbeeldingen op verpakkingen van tabak;*
3. *ontwikkeling van brede multisectoriële strategieën en adequate instrumenten om die uit te voeren; …"* (p.2)

32. TK, 2008–2009, 22112, nr.919.

Op basis van de bovenstaande citaten realiseer ik me dat het er niet alleen om gaat dat flankerend beleid de preventiemaatregel ondersteunt, maar ook dat er geen beleid is dat strijdig is met de preventiemaatregel. Ter illustratie een fictief voorbeeld. Als een ministerie van volksgezondheid succesvol is in haar streven om het roken binnen de bevolking terug te dringen, moet een ministerie van economische zaken niet de mogelijkheden tot het maken van reclame voor roken verruimen om banenverlies in de Tabaksindustrie te bestrijden. De Europese Commissie pleit m.i. mede daarom voor *"brede multisectoriële strategieën"*. De vraag, of er geen beleid is dat strijdig is met de preventiemaatregel, voeg ik toe als aandachtspunt bij de beoordeling van de preventiemaatregel aan de hand van het criterium ´flankerend beleid´.

Voorlichting

In juli 1999 stelt de PvdA in een brief van de vaste commissie voor VWS aan minister Borst-Eilers[33]:

> *"Het is deze leden overigens bekend dat het al jaren bestaande rookverbod in allerlei sectoren van de gezondheids- en onderwijssector pas de laatste jaren enige geldingskracht gaat krijgen. Op welke wijze wil de regering bevorderen dat het al bestaande rookverbod, inclusief de te verwezenlijken uitbreiding daarvan, goed wordt nageleefd? Bestaan er plannen voor voorlichtingscampagnes, of wordt de nadruk gelegd op de bestuursrechtelijke handhaving?* " (p.20)

In 2002 schrijft minister Borst-Eilers aan de Eerste Kamer in een 'Nadere memorie van antwoord' op vragen over haar 'Wetsvoorstel tot wijziging van de Tabakswet'[34]:

> *"In reactie op de leden van de VVD-fractie over het nut van en de voorkeur voor preventie en voorlichting ten opzichte van wetgeving als beleidsinstrument is de regering van mening dat het een niet zonder het ander kan. De kracht van het tabaksontmoedigingsbeleid ligt dan ook in de samenhangende aanpak. Wetgeving versterkt voorlichting en omgekeerd. En accijnsverhoging brengt meer rokers ertoe om nu eindelijk toch maar eens te proberen er écht mee te stoppen. En zelfregulering in de vorm van een rookvrije omgeving heeft de juiste voorbeeldwerking op anderen. Zo zijn er diverse mechanismen, waarlangs de beleidsinstrumenten elkaar versterken."* (p.10)

Uit het bovenstaande citaat blijkt dat er sprake kan zijn van synergie tussen beleidsmaatregelen, die op die manier flankerend beleid voor elkaar vormen.

33. TK, 1998–1999, 26472, nr.5
34. EK, 2001–2002, 26472, nr.59c.

Inventarisatie en oplossing van knelpunten

In juli 2007 overlegt de vaste commissie voor VWS van de Tweede Kamer met minister Klink over zijn besluit tot invoering van het rookverbod in de horeca per 1 juli 2008[35]:

> *"Mevrouw Schippers (VVD): … Veel horecagelegenheden zijn te klein om rookruimtes te creëren. Heeft de minister nagedacht over flankerend beleid om te voorkomen dat in uitgaanscentra overlast ontstaat door op straat vertoevende rokers die vroeger binnen konden roken? En hoe wil de minister handhaven? Moet de kroegbaas politieagent gaan spelen? Wordt er een peloton nieuwe inspecteurs aangesteld dat de kroegen gaat afstruinen?"* (p.3)

In november 2008 debatteren de vaste commissie van de Tweede Kamer voor VWS en minister Klink over de (vermeende) concurrentievervalsing tussen grote en kleine cafés[36]:

> *Minister Klink: … Betekent dit dat ik per definitie blind ben voor de effecten? Nee, want wij komen in de loop van december met een knelpuntennotitie. Volgend jaar gaan wij een en ander nauwgezet monitoren. Betekent dit dat het denken per definitie stil staat? Eenmaal een wet, altijd een wet? Dat hoeft ook niet per se. Ik zie nu echter, gegeven de ontwikkelingen, geen enkele aanleiding om te zeggen dat wij van het rookverbod moeten afwijken. Laten wij eerst een poosje goed handhaven en zorgen dat een en ander goed wordt nageleefd.* (p.13)

> *De heer Zijlstra vraagt welke oplossingen ik bied voor de knelpunten. Ik heb zo-even gezegd dat wij nog een knelpunteninventarisatie aan het maken zijn. Wij bekijken waar de knelpunten liggen. Op voorhand heb ik geen enkele aanleiding om tot compensatie over te gaan in financiële zin, zoals de heer Van Gerven bedoelde, of te definiëren vanaf welke oppervlakte cafés gevrijwaard zijn van het rookverbod.* (p.13)

> *Minister Klink: … Dan over de compensatie. Het kan een tijdelijk fenomeen zijn. Mevrouw Bouwmeester haalde het voorbeeld aan van een accijnsverhoging bij tabak of alcohol, dat niet voortdurend van overheidswege wordt gecompenseerd. Dat doen wij ook bij de pompstations in de grensstreek niet als de accijns op benzine wordt verhoogd. Ik heb dat voorbeeld eerder gebruikt. In de regel doen wij het niet. … Daarbij komt dat hier een heel traject van zelfregulering aan vooraf is gegaan, wat geflankeerd werd door een verbod op de werkplek voor iedereen. In die zin is er altijd een uitzondering voor de horeca gemaakt. Die voegen wij in de volledige reeks van rookvrije werkplekken. Dat had men kunnen weten. Het zelfreguleringstraject is overgegaan in een aankondiging bij het regeerakkoord, inmiddels al bijna twee jaar*

35. TK, 2006–2007, 30800 XVI, nr.182.
36. TK, 2008–2009, 22894, nr.207.

geleden. In die zin betekent ondernemerschap je oriënteren op de veranderingen die zich aftekenen en daar gepaste maatregelen op nemen.(p.13)

13.3.3 Eindoordeel (criterium flankerend beleid)

Op basis van het bovenstaande overzicht van citaten en de door mij bestudeerde parlementaire documenten concludeer ik dat de achtereenvolgende ministers van VWS en het parlement zich ruim voldoende hebben ingezet (geen nalatigheid) om tot een goed flankerend beleid ter ondersteuning van de toepassing van de preventiemaatregel te komen. De politiek heeft zeer veel aandacht besteed (geen onachtzaamheid) aan alle door mij benoemde aspecten van het criterium flankerend beleid:

- inbedding in breder preventiebeleid;
- voorlichting;
- inventarisatie en oplossing van knelpunten.

Het flankerende beleid is niet onlogisch (met name de inbedding in een breder tabaksontmoedigingsbeleid is doelmatig) en niet onrechtvaardig geweest.

Op mijn positieve eindoordeel over het flankerend beleid maak ik één uitzondering. M.i. had minister Klink diepgaander onderzoek moeten doen naar het knelpunt van omzetdalingen in kleine cafés (zonder personeel) ten gevolge van het rookverbod. Op dit punt is er sprake van onachtzaamheid (geen oog hebben voor) en daardoor mogelijk van nalatigheid (niet handelen). Het besluit van minister Klink om kleine cafés met grote omzetdalingen niet (met flankerend beleid) tegemoet te komen, is ***onvoldoende met feiten onderbouwd***. Zie voor een nadere toelichting onder het criterium ‘rechtvaardigheid’ (zie §12.4.3).

13.3.4 Gevolgen van de beoordeling voor het model (criterium flankerend beleid)

De wijze van beoordeling van het criterium ‘flankerend beleid’ heeft mij heel wat hoofdbrekens gekost. Uiteindelijk ben ik tot de onderstaande wijze van beoordeling gekomen.

Beoordeeld wordt:

- of de vormgeving en uitvoering van het flankerend beleid niet onredelijk (onlogisch) of onbillijk (onrechtvaardig) zijn (geweest);
- of er geen sprake is (geweest) van onachtzaamheid of nalatigheid bij de vormgeving en uitvoering van het flankerend beleid.

Deze uitgangspunten voor de beoordeling – redelijkheid, billijkheid, geen onachtzaamheid, geen nalatigheid – gelden niet alleen voor het criterium 'flankerend beleid', maar voor alle criteria van de 3e zeef (implementatie) van het model. Door bij het beoordelen van de 'optimalisatie van de implementatie' uit te gaan van 'de gegeven mogelijkheden' wordt een perfectionistische invalshoek zoveel mogelijk voorkomen.

De vraag, of er geen beleid is dat strijdig is met de preventiemaatregel, heb ik toegevoegd als aandachtspunt bij de beoordeling van de preventiemaatregel aan de hand van het criterium ´flankerend beleid´.

13.4 Veertiende criterium: handhaafbaarheid

13.4.1 Operationalisering en wijze van beoordeling

In het kader van het model definieer ik handhaving als handelingen gericht op bevordering van naleving van (rechts)regels en beëindiging van overtredingen van (rechts)regels[37]. Ik heb het woord 'rechts' tussen haakjes geplaatst, omdat het in het kader van het model niet alleen gaat om de handhaving van wettelijk regels. Het kan bijvoorbeeld, zoals in de eerder besproken casus van Clarian Health, ook gaan om regels die een werkgever heeft vastgesteld.

Het criterium 'handhaafbaarheid' betreft zowel de 'handhaving' van de preventiemaatregel, als het 'toezicht op deze handhaving'. Het onderscheid tussen de begrippen 'naleving', 'handhaving' en 'toezicht op de handhaving', zal ik toelichten aan de hand van de casus 'wettelijk rookverbod in de horeca':
- de ***'naleving'*** betreft de mate waarin rokers zich in de horeca houden aan het wettelijk rookverbod;
- de ***'handhaving'*** betreft de handhaving van het wettelijk rookverbod in de horeca door horeca-eigenaars en horecawerknemers;
- het ***'toezicht op de handhaving'*** betreft het toezicht van de nieuwe Voedsel en Waren Autoriteit (nVWA) op de handhaving van het wettelijk rookverbod in de horeca.

In de praktijk wordt vaak geen onderscheid gemaakt tussen 'handhaving' en 'toezicht op de handhaving'. Dit onderscheid heeft mij echter goed geholpen bij de

37. http://www.thesauruszorgenwelzijn.nl/handhaving.htm

analyse van de handhaving van het wettelijk rookverbod in de horeca. Naar aanleiding hiervan stel ik voor om per preventiemaatregel af te wegen, of het maken van dit onderscheid behulpzaam is bij de beoordeling van de preventiemaatregel aan de hand van het model. Als dit onderscheid niet behulpzaam is, hoeft dit onderscheid niet te worden gemaakt en vervalt de term 'toezicht op de handhaving'.

Beoordeeld wordt of:
- het ontwerp van de 'preventiemaatregel' de naleving en (daarmee) handhaving van de preventiemaatregel waar mogelijk bevordert en niet onnodig ondermijnt;
- de vormgeving en uitvoering van de 'handhaving' en van het 'toezicht op de handhaving' niet onlogisch (ondoelmatig) en niet onrechtvaardig zijn;
- er geen sprake is van onachtzaamheid of nalatigheid bij de vormgeving en uitvoering van de 'handhaving' en van het 'toezicht op de handhaving'.

13.4.2 Beoordeling van casus 'wettelijk rookverbod in horeca' aan de hand van criterium 'handhaafbaarheid'

Ik begin de beoordeling met het geven van een chronologisch overzicht van citaten die te maken hebben met het criterium 'handhaafbaarheid'. Vervolgens zal ik (mede) aan de hand van deze citaten de implementatie van de preventiemaatregel aan de hand van het criterium 'handhaafbaarheid' beoordelen.

In september 1996 hebben de vaste commissies van de Tweede Kamer voor VWS en EZ overleg gevoerd met de ministers Borst-Eilers van VWS en Wijers van EZ over het tabaksontmoedigingsbeleid[38]:

> *"Mevrouw Van Blerck-Woerdman (VVD): … Zij wees er vervolgens op dat uit onderzoek van de inspectie gezondheidsbescherming, medio 1995, is gebleken dat het rookverbod in openbare gebouwen vaak niet wordt nageleefd. … Verder leek het haar zinvoller om, in plaats van nieuwe rookverboden aan te kondigen, eerst de bestaande weten regelgeving beter te handhaven. Anders gaat het sterk in de richting van symboolwetgeving."* (p.7)

In 1999 onderbouwt minister Borst-Eilers in de memorie van toelichting bij haar wetsvoorstel tot wijziging van de Tabakswet uitgebreid en overtuigend haar voorstellen tot:
- introductie van bestuurlijke handhaving en bestuurlijke boetes ter handhaving van de rookverboden;

38. TK, 1996–1997, 24743, nr.3.

– van toepassing blijven van het strafrecht c.q. de Wet op de economische delicten als ultimum remedium[39]:

"Het huidige kabinetsbeleid … houdt onder meer een versterking van de bestuurlijke handhaving in. … Tevens geldt dat het bestuursorgaan belast met de uitvoering van vaak zeer specifieke wetgeving qua deskundigheid en ervaring in beginsel beter dan andere overheidsinstellingen is toegerust om de handhaving efficiënt en effectief te laten zijn. Een dergelijk bestuursorgaan – vaak ook belast met het toezicht op de naleving – is veelal goed op de hoogte van de werkwijze van «het veld», de ontwikkelingen, de actoren en natuurlijk inhoud en doelstelling van het overtreden voorschrift." (p.14)

"Wel merk ik hier nog op dat ik voornemens ben … een meldpunt annex klachtenlijn voor rookoverlast te laten instellen, zodat de burger zich gemakkelijk tot de controlerende instantie kan wenden." (p.14)

"Ik stel dan ook voor het instrument van de bestuurlijke boetes te introduceren voor de reclamebeperkingen, alsmede de verkoop- en rookverboden. Zodoende zal de inspectie W & V ter zake slagvaardiger en adequater kunnen opereren. Overigens zij benadrukt dat de Wet op de economische delicten als ultimum remedium van toepassing blijft." (p.14-15)

"Het karakter van de vigerende en voorgestelde voorschriften is een belangrijke overweging om het instrument van de bestuurlijke boete in de Tabakswet te introduceren. Overtreding pleegt in de regel geen (direct) letsel aan personen of schade aan goederen toe te brengen, noch een direct gevaar daarvoor op te leveren. … Een en ander leidt tot de conclusie dat het enkele overtreden van de norm veelal niet onmiddellijk een situatie in het leven roept, die de rechtsorde ernstig schokt. In dat licht bezien, is een strafrechtelijke sanctie … niet de meest passende reactie. (p.15)

Niettemin kan het overtreden van een wettelijke norm, zeker in combinatie met andere overtredingen, wel degelijk situaties betreffen die op termijn schadelijk zijn voor de volksgezondheid, dan wel leiden tot maatschappelijk onaanvaardbare gedragingen richting de consument. Dit betekent dat adequate handhaving van de normen van de Tabakswet van groot belang is, …" (p.15)

"Bovendien speelt ook de tijdsduur van de procedure een rol. De strafrechtelijke handhavingspraktijk laat zien dat tussen het moment van geconstateerde overtreding en uiteindelijke afdoening door een vonnis … veel tijd verstrijkt. Van een «lik op stuk»-beleid, … komt aldus weinig terecht. De invoering van de bestuurlijke boete in de Tabakswet kan de handhaving van deze wet en de daarop gebaseerde wettelijke voorschriften vanuit dit perspectief bezien, versterken." (p.16)

39. TK, 1998–1999, 26472, nr.3.

In 2000 schrijft minister Borst-Eilers in een brief aan de Tweede Kamer over haar Wetsvoorstel tot wijziging van de Tabakswet[40]:

> *"Evenals de leden van deze fractie [PvdA] heb ik mij in eerste instantie eveneens afgevraagd of niet ook de individuele overtreder van het rookverbod, de roker, bestraft zou moeten worden. Mede gelet op de plaatsen waar zulke overtredingen plaatsvinden, zal het echter niet goed mogelijk zijn om zo een wettelijke bepaling in redelijke mate te handhaven. Het voor zo'n overtreding uitdelen van een bestuurlijke boete door het personeel of de beheerder van de instelling is niet mogelijk. Verder schat ik in dat bij het bestraffen van de roker zelf er een aannemelijke kans bestaat op een negatieve uitwerking. Uiteraard dient de individuele overtreder wel terstond door het personeel van de instelling, de beheerder zelf of het bezoekende publiek te worden aangesproken op zijn rookgedrag, omdat hij of zij niet alleen een maatschappelijke norm overtreedt, maar ook de huisregels van de instelling die voor een deel wettelijk zijn vastgelegd."* (p.52-53)

In 2002 stelt Eerste Kamerlid Werner bij de behandeling van het Wetsvoorstel tot wijziging van de Tabakswet in de Eerste Kamer[41]:

> *"De heer Werner (CDA): ... Wij hebben liever een beperktere wet die simpel en helder is in zijn argumentatie en die misschien hier en daar wat laat schieten, maar die goed te handhaven is dan een heleboel maatregelen waarmee iedereen uiteindelijk een loopje neemt, waardoor het effect van de maatregelen buitengewoon gering zal zijn."* (p.1266)

Over de wijze van beoordeling van de handhaafbaarheid heb ik geschreven: *"Beoordeeld wordt of het ontwerp van de 'preventiemaatregel' de naleving en (daarmee) handhaving van de preventiemaatregel waar mogelijk bevordert en niet onnodig ondermijnt."* Het bovenstaande citaat van Eerste Kamerlid Werner vormt een onderbouwing van mijn uitgangspunt voor de beoordeling, dat het ontwerp van de preventiemaatregel van grote invloed kan zijn op de handhaafbaarheid.

In 2005 stelt het rapport 'Handhaven en gedogen' van de Algemene Rekenkamer[42]:

> *"Transparantie van het handhavingsbeleid. De keuzes die beleidsmakers en handhavende instanties maken ten aanzien van de handhaving blijven veelal impliciet. Zo maken zij over het algemeen niet duidelijk welk niveau van naleving zij minimaal beogen, welke onderdelen van de wet- en regelgeving zij minder intensief of bewust niet handhaven (posterioriteiten), en wat zij onder welke condities gedogen. Als bezwaar tegen het wél expliciteren van dergelijke keuzes, wordt vaak aangevoerd*

40. TK, 1999-2000, 26472, nr.6.
41. EK, 2001-2002, vergaderingnr.24, p.1236-1275 (dossiernr.26472).
42. TK, 2004-2005, 30050, nr.2.

dat dit de feitelijke naleving zou kunnen verminderen. Dit probleem kan naar het oordeel van de Algemene Rekenkamer worden ondervangen door een goede communicatie en het doorgaan met controle van bekende duidelijk geïdentificeerde risicogroepen." (p.6)

Belangrijk voor het model is dat de Algemene Rekenkamer erop wijst dat in bepaalde gevallen ook gedogen een gerechtvaardigde beleidskeuze kan zijn. Gedogen brengt echter ook het risico van verwatering van de handhaving met zich mee. Ik zal het begrip 'gedogen' toevoegen als aandachtspunt bij de 'operationalisering en wijze van beoordeling' van het criterium 'handhaafbaarheid'. In het handhavingsbeleid van het rookverbod in de horeca speelt gedogen geen rol.

In juni 2007 stuurt minister Klink de Tweede Kamer een brief waarin hij zijn besluit toelicht om per 1 juli 2007 een wettelijk rookverbod voor de horeca in te voeren[43]:

"KHN heeft toen,… voorgesteld om de sectoren hotels, restaurants en fastfood per 1 januari 2008 rookvrij te maken. Voor de sectoren cafés en discotheken wil KHN een apart traject dat … niet eerder dan 1 januari 2011, ook leidt tot een rookvrije situatie. …Als criterium om onderscheid te maken tussen de sectoren cafés en restaurants stelt KHN voor het «zittend gebruik maken van maaltijden»." (p.3)

"Ik heb besloten om niet mee te gaan in het voorstel van KHN omdat: …

3. *het onderscheid in de praktijk moeilijk hanteerbaar zal zijn en zal leiden tot «ontwijkgedrag» door ondernemers: wat is zitten?, roken voor en na sluitingstijd keuken, vallen barmaaltijden onder «zittend eten»?, enzovoorts;*
4. *het ingewikkelde onderscheid en de vele grensgevallen de handhaving bemoeilijken en aanleiding zullen zijn voor het ter discussie stellen van het optreden van de VWA en het aanvechten van opgelegde boetes;*
5. *de ervaringen in bijvoorbeeld België leren dat een onderscheid tussen subsectoren ingewikkeld is en in de praktijk veel onduidelijkheid geeft bij horecaondernemers, de handhavende instantie en het publiek."* (p.3-4)

"Mijns inziens is iedereen gebaat bij duidelijkheid ten aanzien van dit onderwerp. Daarom heb ik alles serieus afwegende besloten om:

- *geen onderscheid te maken tussen horecasectoren;*
- *de gehele horeca (inclusief bedrijven zonder personeel) en alle podia per 1 juli 2008 rookvrij te maken;"* (p.4)

In de bovenstaande citaten geeft minister Klink een heldere uitleg van de relatie die er volgens hem bestaat tussen enerzijds het ontwerp van de preventiemaatregel en

43. TK, 2006–2007, 30800 XVI, nr.149.

anderzijds de naleving en handhaafbaarheid. Met het oog op duidelijkheid pleit hij voor eenvoud.

In juli 2007 overlegt de vaste commissie voor VWS van de Tweede Kamer met minister Klink over zijn besluit tot invoering van het wettelijk rookverbod in de horeca per 1 juli 2008[44]:

> *"[Minister Klink:] De cultuuromslag is vrij breed, zowel bij de ondernemers als bij het publiek. Het merendeel van de Nederlandse bevolking is voorstander van het rookvrij maken van de horeca. Dat is belangrijk voor de naleving. De sociale controle is ook vrij groot. De minister meent dat handhaving spontaan zal plaatsvinden."* (p.8)

Belangrijk voor het model is dat minister Klink erop wijst dat 'sociale controle' ook een belangrijke rol kan spelen bij de handhaving. M.i. ondermijnt minister Klink met zijn keuze om de bescherming van bezoekers in de horeca tegen passief roken geen doelstelling van het wettelijk rookverbod te laten zijn, de sociale controle op naleving van het rookverbod. Bezoekers kunnen immers niet ten behoeve van zichzelf naleving van het rookverbod eisen. Opmerkelijk is dat dit argument in het politieke debat door niemand is aangevoerd. Deze ondermijning van de sociale controle lijkt mij echter onvoldoende reden om, met het oog op de handhaafbaarheid, de beperking van de doelstelling van het wettelijk rookverbod tot de bescherming van werknemers ongerechtvaardigd te verklaren. Ik zal het begrip 'sociale controle' toevoegen als aandachtspunt bij de 'operationalisering en wijze van beoordeling' van het criterium 'handhaafbaarheid'.

In december 2008 schrijft minister Klink in een brief aan de Tweede Kamer over de handhaving en naleving van het rookverbod[45]:

> *"Handhaving en naleving*
> ***De inzet en werkwijze van de VWA op het gebied van de rookvrije horeca is grofweg in te delen in drie fasen: …"***
>
> ***"Juli tot en met september 2008***
> *Ik heb er … voor gekozen om direct vanaf het begin te handhaven maar in eerste instantie alleen waarschuwingen uit te delen. … In de eerste drie maanden na de invoering van de rookvrije horeca zijn 7264 bedrijven bezocht … Uit deze inspecties blijkt dat 94 procent van de bedrijven een rookverbod heeft ingesteld. … met uitzondering van de cafés. Bij de cafés bleek 74 procent een rookverbod te hebben ingesteld.*

44. TK, 2006–2007, 30800 XVI, nr.182.
45. TK, 2008–2009, 22894, nr.206.

… Uit de resultaten van de herinspecties in de eerste drie maanden blijkt ook duidelijk dat het uitdelen van waarschuwingen effect heeft: bij circa 80 procent van de bedrijven waar een herinspectie heeft plaatsgevonden, is de overtreding opgeheven. De VWA heeft eind september de eerste boetes opgelegd aan horecagelegenheden die bij herinspectie de regelgeving nog niet goed naleefden."

"1 oktober tot 1 december 2008
In de maand september … kwamen meer berichten dat bepaalde cafés het rookverbod bewust gingen overtreden, dat dit in georganiseerd verband ging plaatsvinden en dat om die reden de handhaving steviger moest worden. Daar waar sprake is van bewuste, moedwillige overtreding van het rookverbod is de VWA vanaf 1 oktober direct boetes op gaan leggen. … De VWA is zich vooral gaan richten op de sector cafés («natte horeca») waar de naleving achterblijft. … In de periode 1 oktober tot circa 1 december heeft de VWA circa 2800 horecagelegenheden bezocht. … Ook zijn er bij diverse bedrijven (enkele tientallen) een tweede, derde of zelfs vierde en vijfde boete opgelegd. In totaal zijn er in de periode 1 juli tot circa 1 december zo'n 10 000 bedrijven door de VWA gecontroleerd en heeft de VWA in totaal zo'n 474 waarschuwingen gegeven en zo'n 821 boetes (of processen-verbaal) opgemaakt."

"Vanaf 1 december 2008
Op 17 en 26 november 2008 heb ik u gemeld dat de VWA … gebruik zal gaan maken van de Wet op de Economische Delicten (WED). De controleurs zullen in ieder geval proces-verbaal opmaken in die gevallen waarin horecaondernemers reeds tweemaal of vaker een bestuurlijke boete is aangezegd. Deze zaken zullen zo spoedig mogelijk voor de economische politierechter worden gebracht. De officier van justitie kan een boete eisen van maximaal € 18 500,–, al dan niet in combinatie met een voorwaardelijke stillegging. Voor deze aanpak is gekozen omdat er in delen van de horeca sprake lijkt van een min of meer gecoördineerde actie om het rookverbod te overtreden. … Tijdens het spoed algemeen overleg op 27 november 2008 bleek dat uw Kamer deze aanpak in meerderheid steunt."

Uit de bovenstaande citaten blijkt dat minister Klink wat betreft de handhaving van het wettelijk rookverbod in de horeca een grote inspanning heeft geleverd en dat hem op dit punt geen onachtzaamheid of nalatigheid verweten kan worden.

In zijn brief aan de Tweede Kamer d.d. 1 december 2009 over de resultaten van één jaar rookvrije horeca schrijft minister Klink over de naleving en handhaving van het rookverbod[46]:

"Als gekeken wordt naar de resultaten van representatieve observaties door bureau INTRAVAL in opdracht van de VWA blijkt dat in de winter van 2008/2009, het

46. TK, 2009–2010, 32011, nr.3.

voorjaar 2009, de zomer 2009 en het najaar van 2009 geen rokers zijn aangetroffen in respectievelijk 94, 97, 96 en 95% van de horecagelegenheden. Als gekeken wordt naar de deelsectoren valt op dat bij restaurants, cafetaria's, hotels en recreatie, sportkantines en kunst/ cultuur er nauwelijks veranderingen zijn: daar blijft het percentage gelegenheden waar geen rokers en geen asbakken zijn waargenomen zeer hoog (tussen 97 en 100%). Bij cafés en discotheken is er een daling waarneembaar van het percentage cafés en discotheken waar geen rokers (van 83 naar 73%) en geen asbakken (van 83 naar 69%) zijn waargenomen. De daling is het grootst bij cafés waar tijdens de observatie maar één personeelslid aanwezig was (bij een aanzienlijk deel daarvan zal het gaan om eenmanszaken). De meest voor de handliggende verklaringen voor deze dalingen zijn de uitspraken van de gerechtshoven in Den Bosch en Leeuwarden en mijn beslissing in reactie daarop om de handhaving voor horeca zonder personeel voorlopig op te schorten." (p.2)

In januari 2001 vindt een overleg plaats tussen de vaste commissie van de Tweede Kamer voor VWS en minister Schippers over (de gevolgen van) haar voorstel tot uitzondering van kleine cafés zonder personeel van het wettelijk rookverbod in de horeca[47]:

"Mevrouw Bouwmeester (PvdA): … De heer Cohen heeft zich afgevraagd of een rookverbod in eenmansbedrijven noodzakelijk is. Wij hebben het daarna onderzocht. Daaruit bleek dat het opheffen van het rookverbod voor eenmansbedrijven zou leiden tot een glijdende schaal. … Wij zijn dus heel realistisch en hebben besloten om niet voor zo'n gedeeltelijke opheffing te kiezen." (p.4)

"De heer Van Gerven (SP): Een kwalijke zaak is de manier waarop de minister van Volksgezondheid de versoepeling van het rookverbod heeft aangekondigd. Met haar mantra van antibetutteling … Het gevolg was dat de handhaving van het rookverbod in drie maanden in een vrije val lijkt te zijn beland. Bij discotheken en cafés wordt het verbod nu massaal overtreden. Het laatste schokkende cijfer dat wij hoorden was dat 51% van de horecagelegenheden het rookverbod overtreedt. Drie maanden geleden lag dat percentage nog op 25. Om het draagvlak onder het rookverbod niet nog verder te ondermijnen, is handhaving van cruciaal belang. Dat erkent de minister … Hoe gaat zij ervoor zorgen dat er in de grote zaken goed wordt gehandhaafd …?" (p.6)

"De heer Van der Staaij (SGP): … Ik heb vragen over de handhaafbaarheid en de werkbaarheid van het criterium. … De vraag is wel hoe controleerbaar en gevoelig voor misbruik het criterium is van wel of geen personeel hebben. … Los van de discussie sec over het rookverbod in de kleine horeca speelt de vraag een rol of er niet een maatschappelijk signaal uitgaat van deze focus op het rookverbod in de kleine horeca. Het signaal kan zijn dat de overheid het niet zo nauw neemt met de hand-

47. TK, 2010–2011, 32011, nr.15.

having. Zouden bedrijven die formeel niet onder de criteria vallen, daar niet gemakkelijk in meegaan en besluiten dat het wel weer mag? Er zal een extra handhavingsinspanning nodig zijn …" (p.13)

"Minister Schippers: … Een aantal horecagelegenheden valt niet onder deze uitzondering, maar daar staan de asbakken wel op tafel. Dat zijn er veel te veel. Een wet is er om nageleefd te worden. Dat betekent dat je moet handhaven. … Het handhaven van deze wet is overigens ook niet zo moeilijk, want alle criteria zijn glashelder. Ik wil dat de handhaving veel meer resultaat oplevert. … Ik zit in een situatie met een beperkt aantal handhavers, namelijk ongeveer 200 inspecteurs. Bovendien zit ik in een tijd van inkrimping: er moeten ambtenaren uit. Is uitbreiding hiervan dan aan de orde? Ik heb daar geen geld voor. Wel zijn er, met de uitzondering van deze kleine cafés zonder personeel, relatief meer inspecteurs voor de rest. Dan de route via het Openbaar Ministerie, die door enkele woordvoerders is aangegeven: halsstarrige overtreders voor de rechter dagen. Dat is in het recente verleden in een beperkt aantal gevallen wel degelijk gebeurd, maar dit is een duur, langdurig en arbeidsintensief traject met een geringe opbrengst. …

Eigenlijk wil ik een veel eenvoudiger aanpassing om de effectiviteit van de handhaving fors te verhogen. Ten eerste wil ik de boetes fors verhogen. De boete is nu ongeveer € 300, in vier stappen oplopend tot € 2400. Voor een grote horecazaak is € 300 natuurlijk een heel bescheiden bedrag. De voordelen kunnen bovendien groter zijn dan € 300. Het tweede punt is het idee van mevrouw Uitslag voor een meldlijn. We hebben [al] een meldlijn bij de VWA. … Ik zal met de VWA bespreken dat de klachten zo veel mogelijk worden gevolgd door inspecties. Ten derde wil ik inzetten op meer en snellere herinspecties." (p.20-21)

In februari 2011 stuurt minister Schippers een brief aan de Tweede Kamer, waarin ze haar toezeggingen m.b.t. de aanscherping van het handhavingsbeleid heeft uitgewerkt[48]:

"Verhoging boetes
Nu de kleine cafés zonder personeel worden uitgezonderd van het rookverbod, de huidige boetes onvoldoende afschrikkende werking blijken te hebben, en openlijke overtreding van het rookverbod plaatsvindt, is het gerechtvaardigd de boetebedragen voor het overtreden van het rookverbod in de horeca te verdubbelen. Dit betekent voor de hoogte van de boetes het volgende:

- *1e overtreding: € 600*
- *2e overtreding: € 1200*
- *3e overtreding: € 2400*
- *4e overtreding: € 4800."* (p.1)

48. TK, 2010–2011, 32011, nr.16.

*"**Meldlijn***
Tenslotte wil ik u nog informeren over de werking van de nVWA meldlijn. De nVWA heeft deze lijn (nog) gebruiksvriendelijker gemaakt. Een melding kan binnen enkele minuten worden gedaan, telefonisch of via internet. … De procedure werkt als volgt. Eerst worden alle meldingen zorgvuldig beoordeeld, voordat er actie wordt ondernomen. Op klachten van werknemers wordt altijd actie ondernomen, waarna ook terugkoppeling plaatsvindt naar degene die de klacht heeft ingediend. Meldingen van klanten en de overige klagers worden geïnventariseerd en op basis van ernst en frequentie geprioriteerd voor nadere inspectie." (p.2)

13.4.3 Eindoordeel (criterium handhaafbaarheid)

Uit de bovenstaande citaten wordt duidelijk dat zonder toezicht op de handhaving van het wettelijk rookverbod op de werkplek er van de handhaving en daarmee naleving onvoldoende terechtkomt. In september 1996 spreekt Tweede Kamerlid Van Blerck-Woerdman van *"symboolwetgeving"*. Achtereenvolgens hebben de ministers Borst-Eilers en Klink veel werk gemaakt van het toezicht op de handhaving van het wettelijk rookverbod op de werkplek. De vormgeving van het toezicht op de handhaving van het wettelijk rookverbod op de werkplek is zorgvuldig met argumenten onderbouwd door minister Borst-Eilers en lijkt logisch en niet onrechtvaardig. Minister Klink heeft bij het ontwerp van het wettelijk rookverbod in de horeca terdege rekening gehouden met de handhaafbaarheid. Verder heeft hij voortgebouwd op de vormgeving van het toezicht op de handhaving, zoals die is ingevoerd door minister Borst-Eilers. Bij de uitvoering van het toezicht op de handhaving heeft minister Klink rekening gehouden met een periode van gewenning, waarin in eerste instantie alleen waarschuwingen werden gegeven en nog geen boetes uitgedeeld. Wat betreft (het toezicht op) de handhaving van het wettelijk rookverbod heeft minister Klink een grote inspanning geleverd en kan hem m.i. geen onachtzaamheid of nalatigheid worden verweten.

In de zomer van 2009 ziet minister Klink zich gedwongen om het toezicht op de handhaving van het wettelijk rookverbod in de horeca zonder personeel tijdelijk op te schorten, naar aanleiding van uitspraken van de gerechtshoven in Den Bosch en Leeuwarden. Hierdoor ontstond direct een terugval in de naleving van het rookverbod in cafés. Minister Klink tracht de (vermeende) problemen met de legitimiteit van de handhaving van het wettelijk rookverbod in kleine cafés zonder personeel te repareren door in juli 2009 voor te stellen om de doelstelling van het wettelijk rookverbod uit te breiden met de bescherming van bezoekers in de horeca tegen passief roken.

In november 2010 schrijft minister Schippers aan de Tweede Kamer dat zij voornemens is om de kleine cafés zonder personeel uit te zonderen van het rookverbod in de horeca. In december 2010 trekt zij het ontwerpbesluit van minister Klink, ter verbreding van de doelstelling van het wettelijk rookverbod in de horeca, in. In het voorjaar van 2011 blijkt dat de aankondiging dat kleine cafés zonder personeel van het wettelijk rookverbod zullen worden uitgezonderd, heeft geleid tot een zeer grote terugval in de naleving van het rookverbod in cafés zonder en met personeel. M.i. had minister Schippers deze terugval, gezien de voorgeschiedenis van de handhaving van het rookverbod in de horeca, kunnen en moeten voorzien. In februari 2011 stelt minister Schippers voor om het toezicht op de naleving van het rookverbod in cafés met personeel te verscherpen. Minister Schippers is m.i. in ieder geval over de periode november 2010 t/m februari 2011 onachtzaam en nalatig geweest m.b.t. (het toezicht op) de handhaving van het wettelijk rookverbod in de horeca. De wijze waarop minister Schippers haar voorstel, tot uitzondering van kleine cafés zonder personeel van het wettelijk rookverbod in de horeca, heeft geïmplementeerd, is m.i. vanuit het perspectief van 'handhaafbaarheid' niet te rechtvaardigen. Een extra inspanning m.b.t. (het toezicht op) de handhaving van het rookverbod in cafés met personeel was aangewezen geweest.

13.4.4 Gevolgen van de beoordeling voor het model (criterium handhaafbaarheid)

De beoordeling van het wettelijk rookverbod in de horeca aan de hand van het model is de aanleiding geweest om een afzonderlijk criterium 'handhaafbaarheid' in het model op te nemen. Voorafgaand aan de daadwerkelijke beoordeling van het wettelijk rookverbod in de horeca aan de hand van het criterium 'handhaafbaarheid', heb ik het criterium 'handhaafbaarheid' als volgt geoperationaliseerd. Het criterium 'handhaafbaarheid' betreft zowel de 'handhaving' van de preventiemaatregel, als het 'toezicht op de handhaving'. In de praktijk wordt vaak geen onderscheid gemaakt tussen 'handhaving' en 'toezicht op de handhaving'. Als dit onderscheid niet behulpzaam is bij de beoordeling van de preventiemaatregel aan de hand van het model, hoeft dit onderscheid niet te worden gemaakt en vervalt de term 'toezicht op de handhaving'.

De wijze van beoordeling behorend bij het criterium 'handhaafbaarheid' is vergelijkbaar met de wijze van beoordeling behorend bij de overige criteria van de 3e zeef (implementatie) van het model. Uitgangspunten voor de wijze van beoordeling zijn: redelijkheid, billijkheid, geen onachtzaamheid, geen nalatigheid.

Naar aanleiding van de daadwerkelijke beoordeling van het wettelijk rookverbod in de horeca aan de hand van het criterium handhaafbaarheid, heb ik de begrippen 'sociale controle' en 'gedogen' (gedoogbeleid) als aandachtspunten bij de beoordeling van de preventiemaatregel aan de hand van het criterium 'handhaafbaarheid'.

13.5 Vijftiende criterium: controleerbaarheid

13.5.1 Operationalisering en wijze van beoordeling

Voordat de preventiemaatregel wordt geïmplementeerd, is vaak niet met zekerheid te voorspellen hoe de 'effecten en neveneffecten' (kosten-batenverhouding en rechtvaardigheid) en 'implementatie' (draagvlak, handhaving, uitvoerbaarheid) zich zullen ontwikkelen. Als ontwikkelingen afwijken van de verwachtingen, moet tijdig kunnen worden bijgestuurd. Dit is des te belangrijker naarmate de negatieve neveneffecten van de preventiemaatregel groter (kunnen) zijn. Daarom is het noodzakelijk de 'effecten en neveneffecten' en 'implementatie' van de preventiemaatregel te monitoren en/of (periodiek) te evalueren.

Beoordeeld wordt of de wijze waarop de 'effecten en neveneffecten' en 'implementatie' van de preventiemaatregel worden gemonitord en/of geëvalueerd, niet onredelijk of onbillijk is. Tevens wordt beoordeeld of men zich voldoende heeft ingezet om de 'effecten en neveneffecten' en implementatie van de preventiemaatregel te monitoren en/of evalueren. Is er op dit punt geen sprake van onachtzaamheid (men heeft risico's over het hoofd gezien) of nalatigheid (men heeft nagelaten om risico's die wel bekend waren goed in de gaten te houden)?

13.5.2 Beoordeling van casus 'wettelijk rookverbod in horeca' aan de hand van criterium 'controleerbaarheid'

Ik begin de beoordeling met het geven van een overzicht van citaten die te maken hebben met het criterium 'controleerbaarheid'. Vervolgens zal ik (mede) aan de hand van deze citaten de gerechtvaardigdheid van de implementatie van de preventiemaatregel aan de hand van het criterium 'controleerbaarheid' beoordelen.

In 1996 vond een algemeen overleg plaats tussen de vaste commissies voor VWS en EZ van de Tweede Kamer en de ministers Borst-Eilers van VWS en Wijers van EZ over het tabaksontmoedigingsbeleid[49]:

> *"Mevrouw Van Blerck-Woerdman (VVD) …Effectiviteit en handhaafbaarheid zijn volgens de brief belangrijke criteria geweest bij het voorstellen van maatregelen en zij was het daarmee wel eens, maar zou daarnaast ook nog het criterium van de toetsbaarheid willen noemen, dus de vraag of gemeten kan worden of het doel van de diverse maatregelen in de praktijk wordt bereikt."* (p.7)

In 2000 schrijft Minister Borst-Eilers aan de Tweede Kamer[50]:

> *"…vragen deze leden of de regering bereid is na een aantal jaren een evaluatie van de feitelijke effecten in het vooruitzicht te stellen. Ik ben hiertoe gaarne bereid en vind dit eerlijk gezegd niet meer dan vanzelfsprekend in het kader van de continue ontwikkeling en beoogde intensivering van het tabaksontmoedigingsbeleid, waarbij voortdurend terugkoppeling van de effecten van maatregelen geschiedt. In concreto stel ik mij voor om binnen vier jaar na de inwerkingtreding van dit wetsvoorstel een evaluatie van de effecten van de genomen maatregelen te plegen en deze aan de Tweede Kamer voor te leggen."* (p.61)

In april 2005 informeert minister Hoogervorst de Tweede Kamer in een brief over de globale opzet van de evaluatie van het stappenplan en het daarbij horende tijdpad[51]:

> *"Ik ben met Koninklijk Horeca Nederland (KHN) overeengekomen dat de monitoring van het stappenplan gezamenlijk zal worden uitgevoerd. Daartoe zal gezamenlijk een onafhankelijk onderzoeksbureau worden geselecteerd dat in overleg met KHN en VWS de onderzoeksopzet en onderzoeksmethode verder zal vormgeven.*
>
> *Het stappenplan beslaat een periode van vier jaar (2005, 2006, 2007 en 2008) en bevat voor elk jaar concrete streefcijfers. Na afloop van elk jaar zal de voortgang van de invoering van de afgesproken maatregelen worden gemeten. Er zal dus over elk jaar een aparte voortgangsrapportage verschijnen waarin wordt teruggekeken op het voorgaande jaar of de voorgaande jaren. Aan het eind van de totale periode zal een eindevaluatie worden opgesteld."* (p.1)
>
> *"Ik zal de jaarlijkse voortgangsrapportages naar de Tweede Kamer sturen zodat u goed op de hoogte bent van de stand van de zaken met betrekking tot de invoering van het stappenplan rookvrije horeca."* (p.2)

49. TK, 1996–1997, 24743, nr.3.
50. TK, 1999-2000, 26472, nr.6.
51. TK, 2004–2005, 29800 XVI, nr.162.

Minister Hoogervorst stelt dat hij onderzoek zal laten uitvoeren door een onafhankelijk onderzoeksbureau. De onafhankelijkheid c.q. objectiviteit van het onderzoek en de evaluatie is belangrijk met het oog op de objectiviteit van de beschikbare 'stuurinformatie'. Ik zal het aspect van onafhankelijkheid van de monitoring en/of evaluatie van de uitvoering daarom als aandachtspunt aan het criterium 'controleerbaarheid' toevoegen.

In december 2008 informeert minister Klink de Tweede Kamer in een brief over de praktische implicaties van de invoering van de horecavrije horeca per 1 juli 2008[52]:

> *"Tijdens het spoeddebat van 7 oktober 2008 … heb ik toegezegd u in december te zullen informeren over de praktische implicaties bij de invoering van de rookvrije horeca. Met deze brief doe ik die toezegging gestand. Ik wil hierbij nadrukken dat dit het voorlopige beeld tot nu toe is. Pas in de loop van 2009 zal een meer definitief beeld gevormd kunnen worden van de ontwikkelingen in de horeca. … Ik zal permanent de ontwikkelingen volgen en daar de Kamer, als er aanleiding toe is, over informeren. Ook ben ik, zoals ik u eerder heb gemeld, van plan de invoering van de rookvrije horeca goed te evalueren. Een dergelijke evaluatie heeft wat mij betreft in ieder geval betrekking op de ontwikkelingen in het eerste jaar na de invoering.*
>
> *In deze brief ga ik achtereenvolgens in op op dit moment bekende gegevens over:*
> 1. *rookvrije werkplek in het belang van de gezondheid van werknemers;*
> 2. *luchtkwaliteit in de horeca, blootstelling en hartaanvallen;*
> 3. *handhaving en naleving;*
> 4. *horecabezoeken, omzetcijfers en faillissementen;*
> 5. *bouwtechnische aanpassingen en oppervlakte-eisen;*
> 6. *vergunningen en overlast en;*
> 7. *verzoeken om uitzondering, subsidie of compensatie." (p.1)*
>
> *"… Dit alles neemt niet weg dat ik de ontwikkelingen rond de rookvrije horeca nauwgezet zal blijven monitoren en uw Kamer daarover zal blijven informeren."* (p.15)

In oktober 2009 verschijnt het onderzoeksrapport 'Even uitblazen. Eén jaar rookvrije horeca' van Regioplan Beleidsonderzoek (Regioplan, 2009). Dit rapport is een review van de onderzoeksrapporten van andere organisaties (TNS NIPO, INTRAVAL, Synovate, Foodstep, RIVM, enz.) over de gevolgen van het rookverbod in de horeca. Het ministerie van VWS heeft Regioplan opdracht gegeven tot dit reviewonderzoek.

---

52. TK, 2008–2009, 22894, nr.206.

Voor zover ik het kan beoordelen is minister Klink op basis van het rapport van TNS NIPO (2009) in zijn brief d.d. 1 december 2009 tot de volgende conclusie gekomen:

> *"Net als in mijn brief van 16 december 2008 is mijn conclusie dat er vooralsnog geen grond is om vast te stellen dat de rookvrije horeca voor bepaalde sectoren of (sub) groepen buiten proportioneel zou uitwerken."*

Deze conclusie van minister Klink is m.i. meer gebaseerd op het ontbreken van gedetailleerde onderzoekscijfers, waardoor hierover geen conclusies kunnen worden getrokken ('*er is geen grond om vast te stellen*'), dan op duidelijke aanwijzingen dat kleine cafés zonder personeel niet buitenproportioneel door het rookverbod worden geraakt. Zie voor een nadere toelichting bijlage 10.

In februari 2011 informeert minister Schippers de Tweede Kamer in een brief over toezeggingen die zij heeft gedaan tijdens een AO (algemeen overleg) in januari 2011:

> *"Periodieke monitoring. Tijdens het AO heb ik toegezegd de Kamer tweemaal per jaar te informeren over de naleving van het rookverbod. De VWA rapporteert in juni en november aan VWS over de nalevingspercentages van de natte horeca, het aantal bedrijfsbezoeken en het aantal opgelegde maatregelen. Met deze informatie zal ik vervolgens de Tweede Kamer informeren."* (p.1-2)

13.5.3 Eindoordeel (criterium controleerbaarheid)

De achtereenvolgende ministers Borst-Eilers, Hoogervorst en Klink hebben zich ingezet om tot een goede monitoring en evaluatie van de wettelijke rookverboden te komen. Tijdens de bewindsperiode van minister Schippers loopt de monitoring van de naleving en handhaving, zoals die is opgezet door minister Klink, gewoon door. Over het algemeen zijn de monitoring en evaluatie van de effecten en neveneffecten en de implementatie van het wettelijk rookverbod m.i. van voldoende omvang en kwaliteit. Met het oog op de objectiviteit van de onderzoeksresultaten is het van belang dat is gekozen voor onderzoek door onafhankelijke onderzoeksbureaus. Over het algemeen is m.b.t. tot de controle van de implementatie dus geen sprake van onachtzaamheid of nalatigheid.

Minister Klink heeft wel een verdiepend onderzoek laten uitvoeren naar de mogelijkheid van ventilatiesystemen als alternatief voor het rookverbod. Hij heeft echter verzuimd om een verdiepend onderzoek te laten uitvoeren naar de vermeende negatieve effecten van het wettelijk rookverbod in de horeca op de omzet van kleine cafés (zonder personeel). Aan deze keuze van minister Klink zou de politieke overweging ten grondslag kunnen liggen dat het beleidsmatig wel welkom is,

als uit onderzoek blijkt dat ventilatiesystemen kunnen dienen als alternatief voor het rookverbod, maar dat het beleidsmatig niet welkom is, als uit onderzoek blijkt dat de omzet van kleine cafés (zonder personeel) door het rookverbod onevenredig hard terugloopt. Dat minister Klink geen verdiepend onderzoek heeft laten uitvoeren naar de economische gevolgen van het rookverbod voor kleine cafés zonder personeel, is op zijn minst onachtzaam. Vanuit wetenschappelijk perspectief is het niet uitvoeren van een dergelijk verdiepend onderzoek m.i. onredelijk en ten opzichte van de kleine cafés (zonder personeel) is het onbillijk.

13.5.4 Gevolgen van de beoordeling voor het model (criterium controleerbaarheid)

De beoordeling van het wettelijk rookverbod in de horeca aan de hand van het model is de aanleiding geweest om een afzonderlijk criterium ‘controleerbaarheid’ in het model op te nemen. Voorafgaand aan de daadwerkelijke beoordeling van het wettelijk rookverbod in de horeca aan de hand van het criterium ‘controleerbaarheid’, heb ik het criterium ‘controleerbaarheid’ als volgt geoperationaliseerd. Het criterium ‘controleerbaarheid’ betreft de monitoring en (periodieke) evaluatie van de ‘effecten en neveneffecten’ en ‘implementatie’ van de preventiemaatregel. De wijze van beoordeling behorend bij het criterium ‘controleerbaarheid’ is vergelijkbaar met de wijze van beoordeling behorend bij de overige criteria van de 3e zeef (implementatie) van het model. Uitgangspunten voor de wijze van beoordeling zijn: redelijkheid, billijkheid, geen onachtzaamheid en geen nalatigheid.

De onafhankelijkheid c.q. objectiviteit van de monitoring en/of evaluatie van de uitvoering van de preventiemaatregel is belangrijk met het oog op de objectiviteit van de beschikbare ‘stuurinformatie’. Daarom heb ik het aspect van onafhankelijkheid van de monitoring en/of evaluatie als aandachtspunt aan het criterium ‘controleerbaarheid’ toegevoegd.

13.6 Zestiende criterium: uitvoerbaarheid

13.6.1 Operationalisering en wijze van beoordeling

Het criterium ‘uitvoerbaarheid’ betreft het laatste criterium van het model. Op het moment dat de preventiemaatregel wordt beoordeeld aan de hand van het criterium ‘uitvoerbaarheid’, zijn bij de beoordeling van de preventiemaatregel aan de hand van de voorafgaande criteria al veel uitvoeringsaspecten aan de orde gekomen. Eigenlijk hebben alle criteria van de 2e en 3e zeef van het model betrekking

op het ontwerp en de uitvoering van de preventiemaatregel. Onderdeel van de beoordeling bij ieder criterium van de 2e en 3e zeef is immers de vraag of een ander ontwerp of andere uitvoering van de preventiemaatregel leiden tot een betere kosten-batenverhouding of tot meer rechtvaardigheid.

De beoordeling van de preventiemaatregel aan de hand van het criterium 'uitvoerbaarheid' beperk ik tot de uitvoeringsaspecten die nog niet aan de orde zijn geweest. De beoordeling van de preventiemaatregel aan de hand van het criterium 'uitvoerbaarheid' beperkt zich daarom tot de vraag of er voldoende mensen en middelen beschikbaar zijn om de implementatie van de preventiemaatregel te rechtvaardigen. Het gaat hierbij zowel om de haalbaarheid als houdbaarheid van de implementatie.

Beoordeeld wordt:
- of er voldoende mensen en middelen beschikbaar zijn gesteld om een adequate uitvoering van de preventiemaatregel haalbaar en houdbaar (in de tijd) te maken;
- of niet onredelijk weinig of onbillijk weinig mensen en middelen voor de uitvoering van de preventiemaatregel beschikbaar zijn gesteld;
- of men zich voldoende heeft ingezet (geen onachtzaamheid en geen nalatigheid) om voldoende mensen en middelen voor de uitvoering beschikbaar te krijgen.

13.6.2 Beoordeling van casus 'wettelijk rookverbod in horeca' aan de hand van criterium 'uitvoerbaarheid'

Ik begin de beoordeling met het geven van enkele citaten die te maken hebben met het criterium 'uitvoerbaarheid'. Vervolgens zal ik (mede) aan de hand van deze citaten de implementatie van de preventiemaatregel aan de hand van het criterium 'uitvoerbaarheid' beoordelen.

In mei 2004 schrijft minister Hoogervorst in een brief aan de Tweede Kamer, naar aanleiding van het onderzoeksrapport 'Reductie van blootstelling aan omgevingstabaksrook in de horeca door ventilatie en luchtreiniging' van TNO en RIVM[53]:

> *"… Daarenboven brengt de introductie van de meest effectieve ventilatietechnieken voor de horeca <u>prohibitieve investeringen</u> met zich mee. Gezien deze gezondheidskundige overwegingen en bovenstaande praktische voorbehouden kom ik tot het oor-*

53. TK, 2003–2004, 29200 XVI, nr.233.

deel dat ventilatie niet gezien moet worden als een effectieve oplossing om werknemers en bezoekers te beschermen tegen omgevingstabaksrook." (p.2)

Op 19 januari 2011 vindt een overleg plaats tussen de vaste commissie van de Tweede Kamer voor VWS en minister Schippers over (de gevolgen van) haar voorstel tot uitzondering van kleine cafés zonder personeel van het wettelijk rookverbod in de horeca[54]:

> *"Minister Schippers: … Ik wil dat de handhaving veel meer resultaat oplevert. Daar moet dus een tandje bij; … Ik zit in een situatie met een beperkt aantal handhavers, namelijk ongeveer 200 inspecteurs. Bovendien zit ik in een tijd van inkrimping: er moeten ambtenaren uit. Is uitbreiding hiervan dan aan de orde? Ik heb daar geen geld voor. Ik wil ook niet dat inspecteurs die nu toezien op de voedselveiligheid, daarvan af worden gehaald. Wel zijn er, met de uitzondering van deze kleine cafés zonder personeel, relatief meer inspecteurs voor de rest. Dan de route via het Openbaar Ministerie, die door enkele woordvoerders is aangegeven: halsstarrige overtreders voor de rechter dagen. Dat is in het recente verleden in een beperkt aantal gevallen wel degelijk gebeurd, maar dit is een duur, langdurig en arbeidsintensief traject met een geringe opbrengst. … Eigenlijk wil ik een veel eenvoudiger aanpassing om de effectiviteit van de handhaving fors te verhogen. Ten eerste wil ik de boetes fors verhogen. … Ik zoek naar de grenzen van wat ik zelf kan bereiken in een verhoging van die boete."* (p.20-21)

Op 26 januari 2011 dient de PvdA een motie in, omdat zij van opvatting is dat de nVWA, sinds de (aangekondigde) versoepeling van het rookverbod door minister Schippers, onvoldoende inspecteurs beschikbaar heeft voor een adequaat toezicht op de handhaving van het wettelijk rookverbod in de horeca[55]:

- *"overwegende dat het onderscheid in horeca waar wel en niet gerookt mag worden extra bureaucratie veroorzaakt, en toezicht en handhaving daardoor extra mensen vergen;*
- *constaterende dat sinds de aankondiging van versoepeling van het rookverbod in de horeca er momenteel in 51% van de kroegen wordt gerookt;*
- *constaterende dat de nVWA veel te weinig mensen heeft om de controle en handhaving uit te voeren, aangezien er in totaal 40 teams van twee mensen zijn die alle 17.000 kroegen in Nederland moeten controleren;*
- *constaterende dat de regering geen extra controleurs inzet en geen concreet voorstel heeft gedaan voor hogere boetes;*
- *constaterende dat alleen een hoge pakkans in samenhang met hoge boetes afschrikwekkend zal werken;*

54. TK, 2010–2011, 32011, nr.15.
55. TK, 2010- 2011, 44, nr.6.

– *verzoekt de regering, het wettelijk rookverbod te handhaven door voldoende toezicht en handhaving en hogere boetes te garanderen, en hiermee het aantal overtredingen zo veel mogelijk terug te brengen;"* (p.2)

Minister Schippers stelt in hetzelfde debat:

> *"In het verzoek aan de regering staat dat er voldoende toezicht moet zijn. Misschien vindt mevrouw Bouwmeester het niet voldoende, maar ik vind dat wel. … Daar komt bij dat het altijd gemakkelijk is om te zeggen dat het aantal handhavers moet worden uitgebreid. Wij hebben echter ook een huishoudboekje op orde te brengen. Daarvoor moeten heel serieuze en moeilijke keuzes worden gemaakt. Ik maak die keuzes blijkbaar anders dan de PvdA-fractie dat doet."* (p.5)

Analyse van de uitvoerbaarheid

In het politieke debat over onvoldoende beschikbaarheid van mensen of (financiele) middelen voor uitvoering van het wettelijk rookverbod in de horeca zijn twee onderwerpen aan de orde geweest:

- de (te) hoge kosten van ventilatiesystemen als alternatief voor het rookverbod;
- een te gering aantal inspecteurs bij de nVWA voor toezicht op de handhaving van het rookverbod.

Minister Hoogervorst heeft gesteld dat een effectief ventilatiesysteem met name voor kleine cafés *"prohibitieve investeringen"* met zich brengt. Er lijkt politiek consensus te bestaan dat dit het geval is. Nadat wetenschappelijk onderzoek keer op keer heeft aangetoond dat ventilatiesystemen werknemers in de horeca geen effectieve bescherming tegen passief roken bieden, zijn de hoge kosten van effectieve ventilatiesystemen geen issue meer in het politieke debat.

Over het te geringe aantal inspecteurs bij de nVWA voor het toezicht op de handhaving van het rookverbod verschillen de politieke partijen van opvatting. M.i. hebben de problemen met de naleving en handhaving van het rookverbod in cafés meer te maken met het gebrek aan draagvlak voor het rookverbod en de aangekondigde versoepeling van het rookverbod door minister Schippers dan met een te gering aantal inspecteurs. De keuze van minister Schippers om niet het aantal inspecteurs bij de nVWA maar de boetes te verhogen, is m.i. niet evident onredelijk of onbillijk.

13.6.3 Eindoordeel (criterium uitvoerbaarheid)

In het politieke debat is de handhaafbaarheid van het rookverbod in cafés en discotheken een groot issue (geweest). Uit de door mij bestudeerde parlementaire documenten blijkt dat dit meer te maken heeft met:

– het gebrek aan draagvlak voor het rookverbod (vanwege vermeende negatieve gevolgen voor de omzet en 'ongezelligheid'),
– de problemen met de legitimiteit van het rookverbod (rechtszaken tegen het rookverbod in kleine cafés zonder personeel), en
– de uitzonderingspositie van kleine cafés zonder personeel (tijdelijk ingesteld door minister Klink en permanent gemaakt door minister Schippers).

Met de beschikbaarheid van te weinig mensen en middelen hebben de handhavingsproblemen weinig te maken. In 2002 heeft minister Borst-Eilers gesteld [56]:

> "*Wetgeving is en blijft ook een kosteneffectief instrument binnen het totale samenhangende tabaksontmoedigingsbeleid van de regering.*" (p.10).

Waarschijnlijk heeft minister Borst-Eilers hierin gelijk. Op basis van de voorgaande overwegingen kom ik tot de conclusie dat er geen reden is om aan te nemen dat een adequate uitvoering van het rookverbod niet 'haalbaar en houdbaar' is, omdat er onvoldoende mensen of middelen voor de uitvoering beschikbaar zijn.

Op basis van het bovenstaande eindoordeel kom ik tot de conclusie dat de benaming 'uitvoerbaarheid' van het laatste criterium van het model moet worden aangepast. Er zijn immers wel problemen met de haalbaarheid en houdbaarheid van het rookverbod in cafés en discotheken, maar deze problemen vinden m.i. niet hun oorzaak in een gebrek aan mensen en middelen voor uitvoering (van café- en discotheekhouders worden geen 'prohibitieve investeringen' gevraagd) of de handhaving. De betaalbaarheid (de betaling van mensen en middelen) van de uitvoering van het rookverbod in de horeca is haalbaar en houdbaar. Daarom kies ik ervoor om de benaming van het laatste criterium van het model te veranderen. De nieuwe benaming wordt 'betaalbaarheid'. Deze nieuwe benaming doet recht aan mijn opmerking aan het begin van deze subparagraaf (§13.6.1): "Op het moment dat de preventiemaatregel wordt beoordeeld aan de hand van het criterium 'uitvoerbaarheid' zijn bij de beoordeling van de preventiemaatregel aan de hand van de voorafgaande criteria al veel uitvoeringsaspecten aan de orde gekomen."

56. EK, 2001–2002, 26472, nr.59c.

Bij nader inzien stuit ook de benaming 'betaalbaarheid' echter op problemen. Soms is er voldoende geld en dus geen betaalbaarheidsprobleem, maar zijn er toch onvoldoende geschikte mensen (bijvoorbeeld mensen met een gespecialiseerde opleiding) of middelen (bijvoorbeeld gespecialiseerde apparatuur of voldoende ruimte) beschikbaar voor uitvoering van de preventiemaatregel. Uiteindelijk kies ik er daarom voor om het laatste criterium van het model 'uitvoeringscapaciteit' te noemen. Het criterium uitvoeringscapaciteit betreft de vraag of er voldoende mensen en middelen beschikbaar zijn om de uitvoering van de preventiemaatregel haalbaar en houdbaar te maken.

Zoals ik hierboven heb toegelicht is het in de praktijk mogelijk dat de uitvoeringscapaciteit in principe voldoende is, maar dat er toch problemen zijn met de haalbaarheid en houdbaarheid van de uitvoering, die bijvoorbeeld te maken kunnen hebben met de afwezigheid van 'spontane' naleving van de preventiemaatregel, veroorzaakt door een gebrek aan draagvlak.

13.6.4 Gevolgen van de beoordeling voor het model (criterium uitvoerbaarheid)

De beoordeling van het wettelijk rookverbod in de horeca aan de hand van het model is de aanleiding geweest om de naamgeving van het criterium 'uitvoerbaarheid' te veranderen. De nieuwe naamgeving luidt 'uitvoeringscapaciteit'. Met deze nieuwe naamgeving wordt tot uitdrukking gebracht dat het laatste criterium van het model niet alle uitvoeringsaspecten van de preventiemaatregel betreft, maar zich beperkt tot de vraag of er (nu en in de toekomst) voldoende mensen en middelen beschikbaar zijn om de preventiemaatregel uit te voeren.

13.7 Eindoordeel over de 3e zeef: implementatie

13.7.1 Beoordeling van de implementatie van het wettelijk rookverbod in de horeca

Het wettelijk rookverbod in de horeca kan de 3e zeef 'implementatie' van het beoordelingsmodel niet passeren. De wijze waarop is geïmplementeerd is op onderdelen onredelijk (onlogisch) en onbillijk (onrechtvaardig).

Minister Klink heeft bij de implementatie van het wettelijk rookverbod in de horeca m.i. teveel steken laten vallen om de manier waarop is geïmplementeerd gerechtvaardigd te kunnen noemen. Enerzijds heeft hij te weinig oog gehad voor

behoud en versterking van het draagvlak voor het wettelijk rookverbod in de horeca bij KHN en bij horecaondernemers. Zie voor een toelichting onder het kopje ‘eindoordeel’ bij de criteria ‘timing’ en ‘draagvlak’. Anderzijds heeft hij te weinig oog gehad voor de vermeende negatieve (financiële) gevolgen van de implementatie voor kleine cafés zonder personeel. Zie voor een toelichting onder het kopje ‘eindoordeel’ bij de criteria ‘flankerend beleid’ en ‘controleerbaarheid’.

Minister Schippers is m.i. bij de versoepeling van het wettelijk rookverbod in de horeca – de vrijstelling van kleine cafés zonder personeel van dit rookverbod – onachtzaam en nalatig geweest m.b.t. de gevolgen voor de handhaving van het rookverbod. Zie voor een toelichting onder het kopje ‘eindoordeel’ bij het criterium ‘handhaafbaarheid’.

Dat minister Klink te weinig oog heeft gehad voor de vermeende negatieve (financiële) gevolgen van de implementatie voor kleine cafés zonder personeel, acht ik van de bovengenoemde problemen het ernstigst, omdat bepaalde horecaondernemers hierdoor mogelijk ernstig in de (financiële) problemen zijn gekomen, zonder dat hier oog voor was. Door de genoemde onachtzaamheid en nalatigheid van minister Schippers is naar het zich laat aanzien niemand direct ernstig in de problemen gekomen.

Dat de manier waarop minister Schippers vormgeeft aan de handhaving van het wettelijk rookverbod in de cafés suboptimaal is, betekent niet dat de toepassing van het rookverbod in cafés dan maar moet worden stopgezet. In tegendeel, want ondanks de suboptimale handhaving zijn de resultaten van het wettelijk rookverbod in cafés nog steeds zeer de moeite waard.

Een suboptimale handhaving van het wettelijk rookverbod in cafés met personeel leidt echter wel:
- tot een suboptimale effectiviteit (2e zeef),
- tot rechtsongelijkheid tussen de cafés met personeel (2e zeef),
- tot ondermijning van het draagvlak voor het rookverbod in cafés met personeel (3e zeef), en
- mogelijk tot uitholling van de geloofwaardigheid van wetgeving in het algemeen (2e zeef, negatief neveneffect).

Daarom mag van minister Schippers een grote inspanning worden gevraagd om de handhaving te verbeteren.

Op basis van het bovenstaande kom ik tot de volgende conclusie over het model. Als een preventiemaatregel de 3e zeef van het model niet passeert, betekent dat niet automatisch dat de implementatie of toepassing van de preventiemaatregel moet worden stopgezet. Wat dan wel de consequentie is als een preventiemaatregel de 3e zeef van het model niet passeert, bespreek ik in de volgende subparagraaf (§13.7.2).

13.7.2 *Gevolgen van de beoordeling voor het model (3e zeef: implementatie)*

De beoordeling van het wettelijk rookverbod in de horeca aan de hand van de criteria van de 3e zeef van het model heeft geleid tot aanpassingen aan en conclusies over het model. Hieronder worden deze besproken.

Mogelijke rol voor procedurele rechtvaardigheid
De beoordeling van het wettelijk rookverbod in de horeca aan de hand van de criteria 'timing' (§13.1.4) en 'draagvlak' (§13.2.4) bevestigt dat procedurele aspecten relevant kunnen zijn voor de beoordeling van de gerechtvaardigdheid van een leefstijlbeïnvloedende preventiemaatregel aan de hand van het model. (Zie ook §12.4.4).

Aanvulling op operationalisering van draagvlak
De aansluiting van de preventiemaatregel op het beleid van stakeholders kan van belang zijn voor het draagvlak van de preventiemaatregel. Daarom heb ik dit aspect toegevoegd aan het criterium draagvlak van het model.

Introductie van optimalisatiebeginsel
De operationalisering van het criterium flankerend beleid heeft geleid tot introductie van het optimalisatiebeginsel als uitgangspunt voor de beoordeling van de gerechtvaardigdheid van de implementatie van de preventiemaatregel (3e zeef van het model). Het optimalisatiebeginsel betreft de vraag of er bij de implementatie van de preventiemaatregel geen sprake is geweest van onachtzaamheid (onoplettendheid; onvoldoende aandacht besteden aan problemen) of nalatigheid (verzuim; men zet zich onvoldoende in om tot optimale implementatie te komen). Door bij het beoordelen van eventuele nalatigheid bij de implementatie uit te gaan van de gegeven mogelijkheden, wordt voorkomen dat de beoordeling van de implementatie perfectionistisch van karakter wordt. (Zie §13.3.1)

Aanvulling op operationalisering van flankerend beleid
In de praktijk blijkt het er niet alleen om te gaan dat flankerend beleid de preventiemaatregel ondersteunt, maar ook dat er geen beleid is dat strijdig is met de pre-

ventiemaatregel. Het aandachtspunt of er geen ander beleid is dat strijdig is met de doelstelling van de preventiemaatregel, heb ik daarom toegevoegd aan de operationalisering van het criterium 'flankerend beleid'. (Zie §13.3.2)

Aanvulling op operationalisering van handhaafbaarheid
Naar aanleiding van de daadwerkelijke beoordeling van het wettelijk rookverbod in de horeca aan de hand van het criterium handhaafbaarheid, heb ik de begrippen 'sociale controle' en 'gedogen' (gedoogbeleid) als aandachtspunten toegevoegd aan de 'operationalisering en wijze van beoordeling' van het criterium 'handhaafbaarheid'. (Zie §13.4.2)

Verandering naamgeving van criterium uitvoerbaarheid
De beoordeling van het rookverbod aan de hand van het criterium 'uitvoerbaarheid' is aanleiding geweest tot een verandering van de naamgeving van dit criterium. De nieuwe naamgeving luidt 'uitvoeringscapaciteit'. Met deze nieuwe naamgeving wordt tot uitdrukking gebracht dat het laatste criterium van het model niet alle uitvoeringsaspecten van de preventiemaatregel betreft, maar zich beperkt tot de vraag of er voldoende mensen en middelen beschikbaar zijn om de preventiemaatregel uit te voeren. (Zie §13.6.1)

Consequentie van het niet passeren van de 3e zeef van het model
Als (een onderdeel van) een preventiemaatregel de 1e of 2e zeef van het model niet kan passeren, mag (dat onderdeel van) de maatregel niet (meer) worden uitgevoerd. Wat de consequentie is als de preventiemaatregel de 3e zeef van het model niet kan passeren, is minder duidelijk. Het blijkt immers mogelijk dat de effecten en neveneffecten van de preventiemaatregel (2e zeef), ondanks een suboptimale implementatie (3e zeef), nog steeds zeer de moeite waard zijn (dit geldt bijvoorbeeld voor het wettelijk rookverbod in de cafés). In dat geval voert het te ver om de uitvoering van (dat onderdeel van) de preventiemaatregel te verhinderen. Soms is het beter om (onderdelen van) een preventiemaatregel suboptimaal te uit te voeren dan niet uit te voeren ('beter een half ei dan en lege dop'). Mijn conclusie is dat per situatie dien te worden beoordeeld welke consequenties moeten worden verbonden aan het niet passeren van de 3e zeef van het model. Uiteindelijk wordt dit bepaald door de invloed van de suboptimale implementatie (3e zeef) op de 'effecten en neveneffecten' van de preventiemaatregel (2e zeef).

Invloed van de implementatie op effecten en neveneffecten (2e zeef)
Een ingewikkeld gegeven bij de beoordeling van de gerechtvaardigdheid van leefstijlbeïnvloedende preventiemaatregelen is dat de wijze waarop de preventiemaat-

regel wordt geïmplementeerd (3e zeef) van invloed is op de effecten en neveneffecten (2e zeef). Ter illustratie twee voorbeelden:

- voorlichting (criterium 'flankerend beleid', 3e zeef) over de preventiemaatregel kan de naleving en daarmee 'effectiviteit' (2e zeef) verbeteren;
- geld dat men uitgeeft aan de handhaving (criteria 'handhaafbaarheid' en 'uitvoeringscapaciteit', 3e zeef) van een preventiemaatregel is van invloed op de 'kosteneffectiviteit' (2e zeef) van de maatregel.

Daarom dient men in de beoordeling van de 'effecten en neveneffecten' (2e zeef) van de preventiemaatregel, de (verwachte) invloed van de implementatie te verdisconteren. Bij een preventiemaatregel die al wordt toegepast (zoals het rookverbod in de horeca) is de invloed van de implementatie 'automatisch' verdisconteerd in de effecten en neveneffecten die optreden. De 'effectiviteit' (2e zeef) wordt bijvoorbeeld in belangrijke mate bepaald door de naleving en de naleving wordt medebepaald door het draagvlak (12e criterium, 3e zeef) en door de handhaving (14e criterium, 3e zeef). In de effecten die optreden, is de invloed van het draagvlak en van de handhaving dus 'automatisch' verdisconteerd.

Beoordeling van de implementatie op zichzelf (3e zeef)

Bij de 3e zeef van het model wordt de implementatie op zichzelf beoordeeld: wat is de (beoogde) kwaliteit van de implementatie? In de praktijk blijkt het immers mogelijk dat de effecten en neveneffecten van een preventiemaatregel (2e zeef) te rechtvaardigen zijn, terwijl de implementatie (3e zeef) verwijtbaar suboptimaal verloopt. Ten behoeve van de 3e zeef is daarom het optimalisatiebeginsel (geen onachtzaamheid en geen nalatigheid) als uitgangspunt voor de beoordeling aan het model toegevoegd.

Retrospectieve en prospectieve beoordeling

Naar aanleiding van een van de bovenstaande alinea's '*invloed van de implementatie op de effecten en neveneffecten (2e zeef)'* werpt een van mijn begeleiders de vraag op hoe de effecten en neveneffecten van de preventiemaatregel beoordeeld moeten worden als de implementatie nog niet heeft plaatsgevonden. Naar aanleiding van deze vraag ben ik verder gaan nadenken over de (on)mogelijkheden van een retrospectieve en prospectieve beoordeling van de gerechtvaardigdheid van een preventiemaatregel aan de hand van het model. Hieronder werk ik dit uit.

Retrospectieve beoordeling

Bij een retrospectieve beoordeling wordt teruggekeken naar de effecten en neveneffecten (2e zeef) en naar de implementatie (3e zeef) van de preventiematregel, zoals die daadwerkelijk hebben plaatsgevonden. Een retrospectieve beoordeling

kan daarom alleen tijdens of na afloop van de implementatie van een preventiemaatregel plaatsvinden. Een retrospectieve beoordeling kan daarom ook een evaluatie van de gerechtvaardigdheid van de preventiemaatregel genoemd worden. In de effecten en neveneffecten (2e zeef) die zijn opgetreden, zit de invloed van de implementatie automatisch verwerkt.

De criteria van de 3e zeef (implementatie) van het model kunnen, ook na afloop van de implementatiefase, relevant zijn voor de beoordeling van de gerechtvaardigdheid van de preventiemaatregel. Ter illustratie een voorbeeld.

> Het wettelijk recht op een rookvrije werkplek is (met uitzondering van de horecasector) van kracht geworden op 1 januari 2004 (zie §10.3). Stel dat men de gerechtvaardigdheid van dit recht over de periode 2009 t/m 2011 zou willen evalueren. Bij de implementatie van dit recht is, voor zover mij bekend, de einddatum van de implementatiefase niet expliciet vastgesteld. Ten behoeve van dit voorbeeld acht ik het redelijk om te veronderstellen dat de implementatiefase voor 2009 (5 jaar na de invoeringsdatum) was afgerond. Bij de beoordeling van de gerechtvaardigdheid van het recht op een rookvrije werkplek over de periode 2009 t/m 2011 zijn de criteria 'draagvlak, flankerend beleid, handhaafbaarheid, controleerbaarheid en uitvoerbaarheid' uit de 3e zeef (implementatie) van het model waarschijnlijk nog steeds relevant. Het criterium 'timing' uit de 3e zeef is waarschijnlijk niet meer relevant. De feitelijk relevantie kan men vaststellen door de beoordeling daadwerkelijk uit te voeren.

Voor de naamgeving van de 3e zeef van het model is het begrip 'implementatie' dus eigenlijk te beperkt. De criteria uit de 3e zeef kunnen immers ook relevant zijn voor de beoordeling van de gerechtvaardigdheid van de preventiemaatregel na afloop van de implementatiefase. Daarom heb ik overwogen om de naamgeving van de 3e zeef te veranderen van 'implementatie' in 'toepassing in de praktijk'. Na ampele overweging heb ik besloten om deze naamsverandering niet door te voeren, omdat deze de begrijpelijkheid van het model niet ten goede komt. Daarom handhaaf ik toch het begrip 'implementatie' als naam voor de 3e zeef van het model. Daarbij plaats ik de kanttekening dat de criteria uit de 3e zeef ook na afloop van de implementatiefase relevant kunnen zijn voor de beoordeling van de gerechtvaardigdheid van een preventiemaatregel.

Prospectieve beoordeling

Bij een prospectieve beoordeling wordt vooruitgekeken naar de effecten en neveneffecten (2e zeef) en implementatie (3e zeef) van de preventiematregel, die nog moeten plaatsvinden. Een prospectieve beoordeling kan daarom ook een voorspelling van de gerechtvaardigdheid van de preventiemaatregel genoemd worden. Een prospectieve beoordeling kan alleen plaatsvinden op basis van aannames over de

‘effecten en neveneffecten’ en over de ‘implementatie’ van de preventiemaatregel. In het kader van een beoordeling van de gerechtvaardigdheid van een preventiemaatregel aan de hand van het model dienen deze aannames te worden geëxpliciteerd, zodat de redelijkheid en billijkheid (aannemelijkheid) ervan kan worden beoordeeld. Het werken met aannames biedt ook de mogelijkheid om te werken met scenario’s, waarbij de effecten en neveneffecten van de preventiemaatregel worden beoordeeld (voorspeld) aan de hand van verschillende implementatiescenario’s (bijvoorbeeld veel versus weinig inspanning op het gebied van ‘handhaving’). Ook is het mogelijk om via experimenten of pilotstudies, in een gecontroleerde en/of afgebakende omgeving, de ‘effecten en neveneffecten’ en ‘implementatie’ van een preventiemaatregel uit te proberen en te onderzoeken. Desgewenst kan men binnen een of meer experimenten of pilotstudies het ontwerp van de preventiemaatregel variëren.

Samenvoegen van beoordelingscriteria

Een van mijn begeleiders merkt op het aantal van 6 criteria voor de 3e zeef van het model aan de grote kant te vinden. Hiervoor heeft hij de volgende argumenten:

- Een groot aantal criteria vermindert de praktische toepasbaarheid van het model. Voor een groot aantal criteria moet alleen worden gekozen, als dat inhoudelijk een belangrijke meerwaarde heeft. Aan die belangrijke meerwaarde twijfelt hij bij de criteria ‘timing’ en controleerbaarheid’. Het ‘soortelijk gewicht’ van deze criteria lijkt kleiner dan dat van de andere criteria van het model.
- De criteria ‘timing’ en draagvlak vertonen overlap. Diverse citaten die worden gebruikt bij het criterium ‘timing’ blijken ook van toepassing op het criterium ‘draagvlak’ en vice versa. De antwoorden op de vragen behorend bij ‘de criteria ‘timing’ en ‘draagvlak’ hangen onderling samen. Daarom moet worden overwogen deze criteria samen te voegen.
- Onterecht zou de indruk kunnen worden gewekt dat bij de beoordeling van de gerechtvaardigdheid van een preventiemaatregel de implementatie (3^{e} zeef, 6 criteria) belangrijker is dan de ‘effecten en neveneffecten’ (2e zeef, 4 criteria).
- Bij de ontwikkeling van het model is ernaar gestreefd dat het eerste criterium binnen een cluster voor de beoordeling minder afhankelijk is van niet-wetenschappelijk te bewijzen opvattingen dan het tweede criterium (zie §3.3). Het toevoegen van de criteria ‘handhaafbaarheid’ en ‘controleerbaarheid’ aan de 3e zeef (zie §10.4) lijkt deze volgorde te hebben verstoord.[57]

57. Bij de clusters ‘timing – draagvlak’ en ‘flankerend beleid – handhaafbaarheid’ heb ik deze volgorde inderdaad losgelaten ten behoeve van de inhoudelijk logische volgorde van de criteria. In §3.3 heb ik toegelicht waarom binnen het model de inhoudelijk logische volgorde van de criteria leidend is bij het bepalen van de volgorde van de criteria. Het streven, dat het eerste criterium binnen een cluster voor de

Ik kan mij vinden in deze argumenten. Aan de andere kant vind ik dat er om inhoudelijke redenen geen criteria uit de 3e zeef van het model geschrapt kunnen worden en dat de volgorde van alle criteria van het model inhoudelijk juist is. Dit betekent dat ik alleen opeenvolgende criteria kan samenvoegen en geen criteria kan schrappen. Naar aanleiding van de bovenstaande overwegingen heb ik getracht de criteria 'timing' en 'controleerbaarheid' samen te voegen met een ander criterium.

Het criterium 'draagvlak' betreft onder meer de vraag: *"In hoeverre en op welke manier(en) is er aandacht besteed aan het verwerven van draagvlak voor de preventiemaatregel?"*. Een goede 'timing' van de implementatie van de preventiemaatregel is een belangrijke manier om draagvlak te verwerven. Van het criterium 'timing' maak ik daarom een aspect van het criterium 'draagvlak'. Omdat ik de clusterstructuur (2 criteria per cluster) en de logische volgorde van de criteria niet wil veranderen, vormen de criteria 'draagvlak' en 'flankerend beleid' nu samen een beoordelingscluster. Inhoudelijk gezien is hier veel voor te zeggen, omdat afhankelijk van het draagvlak meer of minder flankerend beleid nodig is.

Op het eerste oog ligt het voor de hand om het criterium 'controleerbaarheid' samen te voegen met het criterium 'handhaafbaarheid'. In dat geval zou de handhaafbaarheid een aspect kunnen worden van het criterium 'controleerbaarheid'. Hiertegen zijn de volgende bezwaren:

– In de eerste plaats moet de 'controleerbaarheid' niet te sterk aan de 'handhaafbaarheid' worden gekoppeld. De handhaafbaarheid betreft de handhaafbaarheid binnen het kader van het gekozen 'ontwerp' (1e zeef) van de preventiemaatregel. Het criterium 'controleerbaarheid' betreft echter ook de evaluatie en daarmee heroverweging van het ontwerp van de preventiemaatregel. Uit de beoordeling van de casus 'wettelijk rookverbod in de horeca' wordt duidelijk hoe belangrijk het is om het ontwerp (inclusief de doelstellingen) van een preventiemaatregel van tijd tot tijd te evalueren. Is het bijvoorbeeld wel terecht dat de bescherming van bezoekers in de horeca tegen passief roken (nog steeds) geen doelstelling is van het wettelijk rookverbod (zie §10.6.2)? Nu ik dit opschrijf realiseer ik mij dat ik de reikwijdte van het criterium 'controleerbaarheid' onterecht tot de 'effecten en neveneffecten' (2e zeef) en 'implementatie' (3e zeef) van de preventiemaatregel heb beperkt. De reikwijdte van het crite-

beoordeling minder afhankelijk is van niet-wetenschappelijk te bewijzen opvattingen dan het tweede criterium, is hieraan ondergeschikt.

rium 'controleerbaarheid' dient dus te worden uitgebreid met het ontwerp (1e zeef) van de preventiemaatregel.

– In de tweede plaats dient het aspect van handhaafbaarheid nauw verbonden te blijven met het criterium 'flankerend beleid'. De handhaafbaarheid is immers een onderdeel van het flankerend beleid (zie §13.3.1). Als de handhaafbaarheid een aspect wordt van het criterium 'controleerbaarheid', behoort de handhaafbaarheid niet meer tot hetzelfde beoordelingscluster als het flankerend beleid en dat is inhoudelijk bezwaarlijk. Om de handhaafbaarheid weer in hetzelfde beoordelingscluster te laten vallen als het flankerend beleid, kies ik ervoor om het criterium 'handhaafbaarheid' samen te voegen met het criterium 'flankerend beleid'. De handhaafbaarheid wordt een aspect van het flankerend beleid en vervalt als zelfstandig criterium.

De voorgaande overwegingen leiden tot een aanpassing van de 3e zeef van het model en daarmee tot de 9e versie van het model (zie overzicht 22).

Overzicht 22: vergelijking van de 3e zeef van versie 8 en 9 van het model

**Versie 8 van het model**	**Versie 9 van het model**
**Derde zeef: implementatie**	**Derde zeef: implementatie**
Cluster 6	***Cluster 6***
Timing	Draagvlak (inclusief timing)
Draagvlak	Flankerend beleid (inclusief handhaafbaarheid)
Cluster 7	***Cluster 7***
Flankerend beleid	Controleerbaarheid
Handhaafbaarheid	Uitvoerbaarheid
Cluster 8	
Controleerbaarheid	
Uitvoerbaarheid	

Operationalisering van criteria 'draagvlak' en 'flankerend beleid'
Om een dubbeling in de tekst te voorkomen, volgt de nieuwe operationalisering van de criteria 'draagvlak' en 'flankerend beleid' bij de presentatie van de definitieve versie van het model in de discussie (§15.1).

Volgorde van criteria 'draagvlak' en 'flankerend beleid'
De beoordeling van de preventiemaatregel aan de hand van het criterium 'draagvlak' is minder afhankelijk van niet-wetenschappelijk te bewijzen opvattingen dan

de beoordeling aan de hand van het criterium 'flankerend beleid'. Het draagvlak voor de preventiemaatregel bij relevante partijen, is wetenschappelijk te onderzoeken. Bij de preventiemaatregel betrokken partijen (deskundigen en belanghebbenden) zullen vaak consensus kunnen bereiken over de vragen bij welke partijen en hoe het draagvlak onderzocht dient te worden. Het flankerend beleid ter ondersteuning van de preventiemaatregel kan ook wetenschappelijk worden onderzocht. Bij de preventiemaatregel betrokken partijen zullen echter vaak geen consensus kunnen bereiken over de vragen welk flankerend beleid onderzocht dient te worden en hoe dit onderzocht dient te worden. Welk flankerend beleid men nodig acht, hangt in belangrijke mate af van de opvattingen die men heeft over eigen verantwoordelijkheid, rechtvaardigheid en de noodzaak van handhavingsbeleid.

Volgorde van criteria 'controleerbaarheid' en 'uitvoeringscapaciteit'
Bij een prospectieve beoordeling aan de hand van het criterium 'uitvoeringscapaciteit' moet men een inschatting maken van de benodigde uitvoeringscapaciteit op basis van een groot aantal aannames m.b.t. de effecten en neveneffecten (2e zeef) en implementatie (3e zeef). Bij een prospectieve beoordeling aan de hand van het criterium 'controleerbaarheid' is men minder afhankelijk van aannames. Hierdoor is men bij een prospectieve beoordeling aan de hand van het criterium 'controleerbaarheid' minder afhankelijk van niet-wetenschappelijk te bewijzen opvattingen dan bij een prospectieve beoordeling aan de hand van het criterium 'uitvoeringscapaciteit'. Bij een retrospectieve beoordeling is dit verschil minder duidelijk, omdat men voor het beoordelen van de benodigde uitvoeringscapaciteit dan minder afhankelijk is van aannames. De 'effecten en neveneffecten' en 'implementatie' hebben dan immers al plaatsgevonden.

Soortelijk gewicht van criterium 'controleerbaarheid'
Het bezwaar, dat het 'soortelijk gewicht' van het criterium 'controleerbaarheid' geringer is dan dat van de andere criteria van het model, wordt minder nu ik de reikwijdte van het criterium heb uitgebreid met het 'ontwerp' (1e zeef) van de preventiemaatregel. Deze uitbreiding leidt tot een aanvulling op de beoordeling van het wettelijk rookverbod in de horeca aan de hand van het criterium 'controleerbaarheid' (zie §13.5). Op basis van de door mij bestudeerde documenten kom ik tot de conclusie dat bij de evaluatie van het wettelijk rookverbod (door minister Klink) onvoldoende aandacht is besteed aan de juistheid van het ontwerp van het wettelijk rookverbod in de horeca met name wat betreft de doelstelling. Deze tekortkoming van de evaluatie verandert mijn eindoordeel over de implementatie echter niet, omdat het wettelijk rookverbod in de horeca de 3e zeef van het model toch al niet kon passeren (zie §13.7.1).

Conclusies over juistheid, volledigheid en praktische toepasbaarheid

Met inachtneming van de bovenstaande aanvullingen kunnen de volgende conclusies worden getrokken. De beoordeling van het wettelijk rookverbod in de horeca aan de hand van de 3e zeef van het model leidt niet tot aanwijzingen dat de criteria van de 3e zeef van het model onjuist, onvolledig, of praktisch niet toepasbaar zouden zijn. Het blijkt mogelijk om op basis van de criteria van de 3e zeef van het model tot een beoordeling van de 'gerechtvaardigdheid van de implementatie' van het wettelijk rookverbod in de horeca te komen.

Ik acht het aannemelijk dat de praktische toepasbaarheid van het model nog iets is verbeterd, door het reduceren van het aantal criteria van de 3e zeef van het model van 6 naar 4. Deze reductie heeft niet tot inhoudelijke wijzigingen geleid, omdat ik alleen opeenvolgende criteria (waartussen overlap bestond) heb samengevoegd en geen criteria heb geschrapt.

14 Conclusies van toetsing aan casus ‘wettelijk rookverbod in horeca’ (versie 9 van het model)

In de hoofdstukken 11, 12 en 13 heb ik de gerechtvaardigdheid van het wettelijk rookverbod in de horeca beoordeeld aan de hand van versie 8 van het beoordelingsmodel. Het doel hiervan was het toetsen van de juistheid, volledigheid en praktische toepasbaarheid van het model. In dit hoofdstuk geef ik eerst een eindoordeel over de gerechtvaardigdheid van het wettelijk rookverbod in de horeca (§14.1). Vervolgens geef ik op basis van de toetsing van het model aan de hand van de casus ‘wettelijk rookverbod in de horeca’ een oordeel over de juistheid, volledigheid en praktische toepasbaarheid van het model (§14.2).

14.1 Eindoordeel over de gerechtvaardigdheid van het wettelijk rookverbod in de horeca

14.1.1 Gebrek aan transparantie over de doelstelling

In 2000 heeft Norman Daniels in de British Medical Journal het invloedrijke artikel *“Accountability for reasonableness. Establishing a fair process for priority setting is easier than agreeing on principles”* gepubliceerd:

> *“In pluralist societies we are likely to find reasonable disagreement about principles that should govern priority setting. For example, some will want to give more priority to the worst off, some less, some will be willing to aggregate benefits in ways that others are not. In the absence of consensus on principles, a fair process allows us to agree on what is legitimate and fair. Key elements of a fair process will involve transparency about the grounds for decisions; appeals to rationales that all can accept as relevant to meeting health needs fairly; and procedures for revising decisions in light of challenges to them. Together these elements assure “accountability for reasonableness”.”* (Daniels, 2000, p.1300)

> *“A fair process requires publicity about the reasons and rationales that play a part in decisions. There must be no secrets where justice is involved, for people should not be expected to accept decisions that affect their well being unless they are aware of the grounds for those decisions.”* (Daniels, 2000, p.1301)

"Accountability for reasonableness" waarvoor Daniels een overtuigend pleidooi heeft gehouden, heb ik gekozen als uitgangspunt voor de wijze van beoordeling aan de hand van het model (zie §4.1 en 4.2). ***"Transparency about the grounds for decisions"*** is volgens Daniels een kernelement van *"accountability for reasonableness"*.

In de politiek is in het openbaar uitgebreid gedebatteerd over de invoering van het wettelijk rookverbod in de horeca. Daarom vind ik het opmerkelijk dat uit de bestudering van parlementaire verslagen blijkt, dat dit debat niet heeft geleid tot transparantie over de doelstellingen van het wettelijk rookverbod in de horeca. Achtereenvolgende ministers van VWS hebben over de doelstelling van het wettelijk rookverbod in de horeca wisselende standpunten ingenomen (zie §10.5):

- In oktober 2003 schrijft minister Hoogervorst aan de vaste commissie van de Tweede Kamer voor VWS, dat de doelstelling van het stappenplan van KHN is een rookvrije horeca ter ***bescherming van werknemers en bezoekers*** in de horeca tegen passief roken.
- In juni 2007 (een jaar voor invoering van het wettelijk rookverbod in de horeca) stelt minister Klink in de Tweede Kamer dat de doelstelling ("het oogmerk") van het wettelijk rookverbod in de horeca ***primair*** is de ***bescherming van werknemers*** in de horeca tegen passief roken en ***secundair*** de ***bescherming van bezoekers*** in de horeca tegen passief roken.
- In juli 2007 schrijft minister Klink aan de Tweede Kamer dat de ***bescherming van werknemers en bezoekers*** de reden is om per 1 juli 2008 een wettelijk rookverbod in de horeca in te voeren.
- In juni 2009 (een jaar na invoering van het wettelijk rookverbod in de horeca) stelt minister Klink in een spoeddebat in de Tweede Kamer dat ***alleen de bescherming van werknemers*** in de horeca de reden was om per 1 juli 2008 een wettelijk rookverbod in de horeca in te voeren, en dat dit wat hem betreft zo blijft.
- In juli 2009 wil minister Klink het 'Besluit uitvoering rookvrije werkplek, horeca en andere ruimten' ***zodanig aanpassen, dat werknemers en bezoekers*** in de horeca tegen passief roken ***worden beschermd***. Blijkbaar worden formeel juridisch alleen werknemers beschermd!
- In september 2009 stelt minister Klink dat het uitgangspunt van de Tabakswet steeds is geweest de ***bescherming van werknemers en bezoekers*** in openbare ruimten tegen passief roken. Hij wil dit uitgangspunt nu ook toepassen op de horeca.
- In januari 2011 stelt minister Schippers dat het uitgangspunt van de "wet" is het ***beschermen van werknemers*** tegen tabaksrook. De bescherming van bezoekers

in de horeca tegen passief roken is volgens minister Schippers geen doelstelling van het wettelijk rookverbod in de horeca.

De Tweede Kamer had in juni 2007 m.i. geen genoegen mogen nemen met de uitspraak van minister Klink dat de bescherming van bezoekers een secundaire doelstelling is van het wettelijk rookverbod in de horeca. Het begrip secundaire doelstelling blijkt multi-interpretabel. In juli 2007 vindt minister Klink een secundaire doelstelling 'een doelstelling'. In dat geval valt de bescherming van bezoekers tegen passief roken onder het criterium 'schadelijkheid' van de 1e zeef van het model. In juni 2009 vindt minister Klink een secundaire doelstelling niet meer dan 'een positief neveneffect'. In dat geval valt de bescherming van bezoekers tegen passief roken onder het criterium 'lasten-batenverhouding' van de 2e zeef van het model. Deze wisseling van standpunt over de doelstelling van het wettelijk rookverbod in de horeca is m.i. door minister Klink onvoldoende beargumenteerd. Vanuit het ***perspectief van transparantie*** over de doelstellingen van het wettelijk rookverbod in de horeca, kan dit rookverbod tijdens de bewindsperiode van minister Klink, de 1e zeef van het beoordelingsmodel (logica van het ontwerp) ***niet*** passeren. Invoering van een wettelijk rookverbod in de horeca, zonder dat de doelstellingen helder zijn, acht ik onvoldoende transparant.

Met het oog op de vrije toegankelijkheid van openbare voorzieningen voor iedereen, vind ik persoonlijk dat ook bezoekers in de horeca wettelijk tegen passief roken beschermd dienen te worden. Daarom ben ik het oneens met de stellingname van minister Schippers dat de doelstelling van het wettelijk rookverbod in de horeca zich dient te beperken tot de bescherming van werknemers in de horeca tegen passief roken. Deze stellingname van minister Schippers is in strijd met de geest van (zie onder het criterium 'legitimiteit' in §12.4.2):
- de Tabakswet;
- het WHO-Kaderverdrag inzake tabaksontmoediging (2003);
- de afspraken van minister Hoogervorst van VWS met KHN over het stappenplan ter zelfregulering van het rookbeleid in de horeca (2004);
- de Aanbeveling van de Raad van Europa betreffende rookvrije ruimten (2009).

Mijn persoonlijke opvatting over de noodzakelijkheid van wettelijke bescherming van bezoekers in de horeca tegen passief roken, is bij de beoordeling van de gerechtvaardigdheid van het wettelijk rookverbod in de horeca aan de hand van het model echter niet relevant. Dat de stellingname van minister Schippers niet in overeenstemming is met de geest van de vier bovengenoemde documenten, acht ik onvoldoende reden om de stellingname van minister Schippers ongerechtvaardigd te verklaren. Formeel juridisch vormen de bovengenoemde documenten m.i. geen

(onoverkomelijke) belemmering voor de stellingname van minister Schippers. Vanuit het ***perspectief van transparantie*** over de doelstellingen van het wettelijk rookverbod in de horeca, kan dit rookverbod tijdens de bewindsperiode van minister Schippers, de 1e zeef van het beoordelingsmodel (logica van het ontwerp) ***wel*** passeren. De visie van minister Schippers is transparant: de doelstelling van het wettelijk rookverbod in de horeca dient zich te beperken, en beperkt zich, tot de bescherming van werknemers tegen passief roken.

14.1.2 Gerechtvaardigdheid van het wettelijk rookverbod in de horeca met de formele wetstekst als uitgangspunt

Formele wetstekst

Vanwege de onduidelijkheid over de doelstelling van het wettelijk rookverbod in de horeca tijdens de bewindsperiode van minister Klink, heb ik de gerechtvaardigdheid van het wettelijk rookverbod in de horeca beoordeeld met de formele wetstekst als uitgangspunt. De formele wetstekst biedt alleen werknemers bescherming tegen passief roken (zie §10.5). Deze beperkte doelstelling van het wettelijk rookverbod in de horeca is vanuit het perspectief van het model op zichzelf niet ongerechtvaardigd (zie §12.6.4).

Logica van het ontwerp (1e zeef)

Het wettelijk rookverbod in de horeca kan de 1e zeef van het model ***wel*** passeren: het ontwerp is logisch. Het ontwerp is niet moralistisch, niet perfectionistisch en niet paternalistisch (zie §11.7.1).

Effecten en neveneffecten (2e zeef)

Het wettelijk rookverbod in de horeca, zoals minister Klink dat per 1 juli 2007 heeft ingevoerd, kan de 2e zeef van het model ***niet*** passeren: een van de (mogelijke) neveneffecten is onbillijk (onrechtvaardig) (zie §12.5.1). Het aspect 'hardheid in specifieke gevallen' van het criterium 'rechtvaardigheid' vormt het breekpunt van de gerechtvaardigdheid van het rookverbod in kleine cafés zonder personeel. In verhouding tot de doelstelling van het rookverbod in kleine cafés zonder personeel – het voorkomen van concurrentievervalsing met cafés met personeel – vallen tijdens de bewindsperiode van minister Klink de financiële gevolgen voor deze cafés mogelijk onevenredig hard uit. Ik schrijf 'mogelijk' omdat een degelijke financiële onderbouwing ontbreekt (zie bijlage 10). Hierbij weeg ik mee dat de concurrentievervalsing in de praktijk wel meevalt. Zodra kleine cafés zonder personeel meer bezoekers gaan trekken, omdat er mag worden gerookt, moeten zij immers personeel in dienst nemen en is het rookverbod direct weer van toepassing.

Het huidige wettelijk rookverbod in de horeca, waarvan kleine cafés zonder personeel door minister Schippers zijn uitgezonderd, kan de 2e zeef van het model ***wel*** passeren.

Implementatie (3e zeef)

Het wettelijk rookverbod in de horeca kan de 3e zeef 'implementatie' van het model ***niet*** passeren. De wijze waarop is geïmplementeerd, is op onderdelen onredelijk (onlogisch) en onbillijk (onrechtvaardig) (§13.7.1).

Minister Klink heeft bij de implementatie van het wettelijk rookverbod in de horeca m.i. te veel steken laten vallen om de manier waarop is geïmplementeerd gerechtvaardigd te kunnen noemen. Hij heeft te weinig aandacht besteed aan:

- het behoud en de versterking van het draagvlak voor het wettelijk rookverbod in de horeca bij KHN en bij horecaondernemers (zie §13.1.3 en §13.2.3);
- de vermeende negatieve (financiële) gevolgen van de implementatie voor kleine cafés zonder personeel (zie §13.3.3 en 13.4.3);
- de juistheid van het ontwerp van het wettelijk rookverbod in de horeca (met name wat betreft de doelstelling) bij de evaluatie van het wettelijk rookverbod (zie in §13.7.2 onder het kopje *'Samenvoegen van beoordelingscriteria'*).

Minister Schippers is m.i. bij de versoepeling van het wettelijk rookverbod in de horeca – de vrijstelling van kleine cafés zonder personeel van dit rookverbod – onachtzaam en nalatig geweest m.b.t. de gevolgen voor de handhaving van het rookverbod. Hierdoor is naar het zich laat aanzien niemand direct ernstig in de problemen gekomen (§13.4.3). Dat de manier waarop minister Schippers vormgeeft aan de handhaving van het wettelijk rookverbod in de cafés suboptimaal is, betekent niet dat de toepassing van het rookverbod in cafés met personeel dan maar moet worden stopgezet. In tegendeel, want ondanks de suboptimale handhaving zijn de resultaten hiervan nog steeds zeer de moeite waard.

Een suboptimale handhaving van het wettelijk rookverbod in cafés met personeel leidt echter wel:

- tot een suboptimale effectiviteit (2e zeef),
- tot rechtsongelijkheid tussen de cafés met personeel (2e zeef),
- tot ondermijning van het draagvlak voor het rookverbod in cafés met personeel (3e zeef), en
- mogelijk tot uitholling van de geloofwaardigheid van wetgeving in het algemeen (2e zeef, negatief neveneffect).

Daarom mag van minister Schippers een grote inspanning worden gevraagd om de handhaving te verbeteren.

14.2 Oordeel over de juistheid, volledigheid en praktische toepasbaarheid van het model

In de hoofdstukken 11, 12 en 13 is de juistheid, volledigheid en praktische toepasbaarheid van het model getoetst aan de casus 'wettelijk rookverbod in de horeca'. Deze toetsing heeft geleid tot de volgende aanvullingen en aanpassingen van en conclusies over het model:

- In versie 8 van het model zijn de criteria ***'handhaafbaarheid' en 'controleerbaarheid'*** toegevoegd aan de 3e zeef van het model (zie overzicht 17 in §10.4).
- In versie 9 van het model zijn de criteria ***'timing' en 'handhaafbaarheid'*** vervallen als zelfstandige criteria. De 'timing' is een aspect geworden van het criterium 'draagvlak' en de 'handhaafbaarheid' is een aspect geworden van het criterium 'flankerend beleid' (zie overzicht 22 in §13.7.2).
- De conclusie dat per criterium van het model dient te worden afgewogen in hoeverre ***procedurele aspecten relevant*** zijn voor de inhoudelijke beoordeling van de gerechtvaardigdheid van de preventiemaatregel (zie §12.4.4).
- De introductie van het ***'optimalisatiebeginsel'*** (geen ***onachtzaamheid*** en geen ***nalatigheid***) als aanvullend uitgangspunt voor de beoordeling van de gerechtvaardigdheid van de implementatie van de preventiemaatregel (zie §13.3.1).
- Het veranderen van de naamgeving van het criterium 'uitvoerbaarheid' in ***'uitvoeringscapaciteit'*** (zie §13.6.1).
- De conclusie dat bij de beoordeling van de 'effecten en neveneffecten' van de preventiemaatregel (2e zeef) de invloed (die men verwacht) van de ***implementatie*** dient te worden ***verdisconteerd*** (zie §13.7.2).
- De conclusie dat bij de 3e zeef van het model de ***implementatie op zichzelf*** wordt beoordeeld. De invloed van de implementatie op de 'effecten en neveneffecten' van de preventiemaatregel is al verdisconteerd in de beoordeling van de preventiemaatregel aan de hand van de 2e zeef (effecten en neveneffecten) van het model (zie §13.7.2).
- De conclusie dat ***per situatie*** dient te worden beoordeeld ***welke consequenties*** moeten worden verbonden aan het niet passeren van de 3e zeef van het model. Uiteindelijk hangt dit af van de invloed van de suboptimale implementatie op de 'effecten en neveneffecten' van de preventiemaatregel (zie §13.7.2).
- De kanttekening dat de ***criteria uit de 3e zeef*** van het model ***ook na afloop van de implementatiefase relevant*** kunnen zijn voor de beoordeling van de gerechtvaardigdheid van een preventiemaatregel (zie §13.7.2).

– De conclusie dat bij een ***prospectieve beoordeling*** gewerkt moet worden met ***aannames*** over de effecten en neveneffecten (2e zeef) en implementatie (3e zeef) van de preventiemaatregel en dat gewerkt kan worden met ***implementatiescenario's, experimenten en pilotstudies*** (zie §13.7.2).
– Diverse kleine aanpassingen en aanvullingen van het model die zijn te vinden in de paragrafen 13.1.4, 13.2.4, 13.3.4, 13.4.4, 13.5.4, 13.6.4 en 13.7.2. Deze paragrafen beschrijven ***per criterium*** de gevolgen van de toetsing voor het model.
– Ten slotte heeft de toetsing van het model aan de casus wettelijk rookverbod in de horeca een belangrijke bijdrage geleverd aan de ***operationalisering van de criteria*** van het model. Het resultaat hiervan is te vinden in de discussie (§15.1).

Met inachtneming van de bovenstaande wijzigingen kom ik, op basis van de toetsing van de casus 'wettelijk rookverbod in de horeca' aan de hand van het model, tot de volgende conclusies over het model. De toetsing van de casus 'wettelijk rookverbod in de horeca' aan de hand van het model heeft geen aanwijzingen opgeleverd dat het model onvolledig of onjuist is. Voor de toetsing van de gerechtvaardigdheid van het wettelijk rookverbod in de horeca is het model goed 'praktisch bruikbaar' gebleken. Ik acht het aannemelijk dat de praktische toepasbaarheid van het model nog iets is verbeterd, door het reduceren van het aantal criteria van de 3e zeef van het model van 6 (in versie 8) naar 4 (in versie 9). Nog steeds geldt dat naarmate het model aan meer verschillende casuïstiek wordt getoetst, er (nog) meer zekerheid over de juistheid, volledigheid en praktische toepasbaarheid van het model zal ontstaan. Overzicht 23 (in §15.1.1) toont versie 9 - de laatste versie – van het model.

15 Discussie

De discussie kent de volgende indeling:
- beantwoording van de onderzoeksvraag door presentatie van het model (§15.1);
- beschouwing van mijn onderzoek en beoordelingsmodel (§15.2);
- relevantie van mijn onderzoek en beoordelingsmodel (§15.3).

15.1 Beantwoording van de onderzoeksvraag door presentatie van het model

De definitieve onderzoeksvraag die ik tracht te beantwoorden luidt (zie §1.4):

> Kan een model worden ontwikkeld aan de hand waarvan de gerechtvaardigdheid van preventiemaatregelen kan worden beoordeeld, die ter bestrijding van leefstijlgerelateerde aandoeningen trachten de leefstijl van mensen te beïnvloeden?

In deze paragraaf beantwoord ik de bovenstaande onderzoeksvraag door het presenteren van de definitieve versie van het model. Het model is samengesteld uit drie onderdelen:
1. ***Beoordelingscriteria:***
   - operationalisering van de criteria (§15.1.1).
2. ***Beoordelingsstructuur:***
   - beoordelingszeven (expliciete beoordelingsstructuur) (§15.1.2);
   - beoordelingsclusters (impliciete beoordelingsstructuur) (§15.1.2).
3. ***Wijze van beoordeling:***
   - wijze van beoordeling op hoofdlijnen (§15.1.3);
   - wijze van beoordeling per criterium (§15.1.1).

Voor de overzichtelijkheid presenteer ik de ‘wijze van beoordeling per criterium’ tegelijk met de ‘operationalisering van het criterium’ in §15.1.1.

15.1.1 Beoordelingscriteria

De ontwikkeling van versie 1 t/m 6 van het model is vooral gebaseerd op literatuur over bestaande beoordelingsmodellen (zie hoofdstuk 2 t/m 4). De ontwikkeling van versie 7 t/m 9 van het model is gebaseerd op de toetsing van het model aan casuïstiek uit de praktijk (zie hoofdstuk 7 t/m 14). De definitieve beoordelingscriteria van het model worden weergegeven in overzicht 23.

Overzicht 23: definitieve beoordelingscriteria van het model

preventiemaatregel

↓

Logica van ontwerp (1e zeef)

1. Schadelijkheid
2. Noodzakelijkheid
3. Oorzakelijkheid
4. Verantwoordelijkheid
5. Gerichtheid
6. Geschiktheid

↓

Effecten en neveneffecten (2e zeef)

7. Effectiviteit
8. Indringendheid
9. Lasten-batenverhouding
10. Rechtvaardigheid

↓

Implementatie (3e zeef)

11. Draagvlak
12. Flankerend beleid
13. Controleerbaarheid
14. Uitvoeringscapaciteit

↓

Gerechtvaardigde preventiemaatregel

Hieronder volgt de definitieve operationalisering van de criteria met de bijbehorende wijze van beoordeling.

1. Criterium schadelijkheid

Operationalisering van het criterium

Het criterium ‘schadelijkheid’ betreft de aard en omvang van de risico’s op:

- schade:
  - gezondheidsschade;
  - sociaal-economische schade;
  - inbreuk op het persoonlijk leven;
- hinder of overlast
- schending van morele opvattingen.

Het gaat hierbij om de risico’s die men met de preventiemaatregel tracht te bestrijden. Het begrip ‘risico’ wordt als volgt gedefinieerd: risico = kans x gevolg (zie overzicht 24).

Overzicht 24: risico = kans x gevolg

	Kleine kans	Grote kans
**Klein nadeel**	Beperkt risico	Risico
**Groot nadeel**	Risico	Ernstig risico

De aard van de risico’s kent als invalshoeken:

- van wat voor soort risico’s is er sprake?
- wie worden benadeeld:
  - personen met de ongezonde of onveilige leefstijl zelf, of derden?
  - volwassenen of kinderen?

Voorbeelden van soorten risico’s zijn het risico op (zie bijlage 5):

- gezondheidsschade (fysieke of psychische schade);
- sociaal-economische schade (bv. schade aan sociale contacten, aan maatschappelijke participatie, aan bezit, aan opleiding, aan inkomen, aan arbeidsgeschiktheid, aan werk, aan winst, aan concurrentiepositie);
- inbreuk op het persoonlijk leven (aantasting van de lichamelijke integriteit, van de vrijheid, van de privacy, van de veiligheidsbeleving, van de gelijke behandeling, van de eer en goede naam, van de waardigheid, en van de respectvolle bejegening);
- hinder of overlast (bv. verkeershinder, geluidsoverlast);
- schending van morele opvattingen (bv. schending van de opvatting ‘drugsgebruik is verwerpelijk’, van de opvatting ‘wisselende seksuele contacten zijn verwerpelijk’).

De omvang van de risico's dient zoveel mogelijk te worden gekwantificeerd door deze uit te drukken in geld. Gezondheidsrisico's kunnen worden gekwantificeerd door deze uit te drukken in QALY's, DALY's, of reductie van ziektelast. Immateriele zaken kunnen worden gekwantificeerd door gebruik te maken van concepten als 'Willingness to Pay' en Willingness to Accept'. (Zie §6.6.1)

Wijze van beoordeling
Beoordeeld wordt of de beschikbare informatie een duidelijk beeld geeft van de aard en omvang van de risico's die men met de preventiemaatregel tracht te bestrijden. Als dat oordeel negatief uitvalt, wordt beoordeeld of de informatieverzameling over deze risico's voldoende is geweest. Heeft men voldoende gebruik gemaakt van de mogelijkheden om de risico's te inventariseren en kwantificeren?

2. Criterium noodzakelijkheid

Operationalisering van het criterium
Het criterium 'noodzakelijkheid' betreft de vraag in hoeverre het noodzakelijk is om, ter bestrijding van de onder het eerste criterium geïnventariseerde risico's, leefstijlbeïnvloedende preventiemaatregelen te treffen. Belangrijke aandachtspunten zijn:
- In hoeverre is de preventiemaatregel moralistisch? Tracht men risico's op schade, hinder of overlast te bestrijden? Of tracht men de schending van morele opvattingen te bestrijden (het bestrijden van de schending van morele opvattingen is moralistisch)?
- In hoeverre is de preventiemaatregel perfectionistisch? Is er sprake van een substantieel risico op schade, hinder of overlast? Of overdrijft men de risicopreventie?
- Is er sprake van sancties die worden opgelegd aan personen of organisaties die de risico's (mede) veroorzaken? Deze kunnen een indicatie geven van de noodzakelijkheid van preventie van de betreffende risico's vanuit de optiek van de sanctionerende partij.

In het algemeen kan worden gesteld dat het treffen van een preventiemaatregel noodzakelijk is, als er sprake is van een substantieel risico op schade, hinder of overlast voor derden.

Wijze van beoordeling
Beoordeeld wordt of overtuigend wordt beargumenteerd dat de bestrijding van de risico's, die men met de preventiemaatregel tracht te bestrijden, noodzakelijk is. Tevens wordt beoordeeld of de bestrijding van deze risico's niet te veel ten koste gaat van de bestrijding van andere risico's die ernstiger zijn (optimalisatiebeginsel).

3. Criterium oorzakelijkheid

Operationalisering van het criterium
Het criterium ‘oorzakelijkheid’ betreft de vraag naar de determinanten van de risico’s die men met de preventiemaatregel tracht te bestrijden:
- In hoeverre zijn de leefstijlen waarop de preventiemaatregel zich richt bepalend voor de risico’s die men met de preventiemaatregel tracht te bestrijden (zie criterium ‘schadelijkheid’)?
- Welke factoren zijn bepalend voor de leefstijlen waarop de preventiemaatregel zich richt en in hoeverre zijn deze factoren bepalend?

Factoren die medebepalend zijn voor een bepaalde leefstijl (2e vraag) zijn bijvoorbeeld:
- persoonlijkheidsfactoren (bv. mate van impulsiviteit, afhankelijkheid, wilskracht);
- gezondheidsfactoren (bv. een bepaalde genetische aanleg, bepaalde aandoeningen);
- sociaal-economische factoren (bv. sociaal-economische status, werkloosheid);
- sociaal-culturele factoren (bv. de eetgewoonten van een bepaalde etnische groep, bij tieners de druk vanuit de ‘peergroup’ om te roken of alcohol te drinken);
- (overige) omgevingsfactoren (bv. de obesogene omgeving).

Om reden van pragmatisme dient de focus uit te gaan naar de factoren die in grote mate bepalend zijn voor de leefstijl die men met de preventiemaatregel wil beïnvloeden.
Regelmatig treedt er een wisselwerking op tussen verschillende factoren en zijn bepaalde factoren zowel oorzaak als gevolg in het ontstaan van een leefstijlgerelateerde aandoening. Daarom wordt bij voorkeur niet gesproken over een ‘keten van oorzaken’, maar over een ***‘complex van factoren’*** dat aan het ontstaan van een leefstijlgerelateerde aandoening ten grondslag ligt.

Wijze van beoordeling
Beoordeeld wordt of de beschikbare informatie een duidelijk beeld geeft van (de wisselwerking tussen) de determinanten van de risico’s die men tracht te bestrijden. Is deze informatie juist, volledig, voldoende gedetailleerd en voldoende gekwantificeerd? Als dat oordeel negatief uitvalt, wordt beoordeeld of de informatieverzameling over de oorzakelijkheid voldoende is geweest. Heeft men voldoende gebruik gemaakt van de mogelijkheden om de determinanten van de risico’s die men tracht te bestrijden te inventariseren en te kwantificeren?

4. Criterium verantwoordelijkheid

Operationalisering

Het criterium 'verantwoordelijkheid' betreft de vragen:

- Welke partijen worden verantwoordelijk gehouden voor het ontstaan en/of de instandhouding van de risico's die men met de preventiemaatregel tracht te bestrijden?
- Is bij het vaststellen van de verantwoordelijkheid voor het ontstaan van deze risico's wel rekening gehouden met alle bekende oorzaken c.q. determinanten van deze risico's?
- Is het treffen van de preventiemaatregel niet paternalistisch?

Het antwoord op deze vragen wordt bepaald door een combinatie van empirische feiten met ideologische opvattingen. De empirische feiten betreffen de determinanten van de risico's die men wil bestrijden (zie het criterium 'oorzakelijkheid'). De ideologische opvattingen betreffen de opvattingen die men heeft over:

- de verantwoordelijkheid van volwassenen en van kinderen voor het gedrag, voor de gezondheid, voor het welzijn en voor de omgeving van zichzelf en van derden;
- de verantwoordelijkheid van de overheid voor het gedrag, voor de gezondheid, voor het welzijn en voor de leefomgeving van volwassenen en van kinderen (bv. 'life politics') (Scholtmeijer, 2006);
- de verantwoordelijkheid van private partijen (bv. scholen, zorginstellingen, bedrijven) voor het gedrag, voor de gezondheid, voor het welzijn en voor de leefomgeving van volwassenen en van kinderen;
- de fenomenen 'accumulerende schade' en 'collectieve verantwoordelijkheid' (Kernohan, 1993);
- de (on)wenselijkheid van varianten van paternalisme (bv. hard paternalisme, zacht paternalisme en libertair paternalisme) (Sunstein en Thaler, 2003) (Camerer, 2003).

De (financiële) aansprakelijkheid voor de (kosten van) de schade en schadepreventie komt pas bij het 9e criterium 'rechtvaardigheid' aan de orde.

Wijze van beoordeling

Beoordeeld wordt of:

- duidelijk is welke partijen verantwoordelijk worden gehouden voor het ontstaan en/of de instandhouding van de risico's die men met de preventiemaatregel tracht te bestrijden;

– bij het vaststellen van de verantwoordelijkheid voor het ontstaan van deze risico's voldoende rekening is gehouden met alle bekende oorzaken c.q. determinanten van deze risico's;
– het treffen van de preventiemaatregel niet ongewenst paternalistisch is.

5. Criterium gerichtheid

Operationalisering van het criterium
Het criterium 'gerichtheid' betreft de doelgroep en de leefstijlbeïnvloedende factoren waarop de preventiemaatregel zich richt. Het gaat hierbij om de volgende vragen:
– Hangt de keuze van de doelgroep en van de leefstijlbeïnvloedende factoren logisch samen met de determinanten van de risico's die men tracht te bestrijden en met de verantwoordelijkheid voor deze risico's?
– In hoeverre worden personen binnen de doelgroep door de preventiemaatregel gemist?
– In hoeverre worden personen buiten de doelgroep door de preventiemaatregel geraakt?

Toelichting:
– De doelgroep van de preventiemaatregel is gedefinieerd als de groep personen waarvan men ter voorkoming van leefstijlgerelateerde aandoeningen de leefstijl tracht te beïnvloeden.
– De keuze van de doelgroep en/of leefstijlbeïnvloedende factoren waarop de preventiemaatregel zich richt, dient logisch samen te hangen met de determinanten van de risico's die men tracht te bestrijden (criterium 'oorzakelijkheid') en met de verantwoordelijkheid voor deze risico's (criterium 'verantwoordelijkheid').
– Een rookverbod in openbare gebouwen is een voorbeeld van een interventie die alleen de doelgroep (rokers in openbare gebouwen) raakt.
– Een accijnsverhoging op alcohol is een voorbeeld van een interventie die niet alleen de doelgroep (mensen met een (potentieel) alcoholprobleem) raakt, maar ook derden (mensen die geen (potentieel) alcoholprobleem hebben).

Wijze van beoordeling
Beoordeeld wordt of:
– onevenredig of onnodig veel personen binnen de doelgroep door de interventie worden gemist;
– onevenredig of onnodig veel personen buiten de doelgroep door de interventie worden geraakt;

- het niet beter zou zijn om de preventiemaatregel op een andere doelgroep of op andere leefstijlbeïnvloedende factoren te richten (optimalisatiebeginsel).

Toelichting:
- 'Onevenredig veel' betekent onevenredig veel ten opzichte van de omvang van de doelgroep.

6. Criterium geschiktheid

Operationalisering van het criterium
Het criterium 'geschiktheid' betreft de partijen die de preventiemaatregel uitvoeren. Het gaat hierbij om de volgende vragen:
- Hangt de keuze van de partijen die de preventiemaatregel uitvoeren logisch samen met de gerichtheid van de preventiemaatregel (doelgroep en leefstijlbeïnvloedende factoren)?
- Zijn de partijen die de preventiemaatregel uitvoeren daartoe bekwaam en bevoegd?
- Zijn de partijen die de preventiemaatregel uitvoeren daartoe geschikt?
- Brengt uitvoering van de preventiemaatregel schade toe aan de aard of het imago van de partijen die de maatregel uitvoeren?

Uitgangspunt is dat wie bekwaam is, daardoor bevoegd is (mits er geen sprake is van een voorbehouden handeling).

Wijze van beoordeling geschiktheid
Beoordeeld wordt of:
- uitvoering van de preventiemaatregel door andere partijen niet logischer is, gezien de doelgroep en leefstijlbeïnvloedende factoren waarop de maatregel zich richt;
- de partijen die de preventiemaatregel uitvoeren daartoe niet onbekwaam of onbevoegd zijn;
- de partijen die de preventiemaatregel uitvoeren daartoe niet ongeschikt zijn;
- de preventiemaatregel geen onaanvaardbare schade toebrengt aan de aard of het imago van de partijen die de maatregel uitvoeren;
- andere partijen niet geschikter zijn om de preventiemaatregel uit te voeren (optimalisatiebeginsel).

7. Criterium effectiviteit

Operationalisering van het criterium

Het criterium 'effectiviteit' betreft de beoogde effecten van de preventiemaatregel op de doelgroep of op derden. De doelgroep is hierbij gedefinieerd als de groep personen waarvan men de leefstijl tracht te beïnvloeden ter voorkoming van leefstijlgerelateerde aandoeningen bij de doelgroep zelf of bij derden. Belangrijke aandachtspunten zijn:

- effectiviteit per risico dat men tracht te bestrijden;
- kwantificering van de effectiviteit;
- gebruik van proces- en uitkomstindicatoren;
- duurzaamheid van de effecten.

Wijze van beoordeling

Beoordeeld wordt of de preventiemaatregel effectief is in de beoogde risicobestrijding en of deze effecten duurzaam zijn. Is de informatie over de effectiviteit volledig, voldoende gedetailleerd en voldoende gekwantificeerd? Als dat oordeel negatief uitvalt, wordt beoordeeld of de informatieverzameling over de effectiviteit voldoende is geweest. Tevens wordt beoordeeld of een ander ontwerp van de preventiemaatregel niet effectiever zou zijn (optimalisatiebeginsel).

8. Criterium indringendheid [1]

Operationalisering van het criterium

Het criterium 'indringendheid' betreft de verhouding tussen de effectiviteit en de indringendheid van de preventiemaatregel. De indringendheid van de preventiemaatregel betreft de inbreuk van de preventiemaatregel op het persoonlijk leven van mensen. De indringendheid wordt in drie stappen beoordeeld:

I. Algemene beoordeling van de indringendheid:
   1. Bij wie wordt een inbreuk gemaakt op het persoonlijk leven?
   2. Door wie wordt een inbreuk gemaakt op het persoonlijk leven?
   3. In welke mate wordt een inbreuk gemaakt op het persoonlijk leven?
   4. Welke reikwijdte heeft de inbreuk op het persoonlijk leven?
   5. Leidt het toestaan van de inbreuk op het persoonlijk leven tot precedentwerking?
   6. Hoe bezwaarlijk is de inbreuk op het persoonlijk leven?

1. Zie voor een uitgebreide beschrijving van het criterium 'indringendheid' §7.2.1.

II. Beoordeling van verschillende aspecten van de indringendheid:
   a. aantasting van de lichamelijke integriteit;
   b. aantasting van de vrijheid;
   c. aantasting van de privacy;
   d. aantasting van de veiligheidsbeleving;
   e. discriminatie;
   f. stigmatisering.
III. Beoordeling van de mate van drang of dwang die wordt toegepast.

Bij stap II kan afhankelijk van de betreffende preventiemaatregel per aspect (a t/m f) worden besloten om, al dan niet en meer of minder uitgebreid, op de zes vragen (1 t/m 6) in te gaan.

Wijze van beoordeling
De preventiemaatregel passeert het criterium 'indringendheid', als niet kan worden aangetoond dat de verhouding tussen de effectiviteit en indringendheid onaanvaardbaar is.

9. Criterium kosten-batenverhouding

Operationalisering van het criterium
Het criterium 'lasten-batenverhouding' betreft de verhouding tussen de lasten en baten van de preventiemaatregel (zie overzicht 25). De kosteneffectiviteit van de preventiemaatregel is onderdeel van het criterium 'lasten-batenverhouding'. De kosteneffectiviteit betreft de verhouding tussen de uitvoeringskosten en de effectiviteit van de preventiemaatregel. Het verdient aanbeveling om de uitvoeringskosten wel afzonderlijk zichtbaar te maken. De uitvoeringskosten vallen onder de overige lasten van de preventiemaatregel (zie overzicht 25).

Overzicht 25: criterium lasten-batenverhouding

Lasten-batenverhouding	
Lasten van de preventiemaatregel	***Baten van de preventiemaatregel***
Indringendheid van de preventiemaatregel (8e criterium)	Effectiviteit van de preventiemaatregel (7e criterium) (beoogde effecten)
Overige lasten van de preventiemaatregel	Overige baten van de preventiemaatregel (positieve neveneffecten)

De indringendheid (8e criterium) en effectiviteit (7e criterium) zijn al geanalyseerd. Alleen de overige lasten en baten (grijs gearceerd) zijn nog niet geanalyseerd. Als alle lasten en baten op dezelfde wijze worden gekwantificeerd, c.q. worden uitgedrukt in geld, kan de lasten-batenverhouding worden berekend door de waarde van alle baten te delen door de waarde van alle lasten. Als de uitkomst van deze deling groter is dan één, dan is de lasten-batenverhouding gunstig. Als deze kleiner is dan één, dan is de lasten-batenverhouding ongunstig en is toepassing van de preventiemaatregel niet gerechtvaardigd. Ook als het niet lukt om alle lasten en baten te kwantificeren, is het nuttig om alle lasten en baten in één overzichtelijk schema te plaatsen. De duurzaamheid van de lasten en van de baten vormt een belangrijkrijk aandachtspunt.

Wijze van beoordeling
Beoordeeld wordt of de 'lasten-batenverhouding' gunstig is en hoe deze zich in de loop van de tijd ontwikkelt. Tevens wordt beoordeeld of de beschikbare informatie over de lasten-batenverhouding volledig, voldoende gedetailleerd en voldoende gekwantificeerd is. Als dat oordeel negatief uitvalt, wordt beoordeeld of de informatieverzameling over de lasten-batenverhouding voldoende is geweest. Ten slotte wordt beoordeeld of een ander ontwerp van de preventiemaatregel niet gunstiger zou zijn voor de lasten-batenverhouding (optimalisatiebeginsel).

De toepassing van preventiemaatregelen met een ongunstige lasten-batenverhouding is niet gerechtvaardigd. In theorie is het mogelijk dat de beoordeling van de preventiemaatregel aan de hand van de criteria 'effectiviteit' en 'indringendheid' ongunstig uitvalt, terwijl de lasten-batenverhouding toch gunstig uitvalt. Dat kan het geval zijn als de positieve neveneffecten zeer groot zijn. M.i. is het onredelijk om een ongunstige beoordeling van de 'effectiviteit' en 'indringendheid' te compenseren met een gunstige beoordeling van de 'lasten-batenverhouding'. Als men in dat geval toch de toepassing van de preventiemaatregel wil rechtvaardigen, is een mogelijke oplossing om de positieve neveneffecten aan de doelstelling van de preventiemaatregel toe te voegen en de preventiemaatregel vanaf het 1e criterium opnieuw te beoordelen.

10. Criterium rechtvaardigheid

Operationalisering van het criterium
Het criterium 'rechtvaardigheid' betreft de distributieve rechtvaardigheid, vergelijkende rechtvaardigheid en legitimiteit van de preventiemaatregel:
- de distributieve rechtvaardigheid betreft de vraag of de baten en lasten van de preventiemaatregel rechtvaardig over partijen worden verdeeld.

– de vergelijkende rechtvaardigheid betreft de vraag of partijen in vergelijkbare situaties vergelijkbaar worden behandeld. Hierbij kan ook een vergelijking worden gemaakt tussen partijen die wel en niet (financieel of anderszins) door toepassing van de preventiemaatregel worden geraakt.
– de legitimiteit van de preventiemaatregel betreft de vraag of de preventiemaatregel niet strijdig is met het vigerende recht (wet- en regelgeving, nationale en internationale verdragen, rechtsgeldige overeenkomsten, e.d.).

Het criterium ‘rechtvaardigheid’ kent de volgende aspecten:

1. Aspect van verantwoordelijkheid
   In hoeverre worden de lasten van de preventiemaatregel over partijen verdeeld naar rato van hun verantwoordelijkheid voor het risico op schade?
2. Aspect van gelijke behandeling
   Worden partijen in gelijke gevallen gelijk behandeld (zonder rekening te houden met draagkracht)?
3. Aspect van draagkracht
   In hoeverre houdt de preventiemaatregel rekening met de draagkracht van mensen?
4. Aspect van hardheid in specifieke gevallen
   In hoeverre houdt de preventiemaatregel rekening met de persoonlijke omstandigheden van mensen of specifieke omstandigheden van organisaties?
5. Aspect van gelijke toegang tot gezondheidszorg en preventie
   Welke invloed heeft de preventiemaatregel op de gelijke kansen van betrokken partijen wat betreft de toegang tot gezondheidszorg en preventie?
6. Aspect van verdeling naar behoefte van gezondheidszorg en preventie
   Welke invloed heeft de preventiemaatregel op de verdeling van de voor gezondheidszorg en preventie beschikbare middelen naar behoefte van betrokken partijen?
7. Aspect van legitimiteit
   Is de preventiemaatregel niet strijdig met het vigerende recht (wet- en regelgeving, nationale en internationale verdragen, rechtsgeldige overeenkomsten, e.d.)?

Wijze van beoordeling
Beoordeeld wordt of toepassing van de preventiemaatregel vanuit het perspectief van de distributieve en vergelijkende rechtvaardigheid niet onredelijk of onbillijk is (aspecten 1 t/m 6) en niet strijdig is met het vigerende recht (aspect 7). Tevens wordt beoordeeld of een ander ontwerp van de preventiemaatregel niet rechtvaardiger zou zijn (optimalisatiebeginsel).

11. Draagvlak

Operationalisering van het criterium

Het criterium 'draagvlak' betreft de volgende vragen:

- In hoeverre is de tijd rijp om de beoogde leefstijlverandering na te streven door het toepassen van drang of dwang?
- In hoeverre zijn mensen en organisaties die door de preventiemaatregel worden geraakt geleidelijk op de preventiemaatregel voorbereid en krijgen zij de tijd om zich aan de nieuwe regels aan te passen?
- In hoeverre en op welke manier(en) is er aandacht besteed aan het verwerven van draagvlak voor de preventiemaatregel?
- In hoeverre zijn de actuele politieke, economische en maatschappelijke omstandigheden geschikt voor implementatie?
- In hoeverre sluit de preventiemaatregel aan op het beleid van stakeholders (bv. overheden, branchegenoten, cliëntenorganisaties, beroepsorganisaties en ideële organisaties)?
- Hoeveel draagvlak is er voor de preventiemaatregel?

Het gaat om het draagvlak bij alle betrokken partijen en in ieder geval bij de:

- doelgroep waarop de preventiemaatregel zich richt;
- belanghebbenden die worden beschermd tegen schade, hinder of overlast;
- partijen die de preventiemaatregel uitvoeren en/of handhaven;
- partijen die voor- of nadeel ondervinden van de preventiemaatregel.

Indien relevant gaat het ook om maatschappelijk draagvlak en politiek draagvlak.

Wijze van beoordeling

Beoordeeld wordt of:

- er voldoende draagvlak bestaat (bestond) bij de betrokken partijen voor toepassing van de preventiemaatregel, zodat de handhaving en uitvoering niet onnodig moeilijk en/of onevenredig kostbaar zijn (geweest);
- het draagvlak niet onnodig wordt (is) ondermijnd;
- er voldoende aandacht wordt (is) besteed aan het verwerven van draagvlak voor de preventiemaatregel (optimalisatiebeginsel).

12. Criterium flankerend beleid

Operationalisering van het criterium

Flankerend beleid is beleid bedoeld om de uitvoering van een maatregel of wet te ondersteunen. Dit kan zowel door stimulerende maatregelen als door het wegne-

men van belemmeringen. Bij de beoordeling van het flankerend beleid dient in ieder geval aandacht te worden besteed aan:

- inbedding van de preventiemaatregel in breder (preventie)beleid;
- afwezigheid van beleid dat strijdig is met de preventiemaatregel;
- voorlichting over de preventiemaatregel aan betrokken partijen;
- handhaving van de naleving van de preventiemaatregel;
- inventarisatie en oplossing van knelpunten (het gaat hierbij zowel om knelpunten die de implementatie van de preventiemaatregel belemmeren, als knelpunten ten gevolge van de preventiemaatregel).

In het kader van het model is handhaving gedefinieerd als handelingen gericht op bevordering van naleving van (rechts)regels en beëindiging van overtredingen van (rechts)regels. Het woord 'rechts' is tussen haakjes geplaatst, omdat het niet alleen gaat om de handhaving van wettelijk regels. Het kan bijvoorbeeld ook gaan om regels die een werkgever heeft vastgesteld. Het onderscheid tussen de begrippen naleving, handhaving, en toezicht op de handhaving, kan worden toegelicht aan de hand van het 'wettelijk rookverbod in de horeca':

- de 'naleving' betreft de mate waarin rokers zich in de horeca houden aan het wettelijk rookverbod;
- de 'handhaving' betreft de handhaving van het wettelijk rookverbod in de horeca door horeca-eigenaars en horecawerknemers;
- het 'toezicht op de handhaving' betreft het toezicht van de nieuwe Voedsel en Waren Autoriteit op de handhaving van het wettelijk rookverbod in de horeca. Van toezicht op de handhaving is in de praktijk vaak geen sprake. Toch acht ik het zinvol om ook vanuit dit perspectief naar de handhaving te kijken.

De invloed van sociale controle en gedoogbeleid op de naleving zijn aandachtspunten bij de beoordeling.

Wijze van beoordeling

Beoordeeld wordt of:

- de vormgeving van de preventiemaatregel de naleving waar mogelijk bevordert (heeft bevorderd) en niet onnodig ondermijnt (heeft ondermijnd);
- de vormgeving en uitvoering van het flankerend beleid niet onredelijk of onbillijk zijn (geweest);
- er geen sprake is (geweest) van onachtzaamheid of nalatigheid bij de vormgeving en uitvoering van het flankerend beleid (optimalisatiebeginsel).

13. Criterium controleerbaarheid

Operationalisering van het criterium
Voordat de preventiemaatregel wordt geïmplementeerd, is vaak niet met zekerheid te voorspellen hoe de 'effecten en neveneffecten' (effectiviteit, indringendheid, kosten-batenverhouding en rechtvaardigheid) en 'implementatie' (draagvlak, flankerend beleid en uitvoerbaarheid) zich zullen ontwikkelen. Als ontwikkelingen afwijken van de verwachtingen moet tijdig kunnen worden bijgestuurd. Dit is des te belangrijker naarmate de negatieve neveneffecten van de preventiemaatregel groter (kunnen) zijn. Het is echter ook mogelijk dat nieuwe ontwikkelingen vragen om aanpassing van de doelstelling van de preventiemaatregel. Daarom is het van belang om niet alleen de 'effecten en neveneffecten' en 'implementatie' van de preventiemaatregel te monitoren en/of (periodiek) te evalueren, maar ook het 'ontwerp'. De onafhankelijkheid c.q. objectiviteit van monitoring en evaluatie is belangrijk met het oog op de objectiviteit van de beschikbare stuurinformatie.

Wijze van beoordeling
Beoordeeld wordt of:
- de wijze, waarop het 'ontwerp', de 'effecten en neveneffecten' en 'implementatie' van de preventiemaatregel worden (zijn) gemonitord en/of geëvalueerd, niet onredelijk of onbillijk is (geweest);
- men zich voldoende inzet (heeft ingezet) om het 'ontwerp', de 'effecten en neveneffecten' en implementatie van de preventiemaatregel te monitoren en/of evalueren (optimalisatiebeginsel).

14. Criterium uitvoeringscapaciteit

Operationalisering van het criterium
De beoordeling van de preventiemaatregel aan de hand van het criterium 'uitvoeringscapaciteit' beperkt zicht tot de uitvoeringsaspecten die nog niet bij een voorafgaand criterium aan de orde zijn geweest. Daarom beperkt de beoordeling zich tot de vraag of er voldoende mensen en middelen beschikbaar zijn om de implementatie van de preventiemaatregel te rechtvaardigen. Het gaat hierbij zowel om de haalbaarheid als de houdbaarheid van de implementatie.

Wijze van beoordeling
Beoordeeld wordt of:
- voldoende mensen en middelen beschikbaar worden (zijn) gesteld om een adequate implementatie van de preventiemaatregel haalbaar en houdbaar (in de tijd) te maken;

- niet onredelijk weinig of onbillijk weinig mensen en middelen voor de uitvoering van de preventiemaatregel beschikbaar worden (zijn) gesteld. Het is immers denkbaar dat het beschikbaar stellen van net voldoende mensen en middelen wel onredelijk weinig is;
- bij de uitvoering van de preventiemaatregel geen sprake is (geweest) van onachtzaamheid of nalatigheid (optimalisatiebeginsel).

15.1.2 Structuur van het beoordelingsmodel

Het idee voor de zeefstructuur van mijn model heb ik ontleend aan de 'Trechter van Dunning'. Dit is het beoordelingsmodel dat in 1991 onder voorzitterschap van Dunning is ontwikkeld door de Commissie Keuzen in de Zorg. De eerste reden om gebruik te maken van het principe van een trechter met achtereenvolgende zeven, is dat de ***volgorde*** waarin de beoordelingscriteria worden gehanteerd binnen mijn model van grote invloed is op de inhoud en daarmee op de ***validiteit*** van het beoordelingsproces. Door de clustering van de beoordelingscriteria in zeven wordt inzichtelijker waarom de volgorde van de beoordelingscriteria essentieel is. De tweede reden om gebruik te maken van het principe van een trechter met achtereenvolgende zeven, is dat hierdoor het aantal van veertien criteria beter hanteerbaar wordt. Als een bepaalde zeef niet wordt gepasseerd, hoeven de achterliggende criteria immers niet meer te worden onderzocht. De derde reden om gebruik te maken van het principe van een trechter met achtereenvolgende zeven is de volgende. ***Criteria binnen een zeef mogen tegen elkaar worden gewogen.*** Dit betekent dat een goede score op een bepaald criterium een slechte score op een ander criterium kan compenseren. ***Criteria afkomstig uit verschillende zeven mogen niet tegen elkaar worden gewogen.*** Ter illustratie hiervan twee voorbeelden:
- als preventie niet noodzakelijk (1e zeef) is, is het niet relevant om de effectiviteit (2e zeef) nog te beoordelen;
- als de preventiemaatregel niet effectief (2e zeef) is, is implementatie (3e zeef) van de preventiemaatregel niet meer aan de orde.

Overzicht 26: zeefstructuur van het beoordelingsmodel

preventiemaatregel

↓

Logica van ontwerp (1e zeef)
(criteria 1 t/m 6)

↓

Effecten en neveneffecten (2e zeef)
(criteria 7 t/m 10)

↓

Implementatie (3e zeef)
(criteria 11 en 14)

↓

Gerechtvaardigde preventiemaatregel

Toelichting op de volgorde van de beoordelingscriteria (§3.3)

Het ontwikkelde beoordelingsmodel is een argumentatief model. Ten behoeve van de rationaliteit van de beoordeling dient binnen het model zoveel mogelijk gebruik te worden gemaakt van wetenschappelijke gegevens ter onderbouwing van de argumentaties. Met het oog op de objectiviteit, vergelijkbaarheid en generaliseerbaarheid van de beoordeling, verdient het gebruik van kwantitatieve onderzoeksgegevens hierbij de voorkeur boven het gebruik van kwalitatieve onderzoeksgegevens. In overzicht 27 wordt mijn visie op de juiste volgorde van de argumentatie in een rationeel beoordelingsproces schematisch weergegeven.

Overzicht 27: fasering van de argumentatie in een rationeel beoordelingsproces

1. Argumenten op basis van *kwantitatieve* wetenschappelijke onderzoeksgegevens

↓

2. Argumenten op basis van *kwalitatieve* wetenschappelijke onderzoeksgegevens

↓

3. Niet-wetenschappelijk-bewezen argumenten die niet in strijd zijn met wetenschappelijke onderzoeksgegevens of met de logica.

Voor het kwantificeren c.q. uitdrukken in geld van immateriële zaken - zoals overlast, inbreuk op het persoonlijk leven en schending van morele opvattingen – kan gebruik worden gemaakt van concepten als 'Willingness to Pay' en Willingness to Accept' (zie §6.6.1).

Niet-wetenschappelijk bewezen argumenten zijn bijvoorbeeld argumenten van morele of politiek-ideologische aard, zoals het verbieden van harddrugsgebruik omdat men harddrugsgebruik moreel verwerpelijk acht.

De volgorde van de beoordelingscriteria in mijn model wordt bepaald door twee ordeningsprincipes:
– de fasering van de argumentatie van een rationeel beoordelingsproces, zoals weergegeven in overzicht 27;
– de logische volgorde van de beoordelingscriteria vanuit inhoudelijk perspectief.

Voor de rationaliteit van het beoordelingsmodel acht ik 'de logische volgorde vanuit inhoudelijk perspectief' het belangrijkst. Om aan beide ordeningsprincipes tegemoet te komen, heb ik de criteria van mijn model geclusterd:
– de volgorde van de clusters en de volgorde van de criteria binnen ieder cluster is gebaseerd op de logische volgorde vanuit inhoudelijk perspectief;
– de criteria binnen een cluster hangen inhoudelijk logisch samen;
– binnen de clusters is gestreefd naar een volgorde van de criteria conform overzicht 27. Dit streven heeft er bijvoorbeeld toe geleid dat ik in cluster 1 het criterium 'noodzakelijkheid' heb opgesplitst in een wetenschappelijk te objectiveren criterium 'schadelijkheid' en een wetenschappelijk niet te objectiveren criterium 'noodzakelijkheid'[2].

Impliciet kent mijn model 7 clusters van 2 criteria:
– Cluster 1:
  1. Schadelijkheid
  2. Noodzakelijkheid
– Cluster 2:
  3. Oorzakelijkheid
  4. Verantwoordelijkheid
– Cluster 3:
  5. Gerichtheid

2. In tegenstelling tot in het model is bijvoorbeeld in de Trechter van Dunning de 'schadelijkheid' geen afzonderlijk criterium, maar onderdeel van het criterium 'noodzakelijkheid'.

6. Geschiktheid
- Cluster 4:
  7. Effectiviteit
  8. Indringendheid
- Cluster 5:
  9. Lasten-batenverhouding
  10. Rechtvaardigheid
- Cluster 6:
  11. Draagvlak
  12. Flankerend beleid
- Cluster 7:
  13. Controleerbaarheid
  14. Uitvoeringscapaciteit

De indeling van mijn beoordelingsmodel in 7 clusters van elk 2 criteria heb ik gebruikt voor het bepalen van de juiste volgorde van de beoordelingscriteria bij de bouw van het model. Om het model voor de gebruiker niet te complex te maken, heb ik ervoor gekozen om deze clusters in het model niet expliciet zichtbaar te maken. Door de ***dwingende structuur*** van het beoordelingsmodel – de criteria dienen in de gegeven vaste volgorde te worden beoordeeld – wordt de beoordelaar geholpen bij het beoordelen van de logische samenhang tussen de beoordelingscriteria.

15.1.3 Wijze van beoordeling

Gerechtvaardigdheid van de argumentatie

De gerechtvaardigdheid van de toepassing van een preventiemaatregel berust op twee pijlers:

- de gerechtvaardigdheid van de ***argumentatie*** die heeft geleid tot het besluit om de preventiemaatregel toe te passen;
- de gerechtvaardigdheid van de ***procedure*** (welke partijen kunnen op welk moment welke invloed uitoefenen op de besluitvorming?) die heeft geleid tot het besluit om de preventiemaatregel toe te passen.

Mijn beoordelingsmodel richt zich op de gerechtvaardigdheid van de argumentatie. Afhankelijk van de casus dient per criterium te worden afgewogen in hoeverre procedurele aspecten relevant zijn voor de inhoudelijke beoordeling van de gerechtvaardigdheid van de preventiemaatregel. Op zichzelf valt de beoordeling van de procedurele gerechtvaardigdheid buiten het kader van mijn model (zie §12.4.4).

In pluralistische samenlevingen is het niet mogelijk om *toetsings*criteria voor de beoordeling van de gerechtvaardigdheid van leefstijlbeïnvloedende preventiemaatregelen vast te stellen die algemeen worden aanvaard. Om niet de suggestie te wekken dat de gerechtvaardigdheid van preventiemaatregelen met behulp van mijn model wordt getoetst aan de hand van harde, meetbare criteria, spreek ik in het kader van mijn model over de beoordeling van de gerechtvaardigdheid en niet over de toetsing van de gerechtvaardigdheid. (Zie hoofdstuk 4)

Bij de beoordeling van de gerechtvaardigdheid van een preventiemaatregel worden in het kader van mijn model de ***redelijkheid en billijkheid*** van de argumentatie die ten grondslag ligt aan de toepassing van de preventiemaatregel beoordeeld. De begrippen redelijkheid en billijkheid zijn moeilijk strikt van elkaar te scheiden. De redelijkheid van de argumentatie refereert vooral aan de rationaliteit van de argumentatie (rede = ratio = verstand). De billijkheid van de argumentatie refereert vooral aan de 'fairheid' van de argumentatie (billijk = fair = eerlijk).

Redelijkheid en billijkheid van de argumentatie

Per criterium van mijn model worden de redelijkheid en billijkheid van de argumentatie die ten grondslag ligt aan de toepassing van de preventiemaatregel, beoordeeld. Deze beoordeling is eigenlijk een zoektocht naar falsificatie van de redelijkheid en billijkheid van de volgende aspecten van de argumentatie (zie §4.5):

1. ***Juistheid*** van de argumentatie (is de argumentatie niet strijdig met wetenschappelijke informatie of onlogisch?);
2. ***Volledigheid*** van de argumentatie (is geen beschikbare wetenschappelijke informatie en zijn geen relevante argumenten over het hoofd gezien of genegeerd?);
3. Kracht van het ***bewijs*** (is het bewijs voor de argumentatie sterk genoeg?);
4. ***Transparantie*** van het bewijs (is de argumentatie voor iedereen vrij en eenvoudig toegankelijk?);
5. ***Interne consistentie*** van de argumentatie (voldoet de preventiemaatregel aan de vastgestelde samenhang tussen de criteria van het model?);
6. Verhouding tot het ***schadebeginsel*** (is de preventiemaatregel niet moralistisch, perfectionistisch, of paternalistisch?)
7. Mogelijkheid van ***betere alternatieven*** (zijn geen betere alternatieven voor de vormgeving, het ontwerp, of de implementatie van de preventiemaatregel mogelijk?).

Optimalisatiebeginsel

Als de argumentatie ter onderbouwing van de preventiemaatregel m.b.t. de aspecten 1 t/m 6 ernstig tekortschiet, is toepassing van de preventiemaatregel niet

gerechtvaardigd. Als de argumentatie ter onderbouwing van de preventiemaatregel m.b.t. het optimalisatiebeginsel (7e aspect) tekortschiet, betekent dat niet automatisch dat toepassing van de preventiemaatregel niet gerechtvaardigd is. Dat het ontwerp (1e zeef), de effecten en neveneffecten (2e zeef) of de implementatie (3e zeef) van de preventiemaatregel suboptimaal zijn, hoeft immers niet te betekenen dat deze niet goed genoeg zijn. Soms is een suboptimale toepassing van een suboptimaal ontworpen preventiemaatregel te verkiezen boven het helemaal niet toepassen van de preventiemaatregel: ***beter een half ei dan een lege dop***. Als suboptimaliteit wordt geconstateerd is dat altijd een aansporing tot verbetering. Door bij het beoordelen van de gerechtvaardigdheid van de preventiemaatregel aan de hand van het optimalisatiebeginsel uit te gaan van de gegeven mogelijkheden (bv. de beschikbare mensen en middelen) tracht ik een perfectionistische invulling van de beoordeling te voorkomen.

Schadebeginsel

Vrij vertaald stelt het schadebeginsel dat de vrijheid van mensen alleen mag worden ingeperkt om te voorkomen dat zij schade berokkenen aan derden. Dit is een zeer liberaal uitgangspunt, dat veel mensen te ver gaat. Ik ga uit van een ***relatieve opvatting van het schadebeginsel***. Dit betekent dat ik paternalistische en moralistische vrijheidsbeperkingen niet rücksichtslos afwijs, maar enkel stel dat deze minder sterk gerechtvaardigd zijn dan vrijheidsbeperkingen ter voorkoming van schade. Op basis van een relatieve opvatting van het schadebeginsel kom ik tot de volgende ***algemeen aanvaarde uitgangspunten*** voor de beoordeling van de gerechtvaardigdheid van vrijheidsbeperkingen (zie §4.3.5):

- Preventie van schade aan rechtmatige belangen vormt een sterkere rechtvaardiging van een bepaalde leefstijlbeïnvloedende interventie dan bevordering van rechtmatige beleidsdoelen;
- Preventie van schade of overlast vormt een sterkere rechtvaardiging van een bepaalde leefstijlbeïnvloedende interventie dan preventie van de schending van morele opvattingen;
- Preventie van schade die men berokkent aan derden vormt een sterkere rechtvaardiging van een bepaalde leefstijlbeïnvloedende interventie dan preventie van schade die men berokkent aan zichzelf (als het gaat om ‘dezelfde’ schade);
- Preventie van schade die kinderen berokkenen aan zichzelf vormt een sterkere rechtvaardiging van een bepaalde leefstijlbeïnvloedende interventie dan preventie van schade die volwassenen berokkenen aan zichzelf (als het gaat om ‘dezelfde’ schade);
- Naarmate de autonomie, keuzevrijheid, wilsbekwaamheid en geïnformeerdheid van mensen groter is, is paternalisme minder gerechtvaardigd.

Gerechtvaardigdheid van de implementatie
De beoordeling van de gerechtvaardigdheid van de implementatie van de preventiemaatregel (3e zeef van het model) ***leunt zwaar op het optimalisatiebeginsel***. Op het moment dat de preventiemaatregel wordt beoordeeld aan de hand van de 3e zeef (implementatie), is deze de 1e en 2e zeef al gepasseerd. Dit betekent dat de logica van het ontwerp (1e zeef) en de effecten en neveneffecten (2e zeef) in orde zijn bevonden (zie §15.1.8). Als bij de beoordeling van de 'effecten en neveneffecten' de gevolgen van de implementatie zijn meegewogen, impliceert dit dat de implementatie niet ongerechtvaardigd is. Wel is het mogelijk dat de implementatie suboptimaal plaatsvindt of heeft plaatsgevonden. Zoals hierboven toegelicht, betekent een suboptimale implementatie niet dat de preventiemaatregel dan beter helemaal niet meer kan worden toegepast (zie §15.1.9). Wel is het een aansporing tot verbetering.

Retrospectieve beoordeling (evaluatie)
Bij een retrospectieve beoordeling wordt teruggekeken naar de effecten en neveneffecten (2e zeef) en implementatie (3e zeef) van de preventiematregel, zoals die daadwerkelijk hebben plaatsgevonden. Een retrospectieve beoordeling kan daarom alleen tijdens of na afloop van de implementatie van een preventiemaatregel plaatsvinden. In de effecten en neveneffecten die zijn opgetreden, zit de invloed van de implementatie automatisch verwerkt. Als de beoordeling een periode na afloop van de implementatiefase betreft, kunnen de criteria van de 3e zeef (implementatie) nog steeds relevant zijn voor de beoordeling. (zie §13.7.2)

Prospectieve beoordeling (voorspelling)
Bij een prospectieve beoordeling wordt vooruitgekeken naar de effecten en neveneffecten (2e zeef) en implementatie (3e zeef) van de preventiematregel, die nog moeten plaatsvinden. Een prospectieve beoordeling kan alleen plaatsvinden op basis van aannames over de 'effecten en neveneffecten' en 'implementatie' van de preventiemaatregel. Het werken met aannames biedt de mogelijkheid om te werken met implementatiescenario's (bijvoorbeeld veel versus weinig inspanning op het gebied van 'handhaving'). Ook is het mogelijk om via experimenten of pilotstudies de 'effecten en neveneffecten' en 'implementatie' van een preventiemaatregel uit te proberen en te onderzoeken. (zie §13.7.2)

15.2 Beschouwing van mijn onderzoek en beoordelingsmodel

15.2.1 Objectiviteit en volledigheid

Modelontwikkeling aan de hand van bestaande modellen

Bij de ontwikkeling van mijn model heb ik zo goed mogelijk gebruik gemaakt van bestaande beoordelingsmodellen, die relevant zouden kunnen zijn voor het door mij beoogde model (zie §2.6, 2.7 en 4.2):

- de criteria van Wilson en Jungner voor beslissingen over de aanvaardbaarheid van screening van de bevolking op aandoeningen (1968);
- de Trechter van Dunning voor pakketbeslissingen (1991);
- de criteria van de Gezondheidsraad voor de aanvaardbaarheid van genetische screening (1994);
- een model over de gerechtvaardigdheid van public health interventies (Gostin, 2000);
- de criteria van Childress e.a. voor de rechtvaardiging van preventiemaatregelen in de public health (2002);
- een model – de Preffi – voor het effectief managen van gezondheidsbevordering (Molleman, 2003, 2005, 2006);
- de criteria van het CVZ voor pakketbeslissingen (Mastenbroek, 2006);
- een model van de RVZ voor pakketbeslissingen (2006);
- de aanbevelingen van Mello voor het ontwerp van gelegitimeerde 'incentive programs' in de public health (2008).

De negen modellen hebben gedeeltelijk een andere doelstelling. Van ***al deze modellen*** ben ik nagegaan of ik een of meer criteria zou moeten overnemen in het door mij beoogde model. Deze werkwijze vormt tot op zekere hoogte een waarborg voor de objectiviteit van de modelontwikkeling en voor de volledigheid (theoretische verzadiging) van het model. Met het oog op de volledigheid van het model, is het van belang dat het model van de RVZ is gebaseerd op een ***internationaal vergelijkende studie***, waarbij modellen met elkaar zijn vergeleken die zijn ontwikkeld in verschillende landen.

In juni 2010 promoveerde Marieke ten Have op een proefschrift waarin zij een ethisch kader voor de beoordeling van preventiemaatregelen ter bestrijding van overgewicht en obesitas heeft ontwikkeld (Have ten, 2011). Hoofdstuk 4 van dit proefschrift bevat een overzicht van zes ethische kaders ('*ethical frameworks*') op het gebied van publieke gezondheidszorg, die op basis van een uitgebreid literatuuronderzoek zijn gevonden, en waarover reeds in oktober 2010 wetenschappelijk is gepubliceerd (Have ten, 2010). Van deze zes ethische kaders heb ik er twee

gebruikt bij de ontwikkeling van mijn model: het ethische kader van Childress e.a. (zie §4.2) en dat van Kass (zie §4.4.3). De beoordelingscriteria (*'main ethical values'*) van de zes genoemde ethische kaders komen inhoudelijk wel grotendeels overeen met de criteria van mijn model (al is soms een andere formulering gekozen). Het overzicht bevat geen criteria die niet onder een criterium van mijn model kunnen worden ondergebracht. Zo bezien vormt dit overzicht een bevestiging van de juistheid en volledigheid van de criteria van mijn model. Het overzicht bevat ook enkele criteria die ik onvoldoende waardeneutraal acht om als (sub)criterium in mijn model op te nemen. Dit geldt met name voor de criteria *'cultural value pluralism; communitarianism; sustainability; social responsibility'* (Have ten, 2011, p.66).

De kern van het ethische kader dat Ten Have heeft ontwikkeld bestaat uit acht vragen (*'ethical pitfalls'*) (p.86):

- *How does the program affect physical health?*
- *How does the program affect psychosocial well-being?*
- *How does the program affect equality?*
- *How does the program affect informed choice?*
- *How does the program affect social and cultural values?*
- *How does the program affect privacy?*
- *How does the program affect the attribution of responsibilities?*
- *How does the program affect liberty?*

Met uitzondering van de criteria *'informed choice'* en *'social and cultural values'* zijn deze criteria ook onderdeel van mijn model. De reikwijdte van mijn model is beperkt tot leefstijlbeïnvloedende preventiemaatregelen waarbij sprake is van drang of dwang (zie §15.1.7). Daarom is *'informed choice'* geen (sub)criterium in mijn model. Wel is 'beperking van de keuzevrijheid' onderdeel van het criterium 'indringendheid' van mijn model. Het criterium *'social and cultural values'* acht ik onvoldoende waardeneutraal om als (sub)criterium in mijn model op te nemen. In het model van Ten Have wordt respect voor sociale en culturele waarden positief gewaardeerd en aantasting van deze waarden negatief (p.88 en 92). Volgens mij verdienen niet alle sociale en culturele waarden automatisch een positieve waardering en soms kan juist het ter discussie stellen van deze waarden positief worden gewaard. Denk bijvoorbeeld aan de achterstelling van vrouwen of de afwijzing van homofilie in bepaalde culturen. Aan het risico op stigmatisering wijdt Ten Have een afzonderlijk hoofdstuk in haar proefschrift. Het risico op stigmatisering is onderdeel van het criterium 'indringendheid' van mijn model.

Toetsing van het model aan casuïstiek uit de praktijk
De juistheid, volledigheid en praktische toepasbaarheid van mijn model heb ik getoetst aan twee cases uit de praktijk: het preventieplan van Clarian Health (hoofdstuk 5 t/m 9) en het wettelijk rookverbod in de horeca (hoofdstuk 10 t/m 14). Een andere mogelijkheid was geweest om deskundigen en belanghebbenden, bijvoorbeeld via interviews of enquêtes, te bevragen over de juistheid, volledigheid en praktische toepasbaarheid van het model. In overleg met de begeleiders van mijn onderzoek heb ik hier bewust niet voor gekozen. Bij het bevragen van deskundigen en belanghebbenden over een nieuw model is het risico namelijk groot dat de toetsing (de vragenlijst) zich vormt naar de inhoud en vorm van het nieuwe model. De toetsing van de juistheid, volledigheid en praktische toepasbaarheid van het model aan door anderen aangevoerde argumenten, die niet ten behoeve van mijn onderzoek op schrift zijn gesteld, heeft bijgedragen aan de objectiviteit van de toetsing.

Van ***alle argumenten*** die ik heb aangetroffen in de door mij gescreende teksten over het preventieplan van Clarian Health (zie hoofdstuk 5) en over het wettelijk rookverbod in de horeca (zie §10.2) heb ik getoetst of deze onder een criterium van mijn model konden worden ondergebracht. Als dat niet het geval was, heb ik voor het betreffende argument een criterium aan het model toegevoegd. Op deze wijze heb ik gestreefd naar volledigheid van de criteria van het model. Op basis van de toetsing van het model aan de casus Clarian Health heb ik het criterium 'timing' aan het model toegevoegd. Op basis van de toetsing aan de casus 'wettelijk rookverbod in de horeca' heb ik de criteria 'handhaafbaarheid' en 'controleerbaarheid' toegevoegd en heb ik het criterium 'uitvoerbaarheid' hernoemd tot het criterium 'uitvoeringscapaciteit'. Het criterium 'timing' heb ik uiteindelijk samengevoegd met het criterium 'draagvlak' en het criterium 'handhaafbaarheid' met het criterium 'flankerend beleid' (zie §13.7.2).

Representativiteit van teksten over preventieplan van Clarian Health
De teksten over het preventieplan van Clarian Health waaraan ik de juistheid volledigheid en praktische toepasbaarheid van het model heb getoetst, kunnen worden gezien als een steekproef uit alle opvattingen onder de Amerikaanse bevolking over het preventieplan van Clarian Health. In deze teksten voert kritiek op dit preventieplan de boventoon. Waarschijnlijk is de reden hiervan dat op de weblogs waarvan ik de teksten heb gebruikt, vooral mensen hebben gereageerd die boos waren over dit preventieplan. Boos omdat zij zich vanwege hun eigen ongezonde leefstijl gediscrimineerd en/of (financieel) in het nauw gedreven voelden (dit geldt bijvoorbeeld voor *"the fat acceptance weblog":* www.bigfatblog.com) en/of omdat zij met het oog op de privacy en/of keuzevrijheid van mensen tegen inmenging van

derden (werkgevers) in het persoonlijk leven van mensen (werknemers) zijn. Zo bezien is het risico groot dat er sprake is van een ***'scheve steekproef'*** uit de opvattingen onder de bevolking over het preventieplan van Clarian. Voor de toetsing van het model was dit in zoverre geen bezwaar, dat deze teksten focussen op het meest risicovolle onderdeel van leefstijlbeïnvloedende interventies: de binnendringing van de preventiemaatregel in het persoonlijk leven van mensen. Hierdoor waren de reacties op het preventieplan van Clarian Health zeer bruikbaar bij het ***operationaliseren van het criterium 'indringendheid'*** (zie hoofdstuk 7). Hoewel de casus 'wettelijk rookverbod in de horeca' een periode van ruim 25 jaar beslaat, en de casus 'Clarian Health' een periode van slechts ongeveer 2 jaar, heeft de casus 'Clarian Health' veel meer informatie over het criterium 'indringendheid' opgeleverd dan de casus 'wettelijk rookverbod in de horeca'.

Representativiteit van teksten over wettelijk rookverbod in horeca

De teksten over het wettelijk rookverbod in de horeca, waaraan ik de juistheid, volledigheid en praktische toepasbaarheid van het model heb getoetst, zijn in de meeste gevallen afkomstig uit vergaderstukken van de Tweede en Eerste Kamer (inclusief Kamercommissies) over de totstandkoming en toepassing van dit rookverbod (zie §10.2 voor een overzicht van de herkomst van de door mij gebruikte teksten). Deze teksten zijn m.i. ***representatief*** voor het politieke debat over het wettelijk rookverbod in de horeca, omdat middels een (relatief) objectieve, deskundige en volledige verslaglegging (vergaderstukken van de Tweede en Eerste Kamer) de argumenten van regerings- en oppositiepartijen en van voor- en tegenstanders van het wettelijk rookverbod in de horeca, over een lange periode, op schrift zijn gesteld.

Volledigheid en objectiviteit van de argumentatie

De documentatie door de rijksoverheid van het politieke debat over het wettelijk rookverbod in de horeca is uitgebreid en degelijk. Daarom vormt niet de inhoud van deze documenten, maar vormen mijn inventarisatie, beschrijving en analyse van de argumenten uit deze documenten het grootste risico voor de objectiviteit van de modelontwikkeling en volledigheid van het model. Als onderzoeker heb ik daarom steeds getracht om alle aangevoerde argumenten zo volledig mogelijk te inventariseren en zo objectief mogelijk te beschrijven en analyseren. Als waarborg voor de objectiviteit van mijn beschrijving en analyse van argumenten heb ik ervoor gekozen om de in teksten gevonden argumenten niet alleen in eigen woorden samen te vatten, maar om zoveel mogelijk de ***citaten*** te laten zien, waaruit ik de betreffende argumenten heb gedistilleerd. Soms werd eenzelfde argument in veel verschillende citaten aangevoerd en heb ik ter illustratie van het argument een of enkele citaten gekozen. Bij deze keuze heb ik mij niet laten leiden door de her-

komst van de citaten (van welke persoon is het citaat afkomstig), maar door de inhoud van de citaten (welk citaat geeft het argument het duidelijkst weer). Door zo te werk te gaan, heb ik getracht mijn inventarisatie en analyse van argumenten zo ***transparant*** mogelijk en daarmee zo ***controleerbaar*** mogelijk te maken.

15.2.2 Neutraliteit

De gerechtvaardigdheid van een preventiemaatregel kan aan de hand van mijn model op twee manieren worden beoordeeld:
- een waardeneutrale beoordeling[3];
- een beoordeling gebaseerd op een opvatting, overtuiging of ideologie (een niet-waardeneutrale beoordeling).

In geval van een waardeneutrale beoordeling wordt uitsluitend beoordeeld of de argumentatie die ter rechtvaardiging van de preventiemaatregel wordt aangevoerd volledig is en niet (op basis van wetenschappelijke informatie of logische argumenten) gefalsificeerd kan worden. De beoordelaar dient zich ***strikt objectief*** op te stellen. Een niet-waardeneutrale beoordeling kent twee onderdelen. Enerzijds wordt beoordeeld of de argumentatie die ter rechtvaardiging van de preventiemaatregel wordt aangevoerd volledig is en niet (op basis van wetenschappelijke informatie of logische argumenten) gefalsificeerd kan worden. Anderzijds wordt beoordeeld of de argumentatie die ter rechtvaardiging van de preventiemaatregel wordt aangevoerd in overeenstemming is met de opvatting, overtuiging of ideologie van de beoordelaar of diens opdrachtgever. Bij beide onderdelen van de beoordeling (waardeneutraal en niet-waardeneutraal) dient de beoordelaar zich ***strikt objectief*** op te stellen.

Ten behoeve van het algemeen belang (praktijk, beleid en wetenschap) acht ik een ***waardeneutrale beoordeling het waardevolst***, omdat deze voor (bijna) iedereen aanvaardbaar is. Een waardeneutrale beoordeling is ook het moeilijkst, omdat een kenmerk van een beoordeling is dat deze op waarden (en normen die zijn afgeleid van deze waarden) gebaseerd is. Daarom heb ik bij de toetsing van de juistheid, volledigheid en praktische toepasbaarheid van het model gekozen voor een waardeneutrale beoordeling van de gerechtvaardigdheid van het preventieplan van Cla-

3. Duidelijker vind ik de spelling 'waardenneutraal' met tweemaal de letter 'n'. Het woordenboek omschrijft het woord 'waardevrij' als volgt (Van Dale, 2005): *"vrij van waardeoordelen: waardevrije wetenschap, wetenschap waarbij de onderzoeker zijn onderzoeksresultaten gescheiden houdt van zijn morele, culturele, sociaal-politieke waardeoordelen daaromtrent."* De correcte spelling lijkt mij daarom 'waardeneutraal'.

rian Health en van het wettelijk rookverbod in de horeca. De toetsing van het model aan deze casuïstiek uit de praktijk heeft aangetoond dat met behulp van het model een waardeneutrale beoordeling van de gerechtvaardigdheid van preventiemaatregelen ***mogelijk*** is.

Belangrijk is om op te merken dat een waardeneutrale beoordeling iets anders is dan een waardevrije beoordeling. Onder een waardevrije beoordeling versta ik een wijze van beoordeling waarbij het beoordelingskader vrij is van morele en politiek-ideologische opvattingen. Denk bijvoorbeeld aan natuurkundige metingen van snelheid en temperatuur. Onder een waardeneutrale beoordeling versta ik een wijze van beoordeling waarbij de morele en/of politiek-ideologische opvattingen die onderdeel uitmaken van het beoordelingskader algemeen aanvaard zijn. Dit betekent dat de morele en politiek-ideologische uitgangspunten van de beoordeling niet ter discussie staan. De uitkomst van de beoordeling is wel voor discussie vatbaar, omdat deze kan worden beïnvloed door de morele en/of politiek-ideologische opvattingen van de beoordelaar.

Voor een waardeneutrale beoordeling van preventiemaatregelen aan de hand van het model maak ik gebruik van ***algemeen aanvaarde waarden c.q. algemeen aanvaarde beoordelingsprincipes.*** Deze heb ik beschreven in §4.5. In §13.3.1 heb ik het 'optimalisatiebeginsel' (geen onachtzaamheid en geen nalatigheid) als algemeen aanvaarde waarde c.q. algemeen aanvaard beoordelingsprincipe aan het model toegevoegd.

15.2.3 Validiteit

De validiteit van het model betreft de vraag of men aan de hand van het model tot een algemeen aanvaardbare beoordeling van de gerechtvaardigdheid van iedere 'leefstijlbeïnvloedende preventiemaatregel ter bestrijding van leefstijlgerelateerde aandoeningen' kan komen.

De gerechtvaardigdheid van de te beoordelen preventiemaatregel kan ik niet uitrekenen, omdat door verschillende mensen een verschillende invulling aan het begrip 'gerechtvaardigdheid wordt gegeven. Het beste wat ik kan bieden is een ***fair beoordelingsproces*** (zie §4.1). Ik heb mij hierbij beperkt tot de redelijkheid en billijkheid van de argumentatie ter onderbouwing van de besluitvorming. De redelijkheid en billijkheid van de besluitvormingsprocedure heb ik buiten beschouwing gelaten voor zover deze niet van (grote) invloed is op de argumentatie ter onderbouwing van de besluitvorming (zie §12.4.4).

Objectiviteit van de modelontwikkeling, volledigheid van het model en neutraliteit van de wijze van beoordeling zijn voorwaarden voor een fair beoordelingsproces en daarmee voorwaarden voor de validiteit van het beoordelingsmodel. In §15.1.1 en 15.1.2 heb ik betoogd waarom het volgens mij aannemelijk is, dat het model aan deze voorwaarden voldoet.

Met het model heb ik getracht om een fair argumentatief beoordelingsproces te ontwerpen met als uitgangspunten redelijkheid en billijkheid en transparantie van de argumentatie. Of het door mij ontworpen beoordelingsproces ook leidt tot ***algemeen aanvaardbare uitkomsten*** van de beoordeling van de gerechtvaardigdheid van de preventiemaatregel, kan het best worden onderzocht door veel verschillende preventiemaatregelen aan de hand van het model te beoordelen. De uitkomsten moeten dan steeds algemeen aanvaardbaar zijn.

De aanvaardbaarheid voor mijzelf en mijn begeleiders van de uitkomsten van de beoordeling van het preventieplan van Clarian Health en het wettelijk rookverbod in de horeca aan de hand van het model geven een ***indicatie van de validiteit*** van het model. Om meer zekerheid te krijgen over de validiteit van het model, dienen meer preventiemaatregelen aan de hand van het model te worden beoordeeld. De uitkomsten moeten steeds door verschillende mensen worden beoordeeld om meer zekerheid te krijgen over de algemene aanvaardbaarheid.

15.2.4 Betrouwbaarheid

De betrouwbaarheid van het model betreft de vraag of waardeneutrale beoordeling (zie §15.1.2) van dezelfde preventiemaatregel door verschillende beoordelaars steeds tot dezelfde uitkomsten leidt. Tevens dienen alle mogelijke preventiemaatregelen betrouwbaar te kunnen worden beoordeeld. Om met (meer) zekerheid een uitspraak te kunnen doen over de betrouwbaarheid van het model, dienen veel verschillende preventiemaatregelen door veel verschillende beoordelaars te zijn beoordeeld. Dat is nu nog niet het geval.

Dat de beoordeling aan de hand van het model is gebaseerd op de ***falsificatie van aangevoerde argumenten*** en niet op het bewijzen van de juistheid van aangevoerde argumenten, vormt tot op zekere hoogte een waarborg voor de betrouwbaarheid van de beoordelingen aan de hand van het model. Bij falsificatie kan men immers terugvallen op algemeen aanvaarde argumenten, zoals feitelijke onjuistheid, onvolledigheid en logische redeneerfouten. Dergelijke falsificaties zijn algemeen aanvaardbaar en dat beperkt de interbeoordelaarsvariatie. Bij het beoordelen van de juistheid van aangevoerde argumenten, ontkomt men er niet aan om (per-

soonlijke) morele en/of politiek-ideologische opvattingen te laten meewegen bij de beoordeling. Dat zou de interbeoordelaarsvariatie (sterk) vergroten.

Voor zover mij bekend, wordt de 'indringendheid' van leefstijlbeïnvloedende preventiemaatregelen in de praktijk en in theorie meestal niet gekwantificeerd. In dat geval is alleen een kwalitatieve beoordeling van de indringendheid mogelijk en een kwalitatieve beoordeling is in het algemeen minder objectief en dus minder betrouwbaar dan (de combinatie van een kwalitatieve en) een kwantitatieve beoordeling (zie §3.3).

15.2.5 Praktische toepasbaarheid

'The proof of the pudding is in the eating' geldt ook voor het onderzoeken van de praktische toepasbaarheid van een beoordelingsmodel. Veelgehoorde kritiek op de 'Trechter van Dunning' is dat dit model in de praktijk moeilijk toepasbaar is. Daarom heb ik ervoor gekozen om daadwerkelijk twee praktijkcases aan de hand van het model te beoordelen.

Het daadwerkelijk uitvoeren van de beoordeling heeft ***veel nieuwe inzichten voor en aanpassingen van*** het model opgeleverd, waarvan ik de belangrijkste nog eens noem:
- het niet opnemen van een afzonderlijk criterium 'kosteneffectiviteit' in het model (§12.3.1);
- het toevoegen van de criteria 'timing (§7.5.3), flankerend beleid (§9.2), handhaafbaarheid (§10.4) en controleerbaarheid (§10.4)' aan het model[4];
- de introductie van het falsificatiebeginsel als uitgangspunt voor de beoordeling (§4.3.6);
- de introductie van het 'optimalisatiebeginsel' (geen onachtzaamheid en geen nalatigheid) als uitgangspunt voor de beoordeling (§13.3.1).

In de hoofdstukken 5 t/m 14 is het gelukt om aan de hand van het model tot een daadwerkelijke beoordeling van de gerechtvaardigdheid van praktijkcases te komen. Op basis hiervan acht ik het ***aannemelijk*** dat het model praktisch toepasbaar is. De beoordeling van de casus 'Clarian Health' aan de hand van het model vormt een aanwijzing dat het model praktisch bruikbaar is voor ***veldpartijen*** die een leefstijlbeïnvloedende preventiemaatregel willen toepassen of evalueren. De

4. Uiteindelijk heb ik het criterium 'timing' samengevoegd met het criterium 'draagvlak en heb ik het criterium 'handhaafbaarheid' samengevoegd met het criterium 'flankerend beleid' (zie §13.7.2).

beoordeling van de casus 'wettelijk rookverbod in de horeca' aan de hand van het model vormt een aanwijzing dat het model praktisch bruikbaar is voor ***overheden*** die een leefstijlbeïnvloedende preventiemaatregel willen toepassen of evalueren.

15.2.6 Algemene geldigheid

Ik heb mij ten doel gesteld een model te ontwikkelen dat algemeen geldig en toepasbaar is. Dit betekent dat het model praktisch toepasbaar is voor alle mogelijke preventiemaatregelen ter bestrijding van leefstijlgerelateerde aandoeningen in alle mogelijke contexten (dus ook internationaal).

Generaliseerbaarheid van de casus Clarian Health

Met het oog op de ***internationale bruikbaarheid*** van het model (onafhankelijkheid van de culturele context) is het een voordeel dat de casus 'Clarian Health' het preventieplan van een Amerikaanse werkgever betreft en dat de ontwikkeling en (voorgenomen) implementatie van een dergelijk preventieplan in Nederland (op de korte termijn) onwaarschijnlijk is. Met het oog op de bruikbaarheid van het model voor de beoordeling van preventiemaatregelen ter bestrijding van ***alle mogelijke leefstijlgerelateerde aandoeningen*** is het een voordeel dat de casus 'Clarian Health' betrekking heeft op diverse leefstijlen en diverse leefstijlgerelateerde aandoeningen (roken, ernstig overgewicht, te hoge bloeddruk, te hoog cholesterolgehalte en een te hoog glucosegehalte).

Generaliseerbaarheid van de casus wettelijk rookverbod in de horeca

Met het oog op de algemene geldigheid (***onafhankelijkheid van de politiek-ideologische context***) van het model, is het een voordeel dat de casus 'wettelijk rookverbod in de horeca' over een periode van ruim 25 jaar de argumenten van in principe alle in de Eerste en Tweede Kamer vertegenwoordigde politieke partijen betreft. In dit verband is het ook vermeldenswaardig dat bij de voorbereiding en implementatie van het wettelijk rookverbod in de horeca vier ministers van VWS van drie verschillende politieke partijen het voortouw hebben gehad[5].

Algemene geldigheid

De casus wettelijk rookverbod in de horeca:

5. Ministers van VWS:
   - E. Borst-Eilers (D'66, sociaal liberale partij) (22 augustus 1994 tot 22 juli 2002);
   - H. Hoogervorst (VVD, liberale partij) (27 mei 2003 tot 22 februari 2007);
   - A. Klink (CDA, christen democratische partij) (22 februari 2007 tot 14 oktober 2010);
   - E. Schippers (VVD, liberale partij) (14 oktober 2010 tot juni 2011/ einde van mijn onderzoek).

- is omvangrijk (betreft alle horeca in Nederland),
- kent veel invalshoeken (zelfregulering, wetgeving, internationale beleidsmatige inkadering, alternatieven in de vorm van rookruimten en ventilatiesystemen, negatieve neveneffecten, een gelaagd toezichtsysteem; een boetesysteem; rechterlijke uitspraken; onafhankelijke onderzoeken; enz.),
- is uitgebreid en degelijk gedocumenteerd (vergaderstukken van de Tweede en Eerste Kamer), en
- betreft een lange periode (ruim 25 jaar).

Daarom acht ik het, na toetsing van het model aan de casus wettelijk rookverbod in de horeca, onwaarschijnlijk dat toetsing van het model aan nieuwe casuïstiek nog tot essentiële veranderingen aan het model zal leiden.

Op basis van de toetsing van de juistheid, volledigheid en praktische toepasbaarheid van het model aan de hand van het preventieplan van Clarian Health en het wettelijk rookverbod in de horeca, acht ik het ***aannemelijk*** dat het model ***algemeen geldig*** is. Om met (nog) meer zekerheid een uitspraak te kunnen doen over de algemene geldigheid dienen (veel) meer verschillende preventiemaatregelen te zijn beoordeeld.

15.2.7 Reikwijdte

Met de reikwijdte doel ik op de vraag of het model ook op andere maatregelen dan preventiemaatregelen en ook buiten de sector volksgezondheid en gezondheidszorg van toepassing is.

Ik ***vermoed*** dat de reikwijdte van het model zich ***uitstrekt*** tot alle situaties waarbij wordt getracht de leefstijl of het gedrag van mensen met ***drang of dwang*** te beïnvloeden. Hierbij kan bijvoorbeeld worden gedacht aan:
- preventie van anorexia, van automutilatie, van zelfdoding;
- preventie van milieuschade (bijvoorbeeld ten gevolge van uitlaatgassen van auto's);
- preventie van materiele schade (bijvoorbeeld ten gevolge van vandalisme);
- preventie van financiële schade (bijvoorbeeld ten gevolge van diefstal);
- preventie van geluidsoverlast (bijvoorbeeld ten gevolge van hangjongeren);
- invoering en handhaving van leerplicht;
- invoering van een burkaverbod;
- enz.

Of de leerplicht al dan niet is ingevoerd ter voorkoming van schade aan (de ontwikkeling van) het individu of de maatschappij is discutabel. Het criterium 'schadelijkheid' van het model biedt echter ook de mogelijkheid om morele opvattingen aan te voeren als rechtvaardigingsgrond voor het beïnvloeden van de leefstijl of het gedrag van mensen. Het model stelt enkel dat de preventie van schade in het algemeen een sterkere rechtvaardigingsgrond vormt voor leefstijlinterventies dan het nastreven van bepaalde morele opvattingen (zie bijlage 5). Als mijn vermoeden klopt, is de reikwijdte van het model dus niet beperkt tot de sector volksgezondheid en gezondheidszorg, maar kan het model worden toegepast in alle sectoren van de maatschappij.

Ik ***denk*** dat de reikwijdte c.q. relevantie van het model zich ***beperkt*** tot situaties waarbij wordt getracht de leefstijl of het gedrag van mensen met ***drang of dwang*** te beïnvloeden. Als mensen uit vrije wil instemmen met een bepaalde interventie (er is sprake van informed consent) maken zij individueel de afweging of de effectiviteit van de preventiemaatregel voor hen opweegt tegen de indringendheid. Het model richt zich juist op de algemene aanvaardbaarheid van leefstijlbeïnvloedende interventies, waarbij sprake is van drang of dwang tot leefstijlverandering.

15.2.8 Invloed van implementatie op (neven)effecten

Het beoordelingsmodel is opgebouwd uit een drietal zeven:
- logica van het ontwerp (1e zeef);
- effecten en neveneffecten (2e zeef);
- implementatie (3e zeef).

Een complex gegeven bij de beoordeling van de gerechtvaardigdheid van leefstijlbeïnvloedende preventiemaatregelen is dat de kwaliteit van de implementatie (3e zeef) van invloed is op de effecten en neveneffecten (2e zeef) van de preventiemaatregel. Ter illustratie hiervan volgen twee voorbeelden:
- voorlichting (3e zeef, criterium flankerend beleid) over de preventiemaatregel kan de naleving en daarmee effectiviteit (2e zeef) verbeteren;
- geld dat men uitgeeft aan de handhaving (3e zeef, criteria flankerend beleid en uitvoeringscapaciteit) is van invloed op de kosteneffectiviteit (2e zeef).

Omdat de implementatie van de preventiemaatregel (3e zeef) van invloed is op de effecten en neveneffecten van de preventiemaatregel (2e zeef), dient men bij de beoordeling van de effecten en neveneffecten (2e zeef) de (verwachte) invloed van de implementatie (3e zeef) te verdisconteren.

Als men bij de beoordeling van de preventiemaatregel aan de hand van de 2e zeef de (verwachte) invloed van de implementatie (3e zeef) verdisconteert, wat is dan nog de toegevoegde waarde van beoordeling aan de hand van de 3e zeef? De toegevoegde waarde is dat ***bij de 3e zeef de implementatie zelf wordt beoordeeld:***

- wat is de (beoogde) kwaliteit van de implementatie?
- in hoeverre is de (beoogde) kwaliteit van de implementatie haalbaar en houdbaar?

Het is immers mogelijk dat de effecten en neveneffecten (2e zeef) van een preventiemaatregel te rechtvaardigen zijn, terwijl de implementatie (3e zeef) verwijtbaar suboptimaal verloopt. Ten behoeve van de 3e zeef van het model heb ik daarom het optimalisatiebeginsel (geen onachtzaamheid en geen nalatigheid) als uitgangspunt voor de beoordeling toegevoegd (zie §13.3.1).

15.2.9 Optimalisatiebeginsel

Beoordeling aan de hand van het optimalisatiebeginsel

De toepassing van het optimalisatiebeginsel voor de beoordeling van de gerechtvaardigdheid van de 'implementatie van de preventiemaatregel' heeft als consequentie dat een negatief oordeel over de implementatie (3e zeef) anders dient te worden gewogen dan een negatief oordeel over de effecten en neveneffecten (2e zeef) of over het ontwerp (1e zeef). Als op basis van beoordeling aan de hand van de 1e of 2e zeef blijkt dat implementatie ongerechtvaardigd is, dient de maatregel niet (op die wijze) te worden geïmplementeerd. Als op basis van beoordeling aan de hand van de 3e zeef blijkt dat de implementatie ongerechtvaardigd is, is dat een ***aansporing om de implementatie te verbeteren***. Dit betekent echter niet automatisch dat de implementatie moet worden stopgezet. Als de implementatie suboptimaal wordt uitgevoerd, maar het ontwerp (1e zeef) en de effecten en neveneffecten (2e zeef) zijn ondanks de suboptimale implementatie gerechtvaardigd, dan kan de suboptimale implementatie doorgang vinden.

Reikwijdte van het optimalisatiebeginsel

Tijdens het schrijven van dit hoofdstuk 'discussie' dringt de vraag zich bij mij op waarom ik de toepassing van het optimalisatiebeginsel heb beperkt tot de 3e zeef van het model en niet heb uitgebreid naar de 1e en 2e zeef van het model. In §2.6.2 heb ik bij de bespreking van rij X van overzicht 2 geschreven, dat bij ieder criterium van het model moet worden overwogen of er geen betere alternatieven mogelijk zijn voor het ontwerp en de wijze van toepassing van de preventiemaatregel. Met het oog hierop heb ik in versie 5 van mijn model zowel in de 1e, 2e als 3e zeef van het model een criterium 'alternatieven' opgenomen (zie §2.6.2). Als deze afweging bij ieder criterium moet worden gemaakt, vormt deze afweging in het kader

van het model een algemeen geldig beoordelingsprincipe. Op basis hiervan trek ik nu de conclusie dat het ***optimalisatiebeginsel ook van toepassing is op de 1e en 2e zeef*** van het model.

Als op basis van beoordeling aan de hand van het optimalisatiebeginsel blijkt dat het ontwerp (1e zeef) of de effecten en neveneffecten (2e zeef) van de preventiemaatregel suboptimaal zijn, betekent dat niet automatisch dat toepassing van de beoordeelde preventiemaatregel ongerechtvaardigd is. Het betekent wel dat het ontwerp (1e zeef) en de effecten en neveneffecten (2e zeef) suboptimaal zijn en dat is een aansporing om deze te verbeteren.

15.3 Relevantie van mijn onderzoek en beoordelingsmodel

15.3.1 Nog niet bestaand model

Voor zover ik weet is er nog geen model waarmee de gerechtvaardigdheid van leefstijlbeïnvloedende preventiemaatregelen ter bestrijding van leefstijlgerelateerde aandoeningen op een algemeen aanvaardbare wijze beoordeeld kan worden én waarvoor geldt:

- dat de criteria zodanig geoperationaliseerd zijn dat ze praktisch toepasbaar zijn;
- dat de juistheid, volledigheid en praktische toepasbaarheid van het model aannemelijk zijn gemaakt, door toepassing van het model op casuïstiek uit de praktijk.[6]

Een dergelijk model hoop ik nu te bieden. Wellicht is het model ook buiten de sector volksgezondheid en gezondheidszorg bruikbaar (zie §15.1.7 over de reikwijdte).

15.3.2 Gebruikers en gebruiksmogelijkheden

Mogelijke gebruikers van het model zijn:

- landelijke en gemeentelijke overheid (VWS, RVZ, GGD-en) en politiek;
- burgers;
- bedrijven (denk hierbij bijvoorbeeld aan werkgevers en zorgverzekeraars);
- zorginstellingen en zorgverleners;

6. De conclusie van het artikel '*An overview of ethical frameworks in public health: can they be supportive in the evaluation of programs to prevent overweight*' luidt: *"Public health ethical frameworks may be supportive in the evaluation of overweight prevention programs or policy, but seem to lack practical guidance to address ethical conflicts in this particular area."* (Have ten M, 2010, p.1).

- onderzoeksinstellingen (RIVM, NIGZ);
- rechterlijke macht en advocatuur (denk hierbij bijvoorbeeld aan de rechtszaken tegen het wettelijk rookverbod voor kleine cafés zonder personeel).

Zij kunnen het model gebruiken voor het:
- beoordelen van de gerechtvaardigdheid,
- onderling vergelijken,
- ontwerpen,
- onderzoeken, en
- argumentatief onderbouwen of bekritiseren van leefstijlbeïnvloedende preventiemaatregelen, als er sprake is van drang of dwang. Deze gebruiksmogelijkheden zal ik hieronder kort toelichten.

Beoordelen van de gerechtvaardigdheid
Voor het beoordelen van de gerechtvaardigdheid van leefstijlbeïnvloedende preventiemaatregelen heb ik het model gemaakt. Zie de totstandkoming van de 4e en definitieve versie van de onderzoeksvraag in de inleiding (§1.1 en 1.2). De definitieve versie van het model – beoordelingscriteria, beoordelingsstructuur en beoordelingsprincipes – vormt het antwoord op de definitieve onderzoeksvraag (zie §15.1).

Onderling vergelijken
Het aantal mogelijke leefstijlbeïnvloedende preventiemaatregelen ter bestrijding van een bepaalde leefstijlgerelateerde aandoening is zeer groot (zie §2.2). Als eenmaal voor een bepaalde preventiemaatregel is gekozen, is de variatie in de manier waarop de preventiemaatregel kan worden uitgevoerd nog steeds zeer groot. Bovendien zijn voor de uitvoering meestal geen (medische) richtlijnen beschikbaar die de variatie in de uitvoering inperken. Hierdoor is het complex om de lasten-batenverhouding van verschillende leefstijlbeïnvloedende preventiemaatregelen eerlijk met elkaar te vergelijken. Het aantal variabelen dat relevant is voor de vergelijking – de criteria van het model – is namelijk groot. Als men de lasten-batenverhouding van verschillende leefstijlbeïnvloedende preventiemaatregelen met elkaar wil vergelijken, verdient het met het oog op de rationaliteit en volledigheid van de vergelijking daarom aanbeveling om de criteria van het model als ***checklist*** te gebruiken.

Ontwerpen
Ook bij het ontwerpen van een preventiemaatregel kunnen de criteria van het model als ***checklist*** worden gebruikt. De 1e zeef (logica van het ontwerp) van het model betreft niet het hele ontwerp, maar de hoofdstructuur van de preventie-

maatregel. Voor het ontwerpen van een preventiemaatregel zijn ook de criteria van de 2e en 3e zeef van belang. Zo is het belangrijk om al bij het ontwerp van de preventiemaatregel na te denken over de te verwachten (neven)effecten (denk bijvoorbeeld aan de indringendheid) en over de (on)mogelijkheden bij implementatie in de praktijk (denk bijvoorbeeld aan de handhaving en uitvoeringscapaciteit).

Onderzoeken
Bij het monitoren en evalueren van een preventiemaatregel kunnen de criteria van het model eveneens als ***checklist*** worden gebruikt. De ***operationalisering*** van de criteria (zie §15.1.1) geeft aan onderzoekers handvatten op welke aspecten van de preventiemaatregel zij bij een vooronderzoek, de monitoring en/of evaluatie zouden kunnen en/of moeten focussen.

Argumentatief onderbouwen of bekritiseren
Door de volgorde van de criteria en de impliciete clustering van criteria draagt de structuur van het model in grote mate bij aan de rationaliteit van de beoordeling. Beleidsmakers, ondernemers, burgers, juristen, enz. kunnen van deze structuur gebruik maken voor de ***structuur van de argumentatie*** bij het onderbouwen en/of bekritiseren van de gerechtvaardigdheid van een bepaalde preventiemaatregel. Een volledige beoordeling van de gerechtvaardigdheid, d.w.z. het doorlopen van alle 14 criteria (zie de hoofdstukken 5 t/m 9 en 10 t/m 14) is veel werk en niet altijd nodig of haalbaar. Daarom kan men zich desgewenst ook beperken tot het gebruik van de criteria van één zeef, of zelfs tot het gebruik van de ***operationalisering van één afzonderlijk criterium***. Dit laatste zou bijvoorbeeld kunnen gelden voor de operationalisering van het criterium ‘indringendheid’ (zie §7.2 en 15.1.1) dat bij de beoordeling van leefstijlbeïnvloedende preventiemaatregelen een essentiële rol speelt.

15.3.3 Aanbevelingen voor het beleid

Fasering van pakketbeslissingen
De Raad voor Volksgezondheid en Zorg (RVZ) pleit ervoor om beslissingen over het basispakket van de zorgverzekeringen in twee fasen te nemen. In de eerste fase, de assessment fase, wordt op basis van kwantitatieve onderzoeksgegevens een principebesluit genomen. In de tweede fase, de appraisal fase, wordt dit principebesluit maatschappelijk getoetst. Een belangrijke uitkomst van mijn onderzoek is dat een dergelijke fasering in strijd is met de rationaliteit van het beoordelingsproces in het kader van mijn model. Een dergelijke fasering is m.i. echter ook in strijd met de rationaliteit van het besluitvormingsproces bij pakketbeslissingen. Wel is het wenselijk om, zowel bij mijn model als bij pakketbeslissingen, per criterium een dergelijke fasering van de beoordeling aan te houden. (Zie §3.3.)

Het probleem van de fasering die de RVZ voorstelt, is dat de uitkomst van de beoordeling aan de hand van een bepaald criterium de input kan vormen voor de beoordeling aan de hand van een of meer van de volgende criteria. Binnen de besluitvormingsmodellen van de RVZ en het CVZ voor pakketbeslissingen (zie overzicht 2) geldt dit bijvoorbeeld voor het 1e criterium 'noodzakelijkheid' (welk behandeldoel acht men noodzakelijk) en het 2e criterium 'effectiviteit' (in hoeverre wordt dit behandeldoel gehaald). De logische consequentie is, dat als men op basis van maatschappelijke toetsing (appraisal) van het principebesluit de besluitvorming over een criterium aanpast, het principebesluit mogelijk voor alle navolgende criteria niet meer geldig is. In dat geval dienen voor de zekerheid alle navolgende criteria opnieuw kwantitatief (assessment) te worden beoordeeld.

Op basis van bovenstaande overweging kom ik tot de aanbeveling aan beleidsmakers (met name aan het CVZ als pakketautoriteit) om het besluitvormingsproces bij pakketbeslissingen ***per criterium van het besluitvormingsmodel*** onder te verdelen in ***een 'assessment fase' en 'appraisal fase'***.

Beleidsinstrument voor 'life politics'

In §15.1.7 heb ik het vermoeden uitgesproken dat de reikwijdte van mijn model zich uitstrekt tot alle situaties waarbij wordt getracht de leefstijl of het gedrag van mensen met drang of dwang te beïnvloeden. Als dit vermoeden klopt, zijn de door mij besproken gebruiksmogelijkheden van toepassing op alle overheidsbeleid waarbij de overheid tracht het gedrag van burgers of organisaties te beïnvloeden. Zo bezien zou het beoordelingsmodel een beleidsinstrument kunnen zijn voor het bedrijven van 'life politics' (Giddens, 1994). Zie voor een toelichting op 'life politics' het *'Voorbeeld van tegengestelde politiek-ideologische denkkaders'* in bijlage 6.

15.3.4 Aanbevelingen voor de wetenschap

Kwantificering van inbreuken op het persoonlijk leven

In de afgelopen decennia heeft de wetenschap op het terrein van de volksgezondheid een grote inspanning geleverd om de effecten van preventie en behandeling op de volksgezondheid te kwantificeren. Kwantitatieve uitkomstmaten die de wetenschap heeft ontwikkeld, zijn bijvoorbeeld de QALY, DALY en reductie van ziektelast. M.i. dient de wetenschap een vergelijkbare inspanning te gaan leveren om de effecten te kwantificeren die leefstijlbeïnvloedende preventiemaatregelen hebben op de lichamelijke integriteit, (keuze)vrijheid, privacy, stigmatisering en

discriminatie van mensen[7]. Voor het kwantificeren van immateriële zaken kan gebruik worden gemaakt van concepten als 'Willingness to Pay' en Willingness to Accept' (zie §6.1.1).

Onderzoek naar kansen en risico's van nieuwe wetgeving

Ten behoeve van de bestrijding van leefstijlgerelateerde aandoeningen kan het interessant zijn om nieuwe mogelijkheden tot wetgeving ter bestrijding van leefstijlgerelateerde aandoeningen te onderzoeken op kansen en risico's. Hierbij kan bijvoorbeeld worden gedacht aan:

- wetgeving die portiegroottes in supermarkten en de horeca reguleert;
- wettelijke maximering van de hoeveelheid zout en suiker die de industrie aan voedingsmiddelen toevoegt (de consument kan desgewenst vaak zelf zout en suiker toevoegen);
- een zogenaamde 'vet-tax'.
- een wettelijk verbod op frisdrank- en snoepautomaten in scholen;
- wettelijke bescherming van ***bezoekers*** in de horeca tegen passief roken.

Herhaalde toetsing van het model

Om meer zekerheid te verkrijgen over de volledigheid (§15.1.1), validiteit (§15.1.3) betrouwbaarheid (§15.1.4), praktische toepasbaarheid (§15.15), algemene geldigheid (§15.1.6) en reikwijdte (§15.1.7) van mijn model, pleit ik voor toetsing van het model aan meer praktijkcases door wetenschappers, beleidsmakers en veldpartijen.

7. Met behulp van deze begrippen heb ik het criterium 'indringendheid' van het model geoperationaliseerd.

Samenvatting

De samenvatting van mijn proefschrift is als volgt opgebouwd:
I. inleiding;
II. beschrijving van het onderzoek;
III. beoordeling van de casuïstiek;
IV. presentatie van het beoordelingsmodel;
V. beschouwing va het onderzoek en beoordelingsmodel.

I Inleiding

Leefstijlgerelateerde aandoeningen vormen wereldwijd in relatieve en absolute zin een toenemend gevaar voor de volksgezondheid. In relatieve zin omdat de meeste infectieziekten, die in het verleden de grootste bedreiging voor de volksgezondheid vormden, tegenwoordig met succes kunnen worden bestreden. In absolute zin, omdat mede ten gevolge van de toenemende welvaart, een groot deel van de bevolking steeds ongezonder leeft. Bij een ongezonde leefstijl kan in dit verband ondermeer worden gedacht aan roken, te veel en ongezonde voeding, te weinig bewegen en overmatig alcoholgebruik.

Aan de preventie van leefstijlgerelateerde aandoeningen kan op veel verschillende manieren vorm worden gegeven. Aan de preventie van overgewicht kan bijvoorbeeld vorm worden gegeven door:
- mensen te stimuleren of te verplichten meer te bewegen;
- mensen te stimuleren of te verplichten een dieet te volgen;
- fabrikanten van voedingsmiddelen te stimuleren of te verplichten de hoeveelheid suiker en (ongezonde) vetten in voedingsmiddelen te verlagen;
- regelgeving m.b.t. portiegroottes in supermarkten en de horeca te introduceren;
- beperkingen te stellen aan de reclame voor levens- en genotsmiddelen;
- enz.

Van diverse van de bovenstaande maatregelen staat de effectiviteit ter discussie. Aan de andere kant wordt steeds duidelijker dat alleen het geven van voorlichting er meestal niet toe leidt dat mensen gezonder gaan leven. Steeds meer wetenschappers pleiten daarom voor het toepassen van vormen van ***drang of dwang*** om bur-

gers aan te zetten tot gezonder gedrag en om de voedingsmiddelenindustrie aan te zetten tot een gezonder productenaanbod en tot het minder verleiden van mensen tot overconsumptie. Preventiemaatregelen om de productie, de distributie en de consumptie van levens- en genotsmiddelen te beïnvloeden of zelfs te verbieden, vormen echter een aantasting van de keuzevrijheid van de bevolking en het vrije ondernemerschap. In hoeverre is het toepassen van dergelijke maatregelen gerechtvaardigd en hoe kan dat worden beoordeeld? Deze vraag vormde voor mij de aanleiding om een model te ontwikkelen aan de hand waarvan de gerechtvaardigdheid van leefstijlbeïnvloedende interventies ter bestrijding van leefstijlgerelateerde aandoeningen kan worden beoordeeld.

II Beschrijving van het onderzoek

II.I Probleemstelling en onderzoeksvraag

De risico's van preventiemaatregelen ter bestrijding van leefstijlgerelateerde aandoeningen voor de keuzevrijheid van de bevolking, voor de financiële belangen van het bedrijfsleven, en voor het vrije verkeer van goederen en diensten, roepen de belangrijke vraag op in hoeverre dergelijke preventiemaatregelen gerechtvaardigd zijn. Op basis van deze probleemstelling heb ik aan het begin van mijn onderzoek in 2005 een onderzoeksvraag geformuleerd. Op basis van voortschrijdend inzicht heb ik deze onderzoeksvraag tijdens de uitvoering van het onderzoek een aantal keren aangescherpt (§1.2). De 4e en tevens laatste versie van de onderzoeksvraag luidt als volgt:

Laatste versie van de onderzoeksvraag (2008)

Kan een model worden ontwikkeld aan de hand waarvan de gerechtvaardigdheid van preventiemaatregelen kan worden beoordeeld, die ter bestrijding van leefstijlgerelateerde aandoeningen trachten de leefstijl van mensen te beïnvloeden?

II.II Focus op gerechtvaardigdheid van de argumentatie

De gerechtvaardigdheid van de toepassing van een preventiemaatregel berust m.i. op twee pijlers:

1. De gerechtvaardigdheid van de ***argumentatie*** die heeft geleid tot het besluit om de preventiemaatregel toe te passen;

2. De gerechtvaardigdheid van de ***procedure*** (welke partijen kunnen op welke moment welke invloed uitoefenen op de besluitvorming?) die heeft geleid tot het besluit om de preventiemaatregel toe te passen.

Het beoordelingsmodel richt zich op de gerechtvaardigdheid van de argumentatie. Afhankelijk van de casus dient per criterium te worden afgewogen in hoeverre procedurele aspecten relevant zijn voor de inhoudelijke beoordeling van de gerechtvaardigdheid van de preventiemaatregel. Op zichzelf valt de beoordeling van de procedurele gerechtvaardigdheid buiten het kader van mijn model (§12.4.4).

II.III Onderzoeksaanpak en onderzoeksmethoden

Onderzoeksaanpak

Om de onderzoeksvraag te beantwoorden heb ik onderzoek gedaan. Dit onderzoek valt in twee delen uiteen:
- ontwikkeling van het beoordelingsmodel op basis van ***literatuuronderzoek*** (hoofdstuk 1 t/m 4);
- empirische toetsing van het beoordelingsmodel op basis van ***casuïstiek uit de praktijk*** (hoofdstuk 5 t/m 14).

Inhoudelijk valt het literatuuronderzoek in drie delen uiteen:
- onderzoek naar bestaande beoordelingsmodellen die kunnen worden gebruikt bij de ontwikkeling van het beoogde model (hoofdstuk 2 en 3);
- onderzoek naar beoordelingsprincipes voor het beoogde model (hoofdstuk 4);
- onderzoek naar praktijkvoorbeelden van de preventie van leefstijlgerelateerde aandoeningen (bijlage 6).

De juistheid, volledigheid en praktische toepasbaarheid van het door mij ontwikkelde model heb ik getoetst aan de hand van twee casussen uit de praktijk:
- het preventieplan van de Amerikaanse ziekenhuisketen Clarian Health (hoofdstuk 5 t/m 9);
- het wettelijk rookverbod in de Nederlandse horeca (hoofdstuk 10 t/m 14).

Waarom deze praktijkcases zijn gekozen, wordt toegelicht aan het begin van hoofdstuk 5 (Clarian) en aan het begin van hoofdstuk 10 (rookverbod in de horeca).

Tijdens de empirische toetsing van het model aan de casuïstiek uit de praktijk heb ik de criteria van het model geoperationaliseerd en de wijze van beoordeling per

criterium vastgesteld. Voor het operationaliseren van de criteria en het vaststellen van de bijbehorende wijze van beoordeling heb ik zowel gebruik gemaakt van de door mij bestudeerde literatuur als van de genoemde casuïstiek.

Beschrijving van de casus Clarian Health
In 2007 kondigde de Amerikaanse ziekenhuisketen Clarian Health aan voornemens te zijn om in 2009 te starten met een geheel nieuwe benadering om de jaarlijkse ziektekosten van haar werknemers te reduceren. Het plan van Clarian bestond eruit om werknemers financieel te straffen voor hun ongezonde leefstijl. Daartoe had Clarian de volgende indicatoren van ongezondheid vastgesteld – roken, ernstig overgewicht, te hoge bloeddruk, te hoog cholesterolgehalte en een te hoog glucosegehalte – op basis waarvan werknemers maandelijks zouden worden beboet. Het preventieplan van Clarian heeft tot veel commotie in de media geleid. Op internet is op nieuwspagina's en weblogs veel aandacht aan dit preventieplan besteed. Voor mij boden de vele schriftelijke reacties op dit preventieplan een uitgelezen kans om de juistheid, volledigheid en praktische toepasbaarheid van het model empirisch te toetsen. Hiertoe heb ik de gerechtvaardigdheid van het preventieplan van Clarian beoordeeld aan de hand van (de criteria van) het model.

Beschrijving van de casus wettelijk rookverbod in de horeca
De juistheid, volledigheid en praktische toepasbaarheid van het model heb ik nogmaals getoetst door de gerechtvaardigdheid van het wettelijk rookverbod in de horeca te beoordelen aan de hand van het model ('the proof of the pudding is in the eating'). Het wettelijk rookverbod in de Nederlandse horeca is ingesteld door de Nederlandse overheid en van kracht geworden op 1 juli 2008. Daarom heb ik de beoordeling van de gerechtvaardigdheid van dit rookverbod gebaseerd op de formele documenten waarmee de Nederlandse overheid de totstandkoming en toepassing van dit rookverbod tot dusver heeft onderbouwd. Mijn literatuuronderzoek naar de argumenten waarmee de overheid de invoering en handhaving van dit rookverbod heeft onderbouwd, betreft een periode van ruim 25 jaar (november 1984 t/m juni 2011). De kern van dit literatuuronderzoek wordt gevormd door vergaderstukken van de Tweede en Eerste Kamer, nieuwsberichten op de website van het ministerie van VWS en relevante wet- en regelgeving (§10.2).

Onderzoeksmethoden
Voor mijn onderzoek heb ik gebruik gemaakt van drie onderzoeksmethoden (§1.5):
- literatuuronderzoek;
- argumentatieve tekstanalyse;
- redeneren.

III Beoordeling van de casuïstiek

III.1 Gerechtvaardigdheid van het preventieplan van Clarian Health

Op basis van de informatie die via internet over het preventieplan van Clarian beschikbaar is, zijn het ontwerp (1e zeef van het model), de effecten en neveneffecten (2e zeef) en de wijze van implementatie (3^{e} zeef) van dit preventieplan aan de hand van het model ***niet te rechtvaardigen***. In de eerste plaats omdat er over het preventieplan, m.b.t. veel criteria van het model, onvoldoende informatie beschikbaar is om tot een betrouwbare beoordeling te kunnen komen. In de tweede plaats omdat op basis van de informatie die wel beschikbaar is, geconcludeerd kan worden dat toepassing van het preventieplan van Clarian in de huidige vorm niet gerechtvaardigd is. De belangrijkste inhoudelijke punten van kritiek zijn de volgende:

- De keuze van de Amerikaanse federale overheid om werkgevers in de gelegenheid te stellen om, weliswaar binnen een gegeven (financieel) kader[1], naar eigen inzicht de eigen werknemers financieel te straffen voor een ongezonde leefstijl binnen en buiten werktijd, acht ik zeer discutabel. Werkgevers zijn in mijn ogen ongeschikt om dergelijke preventiemaatregelen te ontwerpen en uit te voeren. Ter voorkoming van rechtsongelijkheid tussen werknemers van verschillende werkgevers en willekeur van werkgevers zou het m.i. beter zijn, als de democratisch gekozen rijksoverheid zou beslissen over de vormgeving van dergelijke preventiemaatregelen. De uitvoering van dergelijke preventiemaatregelen kan m.i. beter worden overgelaten aan een 'trusted third party' (bijvoorbeeld de zorgverzekeraar). (§6.7)
- De indringendheid van de preventiemaatregel (structurele aantasting van de lichamelijke integriteit en privacy) acht ik niet te rechtvaardigen voor de werknemers die wel aan de gezondheidscriteria van Clarian voldoen. (§7.7)
- Dat werknemers van Clarian (en hun gezin) door het preventieplan ernstig in de financiële problemen kunnen raken of hun zorgpremie niet meer kunnen betalen, acht ik niet te rechtvaardigen. Wellicht kan een hardheidsclausule op dit punt uitkomst bieden. (§7.7)

1. Zie bijlage 8: Toelichting op §f van de HIPAA.

III.II Gerechtvaardigdheid van het wettelijk rookverbod in de horeca

Gebrek aan transparantie over de doelstelling

In de politiek is in het openbaar uitgebreid gedebatteerd over de invoering van het wettelijk rookverbod in de horeca. Daarom vind ik het opmerkelijk dat uit de bestudering van parlementaire documenten blijkt dat dit debat niet heeft geleid tot transparantie over de doelstellingen van het wettelijk rookverbod in de horeca. Achtereenvolgende ministers van VWS hebben over de doelstelling van het wettelijk rookverbod in de horeca wisselende standpunten ingenomen (§10.5). De Tweede Kamer had m.i. in juni 2007 geen genoegen mogen nemen met de uitspraak van minister Klink van VWS, dat de bescherming van bezoekers een secundaire doelstelling is van het wettelijk rookverbod in de horeca. Het begrip 'secundaire doelstelling' blijkt multi-interpretabel. In juli 2007 vindt minister Klink een secundaire doelstelling 'een doelstelling'. In dat geval valt de bescherming van bezoekers tegen passief roken onder het criterium 'schadelijkheid' van de 1e zeef van het model. In juni 2009 vindt minister Klink een secundaire doelstelling niet meer dan 'een positief neveneffect'. In dat geval valt de bescherming van bezoekers tegen passief roken onder het criterium 'lasten-batenverhouding' uit de 2e zeef van het model. Deze wisseling van standpunt over de doelstelling van het wettelijk rookverbod in de horeca is m.i. door minister Klink onvoldoende beargumenteerd.

Vanuit het perspectief van transparantie over de doelstellingen van het wettelijk rookverbod in de horeca, kan het wettelijk rookverbod in de horeca tijdens de bewindsperiode van minister Klink de ***1e zeef*** van het beoordelingsmodel (logica van het ontwerp) ***niet passeren***. Invoering van een wettelijk rookverbod in de horeca, zonder dat de doelstellingen helder zijn, acht ik ***onvoldoende transparant***.

Met het oog op de vrije toegankelijkheid van openbare voorzieningen voor iedereen, vind ik dat ook bezoekers in de horeca wettelijk tegen passief roken beschermd dienen te worden. Daarom ben ik het oneens met de stellingname van minister Schippers van VWS, dat de doelstelling van het wettelijk rookverbod in de horeca zich dient te beperken tot de bescherming van werknemers in de horeca tegen passief roken. Deze stellingname van minister Schippers is niet in overeenstemming met de geest van (zie onder het criterium 'legitimiteit' in §12.4.2):

- de Tabakswet;
- het WHO-Kaderverdrag inzake tabaksontmoediging (2003);
- de afspraken van minister Hoogervorst van VWS met KHN over het stappenplan ter zelfregulering van het rookbeleid in de horeca (2004);
- de Aanbeveling van de Raad van Europa betreffende rookvrije ruimten (2009).

Mijn persoonlijke opvatting over de noodzakelijkheid van wettelijke bescherming van bezoekers in de horeca tegen passief roken, is in het kader van het model echter niet relevant. Dat de stellingname van minister Schippers niet in overeenstemming is met de geest van de vier bovengenoemde documenten, acht ik onvoldoende reden om de stellingname van minister Schippers ongerechtvaardigd te verklaren. Formeel juridisch vormen de bovengenoemde documenten m.i. geen (onoverkomelijke) belemmering voor de stellingname van minister Schippers.

Vanuit het perspectief van transparantie over de doelstellingen van het wettelijk rookverbod in de horeca, kan het wettelijk rookverbod in de horeca tijdens de bewindsperiode van minister Schippers de ***1e zeef*** van het beoordelingsmodel (logica van het ontwerp) ***wel passeren***. De visie van minister Schippers is ***helder*** (transparant) en niet multi-interpretabel. Volgens minister Schippers dient de doelstelling van het wettelijk rookverbod in de horeca zich te beperken, en beperkt deze zich, tot de bescherming van werknemers tegen passief roken.

Formele wetstekst als uitgangspunt voor beoordeling van wettelijk rookverbod

Bescherming van werknemers als uitgangspunt voor de beoordeling

Vanwege de onduidelijkheid over de doelstelling van het wettelijk rookverbod in de horeca tijdens de bewindsperiode van minister Klink, heb ik de gerechtvaardigdheid van het wettelijk rookverbod in de horeca beoordeeld aan de hand van het model, met de formele wetstekst als uitgangspunt. De formele wetstekst biedt alleen werknemers bescherming tegen passief roken (§10.5). Deze beperkte doelstelling van het wettelijk rookverbod in de horeca is, vanuit het perspectief van het model, op zichzelf niet ongerechtvaardigd (§10.6.4).

Logica van het ontwerp (1e zeef van het model)

Met de bescherming van werknemers in de horeca tegen passief roken als doelstelling van het wettelijk rookverbod, kan het wettelijk rookverbod in de horeca de 1e zeef ***wel passeren***: het ontwerp is logisch. Het ontwerp is niet moralistisch, niet perfectionistisch en niet paternalistisch.

Effecten en neveneffecten (2e zeef van het model)

Het wettelijk rookverbod in de horeca, zoals minister Klink dat ***per 1 juli 2007*** heeft ingevoerd, kan de 2e zeef ***niet passeren***: een van de (mogelijke) neveneffecten is onbillijk. Belangrijk is om op te merken dat de geconstateerde onbillijkheid maar een ***klein deel*** van de horeca betreft, namelijk de kleine cafés zonder personeel. Het aspect ‘hardheid in specifieke gevallen’ van het criterium ‘rechtvaardigheid’ vormt het breekpunt van de gerechtvaardigdheid van het rookverbod in kleine cafés zon-

der personeel. In verhouding tot de doelstelling van het rookverbod voor kleine cafés zonder personeel – het voorkomen van concurrentievervalsing met cafés met personeel – vallen de financiële gevolgen voor deze cafés mogelijk onevenredig hard uit. Ik schrijf ‘mogelijk’, omdat een degelijke financiële onderbouwing ontbreekt. Hierbij weeg ik mee dat de concurrentievervalsing in de praktijk wel meevalt. Zodra kleine cafés zonder personeel meer bezoekers gaan trekken, omdat er mag worden gerookt, moeten zij immers personeel in dienst nemen en is het rookverbod direct weer van toepassing.

Het ***huidige*** wettelijk rookverbod in de horeca, waarvan kleine cafés zonder personeel zijn uitgezonderd, kan de 2e zeef (effecten en neveneffecten) ***wel passeren***.

Wijze van Implementatie (3e zeef van het model)
Het wettelijk rookverbod in de horeca kan de 3e zeef ***niet passeren***. De manier waarop is geïmplementeerd, is op onderdelen onredelijk en onbillijk.

Minister Klink heeft bij de implementatie van het wettelijk rookverbod in de horeca m.i. te veel steken laten vallen om de manier waarop is geïmplementeerd gerechtvaardigd te kunnen noemen. Enerzijds heeft hij te weinig oog gehad voor behoud en versterking van het draagvlak voor het wettelijk rookverbod in de horeca bij KHN en bij horecaondernemers. Zie voor een toelichting onder het kopje ‘eindoordeel’ bij de criteria ‘timing’ (§13.1.3) en ‘draagvlak’ (§13.2.3). Anderzijds heeft hij te weinig oog gehad voor de vermeende negatieve (financiële) gevolgen van de implementatie voor kleine cafés zonder personeel. Zie voor een toelichting onder het kopje ‘eindoordeel’ bij de criteria ‘flankerend beleid’ (§13.3.3) en ‘controleerbaarheid’ (§13.4.3).

Minister Schippers is m.i. bij de versoepeling van het wettelijk rookverbod in de horeca – de vrijstelling van kleine cafés zonder personeel van dit rookverbod – ***onachtzaam en nalatig*** geweest m.b.t. de gevolgen voor de handhaving van het rookverbod. Zie voor een toelichting onder het kopje ‘eindoordeel’ bij het criterium ‘handhaafbaarheid’ (§13.4.3). Belangrijk is om op te merken dat de geconstateerde onachtzaamheid en nalatigheid maar een deel van de horeca betreft, namelijk de cafés. Deze onachtzaamheid en nalatigheid vormen echter onvoldoende reden om de versoepeling van het wettelijk rookverbod die minister Schippers heeft doorgevoerd, ongerechtvaardigd te noemen. Wel is er sprake van een ***suboptimale implementatie van de versoepeling*** van het wettelijk rookverbod door minister Schippers.

IV Presentatie van het beoordelingsmodel

De definitieve onderzoeksvraag die ik heb getracht te beantwoorden luidde (§1.4):

> Kan een model worden ontwikkeld aan de hand waarvan de gerechtvaardigdheid van preventiemaatregelen kan worden beoordeeld, die ter bestrijding van leefstijlgerelateerde aandoeningen trachten de leefstijl van mensen te beïnvloeden?

Deze onderzoeksvraag heb ik beantwoord door het presenteren van de definitieve versie van het model in §15.1.

Het model is samengesteld uit drie onderdelen:

- ***beoordelingscriteria:***
  - operationalisering van de criteria (§15.1.1).
- ***beoordelingsstructuur:***
  - beoordelingszeven (expliciete beoordelingsstructuur) (§15.1.2);
  - beoordelingsclusters (impliciete beoordelingsstructuur) (§15.1.2).
- ***wijze van beoordeling:***
  - wijze van beoordeling op hoofdlijnen (§15.1.3);
  - wijze van beoordeling per criterium (§15.1.1).

IV.1 Beoordelingscriteria

De ontwikkeling van versie 1 t/m 6 van het model is vooral gebaseerd op literatuur over bestaande beoordelingsmodellen (hoofdstuk 2 t/m 4) en de ontwikkeling van versie 7 t/m 9 van op de toetsing van het model aan casuïstiek uit de praktijk (hoofdstuk 7 t/m 14). De definitieve beoordelingscriteria worden hieronder weergegeven in overzicht 28.

Overzicht 28: definitieve beoordelingscriteria van het model

preventiemaatregel

↓

Logica van ontwerp (1e zeef)

1. Schadelijkheid
2. Noodzakelijkheid
3. Oorzakelijkheid
4. Verantwoordelijkheid
5. Gerichtheid
6. Geschiktheid

↓

Effecten en neveneffecten (2e zeef)

7. Effectiviteit
8. Indringendheid
9. Lasten-batenverhouding
10. Rechtvaardigheid

↓

Implementatie (3e zeef)

11. Draagvlak
12. Flankerend beleid
13. Controleerbaarheid
14. Uitvoeringscapaciteit

↓

Gerechtvaardigde preventiemaatregel

Hieronder volgt de operationalisering van de criteria in steekwoorden (zie voor de uitgebreide beschrijving §14.2.1).

1. Criterium schadelijkheid

- Welke schade, hinder of overlast, of van schending van morele opvattingen tracht men met de preventiemaatregel te bestrijden?

2. Criterium noodzakelijkheid

- In hoeverre is de preventiemaatregel noodzakelijk?
- In hoeverre is de preventiemaatregel moralistisch of perfectionistisch?

- Worden er sancties opgelegd aan personen of organisaties die de risico's veroorzaken?

3. Criterium oorzakelijkheid
- In hoeverre zijn de leefstijlen waarop de preventiemaatregel zich richt bepalend voor de risico's die men tracht te bestrijden?
- Welke factoren zijn bepalend voor de leefstijlen waarop de preventiemaatregel zich richt en in hoeverre zijn deze bepalend?

4. Criterium verantwoordelijkheid
- Welke partijen worden verantwoordelijk gehouden voor het ontstaan of de instandhouding van de risico's die men tracht te bestrijden?
- Is bij het vaststellen van de verantwoordelijkheid rekening gehouden met alle bekende determinanten (3e criterium) van deze risico's?
- In hoeverre is de preventiemaatregel paternalistisch?

5. Criterium gerichtheid
- Hangt de keuze van de doelgroep en leefstijlbeïnvloedende factoren logisch samen met de determinanten van de risico's (3e criterium) en verantwoordelijkheid (4e criterium) voor deze risico's?
- Worden personen binnen de doelgroep door de preventiemaatregel gemist of buiten de doelgroep door de preventiemaatregel geraakt?

6. Criterium geschiktheid
- Hangt de keuze van de partijen die de preventiemaatregel uitvoeren logisch samen met de gerichtheid (5e criterium) van de preventiemaatregel?
- Zijn de partijen die de preventiemaatregel uitvoeren daartoe bekwaam, bevoegd en geschikt?
- Brengt uitvoering van de preventiemaatregel geen schade toe aan de aard of het imago van de partijen die de maatregel uitvoeren?

7. Criterium effectiviteit
- In hoeverre worden de beoogde effecten van de preventiemaatregel gerealiseerd?

8. Criterium indringendheid

I. Algemene beoordeling:
- Bij wie wordt een inbreuk gemaakt op het persoonlijk leven?
- Door wie wordt een inbreuk gemaakt op het persoonlijk leven?
- In welke mate wordt een inbreuk gemaakt op het persoonlijk leven?

- Welke reikwijdte heeft de inbreuk op het persoonlijk leven?
- Leidt het toestaan van de inbreuk op het persoonlijk leven tot precedentwerking?
- Hoe bezwaarlijk is de inbreuk op het persoonlijk leven?

II. Beoordeling van verschillende aspecten:
- aantasting van de lichamelijke integriteit;
- aantasting van de vrijheid;
- aantasting van de privacy;
- aantasting van de veiligheidsbeleving;
- discriminatie;
- stigmatisering.

III. Beoordeling van de mate van drang of dwang die wordt toegepast.

9. Criterium lasten-batenverhouding
- Welke lasten en batenverhouding kent de preventiemaatregel en wat is de lasten-batenverhouding?

10. Criterium rechtvaardigheid
- Worden de lasten over partijen verdeeld naar rato van hun verantwoordelijkheid voor het risico op schade?
- Worden partijen in gelijke gevallen gelijk behandeld?
- Wordt rekening gehouden met de draagkracht van mensen?
- Wordt rekening gehouden met de persoonlijke omstandigheden van mensen of specifieke omstandigheden van organisaties?
- Worden de gelijke kansen van betrokken partijen wat betreft hun toegang tot gezondheidszorg en preventie beïnvloed?
- Wordt 'de verdeling van voor gezondheidszorg en preventie beschikbare middelen naar behoefte' beïnvloed?
- Is de preventiemaatregel niet strijdig met het vigerende recht of rechtsgeldige afspraken?

11. Criterium draagvlak
- Is de tijd rijp voor het toepassen van drang of dwang?
- Zijn mensen en organisaties geleidelijk op de preventiemaatregel voorbereid?
- In hoeverre is aandacht besteed aan het verwerven van draagvlak voor de preventiemaatregel?
- Zijn de actuele politieke, economische en maatschappelijke omstandigheden geschikt voor implementatie?
- Sluit de preventiemaatregel aan op het beleid van stakeholders?
- Hoeveel draagvlak is er voor de preventiemaatregel?

12. Criterium flankerend beleid

- Is de preventiemaatregel ingebed in breder (preventie)beleid?
- Is er geen beleid dat strijdig is met de preventiemaatregel?
- Hoe is de voorlichting over de preventiemaatregel geregeld?
- Hoe is de handhaving van de naleving geregeld?
- Worden tijdens de implementatie knelpunten geïnventariseerd en opgelost?

13. Criterium controleerbaarheid

- Worden het 'ontwerp', de 'effecten en neveneffecten' en 'implementatie' van de preventiemaatregel gemonitord en geëvalueerd

14. Criterium uitvoeringscapaciteit

- Worden voldoende mensen en middelen beschikbaar gesteld om de implementatie haalbaar en houdbaar te maken?

IV. Structuur van het beoordelingsmodel

Het idee voor de zeefstructuur heb ik ontleend aan de 'Trechter van Dunning'. Dit is het beoordelingsmodel dat in 1991 is ontwikkeld door de Commissie Keuzen in de Zorg.

De eerste reden om gebruik te maken van het principe van een trechter met achtereenvolgende zeven, is dat de ***volgorde*** waarin de beoordelingscriteria binnen het model worden gehanteerd van grote invloed is op de inhoud en daarmee op de ***validiteit*** van het beoordelingsproces. Door de clustering van de beoordelingscriteria in zeven wordt inzichtelijker waarom de volgorde van de criteria essentieel is.

De tweede reden om gebruik te maken van het principe van een trechter met achtereenvolgende zeven, is dat hierdoor het aantal van veertien criteria beter hanteerbaar wordt. Als een bepaalde zeef niet wordt gepasseerd, hoeven de achterliggende criteria immers niet meer te worden onderzocht.

De derde reden om gebruik te maken van het principe van een trechter met achtereenvolgende zeven is de volgende. ***Criteria binnen een zeef mogen tegen elkaar worden gewogen.*** Dit betekent dat een goede score op een bepaald criterium een slechte score op een ander criterium kan compenseren. ***Criteria afkomstig uit verschillende zeven mogen niet tegen elkaar worden gewogen.*** Ter illustratie hiervan twee voorbeelden:

- als preventie niet noodzakelijk (1e zeef) is, is het niet relevant om de effectiviteit (2e zeef) nog te beoordelen;
- als de preventiemaatregel niet effectief (2e zeef) is, is implementatie (3e zeef) van de preventiemaatregel niet meer aan de orde.

Overzicht 29: zeefstructuur van het beoordelingsmodel

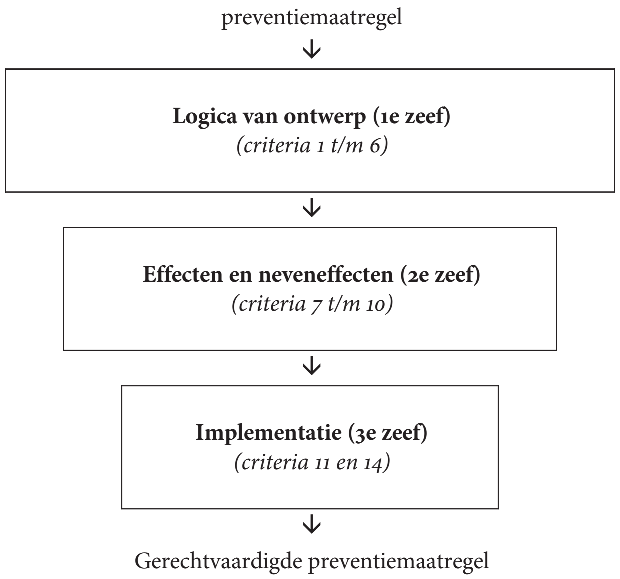

Toelichting op de volgorde van de beoordelingscriteria (§3.3)

Het ontwikkelde beoordelingsmodel is een argumentatief model. Ten behoeve van de rationaliteit van de beoordeling dient binnen het model zoveel mogelijk gebruik te worden gemaakt van wetenschappelijke gegevens ter onderbouwing van de argumentaties. Met het oog op de objectiviteit, vergelijkbaarheid en generaliseerbaarheid van de beoordeling, verdient het gebruik van kwantitatieve onderzoeksgegevens hierbij de voorkeur boven het gebruik van kwalitatieve onderzoeksgegevens. In overzicht 30 wordt mijn visie op de juiste volgorde van de argumentatie in een rationeel beoordelingsproces schematisch weergegeven.

Overzicht 30: fasering van de argumentatie in een rationeel beoordelingsproces

1. Argumenten op basis van *kwantitatieve* wetenschappelijke onderzoeksgegevens

↓

2. Argumenten op basis van *kwalitatieve* wetenschappelijke onderzoeksgegevens

↓

3. Niet-wetenschappelijk-bewezen argumenten die niet in strijd zijn met wetenschappelijke onderzoeksgegevens of met de logica.

Voor het kwantificeren c.q. uitdrukken in geld van immateriële zaken - zoals overlast, inbreuk op het persoonlijk leven en schending van morele opvattingen – kan gebruik worden gemaakt van concepten als 'Willingness to Pay' en Willingness to Accept' (§6.6.1).

Niet-wetenschappelijk bewezen argumenten zijn bijvoorbeeld argumenten van morele of politiek-ideologische aard, zoals het verbieden van harddrugsgebruik omdat men harddrugsgebruik moreel verwerpelijk acht.

De volgorde van de beoordelingscriteria in mijn model wordt bepaald door twee ordeningsprincipes:

- de fasering van de argumentatie van een rationeel beoordelingsproces, zoals weergegeven in overzicht 30;
- de logische volgorde van de beoordelingscriteria vanuit inhoudelijk perspectief.

Voor de rationaliteit van mijn beoordelingsmodel acht ik 'de logische volgorde van de beoordelingscriteria vanuit inhoudelijk perspectief' het belangrijkst. Om aan beide ordeningsprincipes tegemoet te komen, heb ik de criteria van het model geclusterd:

- de volgorde van de clusters en de volgorde van de criteria binnen ieder cluster is gebaseerd op de logische volgorde vanuit inhoudelijk perspectief;
- de criteria binnen een cluster hangen inhoudelijk logisch samen;
- binnen de clusters is gestreefd naar een volgorde van de criteria conform overzicht 30.

Mijn streven naar een volgorde van de criteria binnen een cluster conform overzicht 30 heeft er bijvoorbeeld toe geleid dat ik in cluster 1 het criterium 'noodzakelijkheid' heb opgesplitst in een wetenschappelijk te objectiveren criterium

‘schadelijkheid’ en een wetenschappelijk niet te objectiveren criterium ‘noodzakelijkheid’[2].

Impliciet kent het model 7 clusters van 2 criteria:

- Cluster 1:
  1. Schadelijkheid
  2. Noodzakelijkheid
- Cluster 2:
  3. Oorzakelijkheid
  4. Verantwoordelijkheid
- Cluster 3:
  5. Gerichtheid
  6. Geschiktheid
- Cluster 4:
  7. Effectiviteit
  8. Indringendheid
- Cluster 5:
  9. Lasten-batenverhouding
  10. Rechtvaardigheid
- Cluster 6:
  11. Draagvlak
  12. Flankerend beleid
- Cluster 7:
  13. Controleerbaarheid
  14. Uitvoeringscapaciteit

De indeling van het beoordelingsmodel in 7 clusters van elk 2 criteria heb ik gebruikt voor het bepalen van de juiste volgorde van de beoordelingscriteria bij de bouw van het model. Om het model voor de gebruiker niet te complex te maken, heb ik ervoor gekozen om deze clusters in het model niet expliciet zichtbaar te maken. Door de ***dwingende structuur*** van het beoordelingsmodel – de criteria dienen in de gegeven vaste volgorde te worden beoordeeld – wordt de beoordelaar geholpen bij het beoordelen van de logische samenhang tussen de beoordelingscriteria.

2. In tegenstelling tot in het model is bijvoorbeeld in de Trechter van Dunning de ‘schadelijkheid’ geen afzonderlijk criterium, maar onderdeel van het criterium ‘noodzakelijkheid’.

IV.II Wijze van beoordeling

Gerechtvaardigdheid van de argumentatie

De gerechtvaardigdheid van de toepassing van een preventiemaatregel berust op twee pijlers:

- de gerechtvaardigdheid van de ***argumentatie*** die heeft geleid tot het besluit om de preventiemaatregel toe te passen;
- de gerechtvaardigdheid van de ***procedure*** (welke partijen kunnen op welk moment welke invloed uitoefenen op de besluitvorming?) die heeft geleid tot het besluit om de preventiemaatregel toe te passen.

Het beoordelingsmodel richt zich op de gerechtvaardigdheid van de argumentatie. Afhankelijk van de casus dient per criterium te worden afgewogen in hoeverre procedurele aspecten relevant zijn voor de inhoudelijke beoordeling van de gerechtvaardigdheid. Op zichzelf valt de beoordeling van de procedurele gerechtvaardigdheid buiten het kader van het model (§12.4.4).

In pluralistische samenlevingen is het niet mogelijk om harde, meetbare toetsingscriteria voor de beoordeling van de gerechtvaardigdheid van leefstijlbeïnvloedende preventiemaatregelen vast te stellen die algemeen worden aanvaard. Om niet de suggestie te wekken dat de gerechtvaardigdheid van een preventiemaatregel met behulp van het model wordt getoetst aan de hand van harde, meetbare criteria, spreek ik in het kader van mijn model over de beoordeling van de gerechtvaardigdheid en niet over de toetsing van de gerechtvaardigdheid. (Hoofdstuk 4)

Bij de beoordeling van de gerechtvaardigdheid van een preventiemaatregel worden in het kader van het model de ***redelijkheid en billijkheid*** van de argumentatie die ten grondslag ligt aan de toepassing van de preventiemaatregel beoordeeld. De begrippen redelijkheid en billijkheid zijn moeilijk strikt van elkaar te scheiden. Redelijkheid refereert vooral aan rationaliteit (rede = ratio = verstand) en billijkheid aan 'fairheid' (billijk = fair = eerlijk).

Redelijkheid en billijkheid van de argumentatie

Per criterium van het model worden de redelijkheid en billijkheid van de argumentatie die ten grondslag ligt aan de toepassing van de preventiemaatregel, beoordeeld. Deze beoordeling is eigenlijk een zoektocht naar ***falsificatie*** van de redelijkheid en billijkheid van de volgende aspecten van de argumentatie (§4.5):

1. ***Juistheid*** van de argumentatie (is de argumentatie niet strijdig met wetenschappelijke informatie of onlogisch?);

2. ***Volledigheid*** van de argumentatie (is geen beschikbare wetenschappelijke informatie en zijn geen relevante argumenten over het hoofd gezien of genegeerd?);
3. Kracht van het ***bewijs*** (is het bewijs voor de argumentatie sterk genoeg?);
4. ***Transparantie*** van het bewijs (is de argumentatie voor iedereen vrij en eenvoudig toegankelijk?);
5. ***Interne consistentie*** van de argumentatie (voldoet de preventiemaatregel aan de vastgestelde samenhang tussen de criteria van het model?);
6. Verhouding tot het ***schadebeginsel*** (is de preventiemaatregel niet moralistisch, perfectionistisch, of paternalistisch?)
7. Mogelijkheid van ***betere alternatieven*** (of er betere alternatieven zijn wordt beoordeeld aan de hand van het optimalisatiebeginsel dat hieronder wordt toegelicht).

Optimalisatiebeginsel

Als de argumentatie ter onderbouwing van de preventiemaatregel m.b.t. de aspecten 1 t/m 6 ernstig tekortschiet, is toepassing van de preventiemaatregel niet gerechtvaardigd. Als er betere alternatieven mogelijk zijn (7e aspect), betekent dat niet automatisch dat toepassing van de preventiemaatregel niet gerechtvaardigd is. Dat het ontwerp (1e zeef), de effecten en neveneffecten (2e zeef) of de implementatie (3e zeef) van de preventiemaatregel suboptimaal zijn, hoeft immers niet te betekenen dat deze niet goed genoeg zijn. Soms is een suboptimale toepassing van een suboptimaal ontworpen preventiemaatregel te verkiezen boven het helemaal niet toepassen van de preventiemaatregel: beter een half ei dan een lege dop. Als suboptimaliteit wordt geconstateerd is dat altijd een ***aansporing tot verbetering***. Bij de beoordeling van de preventiemaatregel aan de hand van het optimalisatiebeginsel wordt beoordeeld of er geen sprake is geweest van ***onachtzaamheid*** (onterecht geen oog hebben voor) of ***nalatigheid*** (onterecht niet handelen). Door hierbij uit te gaan van ***de gegeven mogelijkheden*** (bv. de beschikbare mensen en middelen) tracht ik een perfectionistische invulling van de beoordeling te voorkomen. (§13.3.1 en 15.2.9)

Schadebeginsel

Vrij vertaald stelt het schadebeginsel dat de vrijheid van mensen alleen mag worden ingeperkt om te voorkomen dat zij schade berokkenen aan derden. Dit is een zeer liberaal uitgangspunt, dat veel mensen te ver gaat. Ik ga uit van een ***relatieve opvatting van het schadebeginsel***. Dit betekent dat ik paternalistische en moralistische vrijheidsbeperkingen niet rücksichtslos afwijs, maar enkel stel dat deze minder sterk gerechtvaardigd zijn dan vrijheidsbeperkingen ter voorkoming van schade. Op basis van een relatieve opvatting van het schadebeginsel kom ik tot de

volgende ***algemeen aanvaarde uitgangspunten*** voor de beoordeling van de gerechtvaardigdheid van vrijheidsbeperkingen (§4.3.5):

- Preventie van schade aan rechtmatige belangen vormt een sterkere rechtvaardiging van een leefstijlbeïnvloedende interventie dan bevordering van rechtmatige beleidsdoelen;
- Preventie van schade of overlast vormt een sterkere rechtvaardiging van een leefstijlbeïnvloedende interventie dan preventie van de schending van morele opvattingen;
- Preventie van schade die men berokkent aan derden vormt een sterkere rechtvaardiging van een leefstijlbeïnvloedende interventie dan preventie van schade die men berokkent aan zichzelf (als het gaat om 'dezelfde' schade);
- Preventie van schade die kinderen berokkenen aan zichzelf vormt een sterkere rechtvaardiging van een leefstijlbeïnvloedende interventie dan preventie van schade die volwassenen berokkenen aan zichzelf (als het gaat om 'dezelfde' schade);
- Naarmate de autonomie, keuzevrijheid, wilsbekwaamheid en geïnformeerdheid van mensen groter is, is paternalisme minder gerechtvaardigd.

Retrospectieve beoordeling (evaluatie)

Bij een retrospectieve beoordeling wordt teruggekeken naar de effecten en neveneffecten (2e zeef) en implementatie (3e zeef) van de preventiematregel, zoals die daadwerkelijk hebben plaatsgevonden. Een retrospectieve beoordeling kan daarom alleen tijdens of na afloop van de implementatie van een preventiemaatregel plaatsvinden. In de effecten en neveneffecten die zijn opgetreden, zit de invloed van de implementatie automatisch verwerkt. Als de beoordeling een periode na afloop van de implementatiefase betreft, kunnen de criteria van de 3e zeef (implementatie) nog steeds relevant zijn voor de beoordeling. (§13.7.2)

Prospectieve beoordeling (voorspelling)

Bij een prospectieve beoordeling wordt vooruitgekeken naar de effecten en neveneffecten (2e zeef) en implementatie (3e zeef) van de preventiemaatregel, die nog moeten plaatsvinden. Een prospectieve beoordeling kan alleen plaatsvinden op basis van aannames over de 'effecten en neveneffecten' en 'implementatie'. Het werken met aannames biedt de mogelijkheid om te werken met implementatiescenario's (bijvoorbeeld veel versus weinig inspanning op het gebied van 'handhaving'). Ook is het mogelijk om via experimenten of pilotstudies de 'effecten en neveneffecten' en 'implementatie' van een preventiemaatregel in een gecontroleerde en/of afgebakende omgeving uit te proberen en te onderzoeken. (§13.7.2)

V Samenvatting van de discussie

V.1 Beschouwing van mijn onderzoek en beoordelingsmodel

In de discussie (§15.2) heb ik de volgende aspecten van mijn onderzoek en beoordelingsmodel besproken:
- objectiviteit en volledigheid;
- neutraliteit;
- validiteit;
- betrouwbaarheid;
- praktische toepasbaarheid;
- algemene geldigheid;
- reikwijdte.

Hieronder geef ik de belangrijkste conclusies:
- Objectiviteit van de modelontwikkeling (§15.2.1), volledigheid van het model (§15.2.1) en neutraliteit van de wijze van beoordeling (§15.2.2) zijn voorwaarden voor een fair beoordelingsproces en daarmee voorwaarden voor de validiteit van het beoordelingsmodel (§15.2.3). Ik acht het aannemelijk dat het model aan deze voorwaarden voldoet.
- Dat de beoordeling aan de hand van het model is gebaseerd op de falsificatie van aangevoerde argumenten en niet op het bewijzen van de juistheid van aangevoerde argumenten, vormt tot op zekere hoogte een waarborg voor de betrouwbaarheid van de beoordelingen aan de hand van het model. Om met (meer) zekerheid een uitspraak te kunnen doen over de betrouwbaarheid dienen veel preventiemaatregelen door veel verschillende beoordelaars te zijn beoordeeld. (§15.2.4)
- Het is gelukt om aan de hand van het model tot een daadwerkelijke beoordeling van de gerechtvaardigdheid van twee praktijkcases (het preventieplan van Clarian Health en het wettelijk rookverbod in de horeca) te komen. Op basis hiervan acht ik het aannemelijk dat het model praktisch toepasbaar is. (§15.2.5)
- De algemene geldigheid van het model acht ik aannemelijk. Ik acht het onwaarschijnlijk dat toetsing van het model aan nieuwe casuïstiek nog tot essentiële veranderingen aan het model zal leiden. (§15.2.6)
- Ik vermoed dat de reikwijdte van het model zich uitstrekt tot alle situaties waarbij wordt getracht de leefstijl of het gedrag van mensen met ***drang of dwang*** te beïnvloeden (dus bijvoorbeeld ook het instellen en handhaven van leerplicht of van een burkaverbod).

- Ik denk dat de reikwijdte c.q. relevantie van het model zich beperkt tot situaties waarbij wordt getracht de leefstijl of het gedrag van mensen met ***drang of dwang*** te beïnvloeden. (§15.2.7)

V.II Relevantie van mijn onderzoek en beoordelingsmodel

Voor zover ik weet is er nog geen model (§15.3.1):
- waarmee de gerechtvaardigdheid van leefstijlbeïnvloedende preventiemaatregelen ter bestrijding van leefstijlgerelateerde aandoeningen op een algemeen aanvaardbare wijze kan worden beoordeeld, én
- waarvan de criteria zodanig geoperationaliseerd zijn dat ze praktisch toepasbaar zijn, én
- waarvan de juistheid, volledigheid en praktische toepasbaarheid aannemelijk zijn gemaakt door toepassing van het model op casuïstiek uit de praktijk.

Een dergelijk model hoop ik nu te bieden.

Het model kan worden gebruik door de (§15.3.2):
- landelijke en gemeentelijke overheid (VWS, RVZ, GGD-en) en politiek;
- burgers;
- bedrijven (denk hierbij bijvoorbeeld aan werkgevers en zorgverzekeraars);
- zorginstellingen en zorgverleners;
- onderzoeksinstellingen (RIVM, NIGZ);
- rechterlijke macht en advocatuur (denk hierbij bijvoorbeeld aan de rechtszaken tegen het wettelijk rookverbod voor kleine cafés zonder personeel).

Zij kunnen het model gebruiken voor het:
- beoordelen van de gerechtvaardigdheid,
- onderling vergelijken,
- ontwerpen,
- onderzoeken, en
- argumentatief onderbouwen of bekritiseren van leefstijlbeïnvloedende preventiemaatregelen, als er sprake is van drang of dwang.

Ter afsluiting van de discussie worden ***aanbevelingen*** gedaan ten behoeve van het beleid (§15.3.3) en de wetenschap (§15.3.4).

Summary

The summary of my PhD thesis is structured as follows:

I. Introduction
II. Research description
III. Assessment of cases from day-to-day practice
IV. Presentation of the assessment model
V. Consideration of the research and assessment model

I Introduction

Lifestyle-related diseases are increasingly posing a risk to public health across the globe in both relative and absolute terms. In relative terms because today the majority of infectious diseases which in the past posed a threat to public health can be successfully controlled, and in absolute terms because a large proportion of the population is leading an increasingly unhealthier lifestyle in part as a consequence of growing prosperity. An unhealthy lifestyle includes for instance smoking, overconsumption, an unhealthy diet, insufficient physical exercise and excessive alcohol consumption.

The prevention of lifestyle-related diseases can be carried out in a variety of ways. Obesity, for instance, could be prevented, by:

- encouraging or obliging people to take more physical exercise;
- encouraging or obliging people to follow a diet;
- encouraging or obliging manufacturers to reduce the quantity of sugar and fats, particularly unhealthy fats in food;
- introducing compliance regulations for the size of portions available in supermarkets and provided by the catering industry;
- imposing restrictions on advertising food, drinks, and stimulants
- etc.

While the effectiveness of several of the above measures is debatable, on the other side of the coin it is becoming increasingly clearer that the provision of information alone usually fails to spur people to lead healthier lives. A growing number of scientists are therefore advocating that various means be employed ***to***

force or pressurise the public into developing healthier behaviour and to spur the food industry to offer a healthier range of products and curtail their marketing ploys in luring people to over-consume. Prevention measures aimed at influencing or even prohibiting the production, distribution and consumption of food, drinks and stimulants, however, encroach on the population's freedom of choice and on free enterprise. To what extent is the application of such measures justified and how can this be assessed? The question prompted me to develop a model for the purpose of assessing the justification of lifestyle-influencing interventions aimed at controlling lifestyle-related diseases.

II Research description

II.I Definition of the problem and research question

The risks of prevention measures aimed at controlling lifestyle-related diseases for the population's freedom of choice, the financial interests of the business community, and the free movement of goods and services, raise a key question and that is: to what extent are prevention measures justified? On the basis of the definition of the problem, I formulated a research question when I commenced my research in 2005. On the basis of having gained enhanced understanding, I fine-tuned the research question on a number of occasions while performing the research (§1.2). The fourth and final version of the research question is as follows:

Final version of the research question (2008)

> Can a model be developed for the purpose of assessing the justification of prevention measures that seek to influence people's lifestyles in an effort to control lifestyle-related diseases?

II.II Focus on justification of the line of reasoning

In my view two pillars underpin the justification of applying a prevention measure as follows:

1. justification of the ***line of reasoning*** leading to the decision to apply the prevention measure;
2. justification of the ***procedure*** (what influence can be exerted on decision-making by what parties and when?) leading to the decision to apply the prevention measure.

My assessment model focuses on justifying the line of reasoning. Depending on the case, consideration should be given to the extent to which procedural aspects are relevant for substantively assessing justification of the prevention measure. The assessment of the procedural justification in itself falls outside the scope of my assessment model (§12.4.4).

II.III Research approach and methods

Research approach

In order to answer the research question, I conducted research, which comprised two parts:

- developing the assessment model on the basis of a ***literature search*** (Chapters 1-4);
- empirical testing of the assessment model on the basis of ***cases from day-to-day practice*** (Chapter 5-14).

In terms of content, the literature search was broken down into three parts:

- research into established assessment models which can be used to assess the development of the proposed model (Chapters 2 and 3);
- research into the assessment principles underlying the proposed model (Chapter 4);
- research into examples of the prevention of lifestyle-related diseases in day-to-day practice (Appendix 6).

I tested the accuracy, completeness and practical application of the model I developed on the basis of two cases from day-to-day practice:

- the prevention plan of Clarion Health Partners, one of the largest collaborative hospital organisations in the USA (Chapters 5-9);
- the statutory smoking ban in the Dutch catering industry (Chapters 10-14).

Why these cases are chosen, is explained at the beginning of Chapter 5 (Clarian) and at the beginning of Chapter 10 (smoking ban in the catering sector).

While empirically testing my model on the basis of cases from day-to-day practice, I operationalised the criteria for the model and determined the assessment method for each criterion. For the purpose of operationalising the criteria and determining the corresponding assessment method, I used the literature that I had studied as well as the cases referred to above.

Description of the Clarian Health case

In 2007 the collaborative US Hospital organisation Clarian Health announced plans to launch a completely new approach to reduce the annual health-care costs of its employees in 2009. Clarian's plan entailed imposing a financial penalty on its employees for leading an unhealthy lifestyle. To that end Clarian had established the following unhealthy life-style indicators: smoking, seriously overweight, high blood pressure, high cholesterol and glucose levels, on the basis of which employees would be penalised each month. Clarian's prevention plan generated much consternation in the media, with news pages and weblogs on the Internet devoting an enormous amount of coverage to the plan. The numerous written responses to the prevention plan afforded me a unique opportunity to test the accuracy, completeness and practical application of my model empirically. To that end I assessed the justification of Clarian's prevention plan on the basis of the criteria applied in my model.

Description of the case of the statutory smoking ban in the Dutch catering industry

'The proof of the pudding is in the eating'. That's why I tested the accuracy, completeness and practical application of my model once again by assessing the justification of the statutory smoking ban in the catering industry on the basis of my model. The Dutch government imposed the statutory smoking ban on the Dutch catering industry, which entered into force on 1 July 2008. I have therefore based the assessment of the justification of the smoking ban on the official documents which the Dutch government has used to date to substantiate the formulation and application of the smoking ban. My literature search into the government's reasons for substantiating the introduction and enforcement of the smoking ban covers a period of over 25 years (November 1984 - June 2011). The meeting documents of the Lower House and Upper House of Dutch Parliament, news messages on the Ministry of Health, Welfare and Sport website and relevant laws and regulations (§10.2) formed the core of my literature search.

Research methods

I used three research methods (§1.5) to perform my research as follows:

- literature search;
- argumentative text analysis;
- reasoning.

III Assessment of cases from day-to-day-practice

III.I Justification of the Clarian Health prevention plan

On the basis of the information available on the Internet about the Clarian Health prevention plan, the design (first filter of my model), the effects and side effects (second filter) and the implementation method (third filter) of the prevention plan ***are unjustifiable*** according to my model. Firstly, this is because insufficient information is available on many of the criteria in my model for the purpose of making a reliable assessment. And secondly, because based on the information that is available, the conclusion can be drawn that the application of the Clarian Health prevention plan in its present form is unjustifiable. The main points of criticism in terms of substance are as follows:

- I consider the US federal government's decision to give employers – albeit within a finite financial framework[1] – the opportunity to impose at their discretion a financial penalty on their own employees for leading an unhealthy lifestyle inside and outside working hours as highly disputable. In my view employers are not qualified to design and implement prevention measures of this nature. To avoid the inequality of rights among different employers' employees and employer arbitrariness, it would in my view be better for the democratically elected central government to decide on the design of such prevention measures. I believe it would be preferable to leave the implementation of such prevention measures to a trusted third party, such as a health insurer. (§6.7) In my view the intrusive nature of the prevention measure (structural infringement of physical integrity and privacy) cannot be justified for employees who do in fact conform to Clarian's health criteria. (§7.7)
- The fact that the prevention plan could create serious financial difficulties for Clarian employees (and their families) or make them default on the payment of their health insurance premium I regard as unjustifiable. A hardship clause may perhaps help resolve this situation (§7.7).

III.II Justification of the statutory smoking ban in the catering industry

Lack of clarity on the objective

The introduction of the smoking ban in the catering industry involved extensive public political debate. For this reason I find it odd that after having studied the parliamentary documents I found that the debate failed to create clarity on the

1. See Appendix 8: Explanatory notes to §f of the HIPAA.

objectives underlying the statutory smoking ban in the catering industry. The successive Ministers of Health, Welfare and Sport adopted different views on the objective underlying the catering industry smoking ban (§10.5).

In my view, in June 2007 the Lower House of Dutch Parliament should not have concurred with the statement made by Ab Klink, former Minister of Health, Welfare and Sport, who affirmed that visitor protection is a secondary objective of the statutory smoking ban in the catering industry. It emerged that the term 'secondary objective' could be interpreted in multiple ways. In July 2007 Mr Klink regarded 'a secondary objective' as an objective, in which case protecting visitors against passive smoking falls under the criterion 'risk of harm' in the first filter of my model. In July 2009 Mr Klink regarded 'a secondary objective' merely as a 'positive side effect', in which case protecting visitors against passive smoking falls under the criterion 'cost-benefit ratio' in the second filter of my model. I believe Mr Klink failed to adequately substantiate the change in views on the objective underlying the statutory smoking ban imposed on the catering industry.

In terms of the clarity of the objectives underlying the statutory smoking ban in the catering industry, the ban ***cannot pass*** through the ***first filter*** of my assessment model (logic of the design) during Mr Klink's reign as Health Minister. I regard the introduction of a statutory smoking ban in the catering industry without having clear underlying objectives as reflecting ***a lack of clarity***.

Considering that public amenities should be freely accessible to all members of the public, I believe that people visiting catering outlets should be given statutory protection against smoking. I therefore disagree with the position taken by the current caretaker Minister of Health, Welfare and Sport Edith Schippers who says that the objective of the statutory smoking ban in the catering industry should be confined to protecting catering industry employees against passive smoking. The position adopted by Ms Schippers fails to conform to the spirit (see under the criterion 'legitimacy' in §12.4.2) of:

- the Tobacco Act;
- the WHO Framework Convention on Tobacco Control (2003);
- the agreements made by Hans Hoogervorst, former Minister of Health, Welfare and Sport, with Koninklijk Horeca Nederland, the trade association for the catering industry in the Netherlands, concerning the action plan aimed at the self-regulation of smoking policy in the catering industry (2004);
- the Recommendation formulated by the Council of Europe on smoke-free environments (2009).

My personal view on the need to ensure the statutory protection of visitors to catering outlets against passive smoking, however, is irrelevant in the context of my model. I regard the fact that the position adopted by Dutch Health Minister Edith Schippers does not conform to the spirit of the above four documents as an inadequate reason for acknowledging that her position is unjustifiable. In a formal legal sense, in my view the above documents do not – insurmountably – impede the position adopted by the Health Minister Edith Schippers.

In terms of the clarity of the objectives underlying the statutory smoking ban in the catering industry, the ban ***can in fact pass*** through the ***first filter*** of my assessment model (logic of the design) during the reign of Edith Schippers as Health Minister. The Health Minister's view is ***clear*** (transparent) and cannot be interpreted in multiple ways. According to Ms Schippers the objective of the statutory ban in the catering industry should, and is in fact confined to protecting employees against passive smoking.

Formal wording of the Act as a starting point for assessing the statutory smoking ban

Employee protection as a starting point for the assessment

Due to the lack of clarity on the objective of the statutory smoking ban in the catering industry during Ab Klink's reign as Health Minister, I assessed the justification of the statutory ban on the basis of my model, taking the formal wording of the Act as a starting point. The formal wording of the Act only offers employees protection against passive smoking (§10.5). From the perspective of my model, the limited objective of the statutory smoking ban in the catering industry in itself is not unjustifiable (§10.6.4).

Logic of the design (first filter of the model)

The statutory smoking ban in the catering industry ***can pass through*** the first filter with the objective of protecting employees in the catering industry against passive smoking: the design is logical. It is not moralistic, it is not perfectionistic nor is it paternalistic.

Effects and side effects (second filter of the model)

The statutory smoking ban in the catering industry as introduced by the former Health Minister Ab Klink on ***1 July 2007 cannot pass through*** the second filter: one of the possible or actual side effects is unfair. It is important to note that the observed unfairness relates only to a ***minor*** part of the catering industry – the small cafés which have no staff. The aspect of 'hardship in specific cases' under the

‘justification’ criterion serves as the breaking point for justifying the smoking ban in small cafés which have no staff. In relation to the objective of the smoking ban for small cafés which have no staff – i.e. the prevention of unfair competition with cafés with staff – the financial implications for these types of cafés could possibly be disproportionate. I have said ‘possibly’ because no financial validation is available. I have taken into account that there is less unfair competition in day-to-day practice than expected. As soon as small cafés that have no staff attract more visitors on account of the fact that visitors are allowed to smoke in these establishments, the small cafés will indeed need to employ staff. This, in turn, means that the smoking ban will apply with immediate effect.

The current statutory smoking ban in the catering industry, which excludes small cafés which have no staff, ***can pass through*** the second filter (effects and side effects).

Implementation method (third filter of the model)
The statutory smoking ban in the catering industry ***cannot pass*** through the third filter. The manner in which the ban was implemented is unreasonable and unfair in certain areas.

In implementing the statutory smoking ban in the catering industry, in my view the former Health Minister Ab Klink bungled on too many occasions, which makes it difficult to acknowledge that the implementation method is justifiable. On the one hand he had little consideration for ensuring continued and increasing support for the statutory smoking ban in the catering industry from *Koninklijk Horeca Nederland*, the trade association for the catering industry in the Netherlands, and from catering businesses. For more information, see the criteria ‘timing’ (§13.1.3) and ‘support’ (§13.2.3) under the heading ‘Conclusion’. On the other hand, he paid little attention to the supposed unfavourable financial consequences of the implementation of the ban for small cafés which have no staff. For more information, see the criteria ‘complementary policies’ (§13.3.3) and ‘verifiability’ (§13.4.3) under the heading ‘Conclusion’.

In respect of the implications for enforcing the smoking ban, in my view by exempting small cafés which have no staff from the smoking ban the Dutch Health Minister Edith Schippers was ***careless and negligent*** in relaxing the statutory smoking ban in the catering industry. For more information, see the criterion ‘enforceability’ (§13.4.3) under the heading ‘Conclusion’. It is important to note that the carelessness and negligence observed relates only to part of the catering industry - the cafés. The carelessness and negligence, however, form an

inadequate reason for acknowledging that the relaxation of the statutory smoking ban implemented by the Health Minister Edith Schippers is unjustifiable. However, what does apply is the ***sub-optimal implementation of relaxing*** the statutory smoking ban by the Health Minister Edith Schippers.

IV Presentation of the assessment model

The definitive research question I have endeavoured to answer is as follows (§1.4):

> Can a model be developed for the purpose of assessing the justification of prevention measures that seek to influence people's lifestyles in an effort to control lifestyle-related diseases?

I have answered the research question by presenting the definitive version of my model in §15.1.

The model comprises three components:
- ***assessment criteria:***
  - operationalisation of the criteria (§15.1.1).
- ***assessment structure:***
  - assessment filters (explicit assessment structure) (§15.1.2);
  - assessment clusters (implicit assessment structure) (§15.1.2);
- ***assessment method:***
  - assessment method in broad outline (§15.1.3);
  - assessment method detailed by criterion (§15.1.1).

IV.1 Assessment criteria

The development of versions 1-6 of my model is primarily based on literature relating to established assessment models (Chapters 2-4) whereas the development of versions 7-9 is based on testing my model against cases from day-to-day practice (Chapters 7-14). The definitive assessment criteria are shown in Figure 28 below.

Figure 28: Definitive assessment criteria of my model

Prevention measure

↓

Design logic (first filter)

1. Risk of harm
2. Necessity
3. Causality
4. Responsibility
5. Focus
6. Suitability

↓

Effects and side effects (second filter)

7. Effectiveness
8. Intrusiveness
9. Cost-benefit ratio
10. Fairness

↓

Implementation (third filter)

11. Support
12. Complementary policies
13. Verifiability
14. Implementation capacity

↓

Justified prevention measure

The operationalised criteria are set out below in key words (for a detailed description, see §14.2.1).

1. Risk of harm criterion

- What harm, disturbance or nuisance or infringement of moral views does the prevention measure endeavour to control?

2. Necessity criterion

- To what extent is the prevention measure necessary?
- To what extent is the prevention measure moralistic or perfectionistic?
- Are penalties imposed on the persons or organisations causing the risks?

3. Causality criterion

- To what extent do the lifestyles targeted by the prevention measure form the driver of the risks that one seeks to control?
- What are the driving factors behind the lifestyles targeted by the prevention measure and to what extent are they determinant?

4. Responsibility criterion

- What parties are held responsible for creating or maintaining the risks that one seeks to control?
- In determining responsibility, have all known risk drivers (third criterion) been taken into account?
- To what extent is the prevention measure paternalistic?

5. Focus criterion

- Is the choice of target group and life-style influencing factors logically related to the risk drivers (third criterion) and responsibility (fourth criterion) for these risks?
- Have people in the target group been overlooked by the prevention measure or have they fallen outside the target group by the prevention measure?

6. Suitability criterion

- Does the choice of parties implementing the prevention measure relate logically to the focus (fifth criterion) of the prevention measure?
- Are the parties implementing the prevention measure competent, authorised and suitable for doing so?
- Will implementation of the prevention measure not harm the nature or the reputation of the parties that are implementing the measure?

7. Effectiveness criterion

- To what extent have the intended effects of the prevention measure been achieved?

8. Intrusiveness criterion

I. General assessment:
   1. To whom does the intrusion of private life apply?
   2. Who is responsible for the intrusion of private life?
   3. To what extent does the intrusion of private life apply?
   4. How significant is the intrusion of private life?
   5. Is allowing the intrusion of private life creating a precedent?
   6. How inconvenient is the intrusion of private life?

II. Assessment of various aspects:
   a. infringement of physical integrity;
   b. violation of liberties;
   c. infringement of privacy;
   d. infringement of perceived safety;
   e. discrimination;
   f. stigmatisation.

III. Assessment of the degree of pressure or force applied.

9. Cost-benefit ratio

- What are the costs and benefits of the prevention measure and what is the cost-benefit ratio (ratio of burdens to benefits)?

10. Fairness criterion

- Are the costs distributed across the parties in proportion to their level of responsibility for the risk of damage?
- Are the parties treated equally in the same cases?
- Have people's means been taken into account?
- Has account been taken of people's personal circumstances or the specific circumstances of organisations?
- Have the equal opportunities of the parties involved been affected in respect of their access to health care and prevention?
- Has the distribution of the available health-care and prevention funds been influenced in line with needs?
- Does the prevention measure not violate the current Act or legally effective agreements?

11. Support criterion

- Is it the right time to apply pressure or force?
- Have people and organisations been gradually prepared for the prevention measure?
- To what extent has emphasis been placed on garnering support for the prevention measure?
- Are the current political, economic and social circumstances appropriate for implementing the prevention measure?
- Is the prevention measure consistent with the policies pursued by stakeholders?
- How much support is there for the prevention measure?

12. Complementary policies criterion

- Has the prevention measure been anchored in broader prevention or other policies?
- Is there no policy that conflicts with the prevention measure?
- How is information provision concerning the prevention measure organised?
- How is enforcement of compliance with the prevention measure organised?
- Will constraints be identified and resolved during implementation?

13. Verifiability criterion

- Are the 'design', the 'effects and side effects' and 'implementation' of the prevention measure monitored and evaluated?

14. Implementation capacity criterion

- Have a sufficient number of people and funds been made available to ensure that implementation of the prevention measure is feasible and sustainable?

IV.1 Structure of the assessment model

I derived the concept of the filter structure from the '*Trechter van Dunning*', an assessment model based on the concept of a 'funnel' with successive filters (the literal translation of '*trechter*' is 'funnel') developed in 1991 by the Committee on Choices in Health-Care (*Commissie Keuzen in de Zorg*) headed by the late Dutch cardiologist A.J. Dunning.

The first reason for applying the principle of a 'funnel' with successive filters is that the ***sequence*** in which the assessment criteria in my model are used has a huge impact on the substance, and consequently the ***validity*** of the assessment process. The clustering of the assessment criteria in filters clearly shows why the sequence of the criteria is vital.

The second reason for applying the principle of a 'funnel' with successive filters is that it is easier to manage the total number of 14 criteria. If information fails to pass through a certain filter, the underlying criteria need no longer be examined.

The third reason for applying the 'funnel' principle with successive filters is as follows. ***The criteria in a filter may be weighed against each other.*** This means that a good score for a certain criterion will help compensate for a poor score on another criterion. ***The criteria originating from the various filters are not permitted to be weighed against each other.***

The following two examples serve to illustrate the above:

- If prevention is unnecessary (first filter), it is irrelevant to subsequently assess effectiveness (second filter).
- If the prevention measure is ineffective (second filter), implementation of the prevention measure no longer applies (third filter).

Figure 29: Filter structure of the assessment model

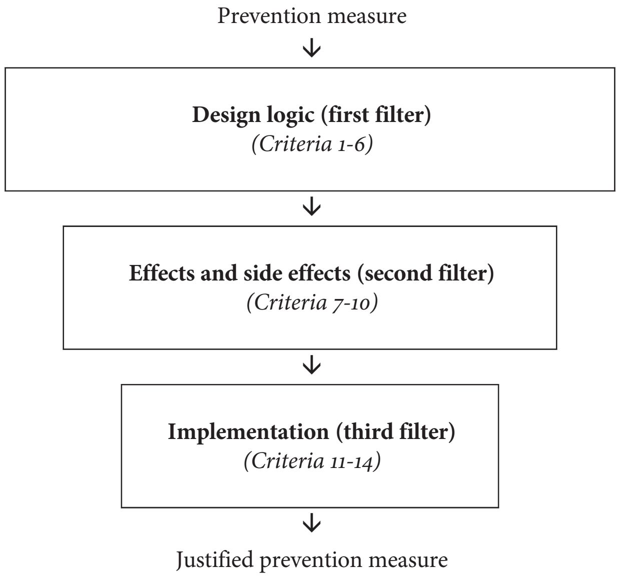

Explanatory notes on the sequence of the assessment criteria (§3.3)

The assessment model that has been developed is an argumentative model. To ensure the rationality of the assessment, as much scientific data as possible should be used in the model to substantiate reasoning. Taking the objectiveness, comparability and the ability to generalise the assessment into consideration, it would preferable to use quantitative rather than qualitative research data. Figure 30 reflects my views on the correct sequence of the reasoning in a rational assessment process.

Figure 30: Stages of reasoning in a rational assessment process

1. Arguments on the basis of *quantitative* scientific research data
   ↓
2. Arguments on the basis of *qualitative* scientific research data
   ↓
3. Non-scientifically proven arguments that do not conflict with scientific research data or logic.

To quantify or express intangible matters, such as disturbance, the intrusion of private life and the infringement of moral views, concepts such as the 'Willingness to Pay' and the 'Willingness to Accept' (§6.6.1) may be used.

Non-scientifically proven arguments, for instance, are those of a moral nature or relating to political ideologies, such as prohibiting the use of hard drugs because the use of hard drugs is deemed morally reprehensible.

Two classification systems are used to determine the sequence of the assessment criteria in the model as follows:
- the stages of reasoning in a rational assessment process, as reflected in the diagram in Figure 30;
- the logical sequence of the assessment criteria in terms of substance.

I consider the logical sequence of the assessment criteria in terms of substance as the most important in ensuring the rationality of the assessment model. To comply with both classification systems, I have clustered the criteria in the model as follows:
- the sequence of the clusters and the criteria within each cluster is based on the logical sequence in terms of substance;
- in terms of substance the criteria in a cluster are logically coherent;
- the sequence of the criteria in the clusters aims to reflect the sequence set out in Figure 30.

My endeavour to use the sequence of the criteria in a cluster as set out in Figure 30 prompted me to split up the criterion 'necessity' in cluster 1 in the scientifically

objectifiable criterion ‘risk of harm’ and the scientifically non-objectifiable criterion ‘necessity’[2].

My model ***implicitly*** features seven clusters each containing two criteria as follows:

- Cluster 1:
  1. Risk of harm
  2. Necessity
- Cluster 2:
  3. Causality
  4. Responsibility
- Cluster 3:
  5. Focus
  6. Suitability
- Cluster 4:
  7. Effectiveness
  8. Intrusiveness
- Cluster 5:
  9. Cost-benefit ratio (ratio of burdens to benefits)
  10. Fairness
- Cluster 6:
  11. Support
  12. Complementary policies
- Cluster 7:
  13. Verifiability
  14. Implementation capacity

I used the breakdown of my assessment model into seven clusters with two criteria each to determine the correct sequence of the assessment criteria when building my model. To avoid making my model too complicated for the user, I explicitly opted not to make these clusters visible in the model.

The ***imperative structure*** of the assessment model – which requires assessment of the criteria in a fixed sequence – helps the assessor to assess the logical relationship between the assessment criteria.

2. Contrary to my model, in the ‘*Trechter van Dunning*’ (‘funnel’ assessment model developed by the Committee on Choices in Health-Care) for example, ‘risk of harm’ is not a separate criterion but forms part of the criterion ‘necessity’.

IV.11 Assessment method

Justification of the line of reasoning

Two pillars underpin the justification of applying a prevention measure as follows:

- justification of the ***line of reasoning*** leading to the decision to apply the prevention measure;
- justification of the ***procedure*** (what influence can be exerted on decision-making by what parties and when?) leading to the decision to apply the prevention measure.

My assessment model focuses on justifying the line of reasoning. Depending on the case, consideration should be given to the extent to which procedural aspects are relevant for substantively assessing justification of the prevention measure. Assessment of the procedural justification in itself falls outside the scope of my assessment model (§12.4.4).

In pluralist societies, it is impossible to determine measurable testing criteria to assess the justification of generally accepted lifestyle-influencing prevention measures. To avoid implying that the justification of a prevention measure with the aid of my model is tested on the basis of firm, measurable criteria, I refer to 'assessing' justification rather than to 'testing' justification for the purpose of my model. (Chapter 4).

Assessment of the justification of a prevention measure in my model is based on the principles of ***reasonableness and fairness*** of the line of reasoning underlying the application of the prevention measure. It is difficult to strictly separate the terms 'reasonableness' and 'fairness'. 'Reasonableness' refers primarily to rationality', i.e. reason = ratio = power of reason, while 'fairness' refers to fair = honest.

The reasonableness and fairness of the line of reasoning

The reasonableness and fairness of the line of reasoning underlying the application of the prevention measure have been assessed for each criterion. The assessment actually represents a quest to uncover the ***falsification*** of the reasonableness and fairness of the following aspects of the line of reasoning (§4.5) as follows:

1. ***Accuracy*** of the line of reasoning (does the line of reasoning not conflict with scientific information or is it illogical?);
2. ***Completeness*** of the line of reasoning (have any relevant arguments and available scientific information been overlooked or ignored?);

3. Robustness of the ***evidence*** (is the evidence of the line of reasoning sufficiently strong?);
4. ***Clarity*** of the evidence (is the line of reasoning freely and easily available to all parties?);
5. ***Internal consistency*** of the line of reasoning (does the prevention measure conform to the relationship established between the criteria of my model?);
6. Relation to the ***harm principle*** (is the prevention measure not moralistic, perfectionistic or paternalistic?);
7. The possibility of providing ***better alternatives*** (whether there are any better alternatives is assessed on the basis of the optimisation principle described in detail below).

Optimisation principle
If the line of reasoning that is used to substantiate the prevention measure concerning aspects 1-6 seriously fails, the application of the prevention measure is unjustifiable. If better alternatives are possible (seventh aspect), this does not automatically imply that the application of the prevention measure is unjustifiable. The fact that the design (first filter) and the effects and side effects (second filter) or the implementation (third filter) of the prevention measure are sub-optimal does not necessarily imply that they are not good enough. The sub-optimal application of a sub-optimally designed prevention measure might occasionally be preferred above non-application of the prevention measure, i.e. 'half a loaf is better than none'. If a prevention measure is found to be sub-optimal, this will always ***help spur improvement***. Assessment of the prevention measure by means of the optimisation principle involves assessing whether ***carelessness*** (wrongly not taking aspects into account) or ***negligence*** (wrongly failing to act) are found to have been the case. By taking the ***given possibilities*** as the starting point (e.g. the available people and funds), I have endeavoured to avoid shaping the assessment in a perfectionistic manner (§13.3.1 and 15.2.9).

Harm principle
Freely translated the harm principle entails that people's liberties may only be curtailed to avoid them inflicting harm on third parties. This is quite a liberal starting point, which many people may feel is pushing the limits. I have assumed a ***relative view on the harm principle***. This means that I have not unscrupulously rejected the paternalistic and moralistic restriction of liberty but merely wish to point out that this is less strongly justifiable than the restriction of liberty to prevent harm. Taking a relative view of the harm principle I have arrived at the following ***generally accepted starting points*** for the purpose of assessing the justification of the restriction of liberty (§4.3.5):

- preventing harm to lawful interests justifies a lifestyle-influencing intervention more strongly than promoting lawful policy targets;
- Preventing harm or disturbance justifies a lifestyle-influencing intervention more strongly than preventing the infringement of moral views;
- preventing harm inflicted on third parties justifies a lifestyle-influencing intervention more strongly than preventing harm inflicted on oneself (if this involves the 'same' type of harm);
- preventing harm inflicted by children on themselves justifies a lifestyle-influencing intervention more strongly than preventing harm inflicted by adults on themselves (if this involves the 'same' type of harm);
- as people's autonomy, freedom of choice, competency to express their will grows and as they acquire more information, there is less justification for paternalism.

Retrospective assessment (evaluation)

A retrospective assessment takes a retrospective view of the effects and side effects (second filter) and implementation (third filter) of the prevention measure in the situation in which these actually took place. A retrospective assessment can therefore only be performed during or after a prevention measure has been implemented. The influence of implementation has automatically been incorporated in the effects and side effects that occurred. If the assessment relates to a period after the implementation phase, the criteria in the third filter (implementation) are still relevant to the assessment (§13.7.2).

Prospective assessment (prediction)

A prospective assessment takes a predictive view of the effects and side effects (second filter) and implementation (third filter) of the prevention measure which have yet to take place. A prospective assessment can only be performed on the basis of assumptions on the 'effects and side effects' and 'implementation'. Assumption-based assessment offers the option to work with implementation scenarios (such as putting a great deal of effort versus little effort into 'enforcement'). It is also feasible to trial and research the 'effects and side effects' and 'implementation' of a prevention measure in a controlled and/or defined environment by performing experiments or pilot studies (§13.7.2).

V Summary of the discussion

V.1 Consideration of my research and assessment model

In the discussion (§15.2) I discussed the following aspects of my research and the assessment model:
- objectiveness and completeness;
- neutrality;
- validity;
- reliability;
- practical application;
- universality;
- scope.

Please find below the main conclusions:
- The objectiveness of developing the model (§15.2.1), the completeness of the model (§15.2.1) and the neutrality of the assessment method (§15.2.2) are prerequisites for ensuring the assessment process is fair and therefore are prerequisites for ensuring the validity of the assessment model (§15.2.3). I consider it plausible that the model meets the above prerequisites.
- The fact that an assessment performed with the model is based on the falsification of arguments put forward, rather than on proving the accuracy of arguments put forward, to a certain extent guarantees the reliability of the assessments performed on the basis of the model. To make a statement about the aspect of reliability with greater certitude many prevention measures should be assessed by a range of different assessors (§15.2.4).
- I managed to actually assess the justification of two cases from day-to-day practice (the Clarian Health prevention plan and the statutory smoking ban in the Dutch catering industry) using my model. On the basis of the above, I consider it plausible that the model can be applied in practice (§15.2.5).
- I consider the universality of the model plausible. I consider it unlikely that testing the model against new cases from day-to-day practice will lead to fundamental changes in the model (§15.2.6).
- I presume that the scope of the model covers all situations in which parties seek to influence people's lifestyles or behaviour by exerting ***pressure or force*** (including imposing or enforcing compulsory education or banning the burqa, for instance).
- I believe that the scope or relevance of the model is limited to situations in which parties seek to influence people's lifestyles or behaviour by exerting ***pressure or force*** (§15.2.7).

V.II Consideration of my research and the assessment model

To my knowledge no model currently exists (§15.3.1):

- which enables assessment of the justification of lifestyle-influencing prevention measures aimed at controlling lifestyle-related diseases in a generally accepted manner, and
- the criteria of which have been operationalised such that they can be applied in practice, and
- the accuracy, completeness and practical application of which have been demonstrated by applying the model to cases from day-to-day practice.

I now hope to provide such a model.

The model can be used by the following parties (§15.3.2):
- the national and municipal authorities (Ministry of Health, Welfare and Sport, the Council for Public Health and Care (RVZ), the Municipal Health Services (GGD) and in political circles;
- the public;
- companies (employers and health insurers, for instance);
- health-care institutions and care providers;
- research institutions (the National Institute for Public Health and the Environment (RIVM) and the Netherlands Institute for Health Promotion (NIGZ);
- the judiciary and the legal professional (an example is the legal proceedings instituted against the statutory smoking ban for small cafés which have no staff).

The above parties can use the model for the following purposes:
- assessing justification,
- comparative analysis,
- designing,
- examining and
- substantiating reasons or criticising

lifestyle-influencing prevention measures in situations where pressure or force is exerted.

To conclude the discussion, I have formulated ***recommendations*** for policy (§15.3.3) and scientific purposes (§15.3.4).

Bijlage 1: Toelichting van begrippen (Brug, 2004)

In de vorige eeuw heeft het zwaartepunt van de preventieve gezondheidszorg in Nederland gelegen op gezondheidsbescherming en ziektepreventie.

Gezondheidsbescherming (1a) is er op gericht de hele bevolking of grote delen daaruit te beschermen tegen blootstelling aan risicofactoren. Zuivering van drinkwater, controle van voedingsmiddelen door de keuringsdienst van waren, en een rookverbod in openbare ruimten om passief roken tegen te gaan, zijn hiervan voorbeelden.

Bij ***ziektepreventie (1b)*** staat een specifieke aandoening centraal. De van oudsher belangrijkste ziektepreventieprogramma's zijn gericht op het voorkomen van infectieziekten door isolatie van geïnfecteerde personen en vaccinatie. Ook programma's gericht op de vroege opsporing van aandoeningen behoren tot de ziektepreventie. Voorbeelden hiervan zijn screening op erfelijke aandoeningen, op borstkanker, op baarmoederhalskanker, op gehoorafwijkingen (in de jeugdgezondheidszorg) en op tandbederf. Op het gebied van gezondheidsbescherming en ziektepreventie heeft de overheid altijd een belangrijke stimulerende en uitvoerende rol op zich genomen.

Gezondheidsbevordering (1c) is gericht op het bevorderen van gezond gedrag, zoals stoppen met roken, veilig vrijen, gezonder eten, meer bewegen, enz.[1]. Gezondheidsbevordering maakt van oudsher vooral gebruik van ***gezondheidsvoorlichting*** om kennis over de voordelen van gezond gedrag en de risico's van ongezond gedrag over te dragen. De laatste decennia is duidelijk geworden dat het overdragen van kennis alleen vrijwel nooit voldoende motiveert tot gedragsverandering. Moderne gezondheidsbevordering start daarom met het in kaart brengen van alle biologische, maatschappelijke en psychologische determinanten van (on) gezond gedrag, omdat alle gedragsdeterminanten aanknopingspunten voor interventie in zich dragen.

1. Vergelijk dit met de BRAVO-leefstijlfactoren, die de overheid sinds 1998 in haar beleid hanteert: **B**ewegen, **R**oken, **A**lcohol, **V**eiligheid, **V**eilig vrijen, **V**oeding, **O**ntspanning (RVZ, 2002, p.10). Het door mij beoogde beoordelingsmodel beperkt zich niet tot de BRAVO-leefstijlfactoren c.q. sluit geen leefstijlfactoren uit. Met behulp van het model dient bijvoorbeeld ook de gerechtvaardigdheid van preventiemaatregelen ter bestrijding van drugsgebruik te kunnen worden beoordeeld.

Tegenwoordig wordt gezondheidsbevordering gedefinieerd als een combinatie van voorlichting en ***omgevingsverandering*** die (samen) gezond gedrag en gezonde leefomstandigheden stimuleren. Bij een omgevingsverandering ter bevordering van gezond gedrag kan bijvoorbeeld worden gedacht aan een ruimer en goedkoper aanbod van gezond eten in de horeca om gezonder eetgedrag te bevorderen. Vormen van gezondheidsbevordering die zijn gericht op omgevingsverandering, liggen inhoudelijk dicht tegen gezondheidsbescherming aan. Via het onderstaande voorbeeld probeer ik de grens tussen gezondheidsbevordering en gezondheidsbescherming, zoals ik die zie, te verhelderen. Ingeval van het realiseren van een ruimer aanbod van gezonde voedingsmiddelen in supermarkten of in de horeca is er sprake van gezondheidsbevordering. Mensen worden dan niet 'beschermd' tegen ongezonde voedingsmiddelen. Op het moment dat de verkoop van ongezonde voedingsmiddelen wordt bemoeilijkt of verboden, mensen worden hiertegen 'beschermd', is er sprake van gezondheidsbescherming.

Preventiemaatregelen kunnen ook worden ingedeeld naar het stadium van de aandoening waarin men preventief probeert in te grijpen. ***Primaire preventie (2a)*** is gericht op het voorkomen van nieuwe gevallen van een ziekte. ***Secundaire preventi***e ***(2b)*** is er op gericht om een aandoening in een zo vroeg mogelijk stadium te ontdekken zodat vroege behandeling mogelijk is en voorkomen kan worden dat de ziekte verergert. ***Tertiaire preventie (2c)*** is het voorkomen of beperken van de gevolgen van een reeds gediagnosticeerde aandoening. De afgrenzing tussen primaire, secundaire en tertiaire preventie is niet altijd eenduidig te maken.

Bij zowel primaire als secundaire preventie kan een onderscheid worden gemaakt tussen een collectieve en een opportunistische benadering. Bij ***collectieve preventie (3a)*** worden preventieve interventies ontwikkeld en geïmplementeerd gericht op de totale bevolking of grote deelpopulaties daaruit. Bij ***opportunistische preventie (3b)*** worden preventieve interventies toegepast als zich, bijvoorbeeld in een ander kader, daarvoor een goede gelegenheid voordoet. Het leefstijladvies door een huisarts aan een patiënt bij wie beginnend overgewicht is geconstateerd, is hiervan een voorbeeld.

In beleidsnotities van de overheid en zorgaanbieders wordt over het algemeen niet gesproken over collectieve preventie versus opportunistische preventie, maar over ***collectieve preventie (3c)*** versus ***individuele preventie (3d)***. In zijn brief aan de Tweede kamer over de invoering van Diagnose Behandel Combinaties in de Geestelijke Gezondheidszorg geeft Minister Hoogervorst van VWS de volgende

omschrijving van de begrippen individuele en collectieve preventie (Hoogervorst, 2004)[2]:

> *"De feitelijke preventieactiviteiten spelen zich echter op verschillend niveau af. Een gedeelte is gericht op individuen* ***(individuele preventie)*** *en/of gerelateerd aan behandeling van een individu (of is daar een integraal onderdeel van). Bij het andere gedeelte is sprake van vormen van* **collectieve preventie***, waarbij activiteiten meer op 'groepsniveau' worden aangeboden (bijvoorbeeld via leerkrachten op scholen, verpleegkundigen in verpleeghuizen, richting de bevolking als geheel of op risicogroepen)."*

Bij preventie kan ook een onderscheid worden gemaakt tussen een hoogrisicobenadering en een populatiebenadering. Bij een ***hoogrisicobenadering (3f)*** wordt eerst een risicogroep geïdentificeerd, bijvoorbeeld op basis van erfelijke eigenschappen, het veel voorkomen van risicogedrag, leeftijd, en dergelijke. Bij een ***populatiebenadering (3e)*** wordt de hele bevolking of een grote deelgroep daaruit benaderd.

2. Hoogervorst, H. Minister van VWS. Voortgangsrapportage DBC-GGZ. Kamerstuk. GVM/2522926. Den Haag, 14 oktober 2004.

Bijlage 2: Categorieën van preventiemaatregelen gecombineerd

	Primaire preventie		Secundaire preventie		Tertiaire preventie	
	Ongerichte preventie	Gerichte preventie	Ongerichte preventie	Gerichte preventie	Ongerichte preventie	Gerichte preventie
Gezondheidsbescherming	1	2	3	4	5	6
Ziektepreventie	7	8	9	10	11	12
Gezondheidsbevordering	13	14	15	16	17	18

1. **Gezondheidsbescherming, primaire preventie, ongerichte preventie**

- veiligheid van het drink- en oppervlaktewater
  - aanleggen van waterleiding en riolering;
  - waterzuivering;
  - controle van de kwaliteit van zwemwater (botulisme);
  - maatregelen ter voorkoming van de besmetting van leidingwater met de legionellabacterie ter voorkoming van veteranenziekte
- veiligheid van levensmiddelen
  - verbod op het gebruik bepaalde bestrijdingsmiddelen in de landbouw
  - verbod op het gebruik van kankerverwekkende stoffen bij de productie van voedingsmiddelen
  - controle van de kwaliteit van voedingsmiddelen door de Keuringsdienst van Waren;
  - verbod op de verkoop van drank aan minderjarigen
- veiligheid van de openbare ruimte
  - ongediertebestrijding
  - verbod op wapenbezit voor burgers
  - rookverbod in openbare ruimten om passief meeroken tegen te gaan
- veiligheid in huis
  - verplichting tot het aanbrengen van brandmelders in iedere woning (niet doorgevoerd)
  - bouwvoorschriften voor woningen m.b.t. het aarden van stopcontacten, verbod op het gebruik van asbest, ventilatie van de woning, enz.
- veiligheid op het werk
  - verplicht gebruik van gehoorbeschermers bij lawaaierige werkzaamheden

  - wettelijke eisen aan de inrichting van kantoorwerkplekken
  - wettelijke eisen aan werktijden en pauzes
- verkeersveiligheid
  - verplichting tot het dragen van een valhelm voor motor- en brommerrijders;
  - dragen van veiligheidsgordels in de auto
  - snelheidsbegrenzer op vrachtwagen
  - verplichting tot het aanbrengen van een kinderslot op autodeuren
  - 'botsveiligheid' van auto's
  - verplichting tot het aanbrengen van zijreflectoren op fietsen
- kindveiligheid
  - verplichting tot het dragen van een fietshelm door kinderen (in Duitsland het geval)
  - kindveilige sluitingen op flessen met schoonmaakmiddel
  - verbod op verkoop gevaarlijk speelgoed
  - "Kijkwijzer" (screening van televisieprogramma's op geweld, sex, enz.) (psychische gezondheid)
  - kindvriendelijke programmering van televisieprogramma's (geweld en sex uitsluitend laat op de avond) (psychische gezondheid)

2. **Gezondheidsbescherming, primaire preventie, hoogrisicobenadering**
- tijdelijke sluiting van een hotel of zwembad na de constatering van de aanwezigheid van de legionellabacterie in het leidingwater (opportunistische benadering)
- controle van voedingsmiddelen op voedselinfecties door de Keuringsdienst van Waren n.a.v. gezondheidsklachten (opportunistische benadering);
- organisatiebreed werkplekonderzoek vanuit de arbodienst na melding van CANS[1] van enkele werknemers (opportunistische benadering)
- ongediertebestrijding na melding van overlast (opportunistische benadering)
- software programma's voor ouderlijk toezicht op internetgebruik door kinderen (psychische gezondheid)
- bescherming van het ongeboren kind
  - invoering van een preconceptioneel consult met het oog op het staken van het gebruik van bepaalde geneesmiddelen en het aanpassen van riskante arbeidsomstandigheden (opportunistische benadering)

1. CANS staat voor Complaints of Arm, Neck and/or Shoulder (voorheen RSI).

  - leefregels voor zwangere vrouwen m.b.t. voedingsmiddelen (geen schimmelkaas, geen rauw vlees), omgang met huisdieren, (niet de kattenbak verschonen), sporten, enz. (opportunistische benadering)
  - weren van (hoog)zwangere vrouwen uit vliegtuigen, achtbanen, enz.
- bescherming van het pas geboren kind
  - leefregels voor vrouwen die borstvoeding geven m.b.t. medicijngebruik, alcoholgebruik, gebruik van voedingsmiddelen, enz.(opportunistische benadering)
- gebruik van tilliften in de gezondheidszorg (verpleging) ter voorkoming van rugklachten

3. **Gezondheidsbescherming, secundaire preventie, populatiebenadering**

- algemene aanstellingskeuring voor werknemers (in Nederland afgeschaft)
- militaire keuring voor het beroepsleger (voorheen keuring voor dienstplicht)

4. **Gezondheidsbescherming, secundaire preventie, hoogrisicobenadering**

- organisatiebreed werkplekonderzoek vanuit de arbodienst n.a.v. CANS bij enkele werknemers
- sanering van een huis na allergische klachten van een bewoner
- rijbewijskeuring voor mensen ouder dan 75 jaar

5. **Gezondheidsbescherming, tertiaire preventie, populatiebenadering**

–

6. **Gezondheidsbescherming, tertiaire preventie, hoogrisicobenadering**

- weerbericht gericht op hooikoorts (pollen) of longpatiënten (smog)
- werkplekonderzoek vanuit de arbodienst bij werknemers met CANS
- sanering van een huis na allergische klachten van een bewoner
- ergonomische aanpassingen op de werkplek

7. **Ziektepreventie, primaire preventie, populatiebenadering**

- onklaar maken van een met cholera besmette waterleiding
- sluiting van een hotel waarvan de waterleiding met de legionellabacterie besmet is ter voorkoming van veteranenziekte;
- isoleren van patiënten met een besmettelijke ziekte om besmetting van de gezonde bevolking te voorkomen;
- informeren van de bevolking over risico's van infectieziekten (rode hond, kinkhoest) voor zwangere vrouwen
- toevoeging van jodium aan het zout dat wordt gebruikt bij de bereiding van brood ter voorkoming van struma of krop;

- vaccineren van kinderen tegen kinderziekten;
- Rijksvaccinatieprogramma (RVP)
- uitvoering van een bevolkingsonderzoek op borstkanker
- verplicht stellen van vaccinatie om toegang te krijgen tot het land
- geautomatiseerd voedingsadvies op maat
- invoeren van regelgeving die het roken verbiedt
- verrijking van voedingsmiddelen om bepaalde deficiënties tegen te gaan
- het beperken van tabaksreclame
- voorlichting over veilig vrijen in kader van HIV-preventie via de massamedia
- testen van donorbloed op HIV
- periconceptioneel gebruik van foliumzuur ter voorkoming van neuralebuisdefecten
- toediening van vitamine K ter voorkoming van hemorragische ziekte van de pasgeborene
- gezondheidsvoorlichting (GVO) ter voorkoming van wiegendood
- gezondheidsvoorlichting (GVO) over SOA/HIV

8. **Ziektepreventie, primaire preventie, hoogrisicobenadering**

- voorlichtingsprogramma gericht op HIV-preventie voor homomannen die bepaalde uitgaansgelegenheden bezoeken;
- gezondheidsvoorlichting (GVO) over SOA/HIV
- vroege opsporing van voorboden van hart- en vaatziekte door opsporing van een hoge bloeddruk (risicofactor) bij patiënten met overgewicht
- gratis aanbieden van vaccinatie op plaatsen waar mensen uit risicogroepen te vinden zijn
- duurder maken van sigaretten
- plaatsing van waarschuwingen op de verpakking van sigaretten
- behandeling met antivirale middelen (bijvoorbeeld na een prikaccident in het ziekenhuis)
- isolatie van met een infectieziekte geïnfecteerde personen
- vaccineren of profylactisch behandelen van personen die in contact zijn geweest met personen met een infectieziekte (bron- en contactopsporing/ BCO)
- outbreak management vindt plaats als er sprake is van een (mogelijke) epidemische verheffing van een infectieziekte
- voorlichting over veilig vrijen in kader van HIV-preventie op scholen of ontmoetingsplaatsen voor homosexuelen
- uitdelen van condooms op meerdaagse popconcerten in kader van HIV-preventie

- ter beschikking stellen van steriele naalden aan intraveneuze drugsgebruikers ter voorkoming van HIV-besmetting
- prenataal onderzoek op een genetische afwijking
- preventieve borstamputatie voor mensen die genetisch belast zijn met een gen dat het risico op het krijgen van borstkanker sterk verhoogd
- medicamenteuze profylaxe (preventieve behandeling) met vitamine D ter voorkoming van rachitis
- vaccinatie van ouderen en doelgroepen ter voorkoming van influenza (griep)
- vaccinatie tegen reizigersziekten
- kraamzorg ter preventie van infecties, borstvoedingsproblemen en psychische klachten

9. **Ziektepreventie, secundaire preventie, populatiebenadering**
- bevolkingsonderzoek (screening en screeningstest): actief zoeken in grote groepen van de bevolking naar aandoeningen, voorstadia of risicofactoren, zodat vroege behandeling mogelijk is
- screenen op kanker, syfilis (tijdens prenatale zorg), gehoorafwijkingen (in de jeugdgezondheidszorg) en tandbederf (bij periodieke gebitscontrole door de tandarts)
- screenen op mogelijke voorboden van kanker, zoals darmpoliepen bij dikkedarmkanker en epitheelafwijkingen bij baarmoederhalskanker
- screening op genetische predispositie voor kanker
- prenatale screening en adviezen ten aanzien van zwangerschapshypertensie en zwangerschapsdiabetes
- lichamelijk onderzoek naar niet-scrotale testes in de jeugdgezondheidszorg
- lichamelijk onderzoek naar congenitale (aangeboren) afwijkingen, zoals hartafwijkingen, heupluxatie en heupdysplasie
- vroege opsporing van stofwisselingsziekten via de hielprik bij pasgeborenen
- lichamelijk onderzoek naar visusafwijkingen bij kinderen
- vroege opsporing van ontwikkelingsstoornissen (waaronder spraak- en taalstoornissen)

10. **Ziektepreventie, secundaire preventie, hoogrisicobenadering**
- bevolkingsonderzoek (screening en screeningstest): actief zoeken in specifieke groepen van de bevolking naar aandoeningen, voorstadia of risicofactoren, zodat vroege behandeling mogelijk is
- toedienen van een griepvaccin aan mensen met een hart- of longaandoening
- voorschrijven van bloeddrukverlagers aan patiënten die een hartinfarct hebben gehad;
- Rijksvaccinatieprogramma (RVP)

- vroege opsporing van kanker bij mensen met verdachte symptomen, zoals onverklaard gewichtsverlies, bloed in de ontlasting, pijn of een recent gevoeld knobbeltje, enz.
- uitvoeriger diagnostiek die wordt aangeboden aan mensen die uit de (bevolkings)screening naar voren komen als mensen met een verhoogd risico op de betreffende aandoening(en)
- screening op genetische predispositie voor kanker
- voorkomen van ongewenste vormen van zwangerschap door verschillende vormen van anticonceptie
- screening op SOA/HIV

11. **Ziektepreventie, tertiaire preventie, populatiebenadering**

–

12. **Ziektepreventie, tertiaire preventie, hoogrisicobenadering**

- dieetadvies aan een diabetespatiënt

13. **Gezondheidsbevordering, primaire preventie, populatiebenadering**

- voorlichting om stoppen met roken te bevorderen;
- landelijke campagne gericht op het stimuleren van een voeding met minder vet en meer groente en fruit;
- gezondheidsvoorlichting en –opvoeding (GVO) wordt gedefinieerd als alle combinaties van leerervaringen die bedoeld zijn om gezond gedrag op vrijwillige basis te stimuleren. Het gaat hierbij ook om het oefenen van praktische vaardigheden om bijvoorbeeld condoomgebruik ter sprake te brengen met een (nieuwe) sexpartner, of om kinderen te stimuleren om groente te eten.
- gezondheidsvoorlichting (GVO) ter voorkoming bij kinderen van voedingsproblemen, mishandeling, ongelukken
- gezondheidsvoorlichting (GVO) over hygiëne ter voorkoming van infectieziekten

14. **Gezondheidsbevordering, primaire preventie, hoogrisicobenadering**

- leefstijladvies aan patiënt met beginnend overgewicht
- stoppen-met- rokenprogramma's
- gezondheidsvoorlichting en –opvoeding (GVO) wordt gedefinieerd als alle combinaties van leerervaringen die bedoeld zijn om gezond gedrag op vrijwillige basis te stimuleren. Het gaat hierbij ook om het oefenen van praktische vaardigheden om bijvoorbeeld nee te zeggen als een sigaret wordt aangeboden.
- minimale-interventiestrategie (MIS) in de huisartsenpraktijk voor stoppen met roken

- gezondheidsvoorlichting (GVO) bij voedingsproblemen, kindermishandeling
- gezondheidsvoorlichting (GVO) ter voorkoming van reizigersziekten
- algemene voorlichting over medicijngebruik gericht op ouderen
- sexuele voorlichting aan allochtone jongeren
- invoering van een preconceptioneel consult ter bevordering van het stoppen met roken en gebruik van alcohol
- leefstijladviezen aan mensen met een hoge bloeddruk

15. **Gezondheidsbevordering, secundaire preventie, populatiebenadering**
- leefstijladviezen aan alle mensen die voor een bepaalde aandoening onder behandeling zijn: HIV, kanker, CANS (wellicht leven de meeste van hen al gezond)
- algemene voorlichting over medicijngebruik aan chronische gebruikers

16. **Gezondheidsbevordering, secundaire preventie, hoogrisicobenadering**
- leefstijladviezen op indicatie aan mensen die voor een bepaalde aandoening onder behandeling zijn: HIV, kanker, CANS
- leefstijladvies aan alle mensen die na een operatie uit het ziekenhuis worden ontslagen
- voorlichting over wondbehandeling
- voorlichting over omgaan met hyperventilatie

17. **Gezondheidsbevordering, tertiaire preventie, populatiebenadering**

–

18. **Gezondheidsbevordering, tertiaire preventie, hoogrisicobenadering**
- revalidatie na een operatie

Bijlage 3: Indeling van preventiemaatregelen naar doelgroep

Momenteel is in Nederland in het kader van de discussie over mogelijke vergoeding van bepaalde preventiemaatregelen via de basiszorgverzekeringen een nieuwe indeling naar 'doelgroep van de preventie' gangbaar:

- collectieve preventie:
  a. universele preventie (populatiebenadering);
  b. selectieve preventie (hoogrisicobenadering);
- individuele preventie:
  c. geïndiceerde preventie;
  d. zorggerichte preventie.

Deze nieuwe indeling leidt echter niet tot andere conclusies m.b.t. de onbruikbaarheid van de categorisering van preventiemaatregelen voor beantwoording van de onderzoeksvragen '*, wie kan, wat mag, wie mag, wat moet, wie moet*'. In geval van 'individuele preventie' is de hoofdregel dat er sprake dient te zijn van 'informed consent' van de patiënt. In geval van collectieve preventie worden de nieuwe benamingen 'universele preventie' en 'selectieve preventie' gegeven aan respectievelijk de bestaande begrippen 'populatiebenadering' en 'hoogrisicobenadering'.

Toelichting van begrippen

Zie: Bos, 2007.

Collectieve preventie omvat universele en selectieve preventie:

- ***Universele preventie (3a)*** is gericht op de algemene bevolking. Het gaat hierbij bijvoorbeeld om publiekscampagnes ter bestrijding van roken of fysieke inactiviteit. Maar ook gaat het hierbij om het realiseren van een goede toegankelijkheid van de samenleving en gelijke rechten voor mensen met een chronische aandoening of handicap.
- ***Selectieve preventie (3b)*** is gericht op hoogrisicogroepen. Zo is in 2006 de voorlichtingscampagne 'Kijk op Diabetes' van start gegaan. Deze campagne beoogt de versnelde opsporing van mensen met nog onbekende diabetes en van mensen die een verhoogd risico hebben om binnen afzienbare tijd diabetes type 2 te krijgen. Speciale aandacht gaat daarbij uit naar allochtonen,

met name Turken, Marokkanen en Hindoestanen, en naar mensen met een lage sociaal-economische status.

Individuele preventie omvat geïndiceerde en zorggerelateerde preventie:

- ***Geïndiceerde preventie (3c)*** is gericht op individuen met een bekend verhoogd gezondheidsrisico. Zo worden huisbezoeken afgelegd bij ouderen met een verhoogd risico op valongevallen, zoals ouderen die slaap- en kalmeringsmiddelen gebruiken.
- ***Zorggerichte preventie (3d)*** is gericht op mensen die een chronische aandoening hebben en die in het individuele hulpverleningscontact preventie krijgen aangeboden, bijvoorbeeld een verwijzing naar een stop-met-roken-training door de huisarts of een verwijzing naar een patiëntenvereniging.

Bijlage 4: Criteria van andere beoordelingsmodellen

Criteria van Dunning

De achtereenvolgende criteria waaruit de Trechter van Dunning is opgebouwd, zijn (Dunning, 1991):

1. *Noodzakelijkheid*
2. *Werkzaamheid*
3. *Doelmatigheid*
4. *Eigen rekening en verantwoording.*

Criteria van het CVZ

Het CVZ is de autoriteit op het gebied van de samenstelling van het basispakket van de Zorgverzekeringswet en AWBZ in Nederland. Het CVZ geeft aan de volgende pakketcriteria te hanteren (Mastenbroek, 2006, p.29):

1. *Noodzakelijkheid:*
   Rechtvaardigt de ziekte of benodigde zorg een claim op solidariteit, gegeven de culturele context?
2. *Effectiviteit:*
   Doet de interventie of zorgvorm wat er in de breedste zin van wordt verwacht?
3. *Kosteneffectiviteit (p.38: kostenutiliteit/ -effectiviteit):*
   Is de verhouding tussen de kosten en de baten in de breedste zin acceptabel?
4. *Uitvoerbaarheid:*
   Is het nu en later haalbaar en houdbaar om de interventie of zorgvorm op te nemen in het pakket?

Criteria van Wilson en Jungner

De criteria van Wilson en Jungner betreffen beslissingen over de aanvaardbaarheid van screening van de bevolking op aandoeningen (Wilson, 1968) (Gezondheidsraad, 1994, p.120):

1. *The condition sought should be an important health problem.*

2. *There should be an accepted treatment for patients with recognised disease.*
3. *Facilities for diagnosis and treatment should be available.*
4. *There should be a recognisable latent or early symptomatic stage.*
5. *There should be a suitable test or examination.*
6. *The test should be acceptable to the population.*
7. *The natural history of the condition, including development from latent to declared disease, should be adequately understood.*
8. *There should be an agreed policy on whom to treat as patients.*
9. *The cost of case-finding (including diagnosis and treatment of patients diagnosed) should be economically balanced in relation to possible expenditure on medical care as a whole.*
10. *Case-finding should be a continuing process and not a 'once and for all' project.*

In het leerboek Volksgezondheid en gezondheidszorg zijn de criteria van Wilson en Jungner als volgt in het Nederlands vertaald (Brug, 2004, p.111):

1. *In welke mate vormt de ziekte een volksgezondheidsprobleem?*
2. *Is er een algemeen aanvaarde behandelwijze van de ziekte?*
3. *Zijn er voldoende voorzieningen voor diagnose en behandeling van de ziekte beschikbaar?*
4. *Is er een herkenbaar latent stadium van de ziekte?*
5. *Wat zijn de eigenschappen van de voorgestelde test of onderzoeksmethode voor het bevolkingsonderzoek?*
6. *Hoe aanvaardbaar is de test voor de bevolking?*
7. *Wat is er bekend over het natuurlijk beloop van de ziekte?*
8. *Wat is het beleid ten aanzien wie er als patiënt behandeld moet worden?*
9. *Hoe zijn de kosten van bevolkingsonderzoek (inclusief behandeling van ontdekte patiënten) in verhouding tot de beschikbare middelen voor volksgezondheid?*
10. *Is het bevolkingsonderzoek georganiseerd als continu proces, en niet als een eenmalig project?*

Onderstaand geef ik de criteria van Wilson en Jungner, t.b.v. de vergelijking met de andere modellen, in steekwoorden weer. Ik maak hierbij gebruik van de oorspronkelijke Engelse tekst (Gezondheidsraad, 1994, p.120) en de bovenstaande Nederlandse vertaling (Brug, 2004, p.111).

Criteria van Wilson en Jungner in steekwoorden:

1. *Belangrijk gezondheidsprobleem*
2. *Algemeen aanvaarde behandelwijze*
3. *Beschikbaarheid van voorzieningen voor diagnose en behandeling*
4. *Herkenbaar latent stadium*

5. *Passende test of onderzoeksmethode*
6. *Aanvaardbaarheid voor bevolking*
7. *Inzicht in beloop van de ziekte*
8. *Algemeen aanvaard beleid over wie als patiënt wordt behandeld*
9. *Verhouding kosten bevolkingsonderzoek versus beschikbare middelen voor volksgezondheid*
10. *Continuïteit van bevolkingsonderzoek*

Criteria van de Gezondheidsraad (1994)

De Gezondheidsraad heeft criteria geformuleerd waaraan een programma voor genetische screening moet voldoen. De Gezondheidsraad heeft zich bij het opstellen van deze criteria mede laten leiden door de criteria van Wilson en Jungner. De criteria van de Gezondheidsraad zijn (Gezondheidsraad, 1994, p.101):

1. *Een genetisch-screeningsprogramma moet een gezondheidsprobleem betreffen of een conditie die daartoe kan leiden bij de onderzochte of diens nageslacht.*
2. *De doelgroep van het screeningsprogramma moet duidelijk zijn omschreven.*
3. *Het doel van het programma moet zijn de deelnemers in staat te stellen kennis te nemen van de aanwezigheid van danwel het risico op een aandoening of dragerschap, en naar aanleiding daarvan een beslissing te nemen. Informed consent*
4. *Er moeten voor de deelnemers zinvolle handelingsopties zijn.*
5. *Deelname aan een genetisch-screeningsprogramma moet geheel vrijwillig zijn en plaatsvinden op basis van toestemming waaraan goede informatie ten grondslag ligt.*
6. *Er moet goede en begrijpelijke voorlichting voor de doelgroep zijn.*
7. *Er moet een voor het doel van de screening geschikte testmethode zijn.*
8. *Er moeten voldoende faciliteiten voor vervolgonderzoek, voor het uitvoeren van de gekozen handelingsopties en voor voorlichting en ondersteuning van de deelnemers zijn.*
9. *Er moeten afdoende maatregelen zijn genomen om bij de omgang met en de opslag van de medische gegevens en het celmateriaal de persoonlijke levenssfeer van de deelnemers te beschermen en hun rechten ten aanzien van hun persoonsgegevens en celmateriaal te respecteren.*
10. *Als in het kader van screening wetenschappelijk onderzoek plaatsvindt, moeten de deelnemers ook ten aanzien van dit aspect vooraf goed zijn geïnformeerd.*
11. *Er dient te zijn voorzien in een voortdurende kwaliteitsbewaking van de werkzaamheid, doelmatigheid en veiligheid van de testprocedure en eventuele vervolgacties, en van de voorlichting aan en ondersteuning van de deelnemers.*

12. *Bij weging van de voor- en nadelen voor de deelnemers aan het programma moet de balans duidelijk naar de zijde van de voordelen uitslaan. Ten behoeve van deze weging moet degene die een screeningsprogramma voorstelt informatie leveren over:*
    a. *de prevalentie van de ziekte of aandoening in de doelgroep;*
    b. *het natuurlijk beloop en de variatie in de ernst van de aandoening;*
    c. *de doelgroepen die voor het onderzoek in aanmerking komen en de overwegingen die hebben geleid tot de keuze van de voorgestelde doelgroep en het voorgestelde tijdstip in het leven om de test uit te voeren;*
    d. *de specificiteit, sensitiviteit en voorspellende waarde van de te gebruiken testmethode en de belasting die het onderzoek voor de deelnemers zal meebrengen;*
    e. *de handelingsopties indien een gezondheidsprobleem of dragerschap is aangetoond;*
    f. *de tijd die de procedure laat voor het overwegen en eventueel uitvoeren van de gekozen handelingsoptie;*
    g. *de mogelijke, zowel gunstige als ongunstige, psychologische, maatschappelijke en andere gevolgen van aanbod en eventuele deelname of niet-deelname aan de screening voor de te onderzoeken persoon en diens familieleden of voor groepen in de samenleving;*
    h. *de kans op foutieve uitslagen, de mogelijke gevolgen daarvan voor de deelnemers en de maatregelen die zijn genomen om eventuele schade ten gevolge daarvan te beperken;*
    i. *de waarborgen die er zijn om te voorkómen dat deelnemers ongerechtvaardigde belemmeringen ondervinden van hun deelname of niet-deelname aan het screeningsprogramma of vervolgonderzoek bij de toegang tot arbeid of particuliere verzekering;*
    j. *de kosten die verbonden zijn aan de screening en aan het realiseren van de vereiste infrastructuur.*

Onderstaand geef ik de criteria, waaraan een programma voor genetische screening volgens de Gezondheidsraad moet voldoen, t.b.v. de vergelijking met de andere modellen, in steekwoorden weer.

Criteria van de Gezondheidsraad in steekwoorden:
1. *Gezondheidsprobleem*
2. *Doelgroep is duidelijk*
3. *Doel is deelnemers faciliteren*
4. *Zinvolle handelingsopties na testuitslag*
5. *Informed consent*
6. *Begrijpelijke voorlichting*

7. *Geschikte testmethode*
8. *Faciliteiten voor vervolgacties*
9. *Bescherming privacy*
10. *Informatie over nevendoelstellingen (wetenschappelijk onderzoek)*
11. *Voortdurende kwaliteitsbewaking*
12. *Meer voordelen dan nadelen voor deelnemers. Weging op basis uitgebreide informatie over:*
    a. *prevalentie;*
    b. *natuurlijk beloop en variatie in ernst van aandoening;*
    c. *waarom van gekozen doelgroepen en tijdstip in leven van test;*
    d. *kwaliteit van testmethode en belasting voor deelnemers;*
    e. *handelingsopties na test;*
    f. *tijd voor overwegen (be)handelingsopties;*
    g. *gunstige en ongunstige, psychologische, maatschappelijke en andere gevolgen van (niet-) deelname;*
    h. *kans op schade ten gevolge van (test)fouten;*
    i. *voorkómen van ongerechtvaardigde gevolgen;*
    j. *kosten van uitvoering (screening en infrastructuur).*

Bijlage 5: Definiëring van het begrip 'schade' ten behoeve van het beoordelingsmodel

In de wetenschappelijke literatuur bestaat ***geen consensus*** over de definitie van het begrip 'schade'. Het wordt door sommigen smal (schade aan materiële belangen, schade aan gezondheidsbelangen, inbreuk op de persoonlijke levenssfeer) en door anderen breed gedefinieerd (schade aan materiële belangen, aan gezondheidsbelangen, schending van mensenrechten, schade aan emotioneel welzijn, aan zelfontplooiing, etc.) (Pope, 2005). Onderstaand tracht ik op basis van de wetenschappelijke literatuur tot een eigen definiëring en operationalisering van het begrip 'schade' te komen ten behoeve van het model. Hierbij baseer ik me, evenals veel andere auteurs, op het standaardwerk 'Harm to others. The moral limits of the criminal law' van Joel Feinberg (1984).

Feinberg definieert schade als 'setback to an interest' (Feinberg, 1984, p.53), (Ripstein, 2006, p218):

> *"To setback an interest is to reverse its course, turn it away, put it back toward the point from which it started. In terms of its associated goals, it is to reverse its progress, to put it in an worse condition than it was formerly in".*

Belangrijk is om op te merken dat het hinderen of frustreren van derden volgens deze definitie nog geen schade genoemd mag worden. Feinberg maakt onderscheid tussen belangen (interests) die bepalend zijn voor iemands welzijn (welfare interests) en overige belangen.

Binnen de 'overige belangen' onderscheidt Feinberg:
- voorbijgaande wensen (bv. zin in een biertje);
- instrumentele wensen (bv. ik wil vroeg naar bed);
- hogere doelen (bv. ik wil een boek schrijven).

Belangen die bepalend zijn voor iemands welzijn zijn ***basisbelangen*** [1]. Basisbelangen (zoals gezondheid en inkomen) hebben als kenmerk dat bijna iedereen ze voor zichzelf en anderen als belang erkend, dat ze geen specifiek persoonlijk doel verte-

1. De term 'basisbelangen' wordt gebruikt door Maris, als hij de 'welfare interests' van Feinberg bespreekt (Maris, 2006, p.94).

genwoordigen, dat ze voorwaardenscheppend zijn voor het realiseren van specifieke persoonlijke doelen (wensen en hogere doelen) en dat ze niet van voorbijgaande aard zijn. Dit in tegenstelling tot ***'overige belangen'***. (Feinberg, 1984, p.37-38 en 60)

In de praktijk kunnen alle verschillende belangen van mensen (basisbelangen, voorbijgaande wensen, instrumentele wensen, en hogere doelen) worden aangetast door het gedrag van derden of door eigen gedrag. Feinberg werpt de vraag op tegen aantasting van welke belangen het strafrecht bescherming dient te bieden. Volgens hem ***redeneert de wetgever altijd terug naar 'schade aan basisbelangen'***. De reden is dat met de wettelijke bescherming van deze belangen ook alle andere belangen tot op zekere hoogte worden beschermd. (Feinberg, 1984, p.61-64)

Ter illustratie een voorbeeld. Als iemand een boek wil schrijven, maar hij heeft geen huisvesting en geen pen en papier, dan ontbreekt het hem aan basisbelangen (huisvesting en inkomen). De wet dient iemand te beschermen tegen het ontbreken van deze basisbelangen. Als het iemand niet lukt om een boek te schrijven, omdat het hem ontbreekt aan inspiratie, hoeft de wet daartegen geen bescherming te bieden. Basisbelangen vormen de minimumvoorwaarden voor mensen om zich te kunnen handhaven en de minimumvoorwaarden om persoonlijke doelen te kunnen realiseren. Basisbelangen zijn voor bijna alle mensen gelijk, waardoor het mogelijk is ze in de wet te beschrijven. Volgens Feinberg vormt de aantasting van basisbelangen de ernstigste schade die iemand kan oplopen. Als niet meer wordt voldaan aan de basisbelangen ontbreken namelijk ook de voorwaarden voor het realiseren van persoonlijke doelen (zoals een boek schrijven). Andersom is dat niet het geval. (Feinberg, 1984, p.37-38).

Vanwege de volgende kenmerken van basisbelangen, acht ik ze geschikt om als uitgangspunt te nemen voor definiëren en operationaliseren van het begrip 'schade' ten behoeve van het model:
- aantasting van basisbelangen vormt schade in de zin van het schadebeginsel;
- aantasting van andere relevante belangen is bijna altijd terug te redeneren naar aantasting van een of meer basisbelangen;
- basisbelangen gelden nagenoeg voor iedereen en worden breed aanvaard.

Ten slotte aanvaardt Feinberg ook aantasting van bepaalde publieke belangen (public interests) (bijvoorbeeld een gezonde economie, of bescherming tegen vijandelijke mogendheden) en bepaalde overheidsbelangen (governmental interests) (bijvoorbeeld innen van belastingen, of registreren van vreemdelingen) als schade in de zin van het schadebeginsel, omdat de aantasting van deze belangen ten koste

gaat van de basisbelangen van het grootste deel van de bevolking. (Feinberg, 1984, p.63).

Feinberg geeft de volgende opsomming van basisbelangen (Feinberg, 1984, p.37 en 60):

- physical health and vigor (lichamelijke gezondheid en kracht);
- integrity and normal functioning of one's body (lichamelijke integriteit en normaal functionering van het lichaam);
- absence of absorbing pain and suffering or grotesque disfigurement (afwezigheid van ernstige pijn en ernstig lijden of groteske misvorming);
- minimal intellectual acuity (tenminste minimale intellectuele capaciteiten);
- emotional stability (emotionele stabiliteit c.q. psychische gezondheid);
- the absence of groundless anxieties and resentments (afwezigheid van bodemloze angst en haat; te vertalen met niet hoeven leven in angst en vrees);
- capacity to engage normally in social intercourse and enjoy and maintain friendships (vermogen tot normale sociale interactie en te genieten van vriendschappen en deze te onderhouden);
- at least minimal income and financial security (tenminste een basaal inkomen en basale financiële zekerheid);
- tolerable social and physical environment (leefbare sociale en fysieke omgeving);
- certain amount of freedom from interference and coercion (zekere mate van vrijheid van bemoeizucht en dwang).

Met behulp van deze opsomming tracht ik het begrip 'schade' ten behoeve van het beoordelingsmodel te definiëren. Ten behoeve van de uitvoerbaarheid van de beoordeling tracht ik door het clusteren van basisbelangen het ***aantal schadecategorieën te beperken***. De laatste decennia pleiten diverse auteurs er voor om het begrip 'schade' niet te breed te definiëren (Dyzenhaus, 1992)[2], want dan is het schadebeginsel niet meer onderscheidend (Feinberg, 1984, p.245) (Pope, 2005, p.166-179). Daarmee ben ik het eens.

Incidentele aantastingen van het emotionele welzijn aanvaard ik niet als schade. Op dit punt volg ik Mill (1859), die heeft gesteld dat ergernis, woede of morele verontwaardiging over het gedrag van een individu geen rechtvaardigingsgrond

2. Het artikel van Dyzenhaus 'John Stuart Mill and the harm of pornography' vormt een voorbeeld van een brede opvatting van het schadebeginsel.

vormen voor overheidsingrijpen en op zichzelf geen schade vertegenwoordigen. Pas als er sprake is van een structurele verstoring van het emotionele welzijn (Feinberg spreekt over het basisbelang van emotionele stabiliteit) definieer ik dat als schade en wel aan de psychische gezondheid.

In navolging van Mill, Feinberg, Maris en vele anderen aanvaard ik een schending van morele opvattingen (opvattingen over goed en kwaad; over wenselijk en onwenselijk gedrag) op zichzelf ook niet als schade. Een schending van een morele opvatting (bijvoorbeeld de opvatting dat alcoholgebruik slecht is) kan leiden tot schade, maar vormt op zichzelf geen schade. Pas als de schending van de morele opvatting leidt tot schade aan basisbelangen, is er sprake van schade in de zin van het schadebeginsel. Voor zover men vanwege de risico's op schade aan basisbelangen bezwaar maakt tegen de schending van de morele opvatting, dient men in het kader van het criterium 'schadelijkheid' van het model deze risico's op schade te beschrijven. Voor zover risico's op schade aan basisbelangen niet de reden vormen dat men bezwaar maakt tegen de schending van de morele opvatting, dient met in het kader van het criterium 'schadelijkheid' van het model te beschrijven, wat dan de beweegredenen van het bezwaar zijn. Bijvoorbeeld: dronkenschap is walgelijk, mijn geloof verbiedt alcoholgebruik, enz. In dat geval dient de schending van de morele opvatting in het kader van het criterium 'schadelijkheid' van het model als schending van morele opvattingen te worden geclassificeerd.

Ten slotte aanvaard ik overlast niet als schade. Overlast kan leiden tot schade, maar vormt op zichzelf geen schade. Ook in geval van overlast is het m.i. mogelijk en wenselijk om, zoals Feinberg voorstelt, terug te redeneren naar schade aan basisbelangen. Als de overlast leidt tot schade aan basisbelangen, is er sprake van schade in de zin van het schadebeginsel. In dat geval dient in het kader van het criterium 'schadelijkheid' van het model de schade aan basisbelangen ten gevolge van de overlast te worden beschreven. Voor zover vanuit de overlast niet naar schade aan basisbelangen kan worden teruggeredeneerd, dient de overlast in het kader van het model als overlast te worden geclassificeerd.

In aanvulling (er is sprake van overlap) op de opsomming van basisbelangen door Feinberg (zie enkele alinea's hierboven) gebruik ik de ***Universele Verklaring van de Rechten van de Mens*** (UVRM) als algemeen aanvaarde (en redelijk uitputtende) opsomming van basisbelangen. Schending van de rechten die zijn vastgelegd in de UVRM wordt algemeen als schade aanvaard.

De UVRM beschrijft ondermeer het recht op (VN, 1948):

- vrijheid, gelijkheid, waardigheid, broederlijke (respectvolle) bejegening (artikel 1);
- gelijke behandeling (artikel 2);
- leven, vrijheid en lichamelijke integriteit (artikel 3);
- privacy (artikel 12);
- behoud van zijn eer en goede naam (artikel 12);
- vrije verplaatsing binnen de landsgrenzen (artikel 13);
- eigendom (artikel 17);
- vrijheid van meningsuiting (artikel 19);
- maatschappelijke zekerheid (artikel 22);
- voorwaarden voor vrije ontplooiing van zijn persoonlijkheid (artikel 22);
- arbeid (artikel 23);
- rust en vrije tijd (artikel 24);
- voorwaarden voor gezondheid en welzijn (artikel 25);
- onderwijs (artikel 26).

Ten behoeve van het beoordelingsmodel definieer ik 'schade' als schade aan basis-belangen' en onderscheid ik drie schadecategorieën:

1. ***Schade aan gezondheidsbelangen (gezondheidsschade);***
   Gezondheidsschade definieer ik ten behoeve van het model als aantasting van de lichamelijke of psychische gezondheid.
2. ***Schade aan sociaal-economische belangen (sociaal-economische schade);***
   Aantasting van financieel-economische belangen (bezit, inkomen, winst, concurrentiepositie, e.d.) wordt algemeen en dus ook door mij als schade aanvaard. In aanvulling hierop en overeenkomstig de opsomming van basisbelangen door Feinberg, aanvaard ik aantasting van sociaal-maatschappelijke belangen (sociale contacten, maatschappelijke participatie, huisvesting, e.d.) en sociaal-economische belangen (huisvesting, bezit, opleiding, inkomen, arbeidsgeschiktheid, werk, e.d.) ook als schade. Sociale vereenzaming, dakloosheid, (gedwongen) ontslag, (moeten) afbreken van een opleiding, e.d. ten gevolge van een bepaalde leefstijl (bijvoorbeeld alcoholmisbruik, drugsgebruik, e.d.) worden algemeen als ontwrichtend voor het 'normale' c.q. basale functioneren van mensen beschouwd. Vanwege de overlap tussen de begrippen 'sociaal-maatschappelijke belangen, sociaal-economische belangen en financieel-economische-belangen', voeg ik deze belangen ten behoeve van het beoordelingsmodel samen tot één schadecategorie: sociaal-economische schade.
3. ***Schending van individuele mensenrechten***
   Schending van individuele mensenrechten definieer ik ten behoeve van het model – op basis van de mensenrechten, zoals beschreven in de UVRM – als een aantasting van de:

– lichamelijke integriteit;
– vrijheid;
– privacy;
– gelijke behandeling;
– eer en goede naam, waardigheid, of respectvolle bejegening.

In §7.2.1 – over het criterium ‘indringendheid’ – werk ik de betekenis van deze begrippen nader uit.

Het onderscheid tussen enerzijds ‘gezondheidsschade en sociaal-economische schade’ en anderzijds ‘schending van individuele mensenrechten’ refereert aan het onderscheid tussen respectievelijk de sociale en klassieke mensenrechten. Ik zal dit toelichten aan de hand van een citaat van een webpagina van de rijksoverheid over grond- en mensenrechten[3]:

“Grondrechten (ook wel mensenrechten genoemd) zijn onderverdeeld in:
- *Burgerlijke en politieke rechten (klassieke grond- of mensenrechten) zoals kiesrechten, diplomatieke bescherming, vrijheid van meningsuiting, recht op privacy, godsdienstvrijheid en discriminatieverbod.*
- *Sociale, economische en culturele rechten zoals recht op huisvesting, sociale zekerheid, gezondheidszorg en onderwijs.*

Klassieke versus sociale grondrechten
Vaak wordt een onderscheid gemaakt tussen klassieke en sociale grondrechten. Bij klassieke grondrechten ligt de nadruk op terreinen waar de overheid zich niet mee mag bemoeien. Terwijl bij bescherming van sociale grondrechten de nadruk meer ligt op bevordering door middel van beleid. De meeste sociale grondrechten zijn op dit moment nog niet afdwingbaar via de rechter. Burgers kunnen klassieke grondrechten wel afdwingen. …”

De drie schadecategorieën die ik heb onderscheiden, vertonen een enige overlap, maar zijn m.i. voldoende onderscheidend om binnen het model goed mee te kunnen werken. Vooral het onderscheid tussen de categorie ‘gezondheidsschade’ en de subcategorie ‘lichamelijke integriteit’ vraagt om een toelichting. Bij de categorie ‘gezondheidsschade’ ligt de nadruk op de ernst van de gezondheidsschade. Bij de subcategorie ‘aantasting van de lichamelijke integriteit’ ligt de nadruk op de ernst van de inbreuk op de persoonlijke levenssfeer. Denk bijvoorbeeld aan een verkrachting, die een zeer ernstige inbreuk vormt op de persoonlijke levenssfeer, maar niet tot ernstige gezondheidsschade hoeft te leiden.

3. http://www.rijksoverheid.nl/onderwerpen/grondwet-en-statuut/grondwet/grondrechten

Een aantasting van (1) triviale, (2) kwaadaardige, of (3) moralistische belangen en een (4) eerlijke competitie om schaarse goederen vormen volgens Feinberg (1984) ***geen schade*** in de zin van het schadebeginsel (Maris, 2007, p.94). Deze inperking van het schadebegrip neem ik over. Schade aan derden vormt volgens het schadebeginsel een rechtvaardiging voor een aantasting van basisbelangen. Een aantasting van (1) triviale belangen van derden kan logischerwijs geen rechtvaardiging vormen voor een aantasting van basisbelangen. Een aantasting van triviale belangen vormt daarom geen schade in de zin van het schadebeginsel. (2) Kwaadaardige belangen (bv. de wens om iemand te vermoorden) zijn niet te billijken en kunnen daarom niet als basisbelang worden aanvaard. Schade heb ik gedefinieerd als de aantasting van basisbelangen. Een aantasting van kwaadaardig belangen (bv. het voorkomen van een moord) mag daarom niet als schade (aan een basisbelang van de potentiële moordenaar) worden opgevat. Een schending van (3) morele opvattingen van derden vormt volgens het schadebeginsel geen rechtvaardigingsgrond voor overheidsingrijpen. Daar het toebrengen van schade aan derden volgens het schadebeginsel wel een rechtvaardigingsgrond vormt voor overheidsingrijpen, vormt een schending van 'morele opvattingen' in de zin van het schadebeginsel geen schade. In het kader van een (4) eerlijke competitie om schaarse goederen kan een individu de belangen van derden (andere marktpartijen) schaden. Deze aantasting van de belangen van derden is volgens Feinberg wel aanvaardbaar. De aantasting van haar belang die een marktpartij ondervindt in het kader van de eerlijke competitie om schaarse goederen, vormt daarom geen schade in de zin van het schadebeginsel.

Geredeneerd volgens het schadebeginsel, zoals geformuleerd door Mill, vormt schade aan derden wel een rechtvaardigingsgrond voor het treffen van vrijheidsbeperkende preventiemaatregelen, maar vormt een schending van morele opvattingen van derden dit niet. In het kader van het model zie ik als bezwaar tegen dit standpunt, dat dit is geënt op een liberale visie, terwijl ik er naar streef om het model ideologisch neutraal te maken. Binnen het model is ruimte voor het maken van ideologische afwegingen, maar het model zelf is ideologisch zo neutraal mogelijk. Vergelijk dit bijvoorbeeld met de Stemwijzer (www.stemwijzer.nl), die behulpzaam is bij het bepalen van politieke voorkeuren, maar die als instrument politiek zo neutraal mogelijk is (zie §15.1.2) Daarom wil ik de ***schending van morele opvattingen*** in het kader van het model ***niet uitsluiten als rechtvaardigingsgrond*** voor het treffen van vrijheidsbeperkende preventiemaatregelen. Daarbij ga ik er vanuit dat risico's op schade door een democratisch gekozen Westerse regering in het algemeen veel zwaarder worden gewogen dan de schending van morele opvattingen. Echter, in iedere samenleving zijn ook andersdenkenden en daarom kunnen

morele opvattingen niet als rechtvaardigingsgrond voor vrijheidsbeperkingen worden uitgesloten[4].

4. Mijn streven is om een algemeen toepasbaar beoordelingsmodel te ontwikkelen. Dit betekent dat het model bijvoorbeeld ook binnen een Islamitisch land, dat alcoholgebruik moreel verwerpelijk acht, bruikbaar moet zijn.

Bijlage 6: Voorbeelden van tegengestelde denkkaders (a versus b)

Eerste voorbeeld van tegengestelde morele denkkaders

a. Overgewicht is primair de schuld van het individu

De primaire oorzaak van overgewicht wordt gevormd door een gebrek aan wilskracht van het individu (Kersh, 2005, p.846-848), (Fuemmeler, 2007, p.47). Overgewicht is daarom primair de schuld van het individu (Oliver, 2005, p.925), (McMenamin, 2006, p.517-518). Partijen die primair het individu verantwoordelijk (4e criterium in het model) houden voor het ontstaan (3e criterium) van overgewicht – volgens Schwartz geldt dat voor de Amerikaanse overheid en voedingsmiddelenindustrie (Schwartz, 2007) – kiezen primair voor interventies gericht (5e criterium) op het individu, zoals voorlichting en dieetbegeleiding. Wetgeving ter regulering van de 'obesogene omgeving' [1, 2] past niet in hun denkkader en wordt niet in overweging genomen of verworpen.

b. Overgewicht is primair de schuld van de voedingsmiddelenindustrie

De primaire oorzaak van overgewicht wordt gevormd door de obesogene omgeving (Brownell, 2005, p.960), (Kersh, 2005, p.848-849), (Guerrieri, 2005), (Alderman, 2007, p.102-103), (Schwartz, 2007, p.79-81), (Lake, 2006), (Cummins, 2005), die is gecreëerd en in stand wordt gehouden door de voedingsmiddelenindustrie. De epidemie van overgewicht waarmee Westerse samenlevingen worden geconfronteerd is primair de schuld van de voedingsmiddelenindustrie. Partijen die primair de obesogene omgeving verantwoordelijk houden voor het ontstaan van overgewicht (diverse wetenschappers in de public health) pleiten primair voor

---

1. Het woord 'obesogene' is een samenstelling van de woorden 'obesitas' (ernstig overgewicht) en 'genese' (ontstaan, wording). Een obesogene omgeving is een omgeving die in belangrijke mate bijdraagt aan het ontstaan van overgewicht. Enerzijds door mensen de gelegenheid te bieden (veel verkooppunten) of te stimuleren (grote porties, reclame) om teveel of ongezonde voedingsmiddelen te gebruiken. Anderzijds door mensen onvoldoende gelegenheid te bieden om te bewegen of door beweging te ontmoedigen. Dagevos en Munnichs spreken zelfs over 'De obesogene samenleving' (Dagevos, 2007).
2. The obesogenicity of an environment has been defined by Swinburn et al. (2002) as 'the sum of influences that the surroundings, opportunities, or conditions of live have on promoting obesity in individuals or populations.' (Lake, 2007, p.262).

interventies gericht (5e criterium) op de omgeving, zoals bijvoorbeeld een verbod om frisdrankautomaten in scholen te plaatsen, een verbod op het gebruik van transvetten in de HORECA, of een verbod op reclame voor snoep en snacks gericht op kinderen. Zij pleiten veelal voor een actieve rol van de overheid in de vorm van wetgeving ter regulering van de obesogene omgeving.

Tweede voorbeeld van tegengestelde morele denkkaders

a. Druggebruik is eenvoudig verkeerd

Ter illustratie van vigerend preventiebeleid, waarbij morele argumenten een grote rol spelen bij de rechtvaardiging, kan de 'war on drugs' in Amerika dienen. Bennet, een voorvechter van de 'war on drugs' stelt (Maris, 2006, p.89) (Bennet, 1991):

> *"Druggebruik is eenvoudig verkeerd. En uiteindelijk is het morele argument het meest dwingende argument."*

b. Een verbod op druggebruik werkt averechts

In diverse publicaties bestrijdt Maris de gerechtvaardigdheid van de strafbaarstelling van druggebruik (Maris, 2006, p.103) (Maris, 1999):

> *"Uit de ervaring met de Amerikaanse drooglegging blijkt dat een strafrechtelijk verbod soms onbedoeld meer schade veroorzaakt dan voorkómt. Voorstanders van liberalisering van drugs gebruiken de vergelijking met de Prohibitie dan juist ook tegen het Amerikaanse oorlogsmodel. Ze betogen dat nu op wereldschaal met drugs precies hetzelfde gebeurt als in de jaren twintig van de vorige eeuw met alcohol in de Verenigde Staten. De oorlog tegen drugs jaagt druggebruikers in het illegale circuit, van waaruit ze anderen grote schade berokkenen."*

Deze tegenstelling tussen Bennet en Maris lijkt te berusten op het verschil tussen respectievelijk een *deontologische* en *teleologische* rechtvaardiging van vrijheidsbeperkende preventiemaatregelen. De deontologische en teleologische benadering vormen hoofdstromingen binnen de normatieve ethiek. Deontologisch is afgeleid van het Griekse woord 'deoon', dat plicht of datgene dat gedaan moet worden, betekent. Bij een deontologische beoordeling van de gerechtvaardigdheid van een handeling, is doorslaggevend of de handeling in zichzelf goed of slecht is. Of de gevolgen van een handeling goed of slecht zijn, wordt van ondergeschikt belang geacht. Teleologisch is afgeleid van het Griekse woord 'telos' dat doel betekent. Bij een teleologische beoordeling van de gerechtvaardigdheid van een handeling, is doorslaggevend of de gevolgen van de handeling goed of slecht zijn. Of de handeling in zichzelf goed of slecht is, wordt van ondergeschikt belang geacht. In plaats

van teleologische wordt ook wel over een utilistische (utilis = nuttig) benadering gesproken. (Graaf, 1980, p.31)

Op basis van de literatuur die ik heb bestudeerd, kom ik tot de conclusie dat de wetenschap meestal kiest voor een eenzijdig teleologische benadering inzake de rechtvaardiging van vrijheidsbeperkende preventiemaatregelen ter bestrijding van leefstijlgerelateerde aandoeningen. Wetenschappelijk kan echter niet worden bewezen dat deontologische argumenten ter rechtvaardiging van dergelijke maatregelen ongeldig zijn. Ethische opvattingen zijn afhankelijk van tijd, plaats en persoon en dat geldt ook voor de waardering van teleologische en deontologische argumenten ter onderbouwing van preventiebeleid. Mijn streven is om een algemeen geldig en toepasbaar model voor de beoordeling van de gerechtvaardigdheid van preventiemaatregelen te ontwikkelen. Een voorwaarde voor algemene geldigheid en toepasbaarheid is m.i. dat het model 'ethisch neutraal' is. Het model biedt daarom ruimte voor zowel teleologische als deontologische argumenten ter rechtvaardiging van preventiemaatregelen.

Voorbeeld van tegengestelde politiek-ideologische denkkaders

a. De overheid dient burgers richting een gezonde leefstijl te sturen

Volgens Giddens dienen de traditionele Westerse verzorgingsstaten te worden omgevormd naar moderne sociale investeringsstaten, waarin de overheid in burgers investeert en burgers activeert om hun capaciteiten te ontwikkelen en te gebruiken. In een traditionele verzorgingsstaat richt de overheid zich bijvoorbeeld primair op het verzorgen van werkloosheidsuitkeringen, terwijl de overheid zich in een sociale investeringsstaat primair richt op het investeren in de opleiding en werkervaring van burgers (sociaal investeren) en op het met financiële prikkels activeren van burgers (life politics) om betaald te werken. (Giddens, 1994, p.14-15, 214) Volgens Scholtmeijer biedt het concept van life politics kansrijke aanknopingspunten voor de bestrijding van overgewicht onder de bevolking. Bijvoorbeeld door als overheid de voedingsmiddelenindustrie te dwingen tot het wijzigen van portiegroottes en door de prijzen van (on)gezonde producten te beïnvloeden. (Scholtmeijer, 2006, p.226, 230). Het voeren van life politics op het terrein van de volksgezondheid, betekent dat de overheid de leefstijl van burgers actief richting gezondheid stuurt.

b. De overheid dient zich aangaande leefstijl neutraal op te stellen

De overheid dient zich aangaande de leefstijl van individuele burgers neutraal op te stellen. Een neutrale opstelling houdt in, dat de overheid alleen mag ingrijpen in de leefstijl van individuele burgers, als deze leefstijl schade toebrengt aan derden (Holtug, 2002, p.362), (Clarke, 2006). Dit uitgangspunt, dat bekend staat als het schadebeginsel, is reeds in 1851 geformuleerd door Mill, maar nog steeds van grote invloed op het overheidsbeleid (wetgeving) in Westerse democratieën. Het recht op het kiezen van een eigen, gezonde of ongezonde, leefstijl is een grondrecht binnen de Westerse democratieën. Er bestaat geen rechtvaardiging voor een plicht tot gezond leven (Dillmann, 1991, p.128-129).

Volgens de klassieke interpretatie van het schadebeginsel dient het concept van life politics van Giddens als een vorm van onaanvaardbaar overheidspaternalisme van de hand te worden gewezen. Sinds 1851 is het klassieke schadebeginsel door diverse auteurs echter steeds verder genuanceerd c.q. 'opgerekt' Bijvoorbeeld door te stellen dat het individu door schade toe te brengen aan zichzelf, indirect schade toebrengt aan derden. Denk bijvoorbeeld aan een ongezonde leefstijl die leidt tot arbeidsongeschiktheid, waardoor men afhankelijk wordt van een uitkering, die door derden (de werkenden) moet worden betaald. De laatste decennia pleiten diverse auteurs er voor om het schadebeginsel niet teveel op te rekken, want met creatief redeneren leidt alle schade die het individu zichzelf toebrengt tot (emotionele) schade aan derden, en dan is het schadebeginsel niet meer onderscheidend (Pope, 2005, p.166-179).

Hoewel de juiste operationalisering van het concept 'schade aan derden' onderwerp is van een (somtijds verhit) wetenschappelijk debat, dat al sinds de formulering van het schadebeginsel in 1851 wordt gevoerd, is het m.i. evident dat een beoordeling van gerechtvaardigdheid van preventiemaatregelen vanuit het denkkader van de liberale staat en het schadebeginsel leidt tot andere uitkomsten dan een beoordeling vanuit het denkkader van de sociale investeringsstaat en life politics.

Voorbeeld van tegengestelde sociaal-culturele denkkaders

(Swinburn, 1999, p.567) (Lang, 2006, p.12)

a. Mensen eten en drinken vooral thuis

Veruit het grootste deel van de consumptie van eten en drinken vindt plaats in de thuissituatie of wordt in de thuissituatie bereid (lunch voor op het werk). Het is

daarom ter bestrijding van overgewicht weinig zinvol om restaurants te verplichten informatie te verstrekken over de samenstelling van en het aantal calorieën in de producten die zij serveren.

b. Mensen eten en drinken vooral buitenshuis

Tegenwoordig besteden Amerikanen ongeveer de helft van hun uitgaven aan eten en drinken buitenshuis. Daarom is de redenering, dat het ter bestrijding van overgewicht weinig zinvol is om restaurants te verplichten informatie te verstrekken over de samenstelling van en het aantal calorieën in de producten die zij serveren, achterhaald (Hayne, 2004, p.394).

Voorbeeld van tegengestelde juridische denkkaders

a. Beperkende wetgeving gericht op de voedingsmiddelenindustrie werkt averechts

Alle jurisprudentie wijst er op dat bedrijven in de voedingsmiddelenindustrie niet aansprakelijk kunnen worden gesteld voor eventuele gezondheidsschade ten gevolge van overgewicht. De reden is dat individuen primair zelf verantwoordelijk zijn voor hun gewicht en niemand hen dwingt om ongezond te eten of te weinig te bewegen. Van wetgeving die de voedingsmiddelenindustrie beknopt in haar mogelijkheden zijn voornamelijk averechtse effecten te verwachten. Dergelijke wetgeving zal er toe leiden dat individuen zich nog meer kunnen onttrekken aan de eigen verantwoordelijkheid voor hun gezondheid (McMenamin, 2006, p.517-518).

b. Beperkende wetgeving gericht op de voedingsmiddelenindustrie is nuttig

Dat het vooralsnog niet gelukt is om bedrijven individueel aansprakelijk te stellen voor de gezondheidsschade ten gevolge van overgewicht, betekent geenszins dat individuen primair aansprakelijk zijn voor overgewicht. De voedingsmiddelenindustrie is collectief verantwoordelijk voor het ontstaan van de obesogene omgeving (Alderman, 2007, p.101-102). Wetgeving lijkt goede aanknopingspunten te bieden om de obesogene omgeving te saneren (Alderman, 2007, p.102), (Gostin, 2007), (Ashe, 2007).

Voorbeeld van tegengestelde financieel-economische denkkaders

a. Consumptiebeperkende preventiemaatregelen zijn financieel-economisch schadelijk

Preventiemaatregelen die consumptie van bepaalde levens- en genotsmiddelen trachten te verminderen, schaden het financiële belang van de voedingsmiddelen-industrie. Bovendien vormen zij een aantasting van de vrije marktwerking, die aan de basis ligt van de welvarende Westerse economieën.

b. Consumptiebeperkende preventiemaatregelen zijn financieel-economisch nuttig

De obesogene omgeving vormt een verstoring van de vrije marktwerking en wetgeving kan deze verstoring corrigeren (Hayne, 2004, p.391). De consumptie van voedingsmiddelen wordt aan consumenten zo sterk opgedrongen, dat zij niet in staat zijn om als collectief de in een vrije markt noodzakelijke countervailing power te vormen tegen de producenten van voedingsmiddelen. De overconsumptie van voedingsmiddelen leidt tot economische schade door het grotere beroep dat mensen met overgewicht doen op de collectief gefinancierde gezondheidszorg en door een vermindering van de arbeidsproductiviteit. Bovendien hebben bedrijven een ‘Corporate Social Responsibility’. Bedrijven achten verantwoord maatschappelijk ondernemen – ondermeer doordat hun producten bijdragen aan de gezondheid van consumenten – van belang voor hun financiële succes op de langere termijn. (Lang, 2006, p.13-15), (Adams, 2007)

Bijlage 7: Artikel over casus Clarian Health in BusinessWeek

Top News August 2, 2007, 12:01AM

Being Unhealthy Could Cost You—Money

Clarian Health is taking a novel approach to reducing health-care costs: It's penalizing workers for indicators of poor health

by Jena McGregor

For employees at Clarian Health, feeling the burn of trying to lose weight will take on new meaning.

In late June, the Indianapolis-based hospital system announced that starting in 2009, it will fine employees $10 per paycheck if their body mass index (BMI, a ratio of height to weight that measures body fat) is over 30. If their cholesterol, blood pressure, and glucose levels are too high, they'll be charged $5 for each standard they don't meet. Ditto if they smoke: Starting next year, they'll be charged another $5 in each check.

Clarian has been making headlines for its aggressive and unusual approach to covering escalating health-care costs. Rather than taking the more common step of giving employees incentives for merely participating in its wellness programs, such as joining a smoking cessation group or using a health coach, Clarian is actually measuring outcomes. And unlike most employers, it is penalizing workers for poor health instead of rewarding them for taking healthy steps.

"More Transparent"

But some employment lawyers and wellness program administrators believe Clarian's approach may not be so unusual in coming years. They see employers, already overwhelmed by rising health-care costs, getting more aggressive in mandating changes in employee behavior. Garry Mathiason, a senior partner at employment law firm Littler Mendelson, says more than 300 companies have requested its

assistance on mandatory wellness initiatives since it released a study on the topic in April. "In reality, you only get a certain amount of participation with incentive and encouragement," he says. "The demand for [curtailing health-care costs] is so great that [employers] are willing to take the next step. It's tough love." As BusinessWeek chronicled in February, some outlier companies have even banned tobacco use for its employees altogether (see BusinessWeek.com, 2/26/07, "Get Healthy—or Else").

In addition, regulations that became effective July 1 could prompt cautious employers to step off the sidelines. The federal government recently issued final rules on how wellness programs could comply with the nondiscrimination conditions of the Health Insurance Portability & Accountability Act (HIPAA). While the new regulations have been proposed for years, the final rules provide employers with some sense of security and more clarification on how much they can reward or penalize employees based on specific health results. "When you get into things that involve discrimination, employers aren't very comfortable with the words 'proposed regulation,'" says Jerry Ripperger, director of consumer health for the Principal Financial Group, a financial-services and insurance firm that offers wellness programs for large employers.

The final regulations were a major catalyst for Clarian Health's new program. Steve Wantz, Clarian's senior vice-president of administration and human resources, says his team had been considering their plan for two years, but the new regulations "gave us a lot more confidence." Another change in Indiana state law that allowed employers to offer financial incentives or surcharges to smokers in their health plans was also an impetus. After benchmarking other companies, Clarian, which had already been encouraging employees to join smoking cessation programs and take health risk tests, decided charging employees was more "transparent." Other companies "were providing what they called incentives through credits or discounts toward health premiums," says Wantz. "What we found was what those employers were doing, many times, was raising their premiums and discounting them back."

Sticks and Carrots

At Clarian, employees who have blood pressure that's above 140 over 90, blood glucose levels over 120, low-density lipoprotein cholesterol over 130, or a BMI over 29.9 could be subject to the paycheck deductions. Of the company's 13,000 employees, about 8,000 are enrolled in the company's health plan. The company estimates that as many as 34% of its employees will meet the definition of being

obese, while it expects lower levels for other health measures. About 26% are tobacco users. The fines are waived for employees who can provide a doctor's note stating it's not advisable for them to try to meet the benchmark—employees will be able to submit new notes from their doctors quarterly—and that they are complying with the proper diet, exercise, and treatment plan.

While Clarian's plan is highly unusual, a few other employers are using the stick approach, too. Western & Southern Financial Group, a Cincinnati-based insurance and financial-services provider, has been issuing employees with high BMIs a surcharge to their premiums since 2001. Depending on their scores, employees are charged a tiered fee of between $15 and $75 a month if their self-reported BMI is high, but can have those fees refunded if their numbers go down. Preventure, a provider of fitness and wellness programs for midsize to large employers, has one client in the manufacturing industry that rewards employees when their BMI falls from their baseline score and charges them more when their number rises. "We haven't seen a lot of companies do it," says Laura Gilligan, director of business development for Preventure, "but I sense it might be a trend."

What's more likely for employers who start measuring health results is that they will provide carrots. In July, UnitedHealthcare began offering a new plan targeted at small businesses called Vital Measures, which combines a high-deductible insurance plan with a supplemental plan that provides discounts for healthy outcomes. Members who choose to participate will receive $500 discounts toward their deductible for each cholesterol, blood pressure, and BMI level they meet, along with refraining from tobacco use or taking a health risk assessment.

Legal Questions

A reward-driven approach will surely be better received by employees, say benefits consultants. "The concept of penalizing for poor health is not well accepted, and a lot of employees would react badly to it," says Bruce Kelley, a consultant in the health-care practice of human resources advisory firm Watson Wyatt Worldwide. Clarian Health admits that its program is aggressive by design, and that employee reaction has been mixed, with much debate on its internal message boards. While some employees were supportive, there's been "the other reaction that this is very personal," says Wantz, with people asking, "'How dare you? This is my personal space.' There's been a lot of questions and confusion."

That's why some benefits consultants and health-care groups think many companies won't adopt a plan like Clarian's anytime soon. "People are not interested in

making their employees unhappy," says Helen Darling, president of the National Business Group on Health, a nonprofit that represents large employers' health-care interests. "We're in a war for talent, and there are more than enough work-related challenges to manage people and performance that [penalizing] for something that is so complicated, so personal, so hard to deal with, just isn't the right thing to do at this point."

Others may steer clear of such aggressive plans until questions are ironed out about other legal issues besides HIPAA. Sharon Cohen, Watson Wyatt's group and health-care benefits counsel, says that "any time an employer tries to influence employee behavior and they start to become more aggressive, there are other laws that are implicated," including the Americans with Disabilities Act. She says it's not clear how the requirement of taking a health risk assessment to be eligible for an insurance plan, like Clarian's, would be seen by the Equal Employment Opportunity Commission under the ADA. Clarian's Wantz says their plan has been extensively reviewed by attorneys.

Still, Clarian's approach has already attracted interest. Wantz says it has received calls from several local companies, along with other hospital systems, interested in their approach. "The most common response from other employers," he says, "has been 'thank you.'"

McGregor is BusinessWeek's management editor.

Bijlage 8: Toelichting op paragraaf f van HIPAA

In het algemeen verbiedt de Amerikaanse wetgeving (HIPAA)[1] het werkgevers om bij het aanbieden van een collectief gezondheidsplan ("group health plan") te discrimineren tussen werknemers op basis van gezondheidsfactoren. Dit verbod op discriminatie betreft de toegang van de werknemers tot het gezondheidsplan, de inhoud ("benefits") van het gezondheidsplan, en de financiële bijdrage van de werkgever aan het gezondheidsplan. Een gezondheidsplan kan bestaan uit een welzijnsprogramma ("wellness program") of de combinatie van een welzijnsprogramma en een zorgverzekering. In geval van Clarian bestaat het gezondheidsplan uit deze combinatie. Paragraaf f van de HIPAA maakt een uitzondering op het hierboven beschreven verbod op discriminatie mogelijk:

> *"HIPAA's nondiscrimination provisions generally prohibit a group health plan or group health insurance issuer from denying an individual eligibility for benefits based on a health factor and from charging an individual a higher premium than a similarly situated individual based on a health factor. An exception provides that plans may vary benefits (including cost-sharing mechanisms) and premiums or contributions based on whether an individual has met the standards of a wellness program that complies with paragraph (f) of the regulations. … If only employees are eligible to participate, the amount of the reward must not exceed 20% of the cost of employee-only coverage under the plan. If employees and any class of dependents are eligible to participate, the reward must not exceed 20% of the cost of coverage in which an employee and any dependents are enrolled."* (US DOL. HIPAA. 2008)

Deze uitzondering kan alleen van toepassing zijn als een bonafide welzijnsprogramma (*"bona fide wellness program"*) onderdeel uitmaakt van het gezondheidsplan. In dat geval mag er onder voorwaarden worden gediscrimineerd tussen werknemers op basis van gezondheidsfactoren m.b.t. de financiële bijdrage van de werkgever aan het gezondheidsplan. De gezondheidsfactoren op basis waarvan wordt gediscrimineerd, dienen 'onderdeel uit te maken' van het betreffende welzijnsprogramma. In een 'Field Assistance Bulletin' heeft het U.S. Department of Labor (US DOL) paragraaf f van de HIPAA uitgewerkt (US DOL. Field Assistance Bulletin. 2008). Het bulletin beschrijft wanneer er sprake is van een 'bonafide welzijnsprogramma', aan welke overige voorwaarden het gezondheidsplan moet vol-

1. Health Insurance Portability and Accountability Act (US DOL. HIPAA. 2008).

doen, en in welke omvang financiële beloningen en/of toeslagen zijn toegestaan. Het begrip "welzijnsprogramma" wordt door het US DOL in dit verband ruim gedefinieerd:

> *"A wide range of wellness programs exist to promote health and prevent disease. ... Examples include: a program that reduces individual's cost-sharing for complying with a preventive care plan; a diagnostic testing program for health problems; and rewards for attending educational classes, following healthy lifestyle recommendations, or meeting certain biometric targets (such as weight, cholesterol, nicotine use, or blood pressure targets).".*

De 'financiële discriminatie' tussen werknemers op basis van gezondheidsfactoren mag niet meer bedragen dan 20% van de totale jaarlijkse kosten voor deelname aan het gezondheidsplan. Het gezondheidsplan van Clarian voldoet hieraan omdat Clarian de kosten voor het niet voldoen van de werknemer aan de gestelde gezondheidseisen heeft gemaximeerd op $25 per loonstrookje. In de discussie in de media is er overeenstemming dat het preventieplan van ***Clarian voldoet aan*** de voorwaarden van de ***HIPAA*** (paragraaf f).

Bijlage 9: Sociaaleconomische gezondheidsverschillen samengevat (Lucht, 2009)

Grote gezondheidsverschillen naar sociaaleconomische status

Er bestaan in Nederland aanzienlijke verschillen in gezondheid naar sociaaleconomische status (SES), gemeten naar opleiding inkomen of beroepsniveau. Dit worden sociaaleconomische gezondheidsverschillen genoemd. Het verschil in levensverwachting tussen laag en hoog opgeleide mannen en vrouwen is 6,9 respectievelijk 5,7 jaar. Bij de gezonde levensverwachting zijn de sociaaleconomische verschillen groter: lager opgeleide mannen en vrouwen leven respectievelijk 18,8 en 16,4 jaar minder in als goed ervaren gezondheid dan hoger opgeleide mannen en vrouwen.

Oorzaak gezondheidsverschillen bij risicofactoren en gezondheidsselectie

De minder goede gezondheid van mensen met een lage SES komt voor een deel door het verhoogd voorkomen van gezondheidsrisico's. Personen met een lage SES hebben een minder gezonde leefstijl: roken en drinken meer, eten minder groenten en fruit en bewegen minder. Ook zijn materiële omstandigheden (woon en werkomstandigheden) vaak minder gunstig voor de gezondheid. Gezondheidsverschillen zijn echter ook voor een deel het gevolg van gezondheidsselectie. Juist een slechtere gezondheidstoestand kan ertoe hebben geleid dat iemand tot een lagere SES-groep behoort. Tot slot blijft tot op heden een deel van de samenhang tussen SES en gezondheid onverklaard.

Geen grote verschillen in gebruik gezondheidszorg tussen SES-groepen

Verschillen in het gebruik van de gezondheidszorg tussen sociaaleconomische status groepen zijn gering. In overeenstemming met hun gezondheidstoestand maken personen met een lage SES meer gebruik van de gezondheidszorg. Lagere SES-groepen maken ook minder gebruik van preventieve voorzieningen.

Bijlage 10: Rapport van TNS NIPO biedt geen uitsluitsel over economische effecten van wettelijk rookverbod voor kleine cafés

Op 1 december 2009 informeert minister Klink de Tweede Kamer per brief over de (neven)effecten van 1 jaar rookvrije horeca[1]. Hij baseert zich hierbij op vier onderzoeksrapporten die hij als bijlagen aan deze brief heeft toegevoegd:

1. Even uitblazen. Eén jaar rookvrije horeca. Eindrapport. Regioplan. (Dekker, 2009);
2. Economische effecten van het rookverbod in de horeca. Evaluatie van het eerste jaar 1 juli 2008 - 1 juli 2009. TNS/NIPO. (Schieveen, 2009);
3. Quick scan. Gemeenten en rookvrije horeca. Eerste jaar na invoering 2008-2009. (Intraval, 2009);
4. De tabaksvrije coffeeshop. Gevolgen van de invoering van de rookvrije horeca voor Nederlandse coffeeshops. Trimbos Instituut. (Vogels, 2009).

Het rapport van Regioplan baseert zich bij uitspraken over 'de oppervlakte van cafés in relatie tot omzetveranderingen' op het rapport van TNS NIPO. Voor zover ik het kan beoordelen is minister Klink op basis van de het rapport van TNS NIPO (of op basis van een samenvatting hiervan in het rapport van Regioplan) in zijn brief d.d. 1 december 2009 tot de volgende conclusie gekomen:

> *"Net als in mijn brief van 16 december 2008 is mijn conclusie dat er vooralsnog geen grond is om vast te stellen dat de rookvrije horeca voor bepaalde sectoren of (sub) groepen buiten proportioneel zou uitwerken."* (p.5)

Deze conclusie van minister Klink is m.i. meer gebaseerd op het ontbreken van gedetailleerde onderzoekscijfers, waardoor hierover geen conclusies kunnen worden getrokken ('*er is geen grond om vast te stellen*'), dan op duidelijke aanwijzingen dat kleine cafés zonder personeel niet buitenproportioneel door het rookverbod worden geraakt. Ik zal dit toelichten aan de hand van drie overzichten:

1. TK, 2009–2010, 32011, nr.3.

– ***overzicht 31:***
  toont de tabel in het rapport van TNS NIPO over de veranderingen in omzet van cafés gerelateerd aan omvang;

– ***overzicht 31a:***
  toont een nadere uitwerking van overzicht 31 m.b.t. het jaar 2008;

– ***overzicht 31b:***
  toont een nadere uitwerking van overzicht 31 m.b.t. het jaar 2009.

Overzicht 31: veranderingen in omzet cafés gerelateerd aan omvang (TNS NIPO, 2009)

	**Totaal**	**Totaal**	**<34**	**<34**	**35-49**	**35-49**	**50-99**	**50-99**	**100-199**	**100-199**	**200-499**	**200-499**	**>500**	**>500**
	08	***09***	***08***	***09***	***08***	***09***	***08***	***09***	***08***	***09***	***08***	***09***	***08***	***09***
n=[9]	*49*	*75*	*1*	*2*	*3*	*3*	*13*	*20*	*12*	*20*	*15*	*25*	*4*	*4*
	%	%	%	%	%	%	%	%	%	%	%	%	%	%
**Stijging**														
> 30%	4	7	-	-	-	-	8	15	9	5	-	4	-	-
20-30%	12	5	-	-	33	-	8	10	-	5	6	4	-	-
15-20%	10	13	-	51	35	-	8	-	17	20	7	20	-	-
10-15%	8	25	-	-	-	34	23	25	42	25	26	24	25	25
5-10%	40	27	100	49	33	33	46	30	24	20	7	24	75	50
0-5%	14	13	-	-	-	33	-	5	8	10	33	24	-	-
weet niet	12	9	-	-	-	-	8	15	-	15	20	-	-	25
n=	*270*	*271*	*3*	*2*	*24*	*23*	*134*	*108*	*71*	*70*	*27*	*42*	*9*	*14*
	%	%	%	%	%	%	%	%	%	%	%	%	%	%
**Daling**														
0-5%	8	10	-	-	4	4	7	9	9	14	15	10	11	7
5-10%	12	21	-	50	12	26	9	19	16	26	15	14	23	36
10-15%	17	15	-	50	17	**4**	16	20	23	9	15	10	-	22
15-20%	14	14	33	-	12	-	14	17	13	17	19	12	11	7
20-30%	20	12	-	-	12	22	23	12	15	10	19	7	33	14
> 30%	19	17	-	-	29	26	19	16	17	17	14	24	11	-
weet niet	10	12	67	-	12	17	10	7	8	7	4	24	11	14

Basis: alle respondenten met een café   Bron: TNS NIPO, 2009

(n=[9]: de ‘[9]’ is een voetnoot in het rapport van TNS NIPO die luidt: *“let op: kleine n-en”*, waarmee wordt bedoeld dat het om een deel van de steekproef gaat)

Toelichting op overzicht 31:

- De bovenste rij van de tabel betreft (vanaf de vierde cel) de oppervlakte van de cafés (kleiner dan 34m²; van 35 m² t/m 49 m²; van 50m² t/m 99 m²; enz.);
- In de rij daaronder (08,09; enz.) betreft '08' de steekproef in de periode 'juli t/m december 2008' en betreft '09' de steekproef in de periode 'januari t/m juni 2009';
- De rij daaronder (n=, 49, 75, 1, 2, 3, 3, 13, 20, enz.) betreft het aantal cafés in de steekproef met een omzetstijging.
- De rij ongeveer halverwege de tabel (n=, 270, 271, 3, 2, 24, 23, 134, 108, enz.) betreft het aantal cafés in de steekproef met een omzetdaling.
- De eerste kolom geeft eerst de schaalverdeling van de omzetstijging (>30%; 20-30%; 15-20%; enz.) en daarna de schaalverdeling van de omzetdaling;

Een ***eerste bezwaar*** van overzicht 31 is dat omzetdalingen c.q. –stijgingen boven de 30% niet worden uitgesplitst (het kan bijvoorbeeld gaan om 31%, maar ook om 50% of 75%).

Een ***tweede bezwaar*** van overzicht 31 is dat de percentages die de omzetstijgingen en omzetdalingen weergeven niet de totale steekproef betreffen:

- In 2008 betreft de totale steekproef (49 + 270=) 319 cafés, waarvan 49 met een omzetstijging en 270 met een omzetdaling. De weergegeven percentages van cafés met een omzetstijging betreffen niet de totale steekproef (N=319), maar de cafés met een omzetstijging (n=49). De weergegeven percentages van cafés met een omzetdaling betreffen niet de totale steekproef (N= 319), maar de cafés met een omzetdaling (n=270).
- In 2009 betreft de totale steekproef (75 + 271=) 346 cafés, waarvan 75 met een omzetstijging en 271 met een omzetdaling. De weergegeven percentages van cafés met een omzetstijging betreffen niet de totale steekproef (N=346), maar de cafés met een omzetstijging (n=75). De weergegeven percentages van cafés met een omzetdaling betreffen niet de totale steekproef (N=346), maar de cafés met een omzetdaling (n=271).

Om de betekenis van de omzetstijgingen en -dalingen goed te duiden, moeten deze worden uitgedrukt als percentage van de totale steekproef. In tabel 31a heb ik dit gedaan voor de omzetdalingen in het jaar 2008 en in tabel 31b voor de omzetdalingen in het jaar 2009

Overzicht 31a: omzetdalingen als percentage van de totale steekproef in 2008

De grijs-gearceerde informatie is overgenomen uit de tabel van TNS NIPO (zie overzicht 31).

Tabel 31a.1	Najaar 2008 (in absolute aantallen)					
	<34m²	35-49m²	50-99m²	100-199m²	200-499m²	>500m²
Stijging (totaal)	1	3	13	12	15	4
Daling (totaal)	3	24	134	71	27	9
Stijging+Daling (totaal)	4	27	147	83	42	13

Tabel 31a.2	Najaar 2008 (in afgeronde percentages)					
	<34m²	35-49m²	50-99m²	100-199m²	200-499m²	>500m²
Daling (totaal)	3	24	134	71	27	9
Daling (0-5%)	0%	4%	7%	9%	15%	11%
Daling (5-10%)	0%	12%	9%	16%	15%	23%
Daling (10-15%)	0%	17%	16%	23%	15%	0%
Daling (15-20%)	33%	12%	14%	13%	19%	11%
Daling (20-30%)	0%	12%	23%	15%	19%	33%
Daling (> 30%)	0%	29%	19%	17%	14%	11%
Weet niet	67%	12%	10%	8%	4%	11%

Tabel 31a.3	Najaar 2008 (niet afgerond op gehele aantallen)					
	<34m²	35-49m²	50-99m²	100-199m²	200-499m²	>500m²
Daling (totaal)	3	24	134	71	27	9
Daling (0-5%)	0,00	0,96	9,38	6,39	4,05	0,99
Daling (5-10%)	0,00	2,88	12,06	11,36	4,05	2,07
Daling (10-15%)	0,00	4,08	21,44	16,33	4,05	0,00
Daling (15-20%)	0,99	2,88	18,76	9,23	5,13	0,99
Daling (20-30%)	0,00	2,88	30,82	10,65	5,13	2,97
Daling (> 30%)	0,00	6,96	25,46	12,07	3,78	0,99
Weet niet	2,01	2,88	13,40	5,68	1,08	0,99

Tabel 31a.4	Najaar 2008					
	<34m²	35-49m²	50-99m²	100-199m²	200-499m²	>500m²
Stijging + Daling (totaal)	4	27	147	83	42	13
Daling (20-30%)	0,0%	10,7%	21,0%	12,8%	12,2%	22,8%
Daling (> 30%)	0,0%	***25,8%***	17,3%	14,5%	9,0%	7,6%
Daling > 20%	0,0%	***36,4%***	38,3%	27,4%	21,2%	30,5%

Overzicht 31b: omzetdalingen als percentage van de totale steekproef in 2009
De grijs-gearceerde informatie is afkomstig uit de tabel van TNS NIPO (zie overzicht 31).

Tabel 31b.1	Voorjaar 2009 (in absolute aantallen)					
	<34m²	35-49m²	50-99m²	100-199m²	200-499m²	>500m²
Stijging (totaal)	2	3	20	20	25	4
Daling (totaal)	2	23	108	70	42	14
Stijging + Daling (tot.)	4	26	128	90	67	18

Tabel 31b.2	Voorjaar 2009 (in afgeronde percentages)					
	<34m²	35-49m²	50-99m²	100-199m²	200-499m²	>500m²
Daling (totaal)	2	23	108	70	42	14
Daling (0-5%)	0%	4%	9%	14%	10%	7%
Daling (5-10%)	50%	26%	19%	26%	14%	36%
Daling (10-15%)	50%	4%	20%	9%	10%	22%
Daling (15-20%)	0%	0%	17%	17%	12%	7%
Daling (20-30%)	0%	22%	12%	10%	7%	14%
Daling (> 30%)	0%	26%	16%	17%	24%	0%
Weet niet	0%	17%	7%	7%	24%	14%

Tabel 31b.3	Voorjaar 2009 (niet afgerond op gehele aantallen)					
	<34m²	35-49m²	50-99m²	100-199m²	200-499m²	>500m²
Daling (totaal)	2	23	108	70	42	14
Daling (0-5%)	0,00	0,92	9,72	9,80	4,20	0,98
Daling (5-10%)	1,00	5,98	20,52	18,20	5,88	5,04
Daling (10-15%)	1,00	0,92	21,60	6,30	4,20	3,08
Daling (15-20%)	0,00	0,00	18,36	11,90	5,04	0,98
Daling (20-30%)	0,00	5,06	12,96	7,00	2,94	1,96
Daling (> 30%)	0,00	5,98	17,28	11,90	10,08	0,00
Weet niet	0,00	3,91	7,56	4,90	10,08	1,96

Tabel 31b.4	Voorjaar 2009					
	<34m²	35-49m²	50-99m²	100-199m²	200-499m²	>500m²
Stijging + Daling (tot.)	4	26	128	90	67	18
Daling (20-30%)	0,0%	19,5%	10,1%	7,8%	4,4%	10,9%
Daling (> 30%)	0,0%	***23,0%***	13,5%	13,2%	15,0%	0,0%
Daling > 20%	0,0%	***42,5%***	23,6%	21,0%	19,4%	10,9%

Toelichting op overzicht 31a
Overzicht 31a heeft betrekking op de steekproef van TNS NIPO in 2008. Het doel van de tabellen 31a.1 t/m 31a.3 is om te komen tot tabel 31a.4. In tabel 31a.4 wordt weergeven welk percentage cafés van de totale steekproef in 2008 een omzetdaling heeft van meer dan 20% en van meer dan 30%. Hierbij is een onderverdeling gemaakt naar oppervlakte van de cafés.

De gegevens in de tabellen 31a.1 en 31a.2 zijn (grotendeels) overgenomen uit de tabel van TNS NIPO (zie overzicht 31). In tabel 31.a.3 zijn de percentages uit tabel 31a.2 omgezet in absolute aantallen (omdat tabel 31a.2 afgeronde percentages weergeeft, leidt dit tot kleine afrondingsfouten). De percentages in tabel 31a.4 zijn berekend door het combineren van de aantallen in tabel 31a.3 met de onderste rij van tabel 31a.1.

Toelichting op overzicht 31b
Overzicht 31b heeft betrekking op de steekproef van TNS NIPO in 2009. Overzicht 31b kent precies dezelfde opbouw als overzicht 31a. Daarom wordt voor de toelichting op overzicht 31b verwezen naar de toelichting op overzicht 31a.

Conclusies over economische effecten van wettelijk rookverbod voor kleine cafés
In 2009 definieert minister Schippers in het 'Ontwerpbesluit houdende wijziging van het Besluit uitvoering rookvrije werkplek, horeca en andere ruimten' kleine cafés als cafés met een oppervlakte van minder dan 70m^2.[2] De cafés met een oppervlakte van 35m2 t/m 49m2 vallen hier dus zeker onder.

De overzichten 31a en 31b laten zien dat met name de kleine cafés met een oppervlakte van 35m^2 t/m 49m^2 een grote terugval in omzet hebben:
- over het laatste half jaar van 2008 rapporteert ***36,4%*** van deze cafés een omzetdaling van meer dan ***20%*** en rapporteert ***25,8%*** een omzetdaling van meer dan ***30%*** (zie tabel 31a.4);
- over het eerste half jaar van 2009 rapporteert ***42,5%*** van deze cafés een omzetdaling van meer dan ***20%*** en rapporteert ***23,0%*** een omzetdaling van meer dan ***30%*** (zie tabel 31b.4).

De bovenstaande ***vetcursief***-gedrukte getallen kan ik niet rijmen met de uitspraak van Minister Klink aan het begin van dit deze bijlage:

2. Bijlage bij TK, 2010–2011, 32011, nr.9.

"Net als in mijn brief van 16 december 2008 is mijn conclusie dat er vooralsnog geen grond is om vast te stellen dat de rookvrije horeca voor bepaalde sectoren of (sub) groepen buiten proportioneel zou uitwerken."

De vetcursief-gedrukte getallen vormen geen bewijs dat het rookverbod voor bepaalde subgroepen buitenproportioneel uitwerkt, maar vormen wel een ***aanwijzing*** dat dit het geval zou kunnen zijn. Een omzetdaling van meer dan 20% lijkt mij ernstig. Een omzetdaling van meer dan 30% is dan zeer ernstig. Zeker als men in acht neemt dat een omzetdaling van meer dan 30% (in theorie) alle percentages tussen de 30% en 100% kan betreffen. Hoe ernstig een omzetdaling van (meer dan) 20% of van (meer dan) 30% is, hangt af van de financiële draagkracht van de betreffende cafés. Juist bij de kleinere cafés zou de financiële draagkracht om grote omzetdalingen op te kunnen vangen wel eens (te) klein kunnen zijn.

In maart 2009 stelt Tweede Kamerlid Agema tijdens een overleg van de vaste Tweede Kamercommissie van VWS met minister Klink, dat een omzetdaling van 4% voor kleine cafés al een groot probleem kan zijn[3]:

"Minister Klink schrijft dat een vermindering van de omzet met 4% hem bescheiden voorkomt, maar dat begrijp ik niet goed. Meestal gaat het zo in een horecabedrijf: de omzet van de eerste week ben je kwijt aan pacht en personeel, van de tweede week aan de toeleveranciers, van de derde week aan verzekeringen, gas, water en elektriciteit en al die andere rekeningen. Als er aan het einde van de rit nog wat overblijft, dan is dat je inkomen. Als dat maar 15 of 20% is en er gaat 4% vanaf, dan valt een heel groot deel van het inkomen weg. Daardoor kunnen horecaondernemers in de problemen komen, en dan vooral door het rookverbod. 70% van de cafés tot 100 m2 heeft een omzetdaling. De minister weet niet hoeveel ondernemingen extra leningen hebben aangevraagd als gevolg hiervan. Hij stelt in de brief dat er geen relatie is met het rookverbod. Het zijn echter vertroebelde cijfers, die ontvlecht zouden moeten worden. Ik heb hier gegevens over 64 cafés, onderverdeeld in roken, rookruimte en niet roken. Bij de cafés waar niet wordt gerookt, is heel duidelijk sprake van een enorme omzetdaling. Dat geldt ook voor de cafés met een rookruimte, want dat is gewoon veel minder gezellig." (p.2)

Ter verduidelijking. De omzetdaling van 4% uit het bovenstaande citaat betreft de horeca als geheel en is afkomstig uit een brief van minister Klink aan de Tweede Kamer d.d. 16 december 2008[4]:

"Koninklijke Horeca Nederland en het Bedrijfschap Horeca en Catering hebben onderzoek laten uitvoeren naar de omzet van horecabedrijven in de maanden juli

3. TK, 2008–2009, 22894, nr.216.
4. TK, 2008–2009, 22894, nr.206.

en augustus 2008. De geïnterviewde horecaondernemers meldden een omzetdaling van gemiddeld 4 procent in de maanden juli en augustus 2008 ten opzichte van juli en augustus 2007." (p.4)

Voor zover ik weet heeft geen (wetenschappelijk) onderzoek plaatsgevonden naar de gevolgen – bijvoorbeeld (dreigende) faillissementen of grote schulden – van de door het NIPO aangetoonde grote omzetdalingen in kleine cafés. In parlementaire documenten heb ik hierover geen informatie kunnen vinden.

M.i. kan op basis van het rapport van TNS NIPO (2009) niet worden geconcludeerd of het rookverbod in de horeca al dan niet buitenproportioneel uitwerkt voor bepaalde (sub)groepen c.q. kleine cafés zonder personeel. De reden dat dit niet kan, is dat de gevolgen voor kleine cafés van de grote omzetdalingen in kleine cafés niet zijn onderzocht. Overigens kan op basis van het rapport van TNS NIPO (2009), om dezelfde reden, ook niet worden geconcludeerd dat kleine cafés zonder personeel wel buiten proportioneel door het rookverbod worden geraakt.

Literatuur

Abigail C, Saguy K en Riley W. Weighing both sides: morality, mortality, and framing contests over obesity. Journal of Health Politics, Policy and Law. Vol.30, no.5, October 2005, (p.869-921).

Adams C. Reframing the obesity debate: McDonald's role may surprise you. Sidebar commentary – industry perspective. Journal of Law, Medicine & Ethics. Vol.35, No.1, spring 2007, (p.154-157).

Alderman J, Smith J, Fried E, and Daynard R. Application of law to thr childhood obesity epidemic. Journal of Law, Medicine & Ethics. Vol.35, No.1, spring 2007, (p.90-112).

Allen A. Privacy and Medicine. Paragraph 2.3: Bodily integrity. Stanford Encyclopedia of Philosophy. First published Wed May 13, 2009.

Altman A. Civil Rights. Stanford Encyclopedia of Philosophy. First published Mon Feb 3, 2003; substantive revision Tue Dec 11, 2007.

Alvarez F, and Soltis M. Preventive medicine: employee wellness programs are prone to legal maladies that require careful monitoring. HR Magazine. Vol.51, no.1. January 2006.

Andrews M. America's Best Health Plans. Desperate to control healthcare costs, employers are rolling out wellness programs with teeth. US News and World report. Posted October 25, 2007.

Ashe M, Feldstein L, Graff S, Kline R, Pinkas D and Zellers L. Local venues for change: legal strategies for healthy environments. Journal of Law, Medicine & Ethics. Vol.35, No.1, spring 2007, (p.138-147).

Baker B. Now, the Stick. Workers Pay for Poor Health Habits. Special to The Washington Post. Tuesday, November 13, 2007.

Bal R en Lindeloof van de A. Publieksparticipatie bij pakketbeslissingen: leren van buitenlandse ervaringen. In: Zicht op zinnige en duurzame zorg. Achtergrondstudie uitgebracht door de Raad voor de Volksgezondheid en Zorg (RVZ) bij het advies Zinnige en duurzame zorg. Zoetermeer, 2006 (p.169-228).

Baron J. Biases in the quantitative measurement of values for public decisions. Psychological Bulletin. 1997, Vol.122, no.1, (p.72-88).

Barry C, Brescoll V, Brownell K and Schlesinger M. Obesity Metaphors: How Beliefs about the Causes of Obesity Affect Support for Public Policy. The Milbank Quarterly. Vol.87, no.1, 2009, (p.7–47).

Bates C, Jarvis M, Connolly G. Report Tobacco additives. Cigarette engineering and nicotine addiction. Bates, Action on Smoking and Health (ASH) (London); Jarvis, Imperial Cancer Research Fund (London); Connolly, Massachusetts Tobacco Control Program (Boston). July 14, 1999.

Bennet W. The plea to legalize drugs is a siren call to surrender. In: M Lyman and G Potter. Drugs in society. Cincinnati, 1991.

Berlin I. Two concepts of liberty. Inaugural lecture delivered by Isaiah Berlin before the University of Oxford on October 31, 1958.

Big Fat Blog. Clarian Health, Others will dock your paycheck if you're fat. The fat acceptance weblog. August 8, 2007.

BMJ. Moving forward on rationing: A clinical view (Norheim O. p.903-904); An ethical perspective (Daniels N, and Sabin J. p.904-905); An economic view (Donaldson C, Bate A, Brambleby P, and Waldner H. p.905-906). British Medical Journal. 2008; 337: a1850, (p.903-906).

Bonsel, G. Evaluatie in de gezondheidszorg. Hoofdstuk 11 in: Volksgezondheid en gezondheidszorg. Redactie: Mackenbach, J. en Maas, P. van der. Elsevier gezondheidszorg. Derde, geheel herziene druk. Maarssen, 2004.

Boomen I van den, Vlaskamp A. Onder voorbehoud. Informatie over de bevoegdheidsregeling voorbehouden handelingen in de Wet BIG. Ministerie van Volksgezondheid, Welzijn en Sport. Rijswijk, september 1996.

Bos, T van den. Loopt preventie voor de chroniciteit uit? In: Essaybundel: Van preventie verzekerd. Acht invalshoeken op preventie en de verzekerde zorg. College voor zorgverzekeringen, juli 2007.

Brownell K. The chronicling of obesity: growing awareness of its social, economic, and political contexts. Journal of Healt Politics, Policy and Law. Vol.30, no.5, October, 2005, (p.955-964).

Brownell K and Warner K. The perils of ignoring history: big tobacco played dirty and millions died. How similar is big food? The Milbank Quarterly. Vol.87, no.1, 2009 (p.259–294).

Brug J, Gunning L en Habbema J. Theorie en methoden van preventie. Hoofdstuk 3 in: Volksgezondheid en Gezondheidszorg. Redactie: Mackenbach J en Maas van der P. Derde druk. Maarssen, 2004.

Brunekreef B, Ayres J, Sigsgaard T, Nazaroff W, Tellep D, Jongste J and Bennink R. Second Hand Smoke in Bars and Restaurants. A second opinion on possibilities to reduce exposure to second hand smoke in bars and restaurants to safe levels. Quality Assurance Netherlands Universities (QANU). November 2009.

BusinessWeek, McGregor J. Being unhealthy could cost you money. August 2, 2007.

Camerer C, Issacharoff S, Loewenstein G, O'Donoghue T, and Rabin M. Regulations for conservatives: behavioral economics and the case for 'asymmetric paternalism'. University of Pennsylvania Law Review, Vol.151, 2003, (p.1211-1254).

Carter I. Positive and Negative Liberty. Stanford Encyclopedia of Philosophy. First published Thu Feb 27, 2003; substantive revision Mon Oct 8, 2007.

Chalmers A. What is this thing called science? University of Queensland Press, St. Lucia, 1976. Vertaling van W. Stange. Wat heet wetenschap? Over aard en status van de wetenschap en haar methoden. Boom, Meppel Amsterdam, 3e druk, 1984.

Childress J, Faden R, Gaar R, Gostin L, Kahn J, Bonnie R, Kass N, Mastroianni A, Moreno J and Nieburg P. Public health ethics: mapping the terrain. Journal of Law, Medicine & Ethics, 30 (2002) (p.170-178).

Childress J, and Bernheim R. Public health ethics. Public justification and public trust. Bundesgesundheitsblatt - Gesundheitsforschung – Gesundheitsschutz. Vol.51, no.2, February 2008, (p.158-163).

Christman J. Autonomy in Moral and Political Philosophy. Stanford Encyclopedia of Philosophy. First published Mon Jul 28, 2003; substantive revision Tue Aug 11, 2009.

Clarke S. Debate: state paternalism, neutrality and perfectionism. The Journal of Political Philosophy: Vol.14, no.1, 2006, (p.111-121).

Coughlin S. How many principles for public health ethics? The Open Public health Journal. 2008, 1, (p.8-16).
Cummins S and Macintyre S. Food environments and obesity – neighbourhood or nation? International Journal of Epidemiology. Vol.35, 2006, (p.100-104).

Dagevos H en Munnichs G (redactie). De obesogene samenleving. Maatschappelijke perspectieven op overgewicht. Amsterdam, 2007.
Daniels N. Accountability for reasonableness. Establishing a fair process for priority setting is easier than agreeing on principles. British Medical Journal. November 25, 2000, (p.1300-1301).
DeCew J. Privacy. Stanford Encyclopedia of Philosophy. First published Tue May 14, 2002; substantive revision Mon Sep 18, 2006.
Dekker ZH, Soethout J en Tijsmans N. Even uitblazen. Eén jaar rookvrije horeca. Eindrapport. Regioplan. Amsterdam, oktober 2009.
Diepen van M. Onderzoek zelfregulering rookbeleid in de horeca en cultuursector. Resultaten 2006: 2e monitor. Rapport. TNS/NIPO. Amsterdam, mei 2007.
Diepen M, Antonis N. horeca en omzet. Onderzoek onder 1016 horecagelegenheden. Samenvatting. TNS/NIPO. Amsterdam, 11 december 2008.
Dillman R. Ethiek en preventie. Ethiek en recht in gezondheidszorg. 6 Oktober 1991, (p.103-199).
Donaldson C, Bate A, Brambleby P and Waldner H. Moving forward on rationing. British Medical Journal. 18 October 2008, (p.903-906).
Dunning e.a.. Commissie Keuzen in de zorg. Kiezen en delen. Advies in hoofdzaken. Den Haag, november 1991.
Dworkin R. Sovereign virtue: the theory and practice of equality. Cambridge, Mass.: Harvard University Press, 2000, (p.270-271).
Dyzenhaus D. John Stuart Mill and the harm of pornography. Ethics. Vol.102, No.3. April, 1992, (p.534-551).

EC. Groenboek. Bevorderen van gezonde voeding en lichaamsbeweging: een Europese dimensie voor de preventie van overgewicht, obesitas en chronische ziekten. Commissie van de Europese Gemeenschappenen. Brussel, 8 december 2005.
EC. Groenboek. Op weg naar een rookvrij Europa: beleidsopties op EU-niveau. Commissie van de Europese Gemeenschappen. Brussel, 30 januari 2007.
EG. Geconsolideerde versie van het verdrag tot oprichting van de Europese Gemeenschap. Publicatieblad van de Europese Gemeenschappen (NL). 24 December 2002. C 325/33.

Feenstra T, Baal van P, Hoogenveen R, Vijgen S, Stolk E en Bemelmans W. Cost-effectiveness of interventions to reduce tobacco smoking in the Netherlands. An application of the RIVM Chronic Disease Model. RIVM report 260601003/2005. Bilthoven, 2005.
Feinberg J. Rights, justice and the bounds of liberty. Princeton, New Jersey, 1980.
Feinberg J. The moral limits of the criminal law. Vol.1. Harm to others. New York, Oxford University Press, 1984.
Feinberg J. The moral limits of the criminal law. Vol.3. Harm to Self. New York, Oxford University Press, 1986.
Finkelstein E, Linnan L, Tate D and Birken B. A Pilot Study Testing the Effect of Different Levels of Financial Incentives on Weight Loss Among Overweight Employees. Journal of

Occupational and Environmental Medicine (JOEM). Vol.49, no.9, September 2007, (p.981-989).
Flegal K, Carroll M, Kit B and Ogden C. Prevalence of obesity and trends in the distribution of body mass index among US adults, 1999-2010. JAMA. 2012; 307(5), (p.491-496).
FoxNews.com. Company to charge 'unhealthy' workers more for insurance. July 1, 2007.
Fuemmeler B, Baffi C, Mâsse L, Atienza A and Evans W. Employer and healthcare policy interventions aimed at adult obesity. American Journal of Preventive Medicine. Vol.32, No.1, 2007, (p.44-51).

Gabel R, Whitmore H, Pickreign J, Ferguson C, Jain A, Shova K and Scherer H. Obesity and the workplace: current programs and attitudes among employers and employees. Health Affairs. Jan/Feb 2009, 28, 1, (p.46-56).
Gates D, Succop P, Brehm B, Gillespie G and Sommers B. Obesity and presenteeism: the impact of body mass index on workplace productivity. Journal of Occupational and Environmental Medicine. Vol.50, no.1, January 2008, (p.39-45).
Gelder B, Blokstra A, Feenstra T. Environmental tobacco smoke in the Netherlands. First estimates of exposure, review of main health effects and overview of available interventions. RIVM. Bilthoven, juni 2008.
Gezondheidsraad: Commissie Screening erfelijke en aangeboren aandoeningen. Genetische screening. Gezondheidsraad. Den Haag, 1994. Publikatie nr.1994/22.
Gezondheidsraad. Volksgezondheidsschade door passief roken. Advies. Den Haag, 18 november 2003.
Giddens A. Beyond left and right: The future of radical politics. Cambridge: Polity Press, 1994.
Gids W de, Opperhuizen A, Jacobs P, Kornaat W, Phaff J, Knoll B, Rambali B, Vleeming W. Reductie van blootstelling aan omgevingstabaksrook in de horeca door ventilatie en luchtreiniging. TNO en RIVM. RIVM, Bilthoven, 2004.
Goel V & Waechter R. Inductive and deductive reasoning. Encyclopedia of human behavior, 2nd Edition. Elsevier. York University, Toronto, Canada. University of Hull, UK. McMaster University. August, 25, 2010.
Goetzel R and Ozminkowski R. The health and cost benefits of work site health-promotion programs. Annual Review of Public Health. 2008, 29, (p.303–323).
Gostin L. Public health law in a new century. Part III: Public health regulation: a systematic evaluation. JAMA (Journal American Medical Association). Vol.283, no.23, June 21, 2000, (p.3118-3122).
Gostin LO. Law as a tool to facilitate healthier lifestyles and prevent obesity. JAMA (Journal American Medical Association). Vol.297, No.1, January 3, 2007, (p.87-90).
Gostin L. General justifications for public health regulation. Public Health (Journal of the Royal Institute of Public Health). (2007) 121, (p.829-834).
Gostin L and Nass S. Reforming the HIPAA privacy rule: Safeguarding privacy and promoting research. JAMA. 2009; 301(13), (p.1373-1375).
Graaf van der J. Elementair begrip van de ethiek. Utrecht, 1980.
Guerrieri R. Toen de koekjestrommel de fruitschaal verdrong. De toxische omgeving als oorzaak van obesitas. De Psycholoog. December, 2005, (p.641-646).
Haby M, Vos T, Moodie M, Mrakwick A, Magnus A, Tay-teo K-S and Swinburn B. A new approach to assessing the health benefit form obesity interventions in children and adolescents: the assessing cost-effectiveness in obesity project. International Journal of Obesity. Vol.30, October 2006, (p.1463-1475).

Hall B. Health Incentives: The Science and Art of Motivating Healthy Behaviors. Benefits Quarterly. Second Quarter 2008; 24, 2, (p.12-22).

Hauck K, Smith P and Goddard M. The economics of priority setting for health care: a literature review. Health, Nutrition and Population (HNP) discussion paper. The International Bank for Reconstruction and Development/ The World Bank. Washington, DC, September 2004.

Have ten M, Beaufort de I, Mackenbach J en Heide van der A. An overview of ethical frameworks in public health: can they be supportive in the evaluation of programs to prevent overweight. BMC Public health 2010, 10:638 (p.1-11).

Have en M. Prevention of obesity: weighing ethical arguments. Proefschrift. Erasmus Universiteit Rotterdam, juni 2011.

Hayne C, Moran P and Ford M. Regulating environments to reduce obesity. Journal of Public Health Policy. Vol.25, No.3-4, 2004, (p.391-407).

HM Treasury. The green book. Appraisal and evaluation in central government. London, 2003.

Holtug N. The harm principle. Ethical Theory and Moral Practice. No.5, 2002, (p.357-389).

House of Lords. Select Committee on Economic Affairs. Government response to the Management of risk. 7th Report of Session 2005-06. London, United Kingdom, October 13, 2006. http://www.publications.parliament.uk/pa/ld200506/ldselect/ldeconaf/249/249.pdf (last visited: June 25, 2007)

Huse D. Obesity in the workforce: health effects and healthcare costs. Thomson Health Care, April, 2007, (p.1-4).

IBO. Gezond gedrag bevorderd. Eindrapportage van de werkgroep IBO preventie. Interdepartementaal beleidsonderzoek, 2006-2007, nr.1. Juli, 2007.

IFIC. Trends in obesity related media coverage. International Food Information Council. 2007. http://www.ific.org/research/obesitytrends.cfm (last visited: October 2007).

Intraval. Monitor naleving rookvrije werkplek 2008. Rapportage in opdracht van de Voedsel en Waren Autoriteit.

Intraval. Quick scan. Gemeenten en rookvrije horeca. Eerste jaar na invoering 2008-2009. Groningen-Rotterdam, augustus 2009.

Intraval. Inventarisatie naleefniveau rookvrije horeca. Kwartaalrapportages over de periode 'eind 2008 t/m voorjaar 2011' in opdracht van de Voedsel en Waren Autoriteit.

Kaminisky C. Verantwortung und Solidarität bei der Adipositasprävention. (Responsibility and solidarity in obesity prevention. Abstract.) Bundesgesundheitsblatt. 2009; 52, (p.527–533).

Kass N. An ethics framework for public health. American Journal of Public Health. November 2001, Vol.91, no.11, (p.1776-1782).

Keski-Rahkonen A, Hoek H. Susser E, Linna M, Sihvola E, Raevuori A, Bulik C, Kaprio J and Rissanen A. Epidemiology and course of anorexia nervosa in the community. Am J Psychiatry 2007; 164, (p.1259-1265).

Kernohan A. Accumulative harms and the interpretation of the harm principle. Social Theory and Practice. Vol.19 (1993), afl. 1 (01 03), (p.51-72).

Kersh R en Morone J. Obesity, courts, and the new politics of public health. Journal of Health Politics, Policy and Law. Vol.30, no.5, October 2005, (p.839-868).

KHN. Gewoon gastvrij. Stappenplan rookbeleid in de horeca. Koninklijke Horeca Nederland. Woerden, december 2003.

Kroes M, Mastenbroek C, Couwenbergh B, Eijndhoven van M, Festen C, en Rikken F. Van preventie verzekerd. College voor zorgverzekeringen (CVZ). Publicatienummer 250. Diemen, 16 juli 2007.
Kumanyika S. Nutrition and Chronic Disease Prevention: Priorities for US Minority Groups. Nutrition Reviews, Vol.64, no.2 (p.S9–S14).

Lake A. Obesic environments: exploring the built and food environments. The journal of the Royal Society for the Promotion of Health. Vol.126, No.6, November 2006, (p.262-267).
Lang T, Rayner G and Kaelin E. The food industry, diet, physical activity and health: a review of reported commitments and practice of 25 of the world's largest food companies. Centre for Food Policy. City University. London, April 2006.
Lee L and Gostin L. Ethical collection, storage, and use of public health data: A proposal for a national privacy protection. JAMA. 2009; 302(1), (p.82-84).
Lucht F van der, Verweij A. Sociaaleconomische gezondheidsverschillen samengevat. In: Volksgezondheid Toekomst Verkenning, Nationaal Kompas Volksgezondheid. Bilthoven: RIVM, 12 mei 2009.

MacLean L, Edwards N, Garrard M, Sims-Jones N, Clinton K and Ashley L. Obesity, stigma and public health planning. Health Promotion International, Vol.24, no.1, (p.88-93).
Maris C. The disaster of war; repression versus Dutch tolerance in drugs policy. Journal of drug issues. Jrg.29, Nr.3, 1999, (p.493-510).
Maris C. Extase. Drugs en het schadebeginsel. Justitiële verkenningen, jrg. 32, nr.1, 2006, (p.89-110).
Mastenbroek C, Meer van der F, Zwaap J, Rikken F, en Polman P. Pakketbeheer in de praktijk. College voor zorgverzekeringen (CVZ). Publicatienummer 245. Diemen, 21 december 2006.
McMenamin J and Tiglio A. Not the next tobacco: defense to obesity claims. Trends in consumer litigation. Food and drug law journal. Analyzing the laws, regulations, and policies affecting FDA-regulated products. Vol.61, No.3, 2006, (p.445-518).
Meij van der M. Roken, rookte, gerookt. Een analyse van de vormgeving en naleving van de rookverboden in de horeca. Ars Aequi. November 2009. (p.711-720).
Mello M, and Rosenthal M. Wellness programs and lifestyle discrimination – The legal limits. The New England Journal of Medicine. 359; 2, July 10, 2008, (p.192-199).
Mello M and Gostin L. Commentary: A Legal Perspective on Diabetes Surveillance – Privacy and the police power. The Milbank Quarterly. Vol.87, no.3, 2009 (p.575–580).
Mill JS. Over vrijheid. Amsterdam, 1978 (1851).
Molleman G, Peters L, Hommels L en Ploeg M. Health Promotion Effect Management Instrument. Preffi 2.0. Assessment Package. NIGZ. Woerden, 2003.
Molleman G. Preffi 2.0. Health Promotion Effect Management Instrument. Proefschrift. NIGZ. Woerden, 2005.
Molleman G, Peters L, Hosman C, Kok G, en Oosterveld P. Project quality rating by experts and practitioners experience with Preffi 2.0 as a quality assessment instrument. Health Education Research. Vol.21, no.2, 2006, (p.219-229).
MSNBC, TodayShow. Company fines workers for being overweight. The allDay weblog. August 10, 2007.

NBC5. Employer to obese employees: Shape up or pay up. Indiana company charging employers, who smoke, are obese. August 10, 2007.

Nuijten S, Schendel van M, Janssens J. Roken en rookbeleid in de sport. Meting 2006. W.J.H. Mulier Instituut. 's Hertogenbosch, mei 2007.
Nussbaum M. Political objectivity. New Literary History, 2001, 32, (p.883-906).

Ogus A. The paradoxes of legal paternalism and how to resolve them. Legal Studies. Vol.30, no.1, March 2010, (p.61-73).
Oliver E and Lee T. Public opinion and the politics of obesity in America. Journal of Health Politics, Policy and Law, Vol.30, No.5, October 2005, (p.923-954).
Online data supplement. This data supplement consists of supplemental material that, because of space constraints, was not included with the published paper by Steven D. Pearson and Sarah R. Lieber. (Published paper: Financial penalties for the unhealthy? Ethical guidelines for holding employees responsible for their health. Health Affairs. 2009).
Oortwijn W. (Rand Europe). Naar een rechtvaardige verdeling van de collectieve middelen. In: Zicht op zinnige en duurzame zorg. Achtergrondstudie uitgebracht door de Raad voor de Volksgezondheid en Zorg (RVZ) bij het advies Zinnige en duurzame zorg. Zoetermeer, 2006, (p.137-168).
Opperhuizen A, Sleijffers A, en Cremers H, (RIVM); Jacobs P, Knoll B, Borsboom W, (TNO). Zwerfrook en alternatieven voor rookruimten. RIVM. Bilthoven, 2010.
Ottes L en Rijen van A. Prioriteitenstelling in de gezondheidszorg. Een inventarisatie van de bevindingen van (regerings)commissies in binnen- en buitenland. In: Zicht op zinnige en duurzame zorg. Achtergrondstudie uitgebracht door de Raad voor de Volksgezondheid en Zorg (RVZ) bij het advies Zinnige en duurzame zorg. Zoetermeer, 2006.

Pearson D and Lieber S. Financial penalties for the unhealthy? Ethical guidelines for holding employees responsible for their health. Health Affairs. May/June 2009, Volume 28, number 3, (p.845-852).
Pope TM. Is public health paternalism really never justified. A response to Joel Feinberg. Oklahoma City University Law Review. Vol.30, No.1, spring 2005, (p.121-207).
Puhl M, and Heuer C. Obesity stigma: important considerations for public health. American Journal of Public Health. June 2010, Vol.100, no.6, (p.1019-1028).

Raad van Europa. Verdrag tot bescherming van de rechten van de mens en de fundamentele vrijheden. Rome, 04-11-1950. Geldend op 07-07-2010.
Rabin B, Boehmer T and Brownson R. Cross-national comparison of environmental and policy correlates of obesity in Europe. European Journal of Public Health. Vol.17, no.17, June 2006, (p.53-61).
Ripstein A. Beyond the harm principle. Philosophy and Public Affairs 34, no.3, (p.215-245).
Rives A. You're not the boss of me: A call for federal lifestyle discrimination legislation. The George Washington Law Review. April 2006 Vol.74, no.3, (p.553-567).
RIVM. Volksgezondheid Toekomstverkenning 2006. Dwarse thema's. Gezondheid en zorg voor welvaart. Van gezondheid naar welvaart.
Rutten F en Brouwer W. Afbakening van het basispakket: de rol van het doelmatigheidscriterium. In: Zicht op zinnige en duurzame zorg. Achtergrondstudie uitgebracht door de Raad voor de Volksgezondheid en Zorg (RVZ) bij het advies Zinnige en duurzame zorg. Zoetermeer, 2006 (p.35-88).
RVZ. Gezondheid en gedrag. Zoetermeer, 10 december 2002.
RVZ. Zinnige en duurzame zorg. Raad voor de Volksgezondheid en Zorg. Zoetermeer, 2006.

Saan H en Haes de W. Gezond effect bevorderen. Het organiseren van effectieve gezondheidsbevordering. NIGZ. Woerden, april 2005.
Schieveen van S, Waveren van A, Diepen van M. Economische effecten van het rookverbod in de horeca. Evaluatie van het eerste jaar 1 juli 2008 - 1 juli 2009. TNS/NIPO. Amsterdam, 30 september 2009.
Scholtmeijer C. Life politics: van abstracte theorie naar een bruikbaar model. Bestrijding van overgewicht in Groot-Brittannië en Nederland. Beleid en Maatschappij. Vol.33, no.4, 2006, (p.219-231).
Schulte P, Wagner G, Downes A and Miller D. A framework for the concurrent consideration of occupational hazards and obesity. The Annals of Occupational Hygiene. Vol.52, no.7, 2008, (p.555–566).
Schwartz B. The paradox of choice: why more is less. Ecco Press, 2003.
Schwartz B. The Tyranny of choice. Scientific American, April 2004, (p.70-75).
Schwartz M and Brownell K. Actions necessary to prevent childhood obesity: creating the climate for change. Journal of Law, Medicine & Ethics. Vol.35, no.1, spring 2007, (p.78-89).
Sharma A- and Padwal R. Obesity is a sign – over-eating is a symptom: an aetiological framework for the assessment and management of obesity. Obesity Reviews. Volume 11, issue 5, (p.371 – 379).
Sunstein C and Thaler R. Libertarian paternalism is not an oxymoron. The University of Chicago Law Review. Vol.70, no.4, Fall 2003, (p.1159-1202).
Swinburn B, Egger G and Raza F. Dissecting obesogenic environments: the development and application of a framework for identifying and prioritizing environmental interventions for obesity. Preventive Medicine Vol.29, 1999, (p.563-570).
Swinburn B, Egger G. Preventive strategies against weight gain and obesity. Obesity Reviews. 2002; 3 (4), (p.289-301).

Talhout R, Sleijffers A, Opperhuizen A. Handhaving van een rookvrij binnenmilieu. Briefrapport. RIVM. Bilthoven, juli 2009.
Thaler R, and Sunstein C. Libertarian Paternalism. Behavioral Economics, Public Policy, And Paternalism. Vol.93, no.2, May 2003, (p.175-179).
Toenders, W. Breedte geneesmiddelenpakket. College voor zorgverzekeringen (CVZ). Publicatienummer 01/54. Amstelveen, april 2001.

US DOL (United States Department of Labor). 29 C.F.R. §2590.702 Prohibiting discrimination against participants and beneficiaries based on a health factor. (f) Bona fide wellness programs.
US DOL (United States Department of Labor). Wellness program analysis. Health plan standards and compliance assistance. Field Assistance Bulletin No.2008-02. February 14, 2008.
USDHHS. The health consequences of involuntary exposure to tobacco smoke. A Report of the Surgeon General. U.S. Department of Health and Human Services, Centers for Disease Control and Prevention, Coordinating Center for Health Promotion, National Center for Chronic Disease Prevention and Health Promotion, Office on Smoking and Health. Atlanta, GA, 2006.
USDHHS. U.S. How tobacco smoke causes disease. The biology and behavioral basis for smoking-attributable disease. A Report of the Surgeon General. U.S. Department of Health and Human Services, Centers for Disease Control and Prevention, National Center for Chronic Disease Prevention and Health Promotion, Office on Smoking and Health. Atlanta, GA, 2010.

VN. Universele Verklaring van de Rechten van de Mens, New York, 10-12-1948. Geldend op 07-07-2010.
Vogels N, Rigter S, Dijk van P, Niesink R. De tabaksvrije coffeeshop. Gevolgen van de invoering van de rookvrije horeca voor Nederlandse coffeeshops. Trimbos Instituut. Utrecht, juli 2009.
VWS. Handleiding invoering rookvrije horeca, sport en kunst/cultuur. Ministerie van Volksgezondheid, Welzijn en Sport. Den Haag. Versie 27 juni 2008.

WHO. Global strategy on diet, physical activity and health. A framework to monitor and evaluate implementation. World Health Organization. Geneva, Switzerland, 2006.
WHO. Tobacco Free Initiative (TFI). World Health Organization. Geneva, Switzerland, 2007. http://www.who.int/tobacco
Wieringa N., Dieten H. en Eijgelshoven M. Ziektelast toegepast op het geneesmiddelenpakket. College voor zorgverzekeringen (CVZ). Volgnummer 23071747. Diemen, december 2003.
Wilson J and Jungner G. Principles and practice of screening for disease. Public Health Papers number 34. World Health Organization. Geneva, 1968.
Workforce Management, Wojcik J. Employer to fine unhealthy workers. August 2007.

Dankwoord

Het werk dat ik deed, voordat ik met het promotieonderzoek begon, bestond uit het schrijven van onderzoeks-, advies- en beleidsrapporten. Echter nooit met het doel om bij te dragen aan de wetenschap of een eigen wetenschappelijke carrière, maar altijd om een opdrachtgever verder te helpen of een maatschappelijke verbetering te bewerkstelligen. Na vijftien jaar werkervaring in de sector gezondheidszorg voelde ik de behoefte om de door mij verworven kennis en vaardigheden te integreren en vast te leggen in een boek, want rapporten voor wisselende opdrachtgevers over wisselende onderwerpen zijn vluchtig en de samenhang is niet zichtbaar voor anderen.

Dat het door mij beoogde boek een wetenschappelijk boek is geworden en de vorm heeft gekregen van een proefschrift, berust op een gelukkig toeval. Toeval omdat Gerrit van der Wal onverwacht aan mij vroeg of ik interesse had voor het uitvoeren van een promotieonderzoek. Gelukkig omdat het schrijven aan mijn proefschrift niet een achteruitkijken is geweest, maar het opdoen van veel nieuwe kennis en vaardigheden in aansluiting op de kennis en vaardigheden die ik al had. Het idee van het schrijven van een boek had ik al snel uit mijn hoofd gezet. Binnen de geneeskunde is het namelijk gebruikelijk dat men promoveert op een zestal publicaties in een internationaal wetenschappelijk tijdschrift. Tijdens het schrijven van het eerste te publiceren artikel bleek echter, dat bij het opbreken van mijn onderzoek in een aantal op zichzelf staande artikelen, de samenhang te veel verloren ging. Alle artikelen zouden op elkaar teruggrijpen en dat zou te ingewikkeld worden. In overleg met en met instemming van mijn begeleiders is toen besloten om mijn onderzoek in de vorm van een boek te publiceren.

Terugkijkend denk ik dat de ontwikkeling van het beoogde beoordelingsmodel niet mogelijk was geweest, als ik mijn werk in afzonderlijke publicaties had moeten opleveren. De Vrije Universiteit en mijn begeleiders wil ik bedanken dat ik de mogelijkheid heb gekregen om mijn proefschrift in de vorm van een boek te schrijven, zodat ik het beoogde model heb kunnen ontwikkelen. Prof. dr. Gerrit van der Wal, mijn promotor, wil ik bedanken voor zijn zeer deskundige en stimulerende begeleiding en voor zijn nauwgezetheid. Door zijn begeleiding is mijn enthousiasme voor de wetenschapsbeoefening sterk aangewakkerd. Dr. Lode Wigersma, mijn copromotor, wil ik bedanken voor zijn steun, onafhankelijke oordeelsvor-

ming en heldere logica. Door zijn begeleiding heb ik weinig tijd verspild aan tegenslagen, die zich altijd voordoen. Prof. dr. Kees Schuyt wil ik bedanken voor enkele belangrijke adviezen over de vorm en inhoud van mijn onderzoek aan het begin van het onderzoekstraject. De KNMG, mijn werkgever, en Lode Wigersma, in zijn functie als afdelingshoofd, wil ik bedanken voor hun bijdragen aan de professionalisering van het personeel in het algemeen en de totstandkoming van mijn proefschrift in het bijzonder.

Mijn ouders hebben altijd vertrouwen gehad in mijn denken. Dat is een goede basis om een wetenschappelijke zoektocht (want dat was het) met vertrouwen te beginnen en vol te houden. Dat mijn vader ook inhoudelijk geïnteresseerd was, heb ik heel leuk gevonden. Met hem praten over mijn onderzoek is behulpzaam geweest voor het formuleren van mijn bevindingen en gedachten. Mijn vrienden en collega's dank ik voor hun belangstelling, luisterend oor en adviezen, maar vooral voor de afleiding en gezelligheid, waardoor ik het lees-, denk- en schrijfwerk goed kon volhouden.

Ten slotte wil ik mijn vrouw en kinderen bedanken, dat ik zoveel tijd aan mijn proefschrift heb kunnen besteden, zonder dat zij hier ooit over hebben geklaagd. Lieve Ria, dank voor je steun en het corrigeren van het manuscript op taalfouten. Lieve Katinka en Eva, jullie niet aflatende verbazing dat ik zo lang met het lezen en schrijven over één onderwerp bezig kon zijn, gaf mij de bevestiging dat het schrijven van een proefschrift heel anders is dan alles wat ik hiervoor geschreven had.

Utrecht, najaar 2012

Curriculum vitae

Michiel Wesseling is geboren in Maassluis. Hij studeerde psychologie aan de Universiteit van Utrecht (1984-1990) en is afgestudeerd in de differentiatierichtingen klinische psychologie en organisatiepsychologie. Zijn eerste baan was organisatieonderzoeker bij het Nationaal Ziekenhuisinstituut in Utrecht (1990-1997). Hier deed hij evaluatieonderzoek naar de gevolgen van de substitutie van ziekenhuiszorg naar de eerste lijn, de deconcentratie van instellingen voor verstandelijk gehandicapten en de invoering van de Wet Bopz. Daarna werkte hij bij Twijnstra Gudde in Amersfoort (1997-1998). Als organisatieadviseur was hij betrokken bij fusies en reorganisaties van ziekenhuizen en opnieuw de deconcentratie van instellingen voor verstandelijk gehandicapten. Van 1998 tot 2000 werkte hij als zorginkoper Huisartsenzorg bij Delta Lloyd Nuts Ohra in Den Haag. In de ziekenfondsregio Den Haag werkte hij aan projecten ter uitbreiding van de Doktersnachtdiensten en verbetering van de transmurale CVA-zorg. Vanaf 2000 werkt hij bij de KNMG in Utrecht. Als beleidsmedewerker Stelsel en Kwaliteit heeft hij, in samenwerking met onder meer de federatiepartners van de KNMG, diverse standpunten ontwikkelt over de ZVW, AWBZ en medische zorg voor kwetsbare ouderen. Ook heeft hij wetenschappelijke verenigingen geadviseerd vanuit KNMG Consult. Zijn huidige functies bij de KNMG zijn projectleider Modernisering Medische Vervolgopleidingen en coördinator van Accreditatie Bureau Cluster 1. Hij is getrouwd, heeft twee dochters en woont in Utrecht.
