# Supplementary material for: Assessment model for the justification of intrusive lifestyle interventions: literature study, reasoning and empirical testing
Source: BMC Med Ethics. 2016 Feb 19;17:14. doi: 10.1186/s12910-016-0097-1 (PMC4759762; doi:10.1186/s12910-016-0097-1)
Supplement: Additional file 2: — Assessment of the intrusive prevention plan of Clarian Health 4–9 . (DOCX 18 kb) [file 12910_2016_97_MOESM2_ESM.docx]

**Additional file 2: Assessment of the intrusive prevention plan of Clarian Health**

**Description of the prevention plan**

In 2007 Clarian Health (now known as Indiana University Health) announced plans to launch a completely new approach to reduce the annual health-care costs of its employees in 2009. Clarian’s plan entailed imposing a financial penalty on its employees for leading an unhealthy lifestyle: per paycheque a fine of $10 for obesity and fines of $5 for high cholesterol, high blood pressure, high glucose levels and smoking. Clarian said it would maximize the fines at $25 per paycheque which equates to maximal $650 for an unhealthy employee per year. Clarian’s prevention plan generated much consternation in the media, with news pages and weblogs on the internet devoting an enormous amount of coverage to the plan. The numerous written responses to the prevention plan afforded an unique opportunity to test the correctness, completeness and practical applicability of the model empirically. The assessment of the Clarian Health case is based on six texts of together 19.424 words.^4-9^

**Methods of the assessment of the prevention plan**

The texts used were screened for substantive arguments for and against the preventive measure. For every argument was sought in the text for verbatim quotes that expressed the argument as good as possible. Subsequently, all selected quotations were put in a row, in the same order as in the text. For the case of Clarian Health this resulted in 312 citations. After that, per quote was judged to what criteria this quote was related: 165 of the 312 citations (53%) concerned one criterion, 99 citations (32%) concerned two criteria, 37 citations (12%) concerned three criteria, nine citations (3%) concerned four criteria, one quote (0.3% ) concerned five criteria, and one quote (0.3%) concerned seven criteria.

In the texts about the case Clarian Health were found no quotes that did not fit under any criterion. This is an indication of the completeness of the criteria. Subsequently, all quotes concerning the 1^st^ criterion were clustered (put in a row), then all quotes concerning the 2^nd^ criterion were clustered, and so on. After that, the prevention plan was assessed based on (the arguments of) the first criterion, then based on (the arguments of) the second criterion, and so on. The assessment took place in the form of an argumentative plea. Thereby it was irrelevant how often an argument was adduced, but only that an argument was adduced. The above analysis was conducted by one researcher and all (in between) results have been reviewed by two other researchers.

**Results of the assessment of the prevention plan**

***1^st^ filter: design logic***Under US law, employers may impose a 20% higher health care premium on individual employees, based on health factors, such as unhealthy behaviour (USDL, 2008). Based on this legislation, the prevention plan can pass the 1^st^ filter of the model, provided that:

- the necessity (2^nd^ criterion) of the prevention plan is better substantiated with information about the nature and extent of the financial damage of unhealthy behaviour for Clarian (1^st^ criterion ‘risk of harm’);
- the collecting, analysing and storing of health information is outsourced to an independent third party (6^th^ criterion 'suitability').

***2^nd^ Filter: effects and side-effects***The prevention plan can’t pass the 2^nd^ filter of the model. The prevention plan of Clarian includes periodic examinations of various health indicators (body mass index, cholesterol, blood pressure, glucose, smoking) among all employees. Such examinations are an impairment of physical integrity. The publicly available information is not clear about how the needed health information will be collected. Nor is it clear about how independent and secure the collected health information will be stored and for what purposes the information may and may not be used. It seems unjustifiable that even employees that meet all health criteria will be examined structurally, but more information about the assumed intrusiveness of the examinations might change this judgement. A lack of transparency about the degree of impairment of physical integrity (8^th^ criterion ‘intrusiveness’) and about the safeguarding of privacy (8^th^ criterion ‘intrusiveness’) are the reasons why the prevention plan can't pass the second filter of the model.

***3^rd^ Filter: implementation***The prevention plan can’t pass the 3^rd^ filter of the model. Prior to the implementation of the prevention plan Clarian has paid insufficient attention to acquiring support (11^th^ criterion) for the plan under its employees and in the media. In June 2007, Clarian announced its prevention plan. Already in the autumn of 2007 Clarian withdrew its plan under pressure of fierce criticism from its employees and in the media. Clarian could and should have known that its prevention plan was controversial and that the plan could evoke violent reactions from employees and in the media. Especially a controversial plan calls for good information. At this point, Clarian failed. The impact of the prevention plan for the physical integrity and privacy of employees isn't clear (see the 8^th^ criterion 'intrusiveness'). Improved information about the plan could have prevented some of the confusion and criticism: *"There's been a lot of questions and confusion"* (McGregor, 2007).

**Representativeness of texts used for the assessment**

The analysed texts on the Clarian Health case were derived from websites on which journalists and citizens had reacted to Clarion’s prevention plan. Deriving the arguments from websites, instead of collecting them by questionnaires or interviews (which could be unintentionally biased by the opinions of the researchers) contributed to the objectivity of the testing. On the internet were found far more negative than positive reactions to Clarian’s plan. The reason is probably that mostly people who were angry about the plan felt the need to respond publicly to it. This means there is a risk that the used texts form a skewed sample of the views of the entire population on the plan. For testing the correctness, completeness and practical applicability of the assessment model, this was no objection to the extent that the reactions on the internet focused on the most risky part of lifestyle interventions: the intrusion of intervention measures in people’s personal life. The reactions to Clarian’s plan were very useful in the operationalization of the criterion 'intrusiveness'. Although the smoking ban case (Additional file 3) covers a long period of 25 years, and the Clarian Health case covers a short period of about two years, the Clarian health case yielded much more information about the criterion 'intrusiveness'.
