# Supplementary material for: Assessment model for the justification of intrusive lifestyle interventions: literature study, reasoning and empirical testing
Source: BMC Med Ethics. 2016 Feb 19;17:14. doi: 10.1186/s12910-016-0097-1 (PMC4759762; doi:10.1186/s12910-016-0097-1)
Supplement: Additional file 3: — Assessment of the smoking ban in the Dutch catering sector [ 31 – 35 ] 10–14 . (DOCX 17 kb) [file 12910_2016_97_MOESM3_ESM.docx]

**Additional file 3: Assessment of the smoking ban in the Dutch catering sector**

**Description of the smoking ban**

The Dutch government imposed the statutory smoking ban on the Dutch catering sector, which entered into force on 1 July 2008. Therefore the assessment of the smoking ban was based on the official documents which the Dutch government has used to substantiate the formulation and application of the smoking ban. The literature search into the government’s reasons for introducing and enforcing the smoking ban covers a period of over 25 years (November 1984 - June 2011). The literature search comprised the meeting documents of the Dutch Parliament, publications on the Ministry of Health, Welfare and Sport website, relevant laws and regulations, and documents to which the Dutch government refers to support the introduction of the smoking ban.^10-14,27-31^

**Methods of the assessment of the prevention plan**

The vast majority of the texts on the legal smoking ban comes from meeting documents of the parliament (including committee meetings). These texts can be seen as representative of the political debate on the statutory smoking ban in the Netherlands. The written reports of the parliamentary debate are (relatively) objective, complete and skilled. The arguments of the government and parliament, the supporters and opponents of the statutory smoking ban, are carefully put in writing over a long period. The government’s online documentation of the parliamentary debate is public accessible and therewith controllable.

Not the parliamentary documents themselves, but our research activities posed the greatest risk to the objectivity. Therefore, we tried to inventory, analyze and describe all arguments as objective as possible. We summarized the arguments in our own words. Then we illustrated every argument with one or more verbatim quotes that expressed the argument as good as possible. The selection of these quotes was not based on the origin of the quotes (the persons or parties that were quoted), but on the content of the quotes (the quotes that illustrated the arguments the best). By doing so, we tried to make our research activities as transparent and controllable as possible. It was irrelevant how often or by whom an argument was adduced and only relevant that an argument was adduced. As a result, the probability of selection bias is limited.

**Results of the assessment of the smoking ban**

***1st filter: design logic***In the studied period (2008-2011) the objective of the smoking ban was the protection of all employees in the catering industry against passive smoking. Based on this objective, the smoking ban can pass the first filter of the model: the design of the smoking ban is logical. It isn’t moralistic, it isn’t perfectionistic, nor is it paternalistic.

***2nd filter: effects and side effects***Based on the objective of protecting all employees against passive smoking, the smoking ban can pass the second filter of the model, except:

- for small cafes without employees. According to the Minister of Health the protection of the visitors is a beneficial side effect, but not an objective. In that case the revenue losses are disproportionate for certain cafés without staff (10^th^ criterion "fairness").
- the implementation date of July 1, 2008. With this date the Minister of Health violates a previous agreement between the government and the catering industry that the smoking ban would be introduced no earlier than in 2009.

***3^rd^ Filter: implementation***The smoking ban can’t pass the 3^rd^ filter of the model. The government has made several severe mistakes in the implementation:

- The previously discussed implementation date of July 1, 2008 has unnecessarily undermined the support for the smoking ban in the catering industry (11^th^ criterion 'support').
- In the preparation of the implementation the government has done too little to obtain support for the smoking ban among the various stakeholders. The government has failed to carry out research into support for the smoking ban among employees in the catering industry (protecting them was the main objective) (11^th^ criterion 'support').
- In the autumn of 2010 the Minister announced that small cafes without employees would be exempted from the smoking ban in the next year. This announcement led to a huge drop in compliance with the smoking ban in all cafes. The minister should have foreseen this and should have taken action to prevent it (11^th^ criterion 'support').
- The Minister denied the reported serious revenue losses in certain small cafes without employees. This denial was not sufficiently substantiated with facts (13^th^ criterion 'verifiability').
- The Minister did not thoroughly investigate the (adverse) economic effects of the smoking ban. This is unreasonable from a scientific perspective (13^th^ criterion 'verifiability').

**Representativeness of texts used for the assessment**

The analysed texts on the smoking ban case were derived from the reports and documents belonging to the meetings of the Dutch parliament (House of Representatives and Senate, including parliamentary committees) on the development and application of the statutory smoking ban in the Dutch catering sector. These texts are representative of the political debate on this statutory smoking ban, because the parliamentary reports and documents are objective and complete and cover the arguments of the government and opposition parties and of the supporters and opponents of this smoking ban, over a long period.
