# Supplementary material for: Assessment model for the justification of intrusive lifestyle interventions: literature study, reasoning and empirical testing
Source: BMC Med Ethics. 2016 Feb 19;17:14. doi: 10.1186/s12910-016-0097-1 (PMC4759762; doi:10.1186/s12910-016-0097-1)
Supplement: Additional file 4: — Operationalization of the criteria and assessment procedure, and adjustments to the model, based on the assessment of the Clarian Health case. (DOCX 42 kb) [file 12910_2016_97_MOESM4_ESM.docx]

**Additional file 4**

**Operationalization of the criteria and assessment procedure, and adjustments to the model, based on the assessment of the Clarian Health case**

In order to be able to assess the Clarian Health case, it was necessary to operationalize the criteria as well as the assessment procedure. We show the operationalization of the criteria and assessment procedure in section 1. The Clarian Health case assessment has led to some adjustments to the model, of which we report in section 2. [a]

1. **Operationalization of the criteria and assessment procedure, based on the assessment of the Clarian Health case**

**Operationalization of the 1^st^ criterion, harmfulness**This criterion concerns the nature and extent of the risk of damage, of nuisance and of violation of moral views, which the preventive intervention is meant to combat. To establish the extent of such risks we use the definition of “risk” as operated by insurance companies: chance x outcome.

*Table 1. Extent of risks*

|  | **Small chance** | **Large chance** |
| --- | --- | --- |
| **Small damage** | Limited risk | Risk |
| **Large damage** | Risk | Severe risk |

The nature of the risks is approached as follows:

- What kind of damage, nuisance or violation of moral views is meant to be countered by the preventive intervention?
- Who are at risk to such damage, nuisance or violation of moral views? Persons with the targeted unhealthy or unsafe life style? Adults? Children?

We distinguish three categories of damage [a]:

- Health damage to oneself or others;
- Social economic damage to oneself or others;
- Infringement on personal life.

Nuisance and violation of moral views are not considered to be damage.

In the 10^th^ version of the model, “harmfulness” was withdrawn as a separate criterion, and has become part of the criterion “necessity”.

**Assessment procedure of the 1^st^ criterion, harmfulness**We assessed if the available information provides a clear picture of the nature and extent of the risks one tries to combat with the preventive intervention. Is this information correct, complete, sufficiently detailed and quantified? If the judgement is negative, it should be assessed if the gathering of information about the risks has been sufficient. Have the opportunities to inventory and quantify the risks been used exhaustively?

**Operationalization of the 2^nd^ criterion, necessity**“Necessity” deals with the question if it is necessary to combat the risks. Important issues are:

- To what extent is the preventive intervention moralistic? Does it attempt to reduce risks of damage or nuisance? Or to reduce the violation of moral views? Attempting to reduce the violation of moral views is moralistic.
- To what extent is the preventive measure an attempt to perfection? Is there a substantial risk of damage or nuisance? Or is the risk prevention exaggerated?
- Are there sanctions against persons or organizations which are partly or wholly responsible for the risks? These sanctions may indicate the necessity of prevention of the risks involved, as seen by the party who enforces sanctions.
- Doesn’t the preventive intervention replace a more useful objective of the prevention policy?

In general, a preventive intervention can be seen as necessary in case of substantial damage or nuisance for others.

**Assessment procedure of the 2^nd^ criterion, necessity**
We assess if the available information provides a clear picture of the nature and extent of the risks one tries to counter with the preventive intervention. Is this information correct, complete, sufficiently detailed and quantified? If the judgment is negative, it should be assessed if the gathering of information about the risks has been sufficient. Have the opportunities to inventory and quantify the risks been used sufficiently?

**Operationalization of the 3^rd^ criterion, causality**Causality concerns the determinants of the risks one tries to counter by means of the preventive intervention. The following questions need to be answered:

- To what extent do the lifestyles at which the preventive intervention is aimed, determine the risks the intervention tries to counter? (see also the criterion harmfulness).
- Which factors determine the lifestyles aimed at by the preventive intervention, and to what extent do they determine them?

Factors which are important in determining a certain lifestyle, are personality influences (e.g. impulsiveness, dependency, will power), health factors (e.g. genetic disposition, certain ailments), socioeconomic factors (social status, unemployment), cultural factors (e.g. ethnic dietary habits, peer group pressure) and other environmental factors.

**Assessment procedure of the 3^rd^ criterion, causality**
We assess if the available information provides a clear picture of the determinants of the risks one tries to counter with the preventive intervention, and their interaction. Is this information correct, complete, sufficiently detailed and quantified? If the judgment is negative, it should be assessed if the gathering of information about causality has been sufficient. Have the opportunities to inventory and quantify the determinants of the risks, and their interaction, been used sufficiently?

**Operationalization of the 4^th^ criterion, responsibility**Responsibility deals with the questions:

- Which actors are held responsible for the origin and the maintenance of the risks one tries to counter with the preventive intervention?
- In assessing the responsibility for these risks, have all known causes and determinants of these risks been taken into account?
- Isn’t the execution of the preventive intervention paternalistic?

The answer to these questions is determined by a combination of empirical data and ideological views. Empirical facts are the determinants of the risks one tries to counter (see under the criterion Causality), ideological views are opinions about:

- The responsibility of adults and children for their behavior, their health, their wellbeing and their environment;
- The responsibility of the government for behavior, health, wellbeing and environment of its citizens, adults and children;
- The responsibility of private parties (schools, health care institutions, businesses etc.) for behavior, health, wellbeing and environment;
- Accumulating damage (as in an obesogenic environment: accumulation of damage);
- Collective responsibility (as in collective responsibility for an obesogenic environment);
- Paternalism and its (un)desirability.

The extent to which people will be held accountable for the costs of damage is determined not only by the damage itself, but also by opinions on a just or fair distribution of charges and burdens in society. In our model, the fairness of such distribution, and also the accountability, will be addressed in the 11^th^ criterion ‘fairness’.

If we arrive at the conclusion that people are not responsible for their life-style choices, we might still want to change their behavior with an intervention. The model is meant to be neutral with regard to all criteria; also with regard to the criterion of 'responsibility'. Even we don’t know who is responsible for lifestyle choices, it makes sense to explicate this. In that case, it might be unfair (11^th^ criterion ‘fairness’) if just one party should bear all the burdens of the prevention measure.

**Assessment procedure of the 4^th^ criterion, responsibility**

- Is it clear which parties are held responsible for the risks one tries to counter?
- In assessing the responsibility for these risks, have all known causes and determinants of these risks been taken into account?
- Isn’t the execution of the preventive intervention paternalistic?

**Operationalization of the 5^th^ criterion, focus**Focus concerns the following questions:

- Is the choice of the target population and the life style influence of the chosen intervention logically correlated to the determinants of the risks one tries to combat, as well as to the responsibility for these risks?
- Are persons within the target population likely to be missed by the intervention?
- Are persons outside the target population likely to be targeted by the intervention?

The target population is defined as the group of persons in whom one tries to influence lifestyle in order to prevent lifestyle related conditions. A smoking ban in public buildings is an example of an intervention only influencing the target population, i.e. smokers in public buildings. A tax increase on alcoholic beverages is an example of an intervention not only of influence on people with problematic alcohol intake, but also people who don’t have such a problem.

**Assessment procedure of the 5^th^ criterion, focus**

- Are more logical alternatives available compared to the choice of target population and factors influencing lifestyle?
- Will too many persons within the target population be missed by the intervention?
- Will too many persons outside the target population be targeted by the intervention?

How logical should a choice be, to be fair? Scientifically it is easier to conclude that there are no convincing arguments against a theory or postulate, than it is to prove that a theory or postulate is right. Therefore, in this case we examine if more logical alternatives are possible or not, thus making the test practically workable.

In the 10^th^ version of the model, the criterion “focus”, together with the criterion “suitability”, were merged into a new criterion “appropriate design”.

**Operationalization of the 6^th^ criterion, suitability**Suitability refers to the party responsible for executing the preventive intervention. The following questions must be addressed:

- Is there a logical connection between the choice for the actor that executes the intervention, and the focus of the intervention?
- Is the responsible actor competent, qualified and suitable for this task?
- Will the execution of the intervention cause damage to the actor responsible for the execution of the intervention?

**Assessment procedure of the 6^th^ criterion, suitability**

- Would execution of the preventive intervention by another party be more logical, considering the target population and the lifestyle influencing factors in the intervention?
- Is the party that executes the intervention, competent, qualified and suitable enough?
- Will the execution of the intervention cause damage to the actor responsible for the execution of the intervention?

With regard to the assessment procedure of the 5^th^ criterion, focus, we stated that scientifically it is easier to conclude that there are no convincing arguments against a theory or postulate, than it is to prove that a theory or postulate is right. We use the same principle to assess the suitability of the preventive intervention.

**Operationalization of the 7^th^ criterion, effectiveness**Effectiveness is defined as the intended effect of the preventive intervention on the target population. Important is for which risks the intervention is effective, risk of harm to themselves or of harm to other persons. Effectiveness can be expressed as QALY’s, DALY’s or a reduction of disease burden. Side effects of the preventive intervention, either positive or negative, are not covered by the criterion effectiveness, but by the criterion “cost-benefit ratio”.

Effectiveness can be measured using process or outcome indicators. Process indicators only approximate the intended health effects, but don’t measure them. People may go to the gym but when they talk there instead of exercise, the effect is limited. Also, through exercising people may enhance their calorie intake, so the net effect on their weight is zero. This may discourage people to go to the gym. Outcome indicators are more meaningful but at the same time quite intrusive. They cover all aspects of life. When one wants to lose weight, the outcome is actual weight loss, which limits their daily life quite substantially.

Also, the durability of effects is an important point. (Puhl 2011, p 1021) [b]:

- *“… experts in the obesity field have concluded that weight regain occurs in practically all dietary and behavioral interventions, and other researchers have asserted that dieters who manage to sustain a weight loss are the rare exception, rather than the rule. Dieters who gain back more weight than they lost may very well be the norm, rather than an unlucky minority.”*

(Financial) incentives for lifestyle change may in the end lose their influence (Mello 2008, p. 198) [c]:

- *Of particular interest is whether lifestyle changes and health-risk reduction that wellness programs inspire are sustained over time. Behavioral science literature suggests that although incentives may induce behavior changes in the short term, in some cases they may also dampen intrinsic motivation, with negative long-term consequences for behavior.”*

**Assessment procedure of the 7^th^ criterion, effectiveness**
It is assessed if the preventive intervention is effective in the intended reduction of risks, and if such effects are durable. Is the information on effectiveness complete, and sufficiently detailed and quantified? If that judgment turns out negative, it is assessed if the gathering of information on effectiveness has been sufficient.

**Operationalization of the 8^th^ criterion, intrusiveness**Intrusiveness refers to the intrusion the preventive intervention causes in (targeted) persons’ personal lives. The aspects of this criterion are:

- Affection of physical integrity
- Affection of freedom;
- Affection of privacy;
- Affection of security or feeling of security;
- Discrimination;
- Stigmatization.

Questions connected to these aspects:

- Whose personal lives are being intruded?
- Who intrudes other’s personal lives?
- To what extent are personal lives being intruded?
- What is the scope of the intrusion?
- Does allowing personal life intrusion lead to precedents?
- How bothersome is intrusion on personal life?

These six questions are applicable to all six aspects of the criterion intrusiveness. So 36 questions need to be answered. Table 2 shows this.

*Table 2: assessment of intrusiveness of the preventive intervention*

| Aspect 🡪  -----------------  Question | Physical integrity | Freedom | Privacy | Security (feeling) | Discrimination | Stigmatization |
| --- | --- | --- | --- | --- | --- | --- |
| Whose? |  |  |  |  |  |  |
| By whom? |  |  |  |  |  |  |
| To what extent? |  |  |  |  |  |  |
| Scope? |  |  |  |  |  |  |
| Precedent? |  |  |  |  |  |  |
| Bothersome? |  |  |  |  |  |  |

Finally, also the degree of coercion and pressure used in executing the prevention intervention are in part decisive for the intrusiveness. Coercion and pressure are inherent to all aspects of the preventive intervention, and are therefore discussed separate from the six aspects and six questions mentioned above.

Arrangement of less to more intrusive measures might be as follows:

- Moral appeal to a person to change certain behavior;
- Positive financial incentives to reward a person for changing his behavior;
- Negative financial incentives to punish the person for not changing his behavior;
- Prohibition by law.

This order from less to more intrusive is an indication, not an absolute one. For example: an adolescent may consider his parents’ appeal to abandon alcohol, more intrusive than extra taxes on cigarettes.
The question if (targeted) persons can evade the intrusion on their personal lives is also of influence on the degree of pressure or coercion one experiences.

**Assessment procedure of the 8^th^ criterion, intrusiveness**The focus of the 8^th^ criterion is the acceptability of the balance between intrusiveness and effectiveness of the preventive intervention. Is this balance acceptable? To judge the acceptability, the falsification principle is applied. The preventive intervention passes through this criterion when the balance between intrusiveness and effectiveness is not considered unacceptable.

This judgment may best be executed in this way:

1. The intrusiveness of the intervention is assessed in general, using the six questions.
2. The six different aspects of intrusiveness are then considered in detail. Depending on the nature of the intervention, each of the six questions may be considered and weighed for each aspect. Thus, the most intrusive characteristics of the intervention pass judgment.
3. Finally, the degree of pressure and coercion involved are judged.

**Operationalization of the 9^th^ criterion, cost effectiveness**This criterion concerns the balance between the execution costs (all costs necessary to execute the intervention) and the intended effects of the intervention. If both are expressed in financial terms, cost effectiveness can be calculated by dividing the value of the intended effects by the value of the execution costs. When the outcome is larger than 1, the intervention is cost effective. The intended effects have been summed up under the criterion “effectiveness”.

The durability of the intended effects and the costs is important. It is possible that implementation of a preventive intervention is expensive at start, but getting less costly over time without a decrease in effect. This could be the case when new legislation is introduced, e.g. a legal smoking ban in hotels and restaurants: the enforcement expenses may well decrease over time.
Legislative measures may bring about considerable enforcement costs, which may be the major part of the execution costs and therefore a major determinant for cost effectiveness. Acceptability of execution costs largely depends on the support that exists for the preventive intervention. This, however, will not be judged until the criterion “support” is discussed. Nonetheless, in determining the cost effectiveness we have to realize the degree of influence that (lack of) support for the preventive intervention has on enforcement costs.

**Assessment procedure of the 9^th^ criterion, cost effectiveness**

When testing the preventive intervention against the 9^th^ criterion, it is assessed if the intervention is cost effective and how cost effectiveness develops in the course of time. Implementation of preventive interventions which are not cost effective is not justified: the money is better spent on more useful things.
It is also assessed if the information on cost effectiveness is complete and sufficiently detailed and quantified. In case this judgment turns out to be negative, it is assessed whether the gathering of information on cost effectiveness has been sufficient.

**Operationalization of the 10^th^ criterion, burdens-benefits ratio**

This criterion concerns the balance between all benefits and all burdens of the preventive intervention (table 3).

*Table 3. Burdens-benefits ratio of the intervention*

| **All burdens** | **All benefits** |
| --- | --- |
| Intrusion of personal life  Execution costs  Other burdens | Intended effects  Other benefits |

Only the “other burdens and benefits” have not been analysed yet. The burden ‘intrusion of personal life’ has already been analysed under the criterion ‘intrusiveness; the burden ‘execution costs’ under the criterion ‘cost effectiveness’, and the benefit ‘intended effects’ under the criterion ‘effectiveness’.

The reason to create a separate criterion 'effectiveness', prior to the criterion 'burdens-benefits ratio, is that the intended effects of the prevention measure (the effectiveness) constitutes the main justification for applying the prevention measure. The reason to create a separate criterion 'intrusiveness', prior to the criterion 'burden-benefit analysis', is that the infringement of personal life (the intrusiveness) is so important in case of lifestyle interventions.

When all burdens and benefits are expressed in financial terms, the burdens-benefits ratio can be calculated by dividing the value of all benefits by the value of all burdens. If this division is larger than 1, the ratio is positive. Durability of burdens and benefits is important. Arranging all burdens and benefits in one clear diagram is helpful, even if they cannot all be quantified (Ogus, 2010) [d]:

*“For the analytical framework, I propose to articulate the benefits and costs of paternalist interventions, although these will not be easy to quantify. Even in the absence of concrete figures, setting up a benefit–cost framework facilitates clear thinking about policy options. (p.69) … My benefit–cost analytical framework is not intended as a model which can easily reach definitive results, since quantification of many of the variables will be problematic. But it does suggest a useful way of ordering the relevant issues in a way which should facilitate good policy making.” (p.72)*

**Assessment procedure of the 10^th^ criterion, burdens-benefits ratio**When testing the preventive intervention against the burdens-benefits criterion, the ratio burdens to benefits of the intervention is assessed (is it favourable?) as well as its development in the course of time. Implementation of interventions with an unfavourable burdens-benefits ratio is unjustified, as the money had better be spent on other, more useful things.
It is also assessed if the available information on the burdens-benefits ratio is complete and sufficiently detailed and quantified, or, if not, if the gathering of information has been sufficient.

Theoretically it is possible that the assessment of the preventive intervention against the criteria ‘effectiveness’ and ‘intrusiveness’ turns out negative, whereas the burdens-benefits ratio test is positive. This may be the case when the favourable side effects of the intervention are very large. It is, however, not reasonable to compensate one unfavourable judgment with a favourable one if the latter one is brought about by side effects which were not intended as a result of the preventive intervention. If, because of these favourable side effects, one wants to reconsider the execution of the preventive intervention, the positive side effects should be included in the objective of the intervention, and assessment should be taken up again, starting from criterion 1.

**Operationalization of the 11^th^ criterion, fairness**This criterion deals with distributive fairness, i.e. fairness of the distribution of burdens and benefits over all parties involved in the preventive intervention. Comparison of this distribution between parties involved and parties not involved in het preventive intervention will take place under the criterion “equality of rights”.

The aspects of distributive fairness are (see references in Wesseling, 2012, p.194-196) [a]:

1. Responsibility: are the burdens of the intervention distributed according to the responsibility the parties involved bear for the risks which the preventive intervention is meant to counter?
2. Equality: are parties equally favoured or harmed by the preventive intervention, when bearing capacity is left aside?
3. Bearing capacity: does the intervention take into account physical, psychological, social and financial bearing capacity of people and organisations involved?
4. Hardship clause: does the intervention take into account circumstances of individual persons or organisations? Is there a hardship clause?
5. Equal access to health care and prevention: does the preventive intervention influence equal access?
6. Allocation of health care and prevention according to need: does the intervention influence such distribution?

These aspects have a certain overlap. This, however, does not hamper the assessment of distributive fairness of the intervention, as overlap increases the chance that all aspects of fairness are involved in the judgment.

**Assessment procedure of the 11^th^ criterion, fairness**
A review in a report of the World Bank mentions seven concepts of fairness (Hauck, 2004) [e]:

1. Egalitarianism;
2. Allocation according to need;
3. Rule of rescue;
4. Equality of access;
5. Providing a decent minimum;
6. Rawls’ maximin principle;
7. Libertarianism.

In science and society there is no consensus how these aspects should be weighed against each other when judging the distribution of burdens and benefits of preventive interventions. The weight that is attached, on moral, ideological or political grounds, to different aspects of fairness leads to different outcomes. Generally it is easier to establish that no convincing arguments exist against a statement or theory, than to argue convincingly that the statement or theory is correct. This principle is used in the assessment of distributive fairness of the preventive intervention: is execution of the preventive intervention not evidently dishonest or unfair from a perspective of distributive fairness?

**Operationalization of the 12^th^ criterion, equality of rights**This criterion deals with the comparison between burdens and benefits for parties involved in the preventive intervention, and burdens and benefits for parties not involved. Does the preventive intervention cause inequality of rights between these two parties?

Some examples:

- inequality of rights exists when for health reasons alcohol use is prohibited, but tobacco use isn’t;
- inequality of rights exists when smoking leads to a higher health insurance premium, but alcohol abuse doesn’t;
- inequality of rights exists when severe obesity leads to a higher health insurance premium, but severe undernourishment doesn’t;
- inequality of rights exists when those addicted to gambling may not enter a casino, but those addicted to alcohol may enter cafes.

**Assessment procedure of the 12^th^ criterion, equality of rights**The method of assessment of the criterion ’equality of rights’ is the same as that of the criterion ‘fairness’.

**Operationalization of the 13^th^ criterion, support**The criterion ‘support’ concerns the following questions:

- To what extent and in what way(s) is focused on gaining support for prevention measures?
- How much support is there for the prevention measure?

**Assessment procedure of the 13^h^ criterion, support**The criterion ‘support’ concerns the support from all parties involved and in any case of the:

- target group on which the preventive measure is aimed;
- stakeholders that are protected by the preventive measure;
- organizations that are carrying out, or enforcing the prevention measure.

If relevant, the criterion ‘support’ also concerns public support. Depending on the content of the prevention measure different parties determine public support (for example): the general population, pressure groups, employees, employers, business life, shareholders, government, politics, science, media, people with an unhealthy lifestyle among the aforementioned parties, etc.

It is assessed whether there is sufficient support of the parties involved in the preventive measure, so that the enforcement and implementation are not unduly difficult or expensive. From this perspective, it is also assessed whether sufficient attention is paid to acquiring support for the prevention measure.

**Clarian Health case offers hardly information about the criterion ‘implementation capacity’**The operationalization of the preceding criteria is for an important part based on the collected texts on the prevention plan of Clarian Health (Additional file 2). Because Clarian’s prevention plan has not been implemented, there is little information in these texts about the criterion ‘implementation capacity’. The content of the criterion ‘implementation capacity’ is further elaborated in the assessment of the case 'statutory smoking ban in the catering sector ' (Additional file 3).

**Operationalization of the 14^th^ criterion, implementation capacity**The implementation capacity concerns the feasibility and sustainability of the application of the prevention measure.

**Assessment procedure of the 14^h^ criterion, support**Can reasonably be assumed that a sufficient number of people and funds have been made available to ensure that implementation of the prevention measure is feasible and sustainable?

1. **Adjustments to the model, based on the assessment of the Clarian Health case**

The assessment of the case ‘Intrusive prevention plan of Clarion health’ was based on the 6^th^ version of the model that consisted of 3 filters and 14 criteria:

1. **Design logic (filter):**
2. harmfulness;
3. necessity;
4. causality;
5. responsibility;
6. focus;
7. suitability;
8. **Effects and side effects (filter):**
9. effectiveness;
10. intrusiveness;
11. cost effectiveness;
12. burdens-benefits-ratio;
13. fairness;
14. equality of rights;
15. **Implementation (filter):**
16. support;
17. implementation capacity.

The assessment of the Clarian Health case led to the following additions and adjustments to the 6^th^ version of the model (Wesseling, 2012, p.224) [a]:

- Operationalization of the criteria and assessment procedure (see section 1);
- The theoretical distinction between distributive fairness (criterion ‘fairness’) and comparative justice (criterion ‘equality of rights’) wasn’t useful in assessing the case. Therefore the criteria ‘fairness’ and ‘equality of rights’ were merged to one criterion ‘fairness’ in the 7^th^ version of the model.
- The theoretical distinction between cost effectiveness (9^th^ criterion) and burdens-benefits ratio (10^th^ criterion) wasn’t useful in assessing the case. Therefore the criteria ‘cost effectiveness’ and ‘burdens-benefits ratio’ were merged to one criterion ‘burdens-benefits ratio’ in the 7^th^ version of the model.
- In the 7^th^ version of the model, a criterion ’timing’ was added to the 3^rd^ filter (implementation), prior to the criterion ‘support’. This criterion was operationalized while assessing the case ‘smoking ban’ (see supplement 2).
- In the 7^th^ version of the model, a criterion ’complementary policies’ was added to the 3^rd^ filter (implementation), prior to the criterion ‘implementation capacity’. This criterion was operationalized while assessing the case ‘smoking ban’ (see supplement 2).

**References in this Supplement**

1. Wesseling M. Justification of interventions to influence lifestyle. A value-neutral assessment model. Dissertation. VU University Amsterdam. Amsterdam: VU University Press; Dec 5, 2012. Summary in English (p.515-535). Publication in Dutch: Gerechtvaardigdheid van interventies ter beïnvloeding van leefstijl. Een waardeneutraal beoordelingsmodel.
2. Puhl M, and Heuer C. Obesity stigma: important considerations for public health. American Journal of Public Health. June 2010, Vol.100, no.6, (p.1019-1028).
3. Mello M, Rosenthal M. Wellness programs and lifestyle discrimination - The legal limits. N Engl J Med. 2008;359:192-199.
4. Ogus A. The paradoxes of legal paternalism and how to resolve them. Legal Studies. Vol.30, no.1, March 2010, (p.61-73).
5. Hauck K, Smith P and Goddard M. The economics of priority setting for health care: a literature review. Health, Nutrition and Population (HNP) discussion paper. The International Bank for Reconstruction and Development/ The World Bank. Washington, DC, September 2004.
