# Supplementary material for: Assessment model for the justification of intrusive lifestyle interventions: literature study, reasoning and empirical testing
Source: BMC Med Ethics. 2016 Feb 19;17:14. doi: 10.1186/s12910-016-0097-1 (PMC4759762; doi:10.1186/s12910-016-0097-1)
Supplement: Additional file 5: — Operationalization of the criteria and assessment procedure, and adjustments to the model, based on the assessment of the Smoking Ban case. (DOCX 26 kb) [file 12910_2016_97_MOESM5_ESM.docx]

**Additional file 5**

**Operationalization of the criteria and assessment procedure, and adjustments to the model, based on the assessment of the Smoking Ban case**

Assessment of the Clarian Health case with aid of the model, led to two additional criteria (see section 2 of supplement 1):

- timing (was added to the 3^rd^ filter ‘implementation’);
- complementary policies (was added to the 3^rd^ filter ‘implementation’).

In order to be able to assess the Smoking Ban case (see additional file 3), it was necessary to operationalize these new criteria as well as the corresponding assessment procedures. We show this in section 1. The assessment of the Smoking Ban case has led to some adjustments to the model, of which we report in section 2. [a]

1. **Operationalization of the criteria and assessment procedure, based on the assessment of the Smoking Ban case**

**Operationalization of the new criterion, timing**The criterion timing of implementation concerns the following questions:

- To what extent is the time ripe to pursue the targeted lifestyle change by applying pressure or coercion?
- To what extent are people and organizations, who are affected by the preventive measure, prepared gradually to the prevention measure?
- To what extent are the current political, economic and social conditions suitable for implementation?
- To what extent have people and organizations, who are affected by the preventive measure, time to adapt to the new rules?

**Assessment procedure of the new criterion, timing**Assessed is whether the timing of the implementation is not unreasonable or unfair.

**Operationalization of the new criterion, complementary policies**Complementary policies are policies designed to support the implementation of a measure or law. This can be either through incentives (e.g. encouraging support for the smoking ban by informing the population about the harmful effects of passive smoking) and by removing barriers (for example, flexible regulations of municipalities, when bars try to obtain a license for a terrace).

In the assessment of supporting policies, it is recommended to pay attention at the following aspects:

- the embedding of the prevention measure in broader prevention policy;
- information on the prevention measure to the parties involved;
- obstacles that hinder the implementation of the prevention measure;
- problems as a result of the prevention measure;
- enforcement of the compliance of the prevention measure.

**Assessment procedure of the new criterion, complementary policies**Assessed is whether:

- the design and implementation of the supplementary policy are not unreasonable or unfair;
- there is no carelessness or negligence in the design and implementation of the supplementary policy.

1. **Adjustments to the model, based on the assessment of the Smoking ban case**

The (initial) assessment of the case ‘smoking ban’ was based on the 7^th^ version of the model that consisted of 3 filters and 14 criteria:

1. **Design logic (filter):**
2. harmfulness;
3. necessity;
4. causality;
5. responsibility;
6. focus;
7. suitability;
8. **Effects and side effects (filter):**
9. effectiveness;
10. intrusiveness;
11. burdens-benefits-ratio;
12. fairness;
13. **Implementation (filter):**
14. timing
15. support;
16. complementary policies;
17. implementation capacity.

The assessment of the Smoking Ban case led, inter alia, to the following additions and adjustments to the model (Wesseling, 2012, p.450-451) [a]:

- operationalization of the new criteria (timing and complementary policies) and corresponding assessment procedures (see section 1);
- the distinction between the criteria ‘timing’ and ‘support’ wasn’t useful in assessing the case. Therefore these criteria were merged to one criterion ‘support’ in the 9^th^ version of the model.
- in the 8^th^ version of the model, a criterion ’verifiability’ was added to the 3^rd^ filter ‘implementation’ prior to the criterion ‘implementation capacity’ (see the 10^th^ version of the model in the manuscript);
- the operationalization of the ‘optimization principle’ for the assessment of the criteria of the 3^rd^ filter ‘implementation: no carelessness and no negligence concerning the criteria.

**References in this Supplement**

1. Wesseling M. Justification of interventions to influence lifestyle. A value-neutral assessment model. Dissertation. VU University Amsterdam. Amsterdam: VU University Press; Dec 5, 2012. Summary in English (p.515-535). Publication in Dutch: Gerechtvaardigdheid van interventies ter beïnvloeding van leefstijl. Een waardeneutraal beoordelingsmodel.
